# Bimetallic Ru/Ru‐Catalyzed Asymmetric One‐Pot Sequential Hydrogenations for the Stereodivergent Synthesis of Chiral Lactones

**DOI:** 10.1002/advs.202400621

**Published:** 2024-03-21

**Authors:** Jingli He, Zhaodi Li, Ruhui Li, Xuezhen Kou, Delong Liu, Wanbin Zhang

**Affiliations:** ^1^ Shanghai Key Laboratory for Molecular Engineering of Chiral Drugs School of Pharmacy Shanghai Jiao Tong University 800 Dongchuan Road Shanghai 200240 China; ^2^ Frontiers Science Center for Transformative Molecules School of Chemistry and Chemical Engineering Shanghai Jiao Tong University 800 Dongchuan Road Shanghai 200240 China

**Keywords:** asymmetric sequential hydrogenations, bimetallic catalytic system, chiral lactone, RuPHOX‐Ru, stereodivergent synthesis

## Abstract

Asymmetric sequential hydrogenations of *α*‐methylene *γ*‐ or *δ*‐keto carboxylic acids are established in one‐pot using a bimetallic Ru/Ru catalyst system, achieving the stereodivergent synthesis of all four stereoisomers of both chiral *γ*‐ and *δ*‐lactones with two non‐vicinal carbon stereocenters in high yields (up to 99%) and with excellent stereoselectivities (up to >99% ee and >20:1 dr). The compatibility of the two chiral Ru catalyst systems is investigated in detail, and it is found that the basicity of the reaction system plays a key role in the sequential hydrogenation processes. The protocol can be performed on a gram‐scale with a low catalyst loading (up to 11000 S/C) and the resulting products allow for many transformations, particularly for the synthesis of several key intermediates useful for the preparation of chiral drugs and natural products.

## Graphical abstract

Asymmetric sequential hydrogenations of *α*‐methylene *γ*‐ or *δ*‐keto carboxylic acids are established in one‐pot using a bimetallic Ru/Ru catalyst system, achieving the stereodivergent synthesis of all four stereoisomers of both chiral *γ*‐ and *δ*‐lactones in high yields (up to 99%) and with excellent stereoselectivities (up to >99% ee and >20:1 dr).

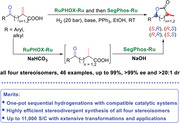

## 1 Introduction

Different stereoisomers of chiral compounds bearing multiple stereocenters usually exhibit different and even opposite therapeutic effects due to the chiral environment provided by enzymes and receptors in vivo.^[^ ^1^ ^]^ The construction of all possible stereoisomers of a multi‐chiral compound is certainly an essential step in the process of drug discovery, development, and production, and also an important research topic in the field of asymmetric catalysis.^[^ ^2^, ^3^, ^4^, ^5^, ^6^, ^7^ ^]^ The stereodivergent synthesis of all these isomers via a one‐pot process using multiple catalytic systems represents the most straightforward and efficient approach to fulfill this goal.^[^ ^3^ ^]^ Among them, chiral bimetallic synergistic catalysis is demonstrated to play an important role in the mentioned stereodivergent synthesis, in which two substrates are activated and controlled by separate catalysts, leading to the creation of two stereocenters in the products simultaneously during new bond formation.^[^ ^4^, ^5^ ^]^ Recently, chiral bimetallic sequential catalysis has also gained significant attention. In this approach, each reaction can be stereoselectively controlled by distinct chiral metal catalysts sequentially, allowing for greater flexibility in substrate design and a broader range of options for catalytic systems.^[^ ^6^ ^]^ In order to further advance our research in chiral bimetallic catalysis, we are focusing on chiral bimetallic two‐step‐sequential catalysis with the aim of expanding the potential applications of stereodivergent synthesis. Unlike synergistic catalysis, where two substrates are typically activated by two different metal catalysts simultaneously, the catalytic systems for the preceding and subsequent reactions in one‐pot chiral bimetallic two‐step‐sequential catalysis can be particularly prone to interfere with each other. Stereodivergent synthesis in this area has mainly employed different kinds of reactions with distinct catalyst system, but even so, it has been severely restricted.^[^ ^7^ ^]^ The pursuit of mutually compatible catalytic systems for sequential reactions, particularly those involving identical reaction types, presents a highly valuable but undoubtedly challenging avenue of research.

**Figure 1:**
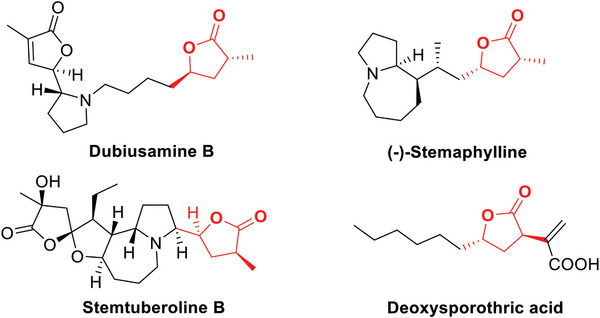
Several chiral *α*,*γ*‐disubstituted *γ*‐lactones.

Chiral lactone skeletons with two non‐vicinal carbon stereocenters are vital structural motifs in a variety of natural products and bioactive molecules,^[^ ^8^ ^]^ such as chiral *α*,*γ*‐disubstituted *γ*‐lactone dubiusamine B,^[^ ^8b^ ^]^ (‐)‐stemaphylline,^[^ ^8c^ ^]^ stemtuberoline B,^[^ ^8e^ ^]^ and deoxysporothric acid,^[^ ^8f^ ^]^ etc. (**Figure** **1**). They are also important synthons in the preparation of a series of key intermediates for natural products and drugs.^[^ ^9^ ^]^ Moreover, their biological activities have proved to be closely dependent on their absolute and relative stereochemical configurations.^[^ ^8^, ^9^ ^]^ Therefore, the efficient construction of all possible stereoisomers of chiral lactones is a worthwhile endeavor but has received little attention.^[^ ^10^ ^]^

**Scheme 1:**
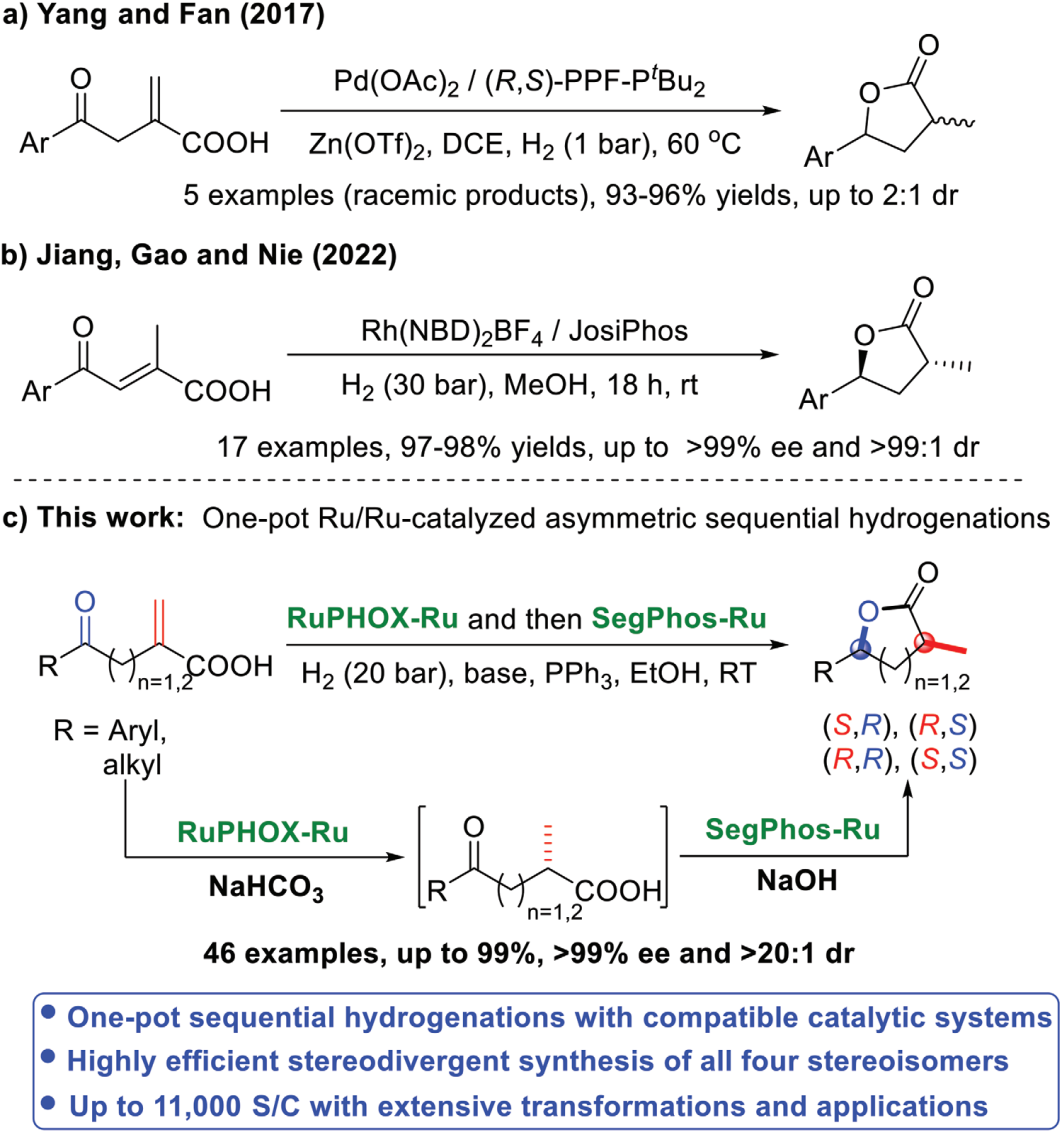
Synthesis of chiral *γ*‐ and *δ*‐lactones via asymmetric hydrogenation.

Transition‐metal‐catalyzed asymmetric hydrogenation represents one of the most efficient, environmentally friendly, and cost‐effective approaches to various chiral compounds.^[^ ^11^ ^]^ The construction of chiral lactones with two non‐vicinal carbon stereocenters via this strategy is no doubt one of the most practical pathways.^[^ ^12^ ^]^ In 2019, Fan's group reported the chiral Pd(II)/Zn co‐catalyzed chemoselective hydrogenation of *α*‐methylene‐*γ*‐keto carboxylic acids, providing racemic *α*,*γ*‐disubstituted *γ*‐lactones in high yields but with poor diastereoselectivities (**Scheme** **1**a).^[^ ^12a^ ^]^ Recently, Nie and coworkers disclosed the Rh‐catalyzed asymmetric cascade hydrogenations of (*E*)‐2‐methyl‐4‐oxo‐2‐alkenoic acids to afford chiral *α*,*γ*‐disubstituted *γ*‐lactones with excellent results (Scheme 1b).^[^ ^12b^ ^]^ However, achieving stereodivergent synthesis of all four stereoisomers of chiral lactones through a single‐catalyst cascade reaction remains highly elusive.^[^ ^12^ ^]^ Herein, we report the base‐mediated bimetallic Ru/Ru‐catalyzed asymmetric sequential hydrogenations of *α*‐methylene *γ*‐ or *δ*‐keto carboxylic acids via a one‐pot process, for the stereodivergent synthesis of all four stereoisomers of both chiral *γ*‐ and *δ*‐lactones with two non‐vicinal carbon stereocenters in high yields with excellent enantio‐ and diastereoselectivities (Scheme 1c).

## 2 Results and Discussion

The compatibility of the two catalyst systems was investigated since it determines whether our sequential hydrogenations are feasible. First of all, the chemoselective hydrogenation of C═C and C═O double bonds of 2‐methylene‐4‐oxo‐4‐phenylbutanoic acid (**1a**) should be conducted, a process that has only been discussed using expensive chiral Rh‐catalyst systems for the hydrogenation of C═C double bond.^[^ ^13^ ^]^ Our group has previously developed the planar chiral RuPHOX‐Ru catalyst which has been successfully applied to asymmetric hydrogenations of substrates bearing C═C and/or C═O double bonds.^[^ ^14^ ^]^ In this context, the reduction of the aforementioned double bonds can be achieved under mild reaction conditions by using bases with different alkalinity. We therefore applied this chiral (*S*,*S*_p_)‐Ru‐catalyst to the asymmetric hydrogenation of **1a** with the aim of achieving the chemo‐ and enantioselective hydrogenation of the two types of double bonds (**Table** **1**). After optimizing the reaction conditions (see details in the Supporting Information), we found that the chemo‐ and enantioselective hydrogenation of the C═C double bond of **1a** could be realized successfully when the reaction was carried out in the presence of a weak inorganic base such as NaHCO_3_, affording the corresponding chiral 2‐methyl‐4‐oxo‐4‐phenylbutanoic acid (**2a**) with excellent results (Table 1, entry 1). It was found that PPh_3_ plays a significant role in the reaction and **2a** was obtained with a slightly lower ee of 92% when the above reaction was conducted in the absence of PPh_3_ (entry 2). If NaHCO_3_ was replaced by a stronger inorganic base, such as KHCO_3_, Na_2_CO_3_, NaOH, or KOH, the selective hydrogenated product **2a** could also be obtained but with somewhat inferior enantioselectivities (entries 3–6). To assess the possibility of further asymmetric hydrogenation of the C═O bond, we conducted the same experiment under 50 bar hydrogen pressure (entries 7 and 8, see details in the Supporting Information). To figure out whether or not the further asymmetric hydrogenation of C═O double could realize, the above examination was also carried out under 50 bar hydrogen pressure (entries 7 and 8, see details in the Supporting Information). It was found that the subsequent (*S*,*S*_p_)‐RuPHOX‐Ru‐catalyzed asymmetric hydrogenation of the C═O double bond of **2a** was possible in the presence of the strong base KOH, with (*3S*,*5R*)‐3‐methyl‐5‐phenyldihydrofuran‐2(3*H*)‐one (**3a**) being obtained in 99% ee but with only 2:1 dr and 12% yield. The results disclosed that the efficient (*S*,*S*_p_)‐RuPHOX‐Ru‐catalyzed chemoselective hydrogenation of the C═C double bond is achievable when the reaction is carried out with a weak base such as NaHCO_3_ and the following asymmetric hydrogenation of the C═O double bond might be realized with a stronger base using a more suitable chiral Ru‐catalyst for the efficient synthesis of **3a**. Finally, the reaction time was also examined and it was found that the hydrogenation of the C═C double bond of **1a** was completed within 6 h (entries 9–11).

**Table 1:** Reaction optimization of the asymmetric hydrogenation of the C═C bond.

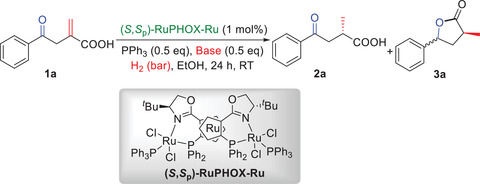					
Entry^a)^	Base	H_2_ [bar]	Yield [%] of 2a/3a^b)^	ee [%] of 2a/3a^c)^	dr of 3a^b)^
1	NaHCO_3_	20	99/‐	>99/‐	‐
2^d)^	NaHCO_3_	20	99/‐	92/‐	‐
3	KHCO_3_	20	99/‐	96/‐	‐
4	Na_2_CO_3_	20	99/‐	95/‐	‐
5	NaOH	20	99/‐	74/‐	‐
6	KOH	20	99/‐	68/‐	‐
7	NaHCO_3_	50	99/‐	>99/‐	‐
8	KOH	50	88/12	90/99, 20	2.0:1
9^e)^	NaHCO_3_	20	99/‐	>99/‐	‐
10^f)^	NaHCO_3_	20	99/‐	>99/‐	‐
11 ^g)^	NaHCO_3_	20	96/‐	>99/‐	‐

^a)^ Reaction conditions: **1a** (19.0 mg, 0.1 mmol), (*S,S*_P_)‐RuPHOX‐Ru (1.7 mg, 1 mol%), PPh_3_ (13.2 mg, 0.5 equiv), base (0.5 equiv), H_2_ (bar), EtOH (0.5 mL), room temperature, 24 h;

^b)^ Determined by ^1^H NMR with 1,3,5‐trimethylbenzene as an internal standard;

^c)^ Determined by HPLC using a Chiralpak AD‐H column (**2a**) or Chiralcel OD‐H (**3a**) column;

^d)^ In the absence of PPh_3_;

^e)^ Reaction time: 12 h;

^f)^ Reaction time: 6 h;

^g)^ Reaction time: 3 h.

We then carried out the asymmetric hydrogenation of the C═O double bond of **2a** with different chiral catalysts and bases under the above optimal reaction conditions (**Table** **2**). After optimizing the reaction conditions (see details in the Supporting Information), RuCl_2_[(*S*)‐(DM‐SegPhos)][(*S*)‐DAIPEN] (abbreviated as “(*S*,*S*)‐SegPhos‐Ru”),^[^ ^15^ ^]^ which could be obtained commercially, presented the best results when 2.0 equiv of NaOH were used (Table 2, entry 1). To our delight, the use of PPh_3_ had no influence on the reaction (entry 2). Lowering the amount of NaOH to 1.5 equiv resulted in a lower reaction activity, with **3a** being obtained in only 67% yield with 99% ee and 14:1 dr (entry 3). To our delight, hydrogenation did not occur when 2.0 equiv of NaHCO_3_ were used (entry 4). These results suggested that the hydrogenation of the C═O double bond will not be affected by the previous (*S*,*S*_p_)‐RuPHOXRu catalyst system and that sequential hydrogenations should be possible using the two mutually compatible catalyst systems.

**Table 2:** Reaction optimization of the asymmetric hydrogenation of the C═O bond.

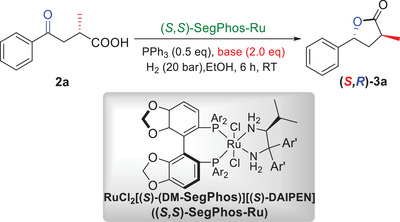				
Entry^a)^	Base	Yield [%]^b)^	ee [%]^c)^	dr^b)^
1	NaOH	99	>99	>20:1
2^d)^	NaOH	99	>99	>20:1
3^e)^	NaOH	67	99	14:1
4	NaHCO_3_	no reaction	‐	‐

^a)^ Reaction conditions: **2a** (19.2 mg, 0.1 mmol), (*S*,*S*)‐Segphos‐Ru (1.2 mg, 1 mol%), PPh_3_ (13.2 mg, 0.5 equiv), base (2.0 equiv), H_2_ (20 bar), EtOH (0.5 mL), room temperature, 6 h;

^b)^ Determined by ^1^H NMR with 1,3,5‐trimethylbenzene as an internal standard;

^c)^ Determined by HPLC using a Chiralcel OD‐H column;

^d)^ In the absence of PPh_3_;

^e)^ NaOH (1.5 equiv).

**Scheme 2:**
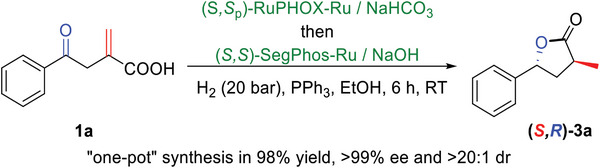
Asymmetric sequential hydrogenations of the C═C and C═O bonds of **1a**.

The bimetallic (*S*,*S*_p_)‐RuPHOX‐Ru/(*S*,*S*)‐SegPhos‐Ru‐catalyzed asymmetric sequential hydrogenations were therefore carried out easily in EtOH under 20 bar hydrogenation pressure at room temperature (**Scheme** **2**). We were pleased to discover that the desired product (*S*,*R*)‐**3a** could be obtained in 98% yield with >99% ee and >20:1 dr.

**Scheme 3:**
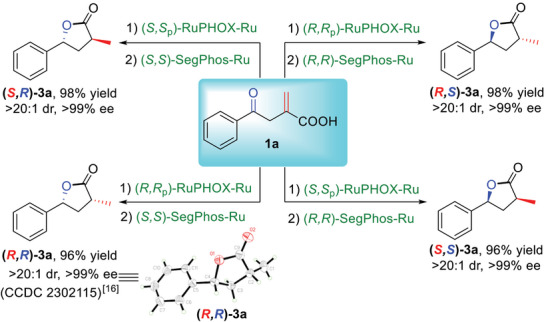
Stereodivergent access to all stereoisomers of **3a**.

Next, the feasibility of the enantio‐ and diastereodivergent preparation of chiral *γ*‐lactones **3a** was investigated. The C═C double bond of **1a** was first hydrogenated utilizing RuPHOX‐Ru, followed by the SegPhos‐Ru catalyzed asymmetric hydrogenation of the C═O double bond of **2a** to afford **3a** stereodivergently in high yields with >99% ee and >20:1 dr. Obviously, the synthesis of all four stereoisomers of **3a** could be achieved easily by simply changing the chiral catalyst combinations under otherwise identical optimized conditions (**Scheme** **3**).

**Scheme 4:**
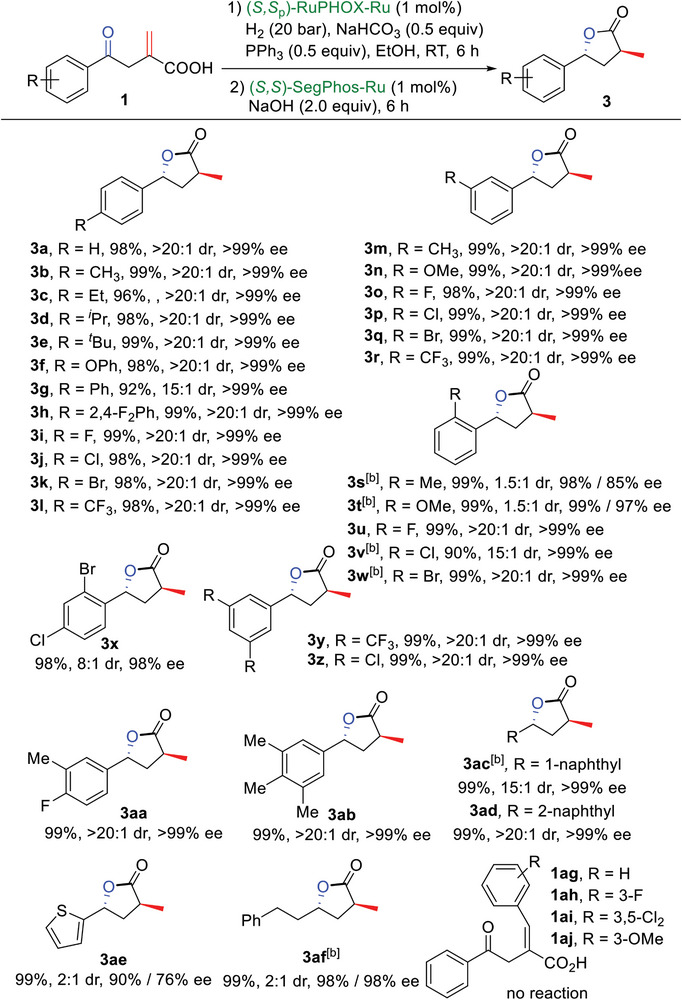
Substrate scope of **1**. ^[a]^ Reaction conditions: **1** (0.2 mmol), (*S,S*_p_)‐RuPHOX‐Ru (3.4 mg, 1 mol%), H_2_ (20 bar), NaHCO_3_ (8.4 mg, 0.5 equiv), PPh_3_ (26.3 mg, 0.5 equiv) and EtOH (2 mL) at room temperature for 6 h; then (*S,S*)‐SegPhos‐Ru (2.4 mg, 1 mol%) and NaOH (16.0 mg, 2.0 equiv) was added to the reaction mixture and stirred for another 6 h; isolated yields; ees were determined by HPLC; drs were determined by ^1^H NMR analysis; the absolute configurations of **3** are assigned to (*R*,*R*)‐**3a** which was determined by X‐ray analysis; ^[b]^ H_2_ (50 bar) in the second step.

Next, the substrate scope of *α*‐methylene *γ*‐keto carboxylic acids (**1**) was investigated using the above optimal reaction conditions (**Scheme** **4**). First, the hydrogenation of **1a‐r** bearing either an electron‐donating or an electron‐withdrawing group at the *para‐* or *meta‐*position of the phenyl ring was carried out. It was shown that these substrates were all tolerated in the sequential hydrogenations, affording the desired chiral *γ*‐lactone products (**3a‐r**) in high yields (92–99%) with excellent enantio‐ (>99% ees) and diastereoselectivities (15:1→20:1 dr). Substrates with an electron‐donating group (Me or OMe) at the *ortho‐*position of the phenyl ring were also examined. The target lactone products **3s** and **3t** were obtained in high yields and with excellent enantioselectivities, but with only 1.5:1 dr. This can be ascribed to unsatisfactory stereoselectivity in the latter (*S*,*S*)‐SegPhos‐Ru catalyzed hydrogenation of the C═O double bond (see details in the Supporting Information). When substrates bearing an electron‐withdrawing group (F, Cl, or Br) at the *ortho*‐position of the phenyl ring were used in the above reaction, the corresponding lactone products **3u‐w** could also be obtained in yields of 90–99% with >99% ees and 15:1→20:1 drs. Next, aromatic *α*‐methylene‐*γ*‐keto carboxylic acids containing disubstituted and trisubstituted groups on the phenyl ring were also explored. Compounds **1x‐z** bearing two electron‐withdrawing groups were hydrogenated smoothly to afford the corresponding products (**3x‐z**) in high yields with excellent enantio‐ and diastereoselectivities. Compound **1aa**, containing both electron‐withdrawing and electron‐donating groups on the phenyl ring, could also be catalyzed to provide the desired product **3aa** in 99% yield with >99% ee and >20:1 dr. To our delight, **1ab** possessing three Me groups also yielded the desired product **3ab** with excellent catalytic behavior. Notably, when **1ac** and **1ad** bearing a 1‐ or 2‐naphthyl group were subjected to the sequential hydrogenations, the target products **3ac** and **3ad** were also obtained in high yields with excellent enantio‐ and diastereoselectivities.

Heteroaromatic **1ae** and aliphatic **1af** were also examined in the sequential hydrogenations. It was found that the desired lactone products **3ae** and **3af** could be obtained in 99% yield and with excellent enantioselectivities but only 2:1 drs. Finally, several *γ*‐keto carboxylic acids with an aryl‐substituted methylene (**1ag‐aj**) were synthesized successfully.^[^ ^13c^ ^]^ Unfortunately, the asymmetric one‐pot sequential hydrogenations of these *γ*‐keto carboxylic acids bearing an aryl‐substituted methylene were unsuccessful. This work demonstrates that chiral bimetallic asymmetric sequential hydrogenations offer a highly efficient and versatile approach for the synthesis of *α*‐methyl chiral *γ*‐lactones.

**Scheme 5:**
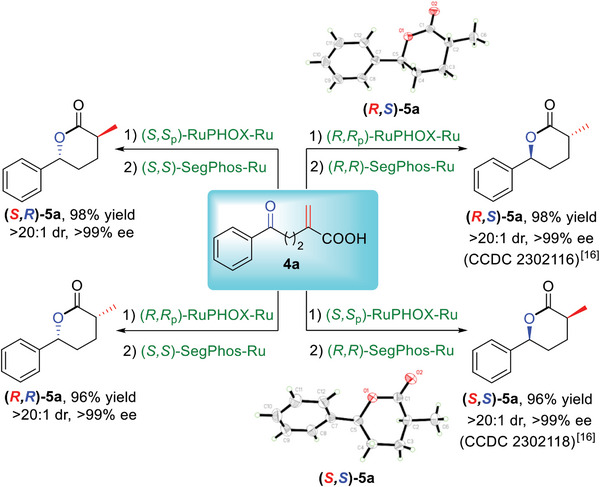
Stereodivergent access to all stereoisomers of 5a.

Chiral *δ*‐lactones with non‐vicinal carbon stereocenters are also important structural motifs in a variety of natural products, pharmaceuticals and biologically active compounds.^[^ ^16^ ^]^ However, no research concerning the construction of such skeletons via the asymmetric hydrogenation of *α*‐methylene *δ*‐keto carboxylic acids (**4**) has been reported. Inspired by our efficient sequential hydrogenations of *α*‐methylene *γ*‐keto carboxylic acids (**1**), we carried out the bimetallic Ru/Ru‐catalyzed asymmetric sequential hydrogenations of the corresponding *δ*‐counterparts **4** under the optimal reaction conditions. The feasibility of the enantio‐ and diastereodivergent approach to chiral *δ*‐lactone **5a** was investigated first (**Scheme** **5**). Following an identical reaction procedure to that of the stereodivergent synthesis of **3a** (Scheme 3), all four stereoisomers of **5a** were obtained in high yields with >99% ee and >20:1 dr by simply changing the chiral catalyst combinations under otherwise identical conditions.

**Scheme 6:**
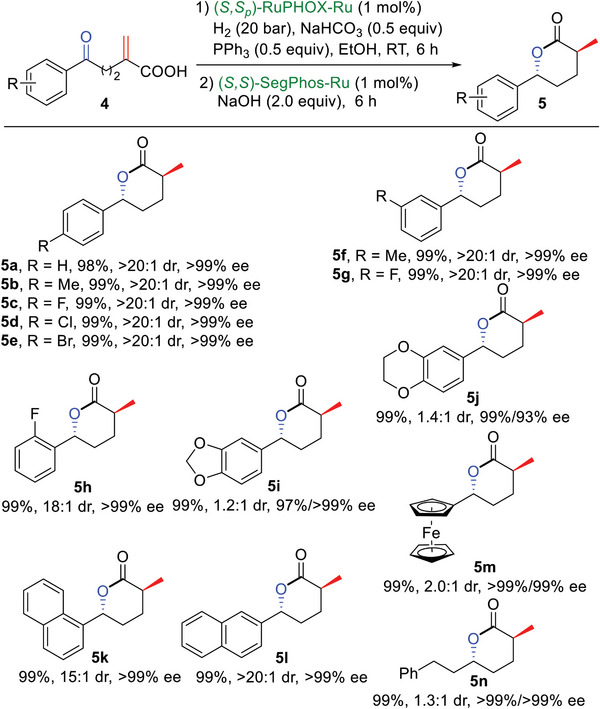
Substrate scope of **4**.^[a]^ Reaction conditions: **4** (0.2 mmol), (*S,S*_p_)‐RuPHOX‐Ru (3.4 mg, 1 mol%), H_2_ (20 bar), NaHCO_3_ (8.3 mg, 0.5 equiv), PPh_3_ (26.3 mg, 0.5 equiv) and EtOH (2 mL) at room temperature for 6 h; then (*S,S*)‐SegPhos‐Ru (2.4 mg, 1 mol%) and NaOH (16.0 mg, 2.0 equiv) was added to the reaction mixture and stirred for another 6 h; isolated yields; ees were determined by HPLC; drs were determined by ^1^H NMR analysis; the absolute configurations of **5** are assigned to (*R,S*)‐**5a** and (*S*,*S*)‐**5a** which were determined by X‐ray analysis.

Next, the substrate scope of **4** was investigated using the above optimal reaction conditions (**Scheme** **6**). When substrates **4a‐h** bearing both electron‐donating and electron‐withdrawing groups on the phenyl ring were examined, the respective target products **5a‐h** could be obtained quantitatively with excellent enantioselectivities (up to >99% ee) and diastereoselectivities (up to >20:1 dr). Substrates **4i** and **4j** bearing benzoheterocyclic substituents were also suitable for the reaction; the desired products were obtained in high yields and excellent enantioselectivities, albeit with somewhat low diastereoselectivities. To our delight, sequential hydrogenations of substrates bearing a 1‐ or 2‐naphthyl group proceeded smoothly, providing **5k** and **5l** with excellent catalytic results. A ferrocene substituted substrate **4m** and aliphatic substrate **4n** were also used in the sequential hydrogenations, providing the corresponding *δ*‐lactone products **5m** and **5n** in high yields and excellent enantioselectivities but with somewhat low diastereoselectivities.

**Scheme 7:**
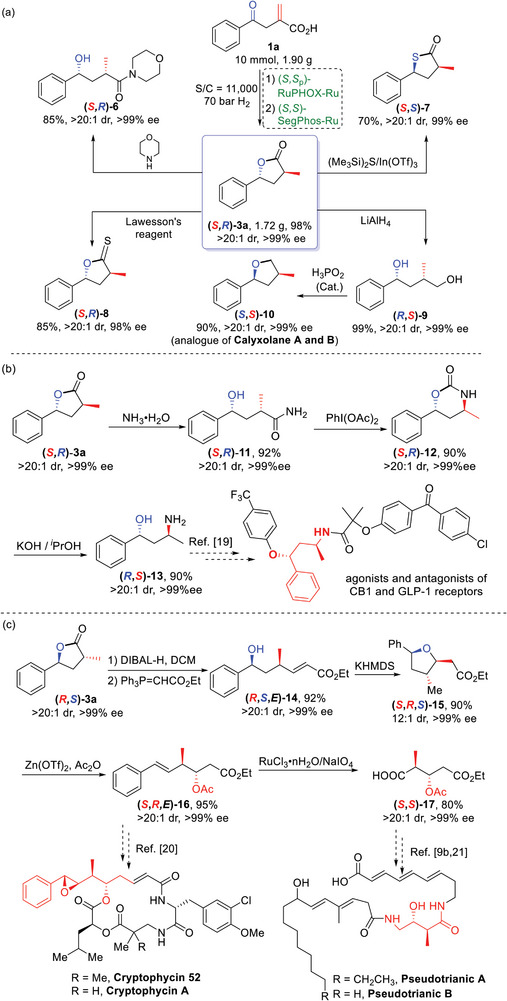
Gram‐scale synthesis and transformations and applications of **3a**.

To further demonstrate the practicality of this sequential hydrogenations, a gram‐scale synthesis and further transformations were performed (**Scheme** **7**). Thus, the hydrogenation of **1a** (1.90 g, 10 mmol) was carried out under the optimal reaction conditions with a lower catalyst loading (11000 S/C) and 70 bar hydrogen pressure. The desired chiral *γ*‐lactone (*S*,*R*)‐**3a** was obtained in 98% yield with >99% ee and >20:1 dr, which could be further converted into several important optically active derivatives (Scheme 7a). First, **3a** could be reacted with morpholine to provide the corresponding amidation product (*S*,*R*)‐**6** (85%, >20:1 dr, >99% ee). By utilizing (Me_3_Si)_2_S, the thiolactone (*S*,*S*)‐**7** could be obtained in 70% yield with excellent enantio‐ and diastereoselectivities. The chiral *γ*‐thionolactone (*S*,*R*)‐**8** could also be synthesized by treating (*S*,*R*)‐**3a** with Lawesson's reagent. The ester group of (*S*,*R*)‐**3a** could be reduced with LiAlH_4_ to afford 1,4‐diol (*R*,*S*)‐**9** without any loss in stereoselectivity; The chiral 2,4‐disubstituted tetrahydrofuran derivative (*S*,*S*)‐**10**, an analogue of the marine natural products calyxolane A and B, was then successfully prepared in 90% yield with >99% ee and >20:1 dr via a H_3_PO_2_‐catalyzed intramolecular stereospecific substitution.^[^ ^17^ ^]^

Compound (*S*,*R*)‐**3a** could also be transformed to the amidation product (*S*,*R*)‐**11** via an ammonolysis with NH_3_·H_2_O, which could then be converted to cyclic carbamate (*S*,*R*)‐**12** via a Hofmann rearrangement. After heating at reflux in KOH/*i*‐PrOH, aminoalcohol (*R*,*S*)‐**13**, a key intermediate for agonists and antagonists of the CB1 and GLP‐1 receptors,^[^ ^18^ ^]^ was obtained in high yield and with excellent stereoselectivity (Scheme 7b).

The acyclic product **14** (92%, >99% ee, and >20:1 dr) was also synthesized from optically active *γ*‐lactone (*R*,*S*)‐**3a** by DIBAL‐H reduction and subsequent Wittig olefination. The product was then converted to the corresponding tetrahydrofuran derivative **15** via an *oxa*‐Michael reaction in 90% yield with >99% ee and 12:1 dr. Upon treatment with Zn(OTf)_2_ catalyst and acetic anhydride, **15** could be converted to **16** (95%, >99% ee, and >20:1 dr) via a ring opening reaction. Compound **16** is a key intermediate for the synthesis of the potent antimitotic antitumor agent (+)‐cryptophycin 52 and A.^[^ ^19^ ^]^ Oxidative cleavage of the C═C double bond of **16** using a catalytic amount of RuCl_3_ and NaIO_4_ as a stoichiometric oxidant could provide carboxylic acid **17** (80%, >99% ee, and >20:1), an important intermediate for the two antimicrobial agents pseudotrienic acid A and B (Scheme 7c).^[^ ^9^, ^20^ ^]^

**Scheme 8:**
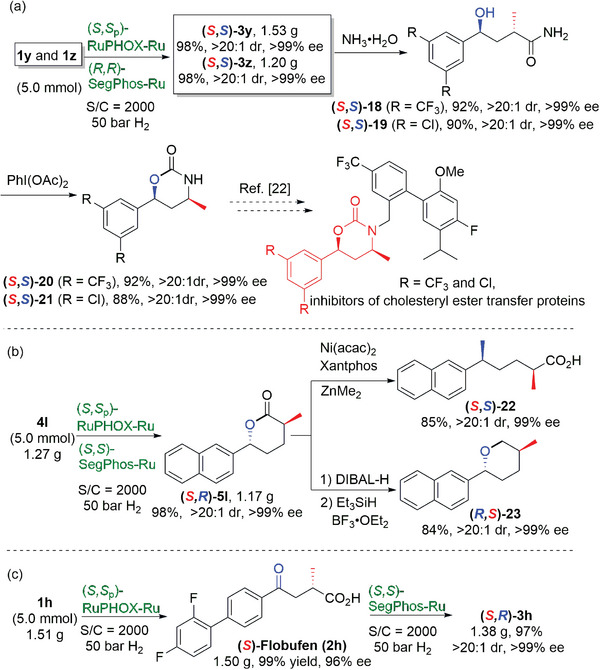
Gram‐scale synthesis and transformations and application of other lactones.

Other applications based on the sequential hydrogenations were also investigated (**Scheme** **8**). Similarly, gram‐scale syntheses of (*S*,*S*)‐**3y** and (*S*,*S*)‐**3z** were realized efficiently via the sequential hydrogenations of **1y** and **1z** (5.0 mmol) with a low catalyst loading (S/C = 2000) under the optimal reactions and 50 bar hydrogen pressure. These could subsequently be converted to the amidation compounds **18** and **19** via an ammonolysis process without any loss in seteroselectivity. The subsequent cyclic carbamates (*S*,*S*)‐**20** and (*S*,*S*)‐**21** were obtained via a Hofmann rearrangement; these compounds are key intermediates for the synthesis of the inhibitors of cholesteryl ester transfer proteins (Scheme 8a).^[^ ^21^ ^]^ The gram‐scale hydrogenation of *α*‐methylene *δ*‐keto carboxylic acid **4l** (1.27 g, 5.0 mmol) was also carried out (Scheme 8b). Under the optimal reaction conditions with 0.05 mol% catalyst loading and 50 bar hydrogen pressure, chiral *δ*‐lactone (*S*,*R*)‐**5l** could be obtained in high yield and with excellent stereoselectivity. This intermediate could be converted to carboxylic acid (*S*,*S*)‐**22** in 85% yield with 99% ee and >20:1 dr via a Ni(acac)_2_/Xantphos catalyzed Negishi‐type cross coupling; the product contains a distal benzylic stereocenter. Alternatively, the ester group of (*S*,*R*)‐**5l** could be reduced with DIBAL‐H and Et_3_SiH/BF_3_·Et_2_O sequentially to afford the chiral 2,5‐disubstituted tetrahydropyran (*R*,*S*)‐**23** (84%, 99% ee and >20:1 dr). In addition, the (*S*,*S*_p_)‐RuPHOX‐Ru‐catalyzed selective hydrogenation of **1** **h** (1.51 g, 5.0 mmol) was carried out on a gram‐scale with low catalyst loading to afford (*S*)‐flobufen (**2h**) in high yield and with excellent enantioselectivity. This compound is used for the treatment of rheumatoid arthritis.^[^ ^22^ ^]^ The final chiral lactone (*S*,*R*)‐**3h** could also be obtained via a (*S*,*S*)‐SegPhos‐Ru catalyzed asymmetric hydrogenation of (*S*)‐flobufen with a low catalyst loading (2000 S/C) in 97% yield with >99% ee and >20:1 dr (Scheme 8c).

## 3 Conclusion 

In conclusion, we have developed an efficient bimetallic Ru/Ru‐catalyzed asymmetric sequential hydrogenations of *γ*‐ and *δ*‐ketoacids for the stereodivergent synthesis of all possible stereoisomers of chiral *γ*‐ and *δ*‐lactones with two non‐vicinal carbon stereocenters. The catalyst system exhibits a broad substrate scope with the desired products being obtained in high yields and excellent stereoselectivities. Investigations into the compatibility of the two chiral Ru catalyst systems revealed that bases with different strengths play a crucial role in the asymmetric sequential hydrogenations. The protocol can be carried out on a gram‐scale with excellent catalytic efficiency (up to S/C = 11000), and the hydrogenated products enable a range of significant transformations, particularly in the synthesis of key intermediates for chiral drugs and natural products. The current strategy provides an efficient and straightforward pathway for the synthesis of all possible stereoisomers of chiral *γ*‐ and *δ*‐lactones bearing *α*‐methyl groups and their derivatives.

## 4 Experimental Methods

### General Procedure for the Chiral Bimetallic Ru/Ru‐Catalyzed Asymmetric Sequential Hydrogenations of *α*‐Methylene‐*γ*‐ and *δ*‐Ketoacids

*α*‐Methylene *γ*‐ or *δ*‐keto carboxylic acids **1** or **4** (0.2 mmol), NaHCO_3_ (8.4 mg, 0.5 equiv), PPh_3_ (26.3 mg, 0.5 equiv) and (*S*,*S*_p_)‐RuPHOX‐Ru (3.4 mg, 1 mol%) were added into a 10 mL vial, followed by the addition of EtOH (1 mL) by a syringe in a nitrogen‐filled glovebox. The vial was subsequently placed in an autoclave which was replaced with hydrogen for three times and charged with hydrogen to 20 bar. The reaction mixture was stirred at room temperature for 6 h. Hydrogen gas was released slowly. A mixture of NaOH (16.0 mg, 2.0 equiv) in EtOH (0.5 mL) was added into the vial in a nitrogen‐filled glovebox, followed by the addition of a solution of (*S*,*S*)‐DM‐Segphos‐Ru (2.4 mg, 1 mol%) in EtOH (0.5 mL). The autoclave was again replaced with hydrogen for three times and charged with hydrogen to 20 bar. The reaction mixture was stirred at room temperature for another 6 h. Hydrogen gas was released slowly and the reaction solvent was removed under reduced pressure. The residue was dissolved in DCM (2 mL) and acidified with HCl (3 m) to pH = 1. The DCM was separated and the aqueous phase was extracted with DCM (2 mL × 3). The combined organic layer was dried over anhydrous Na_2_SO_4_ and evaporated under reduced pressure to afford the crude products. The drs of the products were determined by ^1^H NMR analysis of the crude products which were purified by column chromatography (petroleum ether/EtOAc = 10/1) to afford the pure products **3** or **5**.

CCDC 2302115, CCDC 2302116, and CCDC 2302118 contains the supplementary crystallographic data for this paper. These data can be obtained free of charge from The Cambridge Crystallographic Data Centre via www.ccdc.cam.ac.uk/data_request/cif.

## Conflict of Interest

The authors declare no conflict of interest.

## Author Contributions

J.H. conducted all the synthetic experiments. Z.L. and R.L. conducted part of the melting points and optical rotations test. All authors wrote the manuscript. D.L. and W.Z. directed the project.

## Supporting information

Supporting Information

## Data Availability

The data that support the findings of this study are available in the supplementary material of this article.
