# Supplementary material for: Bimetallic Ru/Ru‐Catalyzed Asymmetric One‐Pot Sequential Hydrogenations for the Stereodivergent Synthesis of Chiral Lactones
Source: Adv Sci (Weinh). 2024 Mar 21;11(23):2400621. doi: 10.1002/advs.202400621 (PMC11187880; doi:10.1002/advs.202400621)
Supplement: Supplementary file 1 — Supporting Information [file ADVS-11-2400621-s001.pdf]

ADVANCED SCIENCE
Open Access

Supporting Information

for *Adv. Sci.*, DOI 10.1002/advs.202400621

Bimetallic Ru/Ru-Catalyzed Asymmetric One-Pot Sequential Hydrogenations for the Stereodivergent Synthesis of Chiral Lactones

Jingli He, Zhaodi Li, Ruhui Li, Xuezhen Kou, Delong Liu and Wanbin Zhang**

Supporting Information

Bimetallic Ru/Ru-Catalyzed Asymmetric One-Pot Sequential Hydrogenations for the Stereodivergent Synthesis of Chiral Lactones

Jingli He,[a] Zhaodi Li,[a] Ruhui Li,[a] Xuezhen Kou,[b] Delong Liu,[a] and Wanbin Zhang[a,b]**

[a] J. He, Z. Li, R. Li, D. Liu and W. Zhang

Shanghai Key Laboratory for Molecular Engineering of Chiral Drugs, School of Pharmacy, Shanghai Jiao Tong University, 800 Dongchuan Road, Shanghai 200240, China

[b] X. Kou and W. Zhang

Frontiers Science Center for Transformative Molecules, School of Chemistry and Chemical Engineering, Shanghai Jiao Tong University, 800 Dongchuan Road, Shanghai 200240, China

Email: dlliu@sjtu.edu.cn; wanbin@sjtu.edu.cn

Contents

1. General Details....................S2
2. Preparation of Substrates....................S2
3. Asymmetric Sequential Hydrogenations of *γ*- and *δ*-Ketoacids....................S22
4. Scale-up Reaction, Transformations and Applications....................S58
5. X-Ray Analysis....................S75
6. Reference....................S78
7. NMR Spectra....................S80
8. HPLC Data....................S250

1. General Details

Unless otherwise specified, the chemicals were obtained commercially and used without further purification. All air- and moisture-sensitive reactions were performed in dried glassware under an atmosphere of nitrogen. All asymmetric reactions were performed in autoclave. Solvents used in the glove box were dried and degassed by standard procedures. ^{1}H, ^{13}C and ^{19}F NMR spectra were obtained using Bruker Avance III HD 400 MHz NMR or Agilent 400 MHz NMR Spectrometer. Melting points were measured with SGW X-4 micro melting point apparatus. Enantioselectivities were determined by high performance liquid chromatography (HPLC) using Daicel Chiralcel OD-H, OJ-H and OC-H columns or Daicel Chiralpak AD-H, AS-H, IC-H and IE-H columns with *n*-hexane/*i*-PrOH as eluent on Shimadzu LC-2010AHT and 2010CHT. Optical rotations were measured on a Rudolph Research Analytical Autopol VI automatic polarimeter using a 50 mm path-length cell at 589 nm. High Resolution Mass Spectrometry (HRMS) analysis was carried out using an electrospray spectrometer Waters Micromass Q-TOF Premier Mass Spectrometer. X-ray single crystal diffraction data were collected on a Bruker D8 VENTURE CMOS photon 100 diffractometer with helios mx multilayer monochrmator Cu Kα radiation (λ = 1.54178 Å) at the Instrumental Analysis Center of Shanghai Jiao Tong University. The optical resolution of **3af**, **5i**, **5j** and **5n** were obtained using chiral preparative HPLC.

2. Preparation of Substrates

The substrates **1a-1k**, **1aa**, **1ab** and **1ad** were synthesized under modified reaction conditions according to the reported procedure.[1a-1b]

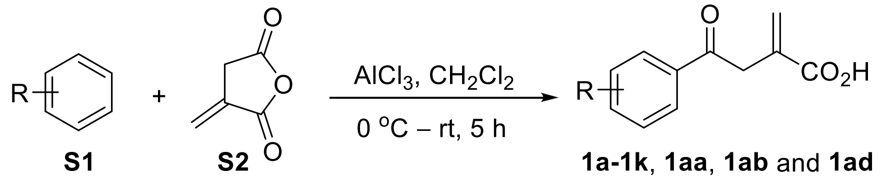

1a-1k, **1aa**, **1ab** and **1ad**

To an oven dried 50 mL flask was added anhydrous DCM (25 mL) followed by the addition of $AlCl_3$ (20 mmol, 2.67 g) slowly under a N_2 atmosphere. Then, the corresponding arene **S1** (15 mmol) was added dropwise before 3-methylenedihydrofuran-2,5-dione **S2** (10 mmol, 1.12 g) was added. The resulting suspension was stirred at room temperature for 5 h. After the reaction was finished, the suspension was poured into ice water (20 mL), aqueous HCl solution (36 wt%, 5 mL) was added dropwise, and the mixture was stirred for 0.5 h. The mixture was extracted

with DCM (50 mL × 5). The combined organic phase was dried over anhydrous Na_2SO_4 and concentrated under reduced pressure to give the crude compound, which was further recrystallized with PE/EtOAc as the solvent to give the pure products.

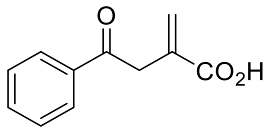

2-Methylene-4-oxo-4-phenylbutanoic acid (1a)[1a-1b]

White solid (1.63 g, 86%). ^{1}H NMR (400 MHz, $CDCl_3$) δ 7.98 (d, J = 7.2 Hz, 2H), 7.58 (t, J = 7.4 Hz, 1H), 7.47 (t, J = 7.6 Hz, 2H), 6.53 (s, 1H), 5.81 (s, 1H), 4.00 (s, 2H); ^{13}C NMR (101 MHz, $CDCl_3$) δ 196.6, 171.3, 136.4, 134.0, 133.4, 131.0, 128.7, 128.3, 41.3.

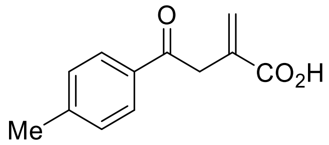

2-Methylene-4-oxo-4-(*p*-tolyl)butanoic acid (1b)[1a-1b]

White solid (1.83 g, 90%). ^{1}H NMR (400 MHz, $CDCl_3$) δ 7.89–7.87 (m, 2H), 7.28–7.26 (m, 2H), 6.53 (d, J = 1.1 Hz, 1H), 5.80 (d, J = 1.1 Hz, 1H), 3.98 (s, 2H), 2.42 (s, 3H); ^{13}C NMR (101 MHz, $CDCl_3$) δ 196.3, 171.5, 144.2, 134.1, 133.9, 130.8, 129.4, 128.4, 41.2, 21.7.

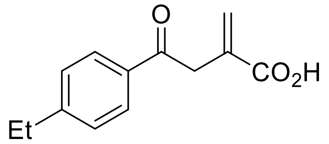

4-(4-Ethylphenyl)-2-methylene-4-oxobutanoic acid (1c)

White solid (2.01 g, 92%). Mp: 148–150 °C; ^{1}H NMR (400 MHz, $CDCl_3$) δ 7.90 (d, J = 7.9 Hz, 2H), 7.29 (d, J = 7.8 Hz, 2H), 6.52 (s, 1H), 5.79 (s, 1H), 3.97 (s, 2H), 2.71 (q, J = 7.6 Hz, 2H), 1.26 (t, J = 7.6 Hz, 3H); ^{13}C NMR (101 MHz, $CDCl_3$) δ 196.2, 171.6, 150.3, 134.1, 130.8, 128.5, 128.1, 41.1, 28.9, 15.1; HRMS (ESI) m/z calcd. for $C_{13}H_{13}O_3$ $[M-H]^-$: 217.0870, found 217.0873.

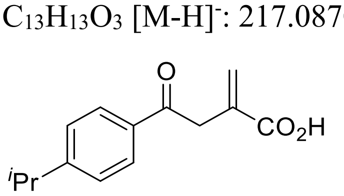

4-(4-Isopropylphenyl)-2-methylene-4-oxobutanoic acid (1d)[1a-1b]

White solid (2.06 g, 89%). ^{1}H NMR (400 MHz, $CDCl_3$) δ 7.91 (d, J = 8.2 Hz, 2H), 7.32 (d, J = 8.2 Hz, 2H), 6.52 (s, 1H), 5.79 (s, 1H), 3.97 (s, 2H), 3.0–2.93 (m, 1H), 1.26 (d, J = 7.0 Hz, 6H); ^{13}C NMR (101 MHz, $CDCl_3$) δ 196.2, 171.7, 154.9, 134.2, 134.1, 130.8, 128.5, 126.7, 41.1, 34.2, 23.6.

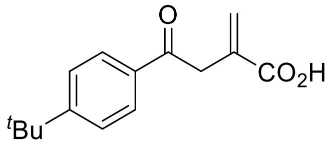

4-(4-(*tert*-Butyl)phenyl)-2-methylene-4-oxobutanoic acid (1e)[1a-1b]

White solid (2.24 g, 91%). ^{1}H NMR (400 MHz, CDCl$_3$) δ 7.92 (d, J = 8.3 Hz, 2H), 7.48 (d, J = 8.0 Hz, 2H), 6.52 (s, 1H), 5.79 (s, 1H), 3.98 (s, 2H), 1.34 (s, 9H); ^{13}C NMR (101 MHz, CDCl$_3$) δ 196.2, 171.8, 157.1, 134.1, 133.8, 130.8, 128.2, 125.6, 41.1, 35.1, 31.0.

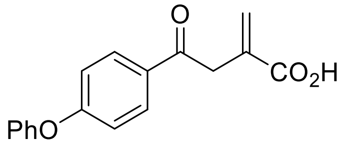

2-Methylene-4-oxo-4-(4-phenoxyphenyl)butanoic acid (1f)[1b]

White solid (2.27 g, 80%). ^{1}H NMR (400 MHz, CDCl$_3$) δ 7.96 (d, J = 9.1 Hz, 2H), 7.40 (t, J = 7.6 Hz, 2H), 7.22–7.18 (m, 1H), 7.09–7.06 (m, 2H), 7.02–6.98 (m, 2H), 6.52 (s, 1H), 5.80 (s, 1H), 3.95 (s, 2H); ^{13}C NMR (101 MHz, CDCl$_3$) δ 195.2, 171.4, 162.3, 155.4, 134.0, 131.0, 130.9, 130.6, 130.1, 124.7, 120.3, 117.4, 41.1.

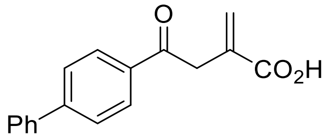

4-([1,1'-Biphenyl]-4-yl)-2-methylene-4-oxobutanoic acid (1g)

White solid (2.07 g, 78%). Mp: 189–191 °C; ^{1}H NMR (400 MHz, DMSO-d_6) δ 12.54 (s, 1H), 8.08 (d, J = 8.4 Hz, 2H), 7.84 (d, J = 8.4 Hz, 2H), 7.76 (d, J = 7.5 Hz, 2H), 7.53-7.49 (m, 2H), 7.46–7.42 (m, 1H), 6.24 (d, J = 1.7 Hz, 1H), 5.76 (d, J = 1.7 Hz, 1H), 4.08 (s, 2H); ^{13}C NMR (101 MHz, DMSO-d_6) δ 197.1, 168.0, 145.1, 139.3, 136.5, 135.6, 129.6, 129.2, 128.9, 128.4, 127.5, 127.4, 42.1; HRMS (ESI) m/z calcd. for $C_{17}H_{13}O_3$ [M-H]$^-$: 265.0870, found 265.0863.

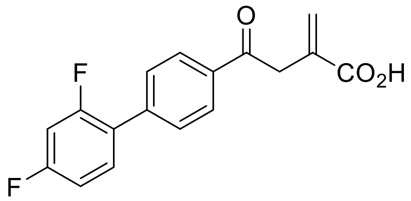

4-(2',4'-Difluoro-[1,1'-biphenyl]-4-yl)-2-methylene-4-oxobutanoic acid (1h)

White solid (1.57 g, 52%). Mp: 169–170 °C; ^{1}H NMR (400 MHz, DMSO-d_6) δ 12.56 (s, 1H), 8.09 (d, J = 8.4 Hz, 2H), 7.71–7.64 (m, 3H), 7.46–7.40 (m, 1H), 7.27–7.22 (m, 1H), 6.24 (s, 1H), 5.77 (s, 1H), 4.09 (s, 2H); ^{13}C NMR (101 MHz, DMSO-d_6) δ 197.2 (d, J = 2.5 Hz), 168.0, 162.4 (dd, J = 303.1, 12.3 Hz), 159.9 (dd, J = 304.6, 12.4 Hz), 139.4, 136.4, 136.0, 132.6 (dd, J = 9.8, 4.5 Hz), 129.6 (d, J = 3.1 Hz), 128.9, 128.5,

124.3 (q, J = 12.9 Hz), 112.8 (dd, J = 21.4, 4.0 Hz), 105.2 (t, J = 26.9 Hz), 42.1; ^{19}F NMR (377 MHz, DMSO-d_6) δ –109.8, –113.3; HRMS (ESI) m/z calcd. for $C_{17}H_{11}F_2O_3$ [M-H]$^-$: 301.0682, found 301.0681.

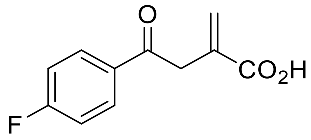

4-(4-Fluorophenyl)-2-methylene-4-oxobutanoic acid (1i)[1a-1b]

White solid (0.94 g, 45%). ^{1}H NMR (400 MHz, $CDCl_3$) δ 8.02–7.98(m, 2H), 7.16–7.11 (m, 2H), 6.54 (s, 1H), 5.81 (s, 1H), 3.96 (s, 2H); ^{13}C NMR (101 MHz, $CDCl_3$) δ 195.0, 171.5, 165.9 (d, J = 256.2 Hz), 133.8, 132.8 (d, J = 3.0 Hz), 131.2, 131.0 (d, J = 9.4 Hz), 115.8 (d, J = 22.0 Hz), 41.2; ^{19}F NMR (376 MHz, $CDCl_3$) δ –104.8.

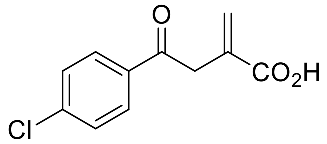

4-(4-Chlorophenyl)-2-methylene-4-oxobutanoic acid (1j)[1a-1b]

White solid (1.12 g, 50%). ^{1}H NMR (400 MHz, $CDCl_3$) δ 7.91 (dd, J = 8.6, 1.4 Hz, 2H), 7.44 (dd, J = 8.6, 1.4 Hz, 2H), 6.54 (s, 1H), 5.81 (s, 1H), 3.96 (s, 2H); ^{13}C NMR (101 MHz, $CDCl_3$) δ 195.4, 171.1, 139.9, 134.7, 133.6, 131.2, 129.7, 129.0, 41.2.

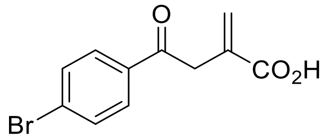

4-(4-Bromophenyl)-2-methylene-4-oxobutanoic acid (1k)[1a]

White solid (1.55 g, 58%). ^{1}H NMR (400 MHz, $CDCl_3$) δ 7.84 (d, J = 8.6 Hz, 2H), 7.62 (d, J = 8.6 Hz, 2H), 6.54 (s, 1H), 5.82 (s, 1H), 3.95 (s, 2H); ^{13}C NMR (101 MHz, $CDCl_3$) δ 195.6, 171.0, 135.1, 133.6, 132.0, 131.2, 129.8, 128.6, 41.2.

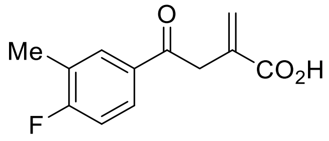

4-(4-Fluoro-3-methylphenyl)-2-methylene-4-oxobutanoic acid (1aa)[1b]

White solid (1.60 g, 72%). ^{1}H NMR (400 MHz, $CDCl_3$) δ 7.86–7.78 (m, 2H), 7.07 (t, J = 8.8 Hz, 1H), 6.53 (s, 1H), 5.80 (s, 1H), 3.95 (s, 2H), 2.32 (s, 3H); ^{13}C NMR (101 MHz, $CDCl_3$) δ 195.3, 171.7, 164.6 (d, J = 254.9 Hz), 133.9, 132.5 (d, J = 3.4 Hz), 132.1 (d, J = 6.7 Hz), 131.1, 128.3 (d, J = 9.5 Hz), 125.5 (d, J = 18.0 Hz), 115.3 (d, J = 23.2 Hz), 41.2, 14.6 (d, J = 3.6 Hz); ^{19}F NMR (376 MHz, $CDCl_3$) δ –108.9.

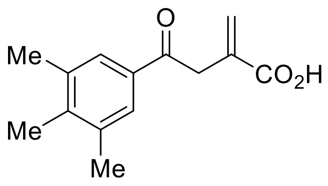

2-Methylene-4-oxo-4-(3,4,5-trimethylphenyl)butanoic acid (1ab)[1b]

White solid (1.35 g, 58%). ^{1}H NMR (400 MHz, $CDCl_3$) δ 7.61 (s, 2H), 6.51 (s, 1H), 5.78 (s, 1H), 3.96 (s, 2H), 2.33 (s, 6H), 2.22 (s, 3H); ^{13}C NMR (101 MHz, $CDCl_3$) δ 196.7, 171.6, 141.6, 136.9, 134.3, 133.5, 130.7, 127.5, 41.1, 20.7, 15.9.

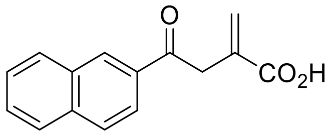

2-Methylene-4-(naphthalen-2-yl)-4-oxobutanoic acid (1ad)[1a-1b]

White solid (1.08 g, 45%). ^{1}H NMR (400 MHz, $CDCl_3$) δ 8.50 (s, 1H), 8.03 (d, J = 8.6 Hz, 1H), 7.96 (d, J = 8.1 Hz, 1H), 7.88 (t, J = 8.8 Hz, 2H), 7.62–7.53 (m, 2H), 6.56 (s, 1H), 5.85 (s, 1H), 4.14 (s, 2H); ^{13}C NMR (101 MHz, $CDCl_3$) δ 196.5, 171.2, 135.7, 134.0, 133.7, 132.5, 131.0, 130.1, 129.6, 128.6, 128.5, 127.8, 126.8, 123.9, 41.4.

The substrates **1l-1z**, **1ac**, **1ae** and **1af** were synthesized under modified reaction conditions according to the reported procedure.[1a-1b]

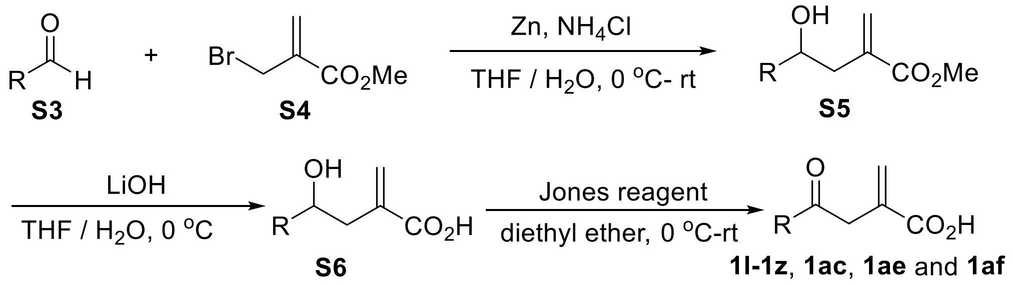

Synthesis of S5: To an oven dried 100 mL flask was added aldehyde **S3** (10 mmol), methyl 2-(bromomethyl)acrylate **S4** (12 mmol, 2.15 g) and THF (40 mL) under N_2 atmosphere. Then, the zinc dust (12 mmol, 0.79 g) and saturated aqueous NH_4Cl solution (5 mL) were added to the suspension solution at 0 °C. The resulting suspension was allowed to warm to room temperature and stirred at this temperature for 4 h. After reaction was completed, water (40 mL) was added. The organic layer was separated and the aqueous phase was extracted with ethyl acetate (50 mL × 3). The combined organic phase was dried over anhydrous Na_2SO_4 and concentrated under reduced pressure to give the crude compound **S5**, which was directly used in the next step without further purification.

Synthesis of S6: The above **S5** and THF (50 mL) were added into a 200 mL round bottom flask, followed by the addition of aqueous LiOH solution (1M, 50 mL) dropwise at 0 °C. The reaction mixture was stirred at 0 °C until the disappearance of the starting material. The reaction mixture was acidified with 1M HCl to pH = 1 and the water phase was extracted with ethyl acetate (50 mL × 3). The combined organic layer was washed with brine (60 mL), dried over anhydrous Na_2SO_4 and concentrated under reduced pressure to give the crude compound **S6**, which was directly used in the next step without further purification.

Synthesis of 1: The above **S6** and diethyl ether (20 mL) were added into 50 mL round bottom flask. Jones reagent (1 M, 20 mmol) was added dropwise under the ice bath. The resulting mixture was allowed to warm to room temperature and stir for 4 h. After reaction was completed, ethyl acetate (30 mL) and H_2O (30 mL) were added into the reaction mixture. After separating the organic layer, the aqueous layer was extracted with ethyl acetate (30 mL × 3). The combined organic layer was washed with brine (60 mL), dried over anhydrous Na_2SO_4 and concentrated under reduced pressure. The residue was purified by column chromatography (PE/EtOAc = 2/1) to give the target products **1**.

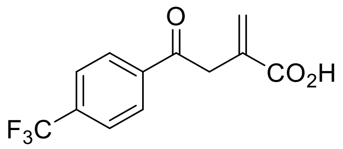

2-Methylene-4-oxo-4-(4-(trifluoromethyl)phenyl)butanoic acid (1l)[1b]

White solid (1.16 g, 45%). ^{1}H NMR (400 MHz, DMSO-*d*$_6$) δ 12.60 (s, 1H), 8.18 (d, J = 8.0 Hz, 2H), 7.92–7.89 (m, 2H), 6.25 (s, 1H), 5.78 (s, 1H), 4.11 (s, 2H); ^{13}C NMR (101 MHz, DMSO-*d*$_6$) δ 197.1, 167.9, 140.0, 136.1, 133.1 (q, J = 32.2 Hz), 129.3, 128.8, 126.2 (d, J = 4.1 Hz), 124.2 (q, J = 273.8 Hz), 42.4; ^{19}F NMR (376 MHz, DMSO-*d*$_6$) δ –61.7.

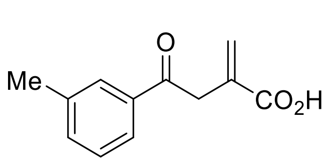

2-Methylene-4-oxo-4-(*m*-tolyl)butanoic acid (1m)[1b]

White solid (1.22 g, 60%). ^{1}H NMR (400 MHz, DMSO-*d*$_6$) δ 12.52 (s, 1H), 7.79 (d, J = 9.2 Hz, 2H), 7.47–7.40 (m, 2H), 6.22 (s, 1H), 5.73 (s, 1H), 4.03 (s, 2H), 2.38 (s, 3H); ^{13}C NMR (101 MHz, DMSO-*d*$_6$) δ 197.6, 168.0, 138.6, 136.9, 136.5, 134.3, 129.1, 128.9, 128.3, 125.7, 42.1, 21.3.

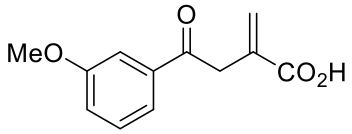

4-(3-Methoxyphenyl)-2-methylene-4-oxobutanoic acid (1n)[1b]

White solid (1.61 g, 73%). ^{1}H NMR (400 MHz, DMSO-d_6) δ 12.53 (s, 1H), 7.60–7.58 (m, 1H), 7.47–7.43 (m, 2H), 7.23–7.20 (m, 1H), 6.22 (d, J = 1.7 Hz, 1H), 5.74 (d, J = 1.7 Hz, 1H), 4.04 (s, 2H), 3.82 (s, 3H); ^{13}C NMR (101 MHz, DMSO-d_6) δ 197.3, 168.0, 159.9, 138.2, 136.5, 130.4, 128.4, 121.0, 119.8, 113.0, 55.8, 42.2.

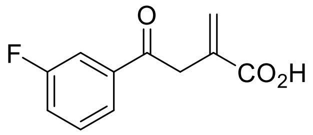

4-(3-Fluorophenyl)-2-methylene-4-oxobutanoic acid (1o)[1b]

White solid (1.27 g, 61%). ^{1}H NMR (400 MHz, $CDCl_3$) δ 7.77–7.74 (m, 1H), 7.67–7.63 (m, 1H), 7.48–7.43 (m, 1H), 7.30–7.27 (m, 1H), 6.55 (s, 1H), 5.82 (s, 1H), 3.97 (s, 2H); ^{13}C NMR (101 MHz, $CDCl_3$) δ 195.4 (d, J = 2.2 Hz), 171.6, 162.9 (d, J = 249.0 Hz), 138.4 (d, J = 6.2 Hz), 133.6, 131.3, 130.4 (d, J = 7.7 Hz), 124.0 (d, J = 3.0 Hz), 120.4 (d, J = 21.5 Hz), 115.0 (d, J = 22.4 Hz), 41.4; ^{19}F NMR (377 MHz, $CDCl_3$) δ –111.7.

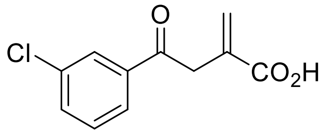

4-(3-Chlorophenyl)-2-methylene-4-oxobutanoic acid (1p)[1b]

White solid (1.43 g, 64%). ^{1}H NMR (400 MHz, $CDCl_3$) δ 7.94 (t, J = 1.9 Hz, 1H), 7.86–7.83 (m, 1H), 7.56–7.53 (m, 1H), 7.41 (t, J = 7.8 Hz, 1H), 6.55 (s, 1H), 5.82 (s, 1H), 3.96 (s, 2H); ^{13}C NMR (101 MHz, $CDCl_3$) δ 195.3, 171.7, 137.9, 135.0, 133.6, 133.3, 131.4, 130.0, 128.4, 126.4, 41.4.

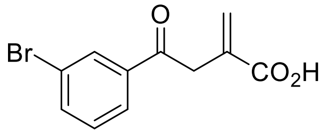

4-(3-Bromophenyl)-2-methylene-4-oxobutanoic acid (1q)

White solid (1.77 g, 66%). Mp: 149–150 °C; ^{1}H NMR (400 MHz, $CDCl_3$) δ 8.10 (t, J = 1.9 Hz, 1H), 7.90–7.88 (m, 1H), 7.71–7.68 (m, 1H), 7.35 (t, J = 7.8 Hz, 1H), 6.55 (d, J = 0.9 Hz, 1H), 5.82 (d, J = 1.1 Hz, 1H), 3.96 (s, 2H); ^{13}C NMR (101 MHz, $CDCl_3$) δ 195.2, 171.6, 138.1, 136.2, 133.6, 131.4, 131.3, 130.3, 126.8, 123.0, 41.4; HRMS (ESI) m/z calcd. for $C_{11}H_8BrO_3$ $[M-H]^-$: 266.9662, found 266.9662.

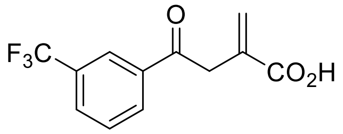

2-Methylene-4-oxo-4-(3-(trifluoromethyl)phenyl)butanoic acid (1r)

White solid (1.21 g, 47%). Mp: 89–91 °C; ^{1}H NMR (400 MHz, $CDCl_3$) δ 8.23–8.15 (m, 2H), 7.83 (d, J = 7.8 Hz, 1H), 7.62 (t, J = 7.8 Hz, 1H), 6.57 (s, 1H), 5.85 (s, 1H), 4.02 (s, 2H); ^{13}C NMR (101 MHz, $CDCl_3$) δ 195.2, 171.7, 136.8, 133.4, 131.6, 131.4, 131.36 (d, J = 32.9), 129.8 (q, J = 3.8 Hz), 129.4, 125.1 (q, J = 4.0 Hz), 123.6 (q, J = 273.4 Hz), 41.4; ^{19}F NMR (376 MHz, $CDCl_3$) δ –62.6; HRMS (ESI) m/z calcd. for $C_{12}H_8F_3O_3$ [M-H]$^-$: 257.0431, found 257.0431.

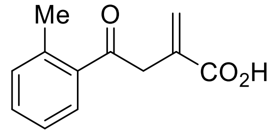

2-Methylene-4-oxo-4-(*o*-tolyl)butanoic acid (1s)

White solid (0.84 g, 41%). Mp: 95–96 °C; ^{1}H NMR (400 MHz, $CDCl_3$) δ 7.71 (dd, J = 7.6, 1.4 Hz, 1H), 7.40–7.36 (m, 1H), 7.29–7.24 (m, 2H), 6.54 (d, J = 1.0 Hz, 1H), 5.81 (d, J = 1.2 Hz, 1H), 3.92 (s, 2H), 2.49 (s, 3H); ^{13}C NMR (101 MHz, $CDCl_3$) δ 200.2, 172.0, 138.6, 137.2, 134.3, 132.0, 131.6, 131.1, 128.7, 125.7, 44.2, 21.3; HRMS (ESI) m/z calcd. for $C_{12}H_{11}O_3$ [M-H]$^-$: 203.0714, found 203.0713.

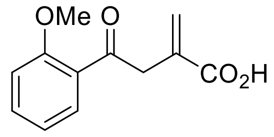

4-(2-Methoxyphenyl)-2-methylene-4-oxobutanoic acid (1t)

White solid (0.92 g, 42%). Mp: 122–124 °C; ^{1}H NMR (400 MHz, $CDCl_3$) δ 7.77 (dd, J = 7.8, 1.9 Hz, 1H), 7.49–7.45 (m, 1H), 7.02–6.95 (m, 2H), 6.45 (d, J = 1.2 Hz, 1H), 5.73 (d, J = 1.3 Hz, 1H), 4.0 (s, 2H), 3.90 (s, 3H); ^{13}C NMR (101 MHz, $CDCl_3$) δ 198.3, 172.2, 158.8, 134.9, 134.0, 130.8, 130.1, 127.2, 120.7, 111.5, 55.5, 46.6; HRMS (ESI) m/z calcd. for $C_{12}H_{11}O_4$ [M-H]$^-$: 219.0663, found 219.0663.

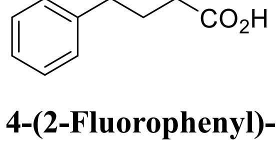

4-(2-Fluorophenyl)- 2-methylene-4-oxobutanoic acid (1u)

White solid (0.79 g, 38%). Mp: 125–127 °C; ^{1}H NMR (400 MHz, $CDCl_3$) δ 7.91–7.87 (m, 1H), 7.56–7.50 (m, 1H), 7.25–7.11 (m, 2H), 6.51 (s, 1H), 5.80 (s, 1H), 3.99 (d, J = 3.0 Hz, 2H); ^{13}C NMR (101 MHz, $CDCl_3$) δ 194.8 (d, J = 4.4 Hz), 171.7, 162.1 (d, J =

255.5 Hz), 134.9 (d, J = 9.1 Hz), 134.0 (d, J = 2.3 Hz), 130.89, 130.86 (d, J = 1.8 Hz), 125.0 (d, J = 13.0 Hz), 124.6 (d, J = 3.3 Hz), 116.7 (d, J = 23.9 Hz), 46.3 (d, J = 8.8 Hz); ^{19}F NMR (376 MHz, $CDCl_3$) δ –109.0; HRMS (ESI) m/z calcd. for $C_{11}H_8FO_3$ $[M-H]^-$: 207.0463, found 207.0465.

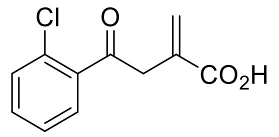

4-(2-Chlorophenyl)-2-methylene-4-oxobutanoic acid (1v)

White solid (0.92 g, 41%). Mp: 101–102 °C; ^{1}H NMR (400 MHz, $CDCl_3$) δ 7.56–7.54 (m, 1H), 7.43–7.31 (m, 3H), 6.54 (s, 1H), 5.89 (s, 1H), 3.96 (s, 2H); ^{13}C NMR (101 MHz, $CDCl_3$) δ 199.4, 171.8, 138.7, 133.6, 132.0, 131.7, 131.0, 130.5, 129.5, 127.0, 45.6; HRMS (ESI) m/z calcd. for $C_{11}H_8ClO_3$ $[M-H]^-$: 223.0167, found 223.0171.

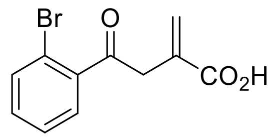

4-(2-Bromophenyl)-2-methylene-4-oxobutanoic acid (1w)

White solid (1.15 g, 43%). Mp: 110–112 °C; ^{1}H NMR (400 MHz, $CDCl_3$) δ 7.61 (dd, J = 8.0, 1.2 Hz, 1H), 7.49 (dd, J = 7.6, 1.8 Hz, 1H), 7.40–7.36 (m, 1H), 7.32–7.28 (m, 1H), 6.55 (d, J = 0.9 Hz, 1H), 5.91 (d, J = 1.1 Hz, 1H), 3.94 (s, 2H); ^{13}C NMR (101 MHz, $CDCl_3$) δ 200.2, 171.6, 141.0, 133.7, 133.4, 131.8, 131.7, 129.0, 127.5, 118.7, 45.2; HRMS (ESI) m/z calcd. for $C_{11}H_8BrO_3$ $[M-H]^-$: 266.9662, found 266.9670.

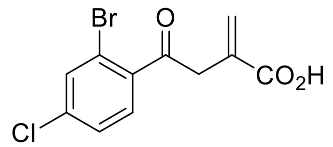

4-(2-Bromo-4-chlorophenyl)-2-methylene-4-oxobutanoic acid (1x)

White solid (1.08 g, 36%). Mp: 99–101 °C; ^{1}H NMR (400 MHz, $CDCl_3$) δ 7.64 (d, J = 1.9 Hz, 1H), 7.47 (d, J = 8.3 Hz, 1H), 7.38–7.36 (m, 1H), 6.55 (d, J = 0.9 Hz, 1H), 5.91 (d, J = 1.0 Hz, 1H), 3.91 (s, 2H); ^{13}C NMR (101 MHz, $CDCl_3$) δ 198.9, 171.5, 139.1, 137.4, 133.5, 133.2, 132.0, 130.2, 127.9, 119.6, 45.1; HRMS (ESI) m/z calcd. for $C_{11}H_7BrClO_3$ $[M-H]^-$: 300.9273, found 300.9273.

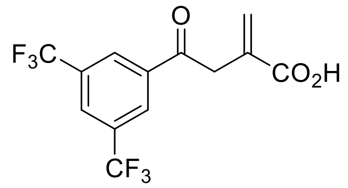

4-(3,5-Bis(trifluoromethyl)phenyl)-2-methylene-4-oxobutanoic acid (1y)

White solid (1.24 g, 38%). Mp: 138–140 °C; ^{1}H NMR (400 MHz, $CDCl_3$) δ 8.41 (d, J = 1.6 Hz, 2H), 8.08 (s, 1H), 6.60 (s, 1H), 5.90 (s, 1H), 4.04 (s, 2H); ^{13}C NMR (101 MHz, $CDCl_3$) δ 193.8, 171.2, 137.8, 132.9, 132.5 (q, J = 34.2 Hz), 132.0, 128.3 (q, J = 3.8 Hz), 126.7–126.3 (m), 122.8 (q, J = 273.8 Hz), 41.5. ^{19}F NMR (377 MHz, $CDCl_3$) δ –62.8; HRMS (ESI) m/z calcd. for $C_{13}H_7F_6O_3$ [M-H]$^-$: 325.0305, found 325.0311.

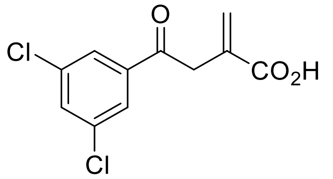

4-(3,5-Dichlorophenyl)-2-methylene-4-oxobutanoic acid (1z)

White solid (1.10 g, 43%). Mp: 177–178 °C; ^{1}H NMR (400 MHz, DMSO-d_6) δ 12.58 (s, 1H), 7.97–7.90 (m, 3H), 6.23 (s, 1H), 5.76 (s, 1H), 4.08 (s, 2H); ^{13}C NMR (101 MHz, DMSO-d_6) δ 195.7, 167.8, 139.7, 136.0, 135.3, 132.9, 128.8, 127.2, 42.3; HRMS (ESI) m/z calcd. for $C_{11}H_7Cl_2O_3$ [M-H]$^-$: 256.9778, found 256.9784.

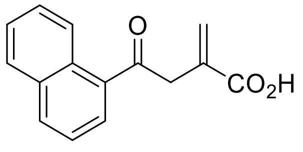

2-Methylene-4-(naphthalen-1-yl)-4-oxobutanoic acid (1ac)[1b]

White solid (0.98 g, 41%). ^{1}H NMR (400 MHz, $CDCl_3$) δ 8.61 (d, J = 8.5 Hz, 1H), 8.01–7.94 (m, 2H), 7.87 (d, J = 8.0 Hz, 1H), 7.59–7.48 (m, 3H), 6.58 (s, 1H), 5.88 (s, 1H), 4.08 (s, 2H); ^{13}C NMR (101 MHz, $CDCl_3$) δ 200.3, 171.7, 135.2, 134.2, 133.9, 132.9, 131.2, 130.2, 128.3, 128.0, 127.8, 126.5, 125.8, 124.3, 44.7.

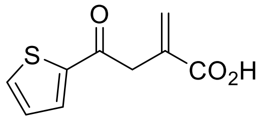

2-Methylene-4-oxo-4-(thiophen-2-yl)butanoic acid (1ae)

White solid (0.61 g, 31%). Mp: 139–141 °C; ^{1}H NMR (400 MHz, $CDCl_3$) δ 7.77 (d, J = 3.8 Hz, 1H), 7.65 (d, J = 4.9 Hz, 1H), 7.14 (t, J = 4.4 Hz, 1H), 6.54 (s, 1H), 5.86 (s, 1H), 3.93 (s, 2H); ^{13}C NMR (101 MHz, $CDCl_3$) δ 189.3, 171.5, 143.4, 134.1, 133.4, 132.5, 131.4, 128.2, 41.7; HRMS (ESI) m/z calcd. for $C_9H_7O_3S$ [M-H]$^-$: 195.0121, found 195.0127.

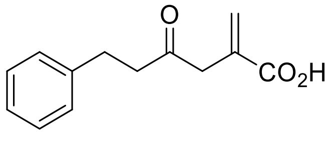

2-Methylene-4-oxo-6-phenylhexanoic acid (1af)[1b]

White solid (1.16 g, 53%). ^1H NMR (400 MHz, $CDCl_3$) δ 7.30–7.17 (m, 5H), 6.47 (s, 1H), 5.73 (s, 1H), 3.38 (s, 2H), 2.94–2.80 (m, 4H); ^{13}C NMR (101 MHz, $CDCl_3$) δ 206.2, 171.6, 140.9, 133.5, 131.3, 128.5, 128.4, 126.2, 45.5, 44.1, 29.7.

The following substrates **1ag-1aj** were synthesized under modified reaction conditions according to the reported procedure.[1c]

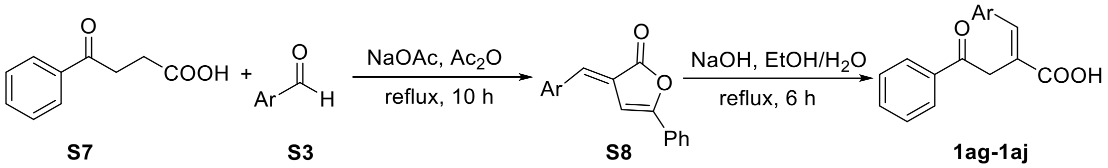

Synthesis of S8: To an oven dried 100 mL flask was added 3-benzoylpropionic acid **S7** (20 mmol, 3.56 g), aldehyde **S3** (20 mmol), sodium acetate (20 mmol, 1.64 g) and acetic anhydride (30 mL). The reaction mixture was stirred with refluxing for 10 hours until the complete consumption of starting material was confirmed by TLC analysis. After cooling to room temperature, water (30 mL) was added with stirring and the solid product was filtered, washed with water and petroleum ether to give the crude compound **S8**, which were directly used in the next step without further purification.

Synthesis of 1ag-1aj: To an oven dried 100 mL flask was added **S8** (8.4 mmol), sodium hydroxide (8.4 mmol, 0.34 g) and ethanol/water (2:1, *v*/*v*, 60 mL). The reaction mixture was stirred with refluxing for 6 hours until the complete consumption of starting material was confirmed by TLC analysis. After cooling to room temperature, ethanol was removed under reduced pressure. Then the reaction mixture was acidified with 1M HCl to pH = 1 and the aqueous layer was extracted with ethyl acetate (30 mL × 3). The combined organic layer was washed with brine (40 mL), dried over anhydrous Na_2SO_4 and concentrated under reduced pressure. The residue was purified by column chromatography (PE/EtOAc = 2/1) to give the desired products **1ag-1aj**.

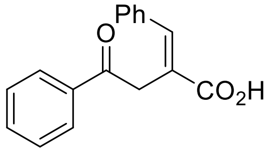

(*E*)-2-Benzylidene-4-oxo-4-phenylbutanoic acid (1ag)[1c]

Light yellow solid (1.89 g, 85%). ^1H NMR (400 MHz, $CDCl_3$) δ 8.14 (s, 1H), 8.01 (d, J = 7.4 Hz, 2H), 7.59 (t, J = 7.4 Hz, 1H), 7.48 (t, J = 7.6 Hz, 2H), 7.37–7.29 (m, 5H), 4.21 (s, 2H); ^{13}C NMR (101 MHz, $CDCl_3$) δ 197.1, 173.1, 144.5, 136.5, 135.0, 133.4, 129.1, 128.9, 128.7, 128.3, 126.3, 37.9; HRMS (ESI) m/z calcd. for $C_{17}H_{14}NaO_3$ $[M+Na]^+$: 289.0835, found 289.0832.

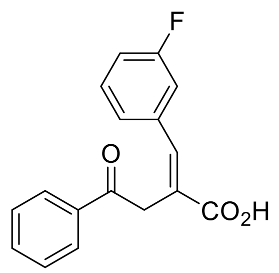

(*E*)-2-(3-Fluorobenzylidene)-4-oxo-4-phenylbutanoic acid (1ah)

Light yellow solid (2.20 g, 92%). Mp: 146–147 °C; ^{1}H NMR (400 MHz, $CDCl_3$) δ 8.07 (s, 1H), 8.00 (d, J = 7.7 Hz, 2H), 7.59 (t, J = 7.5 Hz, 1H), 7.48 (t, J = 7.6 Hz, 2H), 7.34–7.29 (m, 1H), 7.09–7.01 (m, 3H), 4.17 (s, 2H); ^{13}C NMR (101 MHz, $CDCl_3$) δ 196.9, 172.6, 162.7 (d, J = 248.0 Hz), 143.1 (d, J = 2.3 Hz), 137.0 (d, J = 7.8 Hz), 136.4, 133.5, 130.3 (d, J = 8.4 Hz), 128.7, 128.3, 127.5, 124.6 (d, J = 2.9 Hz), 116.1 (d, J = 21.2 Hz), 115.7 (d, J = 22.2 Hz), 37.7; ^{19}F NMR (377 MHz, $CDCl_3$) δ –112.1; HRMS (ESI) m/z calcd. for $C_{17}H_{13}FNaO_3$ $[M+Na]^+$: 307.0741, found 307.0738.

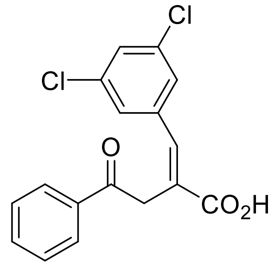

(*E*)-2-(3,5-Dichlorobenzylidene)-4-oxo-4-phenylbutanoic acid (1ai)

White solid (2.53 g, 90%). Mp: 178–182 °C; ^{1}H NMR (400 MHz, DMSO-d_6) δ 8.03 (d, J = 7.3 Hz, 2H), 7.81 (s, 1H), 7.68 (t, J = 7.3 Hz, 1H), 7.61 (t, J = 1.9 Hz, 1H), 7.56 (t, J = 7.6 Hz, 2H), 7.40 (d, J = 1.9 Hz, 2H), 4.19 (s, 2H); ^{13}C NMR (101 MHz, DMSO-d_6) δ 198.0, 168.4, 139.1, 138.3, 136.6, 134.8, 134.1, 131.2, 129.3, 128.7, 128.6, 127.6, 38.2; HRMS (ESI) m/z calcd. for $C_{17}H_{12}Cl_2NaO_3$ $[M+Na]^+$: 357.0056, found 357.0060.

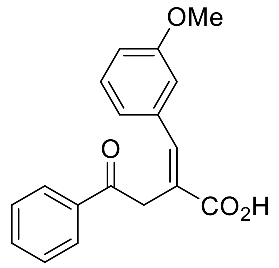

(*E*)-2-(3-Methoxybenzylidene)-4-oxo-4-phenylbutanoic acid (1aj)

Light yellow solid (1.99 g, 80%). Mp: 154–156 °C; ^{1}H NMR (400 MHz, $CDCl_3$) δ 8.13 (s, 1H), 8.03 (d, J = 7.8 Hz, 2H), 7.61 (t, J = 7.2 Hz, 1H), 7.50 (t, J = 7.5 Hz, 2H), 7.30–7.28 (m, 1H), 6.92–6.86 (m, 3H), 4.24 (s, 2H), 3.72 (s, 3H); ^{13}C NMR (101 MHz, $CDCl_3$) δ 197.1, 172.7, 159.6, 144.4, 136.5, 136.3, 133.4, 129.7, 128.7, 128.3, 126.5, 121.2,

115.2, 113.8, 55.2, 38.0; HRMS (ESI) m/z calcd. for $C_{18}H_{16}NaO_4$ [M+Na]$^+$: 319.0941, found 319.0942.

The following substrates **4a-4n** were synthesized according to the reported procedure.[1b]

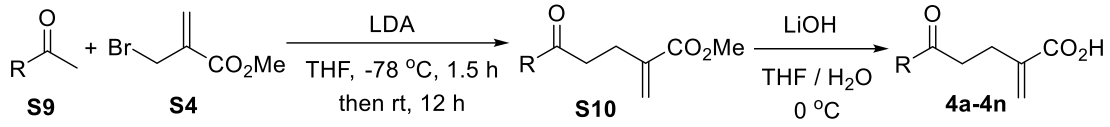

Synthesis of S10: To an oven dried 50 mL double mouth round bottom flask was added **S9** (10 mmol) and dry THF (20 mL) under N_2 atomsphere. Then LDA (5 mL, 2M in THF/hexane) was added dropwise at –78 °C and the resulting reaction was stirred for 0.5 h. The solution of methyl 2-(bromomethyl)acrylate **S4** (12 mmol, 2.15 g) in dry THF (10 mL) was added dropwise. The reaction mixture was stirred for additional 1 h, allowed to warm to room temperature and stirred for 12 h. After the reaction was completed, saturated aqueous NH_4Cl solution (20 mL) was added. The separated aqueous layer was extracted with ethyl acetate (30 mL × 3). The combined organic layer was washed with brine (60 mL), dried over anhydrous Na_2SO_4 and concentrated under reduced pressure. The residue was purified by silicon gel column chromatography (PE/EtOAc = 15/1) to give the desired methyl ester product **S10**.

Synthesis of 4: The methyl ester **S10** (6 mmol), THF (30 mL) and a stir bar were added into a 100 mL round bottom flask and aqueous LiOH solution (1M, 30 mL) was added dropwise at 0 °C. The reaction mixture was stirred at the same temperature and analyzed by TLC. After the reaction was completed, the reaction mixture was acidified with 1M HCl to pH = 1 and the water phase was extracted with ethyl acetate (30 mL × 3). The combined organic layer was washed with brine (40 mL), dried over anhydrous Na_2SO_4 and concentrated under reduced pressure. The residue was purified by column chromatography (PE/EtOAc = 2/1) to give the desired product **4**.

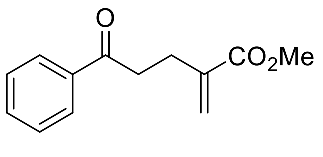

Methyl 2-methylene-5-oxo-5-phenylpentanoate (S10-1)

White solid (1.44 g, 66%). Mp: 46–47 °C; ^{1}H NMR (400 MHz, $CDCl_3$) δ 7.97 (d, J = 7.6 Hz, 2H), 7.56 (t, J = 7.8 Hz, 1H), 7.46 (t, J = 7.6 Hz, 2H), 6.21 (s, 1H), 5.67 (s, 1H), 3.77 (s, 3H), 3.19 (t, J = 7.4 Hz, 2H), 2.76 (t, J = 7.5 Hz, 2H); ^{13}C NMR (101 MHz, $CDCl_3$) δ 199.1, 167.4, 139.3, 136.7, 133.1, 128.6, 128.1, 126.3, 52.0, 37.6, 26.9; HRMS (ESI) m/z calcd. for $C_{13}H_{14}NaO_3$ [M+Na]$^+$: 241.0836, found 241.0838.

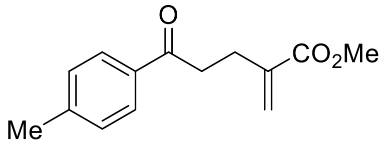

Methyl 2-methylene-5-oxo-5-(*p*-tolyl)pentanoate (S10-2)[1b]

White solid (1.48 g, 64%). ^{1}H NMR (400 MHz, $CDCl_3$) δ 7.87 (d, J = 8.3 Hz, 2H), 7.25 (d, J = 7.9 Hz, 2H), 6.20 (d, J = 1.3 Hz, 1H), 5.65 (d, J = 1.4 Hz, 1H), 3.77 (s, 3H), 3.15 (t, J = 7.5 Hz, 2H), 2.75 (t, J = 7.5 Hz, 2H), 2.40 (s, 3H); ^{13}C NMR (101 MHz, $CDCl_3$) δ 198.7, 167.4, 143.9, 139.4, 134.3, 129.3, 128.2, 126.1, 51.9, 37.5, 27.0, 21.6.

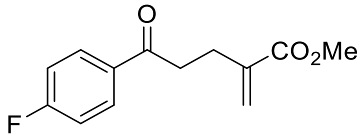

Methyl 5-(4-fluorophenyl)-2-methylene-5-oxopentanoate (S10-3)

White solid (1.65 g, 70%). Mp: 54–56 °C; ^{1}H NMR (400 MHz, $CDCl_3$) δ 8.02–7.97 (m, 2H), 7.14–7.09 (m, 2H), 6.20 (s, 1H), 5.66 (s, 1H), 3.77 (s, 3H), 3.15 (t, J = 7.5 Hz, 2H), 2.74 (t, J = 7.5 Hz, 2H); ^{13}C NMR (101 MHz, $CDCl_3$) δ 197.4, 167.3, 165.8 (d, J = 255.5 Hz), 139.2, 133.2 (d, J = 3.1 Hz), 130.7 (d, J = 9.3 Hz), 126.3, 115.7 (d, J = 22.0 Hz), 51.9, 37.6, 27.0; ^{19}F NMR (377 MHz, $CDCl_3$) δ –105.2; HRMS (ESI) m/z calcd. for $C_{13}H_{13}FNaO_3$ $[M+Na]^+$: 259.0741, found 259.0737.

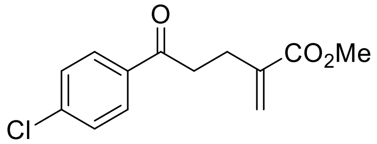

Methyl 5-(4-chlorophenyl)-2-methylene-5-oxopentanoate (S10-4)

White solid (1.71 g, 68%). Mp: 62–64 °C; ^{1}H NMR (400 MHz, $CDCl_3$) δ 7.91 (d, J = 8.8 Hz, 2H), 7.43 (d, J = 7.9 Hz, 2H), 6.20 (s, 1H), 5.66 (s, 1H), 3.77 (s, 3H), 3.15 (t, J = 7.5 Hz, 2H), 2.74 (t, J = 7.5 Hz, 2H); ^{13}C NMR (101 MHz, $CDCl_3$) δ 197.8, 167.3, 139.6, 139.1, 135.1, 129.5, 129.0, 126.4, 51.9, 37.7, 27.0; HRMS (ESI) m/z calcd. for $C_{13}H_{13}ClNaO_3$ $[M+Na]^+$: 275.0445, found 275.0446.

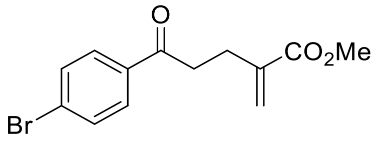

Methyl 5-(4-bromophenyl)-2-methylene-5-oxopentanoate (S10-5)

White solid (1.98 g, 67%). Mp: 98–100 °C; ^{1}H NMR (400 MHz, $CDCl_3$) δ 7.83 (d, J = 8.6 Hz, 2H), 7.60 (d, J = 8.6 Hz, 2H), 6.20 (d, J = 1.2 Hz, 1H), 5.66 (d, J = 1.3 Hz, 1H), 3.77 (s, 3H), 3.15 (t, J =7.5 Hz, 2H), 2.74 (t, J =7.5 Hz, 2H); ^{13}C NMR (101 MHz, $CDCl_3$) δ 198.0, 167.3, 139.1, 135.5, 131.9, 129.6, 128.3, 126.4, 51.9, 37.6, 27.0;

HRMS (ESI) m/z calcd. for $C_{13}H_{13}BrNaO_3$ [M+Na]$^+$: 318.9940, found 318.9940.

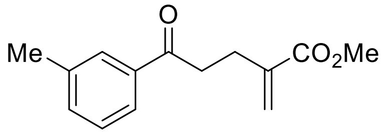

Methyl 2-methylene-5-oxo-5-(*m*-tolyl)pentanoate (S10-6)

White solid (1.42 g, 61%). Mp: 50–51 °C; ^{1}H NMR (400 MHz, $CDCl_3$) δ 7.77–7.74 (m, 2H), 7.38–7.31 (m, 2H), 6.20 (d, J = 1.4 Hz, 1H), 5.65 (d, J = 1.4 Hz, 1H), 3.76 (s, 3H), 3.17 (t, J = 7.5 Hz, 2H), 2.75 (t, J = 7.5 Hz, 2H), 2.40 (s, 3H); ^{13}C NMR (101 MHz, $CDCl_3$) δ 199.2, 167.4, 139.4, 138.4, 136.8, 133.8, 128.6, 128.5, 126.1, 125.3, 51.9, 37.6, 26.9, 21.4; HRMS (ESI) m/z calcd. for $C_{14}H_{16}NaO_3$ [M+Na]$^+$: 255.0992, found 255.0991.

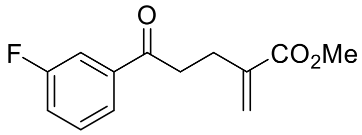

Methyl 5-(3-fluorophenyl)-2-methylene-5-oxopentanoate (S10-7)

White solid (1.58 g, 67%). Mp: 82–84 °C; ^{1}H NMR (400 MHz, $CDCl_3$) δ 7.75 (d, J = 7.7 Hz, 1H), 7.64 (d, J = 9.5 Hz, 1H), 7.46–7.61 (m, 1H), 7.28–7.23 (m, 1H), 6.21 (s, 1H), 5.66 (s, 1H), 3.77 (s, 3H), 3.16 (t, J = 7.5 Hz, 2H), 2.75 (t, J = 7.5 Hz, 2H); ^{13}C NMR (101 MHz, $CDCl_3$) δ 197.7 (d, J = 2.1 Hz), 167.3, 162.9 (d, J = 248.7 Hz), 139.1, 138.9 (d, J = 6.1 Hz), 130.3 (d, J = 7.7 Hz), 126.4, 123.8 (d, J = 2.9 Hz), 120.1 (d, J = 21.6 Hz), 114.8 (d, J = 22.4 Hz), 51.9, 37.8, 26.9; ^{19}F NMR (377 MHz, $CDCl_3$) δ –111.6; HRMS (ESI) m/z calcd. for $C_{13}H_{13}FNaO_3$ [M+Na]$^+$: 259.0741, found 259.0741.

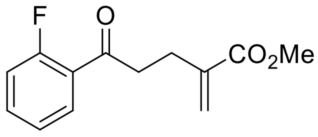

Methyl 5-(2-fluorophenyl)-2-methylene-5-oxopentanoate (S10-8)

White solid (1.60 g, 68%). Mp: 53–54 °C; ^{1}H NMR (400 MHz, $CDCl_3$) δ 7.87–7.82 (m, 1H), 7.53–7.47 (m, 1H), 7.24–7.19 (m, 1H), 7.14–7.09 (m, 1H), 6.19 (d, J = 1.3 Hz, 1H), 5.62 (d, J = 1.3 Hz, 1H), 3.75 (s, 3H), 3.18 (t, J = 7.4 Hz, 2H), 2.74 (t, J = 7.4 Hz, 2H); ^{13}C NMR (101 MHz, $CDCl_3$) δ 197.3 (d, J = 4.1 Hz), 167.3, 161.9 (d, J = 255.3 Hz), 139.3, 134.5 (d, J = 9.1 Hz), 130.6 (d, J = 2.7 Hz), 125.9, 125.6 (d, J = 13.1 Hz), 124.5 (d, J = 3.3 Hz), 116.7 (d, J = 24.0 Hz), 51.9, 42.3 (d, J = 7.5 Hz), 26.4 (d, J = 2.2 Hz); ^{19}F NMR (377 MHz, $CDCl_3$) δ –109.1; HRMS (ESI) m/z calcd. for $C_{13}H_{13}FNaO_3$ [M+Na]$^+$: 259.0741, found 259.0741.

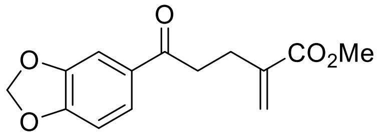

Methyl 5-(benzo[*d*][1,3]dioxol-5-yl)-2-methylene-5-oxopentanoate (S10-9)

White solid (1.89 g, 72%). Mp: 101–103 °C; ^{1}H NMR (400 MHz, $CDCl_3$) δ 7.58–7.55 (m, 1H), 7.44–7.42 (m, 1H), 6.85–6.82 (m, 1H), 6.18 (s, 1H), 6.02 (s, 2H), 5.63 (s, 1H), 3.76 (s, 3H), 3.09 (t, J = 7.5 Hz, 2H), 2.72 (t, J = 7.5 Hz, 2H); ^{13}C NMR (101 MHz, $CDCl_3$) δ 197.1, 167.4, 151.7, 148.2, 139.4, 131.7, 126.1(3), 126.1(1), 124.3, 107.9, 101.8, 51.9, 37.4, 27.2; HRMS (ESI) m/z calcd. for $C_{14}H_{14}NaO_5$ [M+Na]$^+$: 285.0733, found 285.0733.

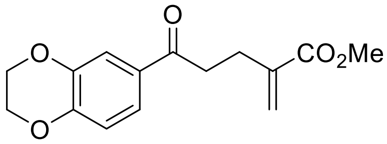

Methyl 5-(2,3-dihydrobenzo[*b*][1,4]dioxin-6-yl)-2-methylene-5-oxopentanoate (S10-10)

White solid (1.88 g, 68%). Mp: 68–70 °C; ^{1}H NMR (400 MHz, $CDCl_3$) δ 7.51–7.49 (m, 2H), 6.89 (d, J = 8.5 Hz, 1H), 6.19 (s, 1H), 5.63 (s, 1H), 4.32–4.26 (m, 4H), 3.76 (s, 3H), 3.09 (t, J = 7.5 Hz, 2H), 2.72 (t, J = 7.5 Hz, 2H); ^{13}C NMR (101 MHz, $CDCl_3$) δ 197.5, 167.4, 148.0, 143.3, 139.4, 130.7, 126.1, 122.2, 117.6, 117.2, 64.7, 64.1, 51.9, 37.3, 27.1; HRMS (ESI) m/z calcd. for $C_{15}H_{16}NaO_5$ [M+Na]$^+$: 299.0890, found 299.0890.

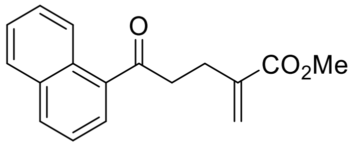

Methyl 2-methylene-5-(naphthalen-1-yl)-5-oxopentanoate (S10-11)

Colourless oil (1.90 g, 71%). ^{1}H NMR (400 MHz, $CDCl_3$) δ 8.61 (d, J = 8.6 Hz, 1H), 7.96 (d, J = 8.2 Hz, 1H), 7.87 (t, J = 7.3 Hz, 2H), 7.60–7.45 (m, 3H), 6.21 (d, J = 1.3 Hz, 1H), 5.67 (d, J = 1.3 Hz, 1H), 3.76 (s, 3H), 3.27 (t, J = 7.5 Hz, 2H), 2.83 (t, J = 7.5 Hz, 2H); ^{13}C NMR (101 MHz, $CDCl_3$) δ 203.2, 167.4, 139.3 135.7, 134.0, 132.7, 130.2, 128.4, 127.9, 127.6, 126.5, 126.2, 125.8, 124.4, 51.9, 40.8, 27.3; HRMS (ESI) m/z calcd. for $C_{17}H_{16}NaO_3$ [M+Na]$^+$: 291.0992, found 291.0992.

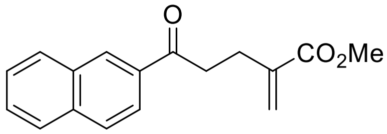

Methyl 2-methylene-5-(naphthalen-2-yl)-5-oxopentanoate (S10-12)

White solid (1.93 g, 72%). Mp: 88–89 °C; ^{1}H NMR (400 MHz, $CDCl_3$) δ 8.49 (s, 1H), 8.03 (dd, J = 8.6, 1.8 Hz, 1H), 7.96 (d, J = 8.1 Hz, 1H), 7.88 (t, J = 8.0 Hz, 2H), 7.61–7.52 (m, 2H), 6.23 (d, J = 1.3 Hz, 1H), 5.70 (d, J = 1.3 Hz, 1H), 3.79 (s, 3H), 3.32 (t, J = 7.5 Hz, 2H), 2.82 (t, J = 7.5 Hz, 2H); ^{13}C NMR (101 MHz, $CDCl_3$) δ 199.0, 167.4, 139.4, 135.6, 134.1, 132.6, 129.8, 129.6, 128.47, 128.45, 127.8, 126.8, 126.3, 123.9, 51.9, 37.7, 27.1; HRMS (ESI) m/z calcd. for $C_{17}H_{16}NaO_3$ $[M+Na]^+$: 291.0992, found 291.0992.

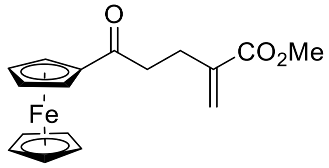

Methyl 2-methylene-5-(ferrocene)-5-oxopentanoate (S10-13)

White solid (1.37 g, 42%). Mp: 83–85 °C; ^{1}H NMR (400 MHz, $CDCl_3$) δ 6.21 (d, J = 1.4 Hz, 1H), 5.70 (d, J = 1.4 Hz, 1H), 4.79 (t, J = 2.0 Hz, 2H), 4.49 (t, J = 1.9 Hz, 2H), 4.19 (s, 5H), 3.78 (s, 3H), 2.92 (t, J = 7.6 Hz, 2H), 2.72 (t, J = 7.5 Hz, 2H); ^{13}C NMR (101 MHz, $CDCl_3$) δ 203.2, 167.4, 139.5, 126.4, 78.8, 72.3, 69.8, 69.3, 51.9, 38.7, 27.2; HRMS (ESI) m/z calcd. for $C_{17}H_{18}FeNaO_3$ $[M+Na]^+$: 349.0498, found 349.0501.

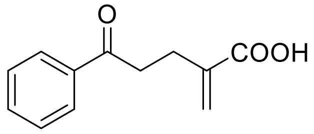

2-Methylene-5-oxo-5-phenylpentanoic acid (4a)

White solid (1.09 g, 89%). Mp: 112–114 °C; ^{1}H NMR (400 MHz, $CDCl_3$) δ 7.97 (d, J = 7.7 Hz, 2H), 7.58–7.44 (m, 3H), 6.37 (s, 1H), 5.80 (s, 1H), 3.23 (t, J = 7.4 Hz, 2H), 2.77 (t, J = 7.4 Hz, 2H); ^{13}C NMR (101 MHz, $CDCl_3$) δ 199.0, 172.5, 138.8, 136.7, 133.2, 128.9, 128.7, 128.1, 37.5, 26.5; HRMS (ESI) m/z calcd. for $C_{12}H_{11}O_3$ $[M-H]^-$: 203.0714, found 203.0715.

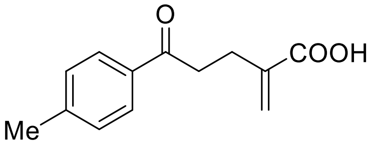

2-Methylene-5-oxo-5-(*p*-tolyl)pentanoic acid (4b)[1b]

White solid (1.12 g, 86%). ^{1}H NMR (400 MHz, $CDCl_3$) δ 7.90–7.88 (m, 2H), 7.28 (d, J = 1.6 Hz, 2H), 6.37 (d, J = 1.2 Hz, 1H), 5.80 (d, J = 1.2 Hz, 1H), 3.21 (t, J = 7.3 Hz, 2H), 2.77 (t, J = 6.9 Hz, 2H), 2.42 (s, 3H); ^{13}C NMR (101 MHz, $CDCl_3$) δ 198.7, 172.3, 144.0, 138.9, 134.3, 129.3, 128.7, 128.2, 37.4, 26.6, 21.7.

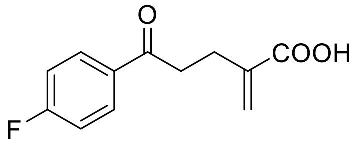

5-(4-Fluorophenyl)-2-methylene-5-oxopentanoic acid (4c)

White solid (1.21 g, 91%). Mp: 135–137 °C; ^{1}H NMR (400 MHz, $CDCl_3$) δ 8.02–7.97 (m, 2H), 7.16–7.05 (m, 2H), 6.37 (d, J = 1.2 Hz, 1H), 5.80 (d, J = 1.2 Hz, 1H), 3.19 (t, J = 7.0 Hz, 2H), 2.76 (t, J = 6.6 Hz, 2H); ^{13}C NMR (101 MHz, $CDCl_3$) δ 197.4, 172.2, 165.8 (d, J = 255.7 Hz), 138.6, 133.2 (d, J = 3.0 Hz), 130.7 (d, J = 9.4 Hz), 128.9, 115.7 (d, J = 21.9 Hz), 37.5, 26.5. ^{19}F NMR (377 MHz, $CDCl_3$) δ –105.1; HRMS (ESI) m/z calcd. for $C_{12}H_{10}FO_3$ $[M-H]^-$: 221.0619, found 221.0622.

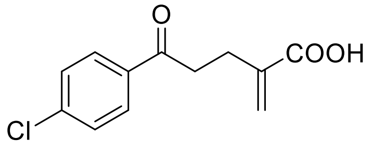

5-(4-Chlorophenyl)-2-methylene-5-oxopentanoic acid (4d)

White solid (1.29 g, 90%). Mp: 146–147 °C; ^{1}H NMR (400 MHz, $CDCl_3$) δ 7.92–7.89 (m, 2H), 7.45–7.42 (m, 2H), 6.37 (d, J = 1.2 Hz, 1H), 5.80 (d, J = 1.2 Hz, 1H), 3.18 (t, J = 7.2 Hz, 2H), 2.76 (t, J = 7.1 Hz, 2H); ^{13}C NMR (101 MHz, $CDCl_3$) δ 197.7, 172.2, 139.6, 138.6, 135.0, 129.5, 129.0, 128.9, 37.5, 26.5; HRMS (ESI) m/z calcd. for $C_{12}H_{10}ClO_3$ $[M-H]^-$: 237.0324, found 237.0328.

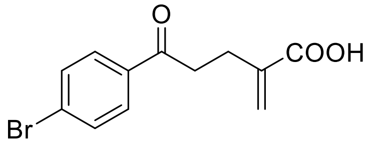

5-(4-Bromophenyl)-2-methylene-5-oxopentanoic acid (4e)

White solid (1.48 g, 88%). Mp: 156–157 °C; ^{1}H NMR (400 MHz, $CDCl_3$) δ 7.85–7.81 (m, 2H), 7.62–7.58 (m, 2H), 6.37 (d, J = 1.2 Hz, 1H), 5.80 (d, J = 1.2 Hz, 1H), 3.18 (t, J = 6.9 Hz, 2H), 2.75 (t, J = 6.9 Hz, 2H); ^{13}C NMR (101 MHz, $CDCl_3$) δ 197.9, 172.1, 138.5, 135.4, 132.0, 129.6, 129.0, 128.4, 37.5, 26.5; HRMS (ESI) m/z calcd. for $C_{12}H_{10}BrO_3$ $[M-H]^-$: 280.9819, found 280.9828.

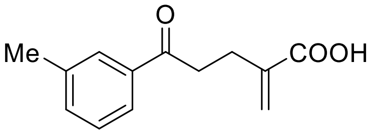

2-Methylene-5-oxo-5-(*m*-tolyl)pentanoic acid (4f)

White solid (1.12 g, 86%). Mp: 102–104 °C; ^{1}H NMR (400 MHz, $CDCl_3$) δ 7.78–7.75 (m, 2H), 7.39–7.32 (m, 2H), 6.36 (d, J = 1.2 Hz, 1H), 5.79 (d, J = 1.2 Hz, 1H), 3.21 (t, J = 7.4 Hz, 2H), 2.76 (t, J = 7.4, 2H), 2.41 (s, 3H); ^{13}C NMR (101 MHz, $CDCl_3$) δ

199.3, 172.4, 138.8, 138.4, 136.8, 133.9, 128.7, 128.6, 128.5, 125.3, 37.6, 26.5, 21.4; HRMS (ESI) m/z calcd. for $C_{13}H_{13}O_3$ [M-H]$^-$: 217.0870, found 217.0874.

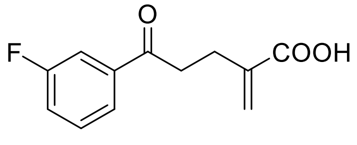

5-(3-Fluorophenyl)-2-methylene-5-oxopentanoic acid (4g)

White solid (1.23 g, 92%). Mp: 119–121 °C; ^{1}H NMR (400 MHz, $CDCl_3$) δ 7.76–7.73 (m, 1H), 7.66–7.63 (m, 1H), 7.47–7.41 (m, 1H), 7.28–7.23 (m, 1H), 6.37 (d, *J* = 1.2 Hz, 1H), 5.80 (d, *J* = 1.2 Hz, 1H), 3.20 (t, *J* = 7.4 Hz, 2H), 2.77 (t, *J* = 7.4 Hz, 2H); ^{13}C NMR (101 MHz, $CDCl_3$) δ 197.7 (d, *J* = 2.2 Hz), 172.3, 162.9 (d, *J* = 248.9 Hz), 138.8 (d, *J* = 6.1 Hz), 138.5, 130.3 (d, *J* = 7.6 Hz), 128.9, 123.8 (d, *J* = 3.1 Hz), 120.2 (d, *J* = 21.5 Hz), 114.8 (d, *J* = 22.3 Hz), 37.7, 26.4; ^{19}F NMR (377 MHz, $CDCl_3$) δ –111.8; HRMS (ESI) m/z calcd. for $C_{12}H_{10}FO_3$ [M-H]$^-$: 221.0619, found 221.0619.

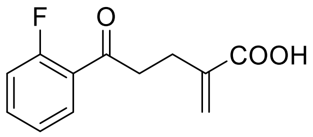

5-(2-Fluorophenyl)-2-methylene-5-oxopentanoic acid (4h)

White solid (1.16 g, 87%). Mp: 66–68 °C; ^{1}H NMR (400 MHz, $CDCl_3$) δ 7.88–7.84 (m, 1H), 7.54–7.49 (m, 1H), 7.25–7.21 (m, 1H), 7.15–7.10 (m, 1H), 6.36 (d, *J* = 1.2 Hz, 1H), 5.77 (d, *J* = 1.2 Hz, 1H), 3.24–3.20 (m, 2H), 2.76 (t, *J* = 7.3 Hz, 2H); ^{13}C NMR (101 MHz, $CDCl_3$) δ 197.3 (d, *J* = 4.1 Hz), 172.1, 162.0 (d, *J* = 255.6 Hz), 138.7, 134.6 (d, *J* = 9.0 Hz), 130.6 (d, *J* = 2.8 Hz), 128.5, 125.5 (d, *J* = 13.0 Hz), 124.5 (d, *J* = 3.4 Hz), 116.7 (d, *J* = 23.9 Hz), 42.3 (d, *J* = 7.6 Hz), 26.0 (d, *J* = 2.2 Hz); ^{19}F NMR (376 MHz, $CDCl_3$) δ –109.2; HRMS (ESI) m/z calcd. for $C_{12}H_{10}FO_3$ [M-H]$^-$: 221.0619, found 221.0621.

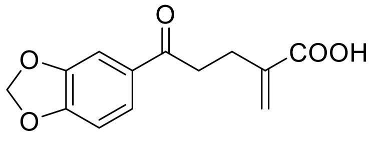

5-(Benzo[*d*][1,3]dioxol-5-yl)-2-methylene-5-oxopentanoic acid (4i)

White solid (1.28 g, 86%). Mp: 174–175 °C; ^{1}H NMR (400 MHz, DMSO-*d*$_6$) δ 12.52 (s, 1H), 7.63 (dd, *J* = 8.2, 1.8 Hz, 1H), 7.46 (d, *J* = 1.7 Hz, 1H), 7.03 (d, *J* = 8.2 Hz, 1H), 6.13 (s, 2H), 6.06 (d, *J* = 1.6 Hz, 1H), 5.65 (d, *J* = 1.6 Hz, 1H), 3.12 (t, *J* = 7.5 Hz, 2H), 2.55–2.51 (t, *J* = 7.5 Hz, 2H); ^{13}C NMR (101 MHz, DMSO-*d*$_6$) δ 197.5, 168.4, 151.9, 148.3, 140.4, 131.7, 125.3, 124.8, 108.5, 107.8, 102.5, 37.0, 26.6; HRMS (ESI) m/z calcd. for $C_{13}H_{11}O_5$ [M-H]$^-$: 247.0612, found 247.0613.

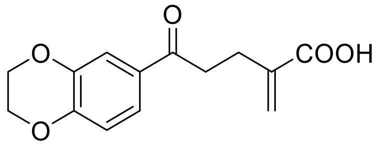

5-(2,3-Dihydrobenzo[*b*][1,4]dioxin-6-yl)-2-methylene-5-oxopentanoic acid (4j)

White solid (1.40 g, 89%). Mp: 123–125 °C; ^{1}H NMR (400 MHz, $CDCl_3$) δ 7.44–7.42 (m, 2H), 6.83–6.87 (m, 1H), 6.27 (d, J = 1.3 Hz, 1H), 5.69 (d, J = 1.3 Hz, 1H), 4.24–4.18 (m, 4H), 3.05 (t, J = 7.5 Hz, 2H), 2.66 (t, J = 7.5 Hz, 1H); ^{13}C NMR (101 MHz, $CDCl_3$) δ 197.5, 172.3, 148.1, 143.4, 138.9, 130.6, 128.5, 122.2, 117.6, 117.2, 64.7, 64.1, 37.2, 26.6; HRMS (ESI) m/z calcd. for $C_{14}H_{13}O_5$ $[M-H]^-$: 261.0768, found 261.0773.

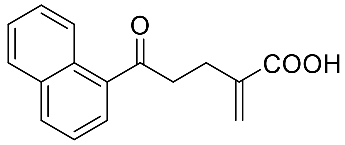

2-Methylene-5-(naphthalen-1-yl)-5-oxopentanoic acid (4k)

White solid (1.28 g, 84%). Mp: 114–116 °C; ^{1}H NMR (400 MHz, $CDCl_3$) δ 8.63 (dd, J = 8.4, 1.2 Hz, 1H), 8.01 (d, J = 8.2 Hz, 1H), 7.92–7.89 (m, 2H), 7.64–7.50 (m, 3H), 6.41 (d, J = 1.2 Hz, 1H), 5.84 (d, J = 1.2 Hz, 1H), 3.33 (t, J =7.4 Hz, 2H), 2.87 (t, J = 7.4 Hz, 1H); ^{13}C NMR (101 MHz, $CDCl_3$) δ 203.2, 172.5, 138.8, 135.7, 134.0, 132.8, 130.1, 128.8, 128.5, 128.0, 127.7, 126.5, 125.8, 124.4, 40.8, 26.9; HRMS (ESI) m/z calcd. for $C_{16}H_{13}O_3$ $[M-H]^-$: 253.0870, found 253.0871.

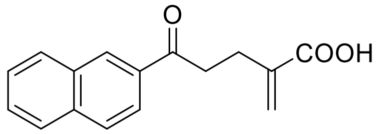

2-Methylene-5-(naphthalen-2-yl)-5-oxopentanoic acid (4l)

White solid (1.33 g, 87%). Mp: 163–164 °C; ^{1}H NMR (400 MHz, $CDCl_3$) δ 8.52 (d, J = 1.7 Hz, 1H), 8.06 (dd, J = 8.6, 1.8 Hz, 1H), 7.98 (dd, J = 8.0, 1.4 Hz, 1H), 7.93–7.88 (m, 2H), 7.64–7.55 (m, 2H), 6.42 (d, J = 1.2 Hz, 1H), 5.86 (s, J = 1.2 Hz, 1H), 3.39 (t, J = 7.7 Hz, 2H), 2.87 (t, J = 7.7 Hz, 2H); ^{13}C NMR (101 MHz, $CDCl_3$) δ 198.9, 172.2, 138.8, 135.6, 134.1, 132.6, 129.8, 129.6, 128.8, 128.51, 128.49, 127.8, 126.8, 123.8, 37.7, 26.7; HRMS (ESI) m/z calcd. for $C_{16}H_{13}O_3$ $[M-H]^-$: 253.0870, found 253.0874.

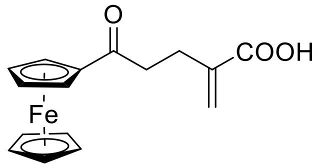

2-Methylene-5-(ferrocene)-5-oxopentanoic acid (4m)

White solid (1.38 g, 74%). Mp: 128–130 °C; ^{1}H NMR (400 MHz, $CDCl_3$) δ 6.37 (s, 1H), 5.83 (s, 1H), 4.80 (s, 2H), 4.50 (s, 2H), 4.20 (s, 5H), 2.95 (t, J = 7.6 Hz, 2H), 2.74 (t, J = 7.6 Hz, 2H); ^{13}C NMR (101 MHz, $CDCl_3$) δ 203.5, 171.9, 139.1, 128.7, 78.7, 72.4, 69.8, 69.4, 38.6, 26.8; HRMS (ESI) m/z calcd. for $C_{16}H_{15}FeO_3$ [M-H]$^-$: 311.0376, found 311.0373.

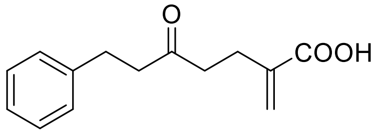

2-Methylene-5-oxo-7-phenylheptanoic acid (4n)

White solid (1.07 g, 77%). Mp: 73–74 °C; ^{1}H NMR (400 MHz, $CDCl_3$) δ 7.32–7.28 (m, 2H), 7.23–7.19 (m, 3H), 6.33 (d, J = 1.2 Hz, 1H), 5.71 (d, J = 1.3 Hz, 1H), 2.93 (t, J = 7.6 Hz, 2H), 2.76 (t, J = 7.6 Hz, 2H), 2.67–2.58 (m, 4H); ^{13}C NMR (101 MHz, $CDCl_3$) δ 208.8, 172.1, 140.9, 138.6, 128.53, 128.51, 128.3, 126.2, 44.3, 41.6, 29.8, 25.8; HRMS (ESI) m/z calcd. for $C_{14}H_{15}O_3$ [M-H]$^-$: 231.1027, found 231.1030.

3. Asymmetric Sequential Hydrogenations of γ- and δ-Ketoacids

3.1 Reaction conditions screening for asymmetric hydrogenation of C=C bond

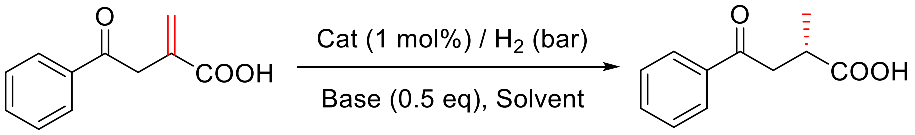

General Procedure: α-Methylene-γ-keto carboxylic acid **1a** (0.1 mmol, 19.0 mg), base (0.5 equiv) and Cat. (1 mol%) were added into a 10 mL vial. Solvent (0.5 mL) was transferred into the vial by a syringe in a nitrogen-filled glovebox. The vial was subsequently placed in an autoclave which was filled with hydrogen three times and charged with hydrogen to an indicated atm. The reaction mixture was stirred at room temperature. Hydrogen gas was released slowly and the reaction solvent was removed under reduced pressure. The residue was dissolved in DCM (2 mL) and acidified with HCl (3M) to pH = 1. After separation of the two layers, the aqueous phase was extracted with DCM (2 mL × 3). The combined organic layer was dried over anhydrous Na_2SO_4 and concentrated under reduced pressure to afford the crude product. The yield of both **2a** and **3a** were determined by ^{1}H NMR analysis of the crude product with 1,3,5-trimethylbenzene as an internal standard. The ee of the corresponding pure products of **2a** and **3a** were determined by HPLC using a Daicel Chiralpak AD-H (**2a**) and Daicel

Chiralcel OD-H (**3a**) columns.

The effect of different chiral catalysts on the hydrogenation of the C=C double bond of **1a** was screened firstly (Table S1). To our delight, our planar chiral RuPHOX-Ru (Cat. 1) presented the best results with **2a** being obtained in 99% yield with 94% ee (entry 1 *vs* entries 2-12).

Table S1 Screening of catalysts.[a]

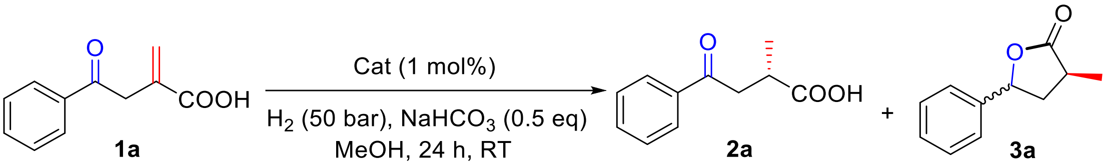

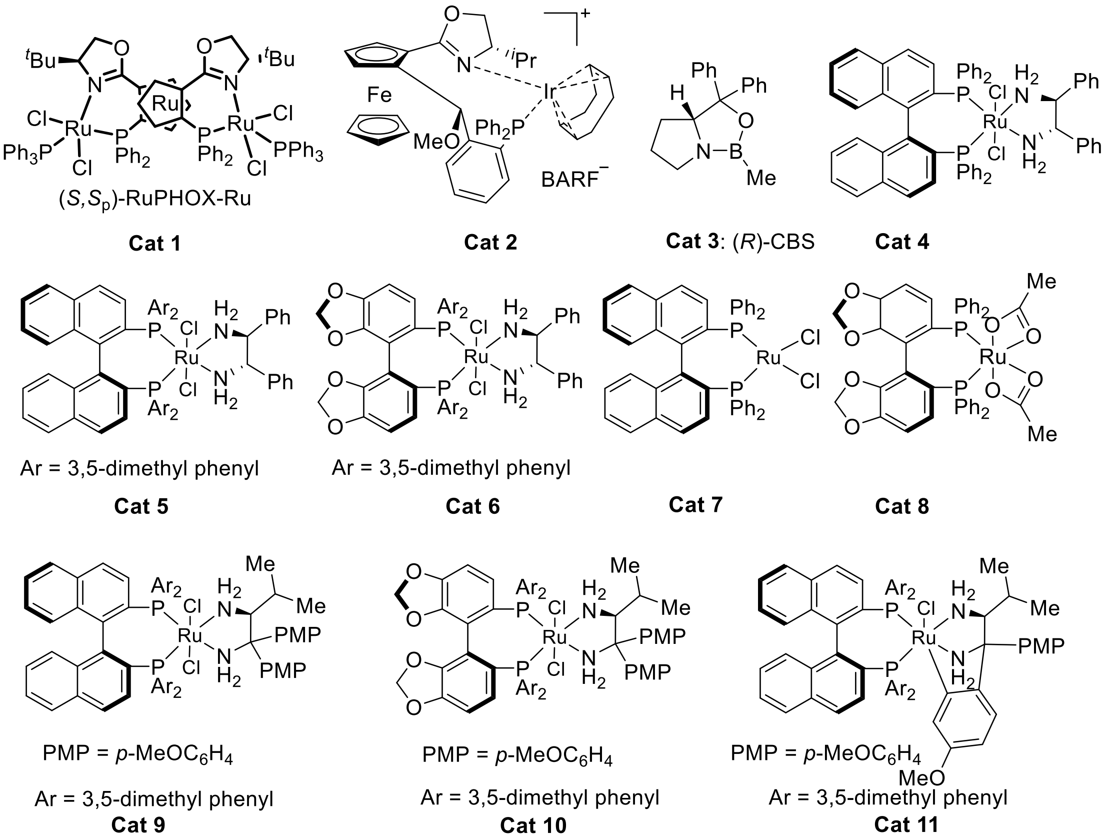

Entry	Catalyst	Yield (%) / **2a** / **3a**[b]	ee (%) / **2a** / **3a**[c]	dr / **3a**[b]
1	Cat 1	99/-	94/-	-
2	Cat 2	8/-	26/-	-
3[d]	Cat 3	20/-	11/-	-
4	Cat 4	94/-	24/-	-
5	Cat 5	96/-	50/-	-
6	Cat 6	97/-	58/-	-
7	Cat 7	None	-	-
8	Cat 8	None	-	-
9	Cat 9	98/-	26/-	-

10	Cat 10	99/-	32/-	-
11	Cat 11	94/-	53/-	-
12	Cat 12	20/-	11/-	-

[a]Reaction conditions: **1a** (0.1 mmol, 19.0 mg), Cat (1 mol%), $NaHCO_3$ (0.5 equiv, 4.2 mg), H_2 (50 bar), MeOH (0.5 mL), room temperature, 24 h; [b]Determined by ^1H NMR with 1,3,5-trimethylbenzene as an internal standard; [c]Determined by HPLC using a Chiralpak AD-H; [d](*R*)-CBS (0.1 equiv), BH_3•THF (1.2 equiv), THF as solvent and without H_2 and $NaHCO_3$.

Then, the effect of bases on the hydrogenation was screened (Table S2). It was shown that the chemoselective hydrogenation of the C=C double bond of **1a** could be achieved in high yields and enantioselectivities. When a weak inorganic base such as $NaHCO_3$ was used, the desired product **2a** could be obtained in 99% yield and > 99% ee (entry 1). It was found that the PPh_3 plays a significant role in the reaction and **2a** was obtained with a slightly lower ee of 94% when the above reaction was conducted in the absence of PPh_3 (entry 2). When a base with stronger alkalinity was used, somewhat lower ee values of **2a** were observed (entries 3-10). When strong bases such as KOH and ^{t}BuOK were employed, the subsequent (S,S_p)-RuPHOX-Ru-catalyzed hydrogenation of the C=O double bond of **2a** could occur but with poor asymmetric catalytic behavior of **3a** (entries 11 and 12). The chemoselective hydrogenation of the C=C double bond of **1a** could also be achieved with several organic bases, giving the desired product **2a** in yields up to 99% and 98% ee (entries 13-15).

Table S2 Screening of bases.[a]

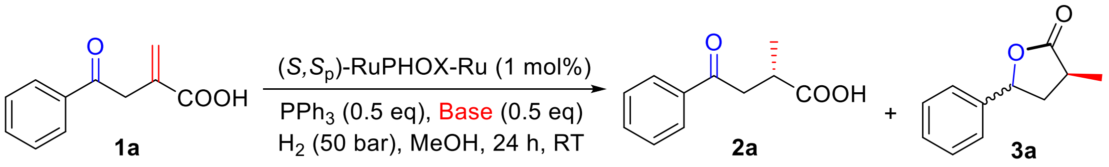

Entry	Base	Yield (%) of **2a/3a**[b]	ee (%) of **2a/3a**[c]	dr of **3a**[b]
1	$NaHCO_3$	99/-	>99/-	-
2[d]	$NaHCO_3$	99/-	94/-	-
3	$KHCO_3$	99/-	98/-	-
4	Na_2CO_3	99/-	98/-	-
5	K_2CO_3	99/-	94/-	-
6	Li_2CO_3	99/-	93/-	-
7	Cs_2CO_3	99/-	92/-	-
8	NaOH	99/-	91/-	-
9	NaOH (1.0 equiv)[e]	60/-	50/-	-
10	NaOH (2.0 equiv)[e]	25/-	24/-	-
11	KOH	88/12	90/99, 20	2.0:1
12	^{t}BuOK	86/14	90/99, 53	2.5:1

13	Et_3N	99/-	98/-	-
14	DIPEA	99/-	98/-	-
15	DBU	99/-	98/-	-

[a]Reaction conditions: **1a** (0.1 mmol, 19.0 mg), (*S*,*S*p)-RuPHOX-Ru (1 mol%, 1.7 mg), PPh_3 (0.5 equiv, 13.2 mg), base (0.5 equiv), H_2 (50 bar), MeOH (0.5 mL), room temperature, 24 h; [b]Determined by ^1H NMR with 1,3,5-trimethylbenzene as an internal standard; [c]Determined by HPLC using a Chiralpak AD-H (**2a**) or Chiralcel OD-H (**3a**) column; [d]Without PPh_3; [e]**1a** was decomposed when large amounts of strong base was used.

The proposed mechanism of the first step of the sequential hydrogenation is illustrated as follows (Figure S1). First, the catalyst RuPHOX–Ru is transformed to the activated Ru–H species **I** in the presence of H_2 and a base, which can coordinate with the C-C double bond of **1** to form complex **II**. The intermediate **III** is then formed after the hydrogen anion of the complex **II** is transferred to the carbon atom of the C=C double bond. Another H_2 molecule is subsequently activated *via* the intermediate **IV** in the similar manner mentioned above to release the Ru–H species **I**, providing the hydrogenated product **2** at the same time.

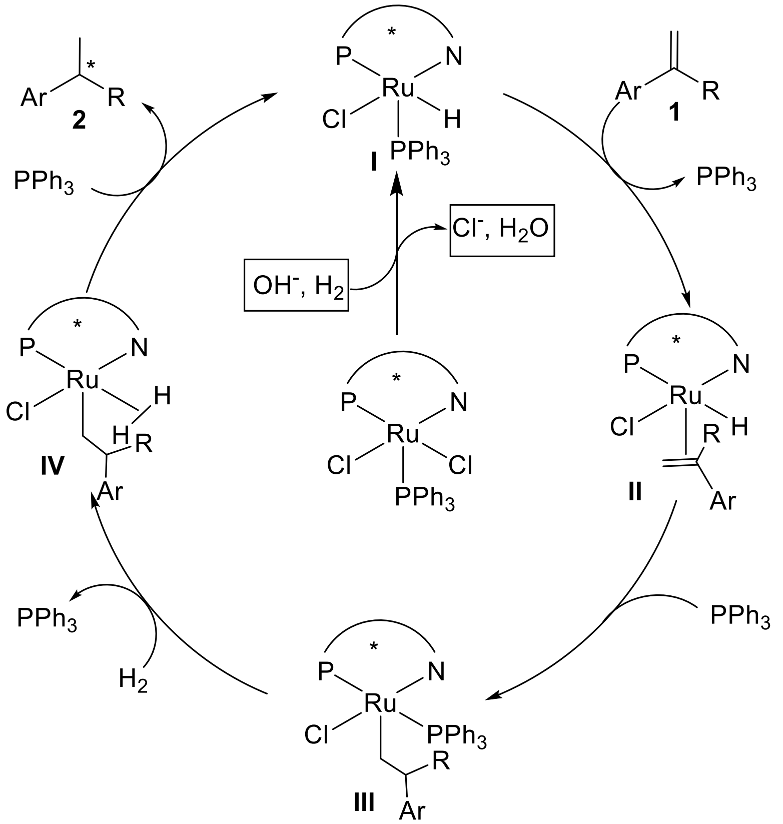

Figure S1. Proposed mechanism of the first step of the sequential hydrogenation

Next, the effect of solvents on the asymmetric hydrogenation was screened (Table S3). Protic solvents (entries 1-3) were better than aprotic solvents (entries 4-9). When a protic solvent such as MeOH or EtOH was used, the desired product **2a** could be obtained in 99% yield and > 99% ee (entries 1 and 2). Taking into consideration that

EtOH is environmentally benign, EtOH was selected as the best solvent for further investigations.

Table S3 Screening of solvents.[a]

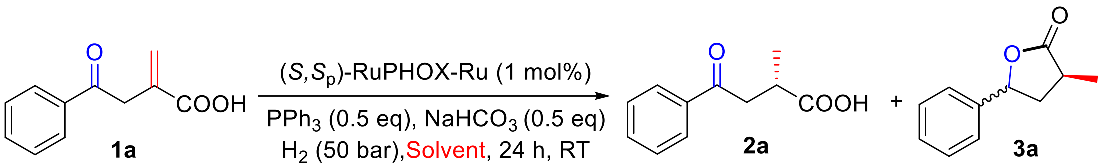

Entry	Base	Yield (%) of **2a**/**3a**[b]	ee (%) of **2a**/**3a**[c]	dr of **3a**[b]
1	MeOH	99/-	>99/-	-
2	EtOH	99/-	>99/-	-
3	^{i}PrOH	82/-	60/-	-
4	TFE	19/-	91/-	-
5	THF	39/-	74/-	-
6	Dioxane	79/-	27/-	-
7	DCM	28/-	90/-	-
8	Toluene	38/-	88/-	-
9	Toluene/H_2O (3:1)	70/-	81/-	-

[a]Reaction conditions: **1a** (0.1 mmol, 19.0 mg), (*S*,*S*$_p$)-RuPHOX-Ru (1 mol%, 1.7 mg), PPh_3 (0.5 equiv, 13.2 mg), $NaHCO_3$ (0.5 equiv, 4.2 mg), H_2 (50 bar), solvent (0.5 mL), room temperature, 24 h; [b]Determined by ^{1}H NMR with 1,3,5-trimethylbenzene as an internal standard; [c]Determined by HPLC using a Chiralpak AD-H column.

Subsequently, the effect of H_2 pressure on the asymmetric hydrogenation was screened (Table S4). It was shown that the reaction was not affected when the hydrogen pressure was reduced to 20 bar (entry 2).

Table S4 Screening of H_2 pressure.[a]

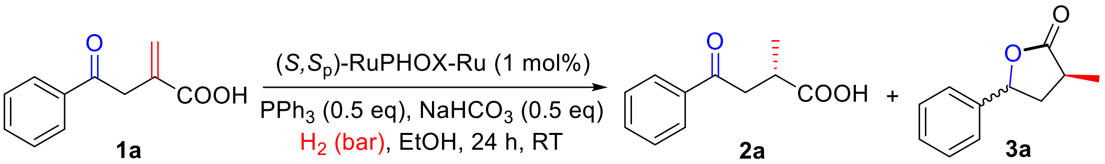

Entry	H_2/bar	Yield (%) of **2a** / **3a**[b]	ee (%) of **2a**/**3a**[c]	dr of **3a**[b]
1	50	99/-	>99/-	-
2	20	99/-	>99/-	-
3	10	99/-	99/-	-
4	5	95/-	90/-	-

[a]Reaction conditions: **1a** (0.1 mmol, 19.0 mg), (*S*,*S*$_p$)-RuPHOX-Ru (1 mol%, 1.7 mg), PPh_3 (0.5 equiv, 13.2 mg), $NaHCO_3$ (0.5 equiv, 4.2 mg), H_2 (bar), EtOH (0.5 mL), room temperature, 24 h; [b]Determined by ^{1}H NMR with 1,3,5-trimethylbenzene as an internal standard; [c]Determined by HPLC using a Chiralpak AD-H column.

Then, the effect of bases on the hydrogenation was again screened with EtOH as the solvent under 20 bar hydrogen pressure (Table S5). The chemoselective hydrogenation

of the C=C double bond of **1a** could be achieved in high yields and good enantioselectivities when weak inorganic bases were used (entries 1-6), and $NaHCO_3$ was better than other weak inorganic bases (entry 1). It was found that the PPh_3 plays a significant role in the reaction and **2a** was obtained with a slightly lower ee of 92% when the above reaction was conducted in the absence of PPh_3 (entry 2). When strong bases such as NaOH, KOH and ^{t}BuOK were employed, the desired product **2a** could be obtained in 99% yields but with low enantioselectivities (entries 7 and 9). The chemoselective hydrogenation of the C=C double bond of **1a** could also occur when several organic bases were examined, and the desired product **2a** could be obtained in 99% yield but with slightly lower enantioselectivities (entries 10-12).

Table S5 Screening of bases.[a]

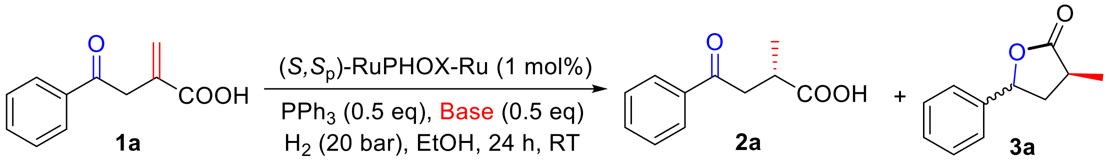

Entry	Base	Yield (%) of **2a**/**3a**[b]	ee (%) of **2a**/**3a**[c]	dr of **3a**[b]
1	$NaHCO_3$	99/-	>99/-	-
2[d]	$NaHCO_3$	99/-	92/-	-
3	$KHCO_3$	99/-	96/-	-
4	Na_2CO_3	99/-	95/-	-
5	K_2CO_3	99/-	93/-	-
6	Li_2CO_3	99/-	92/-	-
7	NaOH	99/-	74/-	-
8	KOH	99/-	68/-	-
9	^{t}BuOK	99/-	66/-	-
10	Et_3N	99/-	97/-	-
11	DIPEA	99/-	97/-	-
12	DBU	99/-	96/-	-

[a]Reaction conditions: **1a** (0.1 mmol, 19.0 mg), (S,S_p)-RuPHOX-Ru (1 mol%, 1.7 mg), PPh_3 (0.5 equiv, 13.2 mg), base (0.5 equiv), H_2 (20 bar), EtOH (0.5 mL), room temperature, 24 h; [b]Determined by ^{1}H NMR with 1,3,5-trimethylbenzene as an internal standard; [c]Determined by HPLC using a Chiralpak AD-H column; [d]In absence of PPh_3.

Finally, the effect of reaction time on the asymmetric hydrogenation was screened (Table S6). It was shown that the reaction went to completion after 6 hours (entry 3).

Table S6 Screening of reaction time.[a]

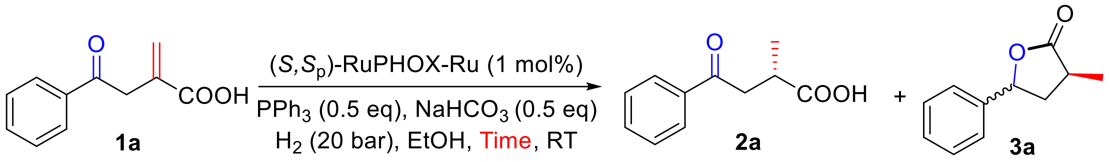

Entry	Time / h	Yield (%) of / **2a** / **3a**[b]	ee (%) of / **2a** / **3a**[c]	dr of / **3a**[b]
1	24	99/-	>99/-	-
2	12	99/-	>99/-	-
3	6	99/-	>99/-	-
4	3	96/-	>99/-	-

[a]Reaction conditions: **1a** (0.1 mmol, 19.0 mg), (*S,S*p)-RuPHOX-Ru (1 mol%, 1.7 mg), PPh_3 (0.5 equiv, 13.2 mg), $NaHCO_3$ (0.5 equiv, 4.2 mg), H_2 (20 bar), EtOH (0.5 mL), room temperature, time; [b]Determined by ^1H NMR with 1,3,5-trimethylbenzene as an internal standard; [c]Determined by HPLC using a Chiralpak AD-H column.

3.2 Reaction conditions screening for asymmetric hydrogenation of C=O bond

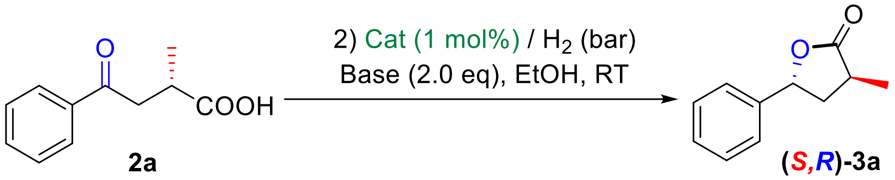

General Procedure: 2a (0.1 mmol, 19.2 mg), base (2.0 equiv) and Cat (1 mol%) were added into a 10 mL vial. EtOH (0.5 mL) was transferred into the vial by a syringe in a nitrogen-filled glovebox. The vial was subsequently placed in an autoclave which was replaced with hydrogen for three times and charged with hydrogen to an indicated atm. The reaction mixture was stirred at room temperature. Hydrogen gas was released slowly and the reaction solvent was removed under reduced pressure. The residue was dissolved with DCM (2 mL) and acidified with HCl (3M) to pH = 1. After separation of the two layers, the aqueous phase was extracted with DCM (2 mL × 3). The combined organic layer was dried over anhydrous Na_2SO_4 and concentrated under reduced pressure to afford the crude product. The dr and yield of **3a** were determined by ^1H NMR analysis of the crude product with 1,3,5-trimethylbenzene as an internal standard. The ee of pure **3a** was determined by HPLC using a Chiralcel OD-H column.

Based on EtOH being the best solvent for the first step, we then used EtOH as the solvent and isolated **2a** (>99% ee) as the model substrate, and investigated different catalysts to screen the reaction conditions of the hydrogenation of the C=O double bond of **2a**. Initially, we employed the (*S,S*p)-RuPHOX-Ru catalyst to examine the impact of both the quantity and alkalinity of bases (Table S7). These factors significantly influenced the reaction activity, albeit with very poor diastereoselectivity. It was shown that the asymmetric hydrogenation of the C=O double bond of **2a** can be achieved in

20% and 49% yields using 3.0 equiv and 4.0 equiv of NaOH, repectively, but low diastereoselectivities were observed (entries 1-6). When a weak inorganic bases such as $NaHCO_3$ (4.0 equiv) was used, the desired product **3a** was not obtained (entry 7). When a stronger base such as KOH (2.0 equiv or 3.0 equiv) was employed, the following (S,S_p)-RuPHOX-Ru catalyzed hydrogenation of the C=O double bond of **2a** could occur in 40% or 80% yields but with low diastereoselectivities (entries 8 and 9).

Table S7 Screening of the amount of base[a]

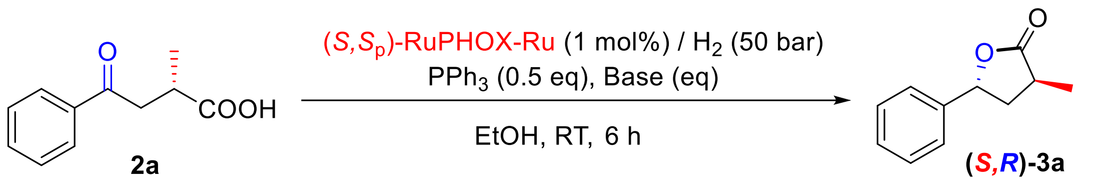

Entry	Base / equiv	Yield (%)[b]	ee (%)[c]	dr[b]
1	NaOH / 0.2	None	-	-
2	NaOH / 0.5	None	-	-
3	NaOH / 1.0	None	-	-
4	NaOH / 2.0	None	-	-
5	NaOH / 3.0	20	>99, 96	1.1:1
6	NaOH / 4.0	49	>99, 94	1.3:1
7	$NaHCO_3$ / 4.0	None	-	-
8	KOH / 2.0	40	>99, 96	1.5:1
9	KOH / 3.0	80	>99, 95	1.4:1

[a]Reaction conditions: **2a** (0.1 mmol, 19.2 mg), (S,S_p)-RuPHOX-Ru (1 mol%, 1.7 mg), PPh_3 (0.5 equiv, 13.2 mg), base (x equiv), H_2 (50 bar), EtOH (0.5 mL), room temperature, 6 h; [b]Determined by ^1H NMR with 1,3,5-trimethylbenzene as an internal standard; [c]Determined by HPLC using a Chiralcel OD-H column.

In view of the above reaction results, we therefore commenced our studies by screening a variety of known asymmetric hydrogenation catalysts, in the hope to find a catalyst that would enable the asymmetric hydrogenaiton of the C=O double bond of intermediate **2a** (Table S8). Firstly, the axis-unfixed biphenylphosphine-oxazoline ligand (BiphPHOX)[2] and ferrocene-based phosphine-oxazoline ligand[3] in our group as well as the well-known (R)-CBS catalyst[4] for the reduction of C=O double bond were tested, but the reaction reactivity and selectivity were very poor (entries 1–3). Then, the commonly used chiral Ru-complexes (**Cat 4**–**Cat 11**) and a strong base (NaOH) were employed for the asymmetric hydrogenation of ketones. Pleasingly, the chiral Ru(II)-diamine-diphosphine complexes (**Cat 4**-**Cat 6**) catalyzed the hydrogenation of the C=O double bond of the intermediate **2a** with excellent yields and enantioselectivities, albeit with low to moderate diastereomeric ratios (entries 4-6). Encouraged by these results, we proceeded to screen two additional Ru(II) complexes (**Cat 7** and **Cat 8**), but the

Table S8 Screening of catalysts.[a]

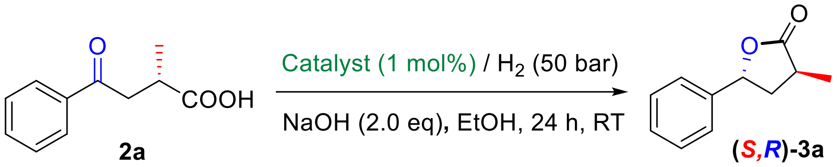

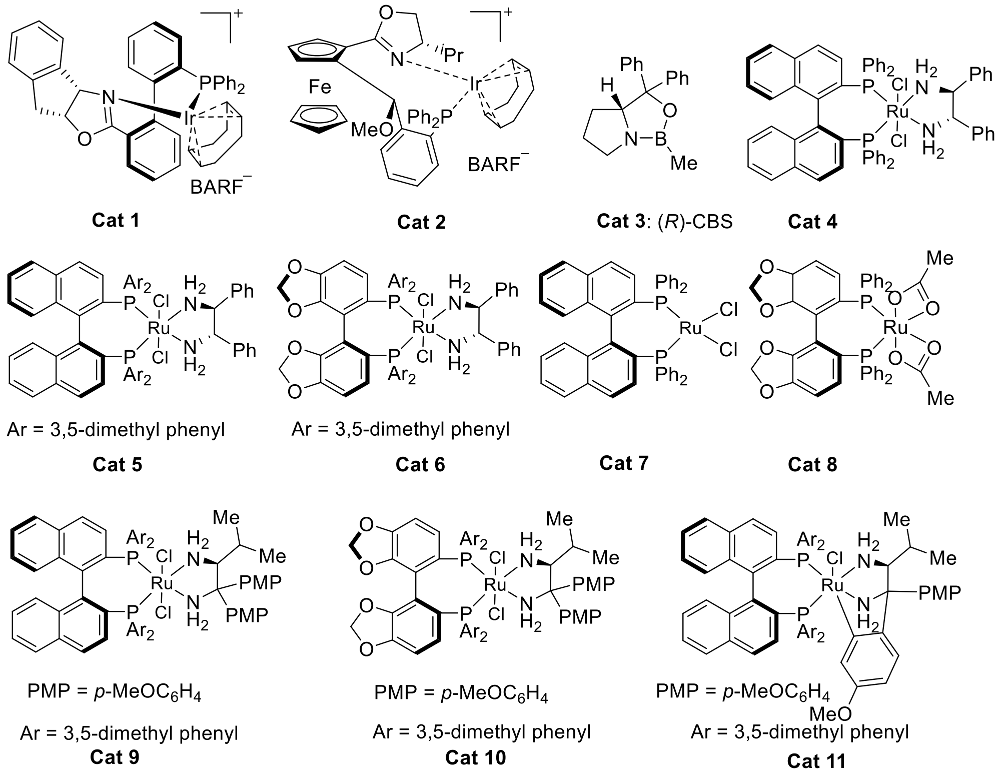

Entry	Catalyst	Yield (%)[b]	ee (%)[c]	dr[b]
1	Cat 1	None	-	-
2	Cat 2	10	99	3.0:1
3[d]	Cat 3	42	99	2.0:1
4	Cat 4	99	>99, 98	1.5:1
5	Cat 5	99	>99	6.2:1
6	Cat 6	99	>99	6.7:1
7	Cat 7	None	-	-
8	Cat 8	None	-	-
9	Cat 9	99	99	7.2:1
10	Cat 10	99	>99	>20:1
11	Cat 11	99	99	>20:1
12[e]	Cat 10	99	>99	>20:1

[a]Reaction conditions: **2a** (0.1 mmol, 19.2 mg), Cat (1 mol%), NaOH (2.0 equiv, 8.0 mg), H_2 (50 bar), EtOH (0.5 mL), room temperature, 24 h; [b]Determined by ^{1}H NMR with 1,3,5-trimethylbenzene as an internal standard; [c]Determined by HPLC using a Chiralcel OD-H column; [d](*R*)-CBS (0.1 equiv), BH_3•THF (1.2 equiv), THF as solvent and without H_2 and $NaHCO_3$. [e]PPh_3 (0.5 equiv, 13.2 mg).

reaction did not occur (entries 7 and 8). We can see that the diamine in the Ru(II) complexes play a vital role in catalyzing the asymmetric hydrogenation of the C=O double bond of **2a**. Therefore, Ru(II)-diamine-diphosphine complexes (**Cat 9-Cat 11**) were screened (entries 9-11). To our delight, **Cat 10** (*S*,*S*)-DM-Segphos-Ru catalyzed hydrogenation of the C=O double bond of **2a** afforded **3a** in 99% yield and >99% ee with >20:1 dr (entry 10). The PPh_3 had no influence to the reaction (entry 12).

Subsequently, the effect of H_2 pressure on the asymmetric hydrogenation of the C=O double bond was screened (Table S9). It was shown that the reaction was not affected when the hydrogen pressure was reduced to 20 bar (entry 2).

Table S9 Screening of H_2 pressure.[a]

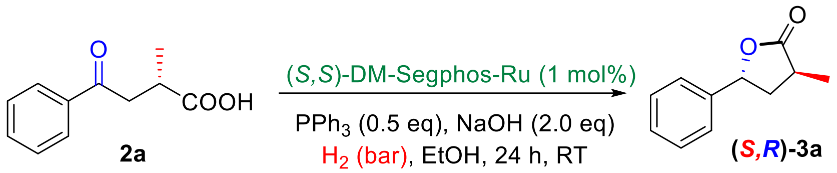

Entry	H_2/bar	Yield (%)[b]	ee (%)[c]	dr[b]
1	50	99	>99	>20:1
2	20	99	>99	>20:1
3	10	96	>99	>20:1
4	5	84	>99	>20:1

[a]Reaction conditions: **2a** (0.1 mmol, 19.2 mg), (*S,S*)-DM-Segphos-Ru (1 mol%, 1.2 mg), PPh_3 (0.5 equiv, 13.2 mg), NaOH (2.0 equiv, 8.0 mg), H_2 (bar), EtOH (0.5 mL), room temperature, 24 h; [b]Determined by ^1H NMR with 1,3,5-trimethylbenzene as an internal standard; [c]Determined by HPLC using a Chiralcel OD-H column.

Next, the effect of reaction time on the asymmetric hydrogenation of the C=O double bond was screened (Table S10). It was shown that the reaction could be completed after 6 hours (entry 3).

Table S10 Screening of reaction time.[a]

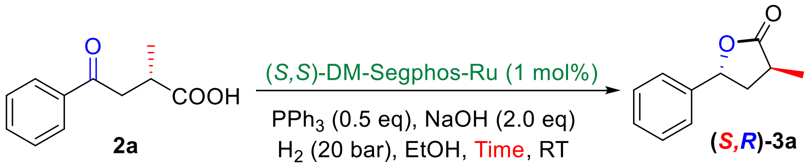

Entry	Time / h	Yield (%)[b]	ee (%)[c]	dr[b]
1	24	99	>99	>20:1
2	12	99	>99	>20:1
3	6	99	>99	>20:1
4	3	92	>99	>20:1

[a]Reaction conditions: **2a** (0.1 mmol, 19.2 mg), (*S,S*)-DM-Segphos-Ru (1 mol%, 1.2 mg), PPh_3 (0.5 equiv, 13.2 mg), NaOH (2.0 equiv, 8.0 mg), H_2 (20 bar), EtOH (0.5 mL), room temperature, time; [b]Determined by ^1H NMR with 1,3,5-trimethylbenzene as an internal standard; [c]Determined by HPLC using a Chiralcel OD-H column.

Then, the effect of bases on the asymmetric hydrogenation of the C=O double bond was investigated (Table S11). It was shown that the alkalinity of the base has a significant influence on the reactivity and stereoselectivity of the asymmetric hydrogenation of the C=O double bond. When a strong base such as NaOH (2.0 equiv) was used, the desired product **3a** could be obtained in 99% yield and > 99% ee as well as >20:1 dr (entry 1). Lowering the amount of NaOH to 1.5 equiv only provided **3a** in 67% yield (entry 2). When strong bases such as KOH and ^{t}BuOK were employed, hydrogenation of the C=O double bond of **2a** could also realized to afford **3a** in 99% yield and 99% ee with >20:1 dr (entries 3 and 4). The yield of **3a** was reduced to 65% when Cs_2CO_3 was used (entry 5). To our delight, hydrogenation of C=O double bond of **2a** could not occur when weak base was used (entries 6-12). Obviously, the hydrogenation of the C=O double bond of **2a** will not be affected by the previous RuPHOX-Ru catalyst system and the sequential hydrogenation should be possible using the two mutually compatible catalyst systems.

Table S11 Screening of bases.[a]

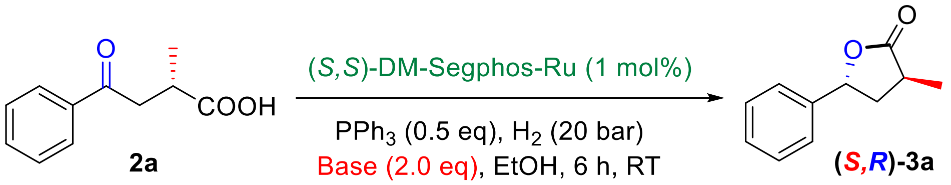

Entry	Base	Yield (%)[b]	ee (%)[c]	dr[b]
1	NaOH	99	>99	>20:1
2	NaOH (1.5 equiv)	67	99	14:1
3	KOH	99	99	>20:1
4	^{t}BuOK	99	99	>20:1
5	Cs_2CO_3	65	99	>20:1
6	Li_2CO_3	None	-	-
7	Na_2CO_3	None	-	-
8	$NaHCO_3$	None	-	-
9	$KHCO_3$	None	-	-
10	Et_3N	None	-	-
11	DABCO	None	-	-
12	DIPEA	None	-	-

[a]Reaction conditions: **2a** (0.1 mmol, 19.2 mg), (*S*,*S*)-DM-Segphos-Ru (1 mol%, 1.2 mg), PPh_3 (0.5 equiv, 13.2 mg), base (2.0 equiv), H_2 (20 bar), EtOH (0.5 mL), room temperature, 6 h; [b]Determined by ^1H NMR with 1,3,5-trimethylbenzene as an internal standard; [c]Determined by HPLC using a Chiralcel OD-H column.

Finally, in order to further investigate the compatibility of the two chiral Ru catalyst systems, ^{31}P NMR experiments of the two chiral Ru catalyst systems were performed

(Figure S2). To our delight, the results show that there is not ligand exchange besides the PPh_3 is oxidized by a small amount of oxygen in the solvent during the reaction from 1 to 16 h, which further indicates that the catalytic system is compatible in the bimetallic Ru/Ru-catalyzed asymmetric sequential hydrogenations.

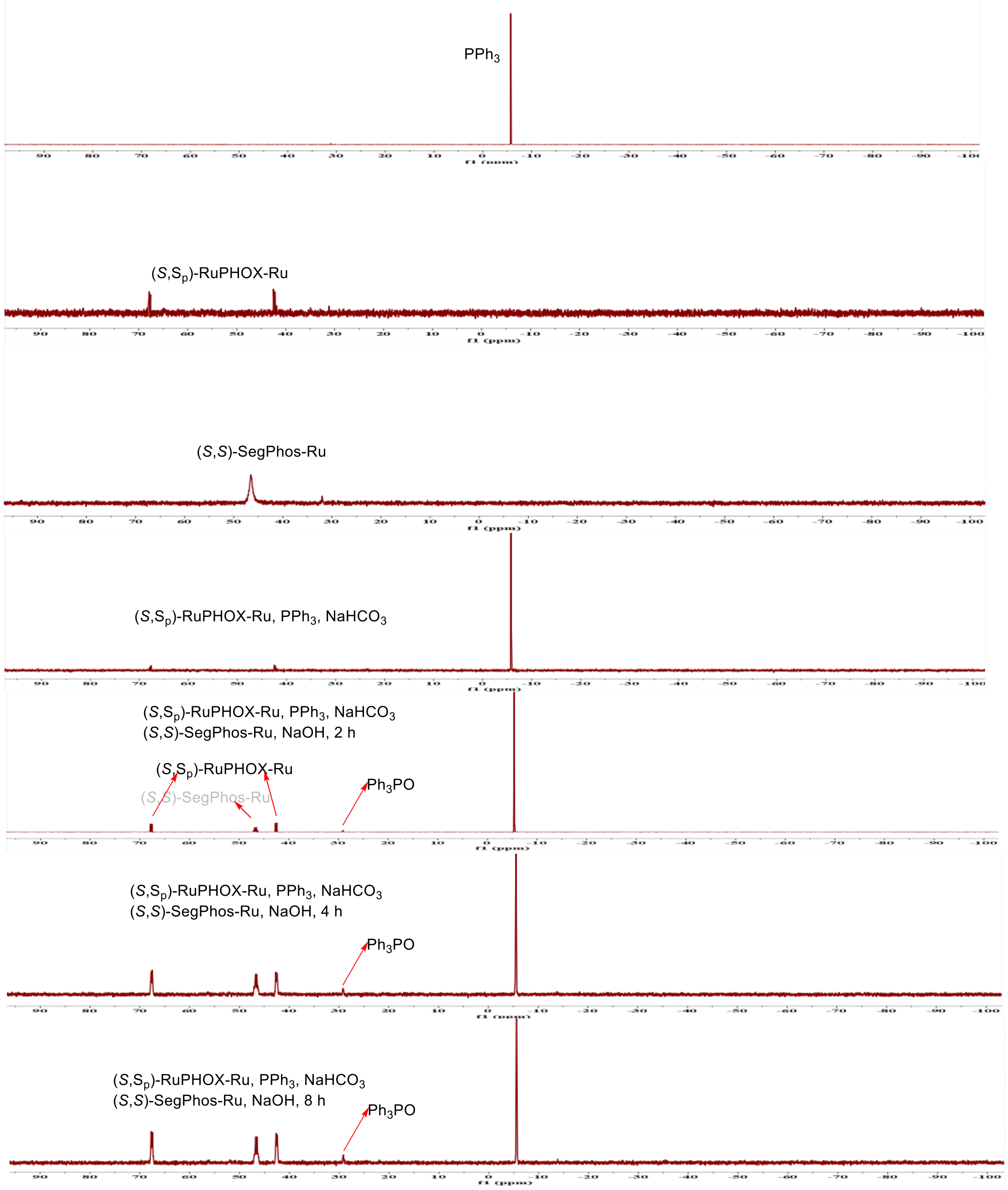

Figure S2. ^{1}P NMR experiment

3.3 Asymmetric Sequential Hydrogenation of *α*-methylene *γ*-ketoacids (1)

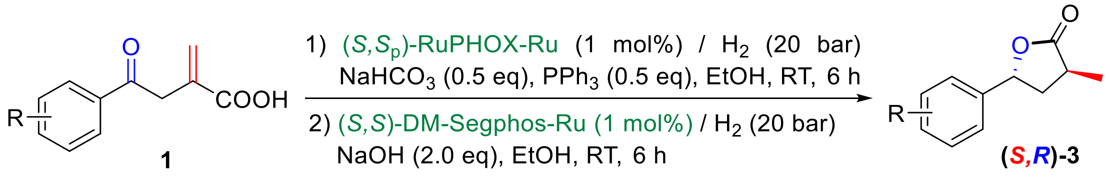

General Procedure: *a*-Methylene-*γ*-keto carboxylic acid **1** (0.2 mmol), $NaHCO_3$ (0.5 equiv, 8.4 mg), PPh_3 (0.5 equiv, 26.3 mg) and (*S,S*p)-RuPHOX-Ru (1 mol%,3.4 mg) were added into 10 mL vial. EtOH (1 mL) was transferred into the vial by a syringe in a nitrogen-filled glovebox. The vial was subsequently placed in an autoclave which was replaced with hydrogen for three times and charged with hydrogen to 20 atm. The reaction mixture was stirred at room temperature for 6 hours. The hydrogen gas was released slowly and NaOH (2.0 equiv, 16.0 mg) with EtOH (0.5 mL) was added into the vital, then (*S,S*)-DM-Segphos-Ru (1 mol%, 2.4 mg) with EtOH (0.5 mL) was transferred into the vital in a nitrogen-filled glovebox after the first step reaction was finished. The autoclave was again replaced with hydrogen for three times and charged with hydrogen to 20 atm. The reaction mixture was stirred at room temperature for another 6 hours. Hydrogen gas was released slowly and the reaction solvent was removed under reduced pressure. The residue was dissolved with DCM (2 mL) and acidified with HCl (3M) to pH = 1. DCM was separated and the aqueous phase was extracted with DCM (2 mL × 3). The combined organic layer was dried over anhydrous Na_2SO_4 and evoparated under reduced pressure to afford the crude product. The drs of the product were determined by ^{1}H NMR analysis of the crude product which was purified by column chromatography (PE/EtOAc = 10/1) to afford the pure product **3**. The ees of the corresponding pure products of **3** were determined by HPLC using chiral column.

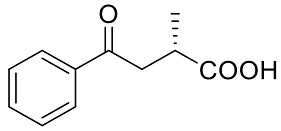

(*S*)-2-Methyl-4-oxo-4-phenylbutanoic acid (2a)[6]

White solid (37.6 mg, 98%, >99% ee). Mp: 120–122 °C; ^{1}H NMR (400 MHz, $CDCl_3$) *δ* 7.97 (d, *J* = 7.3 Hz, 2H), 7.57 (t, *J* = 7.3 Hz, 1H), 7.46 (t, *J* = 7.4 Hz, 2H), 3.47 (dd, *J* = 17.7, 7.6 Hz, 1H), 3.20–3.12 (m, 1H), 3.05 (dd, *J* = 17.7, 5.5 Hz, 1H), 1.32 (d, *J* = 7.1 Hz, 3H); ^{13}C NMR (101 MHz, $CDCl_3$) *δ* 197.9, 182.2, 136.5, 133.3, 128.7, 128.1, 41.7, 34.8, 17.1; HRMS (ESI) m/z calcd. for $C_{11}H_{12}NaO_3$ $[M+Na]^+$: 215.0679, found 215.0676; $[\alpha]_D^{20} = -34.9$ (*c* 0.235, CH_2Cl_2); HPLC conditions: Chiralpak AD-H column, *n*-hexane/*i*-PrOH = 97:3, flow rate = 0.8 mL/min, uv-vis detection at λ = 210 nm, t_R = 35.401 min (minor), 39.254 min (major).

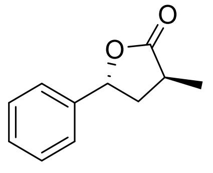

(*3S,5R*)-3-Methyl-5-phenyldihydrofuran-2(3*H*)-one (3a)[7a]

Colourless oil (34.5 mg, 98%, >20:1 dr, >99% ee). ^{1}H NMR (400 MHz, $CDCl_3$) δ 7.40–7.29 (m, 5H), 5.56 (dd, *J* = 7.8, 4.7 Hz, 1H), 2.78-2.68 (m, 1H), 2.47–2.32 (m, 2H), 1.33 (d, *J* = 7.3 Hz, 3H); ^{13}C NMR (101 MHz, $CDCl_3$) δ 178.0, 139.8, 128.8, 128.2, 125.0, 78.4, 38.4, 33.6, 15.4; HRMS (ESI) m/z calcd. for $C_{11}H_{12}NaO_2$ $[M+Na]^+$: 199.0730, found 199.0729; $[\alpha]_D^{20}$ = +3.0 (*c* 0.280, CH_2Cl_2); HPLC conditions: Chiralcel OD-H column, *n*-hexane/*i*-PrOH = 98:2, flow rate = 0.6 mL/min, uv-vis detection at λ = 210 nm, t_R = 27.232 min (minor), 28.580 min (major).

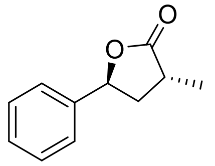

(*3R,5S*)-3-Methyl-5-phenyldihydrofuran-2(*3H*)-one (3a)[7a]

Colourless oil (34.5 mg, 98%, >20:1 dr, >99% ee). ^{1}H NMR (400 MHz, $CDCl_3$) δ 7.40–7.29 (m, 5H), 5.57 (dd, *J* = 7.7, 4.7 Hz, 1H), 2.78–2.68 (m, 1H), 2.48–2.32 (m, 2H), 1.33 (d, *J* = 7.3 Hz, 3H); ^{13}C NMR (101 MHz, $CDCl_3$) δ 179.9, 139.8, 128.8, 128.2, 125.0, 78.4, 38.4, 33.6, 15.4; HRMS (ESI) m/z calcd. for $C_{11}H_{12}NaO_2$ $[M+Na]^+$: 199.0730, found 199.0730; $[\alpha]_D^{20}$ = –2.6 (*c* 0.558, CH_2Cl_2); HPLC conditions: Chiralcel OD-H column, *n*-hexane/*i*-PrOH = 98:2, flow rate = 0.6 mL/min, uv-vis detection at λ = 210 nm, t_R = 26.110 min (major), 28.263 min (minor).

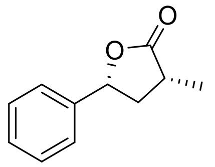

(*3R,5R*)-3-Methyl-5-phenyldihydrofuran-2(*3H*)-one (3a)[7a]

White solid (33.8 mg, 96%, >20:1 dr, >99% ee). Mp: 83–85 °C; ^{1}H NMR (400 MHz, $CDCl_3$) δ 7.41–7.32 (m, 5H), 5.36 (dd, *J* = 11.0, 5.5 Hz, 1H), 2.88–2.77 (m, 2H), 1.90–1.80 (m, 1H), 1.33 (d, *J* = 6.6 Hz, 3H); ^{13}C NMR (101 MHz, $CDCl_3$) δ 179.2, 139.1, 128.7, 128.5, 125.5, 79.2, 40.0, 36.4, 15.0; HRMS (ESI) m/z calcd. for $C_{11}H_{12}NaO_2$ $[M+Na]^+$: 199.0730, found 199.0730; $[\alpha]_D^{20}$ = +17.6 (*c* 0.983, CH_2Cl_2); HPLC conditions: Chiralcel OD-H column, *n*-hexane/*i*-PrOH = 98:2, flow rate = 0.6 mL/min, uv-vis detection at λ = 210 nm, t_R = 31.661 (major), 34.904 min (minor).

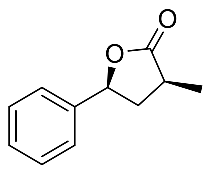

(*3S,5S*)-3-Methyl-5-phenyldihydrofuran-2(3*H*)-one (3a)[7a]

White solid (33.8 mg, 96%, >20:1 dr, >99% ee). Mp: 86–88 °C; ^{1}H NMR (400 MHz, $CDCl_3$) *δ* 7.41–7.31 (m, 5H), 5.35 (dd, *J* = 10.9, 5.4 Hz, 1H), 2.88–2.77 (m, 2H), 1.90–1.81 (m, 1H), 1.33 (d, *J* = 6.7 Hz, 3H); ^{13}C NMR (101 MHz, $CDCl_3$) *δ* 179.2, 139.1, 128.7, 128.5, 125.5, 79.2, 40.0, 36.4, 15.0; HRMS (ESI) m/z calcd. for $C_{11}H_{12}NaO_2$ $[M+Na]^+$: 199.0730, found 199.0730; $[\alpha]_D^{20}$ = –35.8 (*c* 0.979, CH_2Cl_2); HPLC conditions: Chiralcel OD-H column, *n*-hexane/*i*-PrOH = 98:2, flow rate = 0.6 mL/min, uv-vis detection at λ = 210 nm, t_R = 32.796 min (minor), 33.807 min (major).

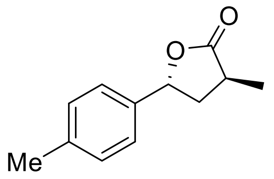

(*3S,5R*)-3-Methyl-5-(*p*-tolyl)dihydrofuran-2(*3H*)-one (3b)[6e]

White solid (37.6 mg, 99%, >20:1 dr, >99% ee). Mp: 68–69 °C; ^{1}H NMR (400 MHz, $CDCl_3$) *δ* 7.19 (s, 4H), 5.55 (dd, *J* = 7.8, 4.8 Hz, 1H), 2.79–2.69 (m, 1H), 2.46-2.40 (m, 1H), 2.35 (s, 3H), 2.37-2.32 (m, 1H), 1.33 (d, *J* = 7.3 Hz, 3H); ^{13}C NMR (101 MHz, $CDCl_3$) *δ* 179.0, 137.0, 135.7, 128.4, 124.0, 77.4, 37.4, 32.7, 20.1, 14.4; HRMS (ESI) m/z calcd. for $C_{12}H_{14}NaO_2$ $[M+Na]^+$: 213.0886, found 213.0886; $[\alpha]_D^{20}$ = –9.0 (*c* 0.268, CH_2Cl_2); HPLC conditions: Chiralcel OZ-H column, *n*-hexane/*i*-PrOH = 98:2, flow rate = 0.6 mL/min, uv-vis detection at λ = 210 nm, t_R = 26.568 min (minor), 29.784 min (major).

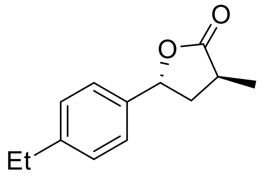

(*3S,5R*)-5-(4-Ethylphenyl)-3-methyldihydrofuran-2(*3H*)-one (3c)

Colourless oil (39.2 mg, 96%, >20:1 dr, >99% ee). ^{1}H NMR (400 MHz, $CDCl_3$) *δ* 7.22 (s, 4H), 5.55 (dd, *J* = 7.7, 6.0 Hz, 1H), 2.78-2.72 (m, 1H), 2.65 (q, *J* = 7.6 Hz, 2H), 2.48-2.41 (m, 1H), 2.37–2.32 (m, 1H), 1.33 (d, *J* = 7.2 Hz, 3H), 1.23 (t, *J* = 7.6 Hz, 3H); ^{13}C NMR (101 MHz, $CDCl_3$) *δ* 179.0, 143.4, 135.9, 127.2, 124.0, 77.4, 37.3, 32.7, 27.5, 14.5, 14.4; HRMS (ESI) m/z calcd. for $C_{13}H_{16}NaO_2$ $[M+Na]^+$: 227.1043, found 227.1043; $[\alpha]_D^{20}$ = –7.9 (*c* 0.152, CH_2Cl_2); HPLC conditions: Chiralcel OD-H column, *n*-hexane/*i*-PrOH = 98:2, flow rate = 0.6 mL/min, uv-vis detection at λ = 210 nm, t_R = 16.586 min (major), 19.258 min (minor).

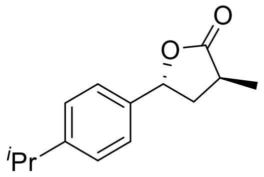

(*3S,5R*)-5-(4-Isopropylphenyl)-3-methyldihydrofuran-2(*3H*)-one (3d)

Colourless oil (42.7 mg, 98%, >20:1 dr, >99% ee). ^{1}H NMR (400 MHz, $CDCl_3$) δ 7.23 (s, 4H), 5.55 (dd, J = 7.7, 4.6 Hz, 1H), 2.94-2.86 (m, 1H), 2.80-2.70 (m, 1H), 2.49-2.42 (m, 1H), 2.37-2.30 (m, 1H), 1.33 (d, J = 7.3 Hz, 3H), 1.24 (d, J = 7.0 Hz, 6H); ^{13}C NMR (101 MHz, $CDCl_3$) δ 179.0, 148.0, 136.0, 125.8, 124.0, 77.4, 37.3, 32.8, 32.7, 22.9, 14.4; HRMS (ESI) m/z calcd. for $C_{14}H_{18}NaO_2$ $[M+Na]^+$: 241.1199, found 241.1198; $[\alpha]^{20}_{D}$ = –8.4 (c 0.325, CH_2Cl_2); HPLC conditions: Chiralcel OZ-H column, *n*-hexane/*i*-PrOH = 98:2, flow rate = 0.6 mL/min, uv-vis detection at λ = 210 nm, t_R = 19.879 min (minor), 23.237 min (major).

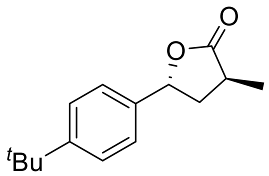

(*3S,5R*)-5-(4-(*tert*-Butyl)phenyl)-3-methyldihydrofuran-2(*3H*)-one (3e)[7a]

Brown solid (45.9 mg, 99%, >20:1 dr, >99% ee). Mp: 61–63 °C; ^{1}H NMR (400 MHz, $CDCl_3$) δ 7.34–7.31 (m, 2H), 7.18–7.15 (m, 2H), 5.49 (dd, J = 7.8, 4.6 Hz, 1H), 2.72–2.64 (m, 1H), 2.42-2.35 (m, 1H), 2.30-2.23 (m, 1H), 1.26 (d, J = 7.4 Hz, 3H), 1.24 (s, 9H); ^{13}C NMR (101 MHz, $CDCl_3$) δ 179.0, 150.2, 135.6, 124.6, 123.8, 77.3, 37.2, 33.6, 32.6, 30.3, 14.4; HRMS (ESI) m/z calcd. for $C_{15}H_{20}NaO_2$ $[M+Na]^+$: 255.1356, found 255.1356; $[\alpha]^{20}_{D}$ = –40.4 (c 0.104, CH_2Cl_2); HPLC conditions: Chiralcel OD-H column, *n*-hexane/*i*-PrOH = 98:2, flow rate = 0.6 mL/min, uv-vis detection at λ = 210 nm, t_R =14.896 min (major), 15.804 min (minor).

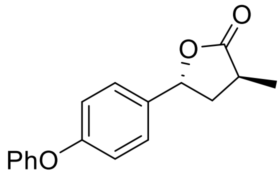

(*3S,5R*)-3-Methyl-5-(4-phenoxyphenyl)dihydrofuran-2(*3H*)-one (3f)

Gray solid (52.5 mg, 98%, >20:1 dr, >99% ee). Mp: 94–97 °C; ^{1}H NMR (400 MHz, $CDCl_3$) δ 7.27 (t, J = 8.0 Hz, 2H), 7.19 (d, J = 8.4 Hz, 2H), 7.05 (t, J = 7.4 Hz, 1H), 6.95–6.92 (m, 4H), 5.48 (dd, J = 7.6, 5.0 Hz, 1H), 2.73-2.64 (m, 1H), 2.41-2.34 (m, 1H), 2.30-2.23 (m, 1H), 1.27 (d, J = 7.3 Hz, 3H); ^{13}C NMR (101 MHz, $CDCl_3$) δ 178.8,

156.3, 155.7, 133.2, 128.8, 125.7, 122.6, 118.1, 117.8, 77.1, 37.2, 32.8, 14.5; HRMS (ESI) m/z calcd. for $C_{17}H_{16}NaO_3$ $[M+Na]^+$: 291.0992, found 291.0992; $[\alpha]_D^{20}$ = –11.2 (*c* 0.814, CH_2Cl_2); HPLC conditions: Chiralcel OD-H column, *n*-hexane/*i*-PrOH = 98:2, flow rate = 0.6 mL/min, uv-vis detection at λ = 210 nm, t_R =39.095 min (major), 54.683 min (minor).

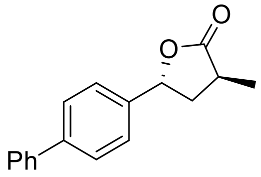

(*3S*,*5R*)-5-([1,1'-Biphenyl]-4-yl)-3-methyldihydrofuran-2(*3H*)-one (3g)

White solid (46.4 mg, 92%, 15:1 dr, >99% ee). Mp: 99–101 °C; ^{1}H NMR (400 MHz, $CDCl_3$) δ 7.62–7.57 (m, 4H), 7.45 (t, *J* = 7.5 Hz, 2H), 7.39–7.35 (m, 3H), 5.63 (dd, *J* = 7.6, 4.5 Hz, 1H), 2.83-2.73 (m, 1H), 2.53–2.46 (m, 1H), 2.43–2.33 (m, 1H), 1.36 (d, *J* = 7.3 Hz, 3H); ^{13}C NMR (101 MHz, $CDCl_3$) δ 178.9, 140.2, 139.4, 137.7, 127.8, 126.5, 126.4, 126.1, 124.4, 78.9, 37.4, 32.6, 14.4; HRMS (ESI) m/z calcd. for $C_{17}H_{16}NaO_2$ $[M+Na]^+$: 275.1043, found 275.1043; $[\alpha]_D^{20}$ = –7.9 (*c* 0.279, CH_2Cl_2); HPLC conditions: Chiralcel OD-H column, *n*-hexane/*i*-PrOH = 96:4, flow rate = 1.0 mL/min, uv-vis detection at λ = 210 nm, t_R =15.573 min (major), 23.541 min (minor).

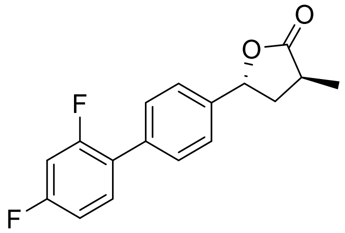

(*3S*,*5R*)-5-(2',4'-Difluoro-[1,1'-biphenyl]-4-yl)-3-methyldihydrofuran-2(*3H*)-one (3h)[7a]

Colourless oil (57.0 mg, 99%, >20:1 dr, >99% ee). ^{1}H NMR (400 MHz, $CDCl_3$) δ 7.53-7.50 (m, 2H), 7.42–7.36 (m, 3H), 6.98–6.89 (m, 2H), 5.62 (dd, *J* = 7.7, 4.6 Hz, 1H), 2.81-2.72 (m, 1H), 2.52-2.46 (m, 1H), 2.43-2.36 (m, 1H), 1.35 (d, *J* = 7.2 Hz, 3H); ^{13}C NMR (101 MHz, $CDCl_3$) δ 179.8, 162.3 (dd, *J* = 269.3, 12.0 Hz), 159.9 (dd, *J* = 270.5, 11.9 Hz), 139.3, 134.9, 131.4 (dd, *J* = 9.6, 4.9 Hz), 129.3 (d, *J* = 3.1 Hz), 125.2, 124.6 (dd, *J* = 13.6, 3.9 Hz), 111.7 (dd, *J* = 21.2, 3.8 Hz), 104.5 (dd, *J* = 26.8, 25.1 Hz), 78.1, 38.4, 33.6, 15.4. ^{19}F NMR (377 MHz, $CDCl_3$) δ –110.9, –113.6; HRMS (ESI) m/z calcd. for $C_{17}H_{14}F_2NaO_2$ $[M+Na]^+$: 311.0854, found 311.0855; $[\alpha]_D^{20}$ = –4.2 (*c* 0.472, CH_2Cl_2); HPLC conditions: Chiralpak AS-H column, *n*-hexane/*i*-PrOH = 93:7, flow rate = 1.0 mL/min, uv-vis detection at λ = 254 nm, t_R = 20.541 min (major), 29.204 min (minor).

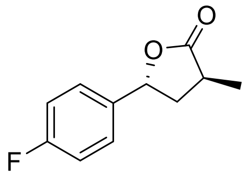

(3*S*,5*R*)-5-(4-Fluorophenyl)-3-methyldihydrofuran-2(3*H*)-one (3i)[7a]

White solid (38.4 mg, 99%, >20:1 dr, >99% ee). Mp: 86–87 °C; ^{1}H NMR (400 MHz, $CDCl_3$) *δ* 7.23–7.19 (m, 2H), 6.99 (t, *J* = 8.6 Hz, 2H), 5.47 (dd, *J* = 7.6, 5.1 Hz, 1H), 2.71-2.63 (m, 1H), 2.38-2.24 (m, 2H), 1.26 (d, *J* = 7.4 Hz, 3H); ^{13}C NMR (101 MHz, $CDCl_3$) *δ* 178.7, 161.5 (d, *J* = 247.7 Hz), 134.5 (d, *J* = 3.2 Hz), 125.8 (d, *J* = 8.3 Hz), 114.7 (d, *J* = 21.8 Hz), 76.8, 37.3, 32.7, 14.4. ^{19}F NMR (377 MHz, $CDCl_3$) *δ* –113.8; HRMS (ESI) m/z calcd. for $C_{11}H_{11}FNaO_2$ $[M+Na]^+$: 217.0635, found 217.0636; $[\alpha]_D^{20}$ = –1.0 (*c* 0.925, CH_2Cl_2); HPLC conditions: Chiralcel OD-H column, *n*-hexane/*i*-PrOH = 98:2, flow rate = 0.6 mL/min, uv-vis detection at λ = 210 nm, t_R =20.418 min (major), 23.853 min (minor).

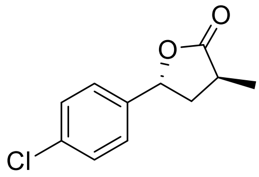

(3*S*,5*R*)-5-(4-Chlorophenyl)-3-methyldihydrofuran-2(3*H*)-one (3j)[7a]

White solid (41.2 mg, 98%, >20:1 dr, >99% ee). Mp: 104–106 °C; ^{1}H NMR (400 MHz, $CDCl_3$) *δ* 7.36–7.34 (m, 2H), 7.25-7.23 (m, 2H), 5.54 (dd, *J* = 7.3, 5.1 Hz, 1H), 2.76-2.66 (m, 1H), 2.43-2.34 (m, 2H), 1.33 (d, J = 7.3 Hz, 3H); ^{13}C NMR (101 MHz, $CDCl_3$) *δ* 178.6, 137.3, 133.0, 127.9, 125.4, 76.6, 37.3, 32.5, 14.4; HRMS (ESI) m/z calcd. for $C_{11}H_{11}ClNaO_2$ $[M+Na]^+$: 233.0340, found 233.0341; $[\alpha]_D^{20}$ = +0.7 (*c* 0.526, CH_2Cl_2); HPLC conditions: Chiralcel OD-H column, *n*-hexane/*i*-PrOH = 98:2, flow rate = 0.6 mL/min, uv-vis detection at λ = 210 nm, t_R =21.979 min (major), 24.438 min (minor).

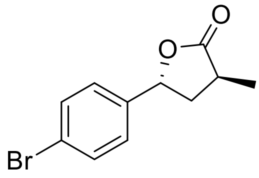

(3*S*,5*R*)-5-(4-Bromophenyl)-3-methyldihydrofuran-2(3*H*)-one (3k)

White solid (49.8 mg, 98%, >20:1 dr, >99% ee). Mp: 96–97 °C; ^{1}H NMR (400 MHz, $CDCl_3$) *δ* 7.51 (d, *J* = 8.3 Hz, 2H), 7.19 (d, *J* = 8.2 Hz, 2H), 5.52 (dd, *J* = 7.4, 4.9 Hz, 1H), 2.76–2.66 (m, 1H), 2.43–2.34 (m, 2H), 1.33 (d, *J* = 7.2 Hz, 3H); ^{13}C NMR (101 MHz, $CDCl_3$) *δ* 178.5, 137.8, 130.9, 125.7, 121.1, 76.6, 37.2, 32.5, 14.4; HRMS (ESI)

m/z calcd. for $C_{11}H_{11}BrNaO_2$ [M+Na]$^+$: 276.9835, found 276.9836; $[\alpha]_D^{20}$ = –6.1 (*c* 0.262, CH_2Cl_2); HPLC conditions: Chiralcel OD-H column, *n*-hexane/*i*-PrOH = 98:2, flow rate = 0.6 mL/min, uv-vis detection at λ = 210 nm, t_R =24.124 min (major), 26.720 min (minor).

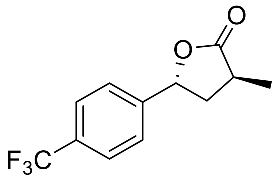

(*3S,5R*)-3-Methyl-5-(4-(trifluoromethyl)phenyl)dihydrofuran-2(*3H*)-one (3l)[7a]

Light yellow solid (47.8 mg, 98%, >20:1 dr, >99% ee). Mp: 52–54 °C; ^{1}H NMR (400 MHz, $CDCl_3$) δ 7.64 (d, *J* = 8.0 Hz, 2H), 7.43 (d, *J* = 8.0 Hz, 2H), 5.61 (t, *J* = 6.4 Hz, 1H), 2.76-2.66 (m, 1H), 2.43-2.40 (m, 2H), 1.34 (d, *J* = 7.3 Hz, 3H); ^{13}C NMR (101 MHz, $CDCl_3$) δ 178.4, 142.9, 129.4 (q, *J* = 32.8 Hz), 124.8 (q, *J* = 3.9 Hz), 124.2, 121.5, 76.4, 37.3, 32.4, 14.3; ^{19}F NMR (377 MHz, $CDCl_3$) δ –62.6; HRMS (ESI) m/z calcd. for $C_{12}H_{11}F_3NaO_2$ [M+Na]$^+$: 267.0603, found 267.0598; $[\alpha]_D^{20}$ = +11.0 (*c* 0.750, CH_2Cl_2); HPLC conditions: Chiralcel OD-OZ-H column, *n*-hexane/*i*-PrOH = 98:2, flow rate = 0.7 mL/min, uv-vis detection at λ = 210 nm, t_R = 58.477 min (minor), 60.647 min (major).

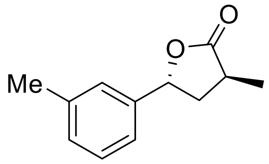

(*3S,5R*)-3-Methyl-5-(*m*-tolyl)dihydrofuran-2(*3H*)-one (3m)[7a]

Colourless oil (37.6 mg, 99%, >20:1 dr, >99% ee). ^{1}H NMR (400 MHz, $CDCl_3$) δ 7.29 (d, *J* = 8.2 Hz, 1H), 7.12 (q, *J* = 7.8 Hz, 3H), 5.55 (dd, *J* = 8.0, 5.0 Hz, 1H), 2.80–2.70 (m, 1H), 2.53–2.39(m, 2H), 2.37 (s, 3H), 1.34 (d, *J* = 7.2 Hz, 3H); ^{13}C NMR (101 MHz, $CDCl_3$) δ 179.0, 138.7, 137.5, 127.9, 127.6, 124.6 121.0, 77.4, 37.4, 32.6, 20.4, 14.4; HRMS (ESI) m/z calcd. for $C_{12}H_{14}NaO_2$ [M+Na]$^+$: 213.0886, found 213.0887; $[\alpha]_D^{20}$ = –2.4 (*c* 0.857, CH_2Cl_2); HPLC conditions: Chiralcel OD-H column, *n*-hexane/*i*-PrOH = 98:2, flow rate = 0.6 mL/min, uv-vis detection at λ = 210 nm, t_R =20.120 min (major), 23.108 min (minor).

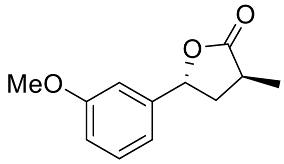

(3*S*,5*R*)-5-(3-Methoxyphenyl)-3-methyldihydrofuran-2(3*H*)-one (3n)[7a]

Colourless oil (40.8 mg, 99%, >20:1 dr, >99% ee). ^{1}H NMR (400 MHz, $CDCl_3$) δ 7.29 (d, J = 8.2 Hz, 1H), 6.87–6.83 (m, 3H), 5.53 (dd, J = 7.8, 4.9 Hz, 1H), 3.80 (s, 3H), 2.77–2.67 (m, 1H), 2.46–2.32 (m, 2H), 1.31 (d, J = 7.3 Hz, 3H); ^{13}C NMR (101 MHz, $CDCl_3$) δ 178.9, 158.9, 140.4, 128.8, 116.0, 112.5, 109.6, 77.1, 54.3, 37.3, 32.5, 14.4; HRMS (ESI) m/z calcd. for $C_{12}H_{14}NaO_3$ [M+Na]$^+$: 229.0835, found 229.0836; $[\alpha]_D^{20}$ = –3.4 (c 0.958, CH_2Cl_2); HPLC conditions: Chiralcel OD and Chiralpak IC-H column, *n*-hexane/*i*-PrOH = 95:5, flow rate = 1.0 mL/min, uv-vis detection at λ = 210 nm, t_R = 59.408 min (minor), 62.212 min (major).

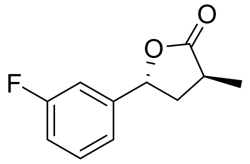

(3*S*,5*R*)-5-(3-Fluorophenyl)-3-methyldihydrofuran-2(3*H*)-one (3o)[7a]

Colourless oil (38.0 mg, 98%, >20:1 dr, >99% ee). ^{1}H NMR (400 MHz, $CDCl_3$) δ 7.34 (q, J = 7.7 Hz, 1H), 7.08–6.98 (m, 3H), 5.54 (dd, J = 7.6, 5.0 Hz, 1H), 2.75-2.68 (m, 1H), 2.45–2.35 (m, 2H), 1.32 (d, J = 7.3 Hz, 3H); ^{13}C NMR (101 MHz, $CDCl_3$) δ 178.5, 161.9 (d, J = 247.9 Hz), 141.4 (d, J = 7.0 Hz), 129.4 (d, J = 8.2 Hz), 119.5 (d, J = 3.0 Hz), 114.1 (d, J = 21.2 Hz), 111.1 (d, J = 22.7 Hz), 76.5 (d, J = 2.0 Hz), 37.2, 32.4, 14.3; ^{19}F NMR (377 MHz, $CDCl_3$) δ –112.0; HRMS (ESI) m/z calcd. for $C_{11}H_{11}FNaO_2$ [M+Na]$^+$: 217.0635, found 217.0637; $[\alpha]_D^{20}$ = +5.5 (c 0.851, CH_2Cl_2); HPLC conditions: Chiralcel OD and Chiralpak IC-H column, *n*-hexane/*i*-PrOH = 97:3, flow rate = 0.7 mL/min, uv-vis detection at λ = 210 nm, t_R = 56.484 min (minor), 58.560 min (major).

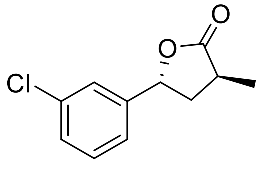

(3*S*,5*R*)-5-(3-Chlorophenyl)-3-methyldihydrofuran-2(3*H*)-one (3p)

Colourless oil (41.5 mg, 99%, >20:1 dr, >99% ee). ^{1}H NMR (400 MHz, $CDCl_3$) δ 7.33–7.28 (m, 3H), 7.19–7.17 (m, 1H), 5.53 (dd, J = 7.6, 5.1 Hz, 1H), 2.76–2.66 (m, 1H), 2.45–2.33 (m, 2H), 1.33 (d, J = 7.2 Hz, 3H); ^{13}C NMR (101 MHz, $CDCl_3$) δ 178.5, 140.9, 133.7, 129.1, 127.3, 124.1, 122.1, 76.4, 37.2, 32.4, 14.3; HRMS (ESI) m/z calcd. for $C_{11}H_{11}ClNaO_2$ [M+Na]$^+$: 233.0340, found 233.0340; $[\alpha]_D^{20}$ = +2.4 (c 0.813, CH_2Cl_2); HPLC conditions: Chiralcel OD-H column, *n*-hexane/*i*-PrOH = 98:2, flow rate = 0.6 mL/min, uv-vis detection at λ = 210 nm, t_R = 25.138 min (minor), 27.614 min (major).

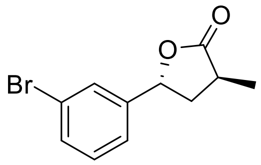

(*3S*,*5R*)-5-(3-Bromophenyl)-3-methyldihydrofuran-2(*3H*)-one (3q)

Colourless oil (50.3 mg, 99%, >20:1 dr, >99% ee). ^{1}H NMR (400 MHz, $CDCl_3$) δ 7.45 (d, J = 7.4 Hz, 2H), 7.27-7.22 (m, 2H), 5.53 (dd, J = 7.8, 5.2 Hz, 1H), 2.76-2.69 (m, 1H), 2.45-2.33 (m, 2H), 1.33 (d, J = 7.4 Hz, 3H); ^{13}C NMR (101 MHz, $CDCl_3$) δ 178.5, 141.1, 130.3, 129.3, 127.0, 122.5, 121.9, 76.4, 37.2, 32.4, 14.3; HRMS (ESI) m/z calcd. for $C_{11}H_{11}BrNaO_2$ [M+Na]$^+$: 276.9835, found 276.9835; $[\alpha]_D^{20}$ = +1.9 (c 0.844, CH_2Cl_2); HPLC conditions: Chiralcel OD-H column, *n*-hexane/*i*-PrOH = 98:2, flow rate = 0.6 mL/min, uv-vis detection at λ = 210 nm, t_R = 28.012 min (minor), 31.240 min (major).

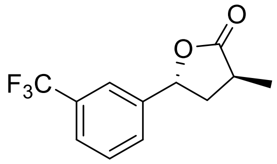

(*3S*,*5R*)-3-Methyl-5-(3-(trifluoromethyl)phenyl)dihydrofuran-2(*3H*)-one (3r)

Colourless oil (48.3 mg, 99%, >20:1 dr, >99% ee). ^{1}H NMR (400 MHz, $CDCl_3$) δ 7.60-7.50 (m, 4H), 5.61 (t, J = 6.4 Hz, 1H), 2.78-2.68 (m, 1H), 2.48-2.39 (m, 2H), 1.35 (d, J = 7.3 Hz, 3H); ^{13}C NMR (101 MHz, $CDCl_3$) δ 178.4, 139.9, 130.2 (q, J = 32.5 Hz), 128.4, 127.3, 124.1 (q, J = 3.7 Hz), 122.8 (q, J = 273.3 Hz), 120.8 (q, J = 3.8 Hz),76.5, 37.2, 32.5, 14.4. ^{19}F NMR (377 MHz, $CDCl_3$) δ –62.7; HRMS (ESI) m/z calcd. for $C_{12}H_{11}F_3NaO_2$ [M+Na]$^+$: 267.0603, found 267.0604; $[\alpha]_D^{20}$ = +6.0 (c 0.816, CH_2Cl_2); HPLC conditions: Chiralcel OD-H column, *n*-hexane/*i*-PrOH = 98:2, flow rate = 0.6 mL/min, uv-vis detection at λ = 210 nm, t_R =18.083 min (major), 21.231 min (minor).

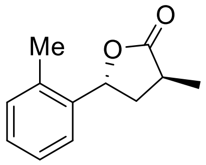

(*3S*,*5R*)-3-Methyl-5-(*o*-tolyl)dihydrofuran-2(*3H*)-one (3s)[7a]

Colourless oil (22.4 mg, 59%, 98% ee). ^{1}H NMR (400 MHz, $CDCl_3$) δ 7.21–7.10 (m, 4H), 5.64 (dd, J = 6.8, 5.7 Hz, 1H), 2.70–2.62 (m, 1H), 2.30–2.26 (m, 2H), 2.24 (s, 3H), 1.25 (d, J = 7.3 Hz, 3H); ^{13}C NMR (101 MHz, $CDCl_3$) δ 180.2, 137.9, 134.0, 130.8, 128.0, 126.3, 123.6, 76.2, 37.0, 33.3, 19.0, 15.4; HRMS (ESI) m/z calcd. for $C_{12}H_{14}NaO_2$ [M+Na]$^+$: 213.0886, found 213.0886; $[\alpha]_D^{20}$ = +33.0 (c 0.758, CH_2Cl_2);

HPLC conditions: Chiralcel OD-H column, *n*-hexane/*i*-PrOH = 98:2, flow rate = 0.6 mL/min, uv-vis detection at λ = 210 nm, 27.915 min (minor), t_R = 31.347 min (major).

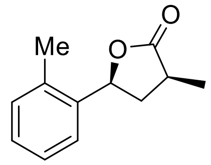

(3*S*,5*S*)-3-Methyl-5-(*o*-tolyl)dihydrofuran-2(3*H*)-one (3s)

Colourless oil (15.2 mg, 40%, 85% ee). ^{1}H NMR (400 MHz, $CDCl_3$) *δ* 7.33–7.31 (m, 1H), 7.19–7.08 (m, 3H), 5.47 (dd, *J* = 10.7, 5.4 Hz, 1H), 2.82–2.71 (m, 2H), 2.27 (s, 3H), 1.76–1.66 (m, 1H), 1.25 (d, *J* = 6.5 Hz, 3H); ^{13}C NMR (101 MHz, $CDCl_3$) *δ* 179.3, 137.2, 134.5, 130.6, 128.2, 126.5, 124.6, 76.9, 38.7, 36.3, 19.1, 15.1; HRMS (ESI) m/z calcd. for $C_{12}H_{14}NaO_2$ $[M+Na]^+$: 213.0886, found 213.0886; $[\alpha]_D^{20}$ = –35.5 (*c* 0.733, CH_2Cl_2); HPLC conditions: Chiralcel OD-H column, *n*-hexane/*i*-PrOH = 98:2, flow rate = 0.6 mL/min, uv-vis detection at λ = 210 nm, t_R = 34.935 min (minor), 38.997 min (major).

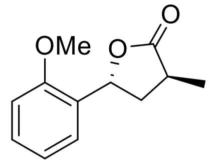

(3*S*,5*R*)-5-(2-Methoxyphenyl)-3-methyldihydrofuran-2(3*H*)-one (3t)

Colourless oil (24.3 mg, 59%, 99% ee). ^{1}H NMR (400 MHz, $CDCl_3$) *δ* 7.32–7.27 (m, 2H), 6.98–6.89 (m, 2H), 5.76 (dd, *J* = 8.0, 4.2 Hz, 1H), 3.85 (s, 3H), 2.73–2.66 (m, 1H), 2.42–2.29 (m, 2H), 1.31 (d, *J* = 7.2 Hz, 3H); ^{13}C NMR (101 MHz, $CDCl_3$) *δ* 179.6, 154.8, 128.1, 127.2, 124.1, 119.5, 109.4, 74.2, 54.3, 36.1, 32.3, 14.5; HRMS (ESI) m/z calcd. for $C_{12}H_{14}NaO_3$ $[M+Na]^+$: 229.0835, found 229.0836; $[\alpha]_D^{20}$ = +27.7 (*c* 0.205, CH_2Cl_2); HPLC conditions: Chiralcel OD-H column, *n*-hexane/*i*-PrOH = 98:2, flow rate = 0.6 mL/min, uv-vis detection at λ = 210 nm, t_R = 23.804 min (minor), 25.564 min (major).

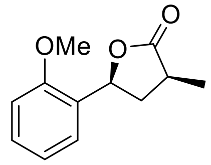

(3*S*,5*S*)-5-(2-Methoxyphenyl)-3-methyldihydrofuran-2(3*H*)-one (3t)

Colourless oil (16.5 mg, 40%, 97% ee). ^{1}H NMR (400 MHz, $CDCl_3$) *δ* 7.40 (d, *J* = 7.6 Hz, 1H), 7.30 (t, *J* = 9.2 Hz, 1H), 6.99 (t, *J* = 7.5 Hz, 1H), 6.89 (d, *J* = 8.2 Hz, 1H), 5.63

(dd, J = 10.2, 5.8 Hz, 1H), 3.84 (s, 3H), 2.94–2.78 (m, 2H), 1.80–1.71 (m, 1H), 1.31 (d, J = 6.8 Hz, 3H); ^{13}C NMR (101 MHz, $CDCl_3$) δ 178.6, 155.1, 128.1, 126.8, 124.6, 119.7, 109.4, 74.5, 54.3, 37.4, 35.1, 14.1; HRMS (ESI) m/z calcd. for $C_{12}H_{14}NaO_3$ $[M+Na]^+$: 229.0835, found 229.0836; $[\alpha]_D^{20}$ = –45.7 (c 0.112, CH_2Cl_2); HPLC conditions: Chiralcel OD-H column, n-hexane/i-PrOH = 98:2, flow rate = 0.6 mL/min, uv-vis detection at λ = 210 nm, t_R = 35.757 min (major), 44.059 min (minor).

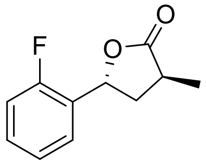

(3*S*,5*R*)-5-(2-Fluorophenyl)-3-methyldihydrofuran-2(3*H*)-one (3u)

Colourless oil (38.4 mg, 99%, >20:1 dr, >99% ee). ^{1}H NMR (400 MHz, $CDCl_3$) δ 7.35–7.30 (m, 2H), 7.17–7.05 (m, 2H), 5.77 (dd, J = 7.8, 4.8 Hz, 1H), 2.79–2.69 (m, 1H), 2.48–2.36 (m, 2H), 1.33 (d, J = 7.3 Hz, 3H); ^{13}C NMR (101 MHz, $CDCl_3$) δ 178.8, 158.4 (d, J = 247.2 Hz), 128.8 (d, J = 8.3 Hz), 126.3 (d, J = 13.0 Hz), 125.1 (d, J = 4.0 Hz), 123.3 (d, J = 3.6 Hz), 114.6 (d, J = 20.7 Hz), 72.7 (d, J = 3.6 Hz), 36.3 (d, J = 1.5 Hz), 32.3, 14.4; ^{19}F NMR (377 MHz, $CDCl_3$) δ –118.2; HRMS (ESI) m/z calcd. for $C_{11}H_{11}FNaO_2$ $[M+Na]^+$: 217.0635, found 217.0635; $[\alpha]_D^{20}$ = +4.9 (c 0.723, CH_2Cl_2); HPLC conditions: Chiralpak IC-AD-H column, n-hexane/i-PrOH = 97:3, flow rate = 0.8 mL/min, uv-vis detection at λ = 210 nm, t_R =38.187 min (major), 42.032 min (minor).

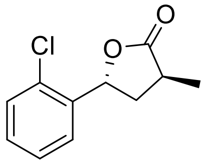

(3*S*,5*R*)-5-(2-Chlorophenyl)-3-methyldihydrofuran-2(3*H*)-one (3v)

White solid (37.8 mg, 90%, 15:1 dr, >99% ee). Mp: 49–50 °C; ^{1}H NMR (400 MHz, $CDCl_3$) δ 7.33–7.28 (m, 2H), 7.23–7.18 (m, 2H), 5.74 (dd, J = 7.9, 4.1 Hz, 1H), 2.66–2.56 (m, 1H), 2.43–2.30 (m, 2H), 1.26 (d, J = 7.2 Hz, 3H); ^{13}C NMR (101 MHz, $CDCl_3$) δ 179.0, 136.6, 130.0, 128.8, 128.2, 126.1, 124.4, 74.8, 36.0, 31.9, 14.2; HRMS (ESI) m/z calcd. for $C_{11}H_{11}ClNaO_2$ $[M+Na]^+$: 233.0340, found 233.0340; $[\alpha]_D^{20}$ = +47.9 (c 0.69, CH_2Cl_2); HPLC conditions: Chiralcel OJ-H column, n-hexane/i-PrOH = 98:2, flow rate = 0.7 mL/min, uv-vis detection at λ = 210 nm, t_R = 16.842 min (major), 18.257 min (minor).

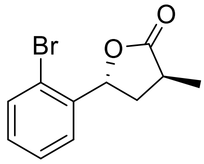

(3*S*,5*R*)-5-(2-Bromophenyl)-3-methyldihydrofuran-2(3*H*)-one (3w)

Colourless oil (50.3 mg, 99%, >20:1 dr, >99% ee). ^{1}H NMR (400 MHz, $CDCl_3$) δ 7.57 (d, *J* = 7.9 Hz, 1H), 7.37–7.32 (m, 2H), 7.22–7.16 (m, 1H), 5.75 (dd, *J* = 8.1, 3.8 Hz, 1H), 2.70–2.63 (m, 1H), 2.50–2.34 (m, 2H), 1.32 (d, *J* = 7.2 Hz, 3H); ^{13}C NMR (101 MHz, $CDCl_3$) δ 179.1, 138.1, 132.1, 128.5, 126.7, 124.6, 119.6, 76.6, 36.2, 31.8, 14.2; HRMS (ESI) m/z calcd. for $C_{11}H_{11}BrNaO_2$ [M+Na]$^+$: 276.9835, found 276.9836; $[\alpha]_D^{20}$ = +50.1 (*c* 1.085, CH_2Cl_2); HPLC conditions: Chiralcel OD-H column, *n*-hexane/*i*-PrOH = 98:2, flow rate = 0.6 mL/min, uv-vis detection at λ = 210 nm, t_R = 21.459 min (minor), 23.697 min (major).

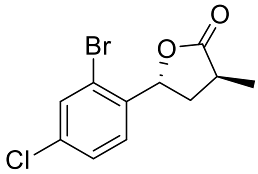

(3*S*,5*R*)-5-(2-Bromo-4-chlorophenyl)-3-methyldihydrofuran-2(3*H*)-one (3x)

Colourless oil (56.4 mg, 98%, 8:1 dr, 98% ee). ^{1}H NMR (400 MHz, $CDCl_3$) δ 7.59 (d, *J* = 2.0 Hz, 1H), 7.34–7.28 (m, 2H), 5.70 (dd, *J* = 8.0, 3.9 Hz, 1H), 2.69–2.61 (m, 1H), 2.51–2.43 (m, 1H), 2.38–2.32 (m, 1H), 1.33 (d, *J* = 7.2 Hz, 3H); ^{13}C NMR (101 MHz, $CDCl_3$) δ 178.7, 136.8, 133.6, 131.8, 127.0, 125.6, 119.8, 76.2, 36.1, 31.9, 14.2; HRMS (ESI) m/z calcd. for $C_{11}H_{10}BrClNaO_2$ [M+Na]$^+$: 310.9445, found 310.9444; $[\alpha]_D^{20}$ = +44.8 (*c* 0.643, CH_2Cl_2); HPLC conditions: Chiralcel OZ-H column, *n*-hexane/*i*-PrOH = 98:2, flow rate = 0.6 mL/min, uv-vis detection at λ = 210 nm, t_R = 100.980 min (major), 105.966 min (minor).

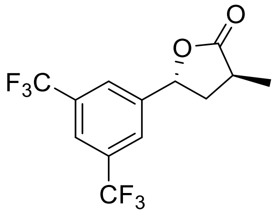

(3*S*,5*R*)-5-(3,5-bis(Trifluoromethyl)phenyl)-3-methyldihydrofuran-2(3*H*)-one (3y)

Colourless oil (61.8 mg, 99%, >20:1 dr, >99% ee). ^{1}H NMR (400 MHz, $CDCl_3$) δ 7.85 (s, 1H), 7.78 (s, 2H), 5.65 (dd, *J* = 7.6, 5.6 Hz, 1H), 2.79–2.70 (m, 1H), 2.53–2.41 (m, 2H), 1.37 (d, *J* = 7.2 Hz, 3H); ^{13}C NMR (101 MHz, $CDCl_3$) δ 178.8, 142.6, 132.3 (q, *J* = 33.7 Hz), 125.2 (d, *J* = 4.1 Hz), 123.0 (q, *J* = 273.8 Hz), 122.2 (q, *J* = 3.7 Hz), 76.7,

38.1, 33.5, 15.4; ^{19}F NMR (377 MHz, $CDCl_3$) δ –63.0; HRMS (ESI) m/z calcd. for $C_{13}H_{10}F_6NaO_2$ $[M+Na]^+$: 335.0477, found 335.0478, $[\alpha]_D^{20}$ = +10.0 (*c* 1.065, CH_2Cl_2); HPLC conditions: Chiralcel OZ-H column, *n*-hexane/*i*-PrOH = 98:2, flow rate = 0.6 mL/min, uv-vis detection at λ = 210 nm, t_R = 12.962 min (major), 14.563 min (minor).

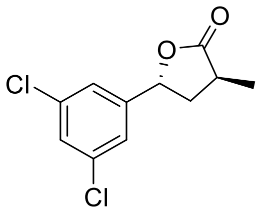

(3*S*,5*R*)-5-(3,5-Dichlorophenyl)-3-methyldihydrofuran-2(3*H*)-one (3z)

Colourless oil (48.3 mg, 99%, >20:1 dr, >99% ee). ^1H NMR (400 MHz, $CDCl_3$) δ 7.30 (s, 1H), 7.19 (s, 2H), 5.48 (t, *J* = 6.3 Hz, 1H), 2.74–2.65 (m, 1H), 2.43–2.35 (m, 2H), 1.32 (d, *J* = 7.2 Hz, 3H); ^{13}C NMR (101 MHz, $CDCl_3$) δ 179.1, 143.4, 135.6, 128.3, 123.5, 76.7, 38.1, 33.3, 15.3; HRMS (ESI) m/z calcd. for $C_{11}H_{10}Cl_2NaO_2$ $[M+Na]^+$: 266.9950, found 266.9951; $[\alpha]_D^{20}$ = +7.5 (*c* 0.997, CH_2Cl_2); HPLC conditions: Chiralcel OD-H column, *n*-hexane/*i*-PrOH = 99:1, flow rate = 0.3 mL/min, uv-vis detection at λ = 210 nm, t_R = 47.492 min (major), 51.622 min (minor).

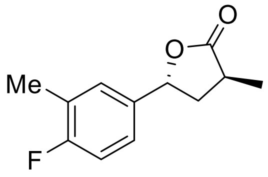

(3*S*,5*R*)-5-(4-Fluoro-3-methylphenyl)-3-methyldihydrofuran-2(3*H*)-one (3aa)

Colourless oil (41.2 mg, 99%, >20:1 dr, >99% ee). ^1H NMR (400 MHz, $CDCl_3$) δ 7.13–7.05 (m, 2H), 6.99 (t, *J* = 8.9 Hz, 1H), 5.50 (dd, *J* = 7.6, 5.1 Hz, 1H), 2.78–2.68 (m, 1H), 2.44–2.29 (m, 2H), 2.27 (s, 3H), 1.32 (d, *J* = 7.3 Hz, 3H); ^{13}C NMR (101 MHz, $CDCl_3$) δ 178.8, 160.0 (d, *J* = 246.4 Hz), 134.1 (d, *J* = 3.6 Hz), 127.2 (d, *J* = 5.4 Hz), 124.3 (d, *J* = 17.7 Hz), 123.0 (d, *J* = 8.4 Hz), 114.2 (d, *J* = 22.8 Hz), 76.9, 37.3, 32.7, 14.4, 13.6 (d, *J* = 3.6 Hz); ^{19}F NMR (377 MHz, $CDCl_3$) δ –118.1; HRMS (ESI) m/z calcd. for $C_{12}H_{13}FNaO_2$ $[M+Na]^+$: 231.0792, found 231.0789; $[\alpha]_D^{20}$ = –3.3 (*c* 0.763, CH_2Cl_2); HPLC conditions: Chiralcel OD-H column, *n*-hexane/*i*-PrOH = 98:2, flow rate = 0.6 mL/min, uv-vis detection at λ = 210 nm, t_R =16.791 min (major), 18.679 min (minor).

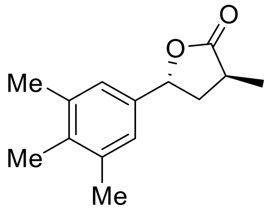

(3*S*,5*R*)-3-Methyl-5-(3,4,5-trimethylphenyl)dihydrofuran-2(3*H*)-one (3ab)

Colourless oil (43.2 mg, 99%, >20:1 dr, >99% ee). ^{1}H NMR (400 MHz, $CDCl_3$) δ 6.94 (s, 2H), 5.49 (dd, *J* = 7.8, 4.6 Hz, 1H), 2.80-2.71 (m, 1H), 2.47-2.41 (m, 2H), 2.29 (s, 6H), 2.17 (s, 3H), 1.33 (d, *J* = 7.3 Hz, 3H); ^{13}C NMR (101 MHz, $CDCl_3$) δ 179.1, 135.9, 135.4, 134.1, 123.1, 77.4, 37.4, 32.7, 19.6, 14.4, 14.2; HRMS (ESI) m/z calcd. for $C_{14}H_{18}NaO_2$ $[M+Na]^+$: 241.1199, found 241.1199; $[\alpha]_D^{20}$ = –7.9 (*c* 0.880, CH_2Cl_2); HPLC conditions: Chiralcel OD-H column, *n*-hexane/*i*-PrOH = 98:2, flow rate = 0.6 mL/min, uv-vis detection at λ = 210 nm, t_R =16.799 min (major), 18.479 min (minor).

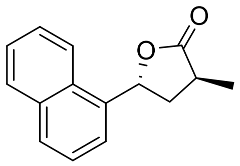

(3*S*,5*R*)-3-Methyl-5-(naphthalen-1-yl)dihydrofuran-2(3*H*)-one (3ac)[7a]

Colourless oil (44.7 mg, 99%, 15:1 dr, >99% ee). ^{1}H NMR (400 MHz, $CDCl_3$) δ 7.91 (d, *J* = 8.0 Hz, 1H), 7.82 (t, *J* = 8.0 Hz, 2H), 7.59–7.44 (m, 4H), 6.27 (t, *J* = 5.7 Hz, 1H), 2.77–2.67 (m, 1H), 2.58–2.54 (m, 2H), 1.35 (d, *J* = 7.1 Hz, 3H); ^{13}C NMR (101 MHz, $CDCl_3$) δ 179.2, 134.0, 132.8, 128.3, 128.1, 127.6, 125.5, 124.9, 124.2, 121.4, 120.2, 74.9, 36.6, 32.0, 14.1; HRMS (ESI) m/z calcd. for $C_{15}H_{14}NaO_2$ $[M+Na]^+$: 249.0886, found 249.0886; $[\alpha]_D^{20}$ = +72.4 (*c* 0.551, CH_2Cl_2); HPLC conditions: Chiralcel OZ-H column, *n*-hexane/*i*-PrOH = 98:2, flow rate = 0.6 mL/min, uv-vis detection at λ = 210 nm, t_R = 33.541 min (major), 42.212 min (minor).

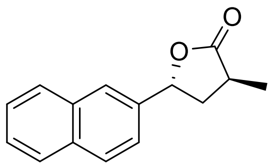

(3*S*,5*R*)-3-Methyl-5-(naphthalen-2-yl)dihydrofuran-2(3*H*)-one (3ad)

Light yellow solid (44.7 mg, 99%, >20:1 dr, >99% ee). Mp: 114–116 °C; ^{1}H NMR (400 MHz, $CDCl_3$) δ 7.89–7.78 (m, 4H), 7.54–7.48 (m, 2H), 7.37 (dd, *J* = 8.5, 1.9 Hz, 1H), 5.74 (dd, *J* = 7.8, 4.4 Hz, 1H), 2.81–2.73 (m, 1H), 2.57–2.50 (m, 1H), 2.46–2.39 (m, 1H), 1.36 (d, *J* = 7.2 Hz, 3H); ^{13}C NMR (101 MHz, $CDCl_3$) δ 179.0, 136.0, 132.0, 131.9, 127.8, 127.0, 126.7, 125.6, 125.4, 122.7, 121.8, 77.4, 37.2, 32.5, 14.4; HRMS (ESI)

m/z calcd. for $C_{15}H_{14}NaO_2$ $[M+Na]^+$: 249.0886, found 249.0886; $[\alpha]_D^{20}$ = –7.2 (*c* 1.241, CH_2Cl_2); HPLC conditions: Chiralcel OD-H column, *n*-hexane/*i*-PrOH = 98:2, flow rate = 0.6 mL/min, uv-vis detection at λ = 210 nm, t_R =34.917 min (major), 41.135 min (minor).

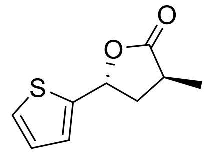

(*3S,5R*)-3-Methyl-5-(thiophen-2-yl)dihydrofuran-2(*3H*)-one (3ae)[7a]

Colourless oil (24.0 mg, 66%, 90% ee). ^1H NMR (400 MHz, $CDCl_3$) *δ* 7.31 (dd, *J* = 5.1, 1.2 Hz, 1H), 7.07–7.05 (m, 1H), 7.00 (dd, *J* = 5.0, 3.5 Hz, 1H), 5.76 (dd, *J* = 7.7, 4.0 Hz, 1H), 2.90–2.80 (m, 1H), 2.65–2.58 (m, 1H), 2.40–2.33 (m, 1H), 1.34 (d, *J* = 7.2 Hz, 3H); ^{13}C NMR (101 MHz, $CDCl_3$) *δ* 178.1, 141.4, 126.0, 124.9, 124.4, 73.9, 37.2, 32.9, 14.4; HRMS (ESI) m/z calcd. for $C_9H_{10}NaO_2S$ $[M+Na]^+$: 205.0294, found 205.0294; $[\alpha]_D^{20}$ = –48.6 (*c* 0.365, CH_2Cl_2); HPLC conditions: Chiralpak IE-H column, *n*-hexane/*i*-PrOH = 95:5, flow rate = 1.0 mL/min, uv-vis detection at λ = 210 nm, 26.050 min (minor), t_R = 31.082 min (major).

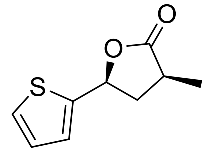

(*3S,5S*)-3-Methyl-5-(thiophen-2-yl)dihydrofuran-2(*3H*)-one (3ae)

White solid (12.0 mg, 33%, 76% ee). Mp: 62–64 °C; ^1H NMR (400 MHz, $CDCl_3$) *δ* 7.35 (dd, *J* = 5.0, 1.3 Hz, 1H), 7.11 (d, *J* = 3.6 Hz, 1H), 7.01 (dd, *J* = 5.1, 3.5 Hz, 1H), 5.57 (dd, *J* = 10.5, 5.0 Hz, 1H), 2.88–2.76 (m, 2H), 2.10–1.99 (m, 1H), 1.35 (d, *J* = 6.6 Hz, 3H); ^{13}C NMR (101 MHz, $CDCl_3$) *δ* 178.4, 141.3, 127.0, 126.4, 126.3, 75.2, 39.7, 36.4, 15.0; HRMS (ESI) m/z calcd. for $C_9H_{10}NaO_2S$ $[M+Na]^+$: 205.0294, found 205.0294; $[\alpha]_D^{20}$ = +16.6 (*c* 0.423, CH_2Cl_2); HPLC conditions: Chiralpak IE-H column, *n*-hexane/*i*-PrOH = 95:5, flow rate = 1.0 mL/min, uv-vis detection at λ = 210 nm, t_R = 28.237 min (minor), 29.542 min (major).

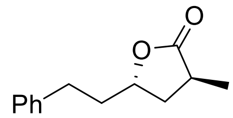

(*3S,5S*)-3-Methyl-5-phenethyldihydrofuran-2(*3H*)-one (3af)

White solid (26.9 mg, 66%, 98% ee). Mp: 76–77 °C; ^1H NMR (400 MHz, $CDCl_3$) *δ* 7.33–7.28 (m, 2H), 7.23–7.19 (m, 3H), 4.35–4.28 (m, 1H), 2.88–2.81 (m, 1H), 2.77–2.71 (m, 1H), 2.70–2.61 (m, 1H), 2.50–2.43 (m, 1H), 2.09–2.00 (m, 1H), 1.98–1.88 (m, 1H), 1.57–1.48 (m, 1H), 1.27 (d, *J* = 7.1 Hz, 3H); ^{13}C NMR (101 MHz, $CDCl_3$) *δ* 179.5,

140.8, 128.6, 128.5, 126.2, 77.6, 37.3(2), 37.3(0), 35.9, 31.7, 15.2; HRMS (ESI) m/z calcd. for $C_{13}H_{16}NaO_2$ $[M+Na]^+$: 227.1043, found 227.1042; $[\alpha]_D^{20}$ = +36.6 (*c* 0.448, CH_2Cl_2); HPLC conditions: Chiralcel OJ-H column, *n*-hexane/*i*-PrOH = 95:5, flow rate = 0.8 mL/min, uv-vis detection at λ = 210 nm, t_R = 30.916 min (major), 47.449 min (minor).

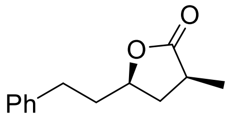

(3*S*,5*R*)-3-Methyl-5-phenethyldihydrofuran-2(3*H*)-one (3af)

Colourless oil (13.5 mg, 33%, 98% ee). ^{1}H NMR (400 MHz, $CDCl_3$) δ 7.32–7.28 (m, 2H), 7.23–7.19 (m, 3H), 4.53–4.47 (m, 1H), 2.85–2.79 (m, 1H), 2.76–2.67 (m, 2H), 2.16–2.09 (m, 1H), 2.06–1.97 (m, 2H), 1.91–1.82 (m, 1H), 1.28 (d, *J* = 7.3 Hz, 3H); ^{13}C NMR (101 MHz, $CDCl_3$) δ 180.0, 140.8, 128.6, 128.5, 126.2, 77.4, 37.3, 35.5, 34.0, 31.7, 15.9; HRMS (ESI) m/z calcd. for $C_{13}H_{16}NaO_2$ $[M+Na]^+$: 227.1043, found 227.1044; $[\alpha]_D^{20}$ = –55.2 (*c* 0.287, CH_2Cl_2); HPLC conditions: Chiralcel OJ-H column, *n*-hexane/*i*-PrOH = 95:5, flow rate = 0.8 mL/min, uv-vis detection at λ = 210 nm, t_R = 37.867 min (minor), 56.262 min (major).

3.4 Asymmetric Sequential Hydrogenation of *α*-methylene *δ*-ketoacids (4)

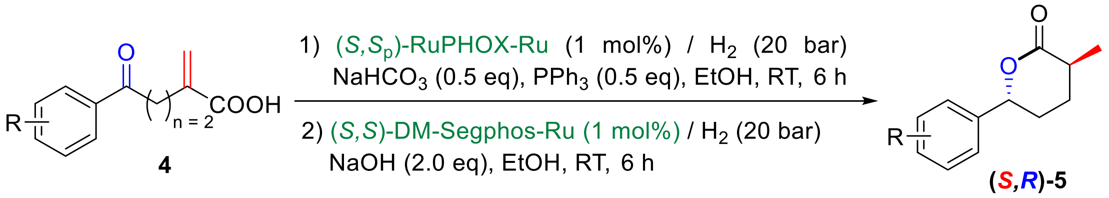

General Procedure: *α*-Methylene-*δ*-keto carboxylic acid **4** (0.2 mmol), $NaHCO_3$ (0.5 equiv, 8.4 mg), PPh_3 (0.5 equiv, 26.3 mg) and (*S,S*p)-RuPHOX-Ru (1 mol%, 3.4 mg) were added into 10 mL vial. EtOH (1.0 mL) was transferred into the vial by a syringe in a nitrogen-filled glovebox. The vial was subsequently placed in an autoclave which was replaced with hydrogen for three times and charged with hydrogen to 20 atm. The reaction mixture was stirred at room temperature for 6 hours. The hydrogen gas was released slowly and NaOH (2.0 equiv, 16.0 mg) with EtOH (0.5 mL) was added into the vital, then (*S,S*)-DM-Segphos-Ru (1 mol%, 2.4 mg) with EtOH (0.5 mL) was transferred into the vital in a nitrogen-filled glovebox after the first step reaction was finished. The autoclave was again replaced with hydrogen for three times and charged with hydrogen to 20 atm. The reaction mixture was stirred at room temperature for another 6 hours. Hydrogen gas was released slowly and the reaction solvent was removed under reduced pressure and residue was dissolved with DCM (2.0 mL) and acidified with HCl (3M) to pH = 1. DCM was separated and the aqueous phase was

extracted with DCM (2.0 mL × 3). The combined organic layer was dried over anhydrous Na_2SO_4 and evoparated under reduced pressure to afford the crude product. The drs of product were determined by ^{1}H NMR analysis of the crude product which was purified by column chromatography (PE/EtOAc = 10/1) to afford the pure product **4**. The ees of the corresponding pure products of **4** were determined by HPLC using chiral column.

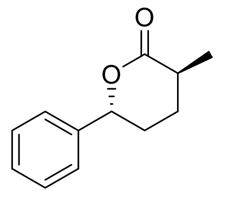

(*3S,6R*)-3-Methyl-6-phenyltetrahydro-*2H*-pyran-2-one (5a)

White solid (37.2 mg, 98%, >20:1 dr, >99% ee). Mp: 122–123 °C; ^{1}H NMR (400 MHz, $CDCl_3$) δ 7.39–7.29 (m, 5H), 5.34 (dd, J = 11.2, 3.3 Hz, 1H), 2.61–2.52 (m, 1H), 2.21–2.09 (m, 2H), 1.97–1.86 (m, 1H), 1.79–1.68 (m, 1H), 1.36 (d, J = 7.1 Hz, 3H); ^{13}C NMR (101 MHz, $CDCl_3$) δ 173.1, 139.0, 127.5, 127.2, 124.6, 81.8, 35.0, 30.6, 27.6, 16.4; HRMS (ESI) m/z calcd. for $C_{12}H_{14}NaO_2$ [M+Na]$^+$: 213.0886, found 213.0885; $[\alpha]_D^{20}$ = +24.0 (*c* 0.909, CH_2Cl_2); HPLC conditions: Chiralcel OD-OJ-H column, *n*-hexane/*i*-PrOH = 92:8, flow rate = 1.0 mL/min, uv-vis detection at λ = 210 nm, t_R = 38.588 min (minor), 42.082 min (major).

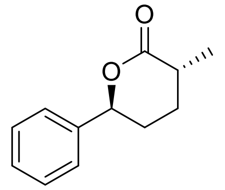

(*3R,6S*)-3-Methyl-6-phenyltetrahydro-*2H*-pyran-2-one (5a)[7b]

White solid (37.2 mg, 98%, >20:1 dr, >99% ee). Mp: 119–121 °C; ^{1}H NMR (400 MHz, $CDCl_3$) δ 7.39–7.29 (m, 5H), 5.34 (dd, J = 11.0, 3.3 Hz, 1H), 2.62–2.52 (m, 1H), 2.22–2.08 (m, 2H), 1.98–1.88 (m, 1H), 1.78–1.68 (m, 1H), 1.36 (d, J = 7.1 Hz, 3H); ^{13}C NMR (101 MHz, $CDCl_3$) δ 174.1, 140.1, 128.6, 128.2, 125.6, 82.8, 36.1, 31.6, 28.7, 17.5; HRMS (ESI) m/z calcd. for $C_{12}H_{14}NaO_2$ [M+Na]$^+$: 213.0886, found 213.0886; $[\alpha]_D^{20}$ = –29.3 (*c* 0.991, CH_2Cl_2); HPLC conditions: Chiralcel OD-OJ-H column, *n*-hexane/*i*-PrOH = 92:8, flow rate = 1.0 mL/min, uv-vis detection at λ = 210 nm, t_R = 39.402 min (major), 42.858 min (minor).

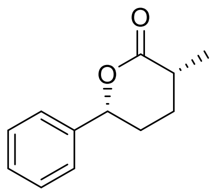

(3*R*,6*R*)-3-Methyl-6-phenyltetrahydro-2*H*-pyran-2-one (5a)

White solid (36.4 mg, 96%, >20:1 dr, >99% ee). Mp: 85–87 °C; ^{1}H NMR (400 MHz, $CDCl_3$) δ 7.39–7.29 (m, 5H), 5.35 (dd, J = 10.9 3.3 Hz, 1H), 2.78–2.71 (m, 1H), 2.23–2.12 (m, 2H), 1.99–1.90 (m, 1H), 1.63–1.55 (m, 1H), 1.29 (d, J = 6.8 Hz, 3H); ^{13}C NMR (101 MHz, $CDCl_3$) δ 175.7, 139.5, 128.6, 128.2, 125.8, 79.5, 33.5, 29.6, 25.6, 16.4; HRMS (ESI) m/z calcd. for $C_{12}H_{14}NaO_2$ $[M+Na]^+$: 213.0886, found 213.0885; $[\alpha]_D^{20}$ = –18.1 (*c* 0.821, CH_2Cl_2); HPLC conditions: Chiralcel OD-OJ-H column, *n*-hexane/*i*-PrOH = 92:8, flow rate = 1.0 mL/min, uv-vis detection at λ = 210 nm, t_R = 36.489 min (minor), 61.897 min (major).

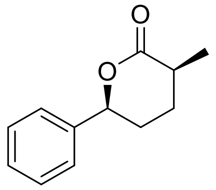

(3*S*,6*S*)-3-Methyl-6-phenyltetrahydro-2*H*-pyran-2-one (5a)

White solid (36.4 mg, 96%, >20:1 dr, >99% ee). Mp: 84–86 °C; ^{1}H NMR (400 MHz, $CDCl_3$) δ 7.39–7.30 (m, 5H), 5.35 (dd, J = 10.9, 3.3 Hz, 1H), 2.78–2.72 (m, 1H), 2.23–2.12 (m, 2H), 2.02–1.90 (m, 1H), 1.64–1.53 (m, 1H), 1.29 (d, J = 6.8 Hz, 3H); ^{13}C NMR (101 MHz, $CDCl_3$) δ 175.7, 139.5, 128.6, 128.2, 125.8, 79.5, 33.5, 29.6, 25.6, 16.4; HRMS (ESI) m/z calcd. for $C_{12}H_{14}NaO_2$ $[M+Na]^+$: 213.0886, found 213.0885; $[\alpha]_D^{20}$ = +19.5 (*c* 0.596, CH_2Cl_2); HPLC conditions: Chiralcel OD-OJ-H column, *n*-hexane/*i*-PrOH = 92:8, flow rate = 1.0 mL/min, uv-vis detection at λ = 210 nm, t_R = 36.017 min (major), 62.544 min (minor).

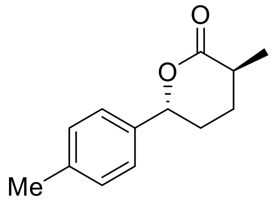

(3*S*,6*R*)-3-Methyl-6-(*p*-tolyl)tetrahydro-2*H*-pyran-2-one (5b)

White solid (40.4 mg, 99%, >20:1 dr, >99% ee). Mp: 121–122 °C; ^{1}H NMR (400 MHz, $CDCl_3$) δ 7.24–7.16 (m, 4H), 5.31 (dd, J = 10.9, 3.0 Hz, 1H), 2.61–2.51 (m, 1H), 2.35 (s, 3H), 2.20–2.08 (m, 2H), 1.98–1.88 (m, 1H), 1.77–1.67 (m, 1H), 1.36 (d, J = 7.0 Hz, 3H); ^{13}C NMR (101 MHz, $CDCl_3$) δ 174.2, 138.0, 137.2, 129.2, 125.6, 82.8, 36.1, 31.5,

28.7, 21.1, 17.5; HRMS (ESI) m/z calcd. for $C_{13}H_{16}NaO_2$ $[M+Na]^+$: 227.1043, found 227.1042; $[\alpha]_D^{20}$ = +26.8 (*c* 0.896, CH_2Cl_2); HPLC conditions: Chiralpak AS-H column, *n*-hexane/*i*-PrOH = 97:3, flow rate = 0.7 mL/min, uv-vis detection at λ = 210 nm, t_R = 41.125 min (major), 70.700 min (minor).

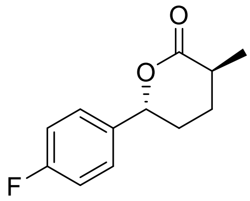

(*3S,6R*)-6-(4-Fluorophenyl)-3-methyltetrahydro-*2H*-pyran-2-one (5c)

White solid (41.2 mg, 99%, >20:1 dr, >99% ee). Mp: 135–137 °C; ^{1}H NMR (400 MHz, $CDCl_3$) *δ* 7.34–7.29 (m, 2H), 7.07–7.01 (m, 2H), 5.31 (dd, *J* = 11.1, 3.0 Hz, 1H), 2.61–2.51 (m, 1H), 2.19-2.08 (m, 2H), 1.95–1.84 (m, 1H), 1.78–1.67 (m, 1H), 1.35 (d, *J* = 7.0 Hz, 3H); ^{13}C NMR (101 MHz, $CDCl_3$) *δ* 173.9, 162.5 (d, *J* = 247.7 Hz), 136.0 (d, *J* = 3.2 Hz), 127.5 (d, *J* = 8.3 Hz), 115.6 (d, *J* = 21.9 Hz), 82.2, 36.1, 31.7, 28.7, 17.4; ^{19}F NMR (377 MHz, $CDCl_3$) *δ* –113.8; HRMS (ESI) m/z calcd. for $C_{12}H_{13}FNaO_2$ $[M+Na]^+$: 231.0792, found 231.0793; $[\alpha]_D^{20}$ = +26.4 (*c* 1.093, CH_2Cl_2); HPLC conditions: Chiralcel OJ-H column, *n*-hexane/*i*-PrOH = 95:5, flow rate = 0.8 mL/min, uv-vis detection at λ = 210 nm, t_R = 30.928 min (major), 34.407 min (minor).

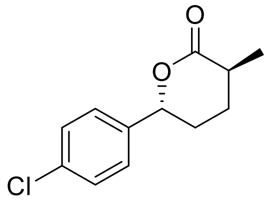

(*3S,6R*)-6-(4-Chlorophenyl)-3-methyltetrahydro-*2H*-pyran-2-one (5d)

White solid (44.3 mg, 99%, >20:1 dr, >99% ee). Mp: 143–145 °C; ^{1}H NMR (400 MHz, $CDCl_3$) *δ* 7.35–7.26 (m, 4H), 5.31 (dd, *J* = 11.0, 3.1 Hz, 1H), 2.61–2.53 (m, 1H), 2.20–2.08 (m, 2H), 1.93–1.82 (m, 1H), 1.78–1.68 (m, 1H), 1.36 (d, *J* = 7.0 Hz, 3H); ^{13}C NMR (101 MHz, $CDCl_3$) *δ* 173.8, 138.6, 134.0, 128.8, 127.0, 82.0, 36.1, 31.6, 28.6, 17.4; HRMS (ESI) m/z calcd. for $C_{12}H_{13}ClNaO_2$ $[M+Na]^+$: 247.0496, found 247.0496; $[\alpha]_D^{20}$ = +31.7 (*c* 0.761, CH_2Cl_2); HPLC conditions: Chiralpak AS-H column, *n*-hexane/*i*-PrOH = 97:3, flow rate = 0.7 mL/min, uv-vis detection at λ = 210 nm, t_R = 51.115 min (major), 73.926 min (minor).

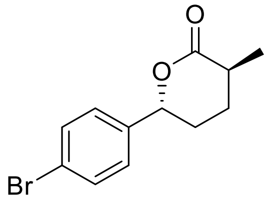

(*3S,6R*)-6-(4-Bromophenyl)-3-methyltetrahydro-*2H*-pyran-2-one (5e)

White solid (53.1 mg, 99%, >20:1 dr, >99% ee). Mp: 147–149 °C; ^{1}H NMR (400 MHz, $CDCl_3$) δ 7.51–7.48 (m, 2H), 7.24–7.20 (m, 2H), 5.30 (dd, J = 11.0, 3.1 Hz, 1H), 2.59–2.53 (m, 1H), 2.20–2.08 (m, 2H), 1.92–1.82 (m, 1H), 1.78–1.68 (m, 1H), 1.36 (d, J = 7.1 Hz, 3H); ^{13}C NMR (101 MHz, $CDCl_3$) δ 173.8, 139.2, 131.8, 127.2, 122.1, 82.1, 36.1, 31.6, 28.6, 17.4; HRMS (ESI) m/z calcd. for $C_{12}H_{13}BrNaO_2$ $[M+Na]^+$: 290.9991, found 290.9991; $[\alpha]_D^{20}$ = +26.3 (*c* 0.734, CH_2Cl_2); HPLC conditions: Chiralpak AS-H column, *n*-hexane/*i*-PrOH = 97:3, flow rate = 0.7 mL/min, uv-vis detection at λ = 210 nm, t_R = 54.604 min (major), 75.215 min (minor).

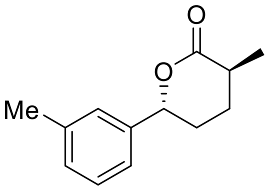

(*3S,6R*)-3-Methyl-6-(*m*-tolyl)tetrahydro-*2H*-pyran-2-one (5f)

White solid (40.4 mg, 99%, >20:1 dr, >99% ee). Mp: 115–116 °C; ^{1}H NMR (400 MHz, $CDCl_3$) δ 7.24–7.25 (m, 1H), 7.16–7.10 (m, 3H), 5.30 (dd, J = 11.1, 3.3 Hz, 1H), 2.60–2.51 (m, 1H), 2.35 (s, 3H), 2.20–2.07 (m, 2H), 1.96–1.86 (m, 1H), 1.76–1.66 (m, 1H), 1.35 (d, J = 7.1 Hz, 3H); ^{13}C NMR (101 MHz, $CDCl_3$) δ 174.1, 140.1, 138.3, 129.0, 128.5, 126.3, 122.7, 82.9, 36.1, 31.6, 28.7, 21.4, 17.5; HRMS (ESI) m/z calcd. for $C_{13}H_{16}NaO_2$ $[M+Na]^+$: 227.1043, found 227.1043; $[\alpha]_D^{20}$ = +30.3 (*c* 1.002, CH_2Cl_2); HPLC conditions: Chiralcel OD-OD-H column, *n*-hexane/*i*-PrOH = 97:3, flow rate = 0.7 mL/min, uv-vis detection at λ = 210 nm, t_R = 49.880 min (minor), 56.006 min (major).

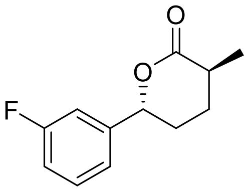

(*3S,6R*)-6-(3-Fluorophenyl)-3-methyltetrahydro-*2H*-pyran-2-one (5g)

White solid (41.2 mg, 99%, >20:1 dr, >99% ee). Mp: 126–128 °C; ^{1}H NMR (400 MHz, $CDCl_3$) δ 7.35–7.30 (m, 1H), 7.12–6.97 (m, 3H), 5.33 (dd, J = 11.0, 3.4 Hz, 1H), 2.61–2.51 (m, 1H), 2.22–2.08 (m, 2H), 1.94–1.83 (m, 1H), 1.78–1.66 (m, 1H), 1.36 (d, J = 7.0 Hz, 3H); ^{13}C NMR (101 MHz, $CDCl_3$) δ 173.7, 162.9 (d, J = 247.4 Hz), 142.7 (d, J = 7.1 Hz), 130.2 (d, J = 8.2 Hz), 121.2 (d, J = 3.0 Hz), 115.1 (d, J = 21.2 Hz), 112.7 (d, J = 22.6 Hz), 81.9 (d, J = 2.0 Hz), 36.1, 31.5, 28.5, 17.4; ^{19}F NMR (377 MHz, $CDCl_3$) δ –112.4; HRMS (ESI) m/z calcd. for $C_{12}H_{13}FNaO_2$ $[M+Na]^+$: 231.0792, found

231.0793; $[\alpha]_D^{20}$ = +32.1 (*c* 0.550, CH_2Cl_2); HPLC conditions: Chiralcel OJ-OZ-H column, *n*-hexane/*i*-PrOH = 97:3, flow rate = 0.7 mL/min, uv-vis detection at λ = 210 nm, t_R = 58.434 min (minor), 65.032 min (major).

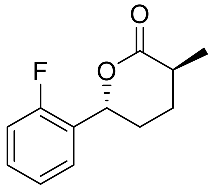

(*3S,6R*)-6-(2-Fluorophenyl)-3-methyltetrahydro-*2H*-pyran-2-one (5h)

White solid (41.2 mg, 99%, 18:1 dr, >99% ee). Mp: 95–97 °C; ^1H NMR (400 MHz, $CDCl_3$) *δ* 7.48–7.41 (m, 1H), 7.32–7.27 (m, 1H), 7.17 (t, *J* = 7.6 Hz, 1H), 7.05 (dd, *J* = 10.6, 8.2 Hz, 1H), 5.62 (dd, *J* = 11.2, 3.4 Hz, 1H), 2.65–2.56 (m, 1H), 2.27–2.21 (m, 1H), 2.15–2.08 (m, 1H), 1.96–1.83 (m, 1H), 1.82–1.71 (m, 1H), 1.38 (d, *J* = 7.0 Hz, 3H); ^{13}C NMR (101 MHz, $CDCl_3$) *δ* 173.9, 159.5 (d, *J* = 247.6 Hz), 129.7 (d, *J* = 8.3 Hz), 127.5 (d, *J* = 12.6 Hz), 127.2 (d, *J* = 3.8 Hz), 124.2 (d, *J* = 3.6 Hz), 115.5 (d, *J* = 21.4 Hz), 77.4 (d, *J* = 3.1 Hz), 36.3, 30.6, 28.7, 17.5; ^{19}F NMR (377 MHz, $CDCl_3$) *δ* –119.4; HRMS (ESI) m/z calcd. for $C_{12}H_{13}FNaO_2$ $[M+Na]^+$: 231.0792, found 231.0792; $[\alpha]_D^{20}$ = +32.1 (*c* 0.203, CH_2Cl_2); HPLC conditions: Chiralcel OD-H column, *n*-hexane/*i*-PrOH = 97:3, flow rate = 0.7 mL/min, uv-vis detection at λ = 210 nm, t_R = 15.639 min (minor), 16.335 min (major).

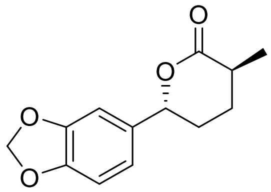

(*3S,6R*)-6-(Benzo[*d*][1,3]dioxol-5-yl)-3-methyltetrahydro-*2H*-pyran-2-one (5i)

White solid (25.2 mg, 54%, 97% ee). Mp: 93–94 °C; ^1H NMR (400 MHz, $CDCl_3$) *δ* 6.84 (s, 1H), 6.81–6.76 (m, 2H), 5.96 (s, 2H), 5.24 (dd, *J* = 11.1, 3.1 Hz, 1H), 2.58–2.52 (m, 1H), 2.16–2.07 (m, 2H), 1.96–1.85 (m, 1H), 1.76–1.65 (m, 1H), 1.35 (d, *J* = 7.0 Hz, 3H); ^{13}C NMR (101 MHz, $CDCl_3$) *δ* 174.0, 147.9, 147.6, 134.0, 119.4, 108.2, 106.4, 101.2, 82.8, 36.0, 31.6, 28.7, 17.4; HRMS (ESI) m/z calcd. for $C_{13}H_{14}NaO_4$ $[M+Na]^+$: 257.0784, found 257.0785; $[\alpha]_D^{20}$ = +22.0 (*c* 0.563, CH_2Cl_2); HPLC conditions: Chiralcel OJ-H column, *n*-hexane/*i*-PrOH = 89:11, flow rate = 1.0 mL/min, uv-vis detection at λ = 210 nm, t_R = 50.444 min (major), 65.631 min (minor).

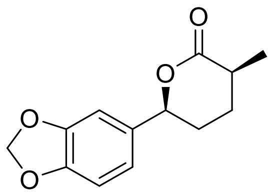

(*3S,6S*)-6-(Benzo[*d*][1,3]dioxol-5-yl)-3-methyltetrahydro-*2H*-pyran-2-one (5i)

White solid (21.0 mg, 45%, >99% ee). Mp: 89–91 °C; ^{1}H NMR (400 MHz, $CDCl_3$) δ 6.85 (s, 1H), 6.81–6.76 (m, 2H), 5.96 (s, 2H), 5.25 (dd, J = 11.1, 3.6 Hz, 1H), 2.75–2.69 (m, 1H), 2.20–2.07 (m, 2H), 1.99–1.88 (m, 1H), 1.63–1.55 (m, 1H), 1.28 (d, J = 6.8 Hz, 3H); ^{13}C NMR (101 MHz, $CDCl_3$) δ 175.7, 148.0, 147.6, 133.4, 119.6, 108.2, 106.6, 101.2, 79.3, 33.3, 29.6, 25.6, 16.3; HRMS (ESI) m/z calcd. for $C_{13}H_{14}NaO_4$ $[M+Na]^+$: 257.0784, found 257.0784; $[\alpha]_D^{20}$ = +12.7 (*c* 0.626, CH_2Cl_2); HPLC conditions: Chiralcel OJ-H column, *n*-hexane/*i*-PrOH = 89:11, flow rate = 1.0 mL/min, uv-vis detection at λ = 210 nm, t_R = 36.060 min (minor), 39.008 min (major).

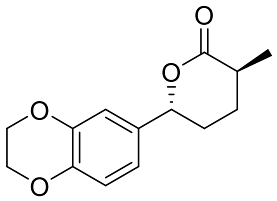

(*3S,6R*)-6-(2,3-Dihydrobenzo[*b*][1,4]dioxin-6-yl)-3-methyltetrahydro-*2H*-pyran-2-one (5j)

White solid (28.8 mg, 58%, >99% ee). Mp: 154–156 °C; ^{1}H NMR (400 MHz, $CDCl_3$) δ 6.86–6.80 (m, 3H), 5.23 (dd, J = 11.0, 3.1 Hz, 1H), 4.25 (s, 4H), 2.59–2.49 (m, 1H), 2.17–2.07 (m, 2H), 1.97–1.86 (m, 1H), 1.75–1.64 (m, 1H), 1.35 (d, J = 7.1 Hz, 3H); ^{13}C NMR (101 MHz, $CDCl_3$) δ 174.1, 143.5, 133.4, 118.9, 117.3, 115.0, 82.5, 64.38, 64.35, 36.0, 31.3, 28.6, 17.4; HRMS (ESI) m/z calcd. for $C_{14}H_{16}NaO_4$ $[M+Na]^+$: 271.0941, found 271.0941; $[\alpha]_D^{20}$ = +24.4 (*c* 0.149, CH_2Cl_2); HPLC conditions: Chiralcel OJ-H column, *n*-hexane/*i*-PrOH = 89:11, flow rate = 1.0 mL/min, uv-vis detection at λ = 210 nm, t_R = 46.897 min (minor), 66.152 min (major).

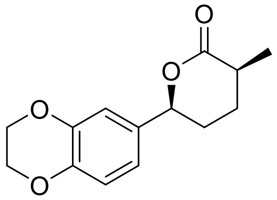

(*3S,6S*)-6-(2,3-Dihydrobenzo[*b*][1,4]dioxin-6-yl)-3-methyltetrahydro-*2H*-pyran-2-one (5j)

White solid (20.8 mg, 42%, 93% ee). Mp: 127–130 °C; ^{1}H NMR (400 MHz, $CDCl_3$) δ 6.87–6.80 (m, 3H), 5.24 (dd, J = 10.9, 3.5 Hz, 1H), 4.25 (s, 4H), 2.75–2.69 (m, 1H),

2.21–2.07 (m, 2H), 2.00–1.89 (m, 1H), 1.63–1.53 (m, 1H), 1.28 (d, *J* = 6.8 Hz, 3H). ^{13}C NMR (101 MHz, $CDCl_3$) *δ* 175.7, 143.5, 132.7, 119.1, 117.3, 115.1, 79.1, 64.38, 64.35, 33.4, 29.4, 25.6, 16.3; HRMS (ESI) m/z calcd. for $C_{14}H_{16}NaO_4$ $[M+Na]^+$: 271.0941, found 271.0941; $[\alpha]_D^{20}$ = +18.2 (*c* 0.305, CH_2Cl_2); HPLC conditions: Chiralcel OJ-H column, *n*-hexane/*i*-PrOH = 89:11, flow rate = 1.0 mL/min, uv-vis detection at λ = 210 nm, t_R = 54.405 min (major), 71.899 min (minor).

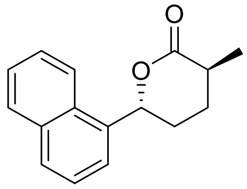

(3*S*,6*R*)-3-Methyl-6-(naphthalen-1-yl)tetrahydro-2*H*-pyran-2-one (5k)

White solid (47.5 mg, 99%, 15:1 dr, >99% ee). Mp: 131–133 °C; ^1H NMR (400 MHz, $CDCl_3$) *δ* 7.94–7.88 (m, 2H), 7.82 (d, *J* = 8.2 Hz, 1H), 7.63–7.61 (m, 1H), 7.55–7.47 (m, 3H), 6.11 (dd, *J* = 11.0, 3.3 Hz, 1H), 2.71–2.65 (m, 1H), 2.42–2.37 (m, 1H), 2.21–2.14 (m, 1H), 2.09–1.99 (m, 1H), 1.91–1.81 (m, 1H), 1.43 (d, *J* = 7.0 Hz, 3H); ^{13}C NMR (101 MHz, $CDCl_3$) *δ* 174.3, 135.5, 133.8, 129.9, 129.1, 128.8, 126.5, 125.7, 125.4, 123.2, 122.5, 80.1, 36.4, 30.9, 28.9, 17.6; HRMS (ESI) m/z calcd. for $C_{16}H_{16}NaO_2$ $[M+Na]^+$: 263.1043, found 263.1042; $[\alpha]_D^{20}$ = +31.9 (*c* 0.639, CH_2Cl_2); HPLC conditions: Chiralcel OJ-H column, *n*-hexane/*i*-PrOH = 95:5, flow rate = 1.0 mL/min, uv-vis detection at λ = 210 nm, t_R = 31.065 min (major), 47.398 min (minor).

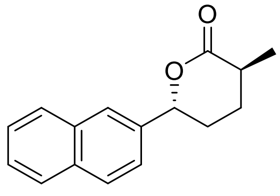

(3*S*,6*R*)-3-Methyl-6-(naphthalen-2-yl)tetrahydro-2*H*-pyran-2-one (5l)[7b]

White solid (47.5 mg, 99%, >20:1 dr, >99% ee). Mp: 136–138 °C; ^1H NMR (400 MHz, $CDCl_3$) *δ* 7.86–7.82 (m, 4H), 7.52–7.42 (m, 3H), 5.51 (dd, *J* = 11.1, 3.4 Hz, 1H), 2.66–2.56 (m, 1H), 2.29–2.23 (m, 1H), 2.17–2.10 (m, 1H), 2.04–1.94 (m, 1H), 1.82–1.72 (m, 1H), 1.39 (d, *J* = 7.1 Hz, 3H); ^{13}C NMR (101 MHz, $CDCl_3$) *δ* 174.1, 137.5, 133.2, 133.1, 128.5, 128.1, 127.7, 126.4, 126.3, 124.5, 123.4, 82.9, 36.1, 31.6, 28.7, 17.5; HRMS (ESI) m/z calcd. for $C_{16}H_{16}NaO_2$ $[M+Na]^+$: 263.1043, found 263.1042; $[\alpha]_D^{20}$ = +30.4 (*c* 0.683, CH_2Cl_2); HPLC conditions: Chiralpak IE-H column, *n*-hexane/*i*-PrOH = 95:5, flow rate = 1.0 mL/min, uv-vis detection at λ = 210 nm, t_R = 49.468 min (major), 59.271 min (minor).

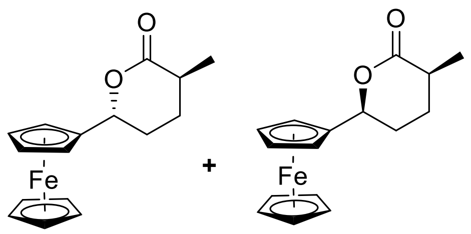

(3*S*,6*R*)-3-Methyl-6-(Ferrocene-1-yl)tetrahydro-2*H*-pyran-2-one (5m)

(3*S*,6*S*)-3-Methyl-6-(Ferrocene-1-yl)tetrahydro-2*H*-pyran-2-one (5m)

Brown solid, (59.0 mg, 99%, 2:1 dr, >99%/99% ee). Mp: 90–91 °C; ^{1}H NMR (400 MHz, $CDCl_3$) δ 5.17 (dd, *J* = 11.0, 3.0 Hz, 1H), 4.26–4.21 (m, 2H), 4.17 (d, *J* = 4.3 Hz, 7H), 2.52–2.44 (m, 1H), 2.28–2.24 (m, 1H), 2.13– 2.06 (m, 1H), 1.99–1.89 (m, 1H), 1.72–1.61 (m, 1H), 1.33 (d, *J* = 6.9 Hz, 3H); ^{13}C NMR (101 MHz, $CDCl_3$) δ 175.7, 174.1, 87.3, 86.7, 79.8, 68.88, 68.85, 68.6, 68.34, 68.29, 68.2, 67.4, 67.2, 66.1, 65.9, 36.0, 33.4, 29.6, 28.6, 28.0, 25.7, 17.5, 16.4; HRMS (ESI) m/z calcd. for $C_{16}H_{18}FeNaO_2$ $[M+Na]^+$: 321.0548, found 321.0551; $[\alpha]_D^{20}$ = +11.5 (*c* 0.719, CH_2Cl_2); HPLC conditions: Chiralcel OZ-H column, *n*-hexane/*i*-PrOH = 90:10, flow rate = 1.0 mL/min, uv-vis detection at λ = 210 nm, **(*S*,*R*)-5m**: t_R = 16.919 min (major), 19.951 min (minor); **(*S*,*S*)-5m**: t_R = 21.448 min (major), 25.360 min (minor).

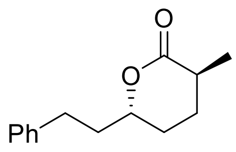

(3*S*,6*R*)-3-Methyl-6-phenethyltetrahydro-2*H*-pyran-2-one (5n)

White solid (24.4 mg, 56%, >99% ee). Mp: 60–61 °C; ^{1}H NMR (400 MHz, $CDCl_3$) δ 7.31–7.27 (m, 2H), 7.21–7.17 (m, 3H), 4.31–4.25 (m, 1H), 2.89–2.82 (m, 1H), 2.78–2.70 (m, 1H), 2.48–2.42 (m, 1H), 2.06–1.97 (m, 2H), 1.94–1.84 (m, 2H), 1.68–1.49 (m, 2H), 1.30 (d, *J* = 7.0 Hz, 3H); ^{13}C NMR (101 MHz, $CDCl_3$) δ 174.4, 141.2, 128.5, 126.1, 80.7, 38.0, 36.2, 31.1, 29.3, 28.5, 17.4; HRMS (ESI) m/z calcd. for $C_{14}H_{18}NaO_2$ $[M+Na]^+$: 241.1199, found 241.1198; $[\alpha]_D^{20}$ = –51.6 (*c* 0.347, CH_2Cl_2); HPLC conditions: Chiralcel OJ-H column, *n*-hexane/*i*-PrOH = 90:10, flow rate = 1.0 mL/min, uv-vis detection at λ = 210 nm, t_R = 13.543 min (minor), 22.804 min (major).

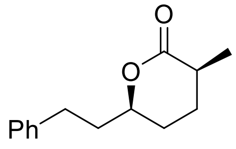

(3*S*,6*S*)-3-Methyl-6-phenethyltetrahydro-2*H*-pyran-2-one (5n)

White solid (18.7 mg, 43%, >99% ee). Mp: 60–61 °C; ^{1}H NMR (400 MHz, $CDCl_3$) δ 7.31–7.27 (m, 2H), 7.22–7.18 (m, 3H), 4.30–4.23 (m, 1H), 2.90–2.83 (m, 1H), 2.77–

2.70 (m, 1H), 2.62–2.52 (m, 1H), 2.12–1.97 (m, 2H), 1.94–1.80 (m, 2H), 1.72–1.61 (m, 1H), 1.57–1.47 (m, 1H), 1.22 (d, J = 6.7 Hz, 3H); ^{13}C NMR (101 MHz, $CDCl_3$) δ 176.4, 141.2, 128.52, 128.49, 126.1, 77.0, 37.0, 33.2, 31.3, 26.8, 25.6, 16.2; HRMS (ESI) m/z calcd. for $C_{14}H_{18}NaO_2$ $[M+Na]^+$: 241.1199, found 241.1199; $[\alpha]_D^{20}$ = +99.1 (*c* 0.138, CH_2Cl_2); HPLC conditions: Chiralcel OJ-H column, *n*-hexane/*i*-PrOH = 90:10, flow rate = 1.0 mL/min, uv-vis detection at λ = 210 nm, t_R = 15.791 min (major), 17.198 min (minor).

4. Scale-Up Reaction, Transformations and Applications

4.1 Scale-up synthesis of 3a

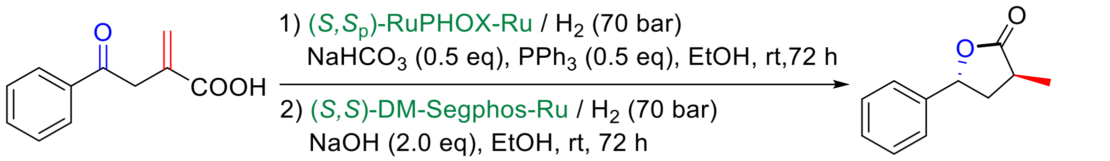

1a (10 mmol, 1.90 g)

(*S*,*R*)-3a, 1.72 g, 98%
> 20:1 dr, >99% ee

α-Methylene-*γ*-keto carboxylic acid **1a** (10 mmol, 1.90 g), $NaHCO_3$ (5.0 mmol, 0.42 g), PPh_3 (5.0 mmol, 1.31 g) and (*S*,*S*$_p$)-RuPHOX-Ru (0.0091 mol%, 1.6 mg) were added into a 100 mL beaker. The beaker was placed in an autoclave and EtOH (40 mL) was transferred into it by a syringe under nitrogen. The autoclave was filled with hydrogen for five times and charged with hydrogen to 70 atm. The reaction mixture was stirred at room temperature for 72 hours. The hydrogen gas was released slowly to 1 bar and NaOH (20 mmol, 0.80 g) with EtOH (20 mL) was added into the autoclave by a syringe, followed by (*S*,*S*)-DM-Segphos-Ru (0.0091 mol%, 1.1 mg) with EtOH (20 mL) by a syringe after the first step reaction was completed. The autoclave was again replaced with hydrogen for five times and charged with hydrogen to 70 atm. The reaction mixture was stirred at room temperature for another 72 hours. Hydrogen gas was released slowly and the reaction solvent was removed under reduced pressure. The residue was dissolved with DCM (10 mL) and acidified with HCl (3M) to pH = 1. DCM was separated and the aqueous phase was extracted with DCM (10 mL × 3). The combined organic layer was dried over anhydrous Na_2SO_4 and evoparated under reduced pressure to afford the crude product. The drs of the product were determined by ^{1}H NMR analysis of the crude product which was purified by column chromatography (PE/EtOAc = 10/1) to afford the pure product **3a** (1.72 g, 98%, >20:1 dr, >99% ee).

The scale-up synthesis of **3a** was manipulated as follows:

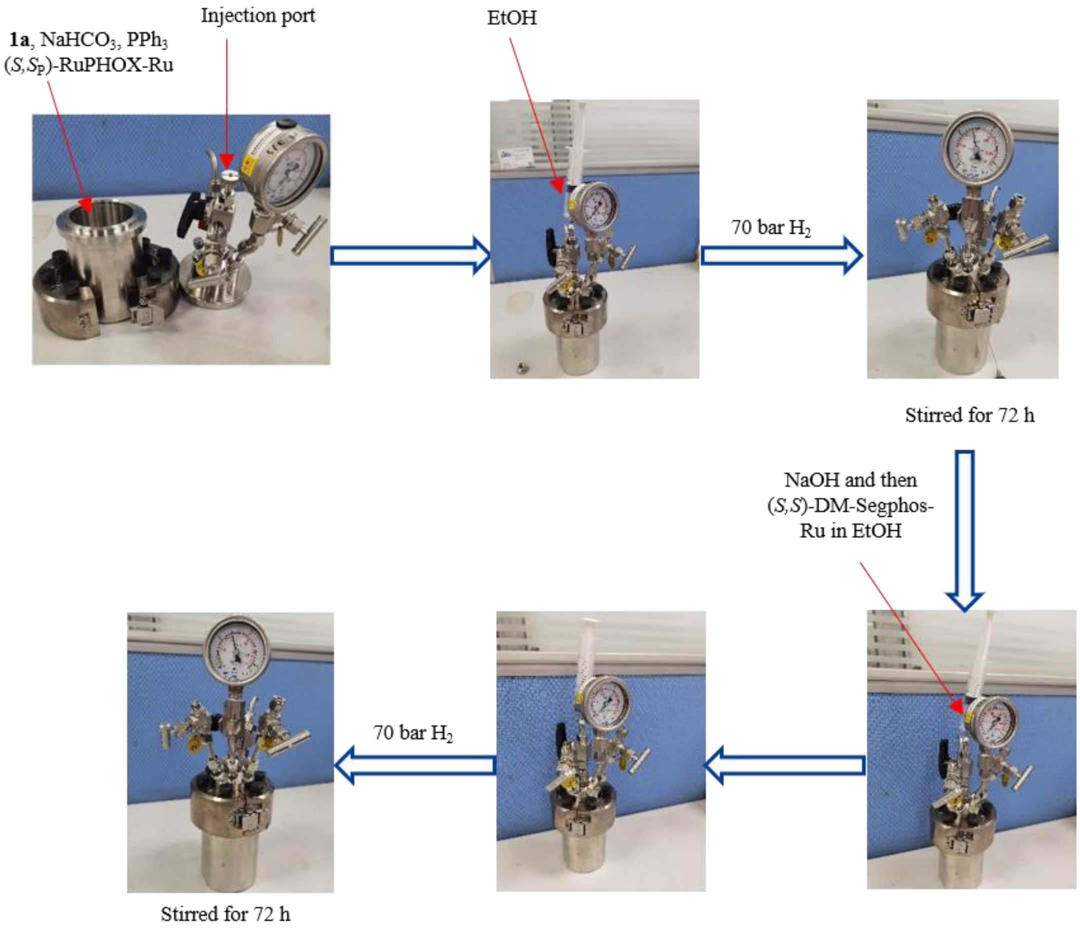

Figure S3. Practical procedure for scale-up synthesis of **3a**

4.2 Transformations of 3a

4.2.1 Synthesis of (*2S,4R*)-4-hydroxy-2-methyl-1-morpholino-4-phenylbutan-1-one (6)[8]

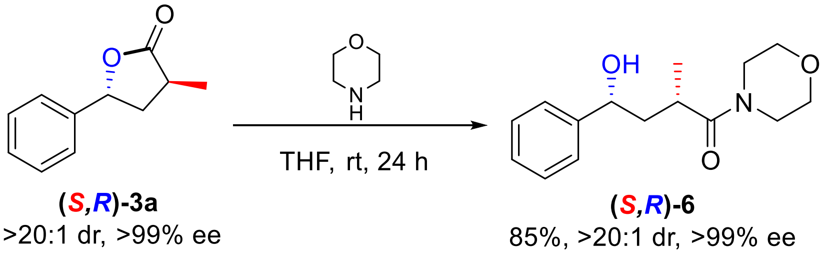

(***3S,5R***)-**3a** (0.2 mmol, 35.2 mg) and THF (2 mL) were added into a 10 mL vial. Then morpholine (0.4 mmol, 34.8 mg) were added and the mixture was stirred at room temperature for 24 hours. After reaction was finished by TLC analysis, the reaction solvent was removed under reduced pressure to afford the crude product. The dr of the product was determined by ^{1}H NMR analysis of the crude product which was purified by column chromatography (PE/EtOAc = 2/1) to give the pure product **6** as a colourless oil (44.7 mg, 85%, >20:1 dr, >99% ee). ^{1}H NMR (400 MHz, $CDCl_3$) δ 7.33 (d, J = 4.4

Hz, 4H), 7.25–7.23 (m, 1H), 4.76 (dd, J = 8.7, 3.4 Hz, 1H), 3.73–3.56 (m, 6H), 3.48–3.37 (m, 2H), 3.12 (s, 1H), 2.92–2.84 (m, 1H), 2.25–2.18 (m, 1H), 1.85–1.78 (m, 1H), 1.14 (d, J = 7.0 Hz, 3H); ^{13}C NMR (101 MHz, $CDCl_3$) δ 175.5, 144.8, 128.4, 127.4, 125.6, 71.8, 67.0, 66.8, 46.1, 42.8, 42.3, 31.9, 18.1; HRMS (ESI) m/z calcd. for $C_{15}H_{21}NNaO_3$ $[M+Na]^+$: 286.1414, found 286.1413; $[\alpha]_D^{20}$ = +50.7 (c 0.214, CH_2Cl_2); HPLC conditions: Chiralpak AD-H column, n-hexane/i-PrOH = 90:10, flow rate = 1.0 mL/min, uv-vis detection at λ = 210 nm, t_R = 10.694 min (minor), 17.101 min (major).

4.2.2 Synthesis of (*3S,5S*)-3-methyl-5-phenyldihydrothiophen-2(*3H*)-one (7)[9]

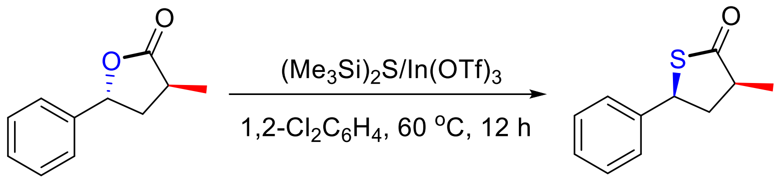

(*S*,*R*)-3a
>20:1 dr, >99% ee

(*S*,*S*)-7
70%, >20:1 dr, 99% ee

(*S*,*R*)-3a (0.20 mmol, 35.2 mg) was added into a 15 mL screw-capped tube. The tube was moved into a nitrogen-filled glovebox, and $In(OTf)_3$ (0.002 mmol, 1.1 mg), 1,2-dichlorobenzene (2 mL) and hexamethyldisilathiane (0.24 mmol, 42.8 mg) were added in sequence. The tube was sealed and removed from the glovebox. The mixture was stirred at 60 ℃ with an oil bath for 12 hours. The reaction was cooled to room temperature and the reaction solvent was removed under reduced pressure, The dr of the product was determined by ^{1}H NMR analysis of the crude product which was purified by flash column chromatography (PE/EtOAc = 10/1) to afford the pure product **7** as a white solid (26.9 mg, 70%, >20:1 dr, 99% ee). Mp: 81–83 ℃; ^{1}H NMR (400 MHz, $CDCl_3$) δ 7.43–7.29 (m, 5H), 4.85 (dd, J = 11.8, 4.8 Hz, 1H), 2.83–2.68 (m, 2H), 2.01–1.92 (m, 1H), 1.26 (d, J = 6.6 Hz, 3H); ^{13}C NMR (101 MHz, $CDCl_3$) δ 209.2, 139.2, 128.9, 128.2, 127.6, 51.0, 49.4, 43.8, 14.3; HRMS (ESI) m/z calcd. for $C_{11}H_{12}NaOS$ $[M+Na]^+$: 215.0501, found 215.0501; $[\alpha]_D^{20}$ = +45.1 (c 0.284, CH_2Cl_2); HPLC conditions: Chiralpak IC-H column, n-hexane/i-PrOH = 97:3, flow rate = 0.8 mL/min, uv-vis detection at λ = 210 nm, t_R = 15.570 min (minor), 19.169 min (major).

4.2.3 Synthesis of (*3S,5R*)-3-methyl-5-phenyldihydrofuran-2(*3H*)-thione (8)[10]

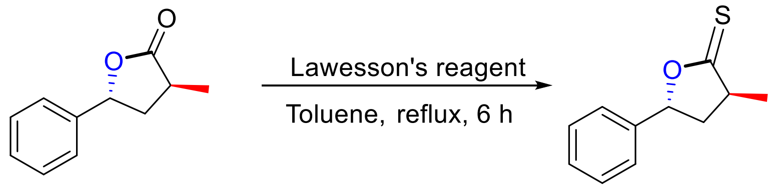

(*S*,*R*)-3a
>20:1 dr, >99% ee

(*S*,*R*)-8
85%, >20:1 dr, 98% ee

(*S*,*R*)-3a (0.20 mmol, 35.2 mg) and 2 mL dry toluene were added into a 10 mL round-bottom flask, and then Lawesson's reagent (0.12 mmol, 48.5 mg) was added slowly. The flask was then connected to a condenser and the reaction mixture stirred with refluxing for 6 hours until the full consumption of starting material was confirmed by TLC analysis. Then the reaction was cooled to room temperature and the reaction solvent was removed under reduced pressure. The dr of the product was determined by ^{1}H NMR analysis of the crude product which was purified by flash column chromatography (PE/EtOAc = 4/1) to to afford the pure product **8** as a colourless oil (32.6 mg, 85%, >20:1 dr, 98% ee). ^{1}H NMR (400 MHz, $CDCl_3$) δ 7.43–7.28 (m, 3H), 7.25–7.19 (m, 2H), 5.65 (dd, J = 11.1, 5.6 Hz, 1H), 3.10–3.00 (m, 1H), 2.89–2.83 (m, 1H), 1.95–1.86 (m, 1H), 1.47 (d, J = 6.8 Hz, 3H); ^{13}C NMR (101 MHz, $CDCl_3$) δ 201.2, 137.8, 128.8, 127.7, 126.1, 87.4, 49.8, 40.6, 18.4; HRMS (ESI) m/z calcd. for $C_{11}H_{12}NaOS$ $[M+Na]^+$: 215.0501, found 215.0504; $[\alpha]_D^{20}$ = –56.7 (c 0.353, CH_2Cl_2); HPLC conditions: Chiralpak IC-H column, n-hexane/i-PrOH = 97:3, flow rate = 0.6 mL/min, uv-vis detection at λ = 210 nm, t_R = 17.542 min (major), 21.337 min (minor).

4.2.4 Synthesis of Calyxolane A and B analogue (10)[11-12]

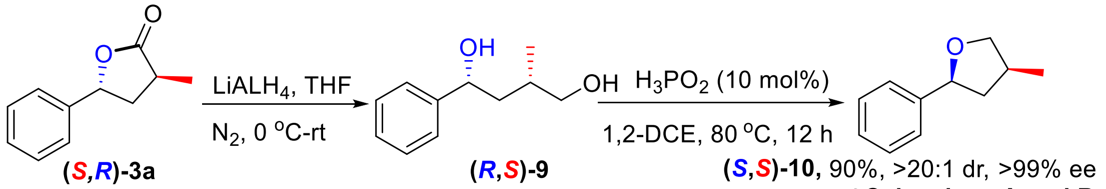

(*S*,*R*)-3a >20:1 dr, >99% ee — **(*R*,*S*)-9** 99%, >20:1 dr, >99% ee — **(*S*,*S*)-10**, 90%, >20:1 dr, >99% ee Analogue of **Calyxolane A and B**

Step 1: Synthesis of (*1R*,*3S*)-3-methyl-1-phenylbutane-1,4-diol (9)

To a solution of **(*S*,*R*)-3a** (0.20 mmol, 35.2 mg) in dry THF (2 mL) was added $LiAlH_4$ (0.8 mmol, 2 M in THF) dropwise under a nitrogen atmosphere at 0 °C. The reaction was stirred at room temperature and analyzed by TLC. After the reaction was over, it was quenched with water (1.0 mL) and extracted with ethyl acetate (2 mL × 3). The combined organic layer was dried over anhydrous Na_2SO_4 and removed under reduced pressure to afford the crude product. The dr of the product was determined by ^{1}H NMR analysis of the crude product which was purified by column chromatography (PE/EtOAc = 2/1) to afford the pure product **9** as a white solid (35.6 mg, 99%, >20:1 dr, >99% ee). Mp: 57–59 °C; ^{1}H NMR (400 MHz, $CDCl_3$) δ 7.39–7.32 (m, 4H), 7.27–7.24 (m, 1H), 4.82 (dd, J = 7.5, 4.3 Hz, 1H), 3.56–3.42 (m, 2H), 3.31 (s, 2H), 1.85–1.73 (m, 3H), 0.92 (d, J = 5.8 Hz, 3H); ^{13}C NMR (101 MHz, $CDCl_3$) δ 144.8, 128.4, 127.3, 125.8, 71.7, 67.8, 43.6, 32.2, 17.2; HRMS (ESI) m/z calcd. for $C_{11}H_{16}NaO_2$ $[M+Na]^+$: 203.1043, found 203.1043; $[\alpha]_D^{20}$ = +30.1 (c 0.154, CH_2Cl_2); HPLC conditions: Chiralcel OC-H column, n-hexane/i-PrOH = 97:3, flow rate = 0.8 mL/min, uv-vis detection at λ

= 210 nm, t_R = 56.211 min (minor), 61.398 min (major).

Step 2: Synthesis of (*2S,4S*)-4-methyl-2-phenyltetrahydrofuran (10)

To a stirred solution of ***(R,S)*-9** (0.15 mmol, 27.0 mg) in 1,2-DCE (1.0 mL) in a 15 mL screw-capped tube was added 50% (v/v) aqueous solution of H_3PO_2 (0.015 mmol, 2.0 μL) under a nitrogen atmosphere. The tube was sealed and put into 80 °C oil bath. The reaction mixture was stirred at 80 °C for 12 hours. The reaction solvent was removed under reduced pressure to afford the crude product. The dr of product was determined by ^1H NMR analysis of the crude product which was purified by column chromatography (PE/EtOAc = 10/1) to afford the pure product **10** as a colourless oil (21.9 mg, 90%, >20:1 dr, >99% ee). ^1H NMR (400 MHz, $CDCl_3$) δ 7.36–7.30 (m, 4H), 7.25–7.22 (m, 1H), 4.91 (dd, J = 10.0, 5.8 Hz, 1H), 4.08 (t, J = 7.7 Hz, 1H), 3.57 (t, J = 8.0 Hz, 1H), 2.54–2.42 (m, 2H), 1.49–1.40 (m, 1H), 1.09 (d, J = 6.2 Hz, 3H); ^{13}C NMR (101 MHz, $CDCl_3$) δ 143.4, 128.3, 127.2, 125.6, 81.6, 75.5, 43.9, 35.0, 17.4; HRMS (ESI) m/z calcd. for $C_{11}H_{14}NaO$ $[M+Na]^+$: 185.0937, found 185.0939; $[\alpha]_D^{20}$ = –45.1 (*c* 0.078, CH_2Cl_2); HPLC conditions: Chiralcel OD-H column, *n*-hexane/*i*-PrOH = 98:2, flow rate = 0.4 mL/min, uv-vis detection at λ = 210 nm, t_R = 25.903 min (minor), 40.279 min (major).

4.2.5 Synthesis of amino alcohol (13)[13]

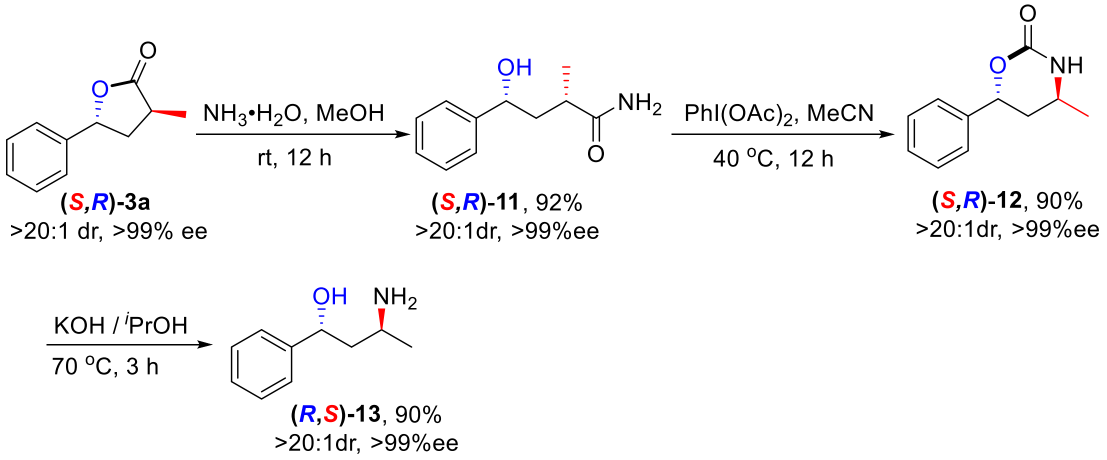

Step 1: Synthesis of (*2S,4R*)-4-hydroxy-2-methyl-4-phenylbutanamide (11)

To a solution of ***(S,R)*-3a** (0.40 mmol, 70.5 mg) in methanol (4 mL) was added ammonium hydroxide (1.0 mL) dropwise. The reaction was stirred at room temperature for 12 hours. Then the reaction solvent was removed under reduced pressure to afford the crude product. The dr of the product was determined by ^1H NMR analysis of the crude product which was purified by column chromatography (PE/EtOAc = 1/1) to afford the pure product **11** as a white solid (71.0 mg, 92%, >20:1 dr, >99% ee). Mp: 95–97 °C; ^1H NMR (400 MHz, $CDCl_3$) δ 7.26–7.16 (m, 5H), 5.88 (d, J = 25.1 Hz, 2H),

4.69 (dd, J = 9.8, 3.4 Hz, 1H), 3.06 (s, 1H), 2.61–2.52 (m, 1H), 1.96–1.87 (m, 1H), 1.76–1.69 (m, 1H), 1.10 (d, J = 7.0 Hz, 3H); ^{13}C NMR (101 MHz, $CDCl_3$) δ 179.7, 144.6, 128.5, 127.5, 125.6, 71.7, 43.3, 37.1, 18.2; HRMS (ESI) m/z calcd. for $C_{11}H_{15}NNaO_2$ $[M+Na]^+$: 216.0995, found 216.0996; $[\alpha]_D^{20}$ = +49.6 (c 0.450, CH_2Cl_2); HPLC conditions: Chiralpak AS-H column, n-hexane/i-PrOH = 85:15, flow rate = 1.0 mL/min, uv-vis detection at λ = 210 nm, t_R = 24.071 min (minor), 40.566 min (major).

Step 2: Synthesis of (*4S,6R*)-4-methyl-6-phenyl-1,3-oxazinan-2-one (12)

To a solution of **(*S*,*R*)-11** (0.25 mmol, 48.3 mg) in acetonitrile (3 mL) in a 15 mL screw-capped tube were added $PhI(OAc)_2$ (0.25 mmol, 80.5 mg). The reaction was stirred at 40 °C oil bath for 12 hours. The reaction solvent was removed under reduced pressure to afford the crude product. The dr of the product was determined by ^{1}H NMR analysis of the crude product which was purified by column chromatography (PE/EtOAc = 1/1) to afford the pure product **12** as a white solid (42.9 mg, 90%, >20:1 dr, >99% ee). Mp: 122–124 °C; ^{1}H NMR (400 MHz, $CDCl_3$) δ 7.39–7.28 (m, 5H), 6.84 (s, 1H), 5.46 (dd, J = 7.2, 3.7 Hz, 1H), 3.56–3.48 (m, 1H), 2.20–2.13 (m, 1H), 1.98–1.92 (m, 1H), 1.27 (d, J = 6.5 Hz, 3H); ^{13}C NMR (101 MHz, $CDCl_3$) δ 154.8, 139.3, 128.7, 128.1, 125.3, 75.8, 43.5, 35.1, 22.2; HRMS (ESI) m/z calcd. for $C_{11}H_{13}NNaO_2$ $[M+Na]^+$: 214.0838, found 214.0839; $[\alpha]_D^{20}$ = +10.9 (c 0.173, CH_2Cl_2); HPLC conditions: Chiralpak AS-H column, n-hexane/i-PrOH = 75:25, flow rate = 1.0 mL/min, uv-vis detection at λ = 210 nm, t_R = 45.102 min (major), 76.683 min (minor).

Step 3: Synthesis of (*1R,3S*)-3-amino-1-phenylbutan-1-ol (13)

To a solution of **(*S*,*R*)-12** (0.15 mmol, 28.7 mg) in ^{i}PrOH (2 mL) and H_2O (0.1 mL) in a 15 mL screw-capped tube were added KOH (0.3 mmol, 16.8 mg). The reaction was stirred at 70 °C oil bath for 3 hours. The reaction solvent was removed under reduced pressure to afford the crude product. The dr of the product was determined by ^{1}H NMR analysis of the crude product which was purified by column chromatography (PE/EtOAc = 1/1) to afford the pure product **13** as a colourless oil (22.3 mg, 90%, >20:1 dr, >99% ee). ^{1}H NMR (400 MHz, $CDCl_3$) δ 7.37–7.27 (m, 4H), 7.24–7.19 (m, 1H), 5.07 (dd, J = 6.8, 4.9 Hz, 1H), 4.17 (s, 3H), 3.42–3.34 (m, 1H), 1.93–1.89 (m, 2H), 1.26 (d, J = 6.6 Hz, 3H); ^{13}C NMR (101 MHz, $CDCl_3$) δ 144.4, 128.4, 127.1, 125.6, 70.8, 45.1, 43.5, 21.8; HRMS (ESI) m/z calcd. for $C_{10}H_{15}NNaO$ $[M+Na]^+$: 188.1046, found 188.1047; $[\alpha]_D^{20}$ = +20.9 (c 0.094, CH_2Cl_2); HPLC conditions: Chiralcel OZ-H column after amidation with acetyl chloride in the presence of pyridine, n-hexane/i-PrOH = 90:10, flow rate = 1.0 mL/min, uv-vis detection at λ = 210 nm, t_R = 14.297 min (minor), 18.525 min (major).

4.2.6 Synthesis of phenyl hexenoate (16)[14] and carboxylic acid (17)[14]

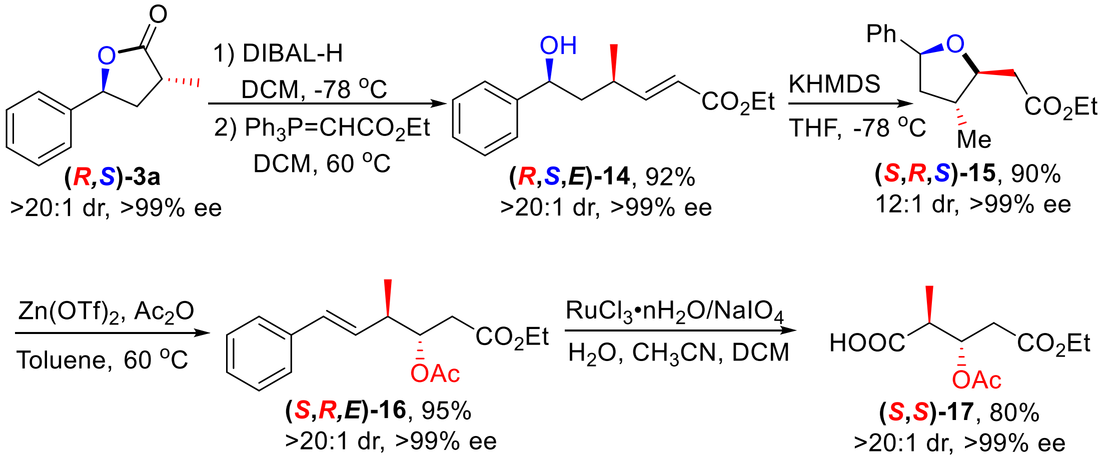

Step 1: Synthesis of ethyl (*4R,6S,E*)-6-hydroxy-4-methyl-6-phenylhex-2-enoate (14)

To a solution of ***(R,S)*-3** (1.5 mmol, 264.3 mg) in DCM (10 mL) was added DIBAL-H (1.9 mmol, 1.9 mL) dropwise at –78 °C. The reaction was stirred for 1 h at this temperature. The reaction was diluted with DCM (10 mL) and quenched with MeOH at –78 °C. The organic phase was washed with aqueous Na-K tartrate (10 mL × 3) to remove the aluminum salts. The combined organic layers were dried with anhydrous Na_2SO_4 and removed under reduced pressure to acquire the crude product, which was directly used for the next step without further purification. The crude product was dissolved with DCM (15 mL) in a 50 mL round-bottom flask and the Wittig reagent Ph_3P=$CHCO_2Et$ (661.9 mg, 1.9 mmol) was added. The flask was then connected to a condenser, and the reaction mixture was refluxed at 60 °C for 12 h. Then the reaction was cooled to room temperature and washed with aqueous $NaHCO_3$. The organic layer was dried with anhydrous Na_2SO_4 and removed under reduced pressure to afford the crude product. The dr of the product was determined by ^{1}H NMR analysis of the crude product which was purified by column chromatography (PE/EtOAc = 4/1) to afford the pure product **14** as a colourless oil (342.2 mg, 92%, >20:1 dr, >99% ee). ^{1}H NMR (400 MHz, $CDCl_3$) δ 7.37–7.27 (m, 5H), 6.87 (dd, J = 15.6, 8.5 Hz, 1H), 5.87 (d, J = 15.6 Hz, 1H), 4.64 (dd, J = 9.7, 4.1 Hz, 1H), 4.19 (q, J = 7.1 Hz, 2H), 2.66–2.59 (m, 1H), 1.90–1.83 (m, 2H), 1.71–1.63 (m, 1H), 1.30 (t, J = 7.1 Hz, 3H), 1.10 (d, J = 6.8 Hz, 3H); ^{13}C NMR (101 MHz, $CDCl_3$) δ 166.9, 153.4, 144.7, 128.6, 127.7, 125.7, 120.6, 72.3, 60.3, 45.4, 33.5, 20.2, 14.3; HRMS (ESI) m/z calcd. for $C_{15}H_{20}NaO_3$ [M+Na]$^+$: 271.1305, found 271.1306; $[\alpha]_D^{20}$ = –21.5 (c 0.546, CH_2Cl_2); HPLC conditions: Chiralcel OD-H column, n-hexane/i-PrOH = 96:4, flow rate = 0.8 mL/min, uv-vis detection at λ = 210 nm, t_R = 14.096 min (minor), 16.163 min (major).

Step 2: Synthesis of ethyl 2-((*2S,3R,5S*)-3-methyl-5-phenyltetrahydrofuran-2-

yl)acetate (15)

To a stirring solution of KHMDS (0.91 mmol, 181.5 mg) in THF (5 mL) was added 14 (178.8 mg, 0.72 mmol) with THF (15 mL) via a syringe over 30 min at –78 °C. The reaction mixture was stirred at this temperature for 2 hours. The reaction was diluted in EtOAc (5 mL) and quenched with saturated NH_4Cl (5 mL) at –78 °C. The aqueous phase was extracted with EtOAc (10 mL × 3). The combined organic layers were dried with anhydrous Na_2SO_4 and removed under reduced pressure to afford the crude product, The dr of the product was determined by ^{1}H NMR analysis of the crude product which was purified by column chromatography (PE/EtOAc = 10/1) to afford pure product **15** as a colourless oil (160.7 mg, 90%, 12:1 dr, >99% ee). ^{1}H NMR (400 MHz, $CDCl_3$) δ 7.34–7.21 (m, 5H), 5.03 (dd, J = 7.5, 6.2 Hz, 1H), 4.23–4.15 (m, 2H), 4.04–3.99 (m, 1H), 2.71–2.60 (m, 2H), 2.12–2.00 (m, 3H), 1.28 (t, J = 7.1 Hz, 3H), 1.10 (d, J = 6.3 Hz, 3H); ^{13}C NMR (101 MHz, $CDCl_3$) δ 171.5, 143.6, 128.2, 127.1, 125.6, 82.7, 79.5, 60.6, 42.7, 40.0, 38.3, 17.2, 14.2; HRMS (ESI) m/z calcd. for $C_{15}H_{20}NaO_3$ $[M+Na]^+$: 271.1305, found 271.1303; $[\alpha]_D^{20}$ = –11.6 (c 0.107, CH_2Cl_2); HPLC conditions: Chiralcel OD-OD-H column, n-hexane/i-PrOH = 98:2, flow rate = 0.4 mL/min, uv-vis detection at λ = 210 nm, t_R = 37.276 min (major), 105.951 min (minor).

Step 3: Synthesis of ethyl (*3S,4R,E*)-3-acetoxy-4-methyl-6-phenylhex-5-enoate (16)

To a stirring suspension of $Zn(OTf)_2$ (0.02 mmol, 7.3 mg) in toluene (1.0 mL) in a 10 mL round-bottom flask was added **15** (0.4 mmol, 99.3 mg) with toluene (5 mL) via a syringe over 10 min. Acetic anhydride (0.72 mL) was added after 2 min. The reaction mixture was stirred in a 60 °C oil bath for 5 hours. The reaction was diluted with EtOAc and quenched with saturated $NaHCO_3$ at room temperature. The layers were separated and the aqueous phase was extracted with EtOAc (10 mL × 3). The organic layer was dried with anhydrous Na_2SO_4 and removed under reduced pressure to afford the crude product. The dr of the product was determined by ^{1}H NMR analysis of the crude product which was purified by column chromatography (PE/EtOAc = 10/1) to afford pure product **16** as a colourless oil (110.2 mg, 95%, >20:1 dr, >99% ee). ^{1}H NMR (400 MHz, $CDCl_3$) δ 7.34–7.18 (m, 5H), 6.40 (d, J = 15.9 Hz, 1H), 6.08 (dd, J = 15.9, 8.3 Hz, 1H), 5.34–5.30 (m, 1H), 4.13–4.08 (m, 2H), 2.68–2.53 (m, 3H), 2.02 (s, 3H), 1.21 (t, J = 7.2 Hz, 3H), 1.11 (d, J = 6.9 Hz, 3H); ^{13}C NMR (101 MHz, $CDCl_3$) δ 170.6, 170.3, 137.2, 131.6, 130.2, 128.6, 127.4, 126.2, 73.2, 60.7, 41.0, 37.0, 21.0, 16.2, 14.2; HRMS (ESI) m/z calcd. for $C_{17}H_{22}NaO_4$ $[M+Na]^+$: 313.1410, found 313.1411; $[\alpha]_D^{20}$ = +9.4 (c 0.229, CH_2Cl_2); HPLC conditions: Chiralpak AS-H column, n-hexane/i-PrOH = 95:5, flow rate = 1.0 mL/min, uv-vis detection at λ = 210 nm, t_R = 5.989 min (major), 7.452 min

(minor).

Step 4: Synthesis of (*2S,3S*)-3-acetoxy-5-ethoxy-2-methyl-5-oxopentanoic acid (17)

To a stirring solution of $NaIO_4$ (1.0 mmol, 213.9 mg) in DI H_2O (1.0 mL) was added a catalytic amount of $RuCl_3$•$(H_2O)n$ (0.02 mmol, 4.1 mg) at room temperature. Then MeCN (1.0 mL) was added and stirred vigorously for 10 min at room temperature. The **16** (0.2 mmol, 58.1 mg) in DCM (1.0 mL) was added slowly. The reaction mixture was stirred at room temperature for 2 hours. The reaction was diluted in CH_2Cl_2 and then quenched with a saturated NH_4Cl solution. The layers were separated and the aqueous phase was extracted with EtOAc (10 mL × 3). The organic layer was dried with anhydrous Na_2SO_4 and removed under reduced pressure to afford the crude product. The dr of the product was determined by ^{1}H NMR analysis of the crude product which was purified by column chromatography (PE/EtOAc = 1/1) to afford pure product **17** as a colourless oil (37.2 mg, 80%, >20:1 dr, 99% ee). ^{1}H NMR (400 MHz, $CDCl_3$) δ 5.52–5.50 (m, 1H), 4.08 (q, J = 7.1 Hz, 2H), 2.69–2.48 (m, 3H), 1.99 (s, 3H), 1.21 (t, J = 7.1 Hz, 3H), 1.03 (d, J = 6.7 Hz, 3H); ^{13}C NMR (101 MHz, $CDCl_3$) δ 179.8, 171.9, 171.2, 72.9, 60.7, 44.9, 36.0, 21.1, 14.1, 12.1; HRMS (ESI) m/z calcd. for $C_{10}H_{16}NaO_6$ $[M+Na]^+$: 255.0839, found 255.0842; $[\alpha]_D^{20}$ = +7.2 (*c* 1.361, CH_2Cl_2); HPLC conditions: Chiralpak AS-H column, *n*-hexane/*i*-PrOH = 85:15, flow rate = 1.0 mL/min, uv-vis detection at λ = 210 nm, t_R = 36.223 min (major), 40.620 min (minor).

4.3 Scale-up synthesis of 3y and 3z

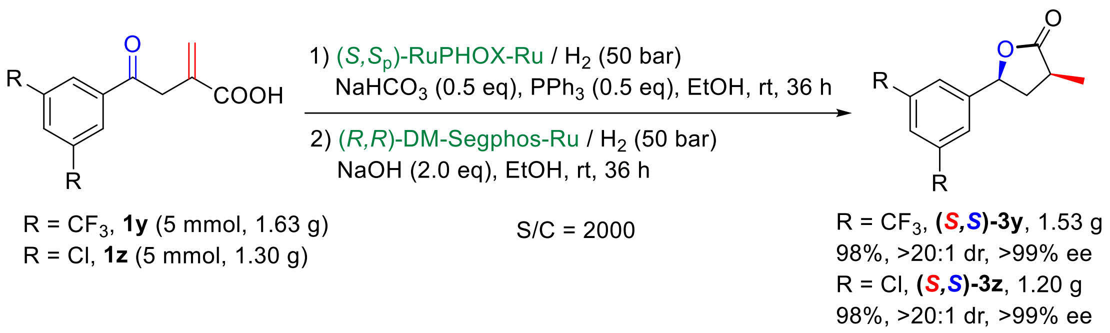

α-Methylene-*γ*-keto carboxylic acid **1y** or **1z** (5 mmol), $NaHCO_3$ (2.5 mmol, 0.21 g), PPh_3 (2.5 mmol, 0.66 g) and (*S,S*p)-RuPHOX-Ru (0.05 mol%, 4.3 mg) were added into a 100 mL beaker. The beaker was placed in an autoclave and EtOH (15 mL) was transferred into it by a syringe under nitrogen. The autoclave was replaced with hydrogen for five times and charged with hydrogen to 50 atm. The reaction mixture was stirred at room temperature for 36 hours. NaOH (10 mmol, 0.40 g) with EtOH (8 mL) was added into the autoclave by a syringe and then (*S,S*)-DM-Segphos-Ru (0.05 mol%, 3.0 mg) with EtOH (7 mL) was transferred into the autoclave by a syringe after

the first step reaction was completed. The autoclave was again replaced with hydrogen for five times and charged with hydrogen to 50 atm. The reaction mixture was stirred at room temperature for another 36 hours. Hydrogen gas was released slowly and the reaction solvent was removed under reduced pressure. The residue was dissolved with DCM (10 mL) and acidified with HCl (3N) to pH = 1. After separation of the two layers, the aqueous phase was extracted with DCM (10 mL × 3). The combined organic layer was dried over anhydrous Na_2SO_4 and removed under reduced pressure to afford the crude product. The dr of the product was determined by ^{1}H NMR analysis of the crude product which was purified by column chromatography (PE/EtOAc = 10/1) to afford the pure products **3y** or **3z**.

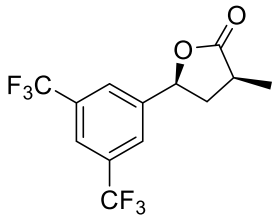

(*3S,5S*)-5-(3,5-Bis(trifluoromethyl)phenyl)-3-methyldihydrofuran-2(*3H*)-one (3y)

Colourless oil (1.53 g, 98%, >20:1 dr, >99% ee). ^{1}H NMR (400 MHz, $CDCl_3$) δ 7.85 (s, 1H), 7.81 (s, 2H), 5.46 (dd, J = 10.4, 5.0 Hz, 1H), 2.97–2.84 (m, 2H), 1.87–1.77 (m, 1H), 1.36 (d, J = 6.8 Hz, 3H); ^{13}C NMR (101 MHz, $CDCl_3$) δ 178.1, 141.9, 132.3 (q, J = 33.7 Hz), 125.5 (d, J = 3.9 Hz), 123.1 (q, J = 273.8 Hz), 122.3 (q, J = 3.6 Hz), 77.3, 39.8, 36.2, 14.8; ^{19}F NMR (377 MHz, $CDCl_3$) δ –63.0; HRMS (ESI) m/z calcd. for $C_{13}H_{10}F_6NaO_2$ $[M+Na]^+$: 335.0477, found 335.0479; $[\alpha]_D^{20}$ = –28.5 (c 0.860, CH_2Cl_2); HPLC conditions: Chiralcel OZ-H column, n-hexane/i-PrOH = 98:2, flow rate = 0.6 mL/min, uv-vis detection at λ = 210 nm, t_R = 20.852 min (minor), 23.962 min (major).

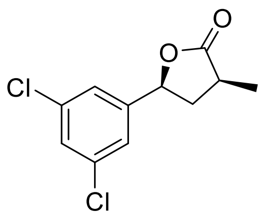

(*3S,5S*)-5-(3,5-Dichlorophenyl)-3-methyldihydrofuran-2(*3H*)-one (3z)

Colourless oil (1.20 g, 98%, >20:1 dr, >99% ee). ^{1}H NMR (400 MHz, $CDCl_3$) δ 7.32 (s, 1H), 7.23 (s, 2H), 5.28 (dd, J = 10.6, 5.2 Hz, 1H), 2.88–2.77 (m, 2H), 1.83–1.72 (m, 1H), 1.32 (d, J = 8.3 Hz, 3H); ^{13}C NMR (101 MHz, $CDCl_3$) δ 178.3, 142.7, 135.5, 128.5, 123.9, 77.4, 39.7, 36.1, 14.9; HRMS (ESI) m/z calcd. for $C_{11}H_{10}Cl_2NaO_2$ $[M+Na]^+$: 266.9950, found 266.9951; $[\alpha]_D^{20}$ = –30.7 (c 0.300, CH_2Cl_2); HPLC conditions: Chiralcel OD-H column, n-hexane/i-PrOH = 99:1, flow rate = 0.3 mL/min, uv-vis detection at λ

= 210 nm, t_R = 65.790 min (minor), 72.933 min (major).

4.3.1 Synthesis of cyclic carbamate (20) and (21)[13a-13c]

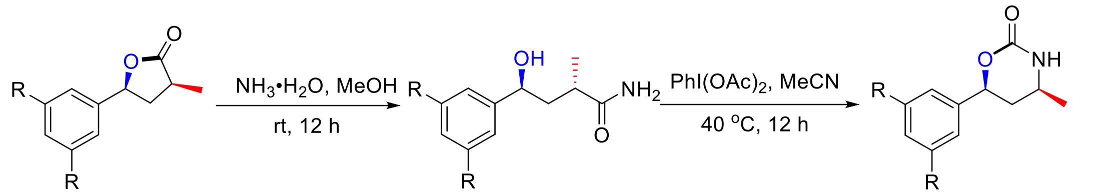

Step 1: Synthesis of (*2S,4S*)-4-hydroxy-2-methyl-4-phenylbutanamide (18) and (19)

To a solution of ***(S,S)*-3** (0.40 mmol) in methanol (4 mL) was added ammonium hydroxide (1 mL) dropwise. The reaction was stirred at room temperature for 12 hours. Then the reaction solvent was removed under reduced pressure to afford the crude product. The dr of the product was determined by ^{1}H NMR analysis of the crude product which was purified by column chromatography (PE/EtOAc = 1/1) to afford the pure products **18** and **19**.

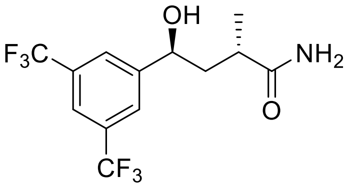

(*2S,4S*)-4-(3,5-Bis(trifluoromethyl)phenyl)-4-hydroxy-2-methylbutanamide (18)

Colourless oil (121.1 mg, 92%, >20:1 dr, >99% ee). ^{1}H NMR (400 MHz, $CDCl_3$) δ 7.81 (s, 2H), 7.76 (s, 1H), 6.04 (s, 1H), 5.87 (s, 1H), 4.85 (d, J = 9.2 Hz, 1H), 3.71 (s, 1H), 2.64–2.55 (m, 1H), 2.18–2.10 (m, 1H), 1.74–1.68 (m, 1H), 1.26 (d, J = 6.8 Hz, 3H); ^{13}C NMR (101 MHz, Methanol-d_4) δ 180.7, 148.8, 131.3 (q, J = 33.2 Hz), 126.2, 123.5 (q, J = 273.0 Hz), 120.8–120.2 (m), 70.1, 42.5, 36.7, 16.7. ^{19}F NMR (377 MHz, MeOD) δ –64.3; HRMS (ESI) m/z calcd. for $C_{13}H_{13}F_6NNaO_2$ $[M+Na]^+$: 352.0743, found 352.0743; $[\alpha]_D^{20}$ = –22.6 (c 0.195, CH_2Cl_2); HPLC conditions: Chiralpak AS-H column, n-hexane/i-PrOH = 85:15, flow rate = 1.0 mL/min, uv-vis detection at λ = 210 nm, t_R = 7.206 min (minor), 11.717 min (major).

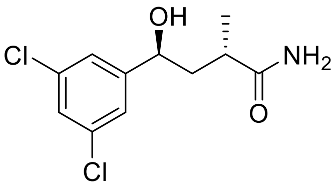

(*2S,4S*)-4-(3,5-Dichlorophenyl)-4-hydroxy-2-methylbutanamide (19)

White solid (93.9 mg, 90%, >20:1 dr, >99% ee). Mp: 55–57 °C; ^{1}H NMR (400 MHz, $CDCl_3$) δ 7.20 (s, 1H), 7.17 (s, 2H), 6.32 (s, 1H), 6.23 (s, 1H), 4.58 (dd, J = 9.7, 3.9 Hz,

1H), 4.23 (s, 1H), 2.52–2.44 (m, 1H), 2.07–1.99 (m, 1H), 1.63–1.57 (m, 1H), 1.16 (d, *J* = 7.0 Hz, 3H); ^{13}C NMR (101 MHz, $CDCl_3$) δ 180.2, 148.3, 134.9, 127.5, 124.3, 71.9, 43.3, 38.2, 18.2; HRMS (ESI) m/z calcd. for $C_{11}H_{13}Cl_2NNaO_2$ $[M+Na]^+$: 284.0216, found 284.0216; $[\alpha]_D^{20}$ = –21.8 (*c* 0.312, CH_2Cl_2; HPLC conditions: Chiralpak AS-H column, *n*-hexane/*i*-PrOH = 85:15, flow rate = 1.0 mL/min, uv-vis detection at λ = 200 nm, t_R = 24.347 min (minor), 36.636 min (major).

Step 2: Synthesis of (*4S,6S*)-6-(3,5-bis(trifluoromethyl)phenyl)-4-methyl-1,3-oxazinan-2-one (20) and (*4S,6S*)-6-(3,5-dichlorophenyl)-4-methyl-1,3-oxazinan-2-one (21)

To a solution of **(*S*,S)-18** or **19** (0.25 mmol) in acetonitrile (3 mL) in a 15 mL screw-capped tube were added $PhI(OAc)_2$ (0.25 mmol, 80.5 mg). The reaction was stirred at 40 °C in an oil bath for 12 hours. After the reaction was over, the reaction solution was removed under reduced pressure to afford the crude product. The dr of the product was determined by ^{1}H NMR analysis of the crude product which was purified by column chromatography (PE/EtOAc = 1/1) to afford the pure products **20** and **21**.

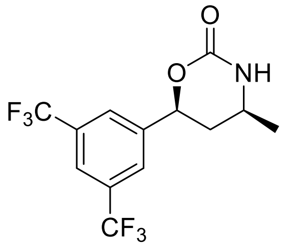

(*4S,6S*)-6-(3,5-Bis(trifluoromethyl)phenyl)-4-methyl-1,3-oxazinan-2-one (20)

White solid (74.3 mg, 92%, >20:1 dr, >99% ee). Mp: 198–200 °C; ^{1}H NMR (400 MHz, $CDCl_3$) δ 7.87 (s, 3H), 6.49 (s, 1H), 5.42 (dd, *J* = 11.8, 2.2 Hz, 1H), 3.87–3.79 (m, 1H), 2.32–2.27 (m, 1H), 1.77–1.68 (m, 1H), 1.32 (d, *J* = 6.2 Hz, 3H); ^{13}C NMR (101 MHz, $CDCl_3$) δ 153.7, 141.5, 132.2 (q, *J* = 33.7 Hz), 126.0 (d, *J* = 4.0 Hz), 123.1 (q, *J* = 274.4 Hz), 122.78–122.10 (m), 77.1, 46.8, 37.8, 21.8. ^{19}F NMR (377 MHz, $CDCl_3$) δ –62.9; HRMS (ESI) m/z calcd. for $C_{13}H_{11}F_6NNaO_2$ $[M+Na]^+$: 350.0586, found 350.0587; $[\alpha]_D^{20}$ = –36.7 (*c* 0.793, CH_2Cl_2); HPLC conditions: Chiralpak IC-H column, *n*-hexane/*i*-PrOH = 87:13, flow rate = 1.0 mL/min, uv-vis detection at λ = 210 nm, t_R = 17.448 min (major), 30.446 min (minor).

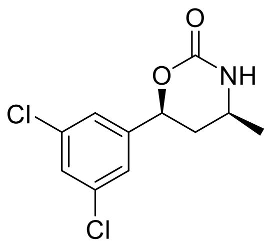

(4*S*,6*S*)-6-(3,5-Dichlorophenyl)-4-methyl-1,3-oxazinan-2-one (21)

Colourless oil (57.0 mg, 88%, >20:1 dr, >99% ee). ^{1}H NMR (400 MHz, $CDCl_3$) δ 7.25 (t, J = 1.9 Hz, 1H), 7.21 (d, J = 2.0 Hz, 2H), 6.92 (s, 1H), 5.16 (dd, J = 11.8, 2.3 Hz, 1H), 3.73–3.65 (m, 1H), 2.16–2.12 (m, 1H), 1.63–1.53 (m, 1H), 1.22 (d, J = 6.3 Hz, 3H); ^{13}C NMR (101 MHz, $CDCl_3$) δ 154.2, 142.3, 135.3, 128.5, 124.3, 77.1, 46.6, 37.7, 21.7; HRMS (ESI) m/z calcd. for $C_{11}H_{11}Cl_2NNaO_2$ $[M+Na]^+$: 282.0059, found 282.0060; $[\alpha]_D^{20}$ = –19.3 (c 2.05, CH_2Cl_2); HPLC conditions: Chiralpak AS-H column, n-hexane/i-PrOH = 80:20, flow rate = 1.0 mL/min, uv-vis detection at λ = 210 nm, t_R = 25.173 min (minor), 40.590 min (major).

4.4 The scale-up synthesis of 5l

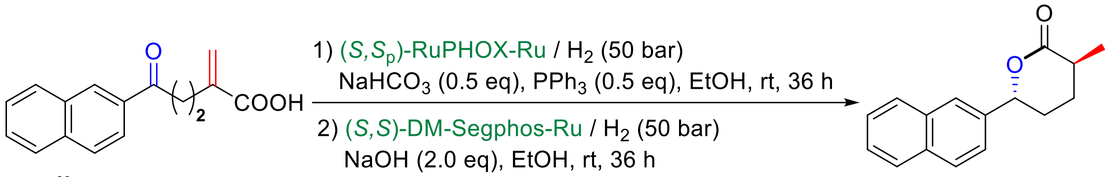

4l (5 mmol, 1.27 g)

(S,R)-5l, 1.17 g, 98%
> 20:1 dr, >99% ee

a-Methylene-*δ*-keto carboxylic acid **4l** (5 mmol, 1.27 g), $NaHCO_3$ (2.5 mmol, 0.21 g), PPh_3 (2.5 mmol, 0.66 g) and (S,S_p)-RuPHOX-Ru (0.05 mol%, 4.3 mg) were added into a 100 mL beaker. The beaker was placed in an autoclave and EtOH (15 mL) was transferred into it by a syringe under nitrogen atmosphere. The autoclave was replaced with hydrogen for five times and charged with hydrogen to 50 atm, and the reaction mixture was stirred at room temperature for 36 hours. The hydrogen gas was released slowly to 1 bar and NaOH (10 mmol, 0.40 g) with EtOH (8 mL) was added into the autoclave by syringe, then (S,S)-DM-Segphos-Ru (0.05 mol%, 3.0 mg) with EtOH (7 mL) was transferred into the autoclave by a syringe after the first step reaction was completed. The autoclave was again replaced with hydrogen for five times and charged with hydrogen to 50 atm, The reaction mixture was stirred at room temperature for another 36 hours. The hydrogen gas was released slowly and the reaction solvent was removed under reduced pressure. The residue was dissolved with DCM (10 mL) and acidified with HCl (3M) to pH = 1. After separation of the two layers, the aqueous phase was extracted with DCM (10 mL × 3). The combined organic layer was dried over anhydrous Na_2SO_4 and removed under reduced pressure to afford the crude product.

The dr of the product was determined by ^{1}H NMR analysis of the crude product which was purified by column chromatography (PE/EtOAc = 10/1) to afford the pure product **5l** (1.17 g, 98%, >20:1 dr, >99% ee).

4.4.1 Synthesis of (*2S,5S*)-2-methyl-5-(naphthalen-2-yl)hexanoic acid (22)[15]

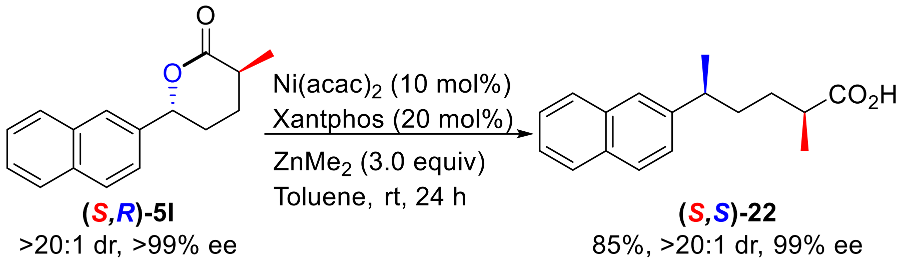

In a glovebox, **(*S*,*R*)-5l** (0.15 mmol, 36.1 mg) was added into a flame-dried 10 mL tube. $Ni(acac)_2$ (0.015 mmol, 3.9 mg), Xantphos (0.03 mmol, 17.3 mg), and toluene (1.5 mL) was added into another flame-dried 10 mL tube. After stirring for 10 min, half of the catalyst complex was transferred into the tube containing substrate, then $ZnMe_2$ (0.45 mmol, 1.0 M in toluene) was added. The reaction was stirred in the glovebox for 2 hours. Then the remaining catalyst complex was added into the reaction tube. The reaction was stirred for an additional 22 hours. The reaction was removed from the glovebox and quenched with 1M HCl (1.0 mL). After separation of the two layers, the aqueous phase was extracted with diethyl ether (2 mL × 3). The combined organic layer was dried over anhydrous Na_2SO_4 and removed under reduced pressure to afford the crude product. The dr of the product was determined by ^{1}H NMR analysis of the crude product which was purified by column chromatography (PE/EtOAc = 3/1) to afford the pure product **22** as a colourless oil (32.6 mg, 85%, >20:1 dr, 99% ee). ^{1}H NMR (400 MHz, $CDCl_3$) δ 7.81–7.77 (m, 3H), 7.60 (d, J = 1.7 Hz, 1H), 7.47–7.39 (m, 2H), 7.33 (dd, J = 8.6, 1.8 Hz, 1H), 2.91–2.82 (m, 1H), 2.50–2.39 (m, 1H), 1.79–1.64 (m, 2H), 1.63–1.53 (m, 1H), 1.49–1.39 (m, 1H), 1.33 (d, J = 6.9 Hz, 3H), 1.14 (d, J = 7.0 Hz, 3H); ^{13}C NMR (101 MHz, $CDCl_3$) δ 181.5, 144.5, 133.6, 132.3, 128.0, 127.59, 127.55, 125.9, 125.6, 125.2, 125.1, 40.0, 39.1, 35.3, 31.6, 22.3, 16.8; HRMS (ESI) m/z calcd. for $C_{17}H_{19}O_2$ $[M-H]^-$: 255.1391, found 255.1391; $[\alpha]_D^{20}$ = +18.14 (c 0.097, CH_2Cl_2); HPLC conditions: Chiralpak IC-H column, n-hexane/i-PrOH = 90:10, flow rate = 1.0 mL/min, uv-vis detection at λ = 210 nm, t_R = 14.269 min (major), 21.248 min (minor).

4.4.2 Synthesis of (*2R,5S*)-5-methyl-2-(naphthalen-2-yl)tetrahydro-2*H*-pyran (23)[9a]

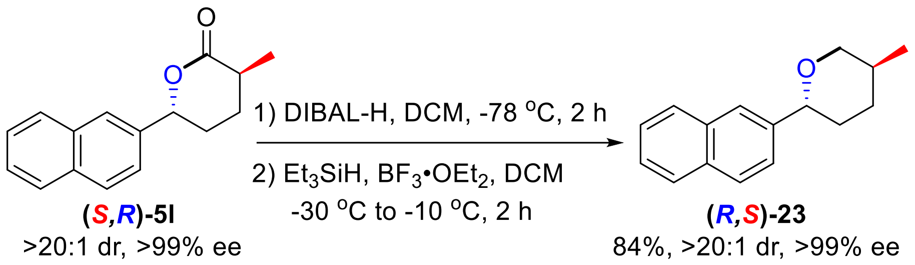

To a solution of **(*S*,*R*)-5l** (0.2 mmol, 48.1 mg) in dry DCM (2 mL) was added a solution of DIBAL-H (0.22 mmol, 1M in toluene) at –78 °C and stirred for 2 hours. The reaction was quenched with H_2O (0.4 mL) and NaOH (0.4 mL, 2M). After separation of the two layers, the aqueous phase was extracted with DCM (5 mL × 3). The combined organic layer was dried over anhydrous Na_2SO_4 and removed under reduced pressure to afford the crude product, which was directly used for the next step without further purification. Then Et_3SiH (0.4 mmol, 46.5 mg) and BF_3•OEt_2 (0.5 mmol, 0.12 mL) were added dropwise to the solution of crude product in dry DCM (2 mL) at –30 °C under nitrogen atmosphere. The resulting mixture was allowed to warm to –10 °C and stirred for 2 hours. Then saturated aqueous sodium bicarbonate solution (1.0 mL) was added and the aqueous layer was extracted with DCM (5 mL×3). The combined organic layer was dried over anhydrous Na_2SO_4 and removed under reduced pressure to afford the crude product. The dr of the product was determined by ^{1}H NMR analysis of the crude product which was purified by column chromatography (PE/EtOAc = 30/1) to afford the pure product **23** as a white solid (38.0 mg, 84%, >20:1 dr, >99% ee). Mp: 58–59 °C; ^{1}H NMR (400 MHz, $CDCl_3$) δ 7.82 (q, J = 4.7 Hz, 4H), 7.49–7.43 (m, 3H), 4.44 (dd, J = 11.4, 2.3 Hz, 1H), 4.12–4.07 (m, 1H), 3.26 (t, J = 11.1 Hz, 1H), 2.03–1.91 (m, 2H), 1.87–1.80 (m, 1H), 1.76–1.69 (m, 2H), 0.88 (d, J = 6.6 Hz, 3H); ^{13}C NMR (101 MHz, $CDCl_3$) δ 140.6, 133.4, 132.9, 128.0, 127.9, 127.6, 125.9, 125.6, 124.34, 124.31, 79.9, 75.3, 34.1, 32.9, 30.8, 17.3; HRMS (ESI) m/z calcd. for $C_{16}H_{18}NaO$ $[M+Na]^+$: 249.1250, found 249.1250; $[\alpha]_D^{20}$ = +38.0 (c 0.156, CH_2Cl_2); HPLC conditions: Chiralpak IC-IC-H column, n-hexane/i-PrOH = 99:1, flow rate = 0.2 mL/min, uv-vis detection at λ = 220 nm, t_R = 83.786 min (major), 175.951 min (minor).

4.5 Scale-up synthesis of 2h[16] and 3h

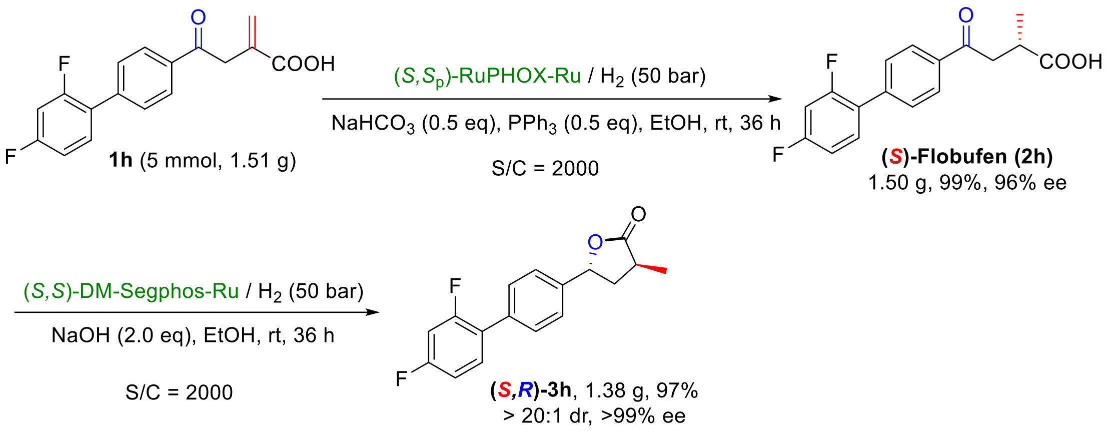

α-Methylene-*γ*-keto carboxylic acid **1h** (5 mmol, 1.51 g), $NaHCO_3$ (2.5 mmol, 0.21 g), PPh_3 (2.5 mmol, 0.66 g) and (*S*,*S*p)-RuPHOX-Ru (0.05 mol%, 4.3 mg) were added into a 50 mL beaker. The beaker was placed in an autoclave and EtOH (15 mL) was transferred into it by a syringe under nitrogen. The autoclave was replaced with hydrogen for five times and charged with hydrogen to 50 atm. The reaction mixture was stirred at room temperature for 36 hours. The hydrogen gas was released slowly and the solvent was removed under reduced pressure. The residue was dissolved with DCM (10 mL) and acidified with HCl (3M) to pH = 1. After separation of the two layers, the aqueous phase was extracted with DCM (10 mL × 3). The combined organic layer was dried over anhydrous Na_2SO_4 and removed under reduced pressure to afford the crude product. The residue was purified by column chromatography (PE/EtOAc = 2/1) to afford the (***S***)-Flobufen product (***S***)**-2h** as a white solid (1.50 g, 99%, 96% ee). Mp: 118–120 °C; ^{1}H NMR (400 MHz, $CDCl_3$) *δ* 8.04 (d, *J* = 8.4 Hz, 2H), 7.62–7.58 (m, 2H), 7.46–7.40 (m, 1H), 7.01–6.91 (m, 2H), 3.51 (dd, *J* = 17.7, 7.6 Hz, 1H), 3.24–3.15 (m, 1H), 3.08 (dd, *J* = 17.7, 5.5 Hz, 1H), 1.34 (d, *J* = 7.2 Hz, 3H); ^{13}C NMR (101 MHz, $CDCl_3$) *δ* 197.4, 181.7, 162.6 (dd, *J* = 302.0, 11.9 Hz), 160.1 (dd, *J* = 303.0, 12.1 Hz), 139.9, 135.6, 131.4 (dd, *J* = 9.6, 4.7 Hz), 129.1 (d, *J* = 3.2 Hz), 128.3, 124.2 (dd, *J* = 13.5, 3.9 Hz), 111.9 (dd, *J* = 21.2, 3.7 Hz), 104.7 (t, *J* = 25.7 Hz), 41.8, 34.8, 17.1; ^{19}F NMR (377 MHz, $CDCl_3$) *δ* –109.8, –112.9; $[\alpha]_D^{20}$ = –30.3 (*c* 0.447, CH_2Cl_2); HPLC conditions: Chiralcel OJ-H column, *n*-hexane/*i*-PrOH = 95:5, flow rate = 1.0 mL/min, uv-vis detection at λ = 254 nm, t_R = 38.879 min (major), 44.262 min (minor).

(***S***)**-2h** (4.95 mmol, 1.50 g), NaOH (9.9 mmol, 0.40 g), (*S*,*S*)-DM-Segphos-Ru (0.05 mol%, 3.0 mg) was added into a 50 mL beaker. The beaker was placed in an autoclave and EtOH (15 mL) was transferred into it by a syringe under nitrogen. The autoclave was replaced with hydrogen for five times and charged with hydrogen to 50 atm. The reaction mixture was stirred at room temperature for 36 hours. The hydrogen gas was released slowly and the solvent was removed under reduced pressure. The residue was

dissolved with DCM (10 mL) and acidified with HCl (3M) to pH = 1. After separation of the two layers, the aqueous phase was extracted with DCM (10 mL × 3). The combined organic layer was dried over anhydrous Na_2SO_4 and removed under reduced pressure to afford the crude product. The dr of the product was determined by ^1H NMR analysis of the crude product which was purified by column chromatography (PE/EtOAc = 10/1) to afford the pure product **3h** (1.38 g, 97%, >20:1 dr, >99% ee).

5. X-Ray Analysis

5.1 Crystal Structure of (*R*,*R*)-3a

X-Ray crystallography data for **(*R*,*R*)-3a** (CDCC: 2302115): A colorless crystal suitable for X-ray crystallography was obtained from a *n*-hexane/dichloromethane solution at room temperature under air.

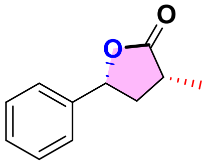

(*R*,*R*)-3a

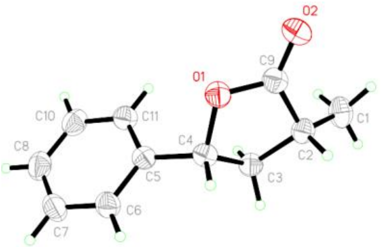

Figure1 The ORTEP drawing (50% probability for thermal ellipsoids) of **(*R*,*R*)-3a**

checkCIF/PLATON report

Structure factors have been supplied for datablock(s) t

THIS REPORT IS FOR GUIDANCE ONLY. IF USED AS PART OF A REVIEW PROCEDURE FOR PUBLICATION, IT SHOULD NOT REPLACE THE EXPERTISE OF AN EXPERIENCED CRYSTALLOGRAPHIC REFEREE.

No syntax errors found. CIF dictionary Interpreting this report

Datablock: t

Bond precision: C-C = 0.0030 A Wavelength=1.54178

Cell:	a=5.8551(3)	b=8.8937(4)	c=9.2870(5)
	alpha=90	beta=93.243(3)	gamma=90
Temperature:	173 K		

	Calculated	Reported
Volume	482.83(4)	482.83(4)
Space group	P 21	P 21
Hall group	P 2yb	P 2yb
Moiety formula	C11 H12 O2	C11 H12 O2
Sum formula	C11 H12 O2	C11 H12 O2
Mr	176.21	176.21
Dx,g cm-3	1.212	1.212
Z	2	2
Mu (mm-1)	0.665	0.665
F000	188.0	188.0
F000'	188.57	
h,k,lmax	7,10,11	7,10,11
Nref	1777[950]	1743
Tmin,Tmax	0.899,0.911	0.595,0.753
Tmin'	0.899	

Correction method= # Reported T Limits: Tmin=0.595 Tmax=0.753
AbsCorr = MULTI-SCAN

Data completeness= 1.83/0.98 Theta(max)= 68.538

R(reflections)= 0.0322(1661) wR2(reflections)= 0.1024(1743)

S = 0.873 Npar= 119

5.2 Crystal Structure of (*R*,*S*)-5a

X-Ray crystallography data for (***R*,*S***)**-5a** (CDCC: 2302116): A colorless crystal suitable for X-ray crystallography was obtained from a *n*-hexane/dichloromethane solution at room temperature under air.

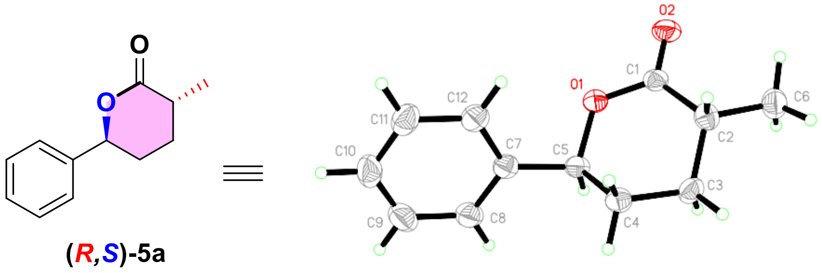

Figure1 The ORTEP drawing (50% probability for thermal ellipsoids) of (***R*,*S***)**-5a**

checkCIF/PLATON report

Structure factors have been supplied for datablock(s) t_a

THIS REPORT IS FOR GUIDANCE ONLY. IF USED AS PART OF A REVIEW PROCEDURE FOR PUBLICATION, IT SHOULD NOT REPLACE THE EXPERTISE OF AN EXPERIENCED CRYSTALLOGRAPHIC REFEREE.

No syntax errors found. CIF dictionary Interpreting this report

Datablock: t_a

Bond precision:	C-C = 0.0020 A		Wavelength=1.54178
Cell:	a=6.0918(1)	b=9.0002(2)	c=18.2506(4)
	alpha=90	beta=90	gamma=90
Temperature:	173 K		

	Calculated	Reported
Volume	1000.63(4)	1000.63(4)
Space group	P 21 21 21	P 21 21 21
Hall group	P 2ac 2ab	P 2ac 2ab
Moiety formula	C12 H14 O2	C12 H14 O2
Sum formula	C12 H14 O2	C12 H14 O2
Mr	190.23	190.23
Dx,g cm-3	1.263	1.263
Z	4	4
Mu (mm-1)	0.678	0.678
F000	408.0	408.0
F000'	409.22	
h,k,lmax	7,10,22	7,10,21
Nref	1833[1095]	1825
Tmin,Tmax	0.897,0.922	0.690,0.753
Tmin'	0.897	

Correction method= # Reported T Limits: Tmin=0.690 Tmax=0.753
AbsCorr = MULTI-SCAN

Data completeness= 1.67/1.00 Theta(max)= 68.346

R(reflections)= 0.0273(1768) wR2(reflections)= 0.0774(1825)

S = 1.040 Npar= 128

5.3 Crystal Structure of (*S*,*S*)-5a

X-Ray crystallography data for (***S***,***S***)-**5a** (CDCC: 2302118): A colorless crystal suitable for X-ray crystallography was obtained from a *n*-hexane/dichloromethane solution at room temperature under air.

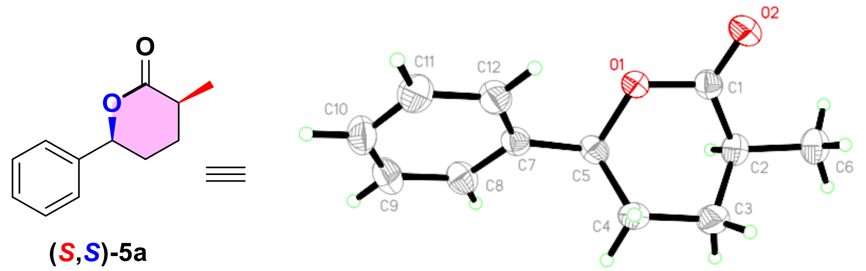

Figure1 The ORTEP drawing (50% probability for thermal ellipsoids) of (***S***,***S***)-**5a**

checkCIF/PLATON report

Structure factors have been supplied for datablock(s) t_a

THIS REPORT IS FOR GUIDANCE ONLY. IF USED AS PART OF A REVIEW PROCEDURE FOR PUBLICATION, IT SHOULD NOT REPLACE THE EXPERTISE OF AN EXPERIENCED CRYSTALLOGRAPHIC REFEREE.

No syntax errors found. CIF dictionary Interpreting this report

Datablock: t_a

Bond precision: C-C = 0.0021 A Wavelength=1.54178

Cell:	a=6.3114(2)	b=7.8294(2)	c=20.4533(5)
	alpha=90	beta=90	gamma=90
Temperature:	173 K		

	Calculated	Reported
Volume	1010.69(5)	1010.69(5)
Space group	P 21 21 21	P 21 21 21
Hall group	P 2ac 2ab	P 2ac 2ab
Moiety formula	C12 H14 O2	C12 H14 O2
Sum formula	C12 H14 O2	C12 H14 O2
Mr	190.23	190.23
Dx,g cm-3	1.250	1.250
Z	4	4
Mu (mm-1)	0.671	0.671
F000	408.0	408.0
F000'	409.22	
h,k,lmax	7,9,24	7,9,24
Nref	1861[1114]	1857
Tmin,Tmax	0.886,0.910	0.700,0.753
Tmin'	0.886	

Correction method= # Reported T Limits: Tmin=0.700 Tmax=0.753
AbsCorr = MULTI-SCAN

Data completeness= 1.67/1.00 Theta(max)= 68.393

R(reflections)= 0.0290(1789) wR2(reflections)= 0.0761(1857)

S = 1.096 Npar= 128

6. Reference

[1] a) C. Chen, Z. Zhang, S. Jin, X. Fan, M. Geng, Y. Zhou, S. Wen, X. Wang, L. W. Chung, X.-Q. Dong, X. Zhang, *Angew. Chem. Int. Ed*. **2017**, *56*, 6808–6812; b) X. Ren, Z. Wang, C. Shen, X. Tian, L. Tang, X. Ji, K. Dong, *Angew. Chem. Int. Ed*. **2021**, *60*, 17693–17700; c) C. Chen, S. Wen, M. Geng, S. Jin, Z. Zhang, X.-Q. Dong, X. Zhang, *Chem. Commun*. **2017**, *53*, 9785–9788.

[2] Y. Nie, Q. Yuan, W. Zhang, *Chem. Rec*. **2023**, *23*, e202300133.

[3] Y. Wang, G. Yang, F. Xie, W. Zhang, *Org. Lett*. **2018**, *20*, 6135−6139.

[4] a) E. J. Corey, R. K. Bakshi, S. Shibata, C.-P. Chen, V. K. Singh, *J. Am. Chem. Soc.* **1987**, *109*, 7925−7926; b) E. J. Corey, J. O. Link, *Tetrahedron Lett*. **1989**, *30*, 6275−6278; c) E. J. Corey, C. J. Helal, *Angew. Chem. Int. Ed*. **1998**, *37*, 1986−2012.

[5] a) R. Noyori, T. Ohkuma, M. Kitamura, H. Takaya, N. Sayo, H. Kumobayashi, S. Akutagawa, *J. Am. Chem. Soc*. **1987**, *109*, 5856–5858; b) M. Kitamura, T. Ohkuma, S. Inoue, N. Sayo, H. Kumobayashi, S. Akutagawa, T. Ohta, H. Takaya, R. Noyori, *J. Am. Chem. Soc*. **1988**, *110*, 629–631; c) R. Noyori, T. Ikeda, T. Ohkuma, M. Widhalm, M. Kitamura, H. Takaya, S. Akutagawa, N. Sayo, T. Saito, T. Taketomi, H. Kumobayashi, *J. Am. Chem. Soc*. **1989**, *111*, 9134–9135; d) T. Ohkuma, H. Ooka, S. Hashiguchi, T. Ikariya, R. Noyori, *J. Am. Chem. Soc*. **1995**, *117*, 2675–2676; e) T. Ohkuma, M. Koizumi, H. Doucet, T. Pham, M. Kozawa, K. Murata, E. Katayama, T. Yokozawa, T. Ikariya, R. Noyori, *J. Am. Chem. Soc*. **1998**, *120*, 13529–13530; f) R. Noyori, T. Ohkuma, *Angew. Chem. Int. Ed.* **2001**, *40*, 40–73; g) W. P. Hems, M. Groarke, A. Zanotti-Gerosa, G. A. Grasa, *Acc. Chem. Res*. **2007**, *40*, 1340–1347; h) C. A. Sandoval, Y. Li, K. Ding, R. Noyori, *Chem. Asian J*. **2008**, *3*, 1801–1810; i) K. Matsumura, N. Arai, K. Hori, T. Saito, N. Sayo, T. Ohkuma, *J. Am. Chem. Soc*. **2011**, *133*, 10696–10699; j) K. Wang, L. Zhang, W. Tang, H. Sun, D. Xue, M. Lei, J. Xiao, C. Wang, *Angew. Chem. Int. Ed*. **2020**, *59*, 11408–11415.

[6] a) ref. [1a]; b) C. Chen, S. Wen, X.-Q. Dong, X. Zhang, *Org. Chem. Front*. **2017**, *4*, 2034–2038; c) ref. [1c]; d) G. Poklukar, M. Stephan, B, Mohar, *Adv. Synth. Catal*. **2018**, *360*, 2566–2570; e) X. Zhang, Y. Gao, R. D. Laishram, K. Li, Y. Yang, Y. Zhan, Y. Luo, B. Fan, *Org. Biomol. Chem*. **2019**, *17*, 2174–2181; f) X. Liu, J. Wen, L. Yao, H. Nie, R. Jiang, W. Chen, X. Zhang, *Org. Lett*. **2020**, *22*, 4812−4816.

[7] a) X. Liu, C. Chen, H. Yuan, X. Tang, R. Jiang, Y. Gao, H. Nie, *Eur. J. Org. Chem*. **2022**, e202200673; b) A. K. Diba, C. Noll, M. Richter, M. T. Gieseler, M. Kalesse, *Angew. Chem. Int. Ed*. **2010**, *49*, 8367–8369.

[8] C.-Q. Deng, J. Deng, O*rg. Lett*. **2022**, *24*, 2494−2498.

[9] a) M. Xue, J. Cui, X. Zhu, F. Wang, D. Lv, Z. Nie, Y. Li, H. Bao, *Angew. Chem. Int. Ed.* **2023**, *62*, e202304275; b) N. Sakai, S. Horikawa, Y. Ogiwara, *Synthesis* **2018**, *50*, 565–574.

[10] a) W. Mahy, P. Plucinski, J. Jover, C. G. Frost, *Angew. Chem. Int. Ed*. **2015**, *54*, 10944–10948; b) Y. Xia, P. Yuan, Y. Zhang, Y. Sun, M. Hong, *Angew. Chem. Int. Ed*. **2023**, *62*, e202217812.

[11] a) ref. [8]; b). ref. [9a].

[12] A. Bunrit, P. Srifa, T. Rukkijakan, C. Dahlstrand, G. Huang, S. Biswas, R. A. Watile, J. S. M. Samec, *ACS Catal*. **2020**, *10*, 1344−1352.

[13] a) J. W. Hilborn, Z.-H. Lu, A. R. Jurgens, Q. K. Fang, P. Byers, S. A. Wald, C. H Senanayake, *Tetrahedron Lett*. **2001**, *42*, 8919–8921; b) G. Xiao, C. Xie, Q. Guo, G. Zi, G. Hou, Y. Huang, *Org. Lett*. **2022**, *24*, 2722−2727; c) ref. [8]; d) M. Hatano, K. Nishikawa, K. Ishihara, *J. Am. Chem. Soc*. **2017**, *139*, 8424−8427.

[14] a) A. K. Ghosh, L. Swanson, *J. Org. Chem*. **2003**, *68*, 9823–9826; b) A. K. Ghosh, S. Rodriguez, *Tetrahedron Lett.* **2016**, *57*, 2884–2887.

[15] E. J. Tollefson, D. D. Dawson, C. A. Osborne, E. R. Jarvo, *J. Am. Chem. Soc*. **2014**, *136*, 14951−14958.

[16] a) F. Trejtnar, R. Král, P. Pávek, V. Wsól, *Chirality* **2003**, *15*, 724–729; b) ref. [6f].

7. NMR Spectra

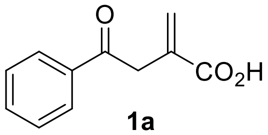

1a

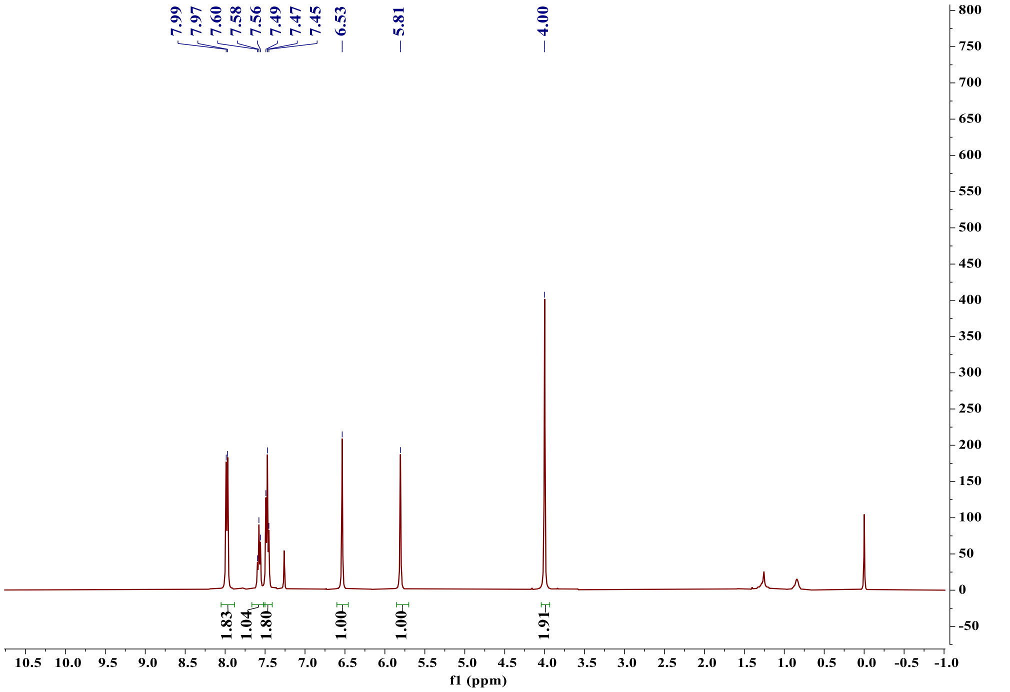

^{1}H NMR (400 MHz, $CDCl_3$) spectrum of **1a**

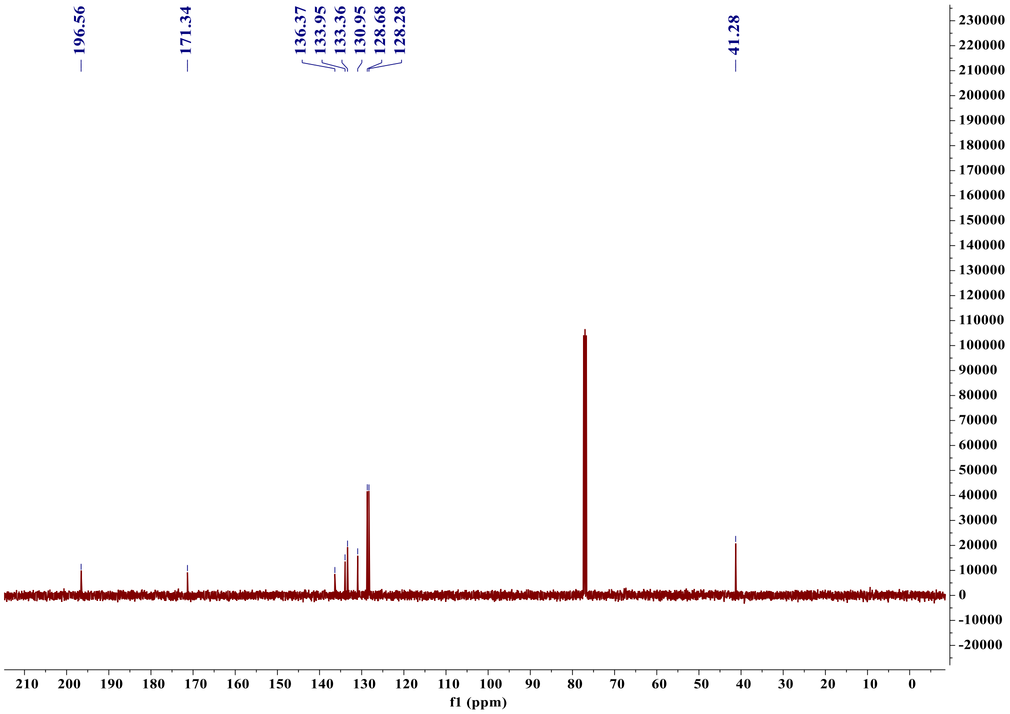

^{13}C NMR (101 MHz, $CDCl_3$) spectrum of **1a**

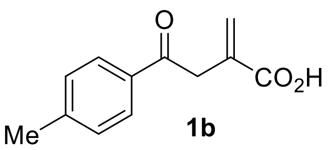

1b

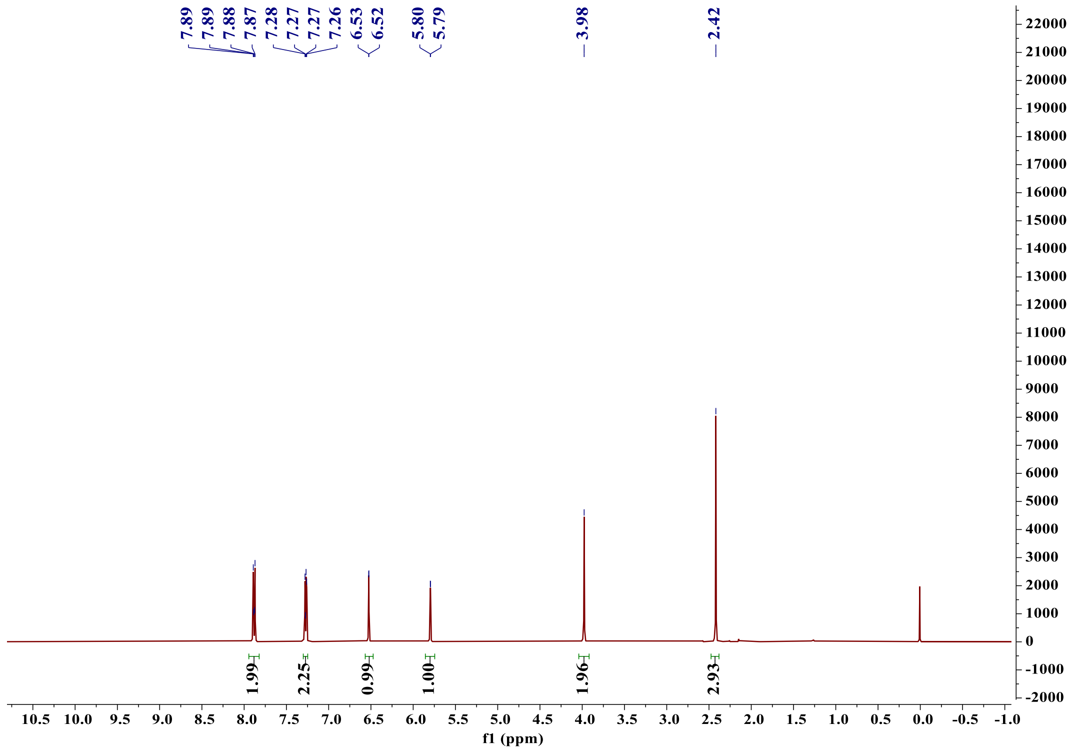

^{1}H NMR (400 MHz, $CDCl_3$) spectrum of **1b**

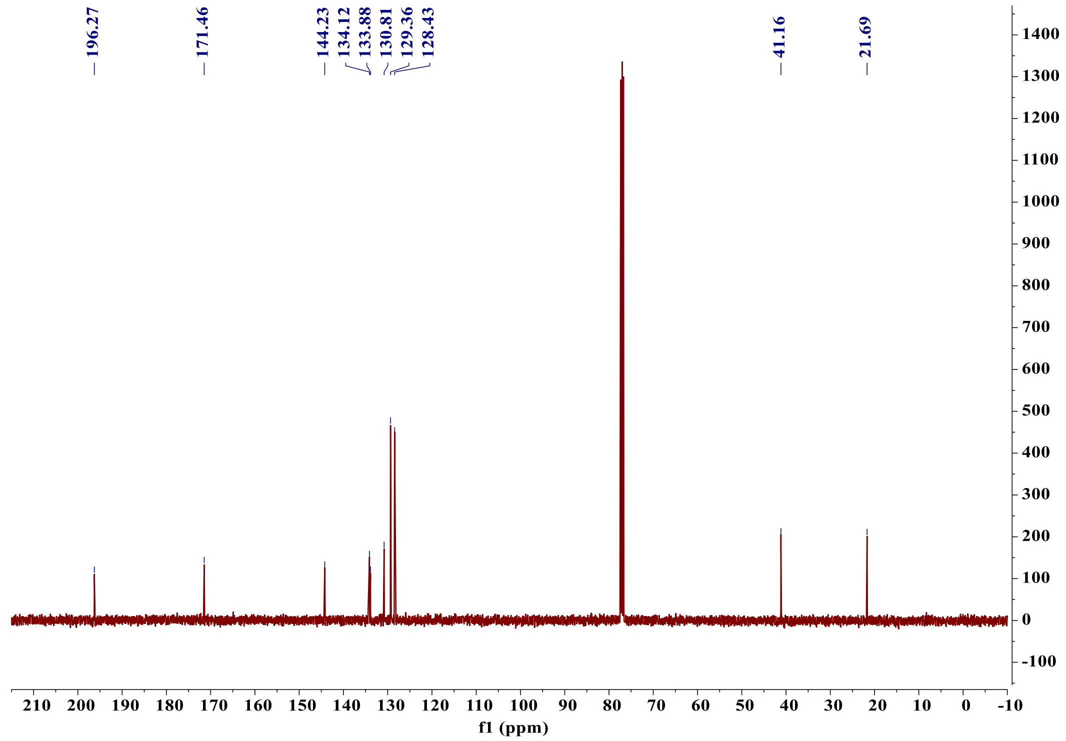

^{13}C NMR (101 MHz, $CDCl_3$) spectrum of **1b**

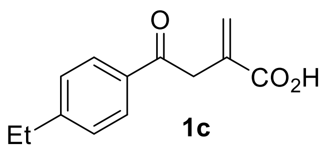

1c

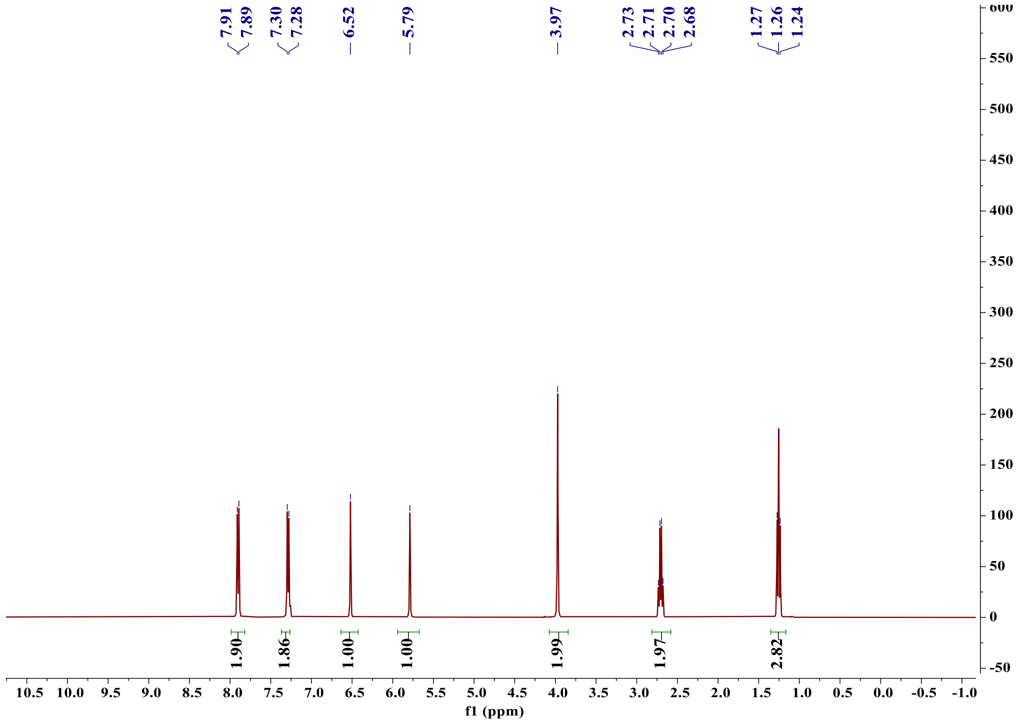

^{1}H NMR (400 MHz, $CDCl_3$) spectrum of **1c**

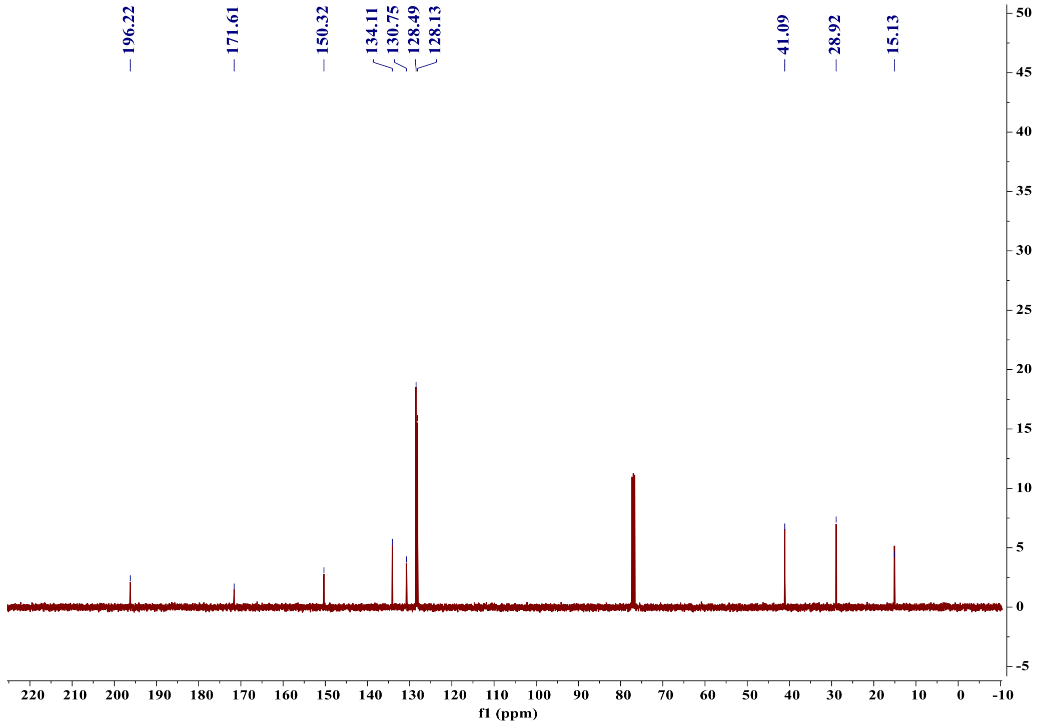

^{13}C NMR (101 MHz, $CDCl_3$) spectrum of **1c**

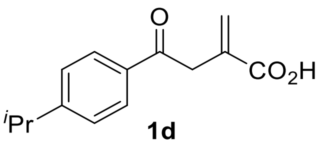

1d

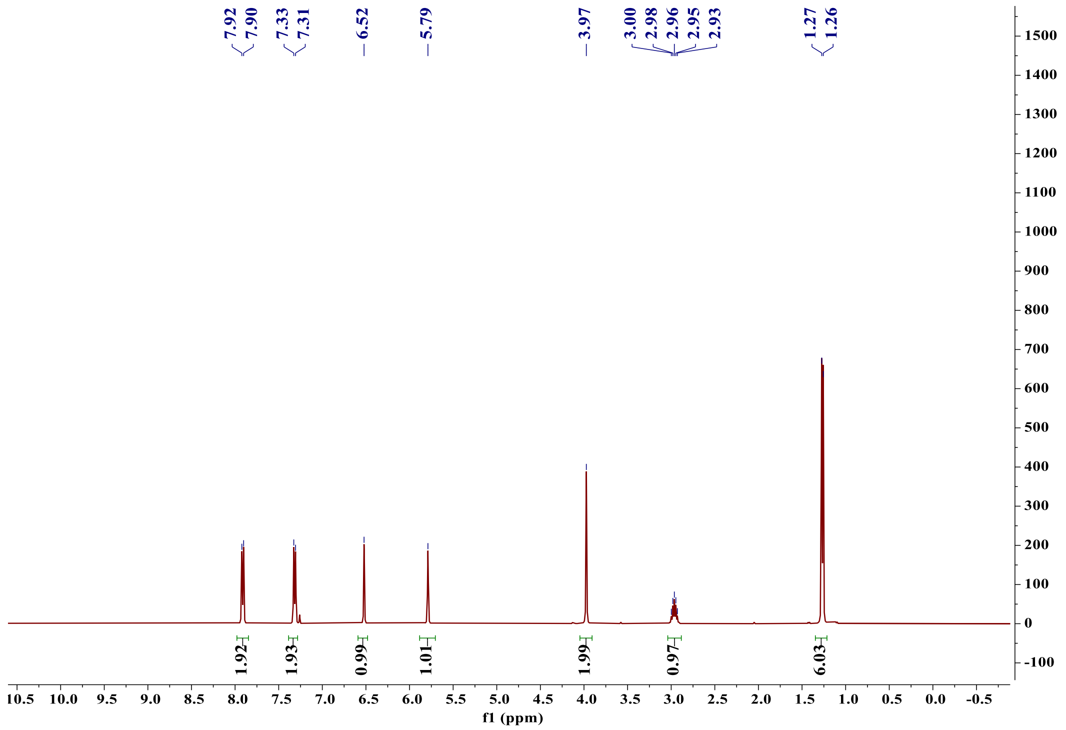

^{1}H NMR (400 MHz, $CDCl_3$) spectrum of **1d**

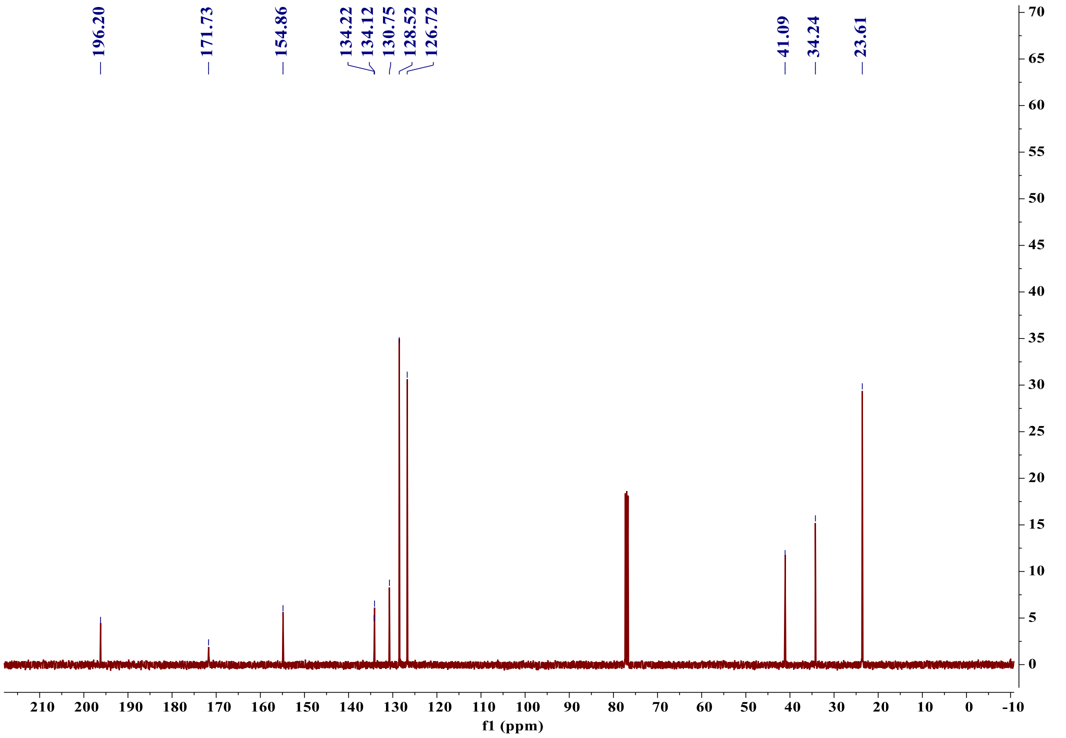

^{13}C NMR (101 MHz, $CDCl_3$) spectrum of **1d**

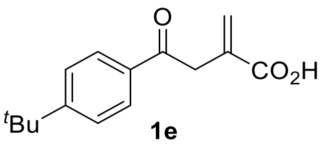

1e

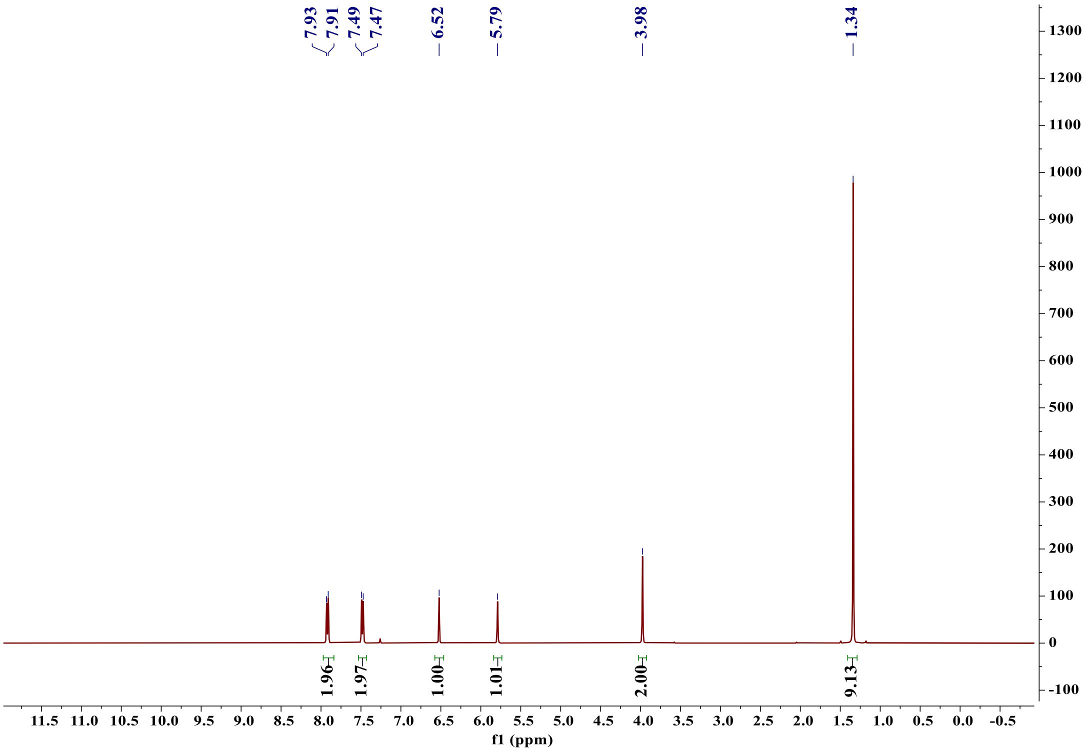

^{1}H NMR (400 MHz, $CDCl_3$) spectrum of **1e**

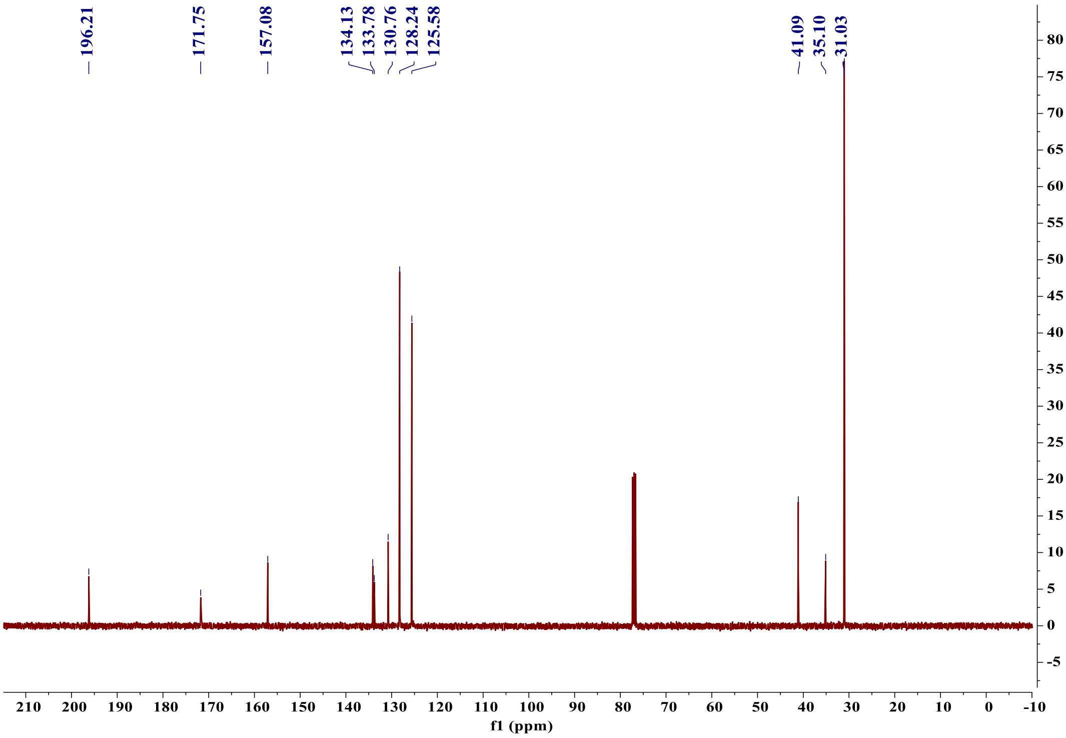

^{13}C NMR (101 MHz, $CDCl_3$) spectrum of **1e**

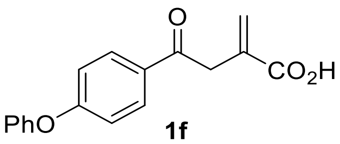

1f

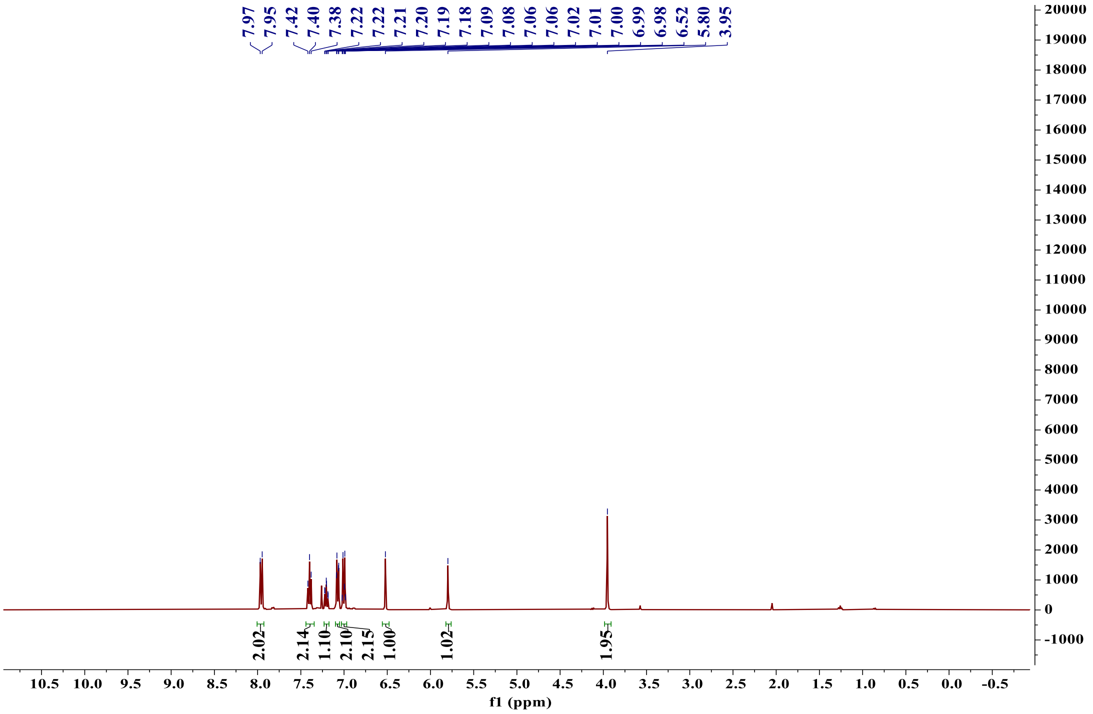

^1H NMR (400 MHz, $CDCl_3$) spectrum of **1f**

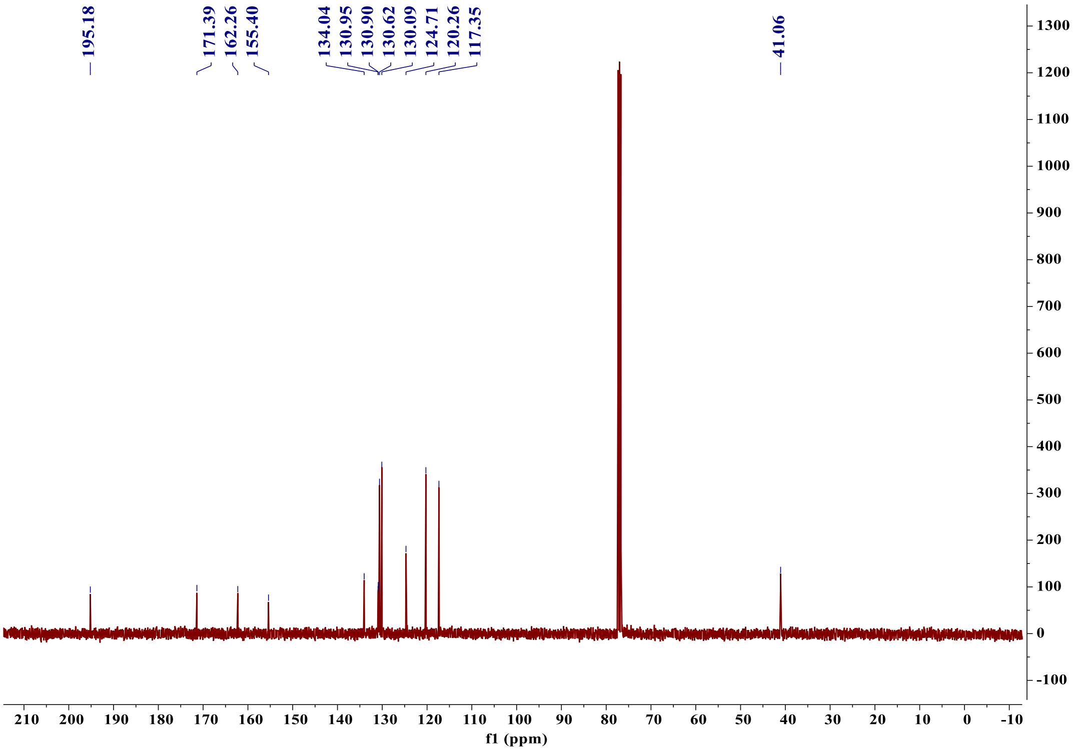

^{13}C NMR (101 MHz, $CDCl_3$) spectrum of **1f**

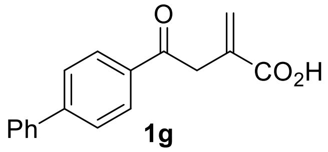

1g

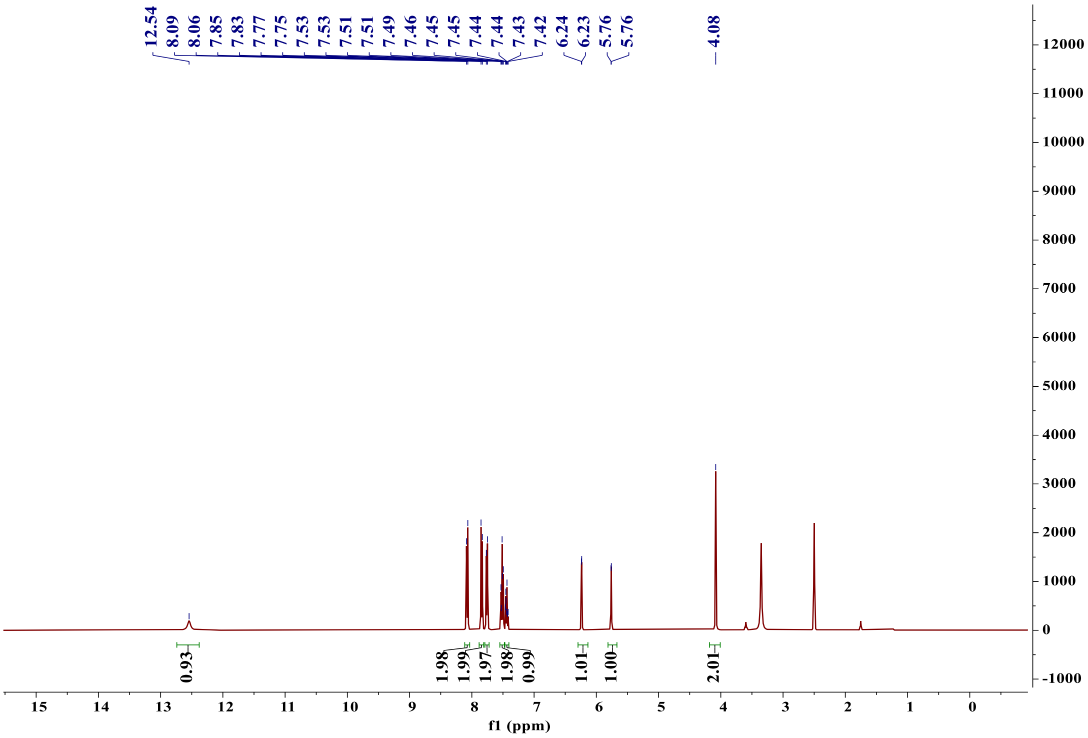

^{1}H NMR (400 MHz, DMSO-d_6) spectrum of **1g**

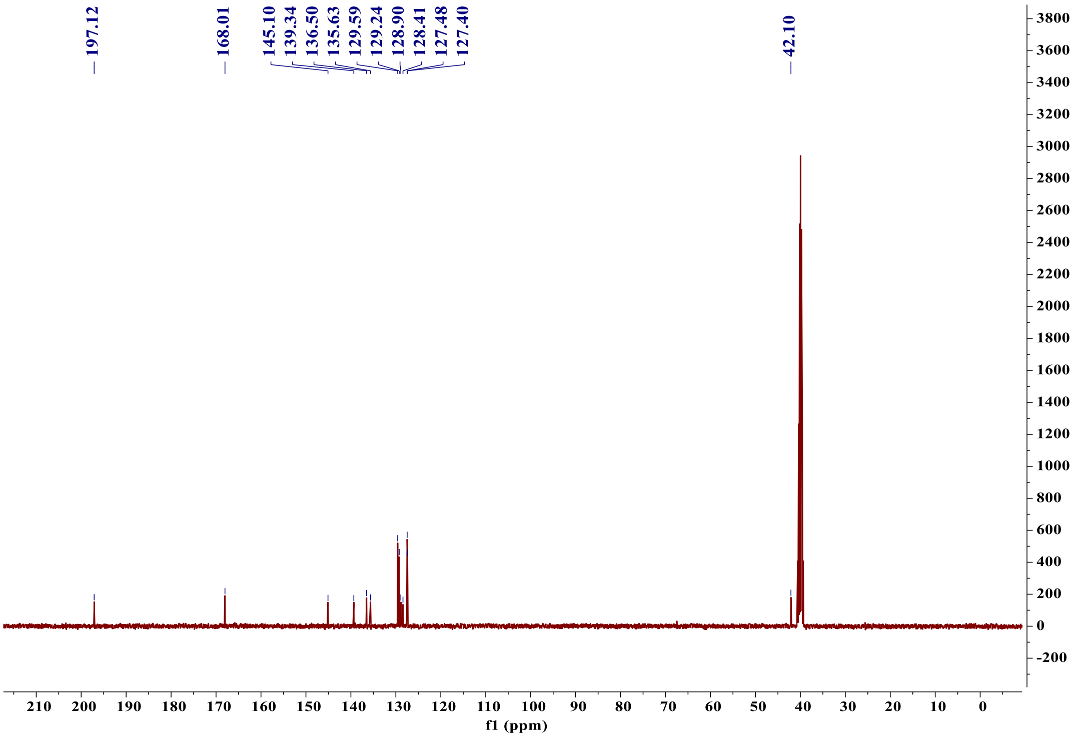

^{13}C NMR (101 MHz, DMSO-d_6) spectrum of **1g**

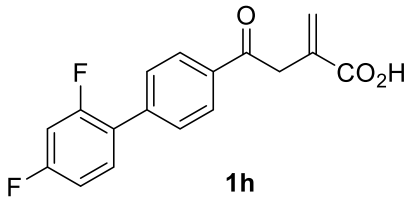

1h

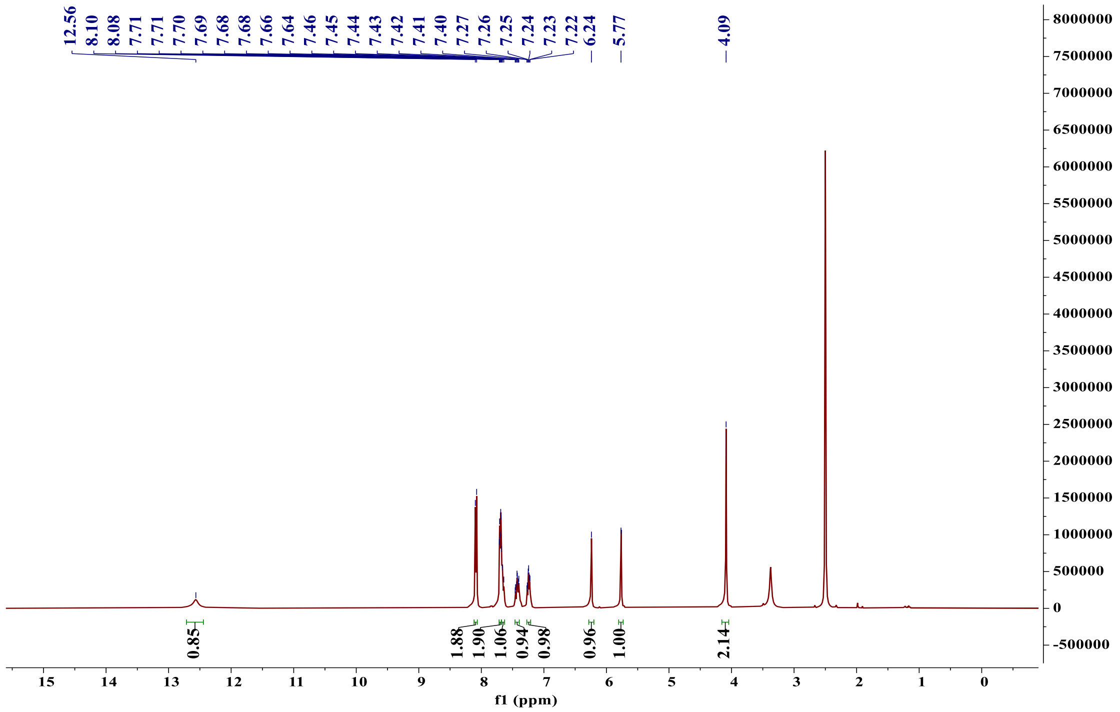

^{1}H NMR (400 MHz, DMSO-d_6) spectrum of **1h**

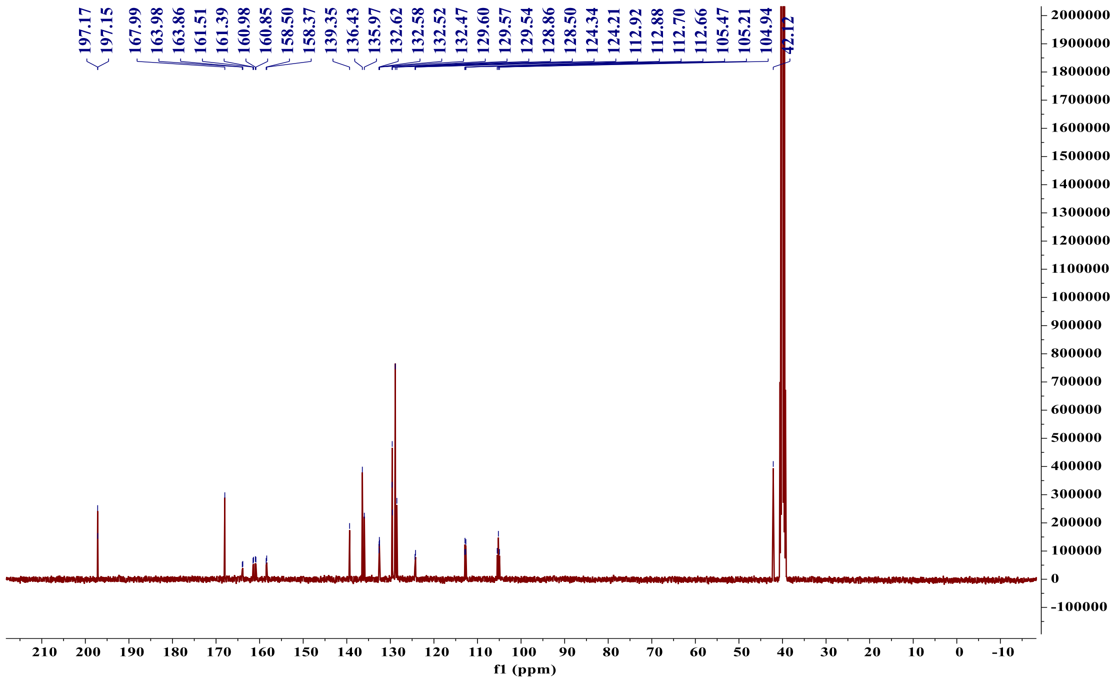

^{13}C NMR (101 MHz, DMSO-d_6) spectrum of **1h**

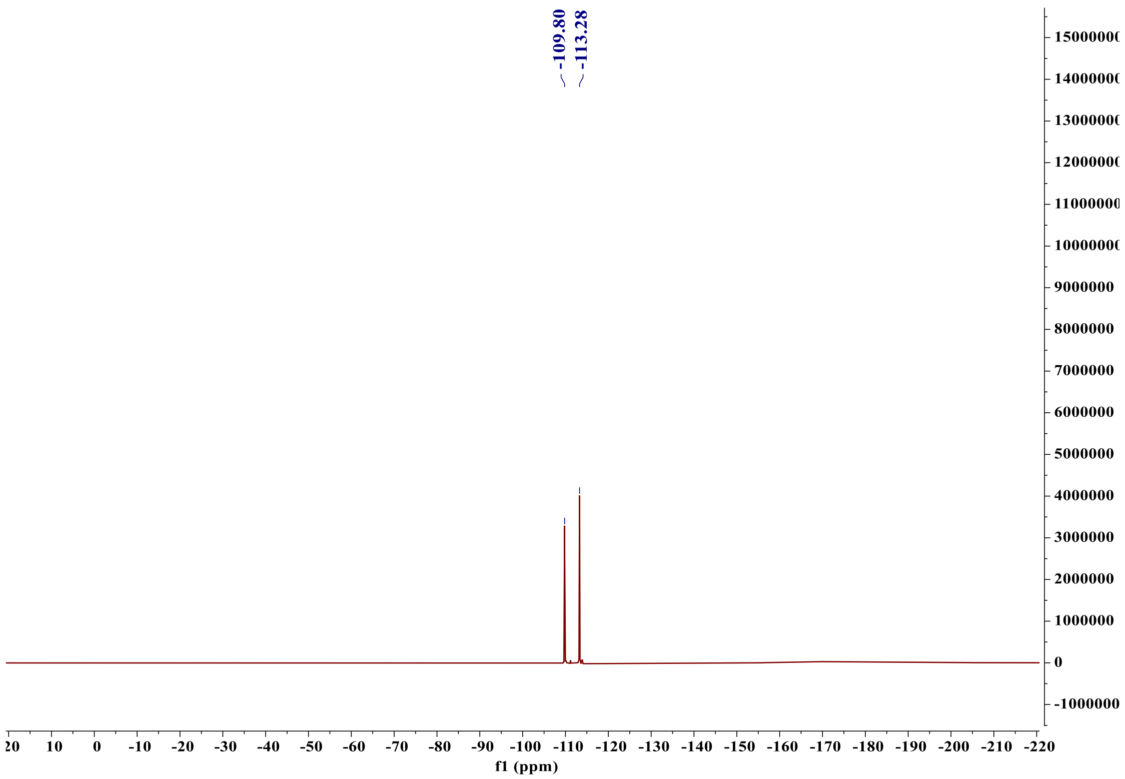

^{19}F NMR (377 MHz, DMSO-d_6) spectrum of **1h**

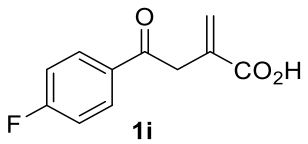

1i

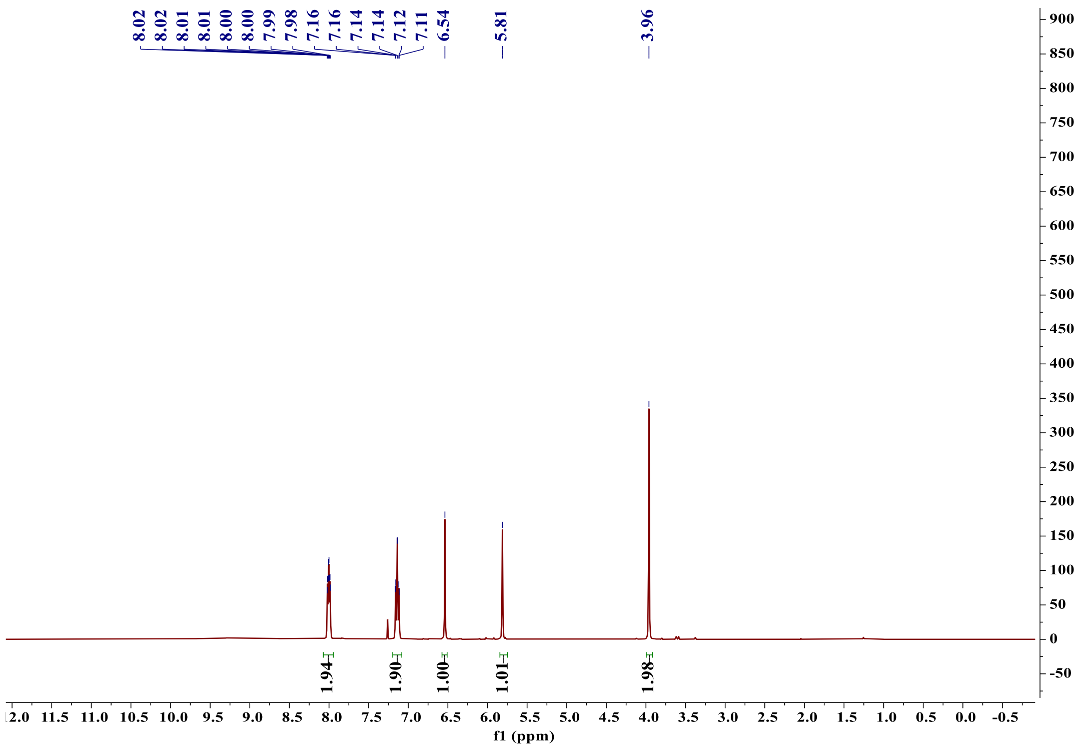

^{1}H NMR (400 MHz, $CDCl_3$) spectrum of **1i**

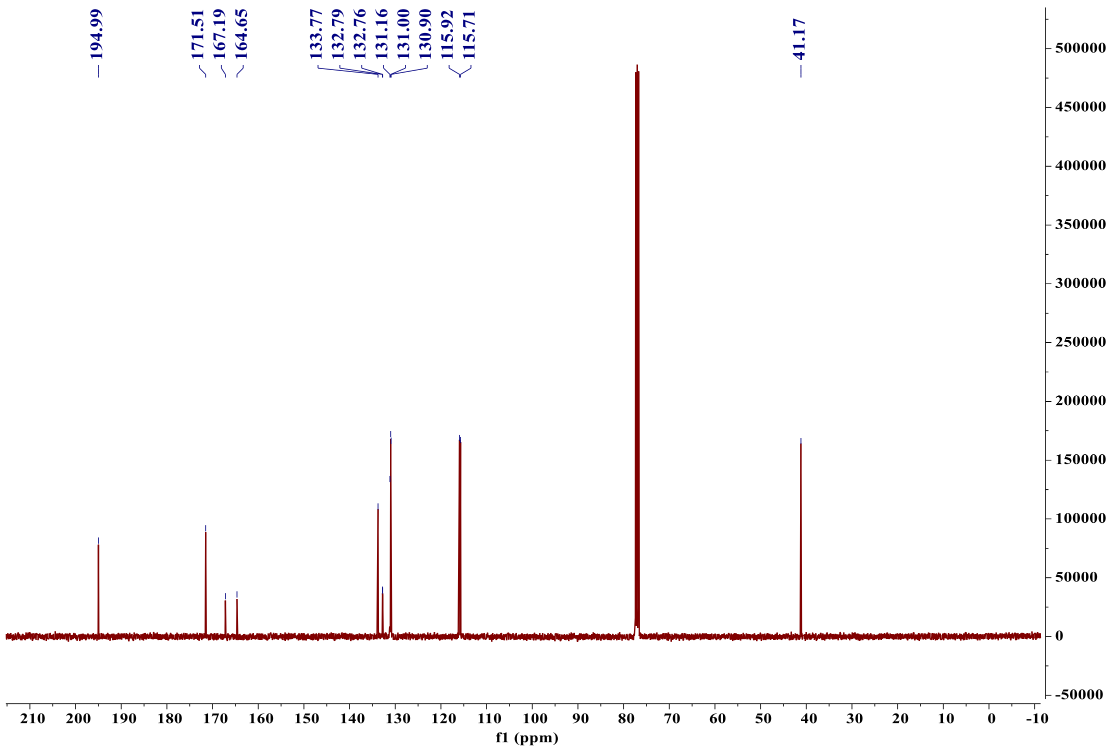

^{13}C NMR (101 MHz, $CDCl_3$) spectrum of **1i**

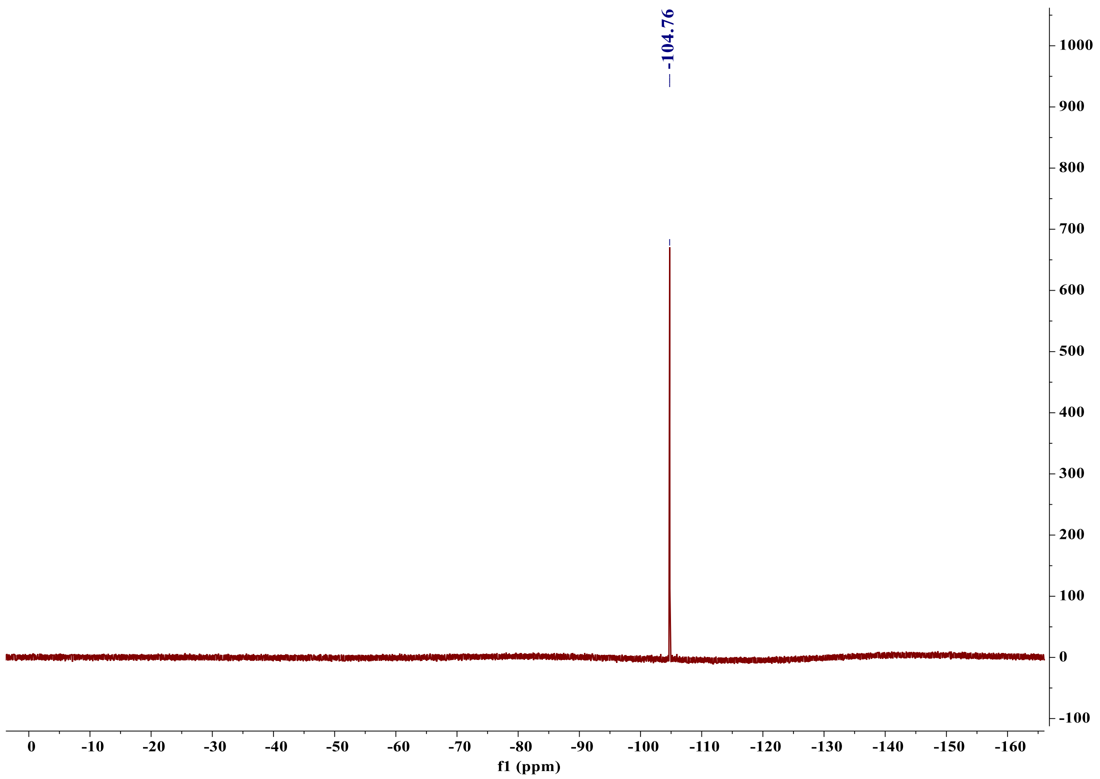

^{19}F NMR (377 MHz, $CDCl_3$) spectrum of **1i**

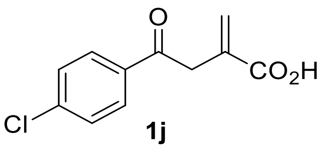

1j

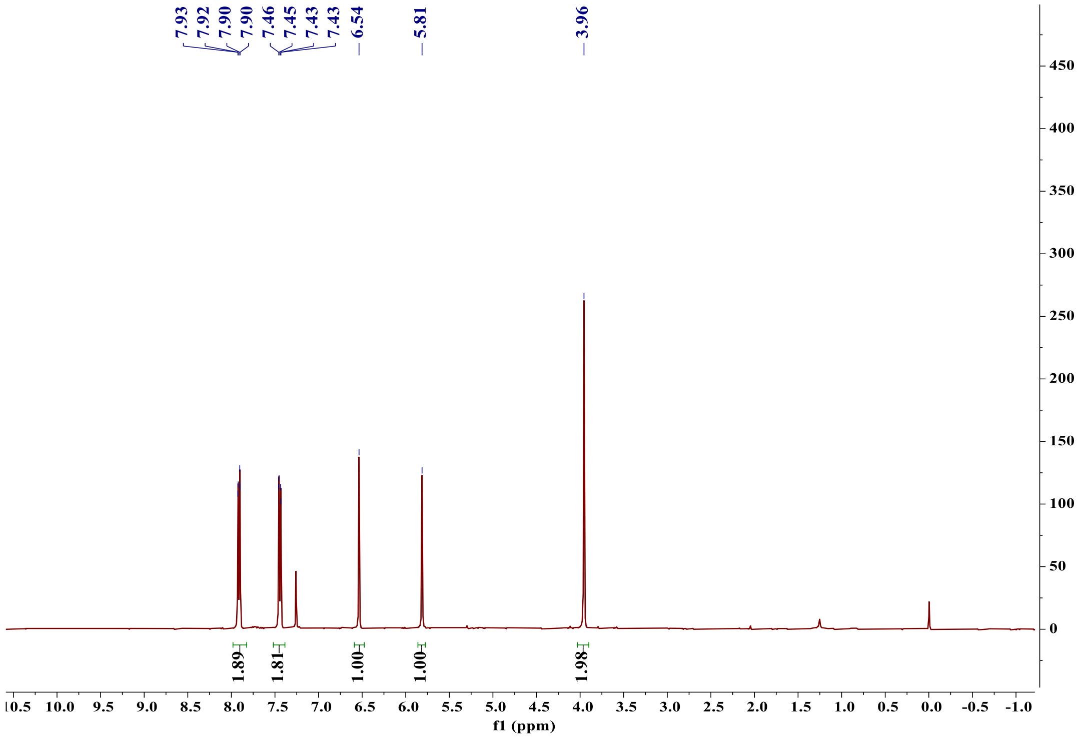

^{1}H NMR (400 MHz, $CDCl_3$) spectrum of **1j**

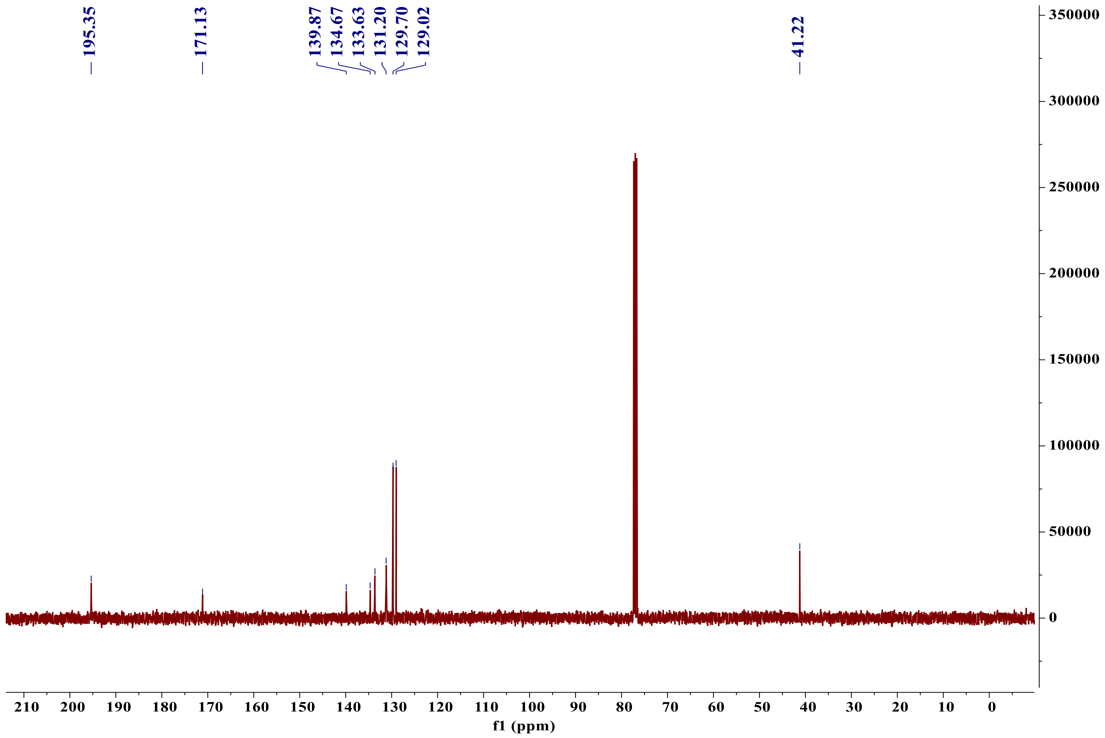

^{13}C NMR (101 MHz, $CDCl_3$) spectrum of **1j**

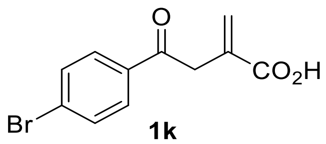

1k

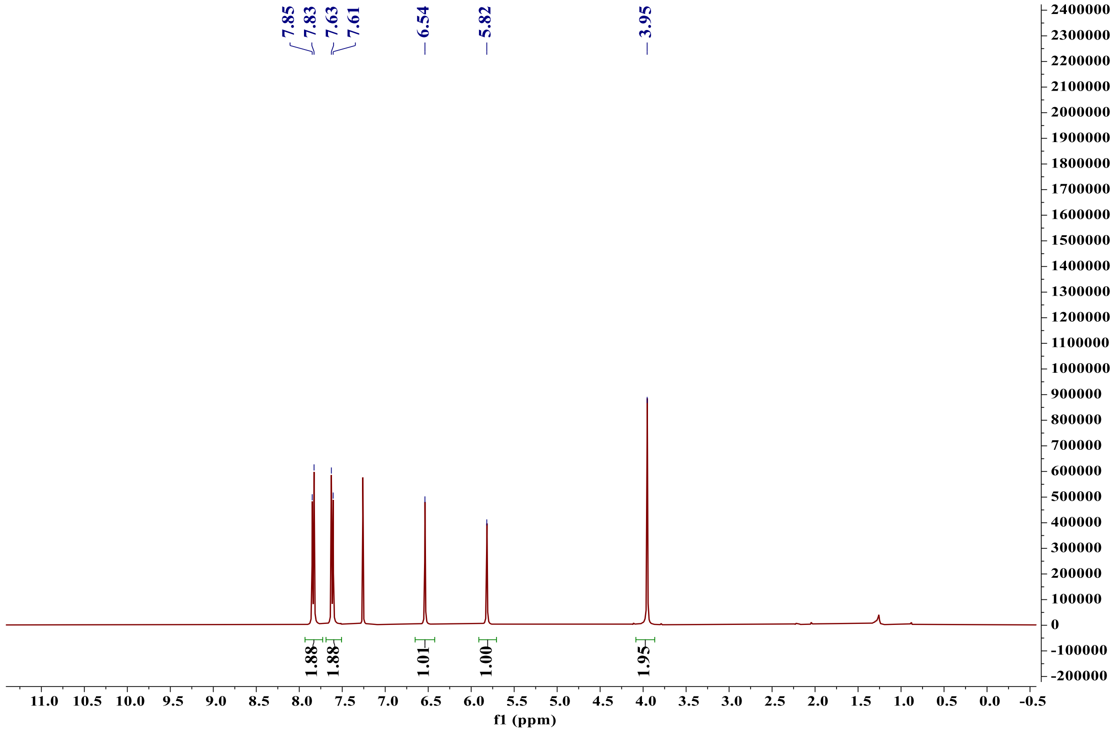

^{1}H NMR (400 MHz, $CDCl_3$) spectrum of **1k**

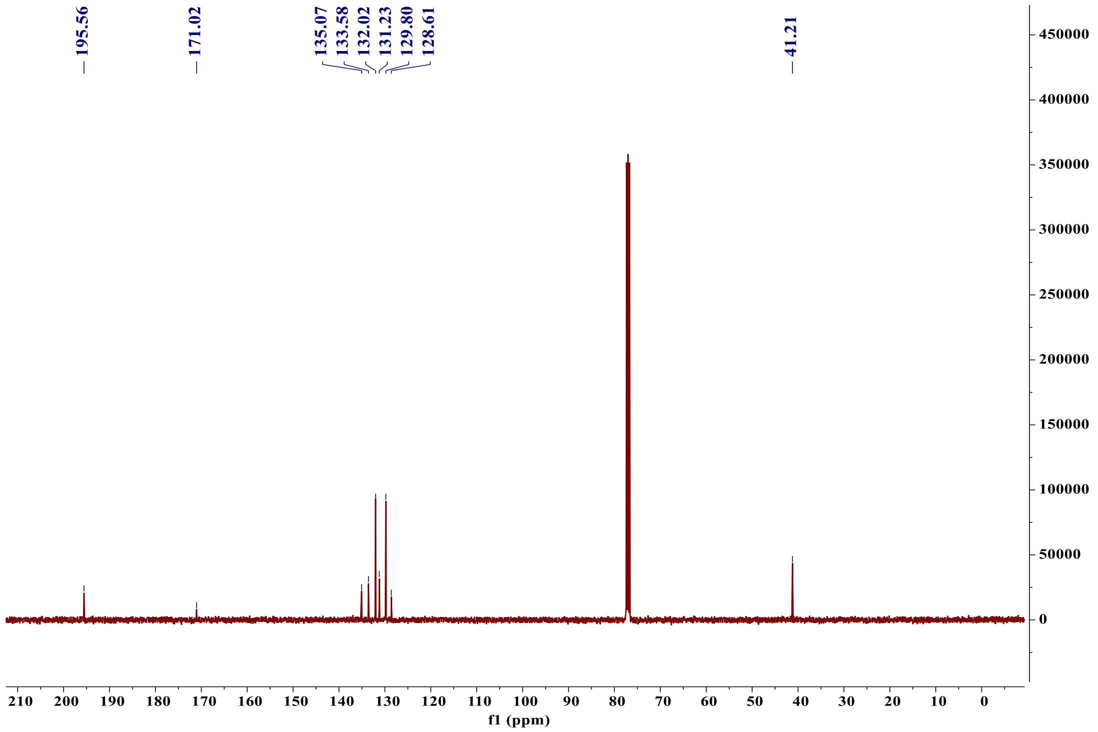

^{13}C NMR (101 MHz, $CDCl_3$) spectrum of **1k**

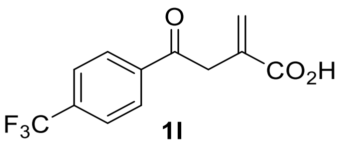

1l

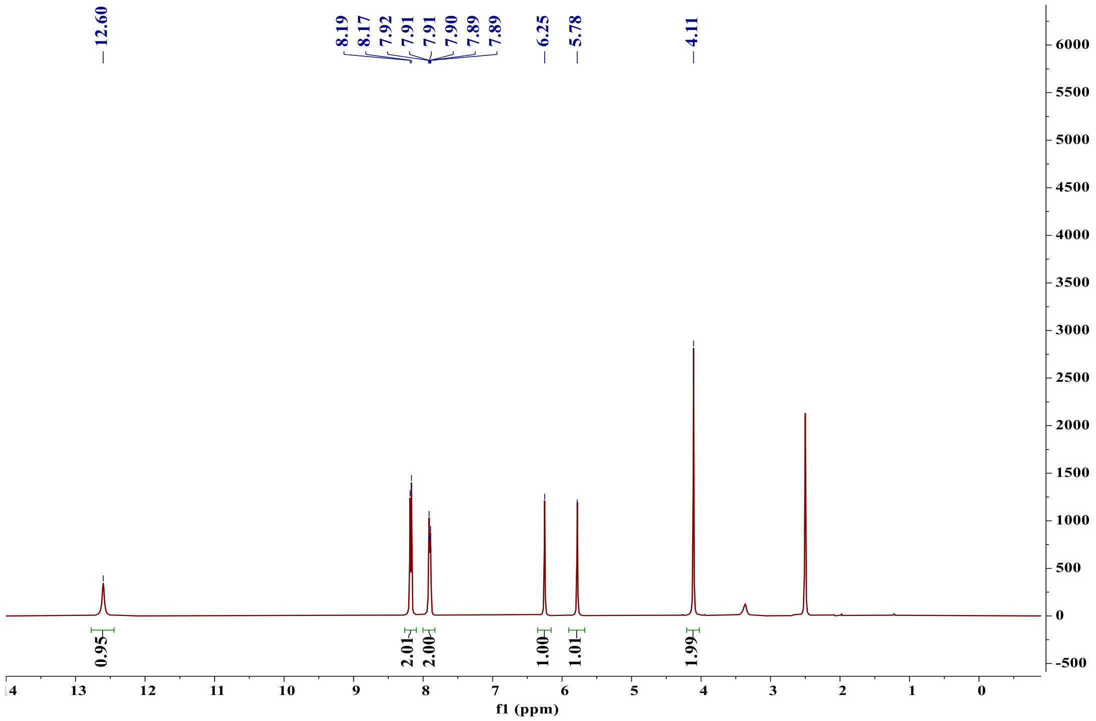

^{1}H NMR (400 MHz, DMSO-d_6) spectrum of **1l**

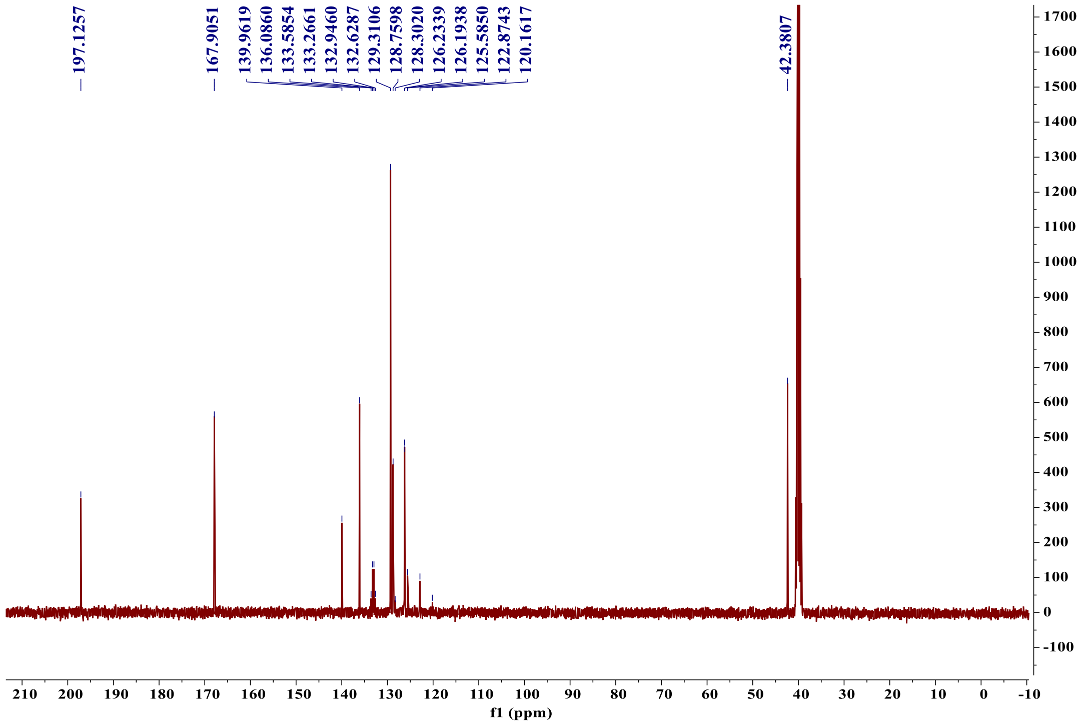

^{13}C NMR (101 MHz, DMSO-d_6) spectrum of **1l**

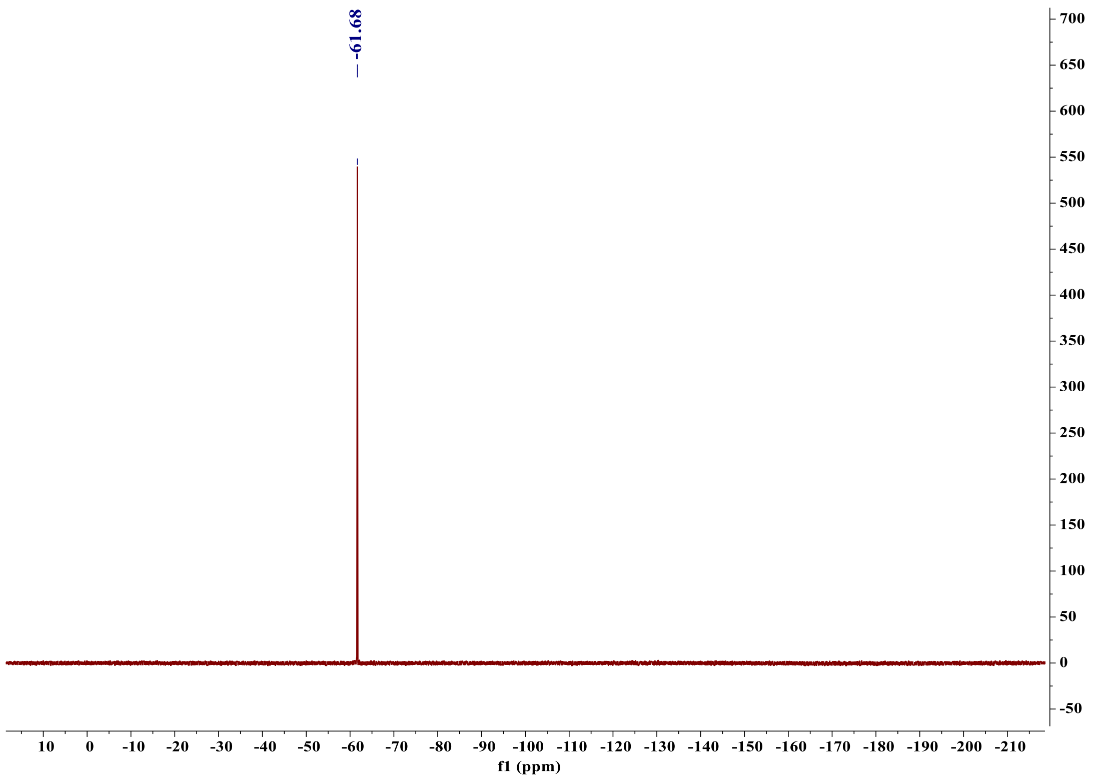

^{19}F NMR (377 MHz, DMSO-d_6) spectrum of **1l**

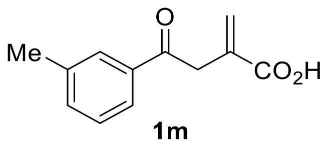

1m

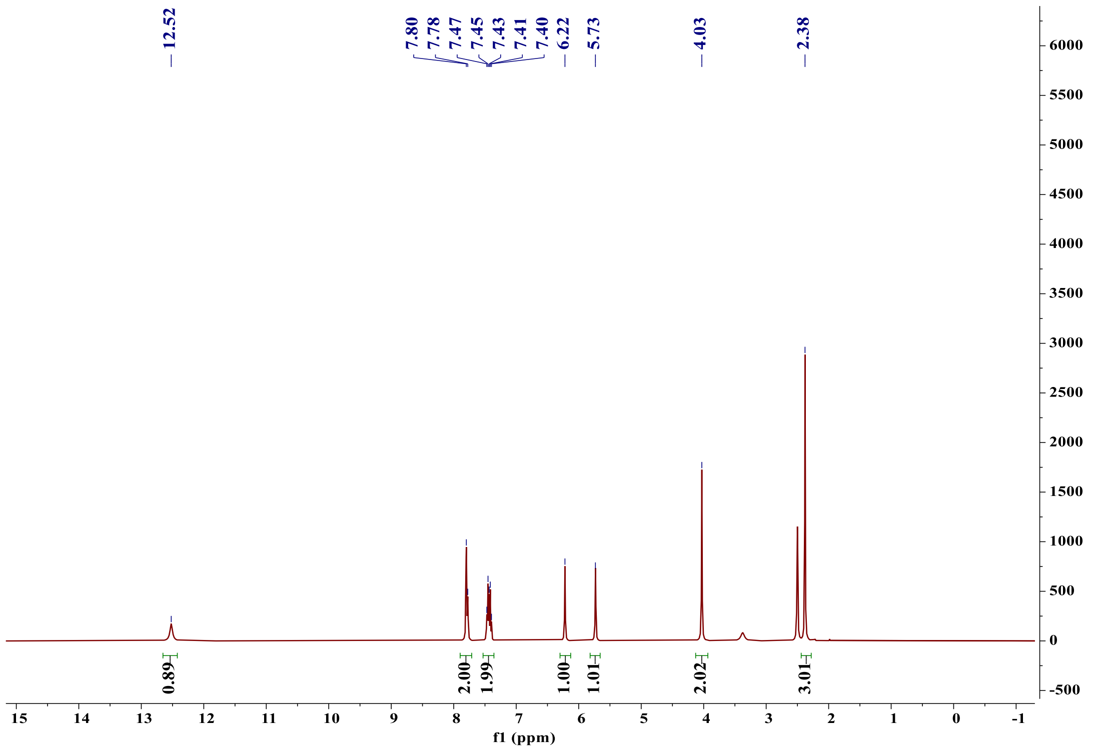

^1H NMR (400 MHz, DMSO-d_6) spectrum of **1m**

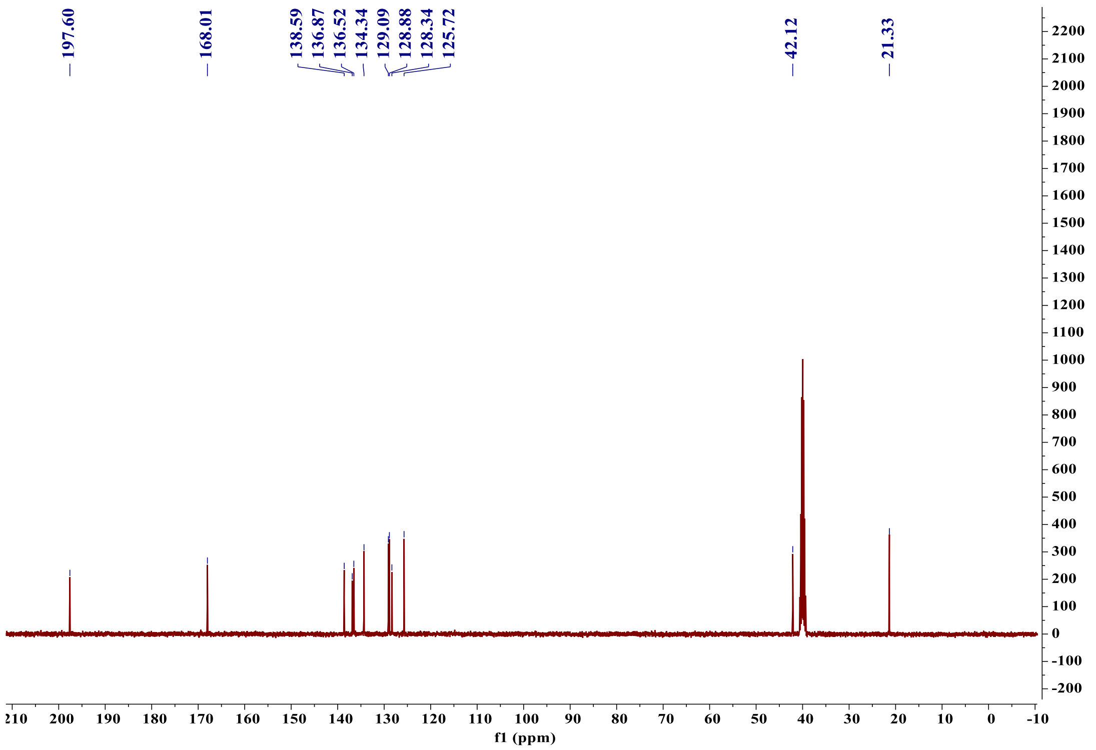

^{13}C NMR (101 MHz, DMSO-d_6) spectrum of **1m**

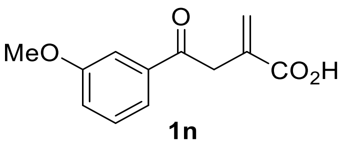

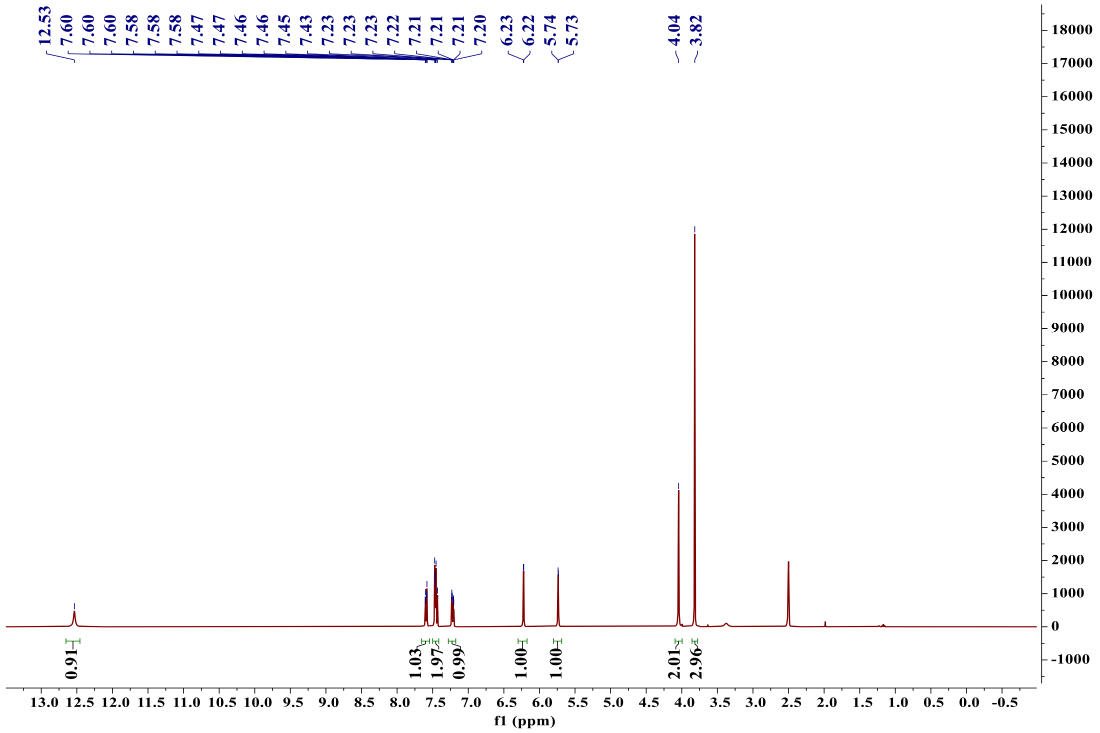

^{1}H NMR (400 MHz, DMSO-d_6) spectrum of **1n**

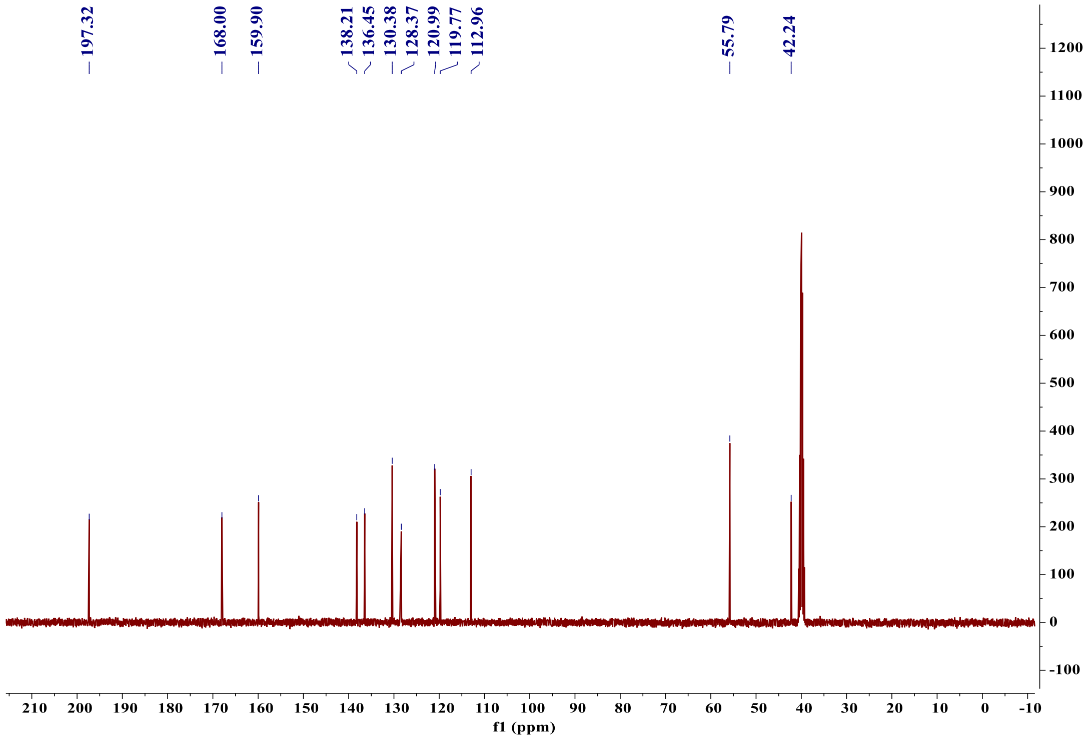

^{13}C NMR (101 MHz, DMSO-d_6) spectrum of **1n**

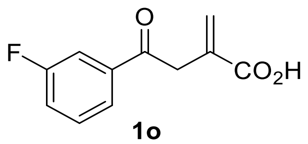

1o

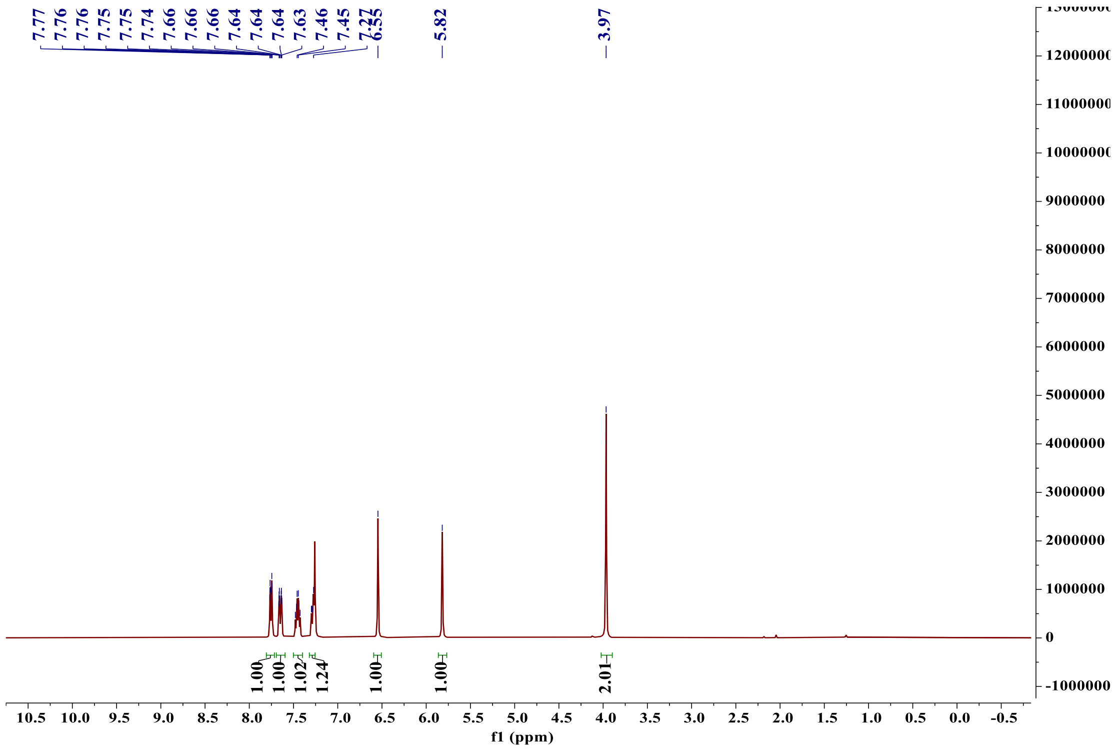

^{1}H NMR (400 MHz, $CDCl_3$) spectrum of **1o**

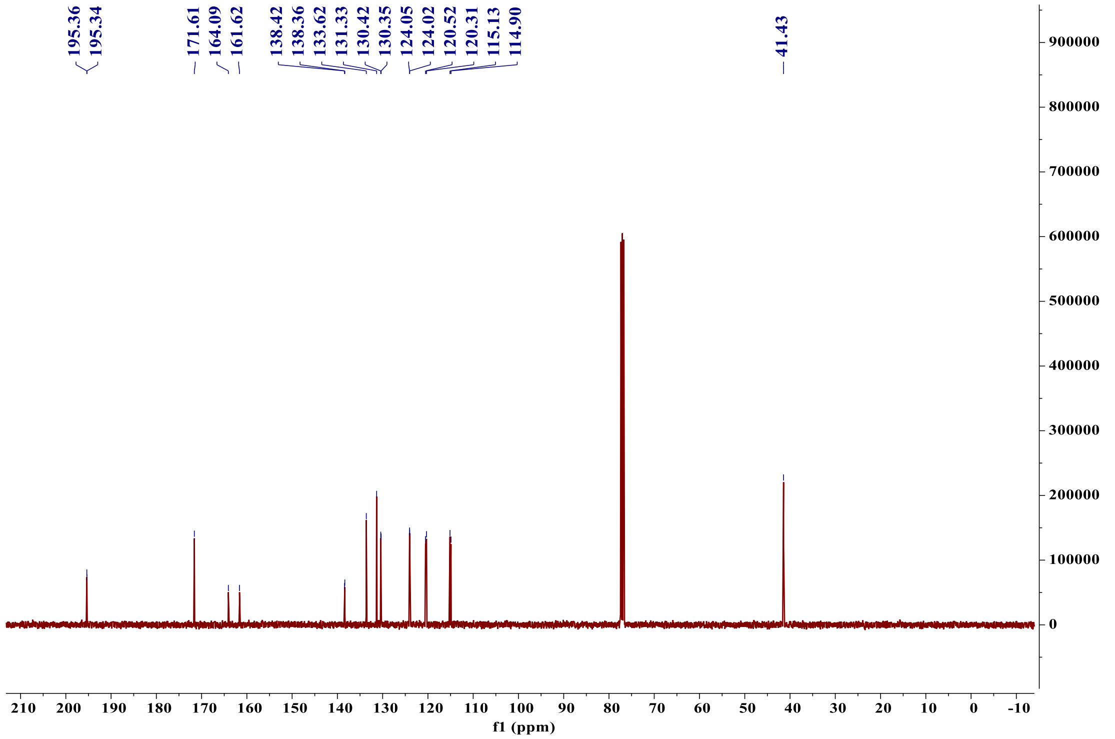

^{13}C NMR (101 MHz, $CDCl_3$) spectrum of **1o**

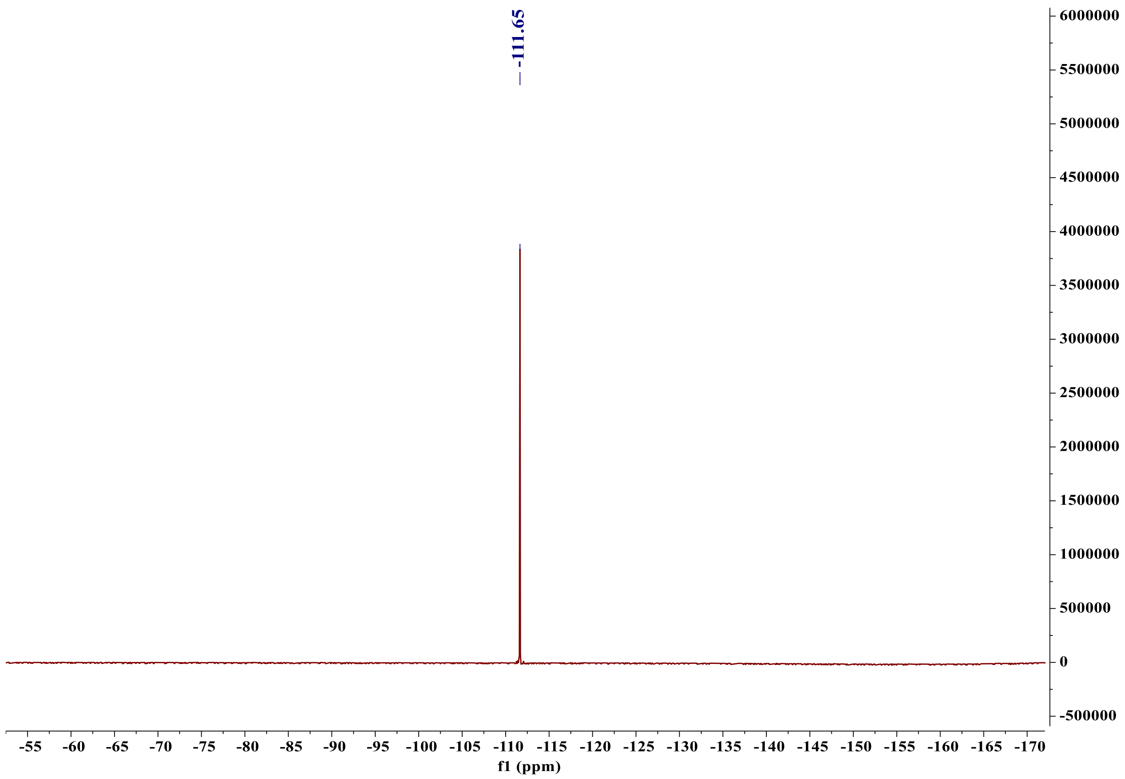

^{19}F NMR (377 MHz, $CDCl_3$) spectrum of **1o**

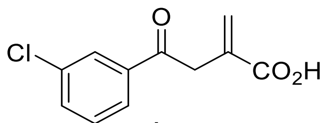

1p

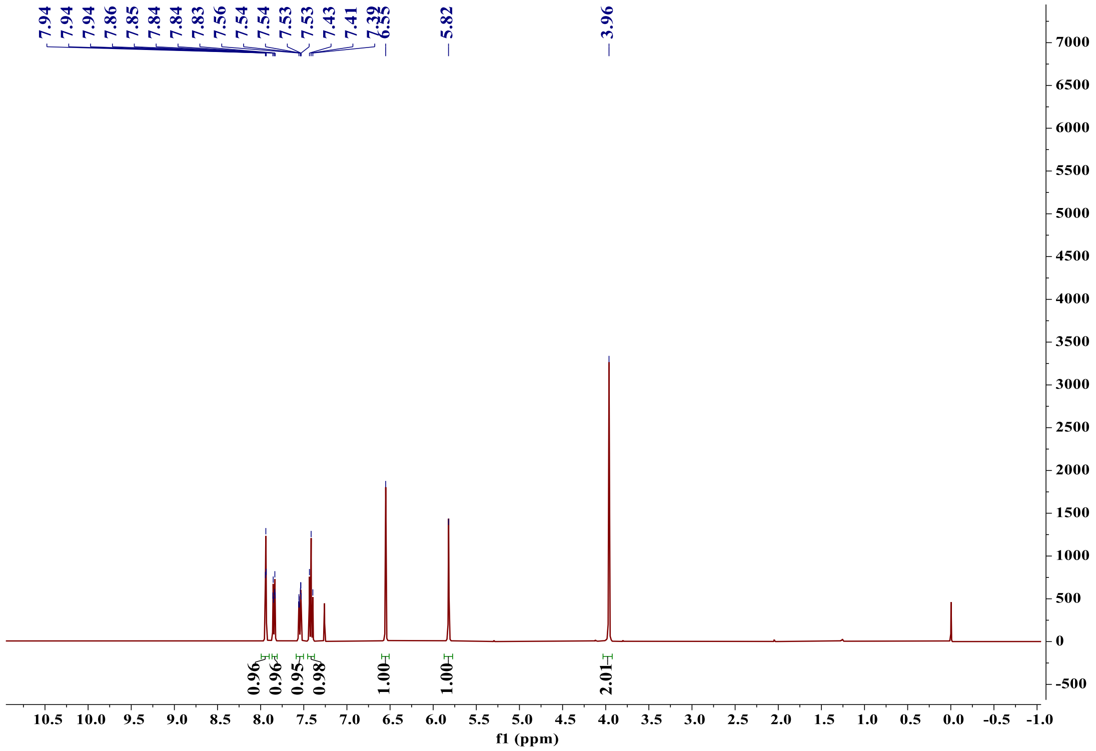

^{1}H NMR (400 MHz, $CDCl_3$) spectrum of **1p**

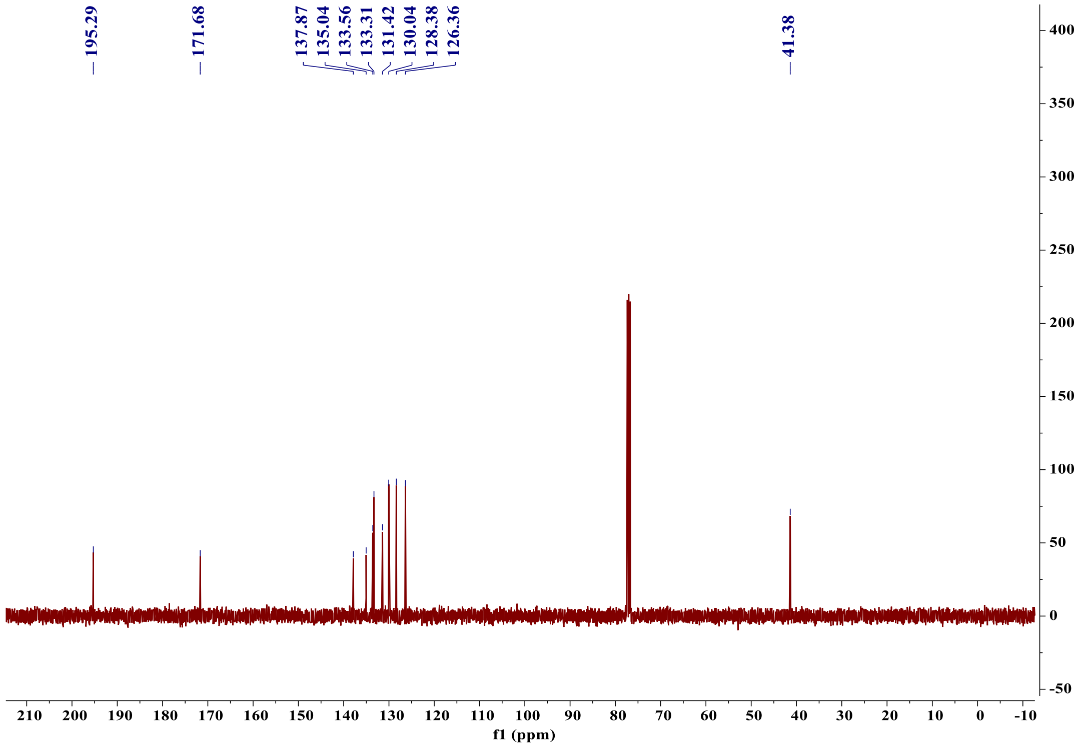

^{13}C NMR (101 MHz, $CDCl_3$) spectrum of **1p**

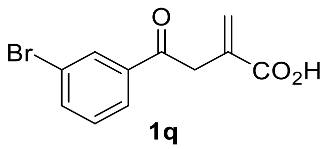

1q

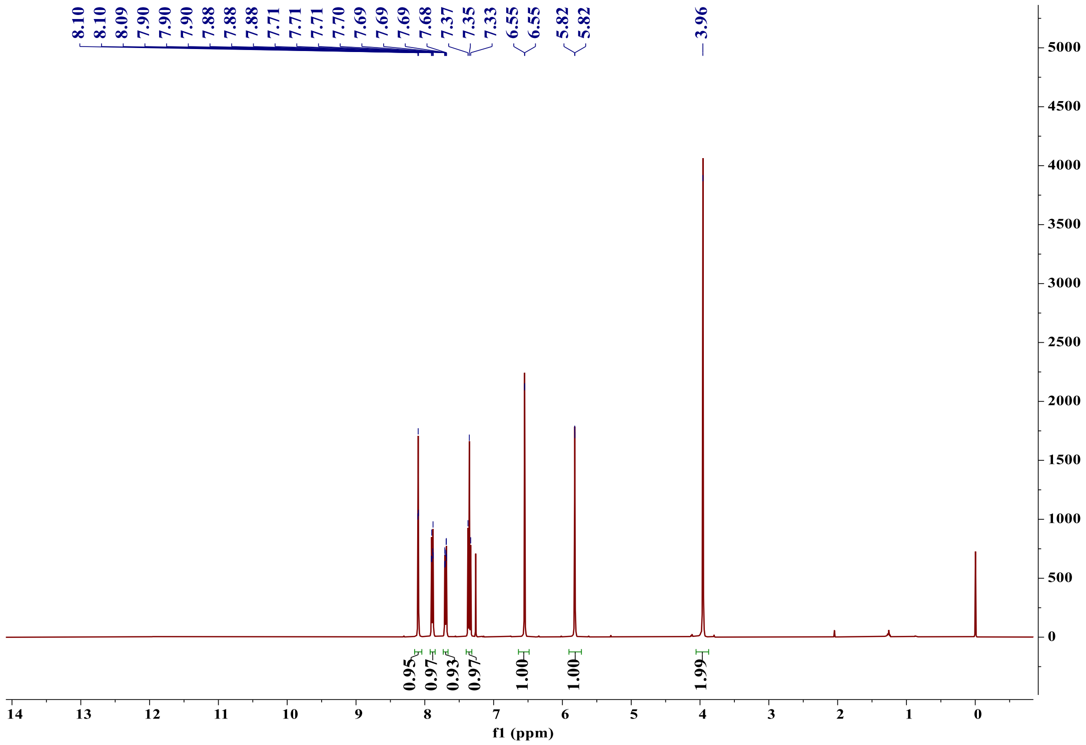

^{1}H NMR (400 MHz, $CDCl_3$) spectrum of **1q**

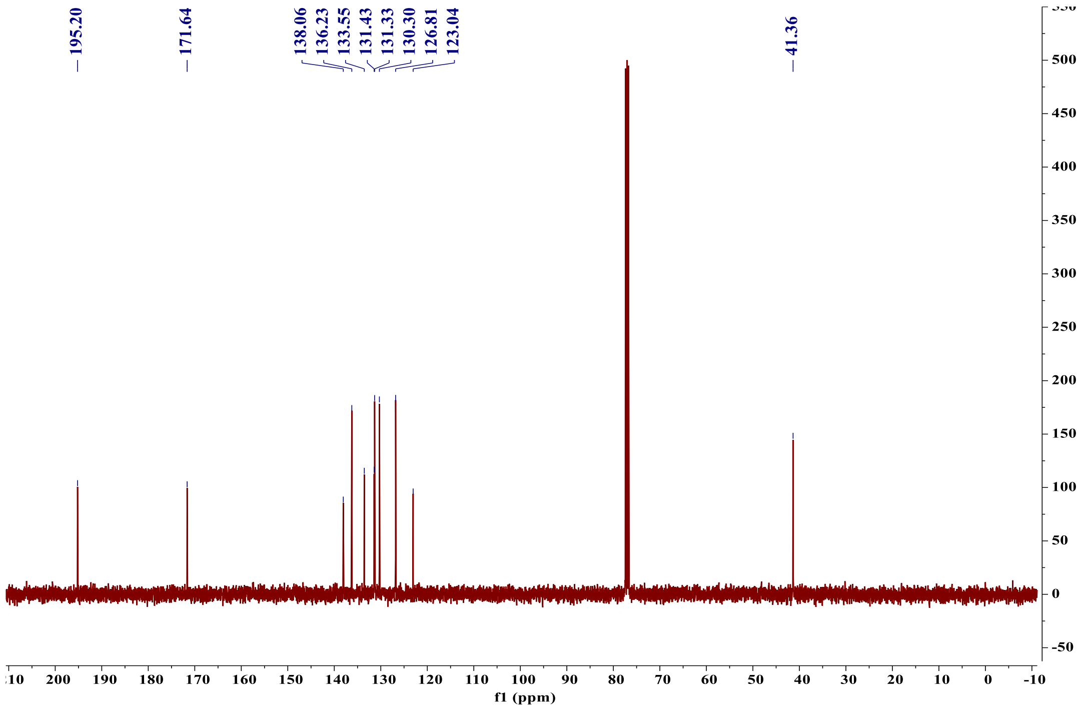

^{13}C NMR (101 MHz, $CDCl_3$) spectrum of **1q**

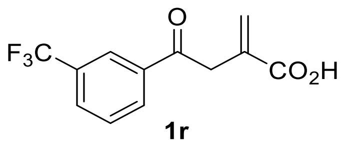

1r

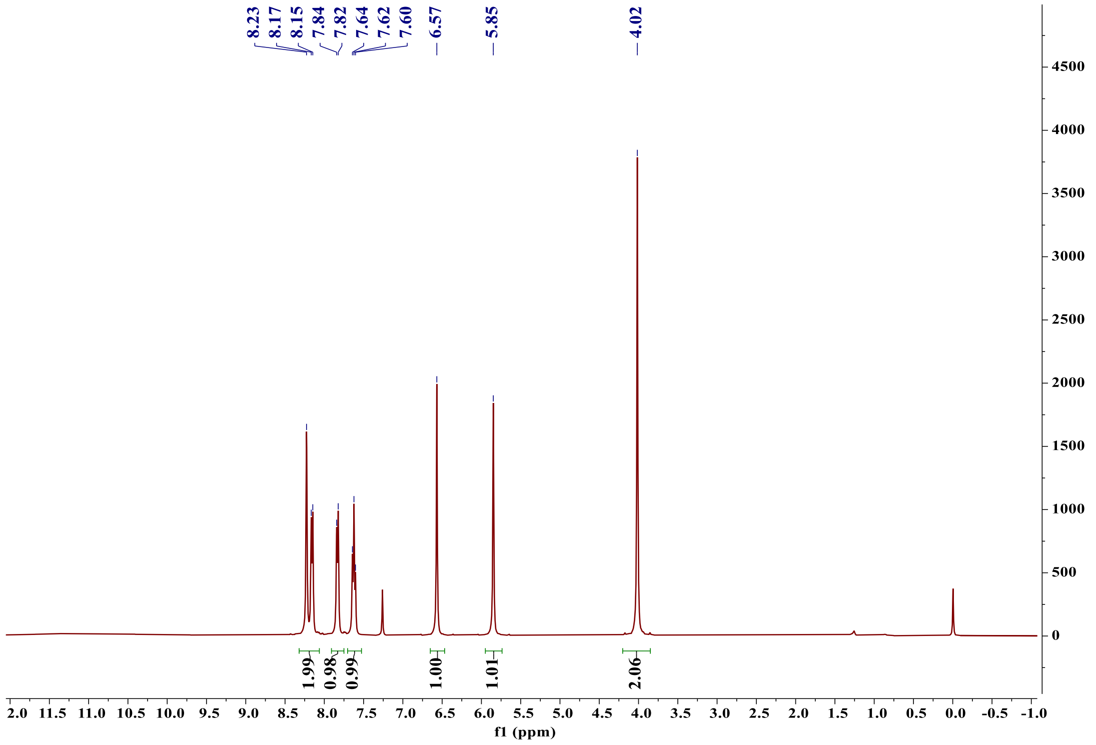

^{1}H NMR (400 MHz, $CDCl_3$) spectrum of **1r**

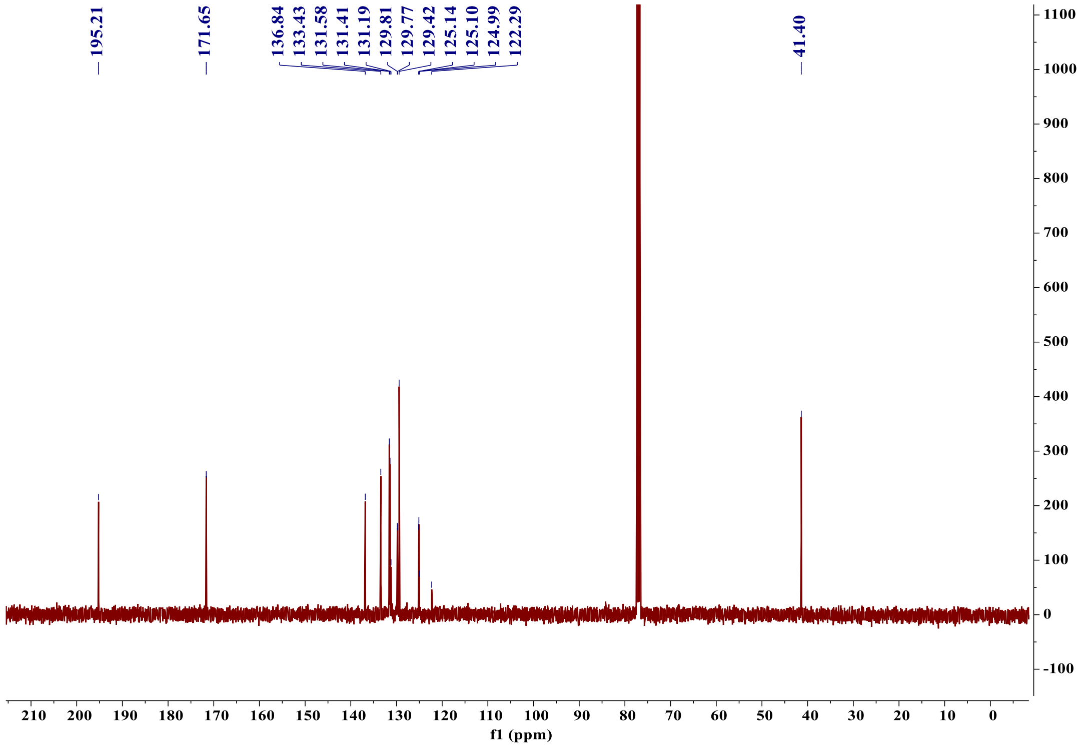

^{13}C NMR (101 MHz, $CDCl_3$) spectrum of **1r**

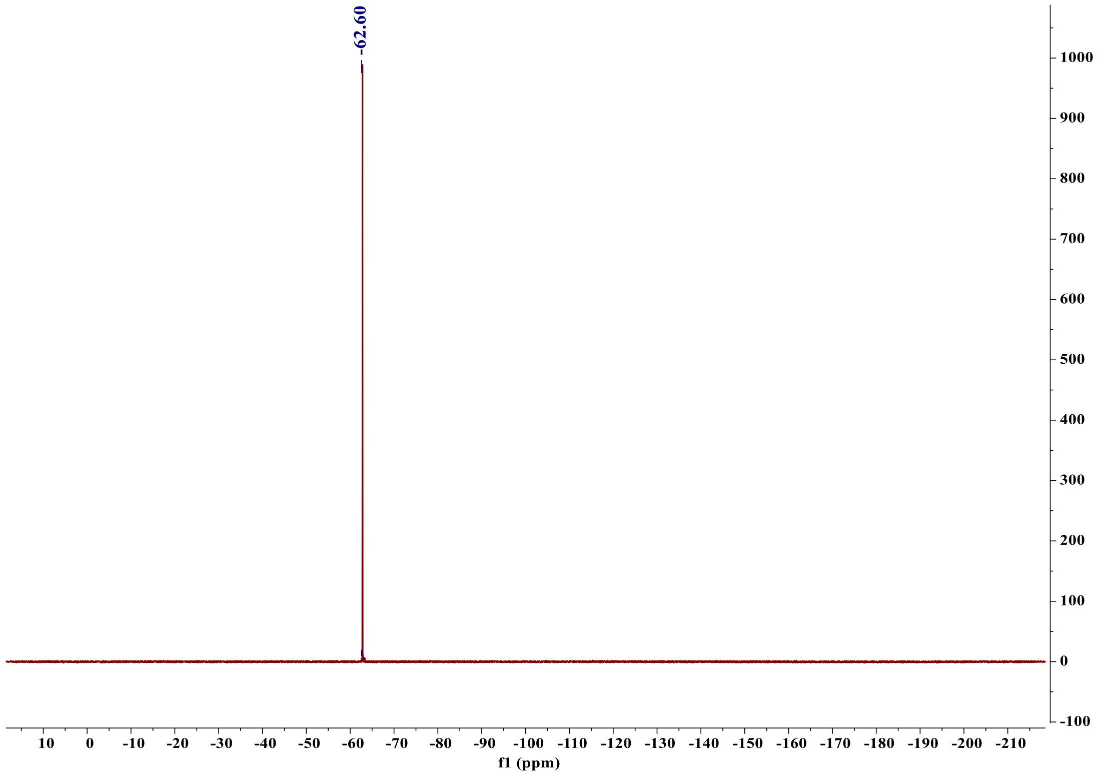

^{19}F NMR (377 MHz, $CDCl_3$) spectrum of **1r**

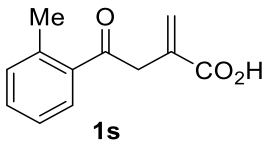

1s

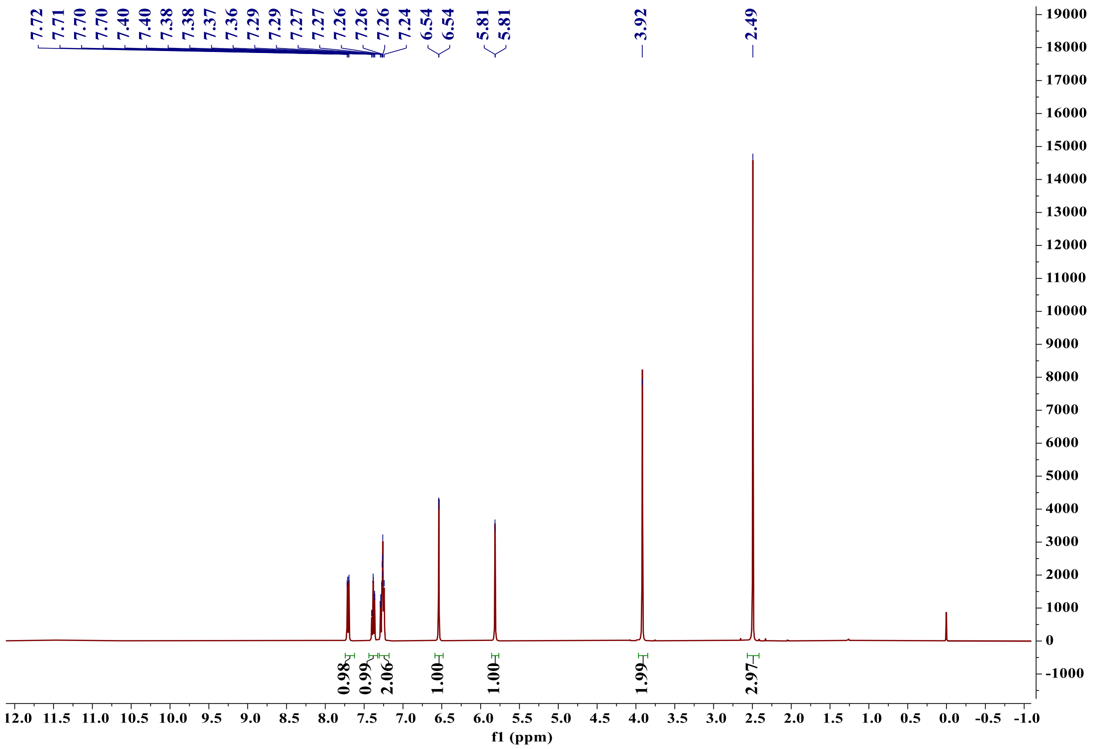

^{1}H NMR (400 MHz, $CDCl_3$) spectrum of **1s**

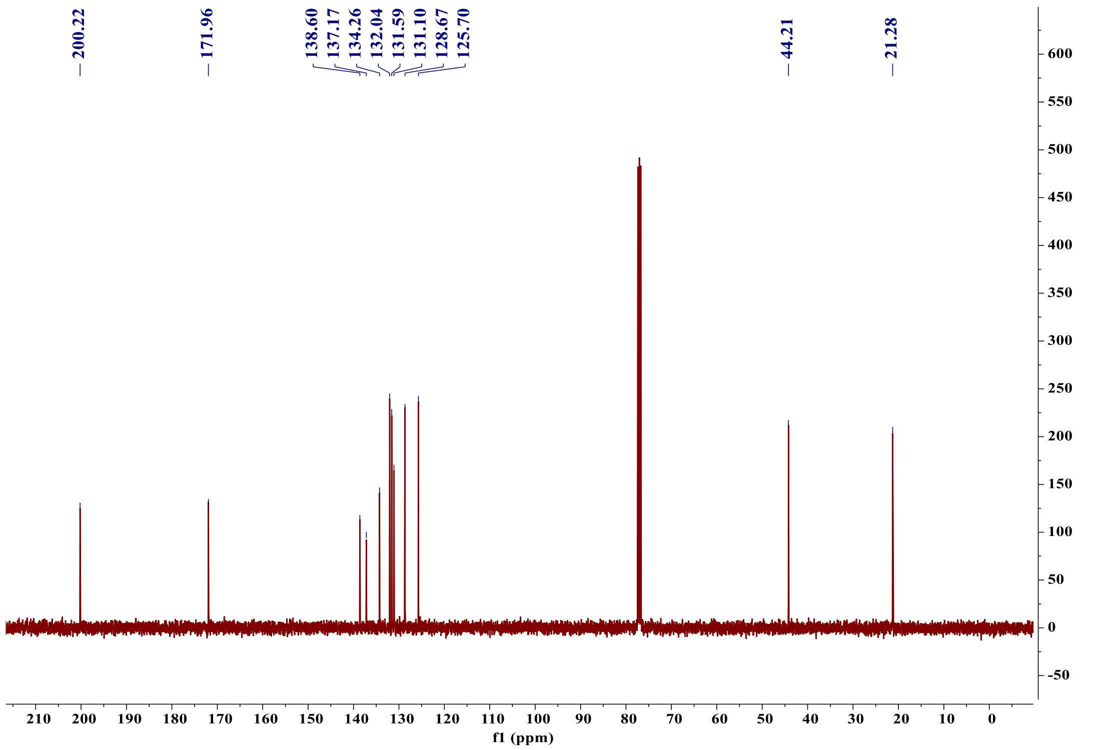

^{13}C NMR (101 MHz, $CDCl_3$) spectrum of **1s**

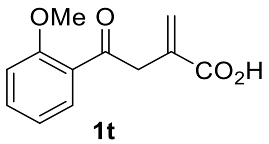

1t

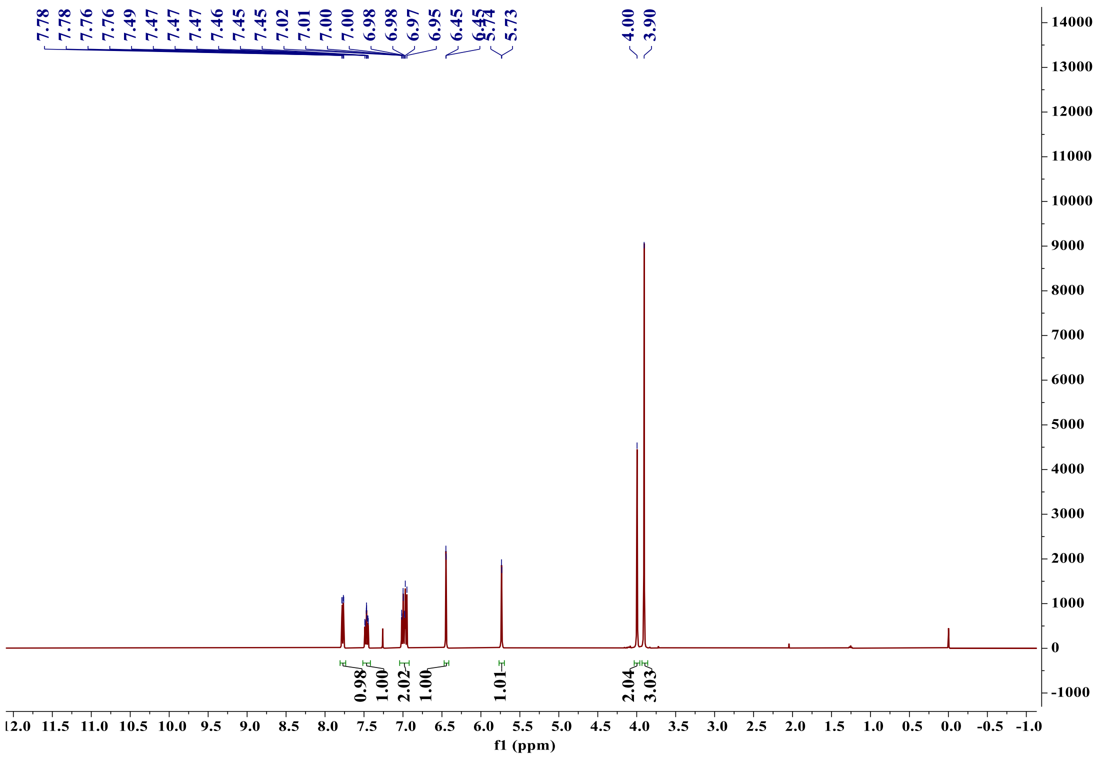

^{1}H NMR (400 MHz, $CDCl_3$) spectrum of **1t**

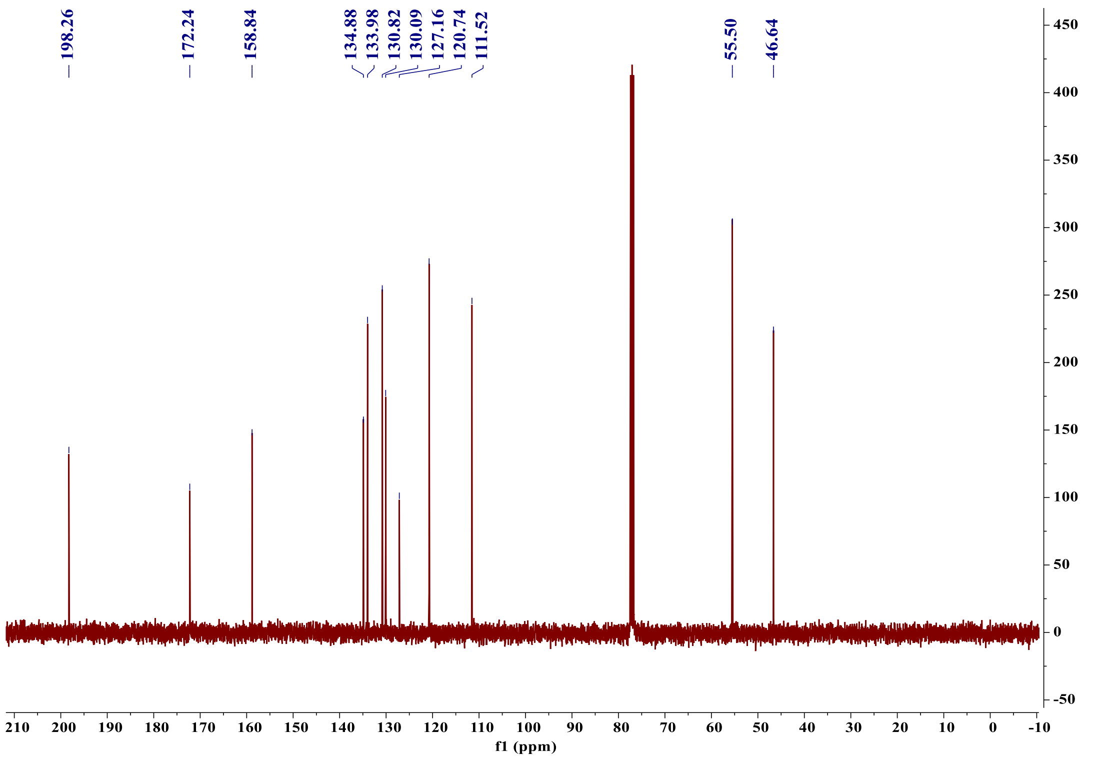

^{13}C NMR (101 MHz, $CDCl_3$) spectrum of **1t**

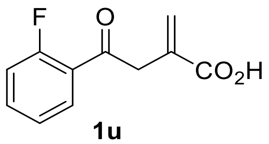

1u

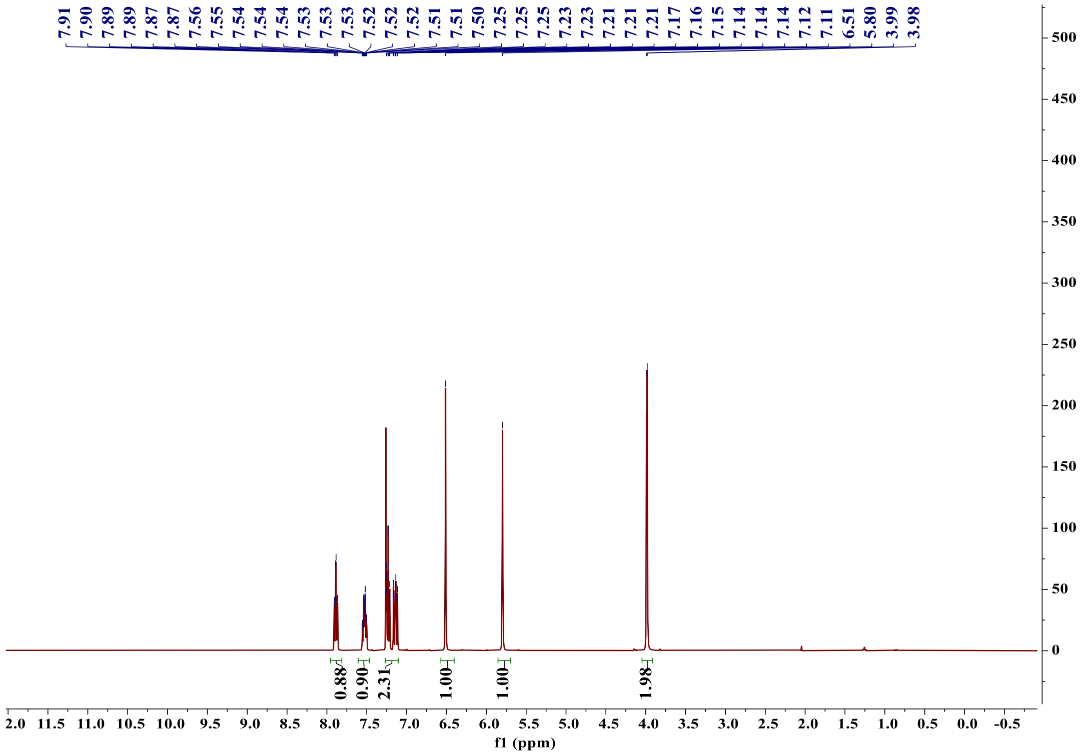

^{1}H NMR (400 MHz, $CDCl_3$) spectrum of **1u**

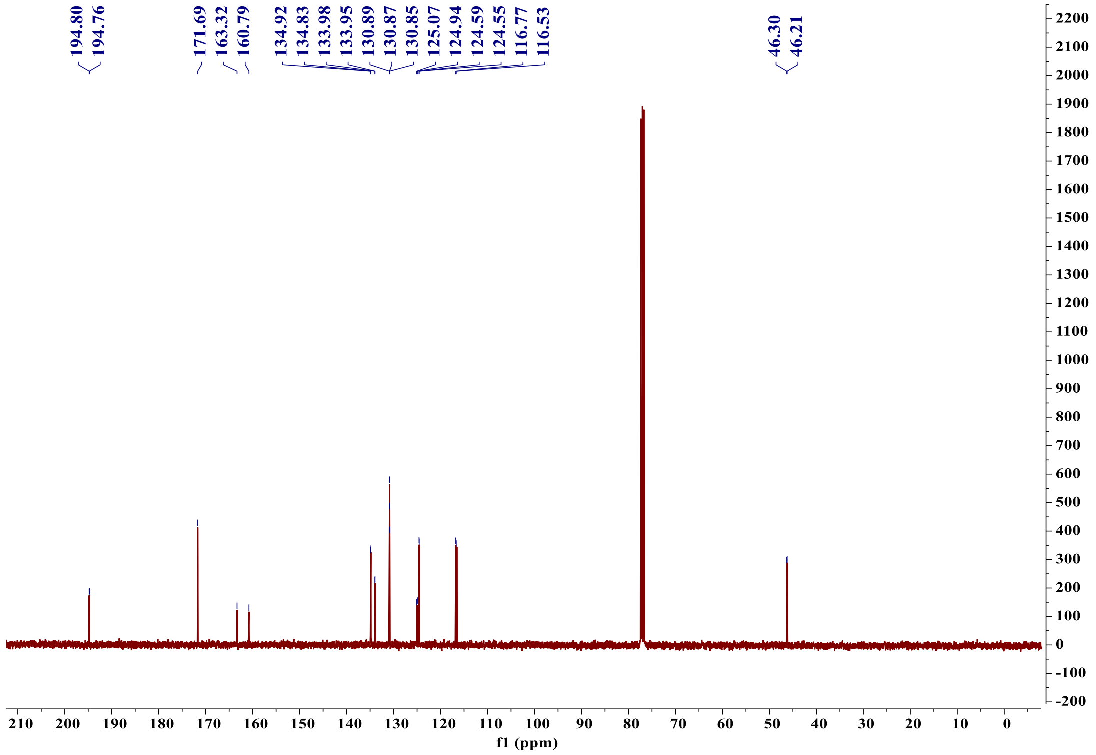

^{13}C NMR (101 MHz, $CDCl_3$) spectrum of **1u**

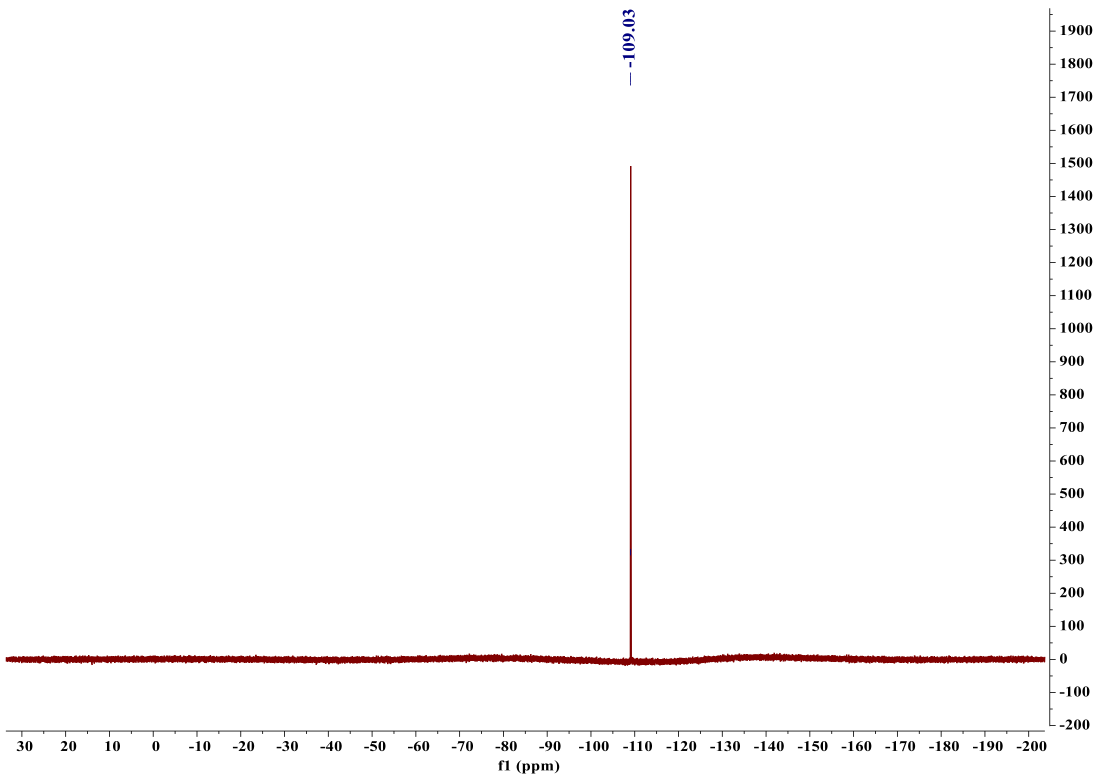

^{19}F NMR (377 MHz, $CDCl_3$) spectrum of **1u**

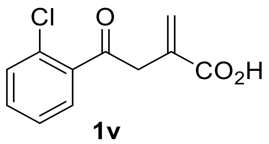

1v

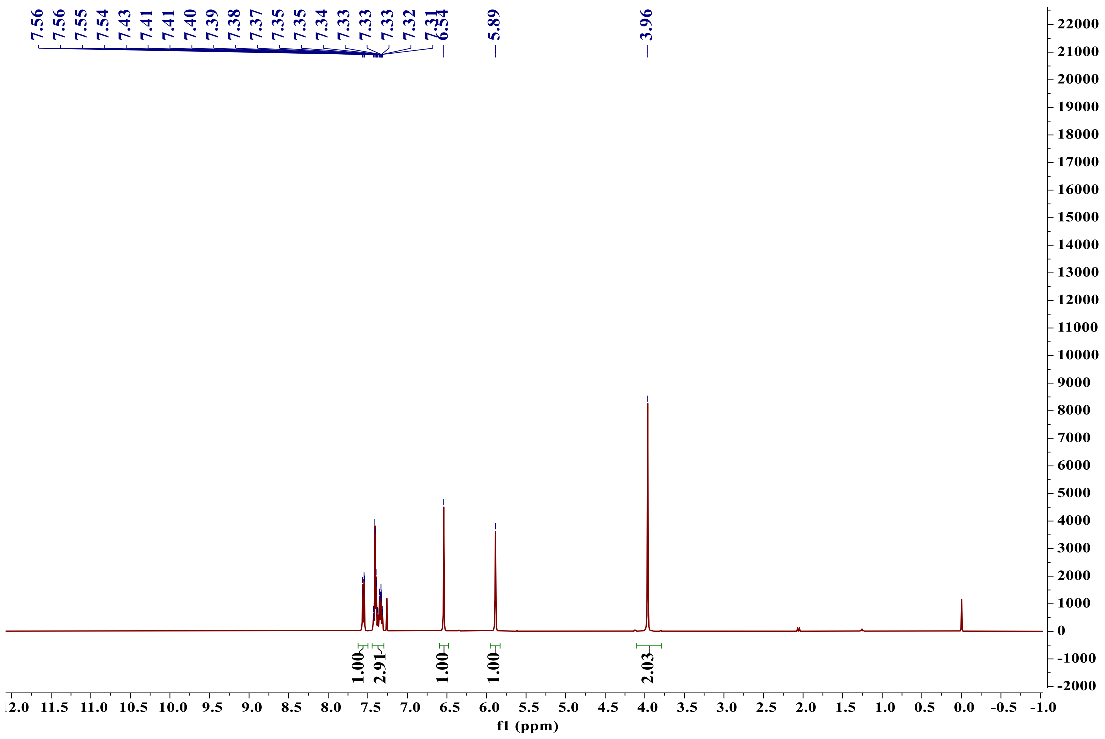

^1H NMR (400 MHz, $CDCl_3$) spectrum of **1v**

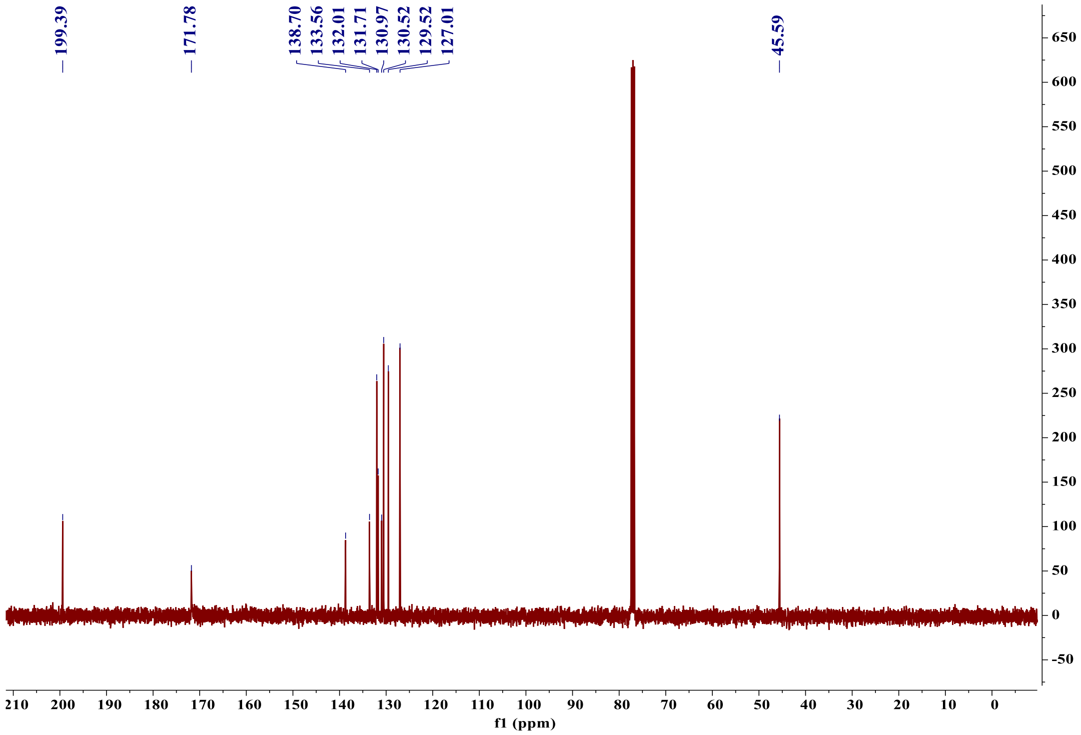

^{13}C NMR (101 MHz, $CDCl_3$) spectrum of **1v**

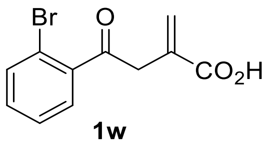

1w

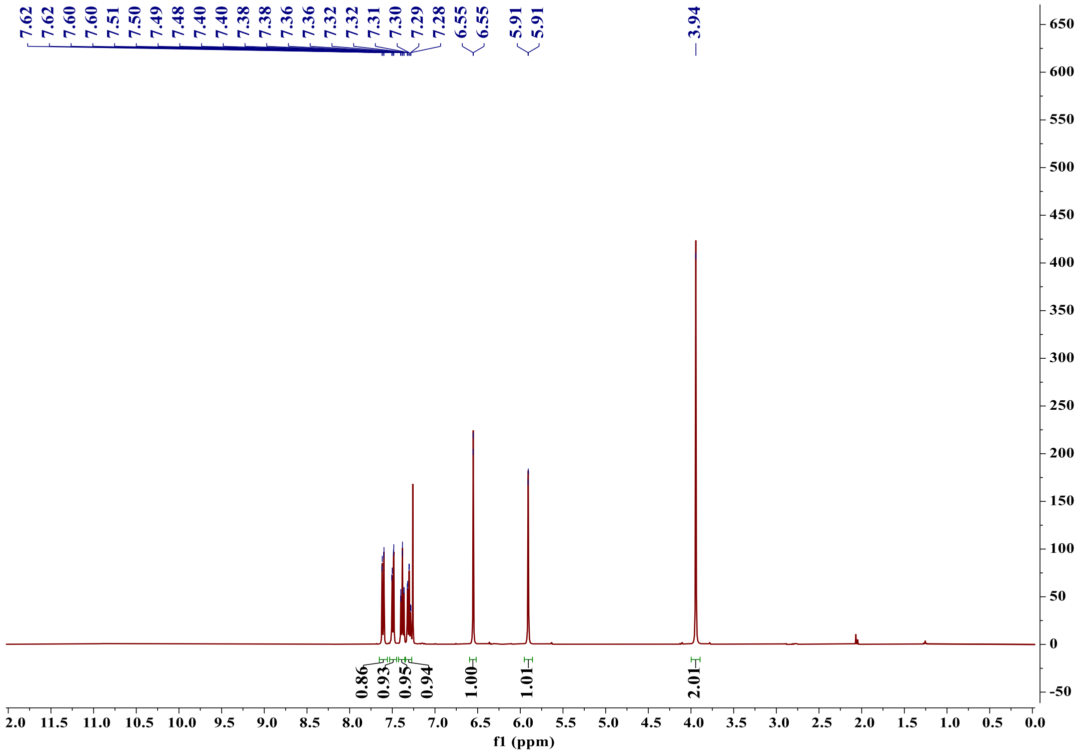

^{1}H NMR (400 MHz, $CDCl_3$) spectrum of **1w**

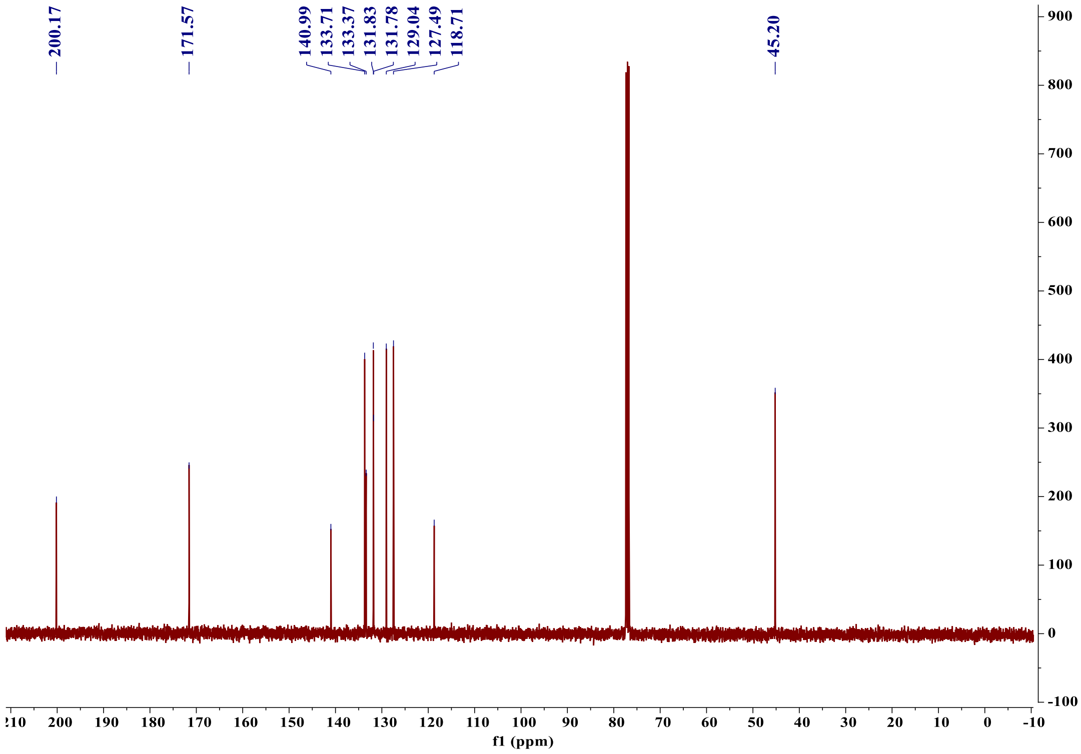

^{13}C NMR (101 MHz, $CDCl_3$) spectrum of **1w**

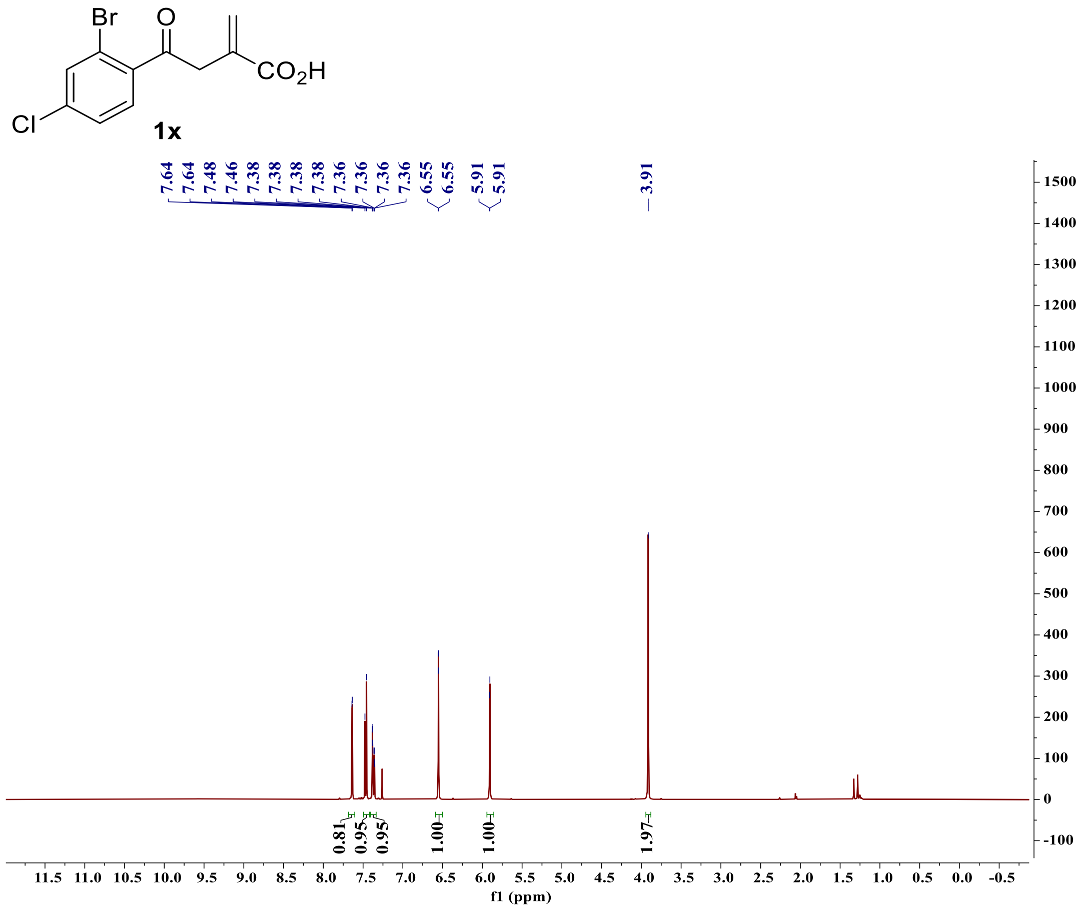

^{1}H NMR (400 MHz, $CDCl_3$) spectrum of **1x**

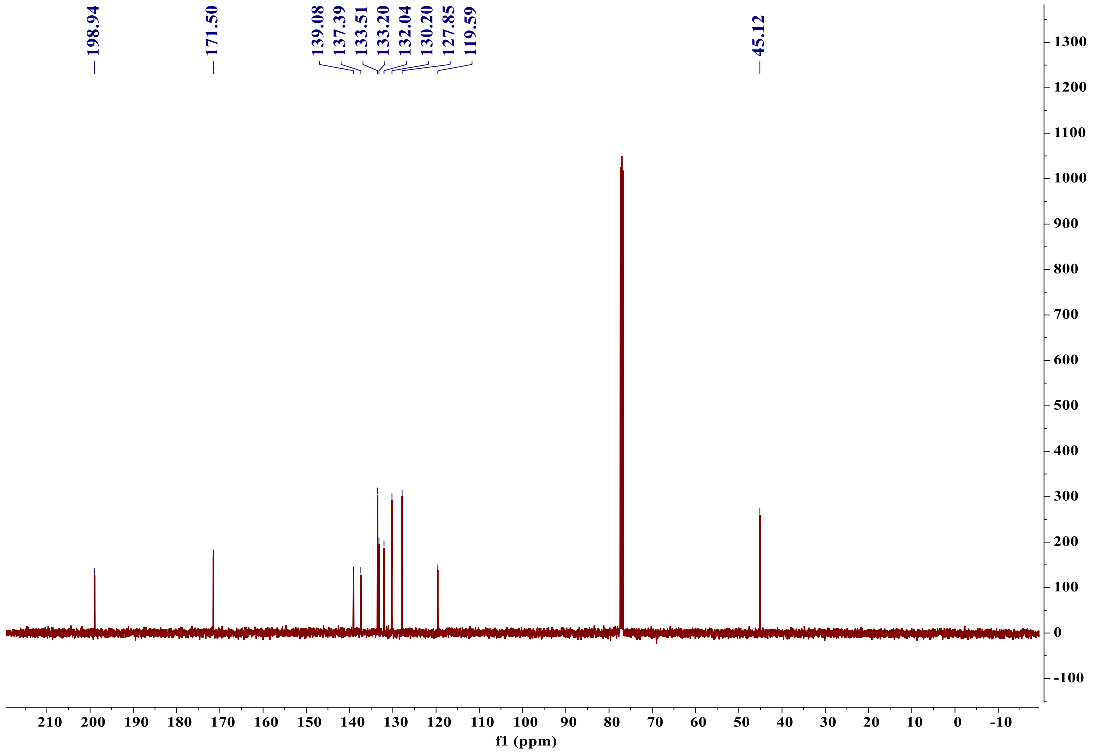

^{13}C NMR (101 MHz, $CDCl_3$) spectrum of **1x**

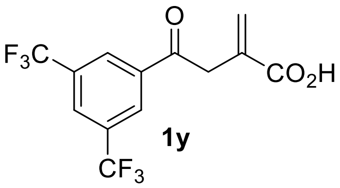

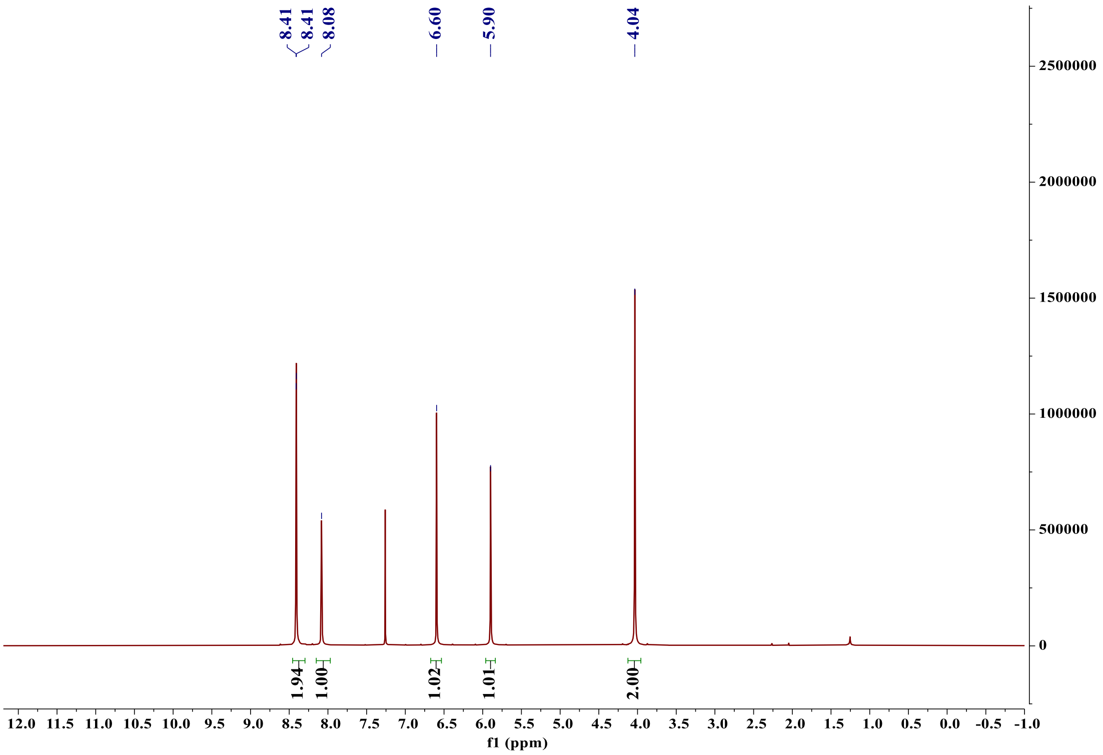

^{1}H NMR (400 MHz, $CDCl_3$) spectrum of **1y**

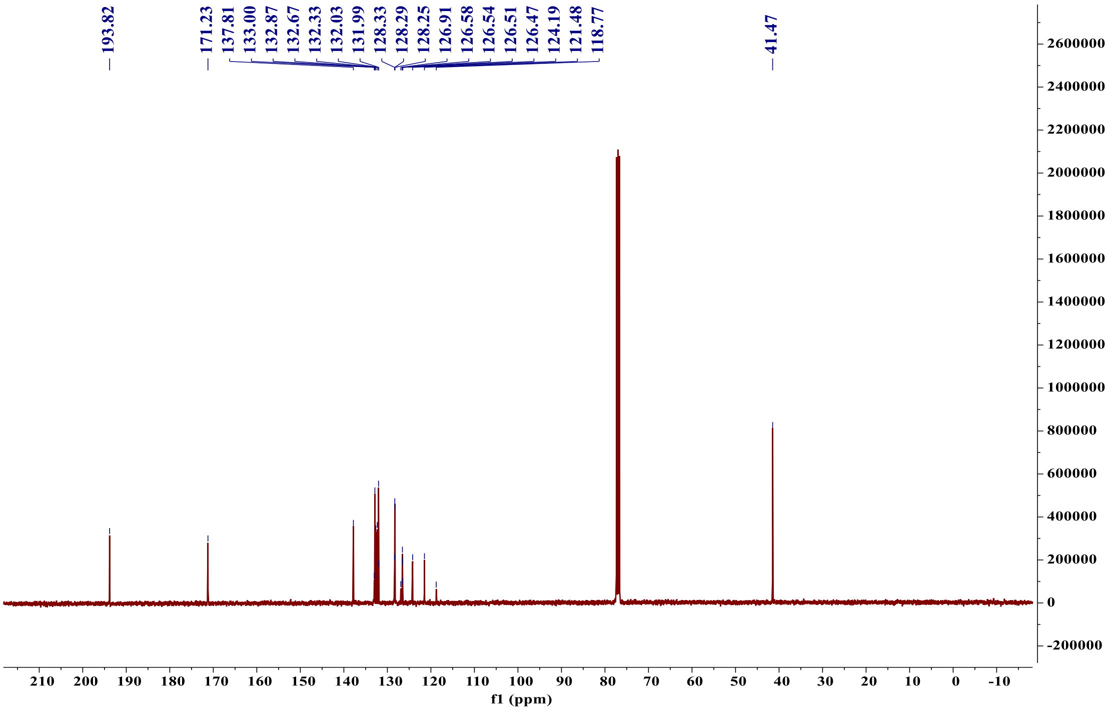

^{13}C NMR (101 MHz, $CDCl_3$) spectrum of **1y**

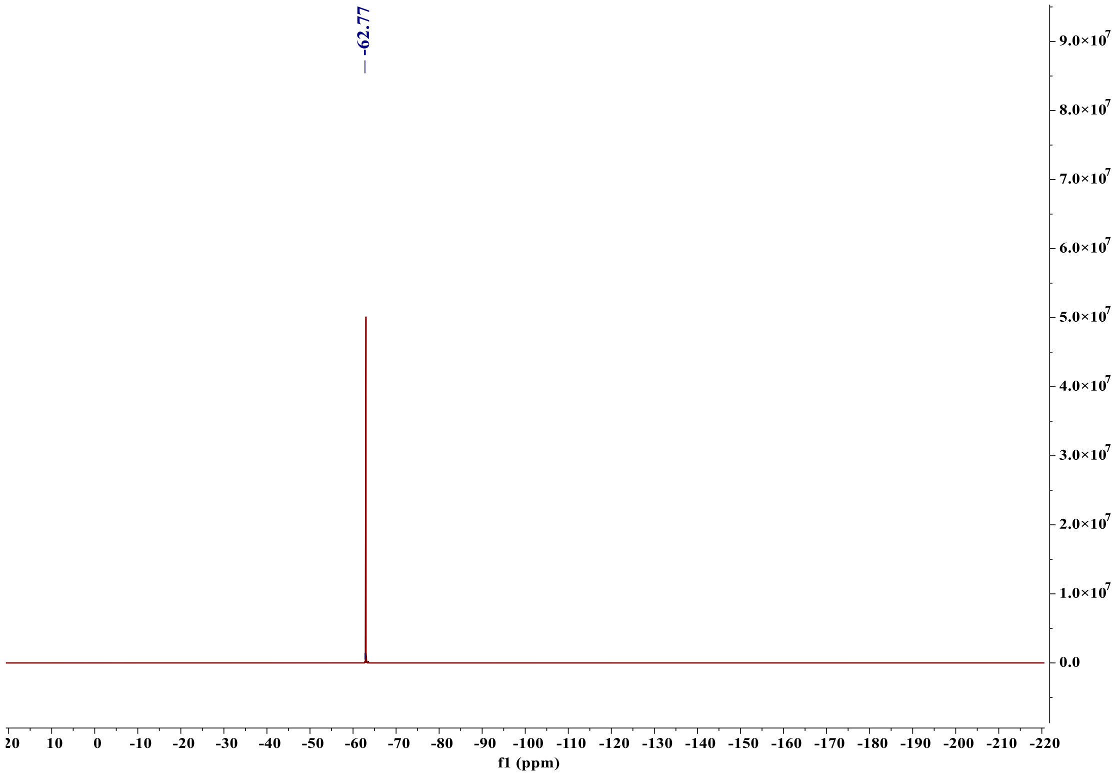

^{19}F NMR (377 MHz, $CDCl_3$) spectrum of **1y**

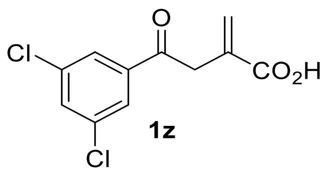

1z

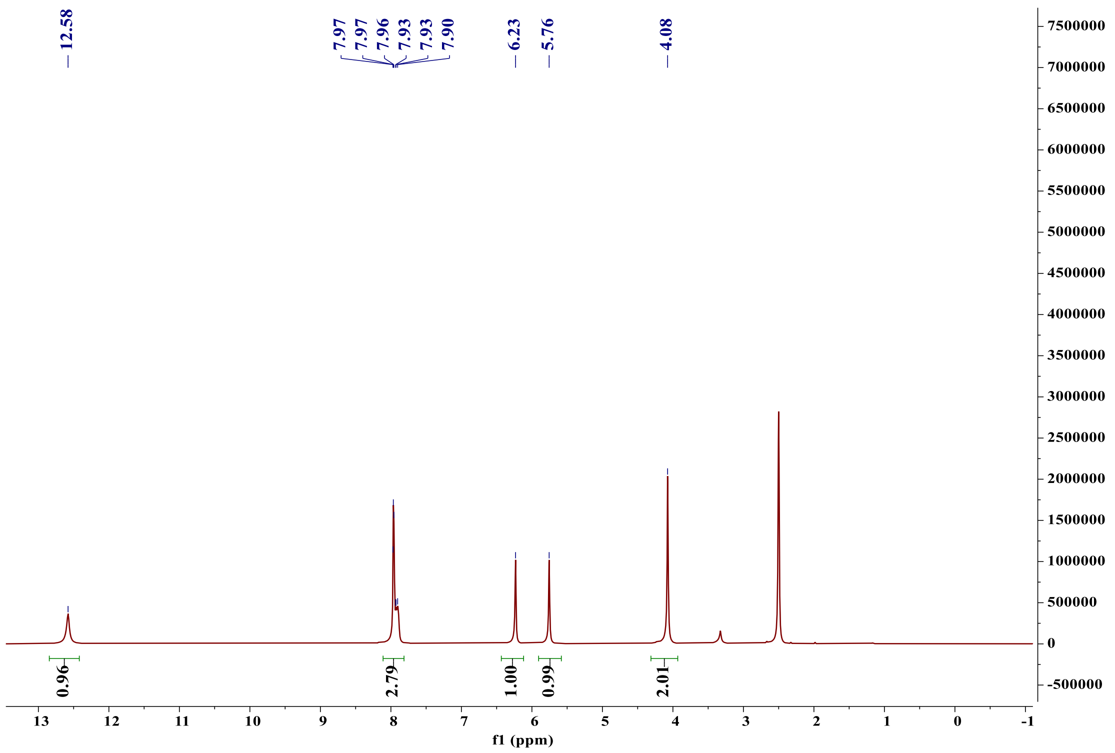

^{1}H NMR (400 MHz, DMSO-d_6) spectrum of **1z**

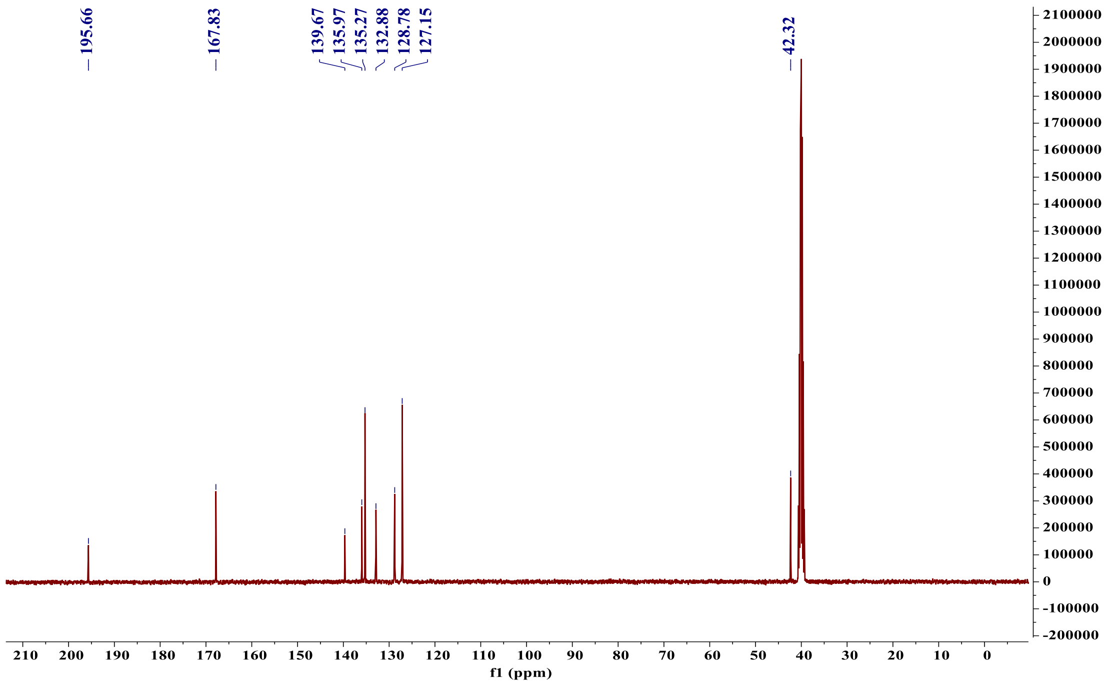

^{13}C NMR (101 MHz, DMSO-d_6) spectrum of **1z**

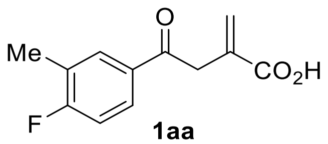

1aa

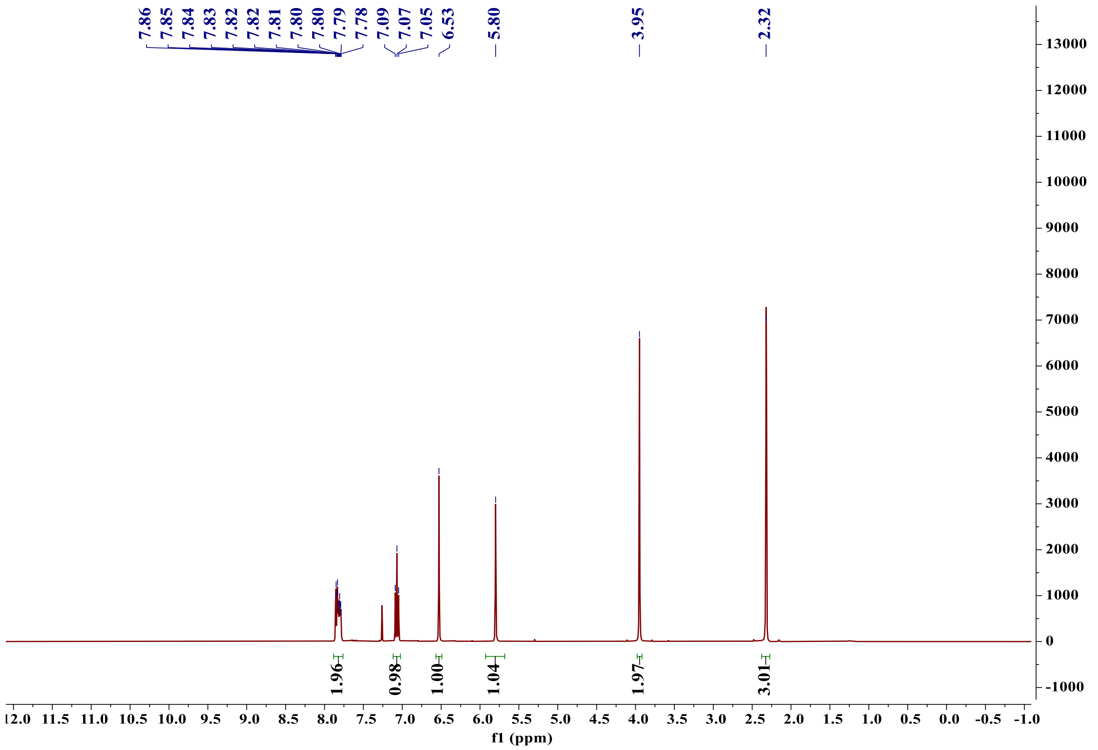

^{1}H NMR (400 MHz, $CDCl_3$) spectrum of **1aa**

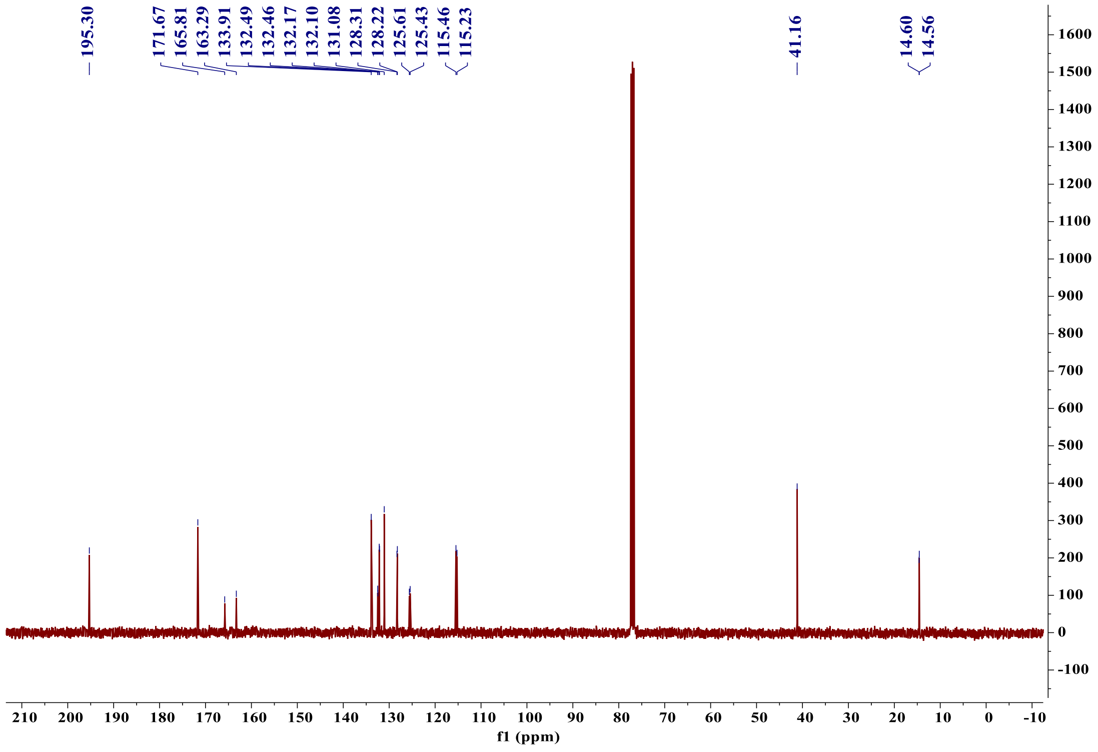

^{13}C NMR (101 MHz, $CDCl_3$) spectrum of **1aa**

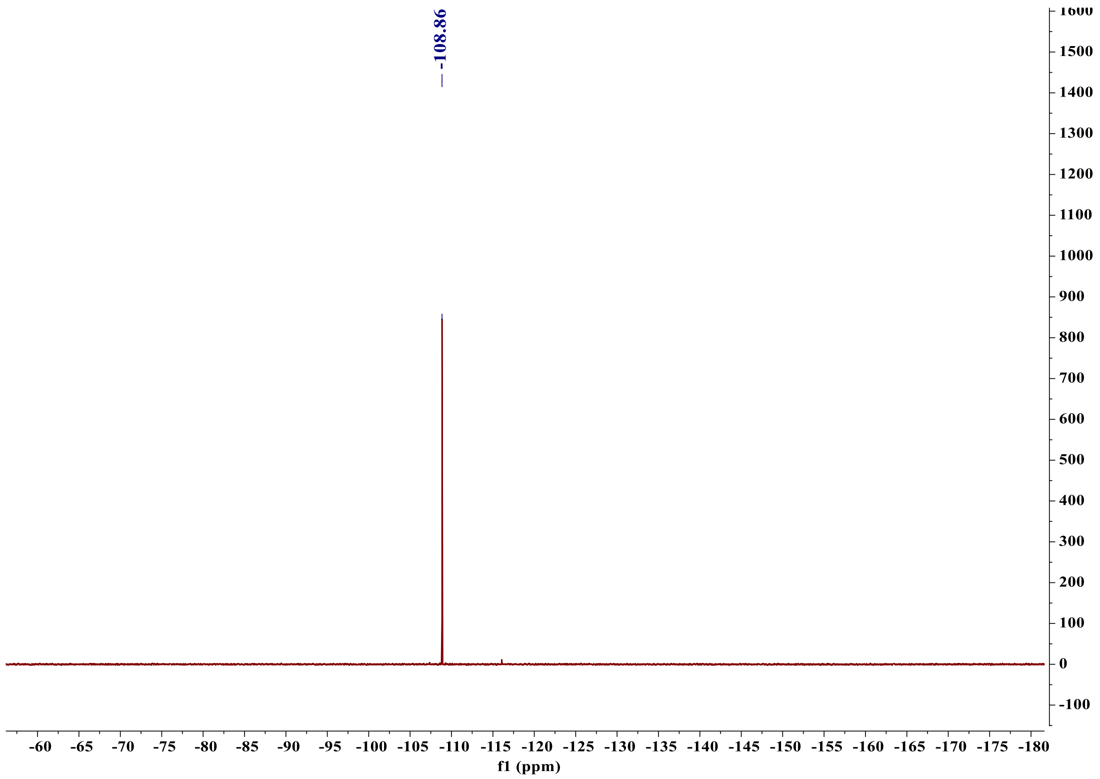

^{19}F NMR (377 MHz, $CDCl_3$) spectrum of **1aa**

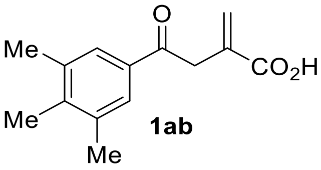

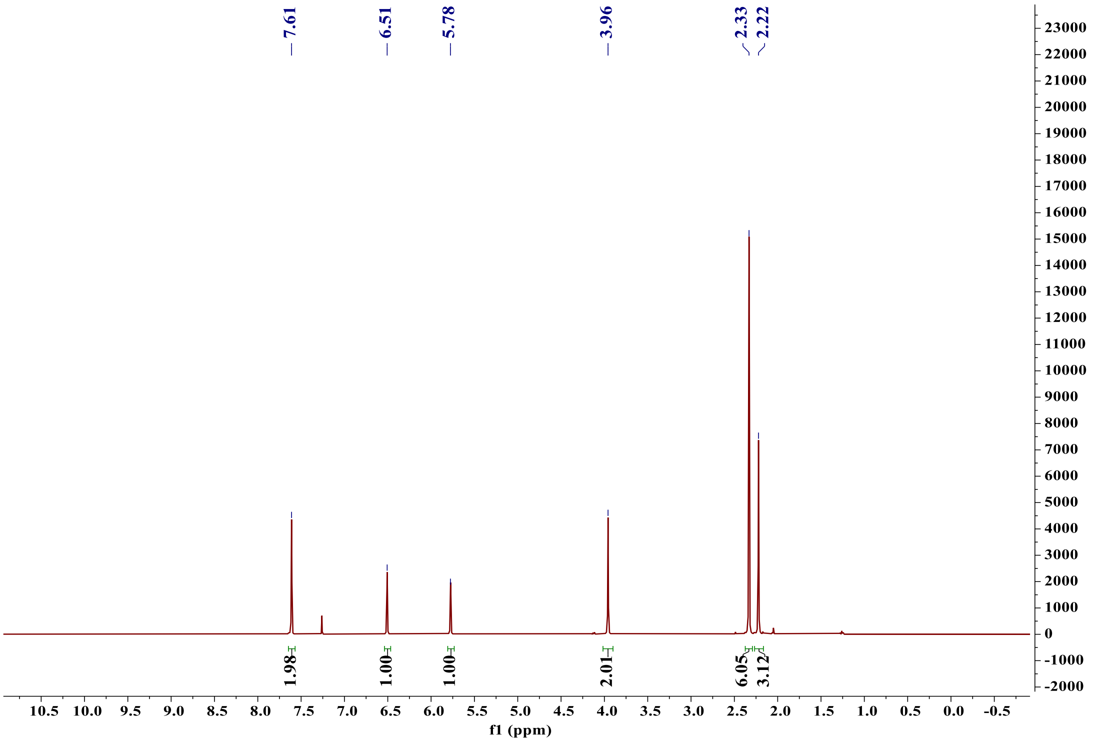

^{1}H NMR (400 MHz, $CDCl_3$) spectrum of **1ab**

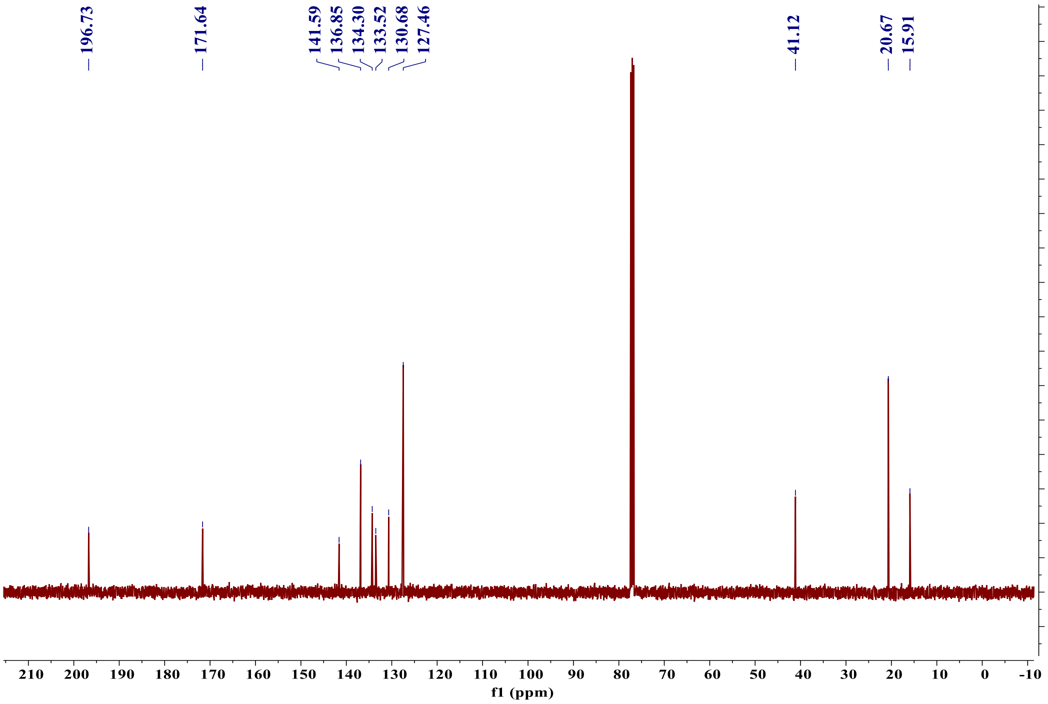

^{13}C NMR (101 MHz, $CDCl_3$) spectrum of **1ab**

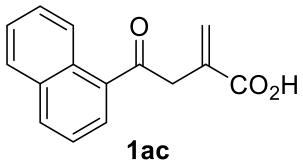

1ac

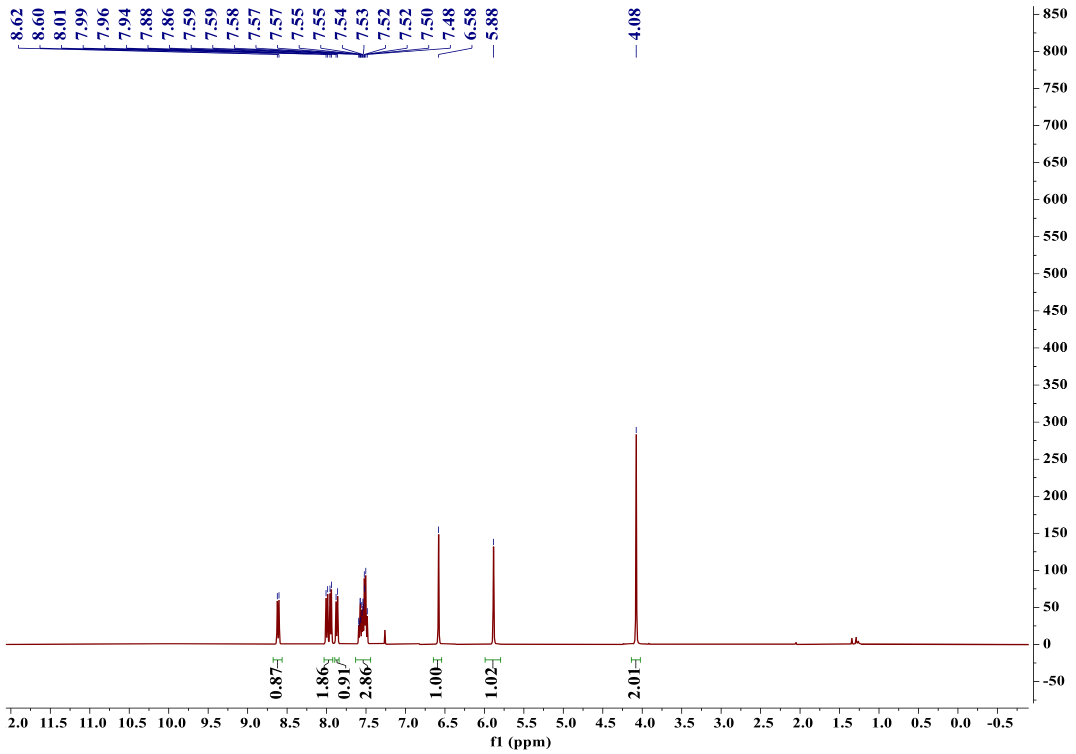

^{1}H NMR (400 MHz, CDCl$_3$) spectrum of **1ac**

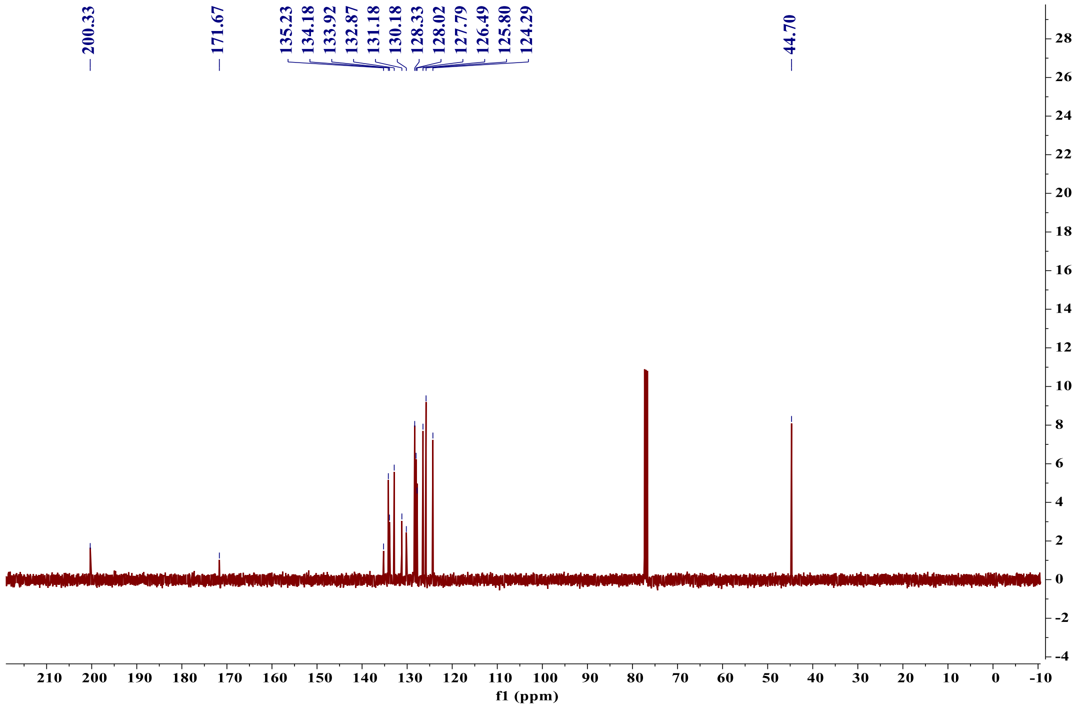

^{13}C NMR (101 MHz, CDCl$_3$) spectrum of **1ac**

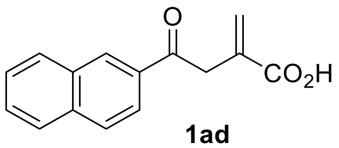

1ad

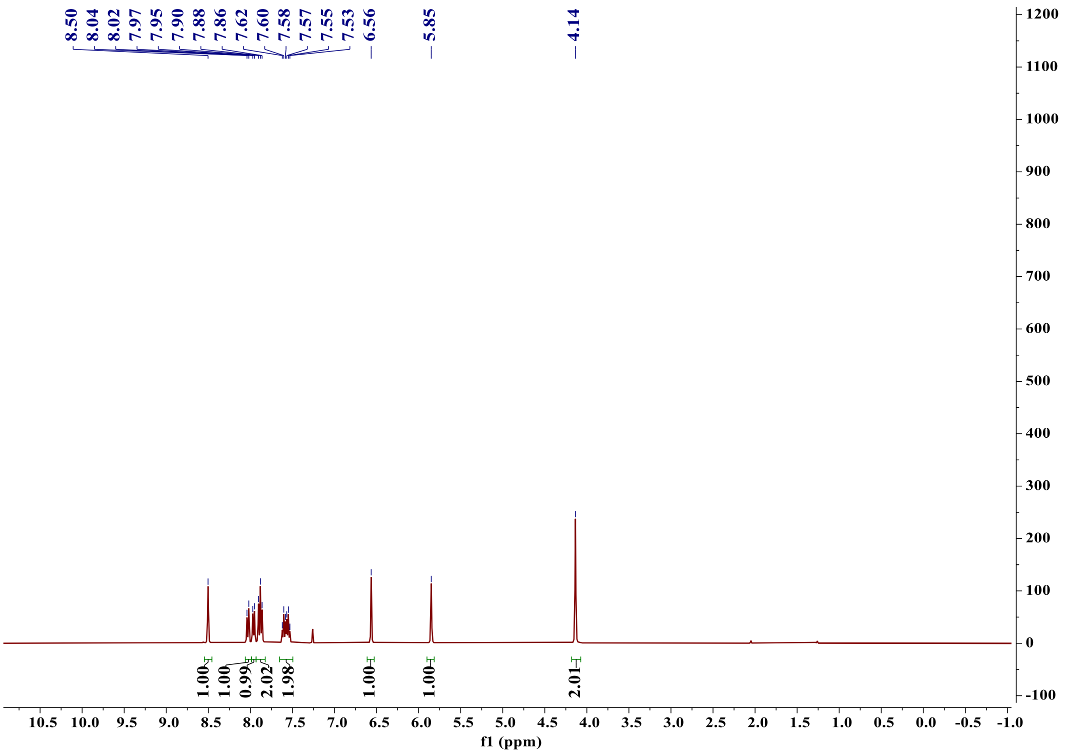

^{1}H NMR (400 MHz, $CDCl_3$) spectrum of **1ad**

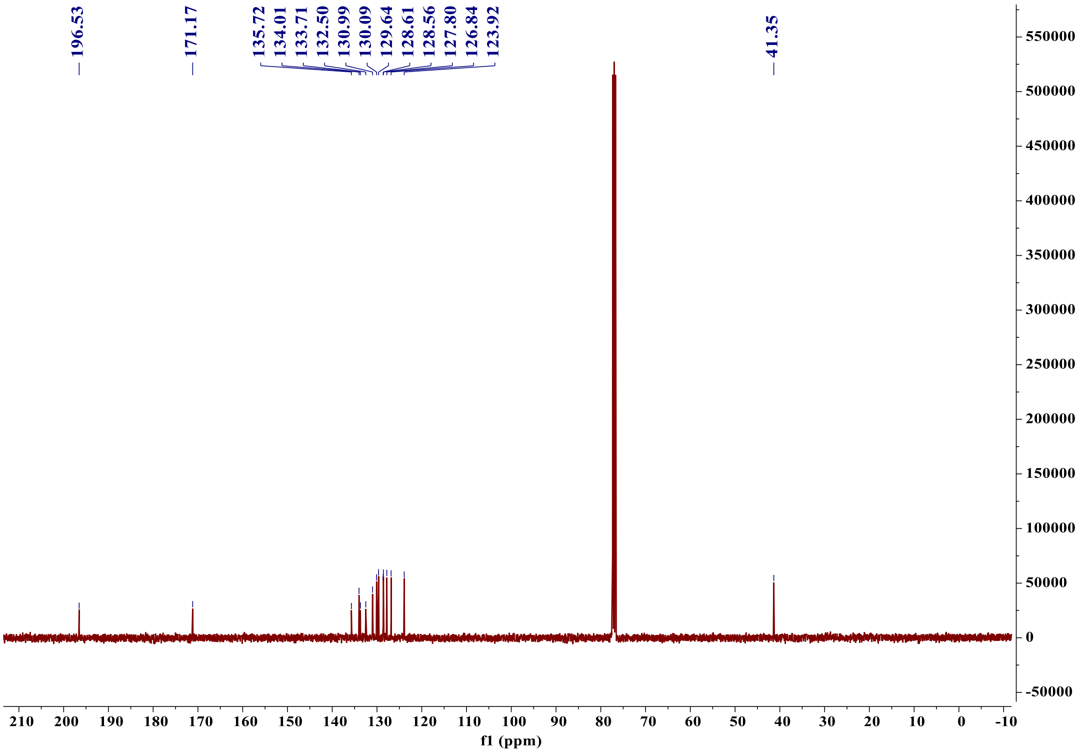

^{13}C NMR (101 MHz, $CDCl_3$) spectrum of **1ad**

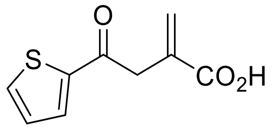

1ae

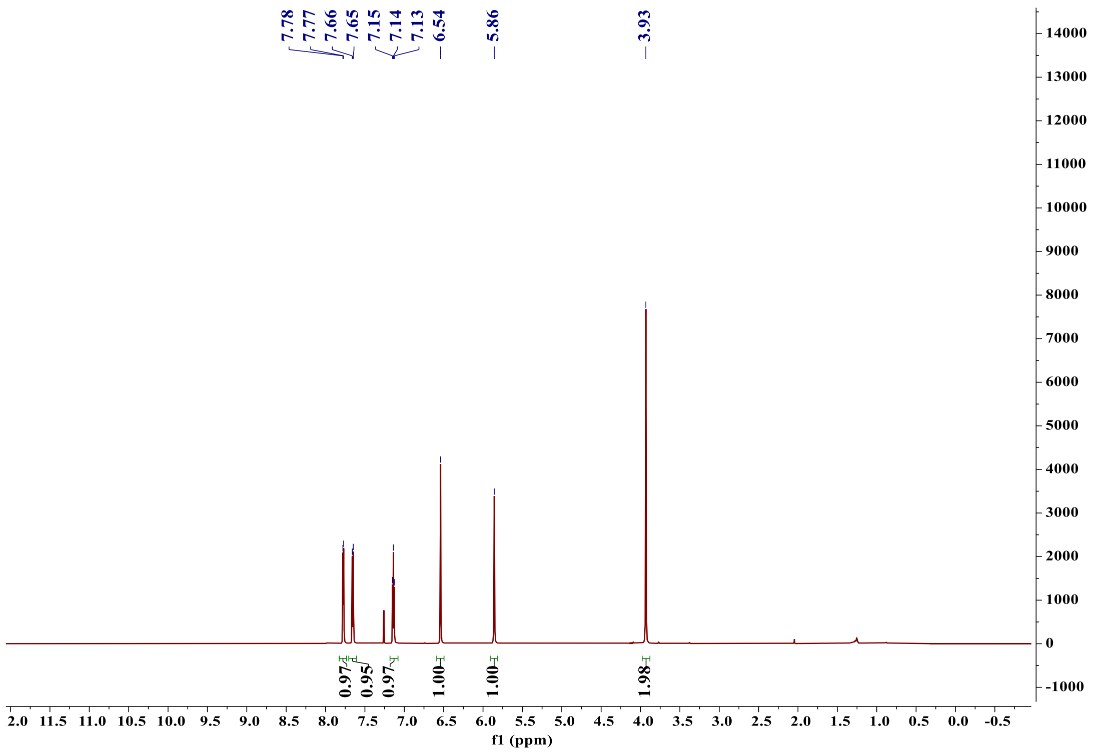

^{1}H NMR (400 MHz, $CDCl_3$) spectrum of **1ae**

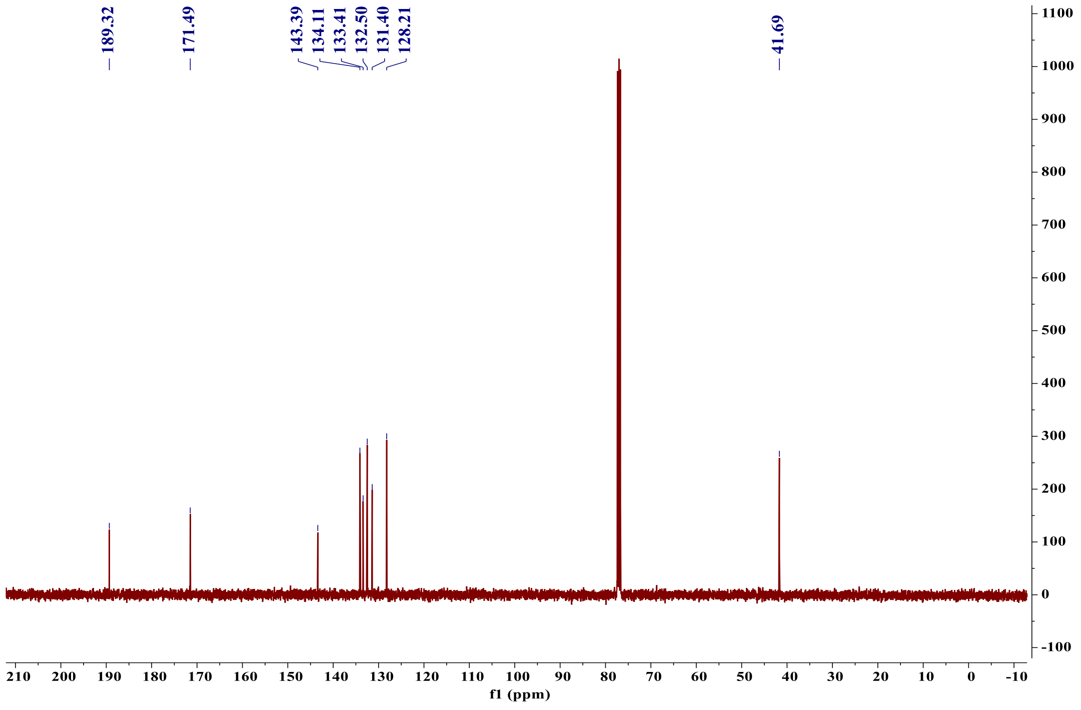

^{13}C NMR (101 MHz, $CDCl_3$) spectrum of **1ae**

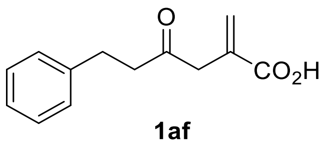

1af

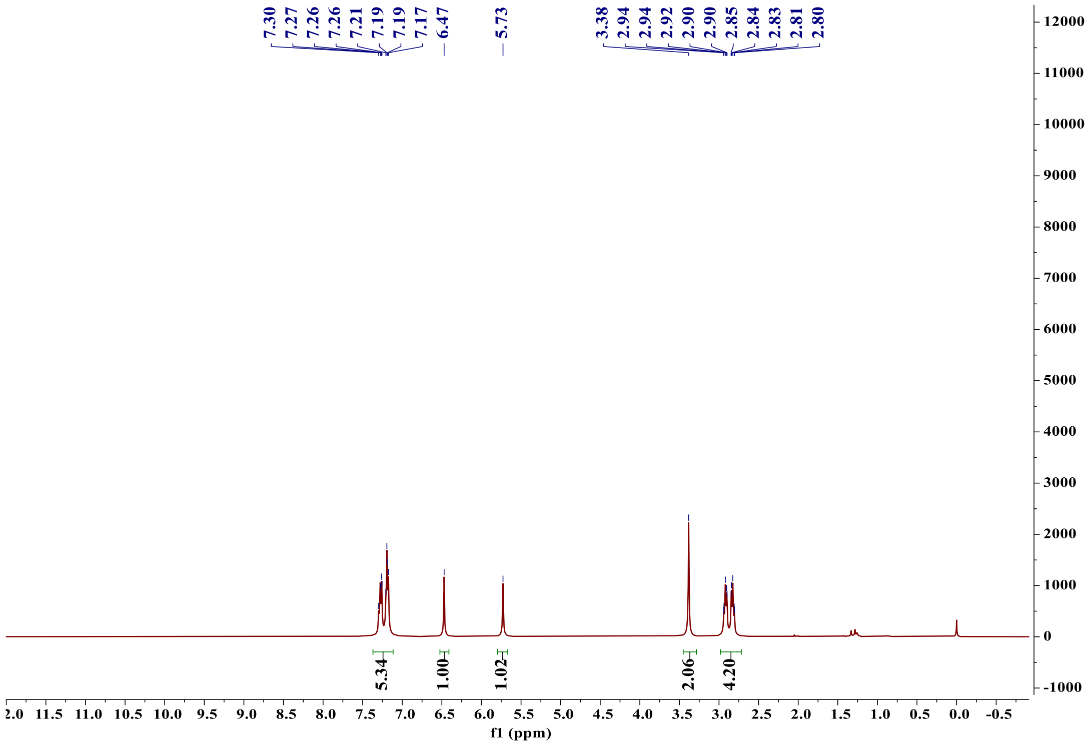

^{1}H NMR (400 MHz, $CDCl_3$) spectrum of **1af**

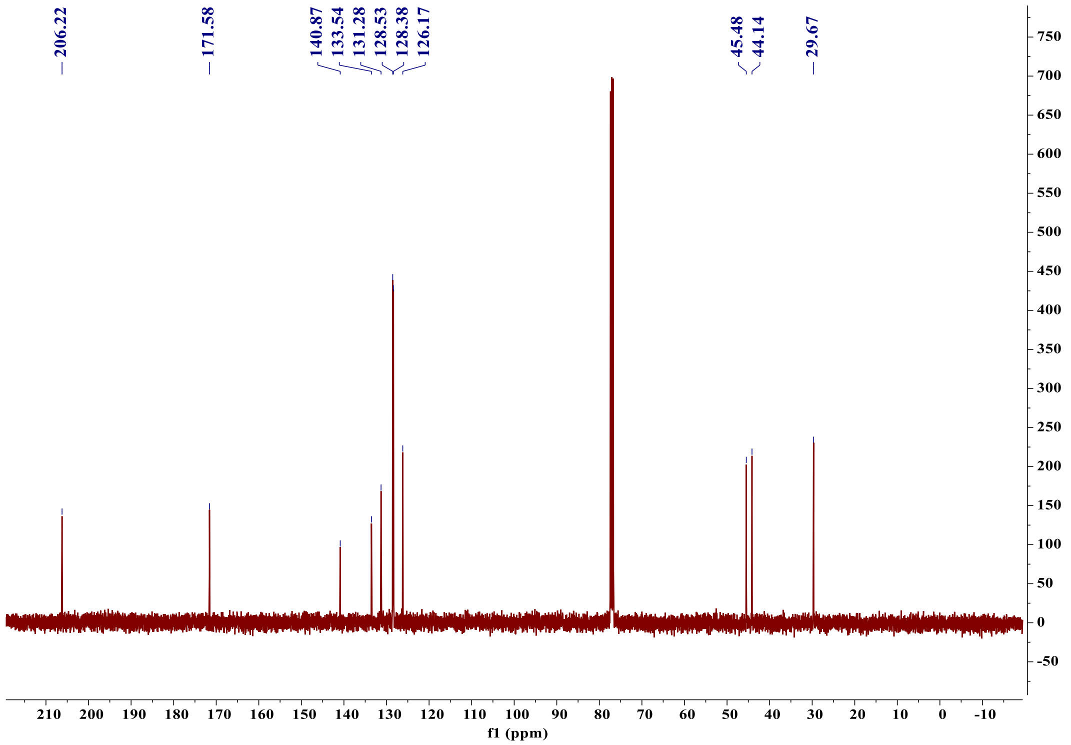

^{13}C NMR (101 MHz, $CDCl_3$) spectrum of **1af**

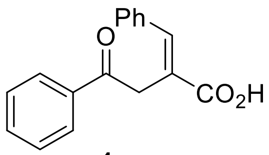

1ag

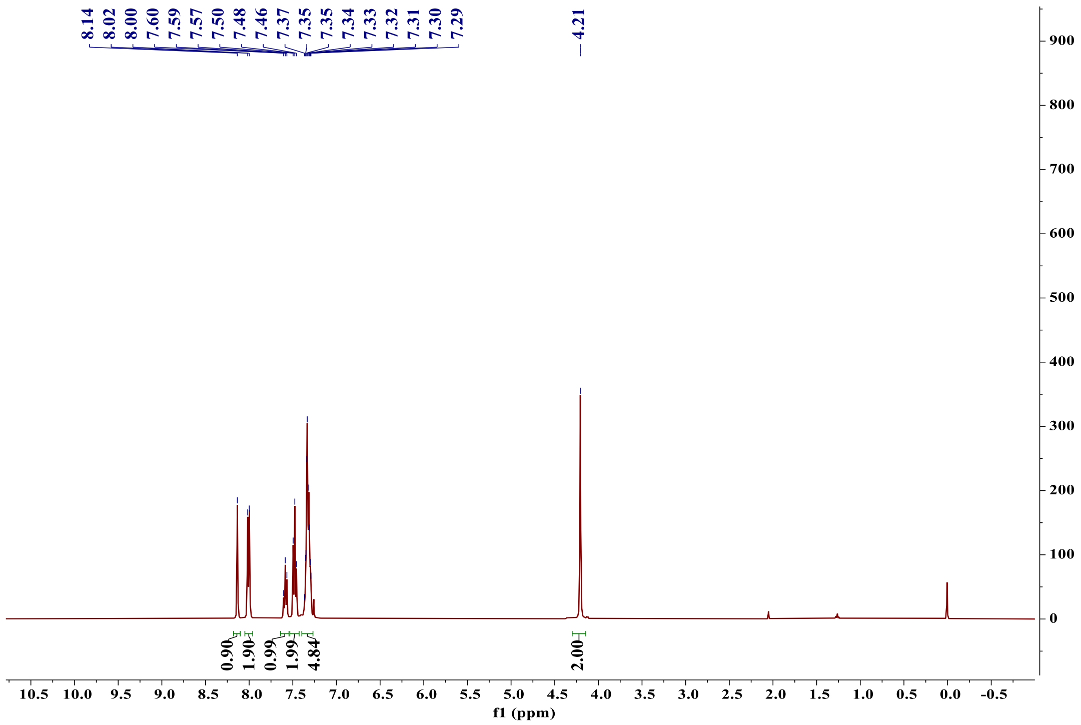

^{1}H NMR (400 MHz, $CDCl_3$) spectrum of **1ag**

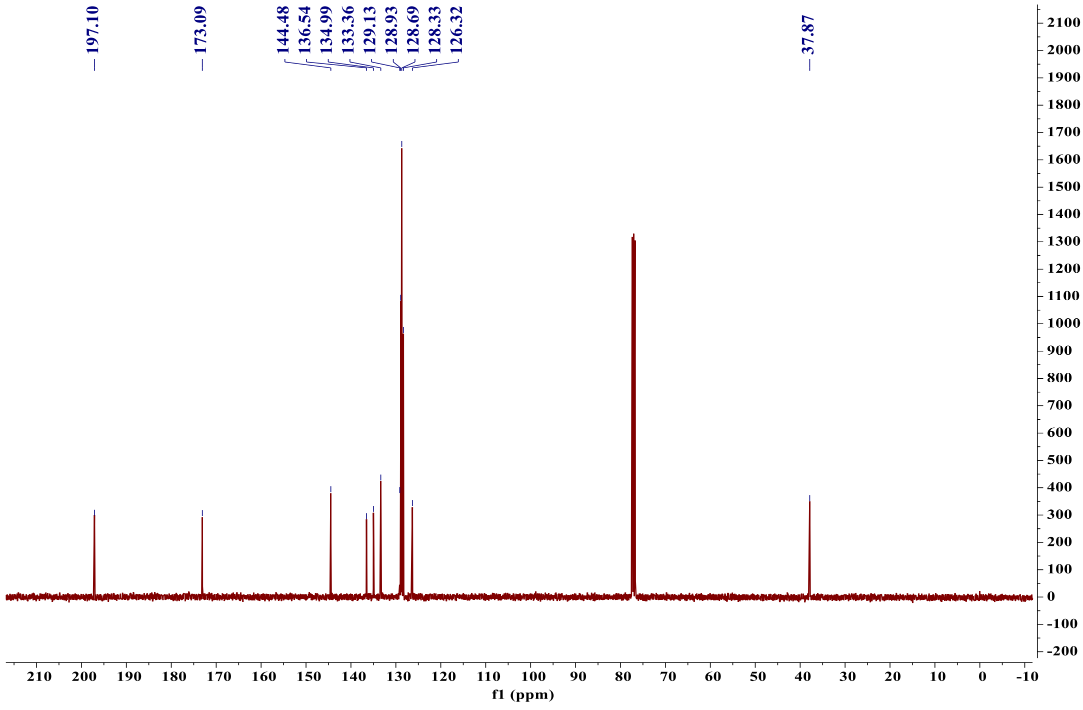

^{13}C NMR (101 MHz, $CDCl_3$) spectrum of **1ag**

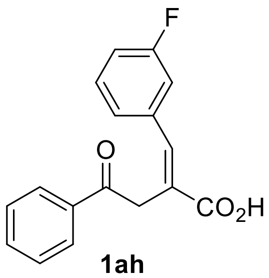

1ah

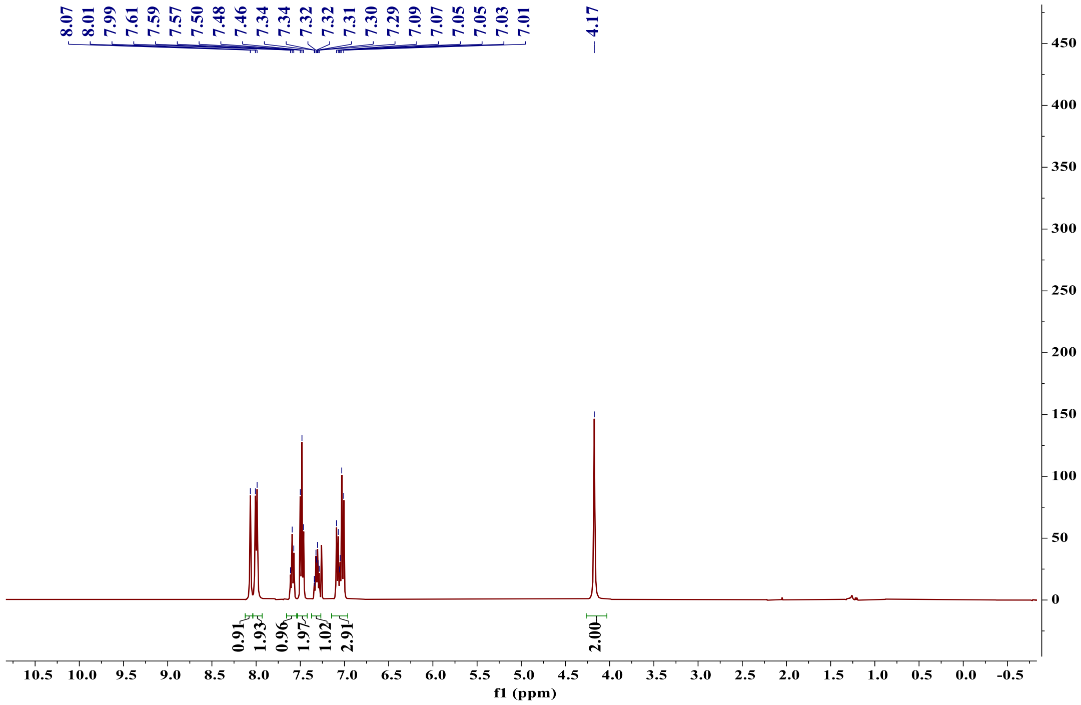

^{1}H NMR (400 MHz, $CDCl_3$) spectrum of **1ah**

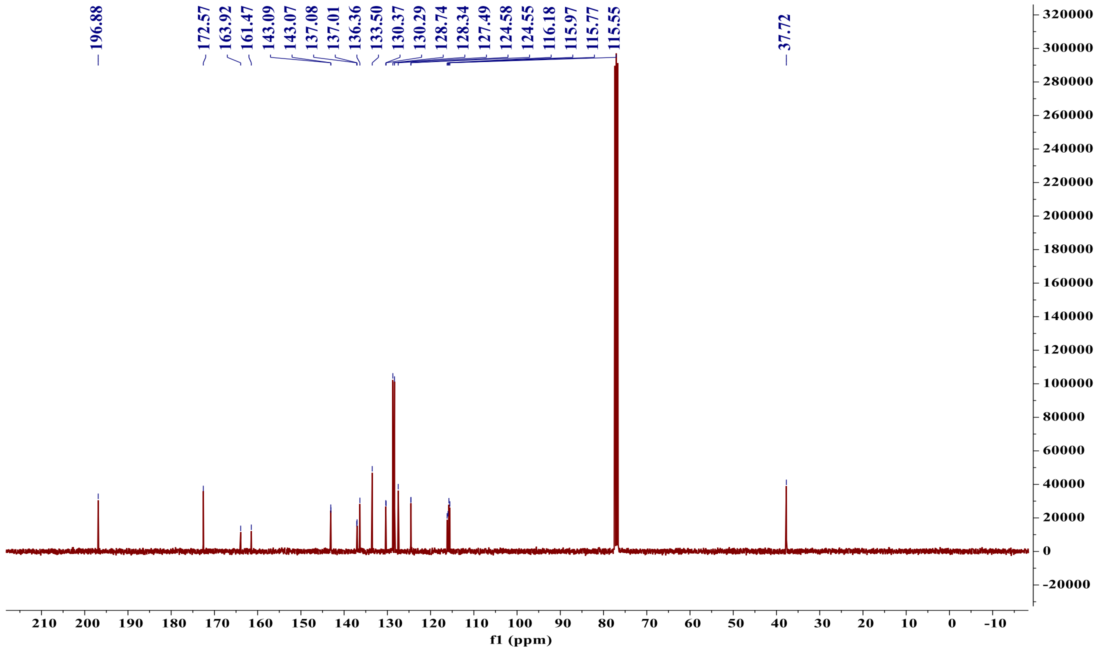

^{13}C NMR (101 MHz, $CDCl_3$) spectrum of **1ah**

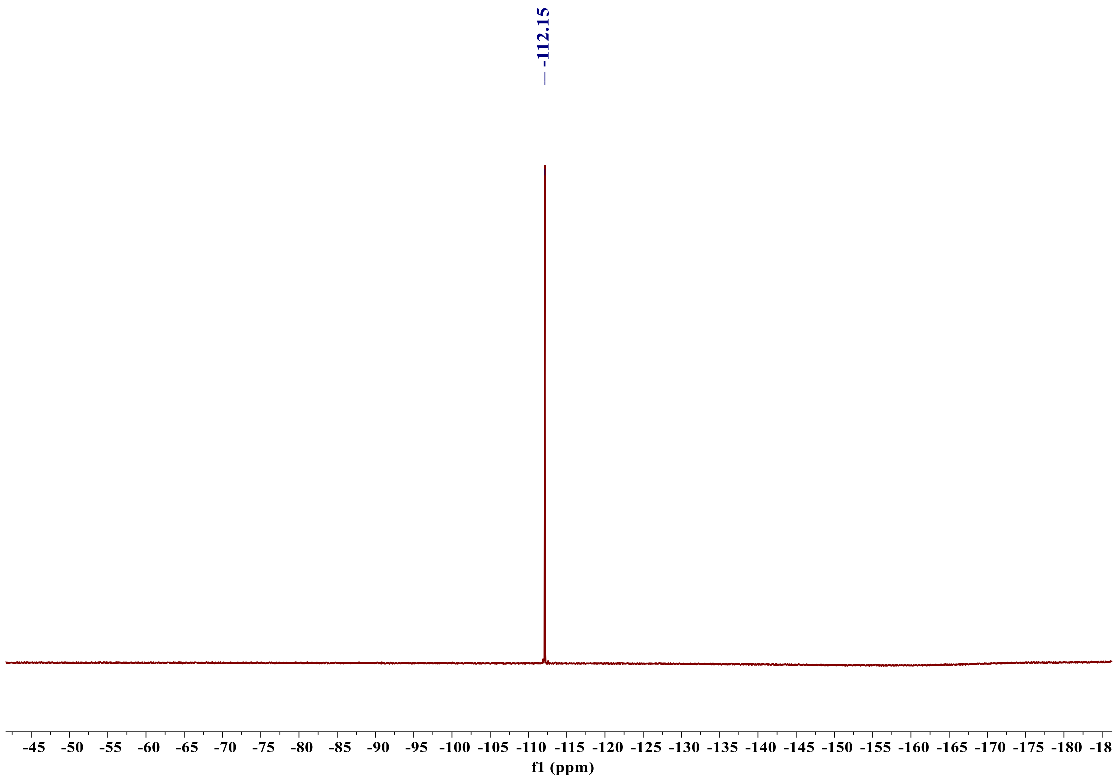

^{19}F NMR (377 MHz, $CDCl_3$) spectrum of **1ah**

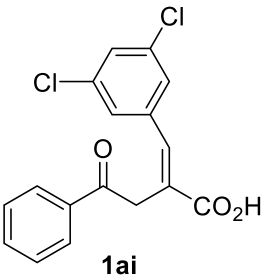

1ai

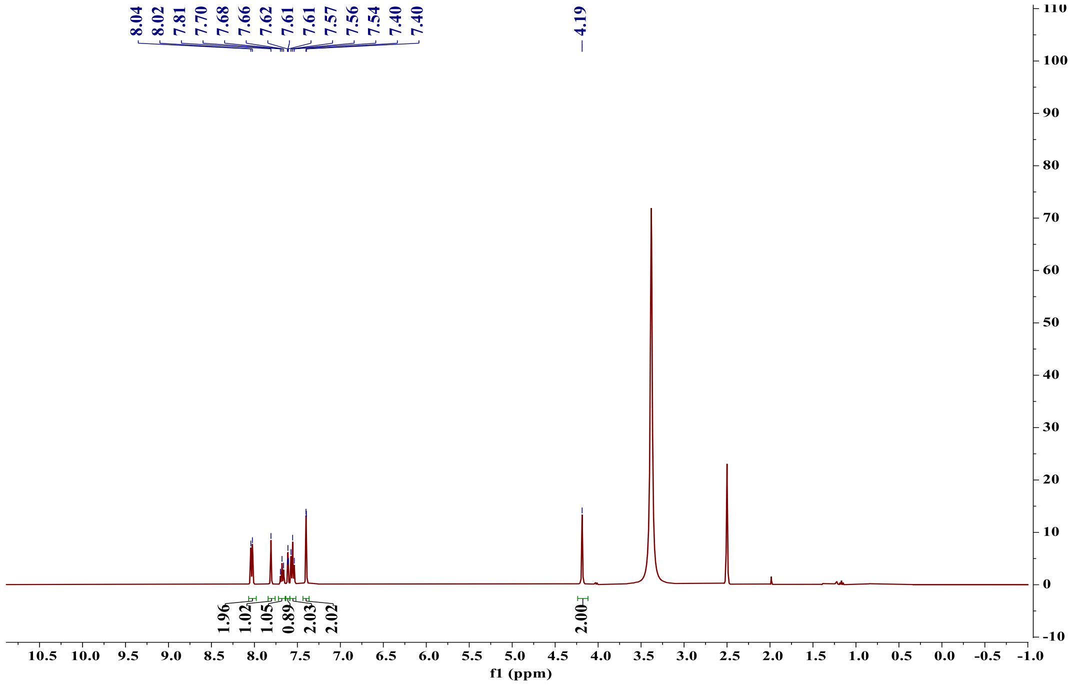

^{1}H NMR (400 MHz, DMSO-d_6) spectrum of **1ai**

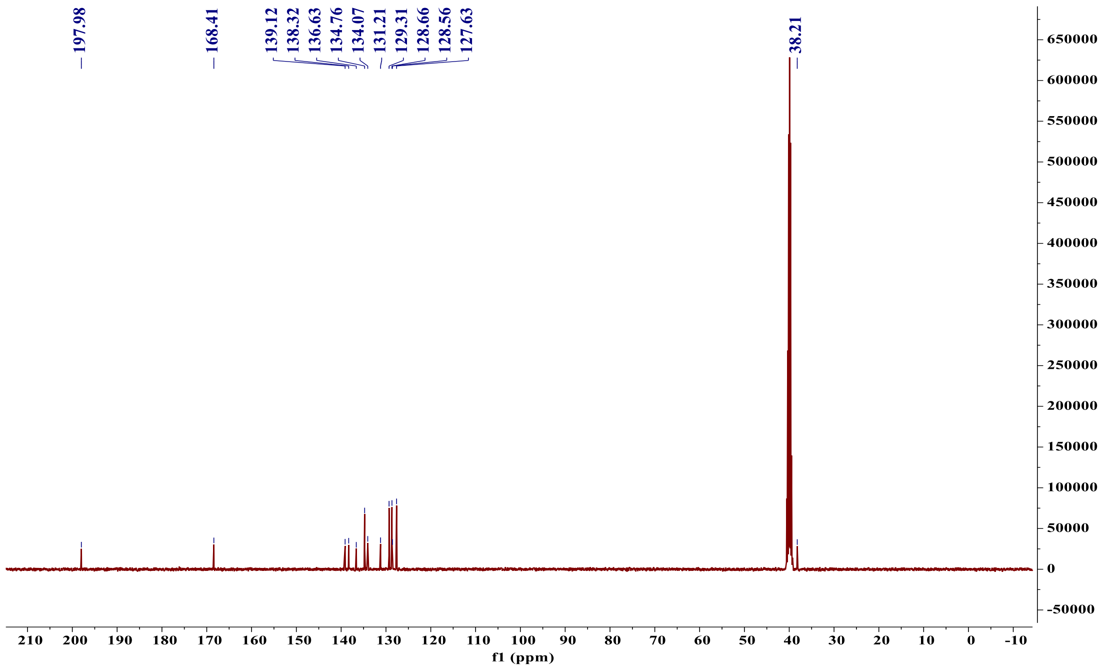

^{13}C NMR (101 MHz, DMSO-d_6) spectrum of **1ai**

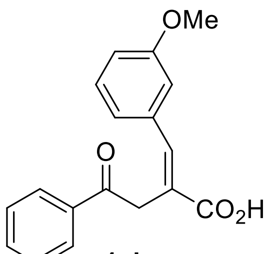

1aj

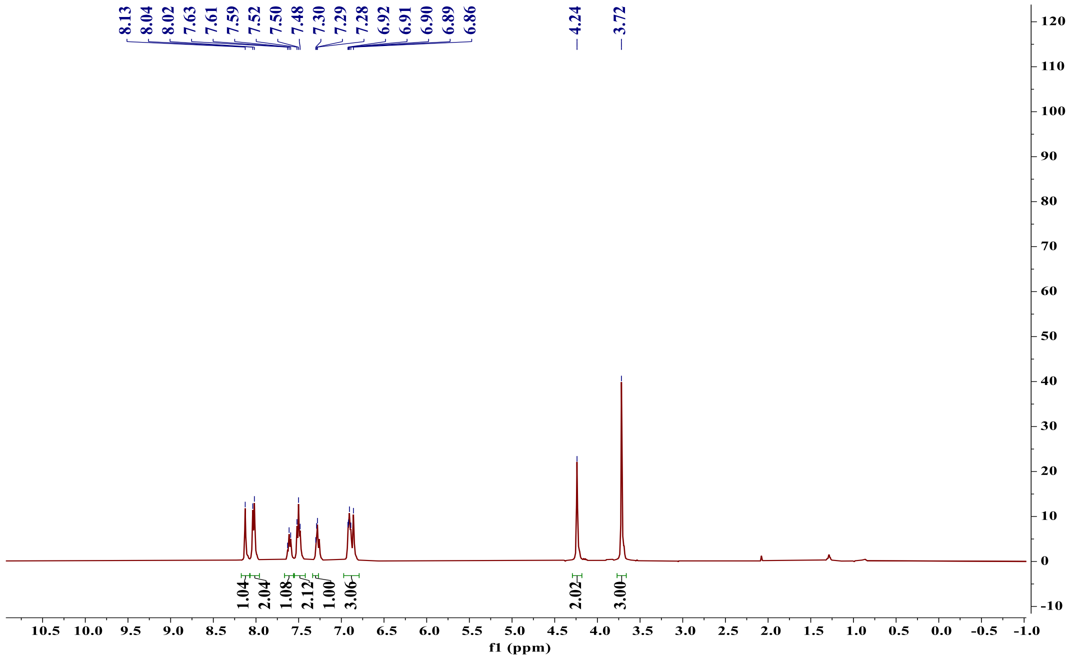

^{1}H NMR (400 MHz, CDCl$_3$) spectrum of **1aj**

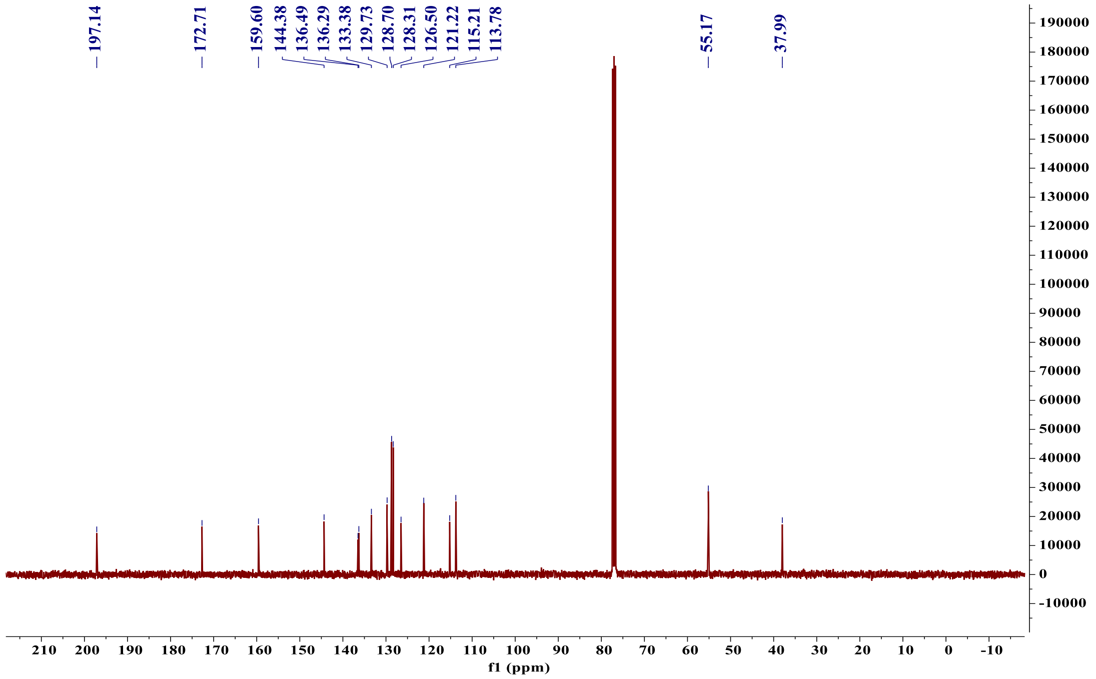

^{13}C NMR (101 MHz, CDCl$_3$) spectrum of **1aj**

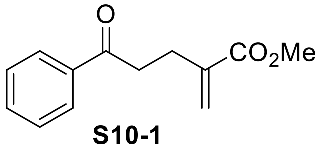

S10-1

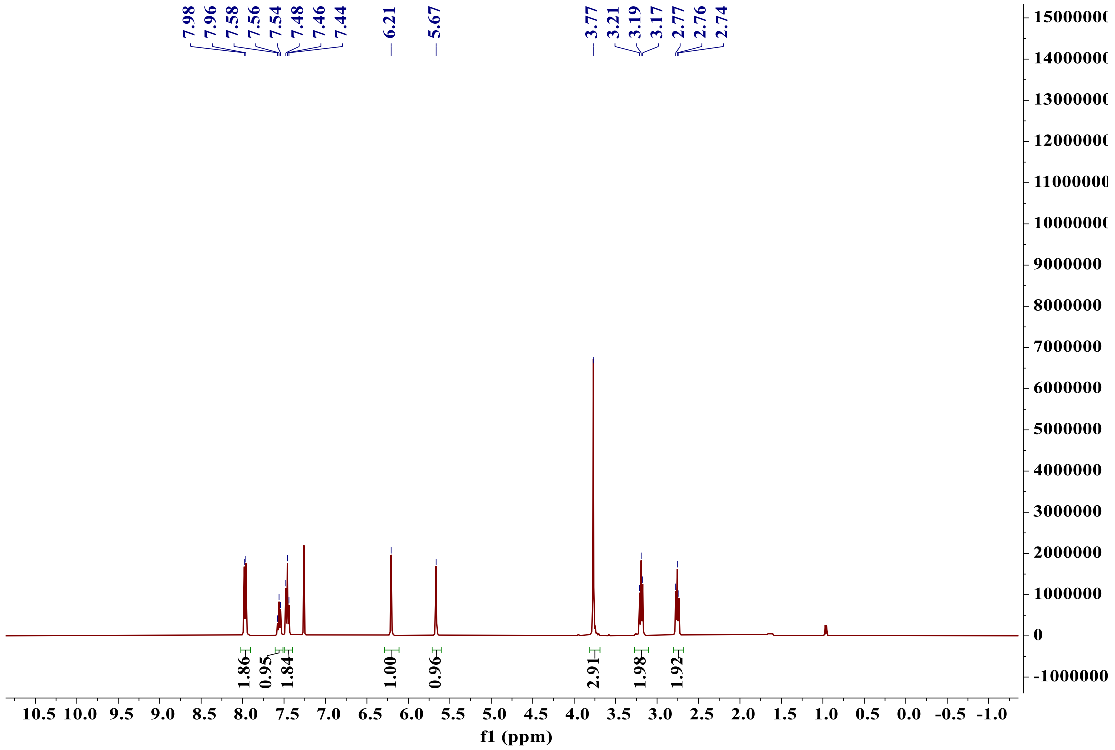

^{1}H NMR (400 MHz, $CDCl_3$) spectrum of **S10-1**

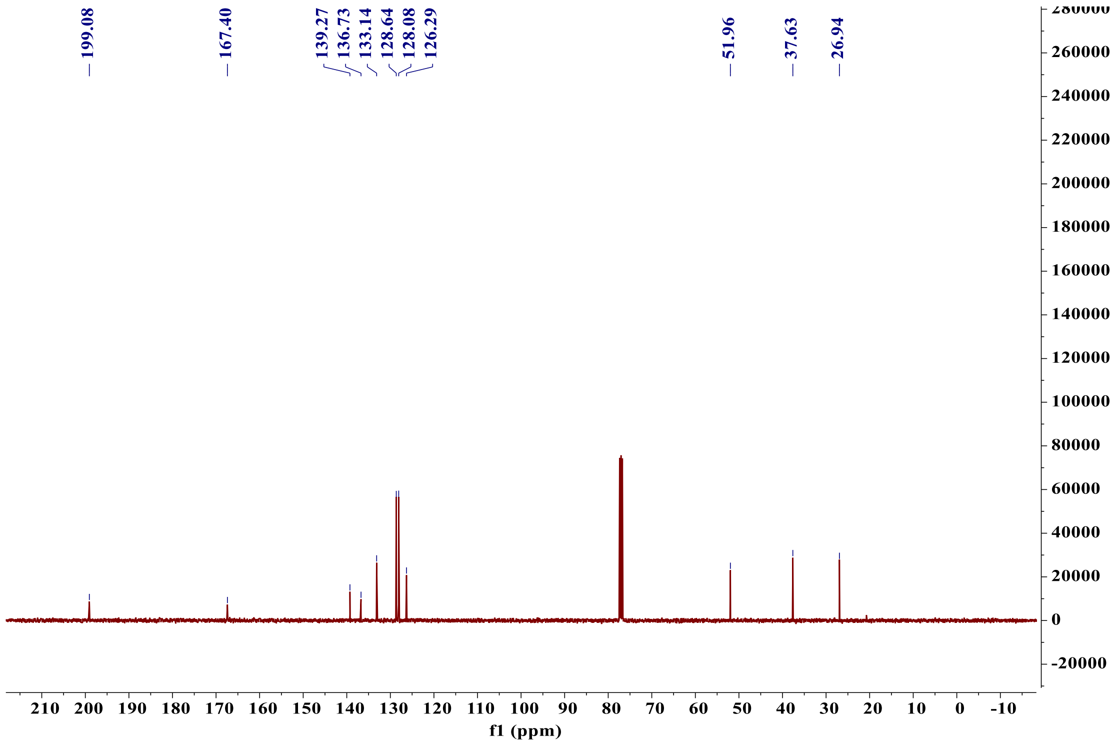

^{13}C NMR (101 MHz, $CDCl_3$) spectrum of **S10-1**

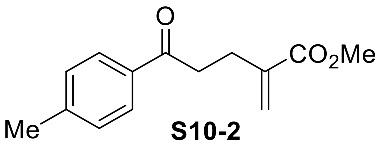

S10-2

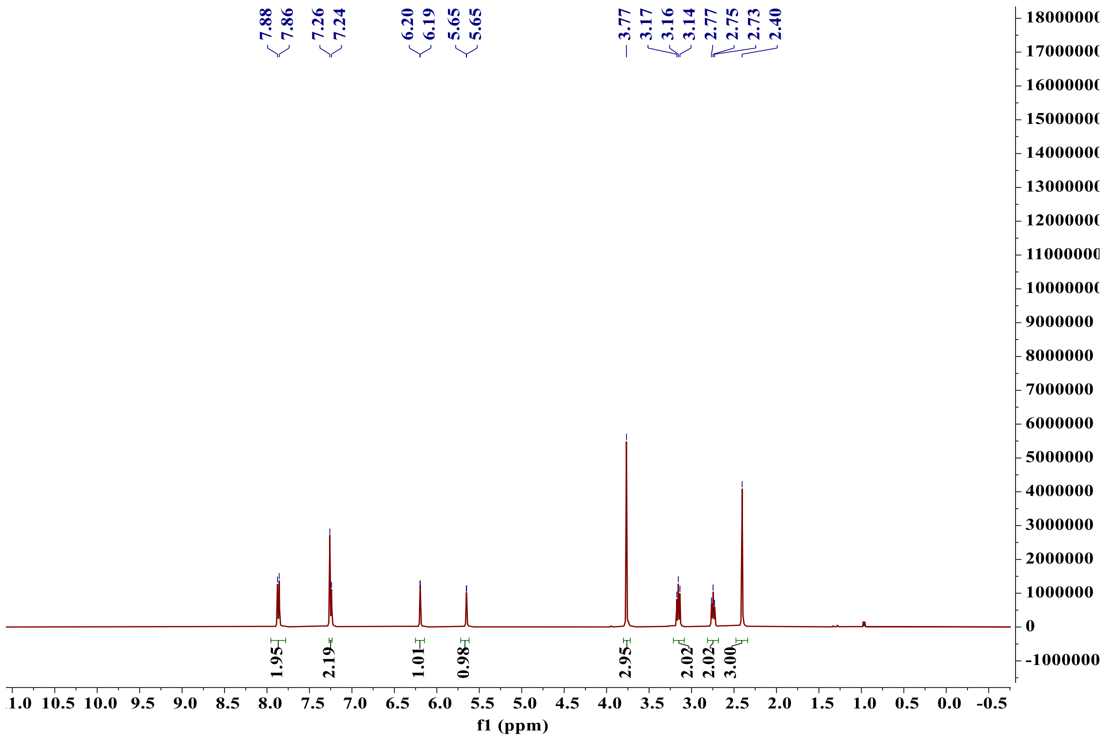

^{1}H NMR (400 MHz, $CDCl_3$) spectrum of **S10-2**

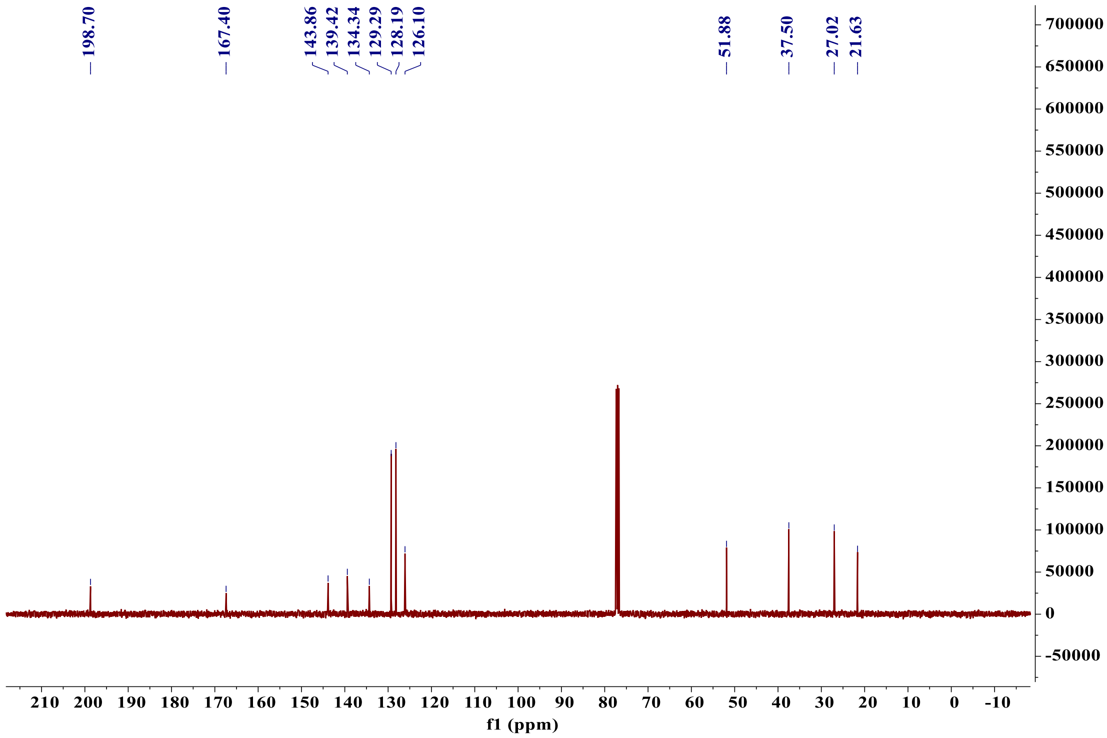

^{13}C NMR (101 MHz, $CDCl_3$) spectrum of **S10-2**

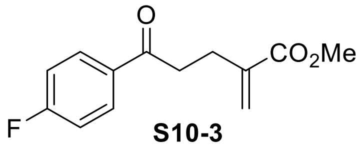

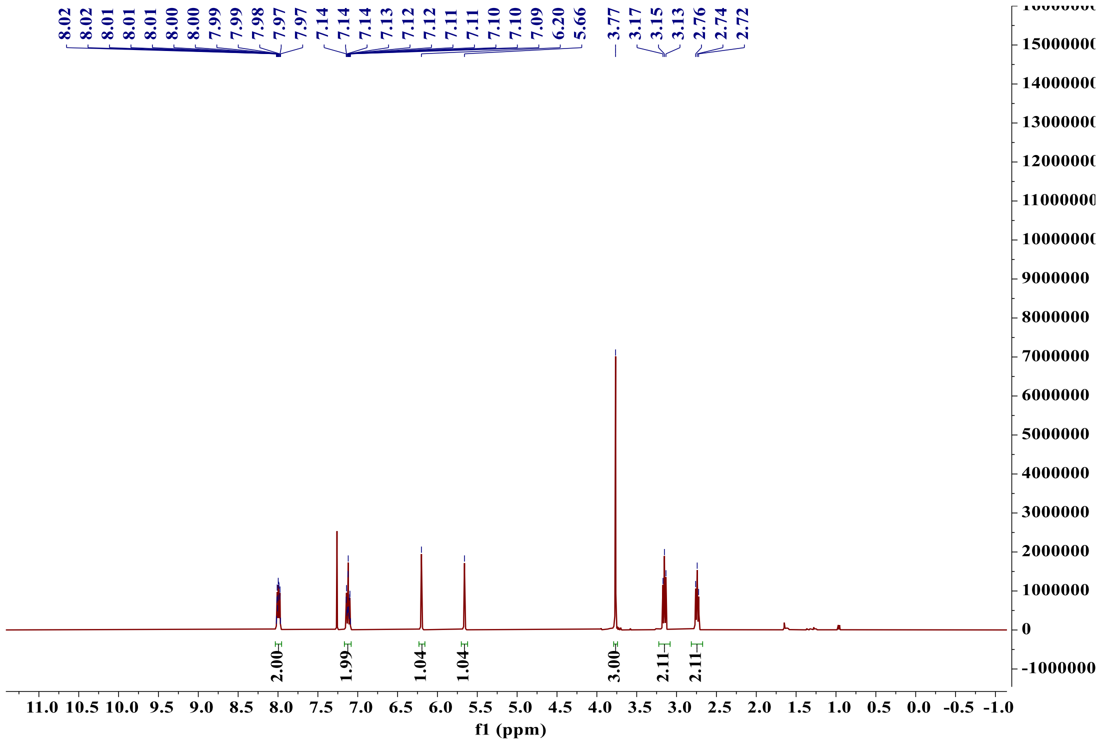

^{1}H NMR (400 MHz, $CDCl_3$) spectrum of **S10-3**

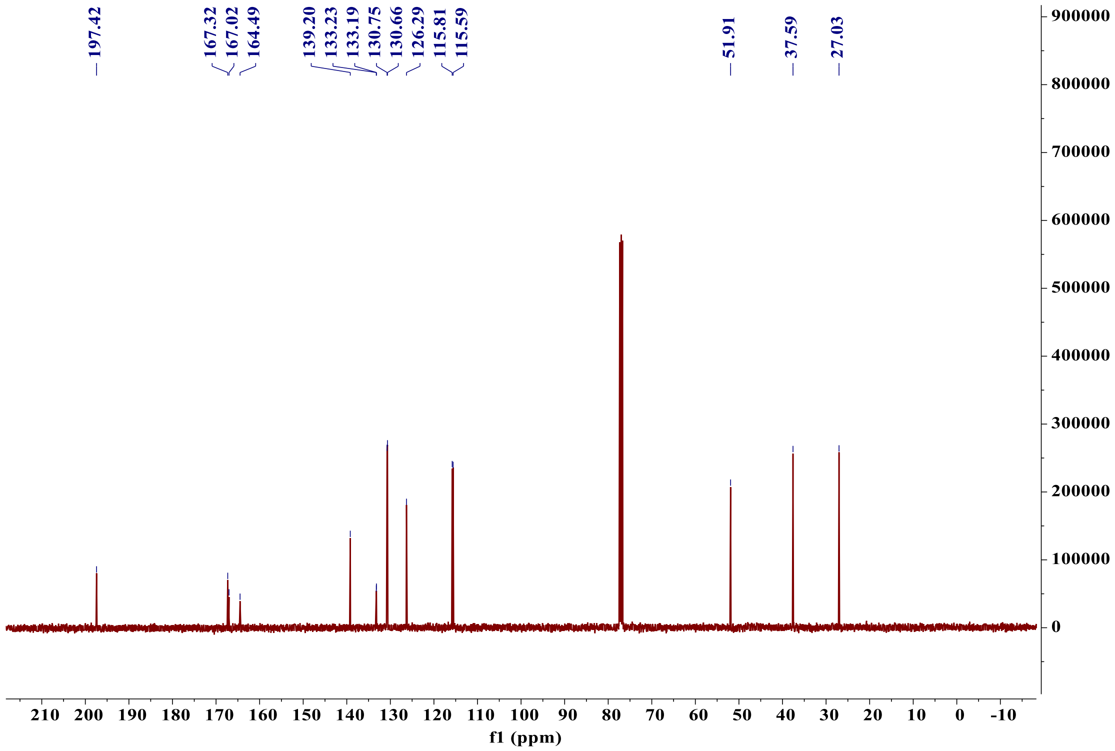

^{13}C NMR (101 MHz, $CDCl_3$) spectrum of **S10-3**

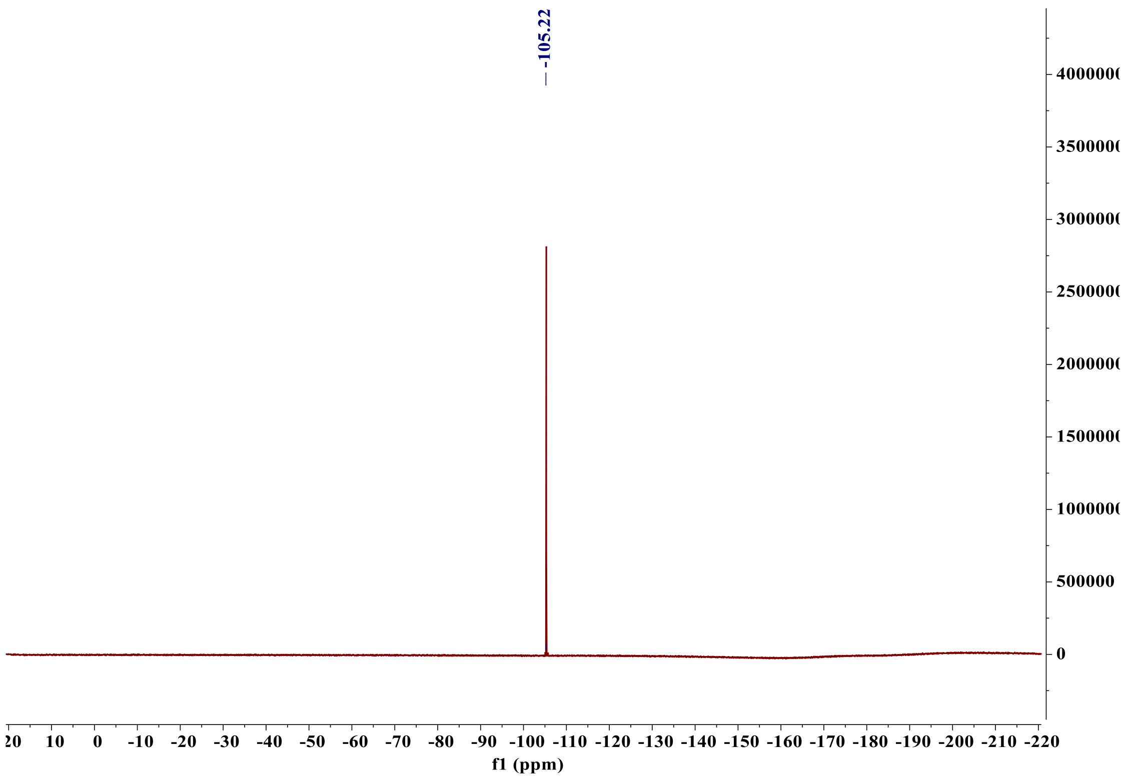

^{19}F NMR (377 MHz, $CDCl_3$) spectrum of **S10-3**

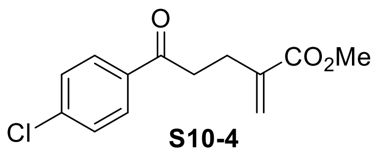

S10-4

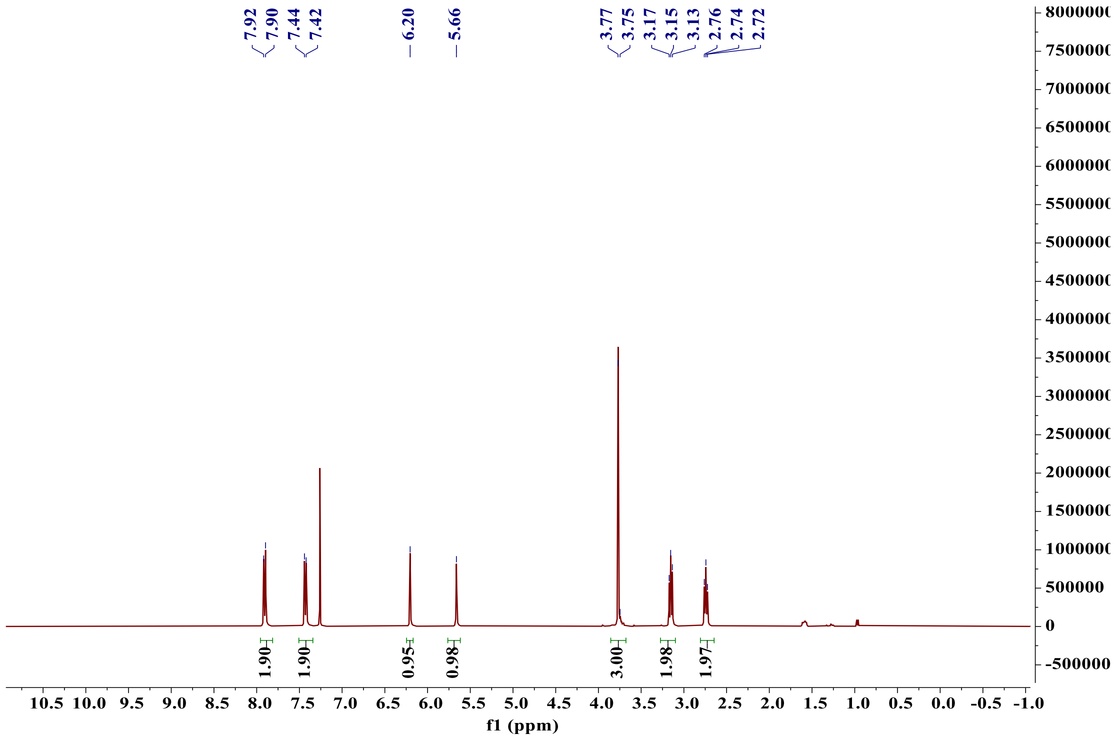

^1H NMR (400 MHz, $CDCl_3$) spectrum of **S10-4**

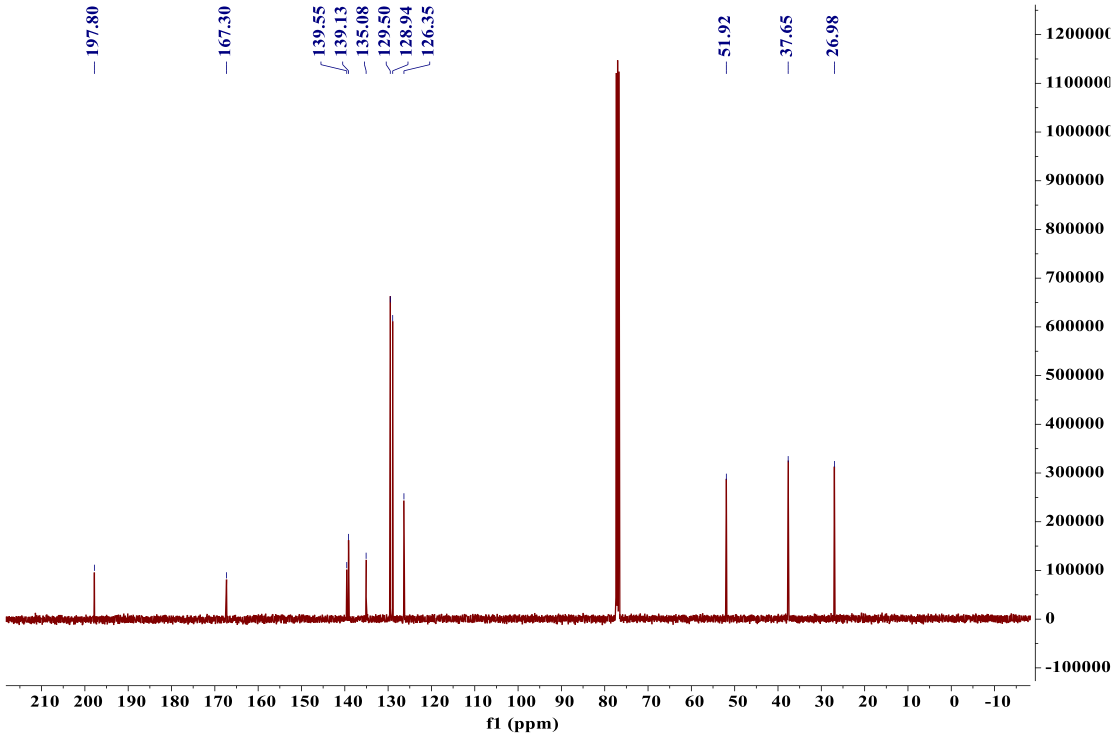

^{13}C NMR (101 MHz, $CDCl_3$) spectrum of **S10-4**

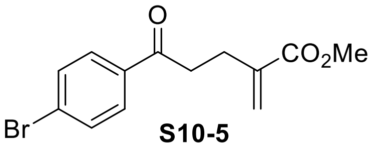

S10-5

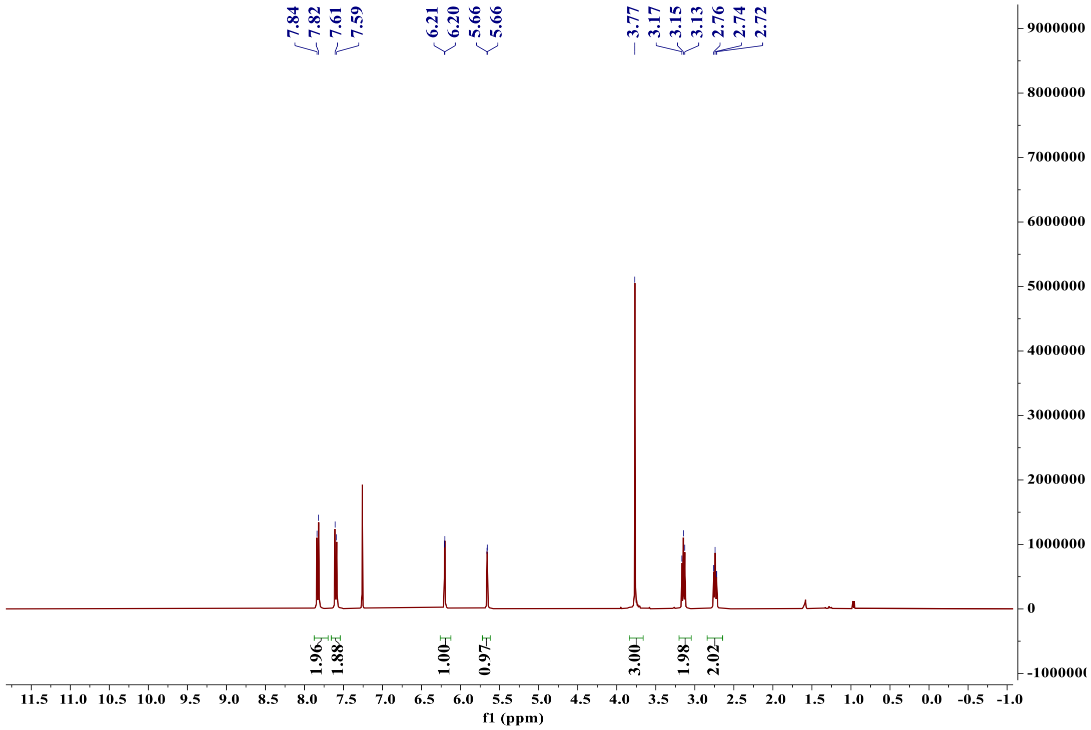

^{1}H NMR (400 MHz, CDCl3) spectrum of **S10-5**

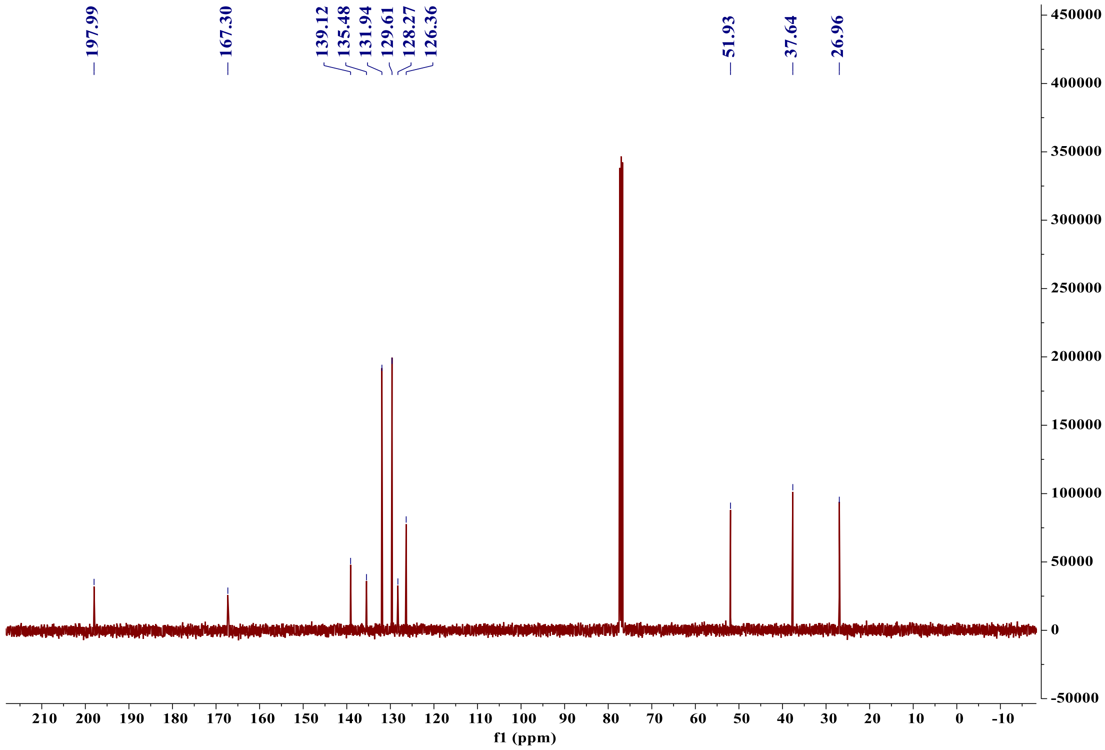

^{13}C NMR (101 MHz, CDCl3) spectrum of **S10-5**

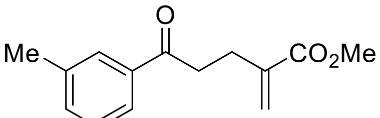

S10-6

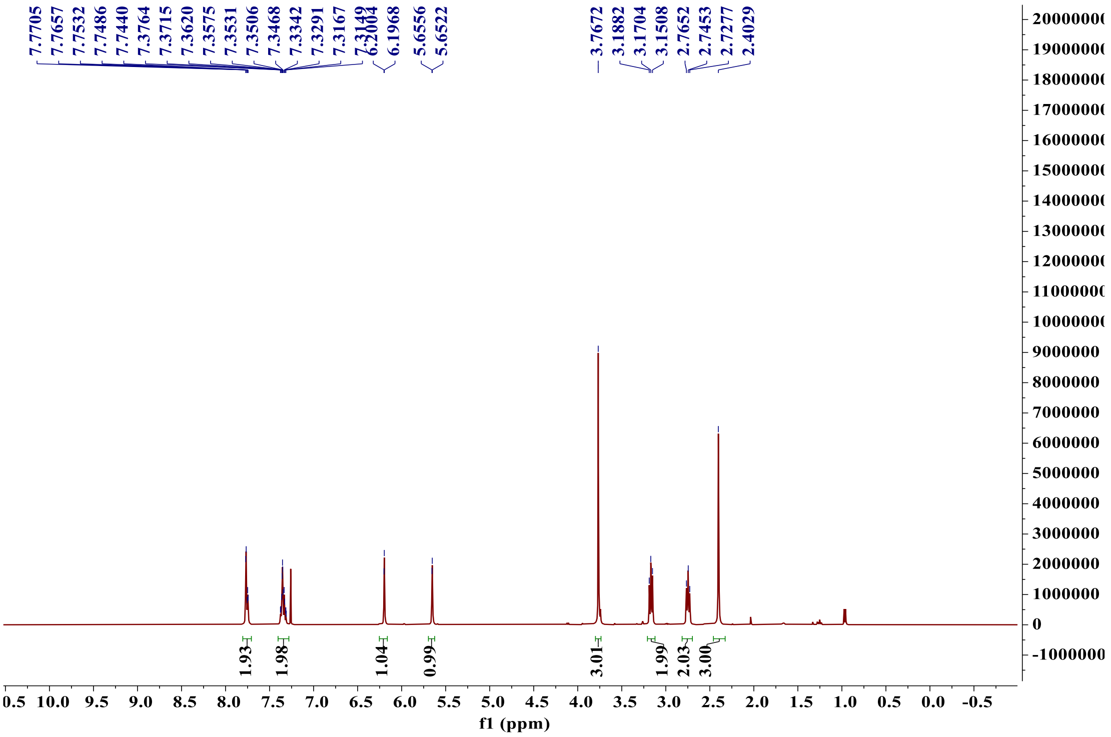

^{1}H NMR (400 MHz, $CDCl_3$) spectrum of **S10-6**

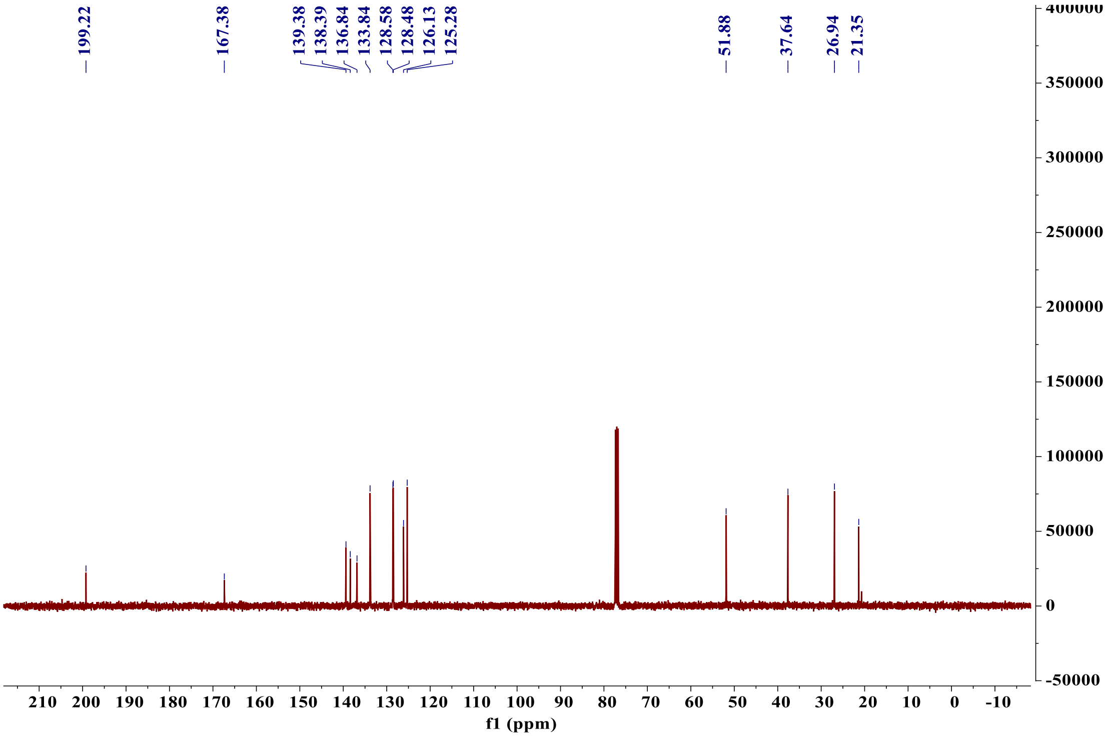

^{13}C NMR (101 MHz, $CDCl_3$) spectrum of **S10-6**

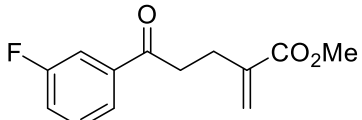

S10-7

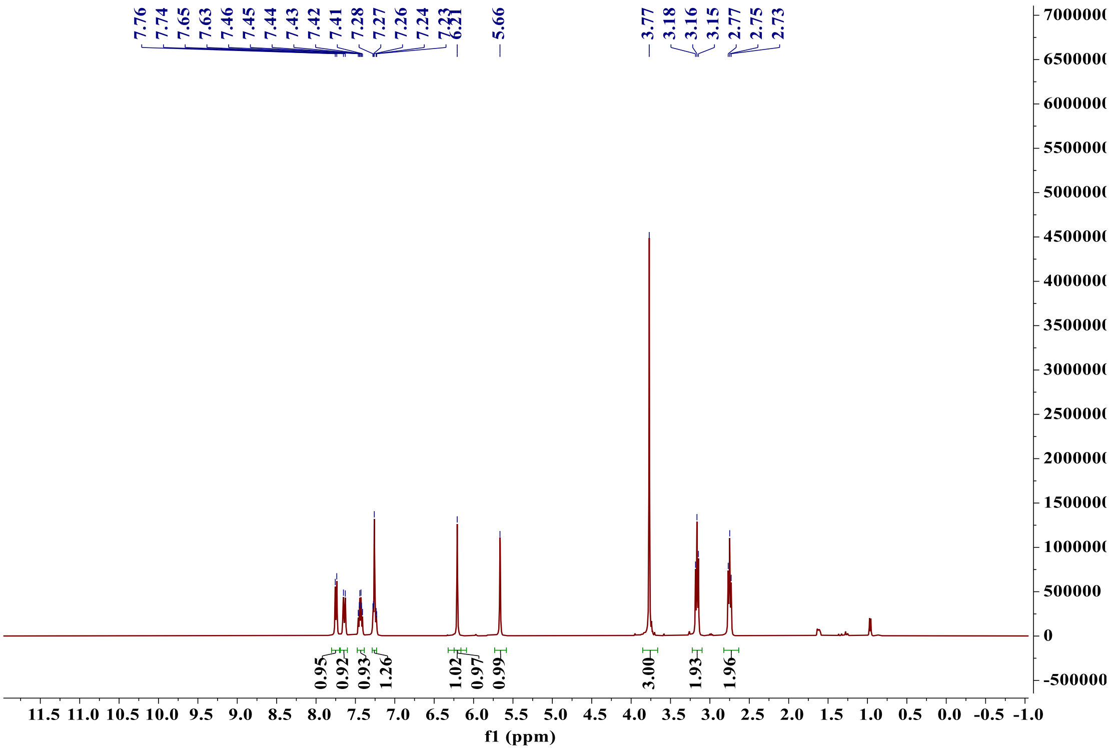

^1H NMR (400 MHz, $CDCl_3$) spectrum of **S10-7**

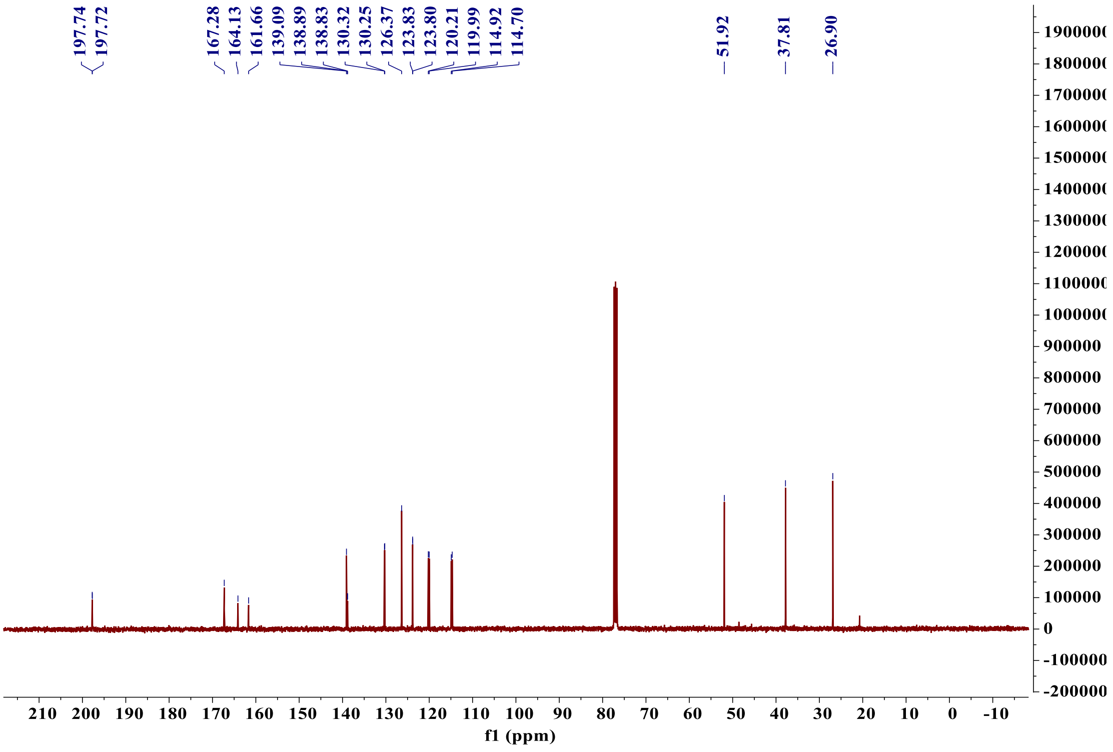

^{13}C NMR (101 MHz, $CDCl_3$) spectrum of **S10-7**

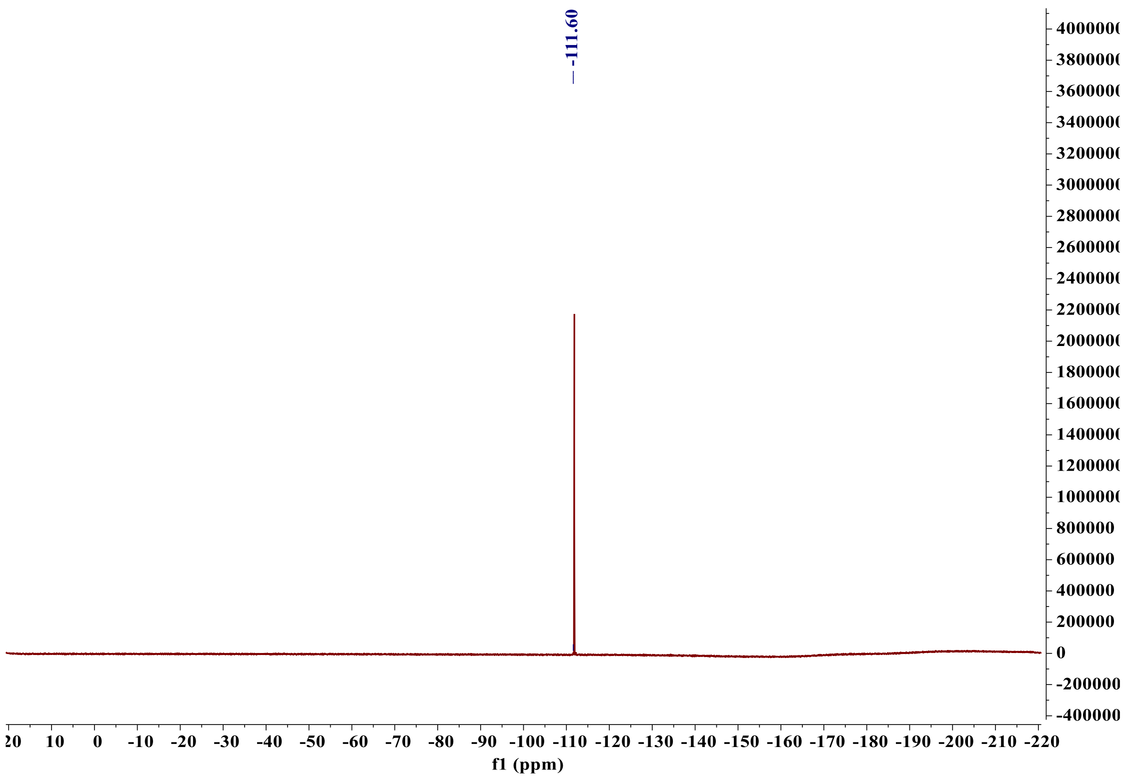

^{19}F NMR (377 MHz, $CDCl_3$) spectrum of **S10-7**

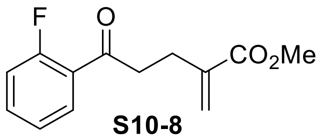

S10-8

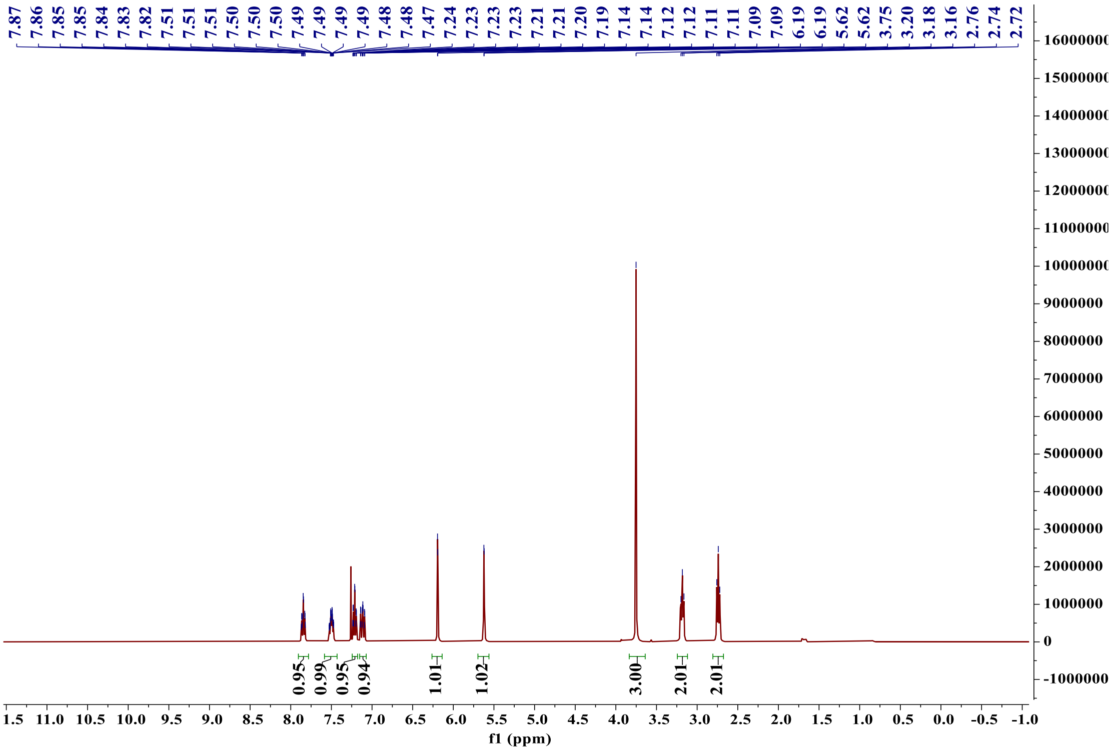

^{1}H NMR (400 MHz, $CDCl_3$) spectrum of **S10-8**

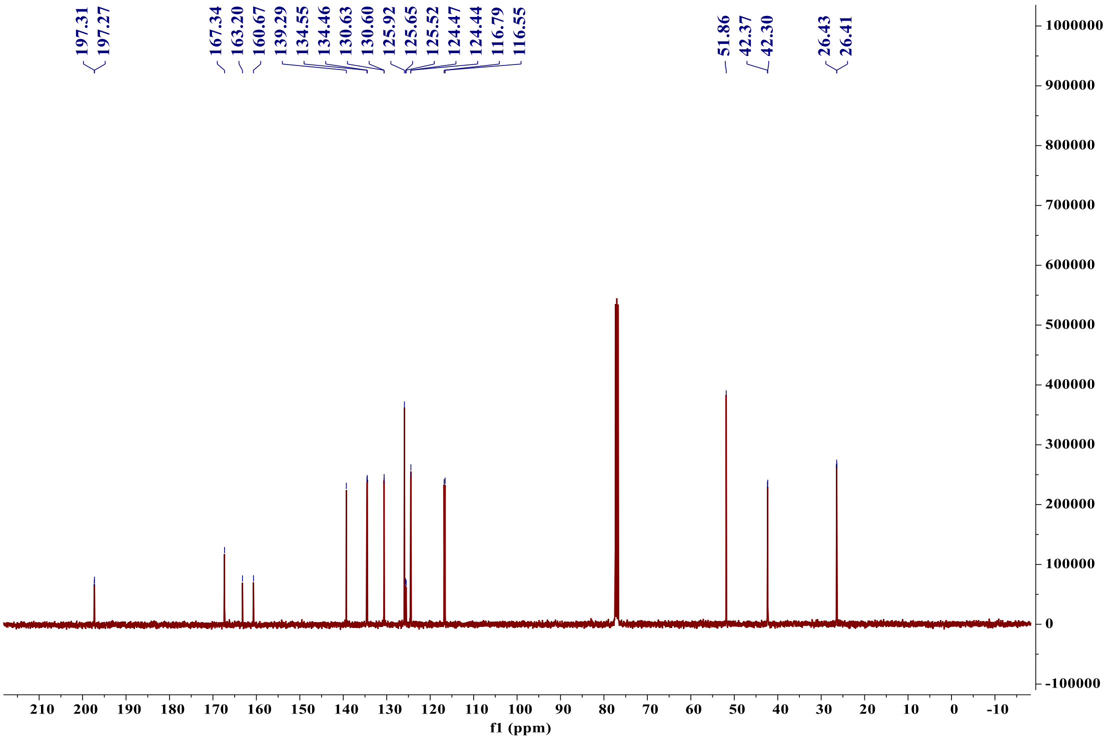

^{13}C NMR (101 MHz, $CDCl_3$) spectrum of **S10-8**

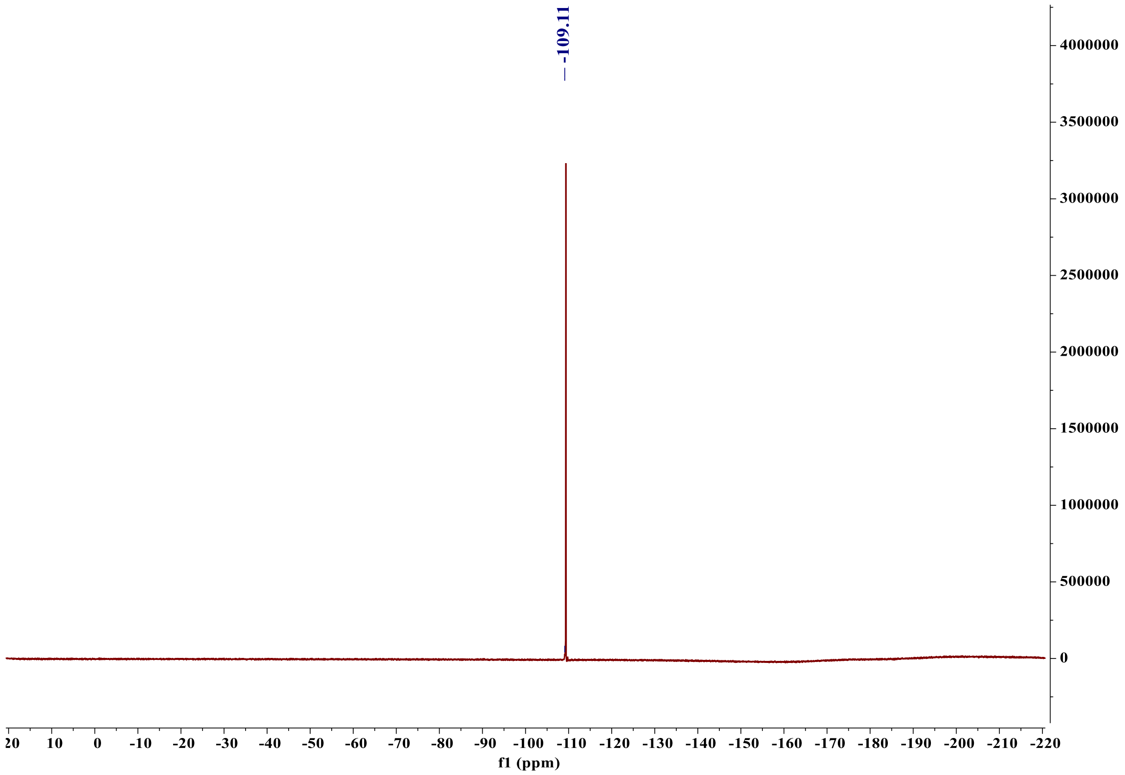

^{19}F NMR (377 MHz, $CDCl_3$) spectrum of **S10-8**

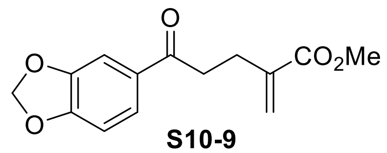

S10-9

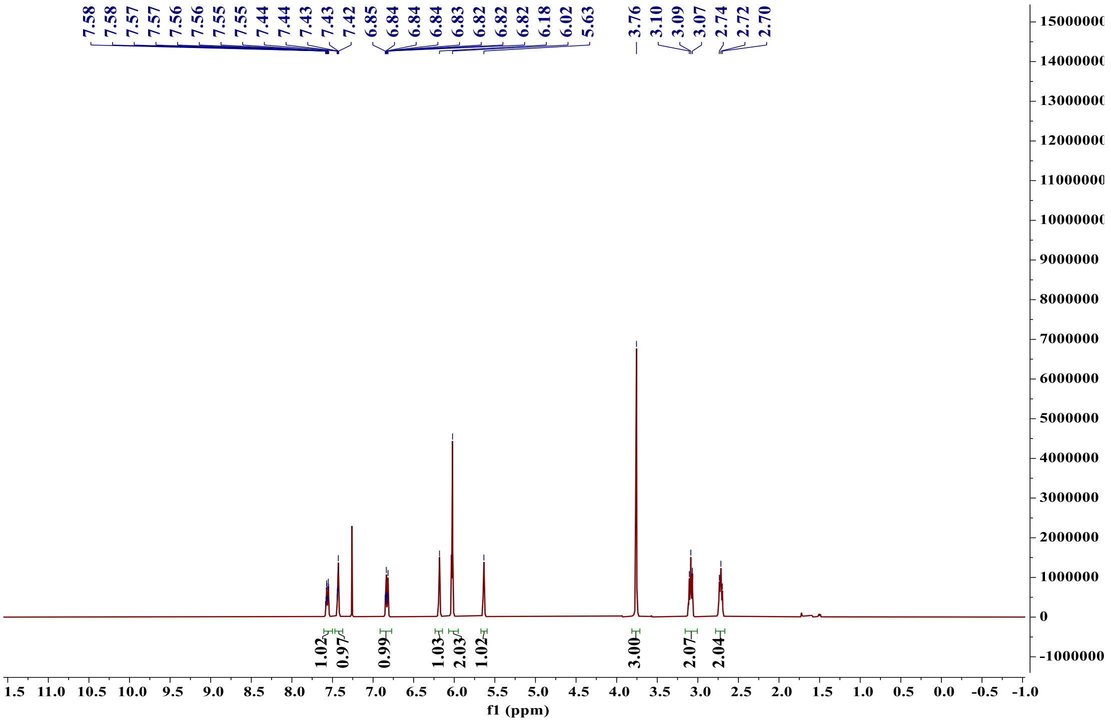

^{1}H NMR (400 MHz, $CDCl_3$) spectrum of **S10-9**

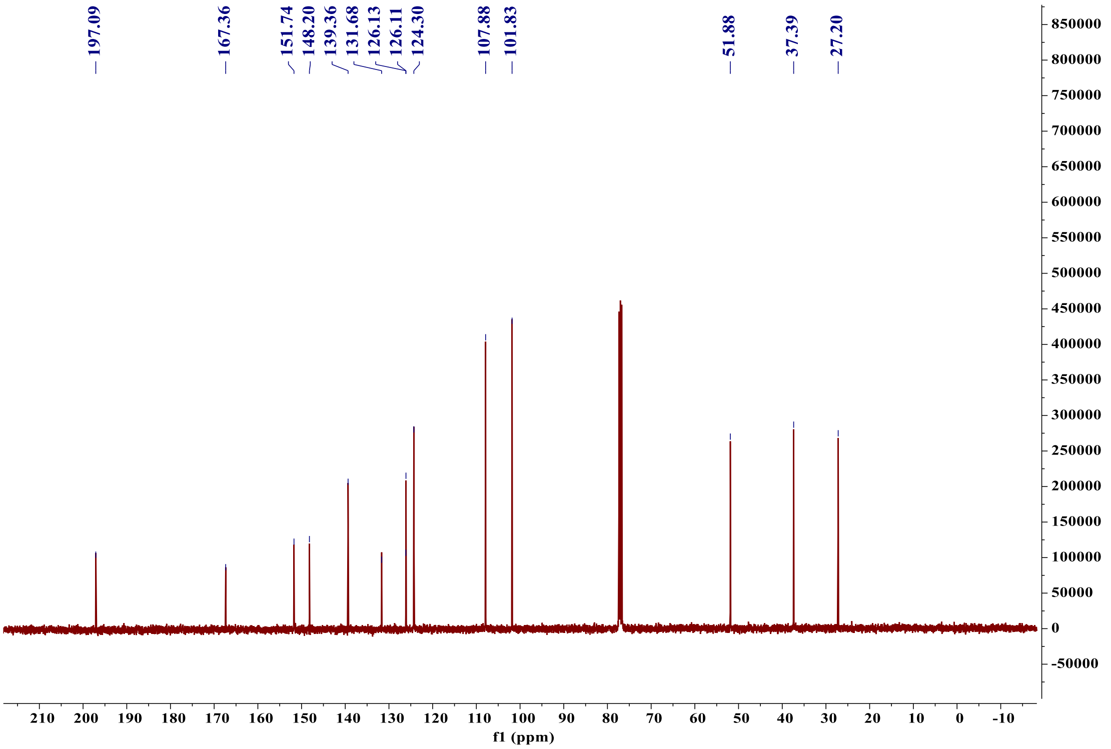

^{13}C NMR (101 MHz, $CDCl_3$) spectrum of **S10-9**

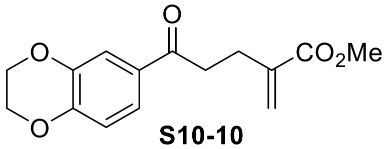

S10-10

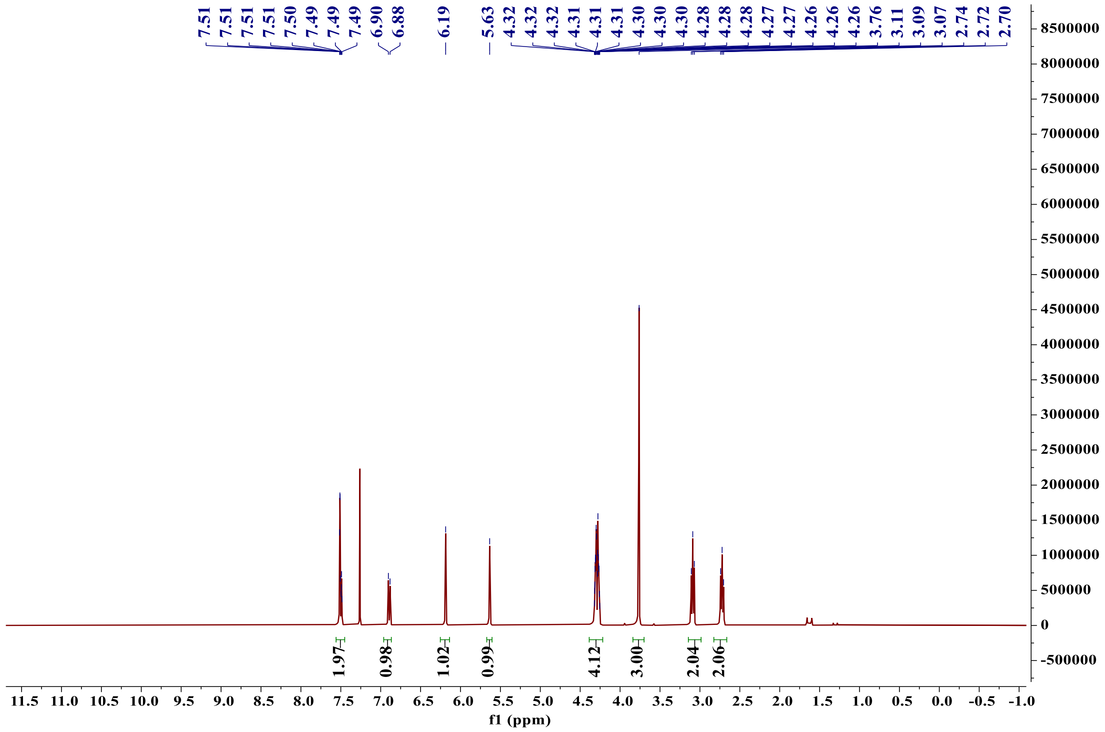

^{1}H NMR (400 MHz, $CDCl_3$) spectrum of **S10-10**

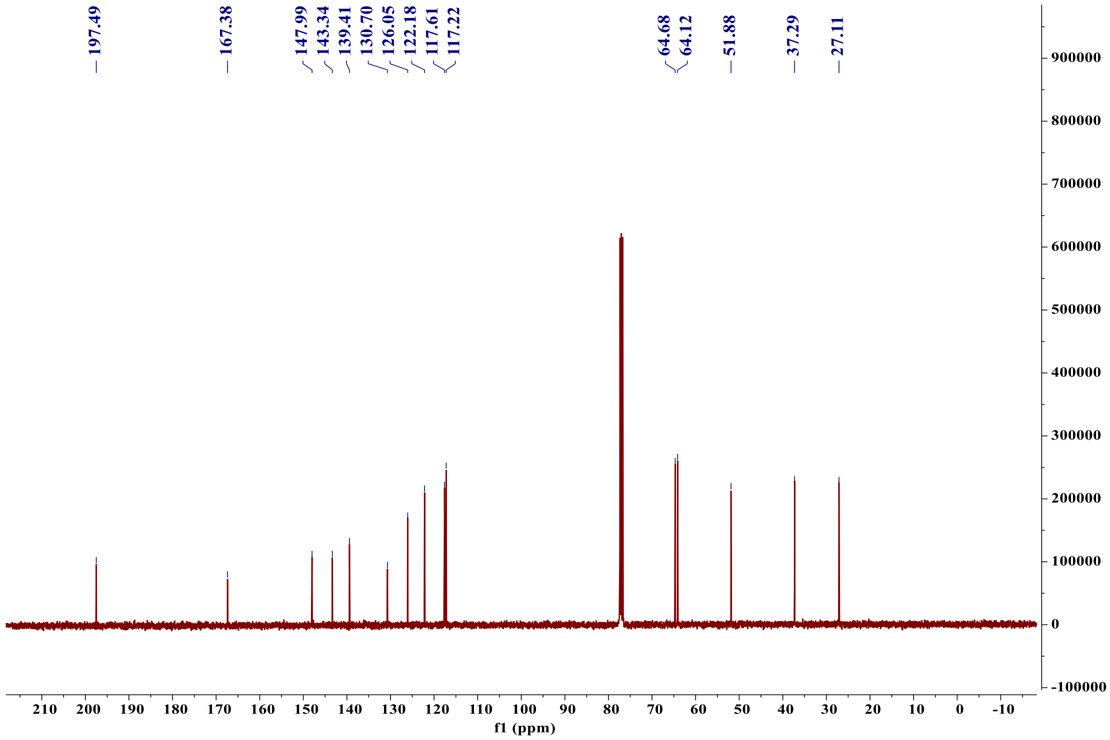

^{13}C NMR (101 MHz, $CDCl_3$) spectrum of **S10-10**

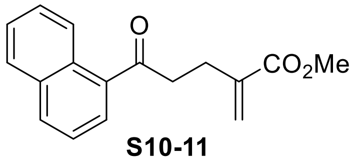

S10-11

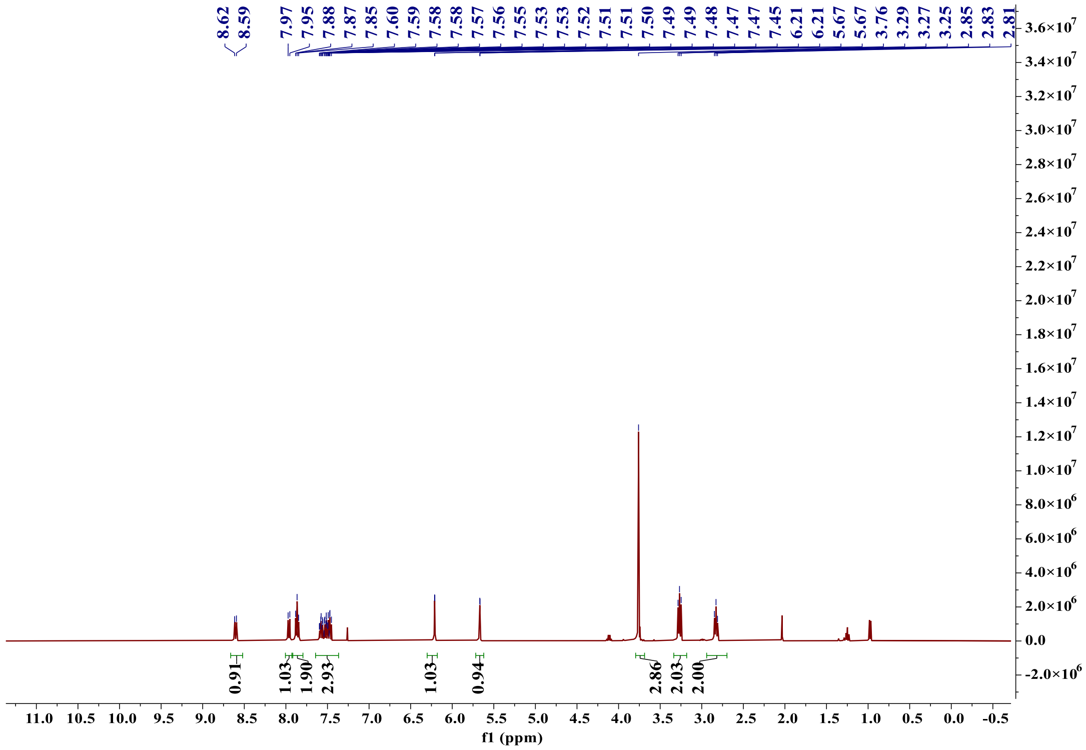

^{1}H NMR (400 MHz, $CDCl_3$) spectrum of **S10-11**

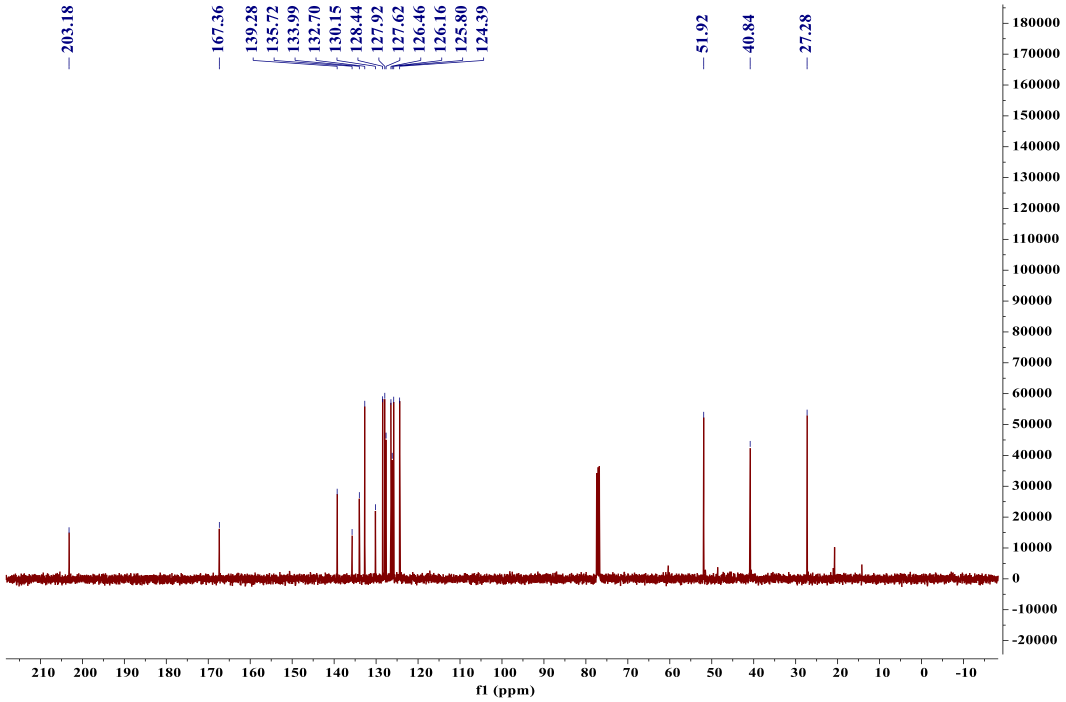

^{13}C NMR (101 MHz, $CDCl_3$) spectrum of **S10-11**

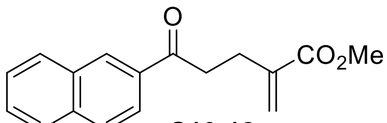

S10-12

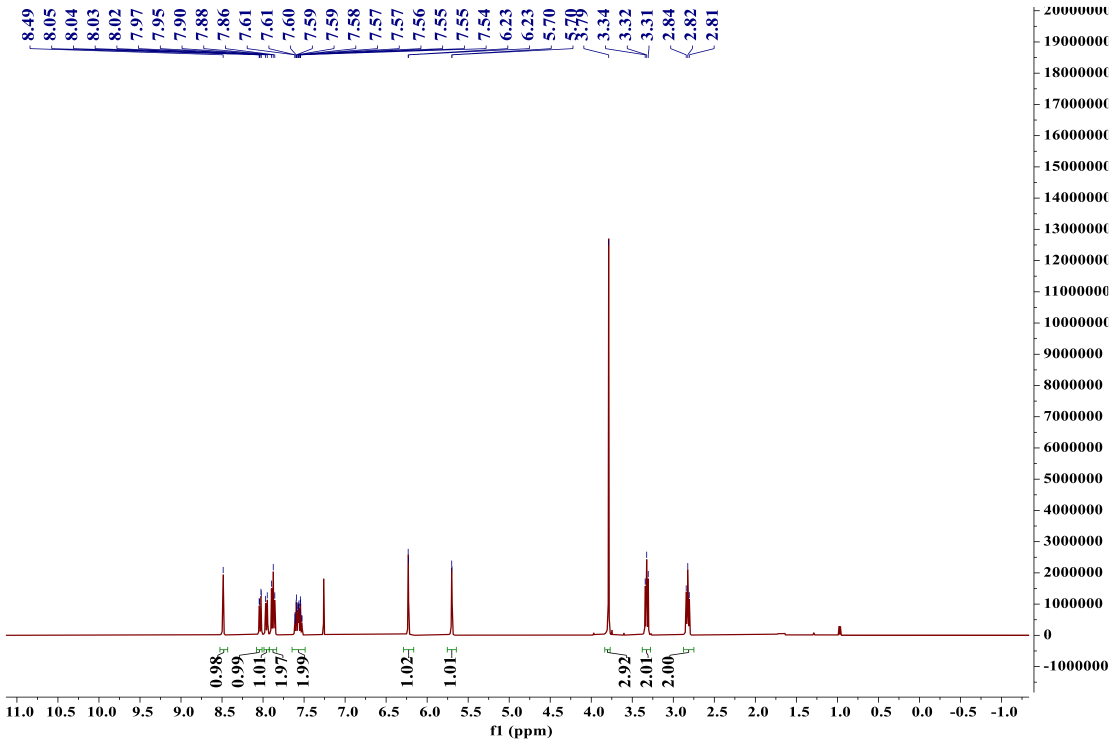

^{1}H NMR (400 MHz, $CDCl_3$) spectrum of **S10-12**

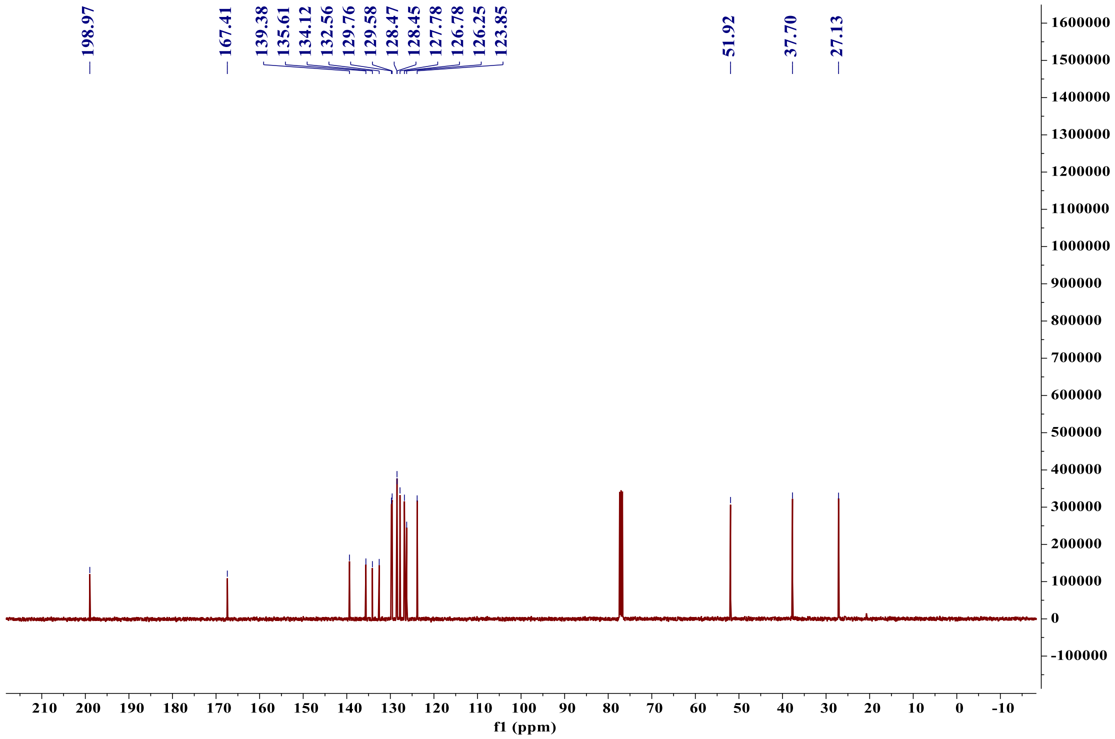

^{13}C NMR (101 MHz, $CDCl_3$) spectrum of **S10-12**

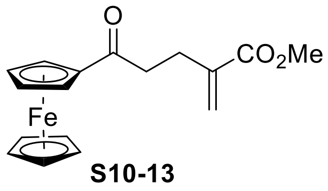

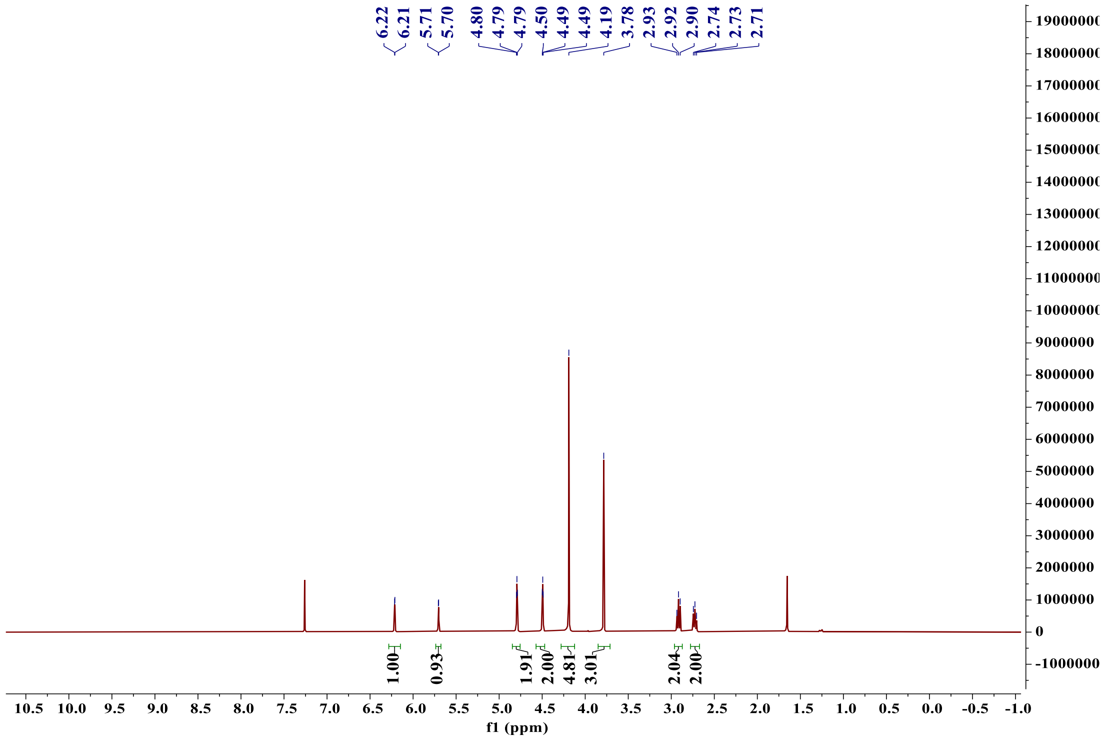

^{1}H NMR (400 MHz, $CDCl_3$) spectrum of **S10-13**

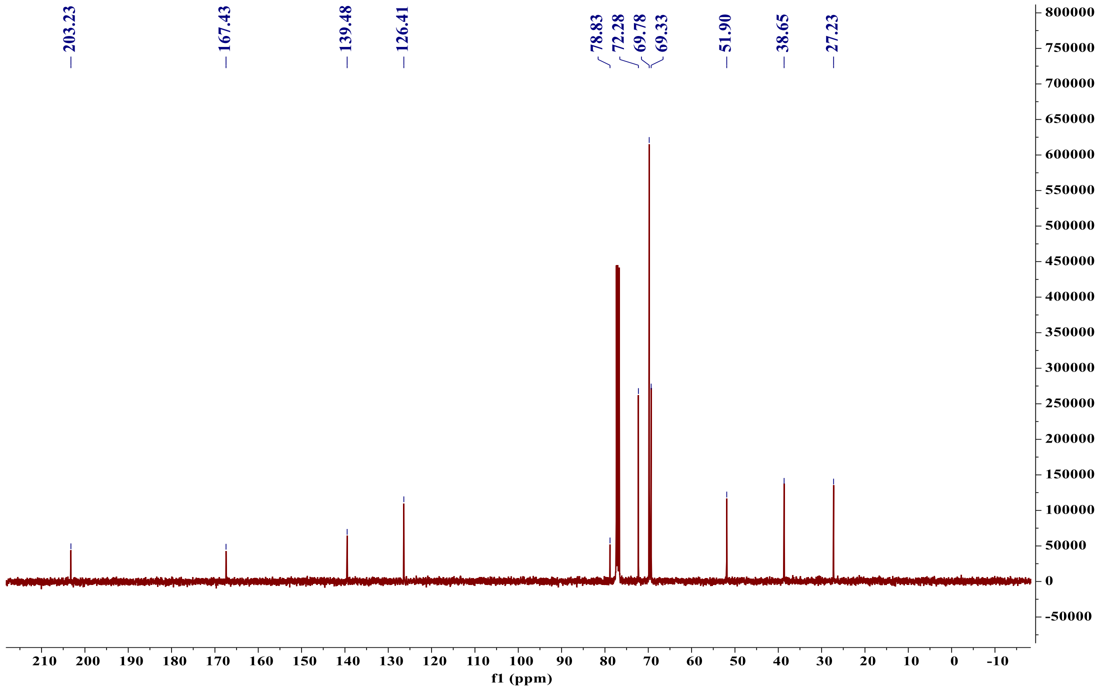

^{13}C NMR (101 MHz, $CDCl_3$) spectrum of **S10-13**

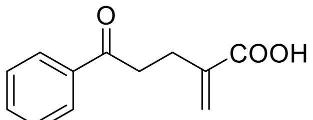

4a

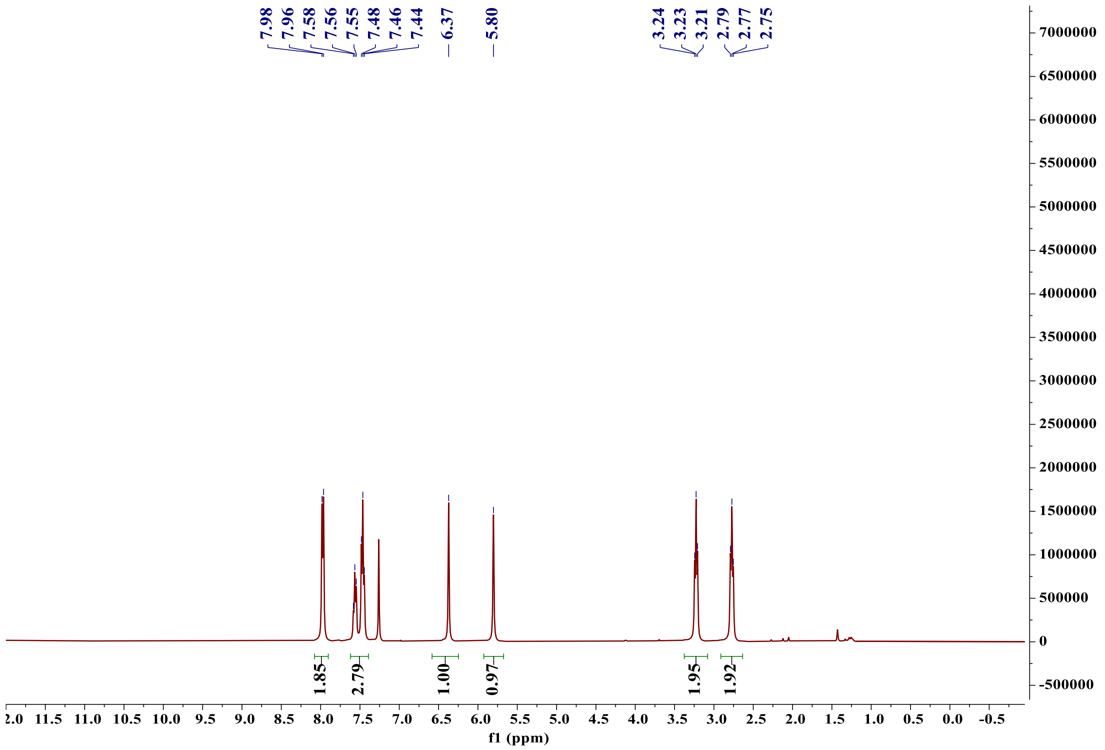

^{1}H NMR (400 MHz, $CDCl_3$) spectrum of **4a**

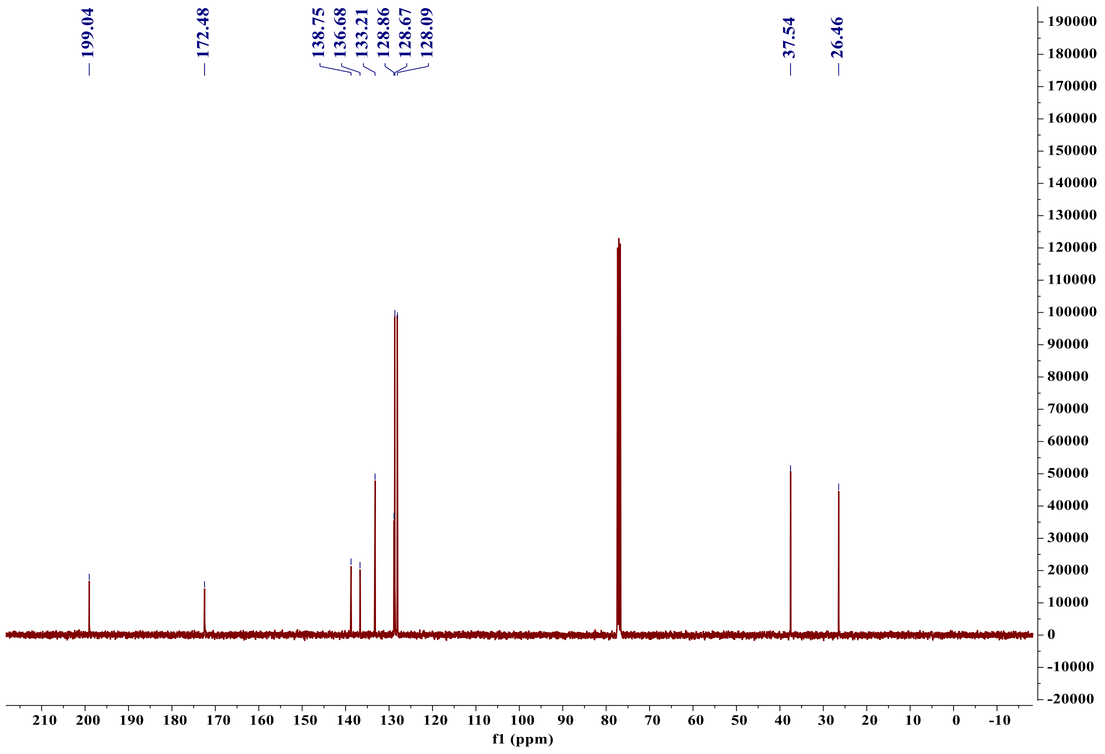

^{13}C NMR (101 MHz, $CDCl_3$) spectrum of **4a**

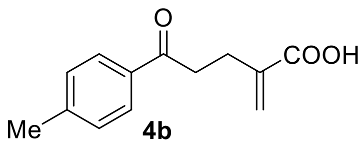

4b

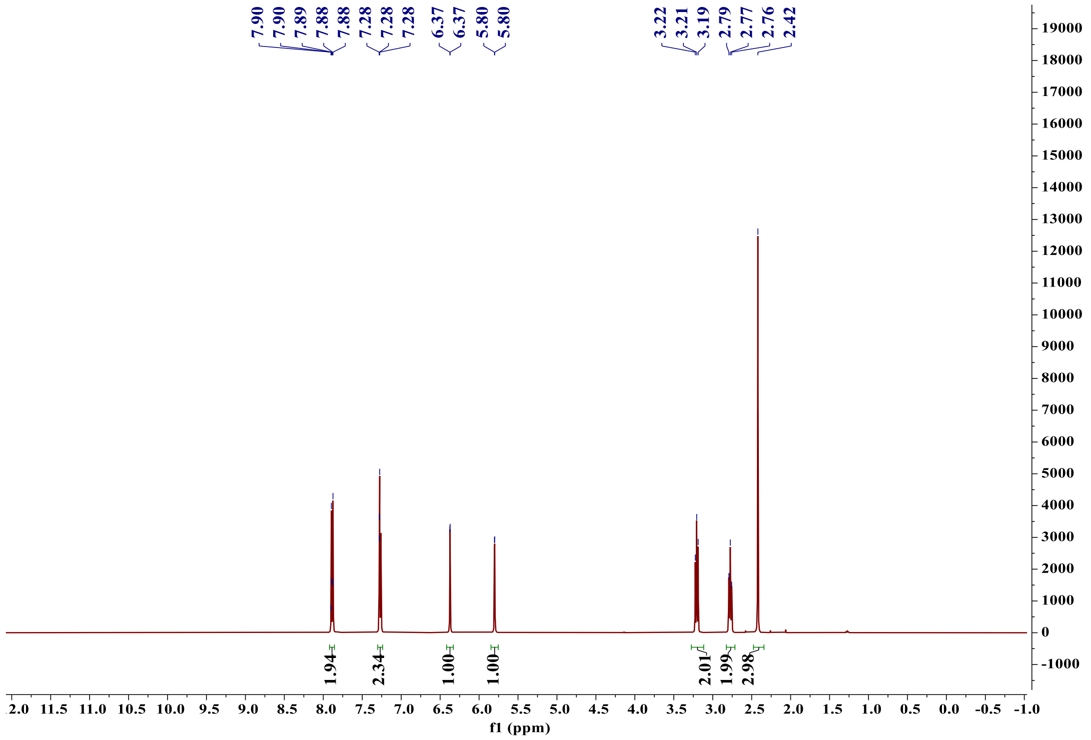

^{1}H NMR (400 MHz, $CDCl_3$) spectrum of **4b**

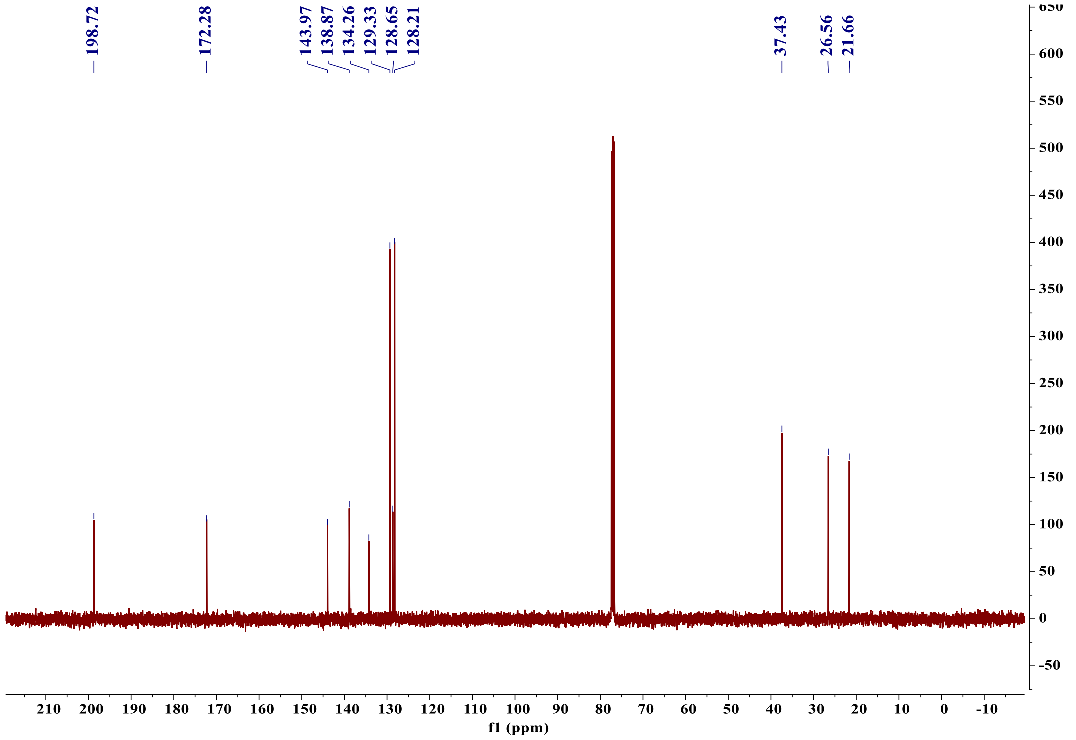

^{13}C NMR (101 MHz, $CDCl_3$) spectrum of **4b**

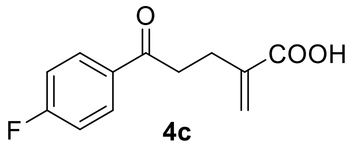

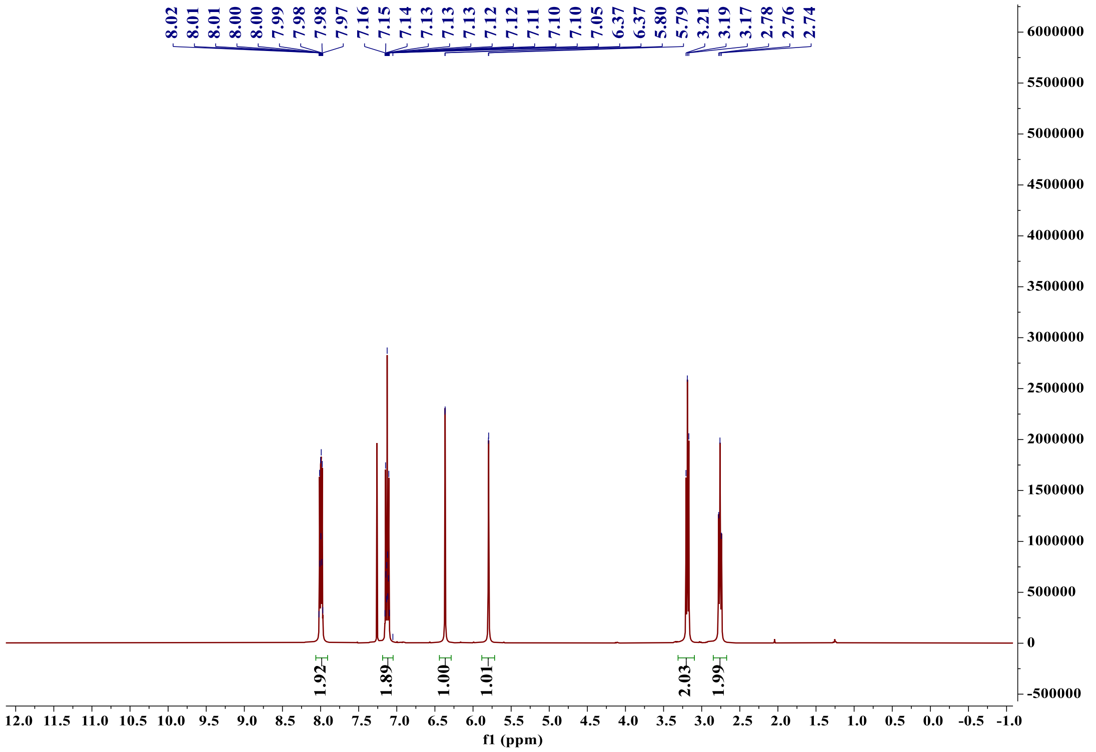

^{1}H NMR (400 MHz, $CDCl_3$) spectrum of **4c**

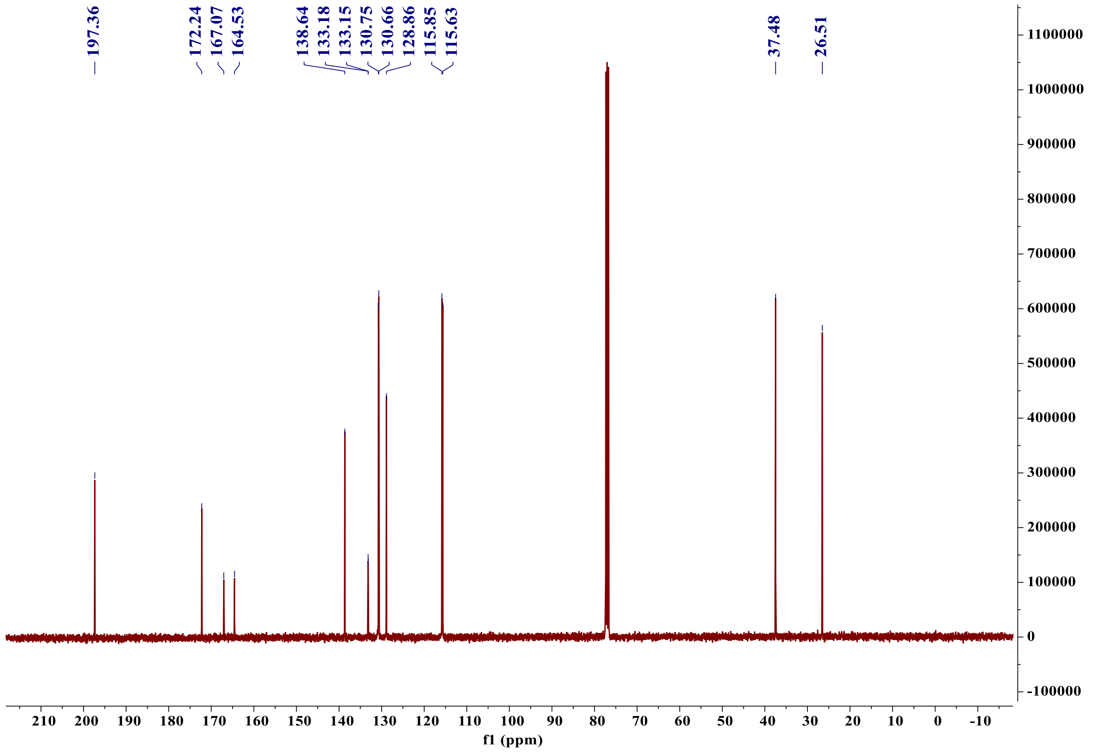

^{13}C NMR (101 MHz, $CDCl_3$) spectrum of **4c**

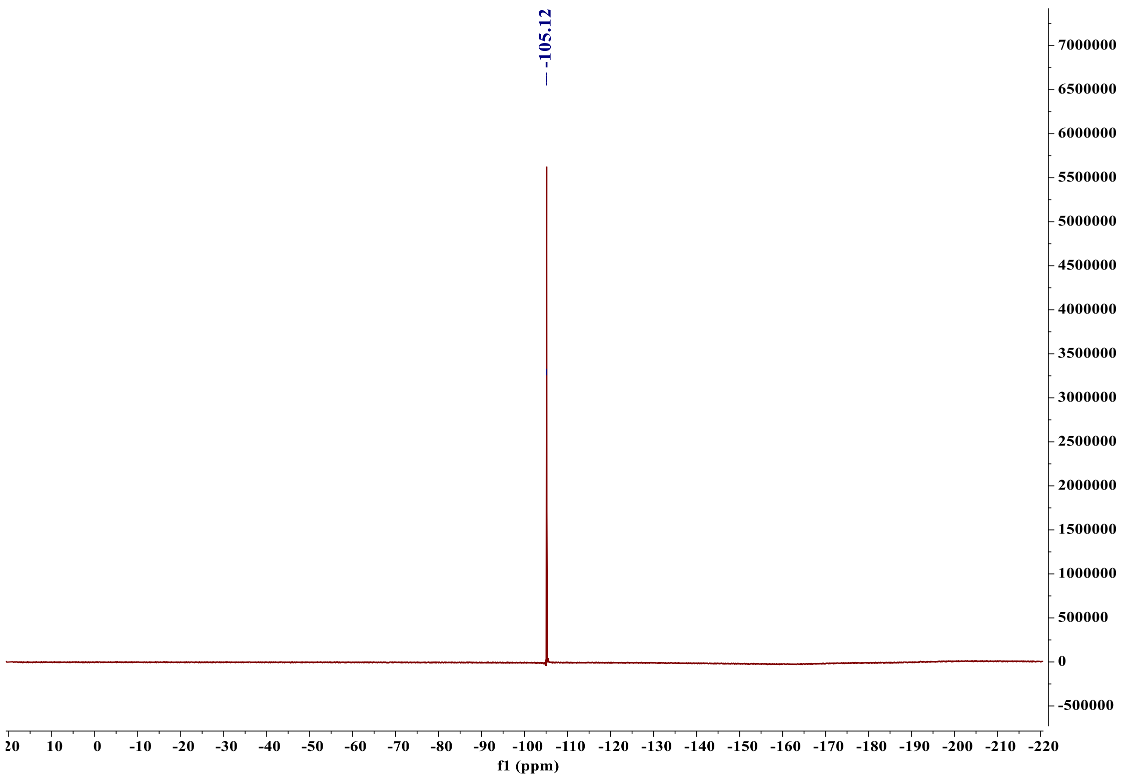

^{19}F NMR (377 MHz, $CDCl_3$) spectrum of **4c**

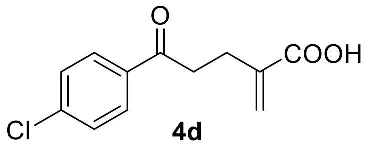

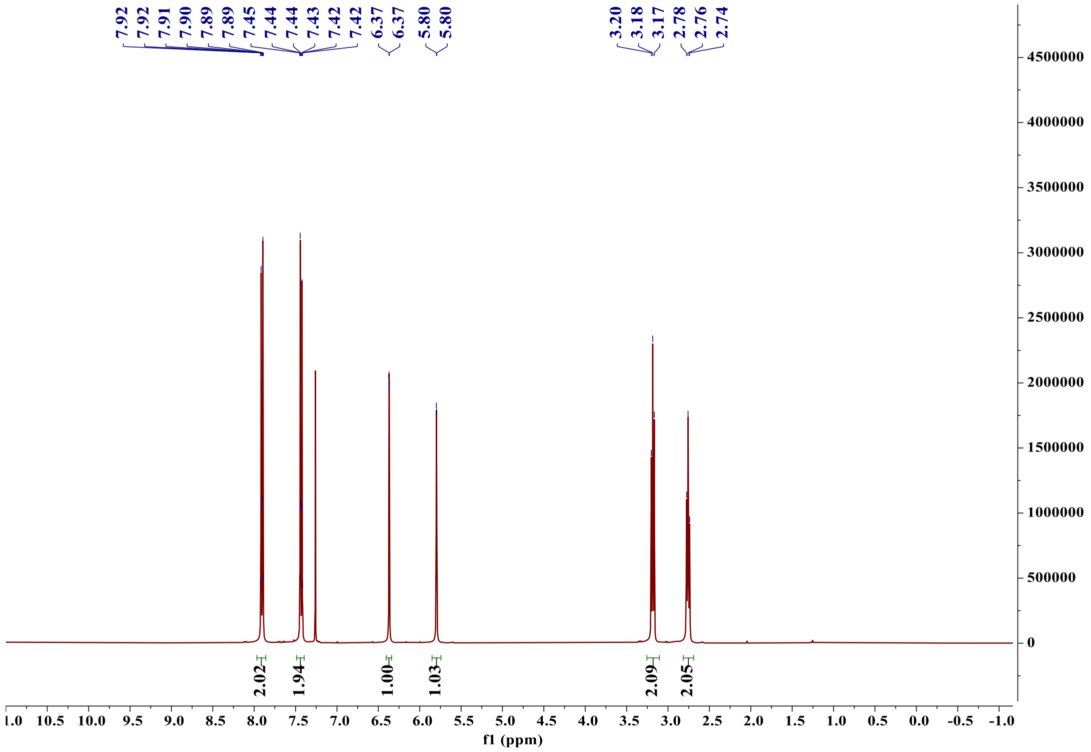

^{1}H NMR (400 MHz, $CDCl_3$) spectrum of **4d**

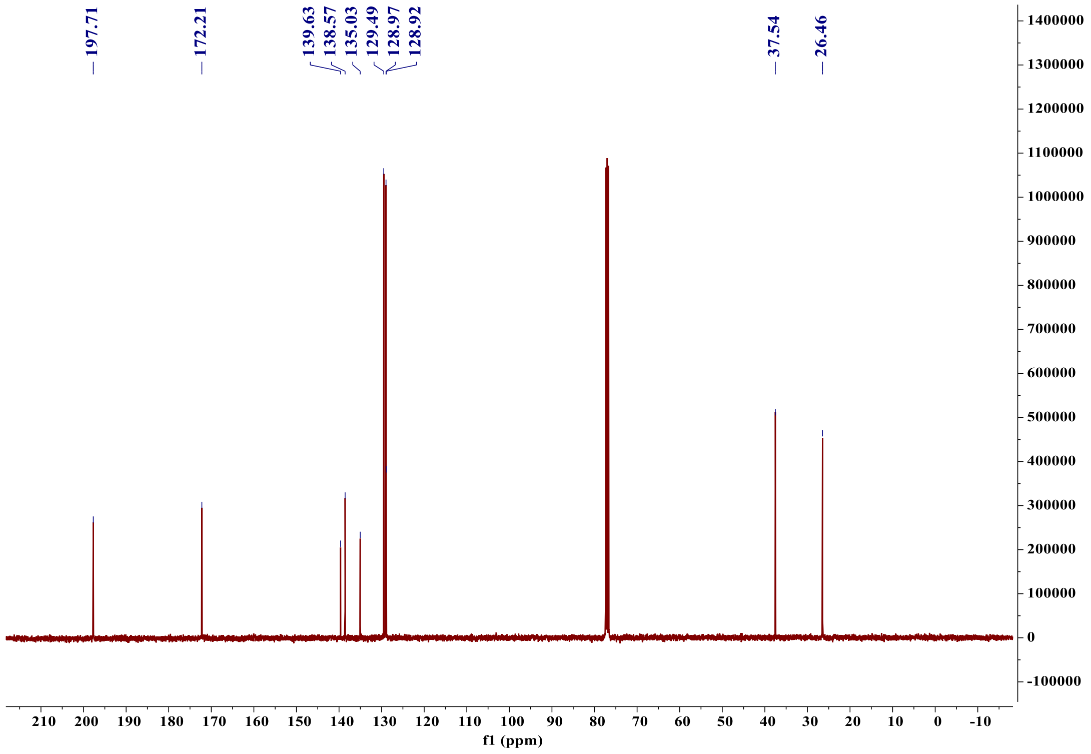

^{13}C NMR (101 MHz, $CDCl_3$) spectrum of **4d**

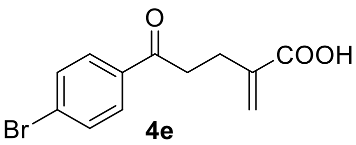

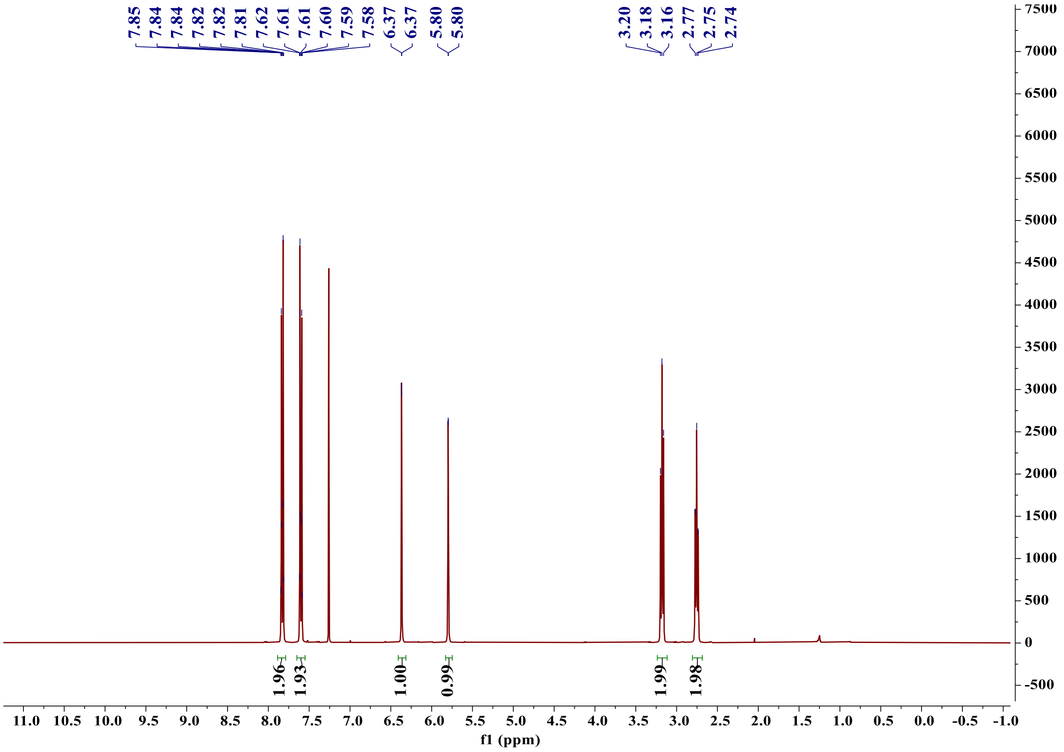

^{1}H NMR (400 MHz, CDCl$_3$) spectrum of **4e**

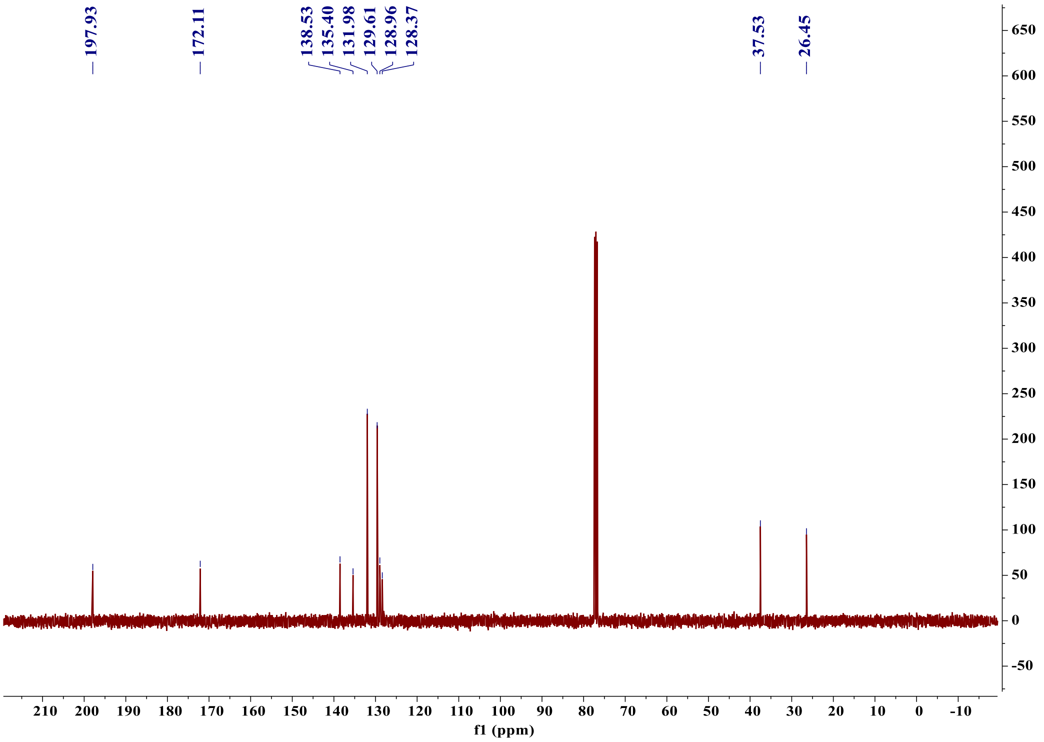

^{13}C NMR (101 MHz, CDCl$_3$) spectrum of **4e**

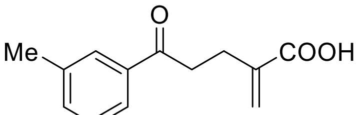

4f

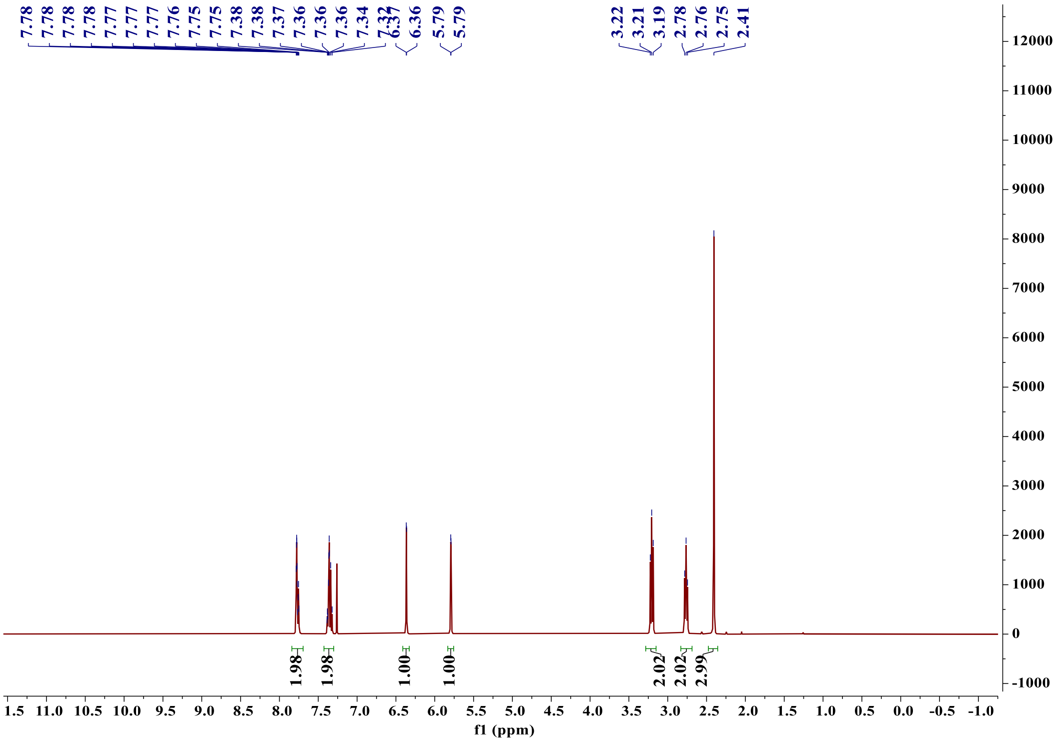

^{1}H NMR (400 MHz, $CDCl_3$) spectrum of **4f**

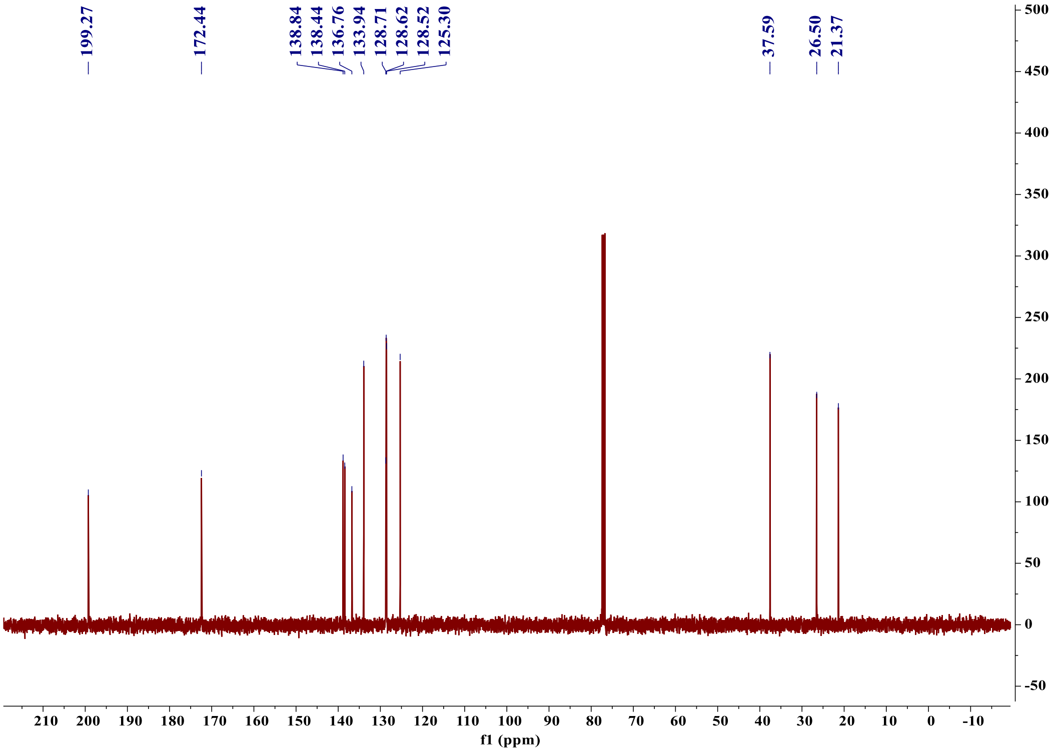

^{13}C NMR (101 MHz, $CDCl_3$) spectrum of **4f**

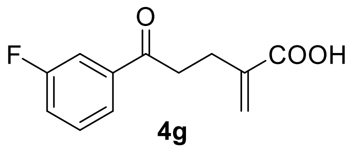

4g

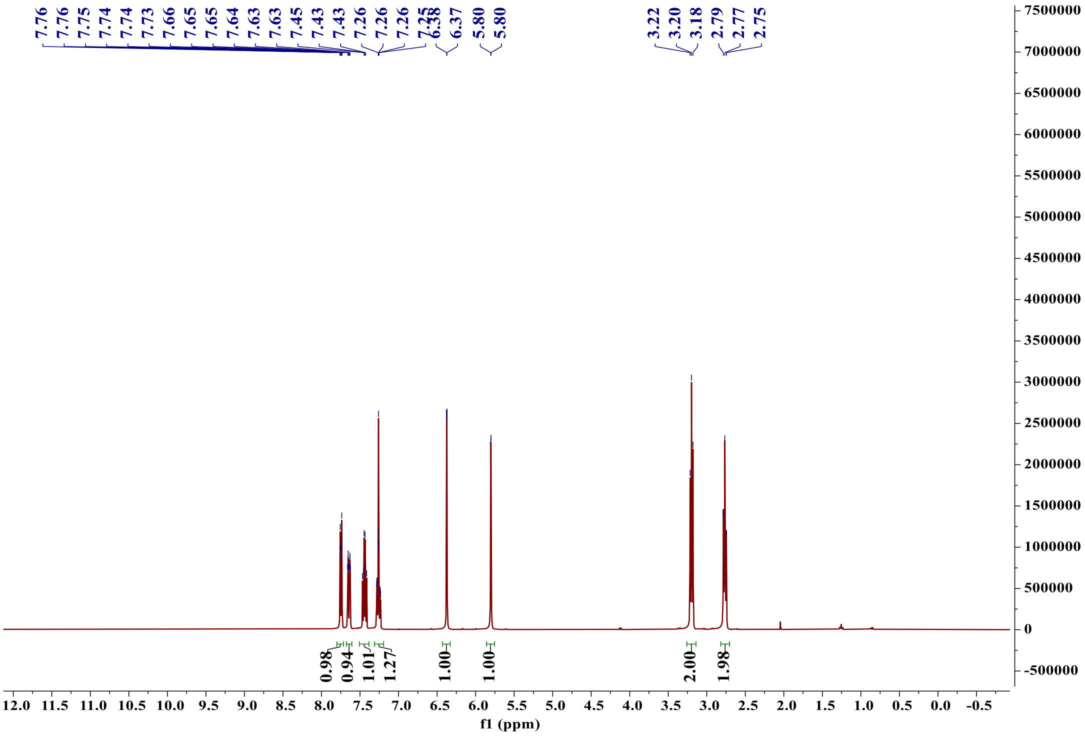

^{1}H NMR (400 MHz, $CDCl_3$) spectrum of **4g**

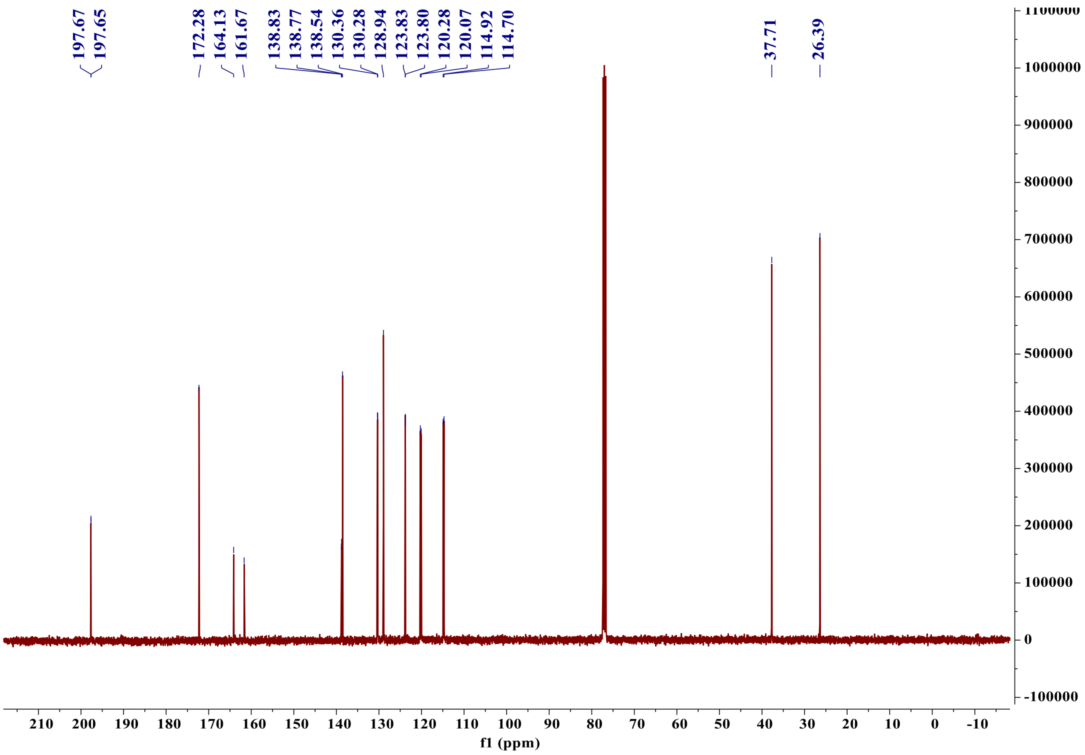

^{13}C NMR (101 MHz, $CDCl_3$) spectrum of **4g**

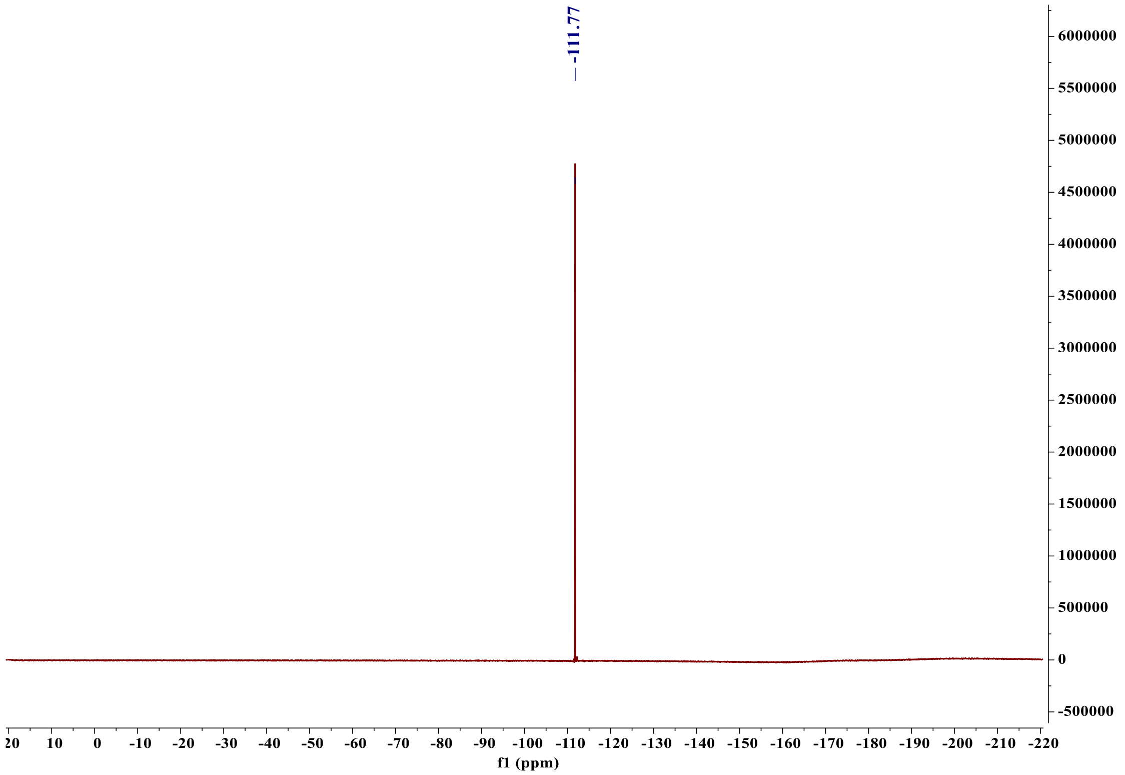

^{19}F NMR (377 MHz, $CDCl_3$) spectrum of **4g**

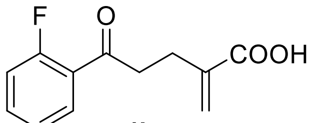

4h

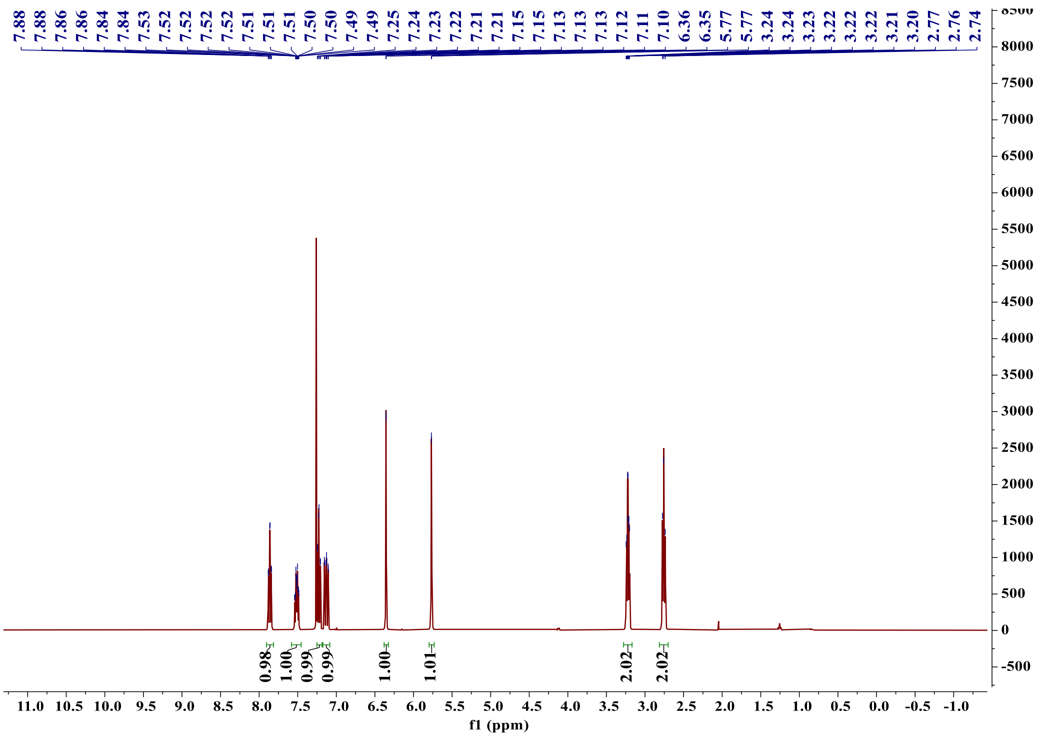

^{1}H NMR (400 MHz, $CDCl_3$) spectrum of **4h**

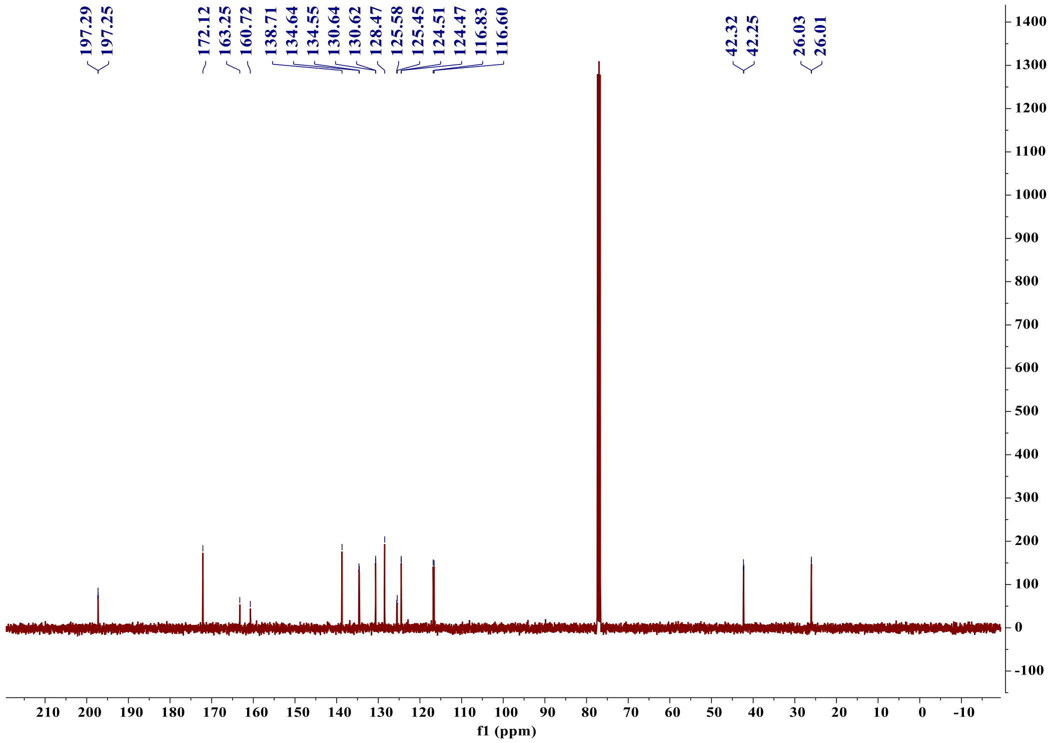

^{13}C NMR (101 MHz, $CDCl_3$) spectrum of **4h**

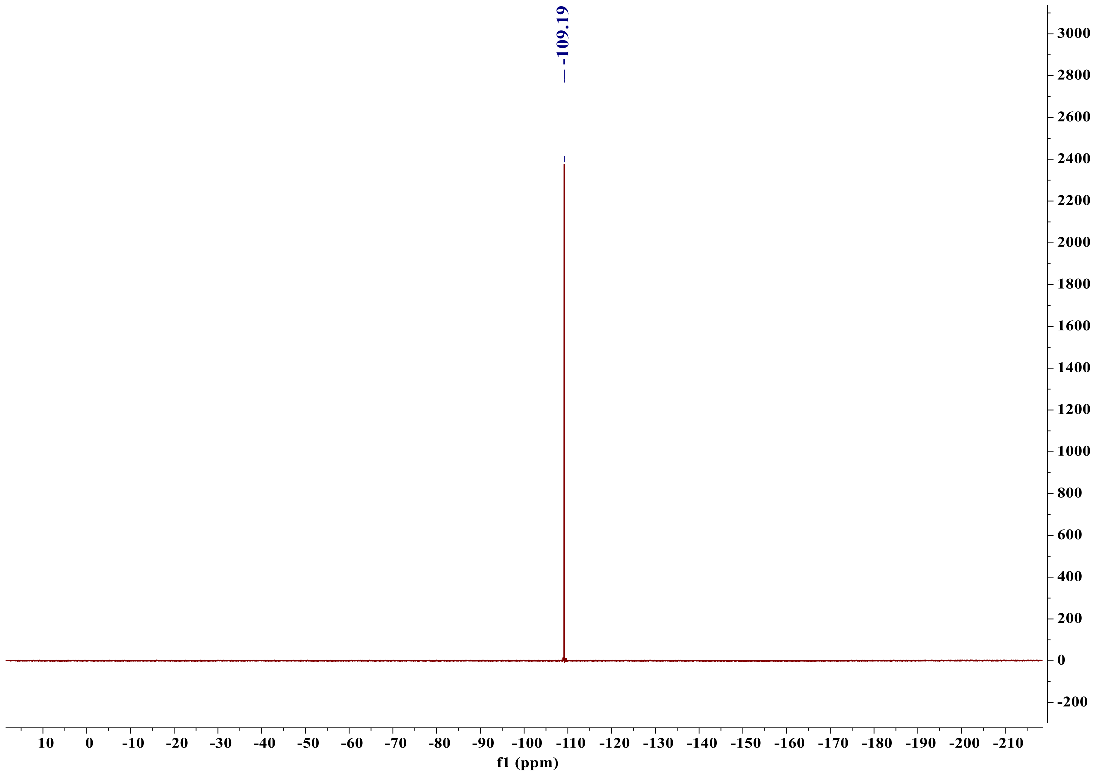

^{19}F NMR (377 MHz, $CDCl_3$) spectrum of **4h**

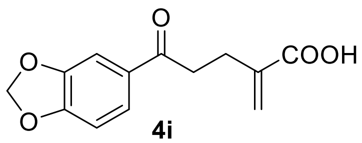

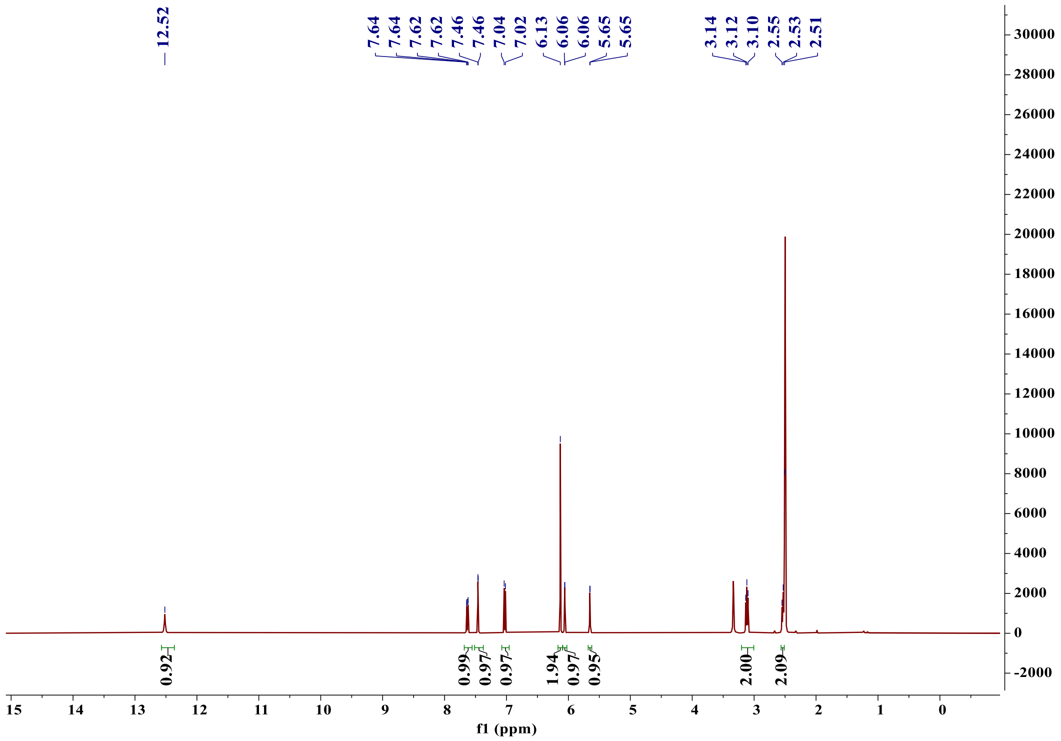

^{1}H NMR (400 MHz, DMSO-d_6) spectrum of **4i**

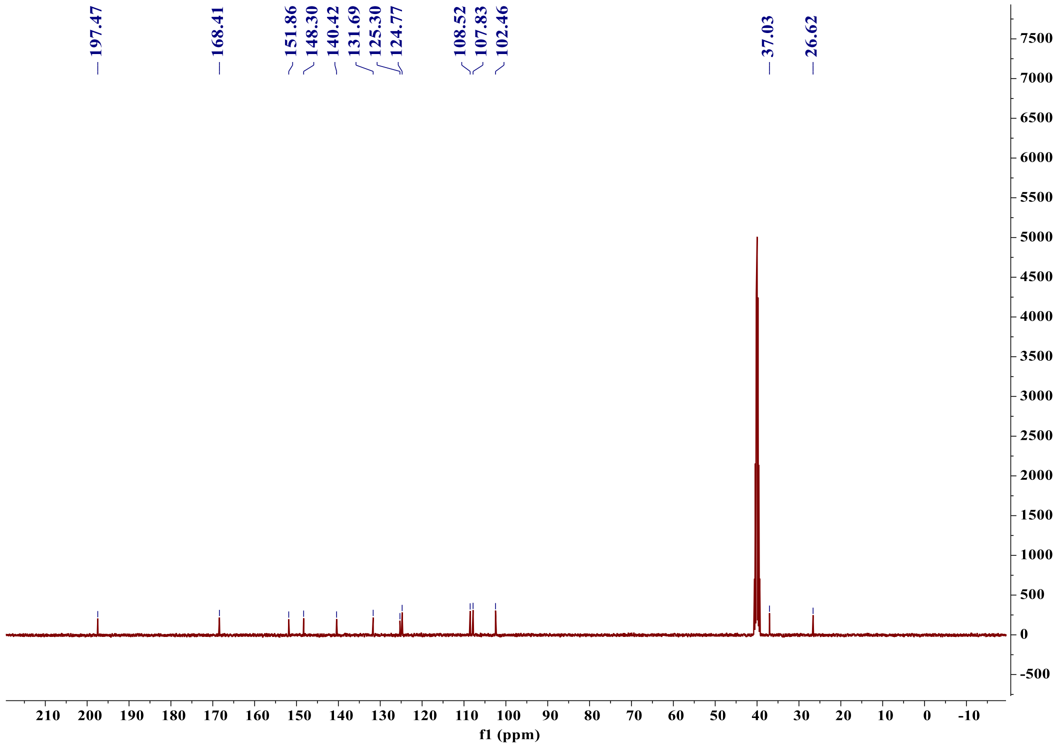

^{13}C NMR (101 MHz, DMSO-d_6) spectrum of **4i**

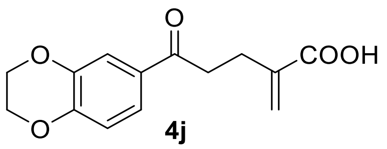

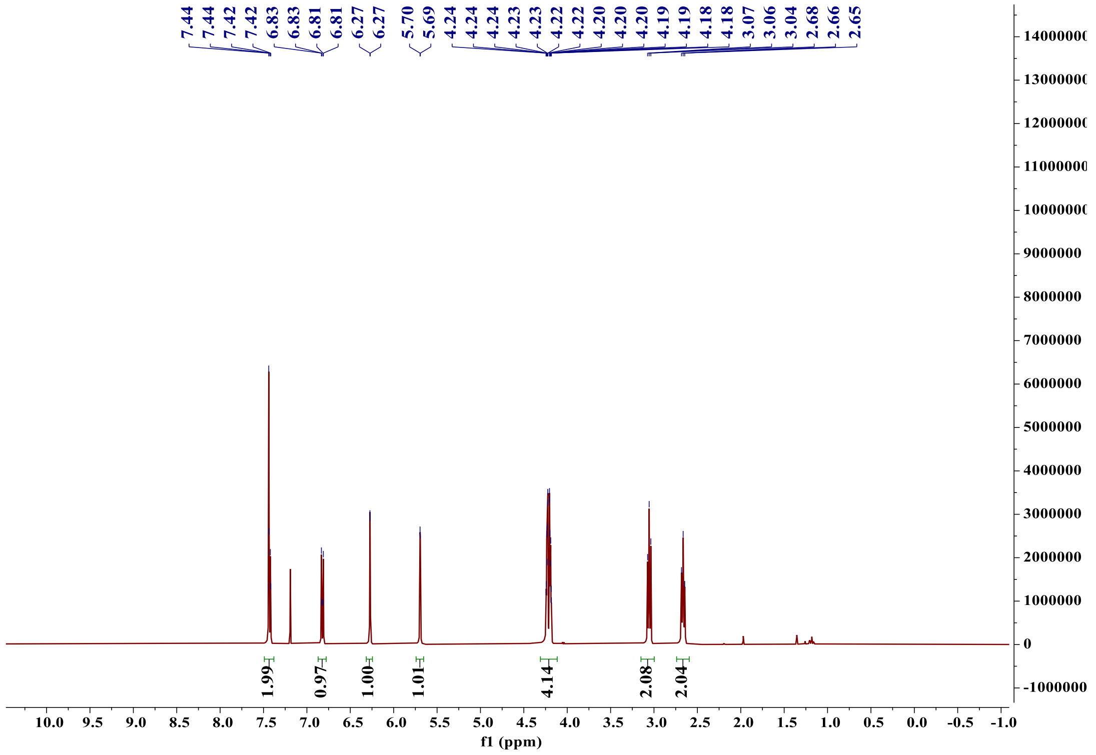

^{1}H NMR (400 MHz, $CDCl_3$) spectrum of **4j**

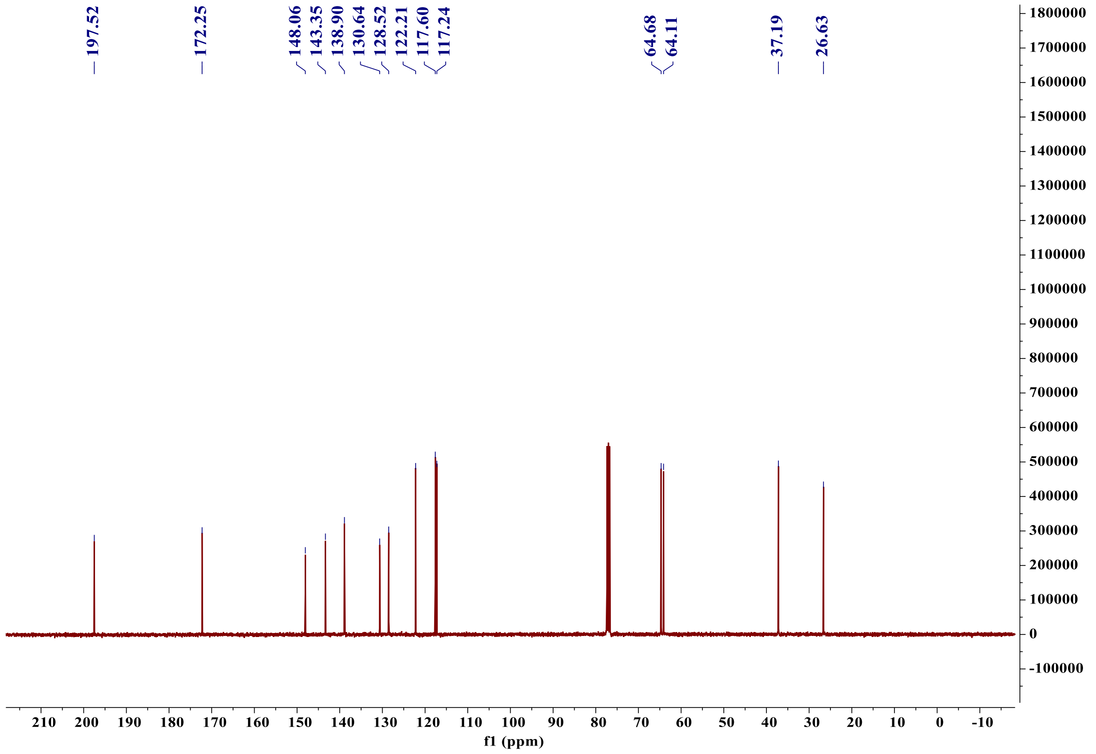

^{13}C NMR (101 MHz, $CDCl_3$) spectrum of **4j**

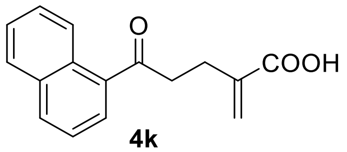

4k

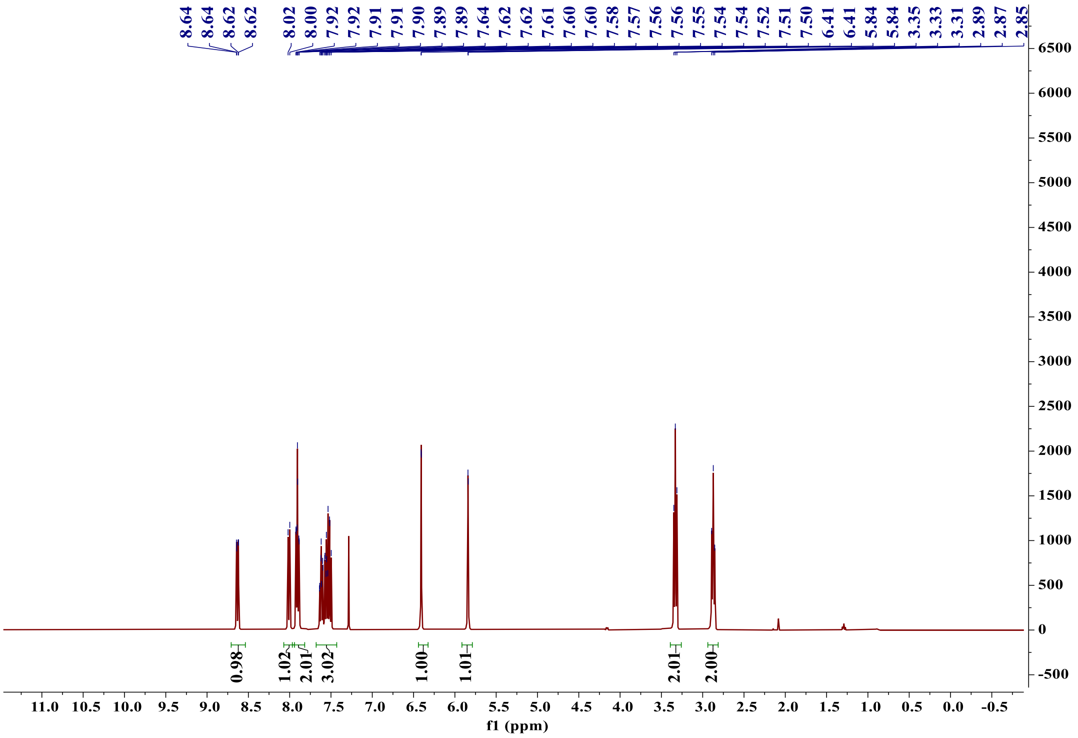

^{1}H NMR (400 MHz, CDCl₃) spectrum of **4k**

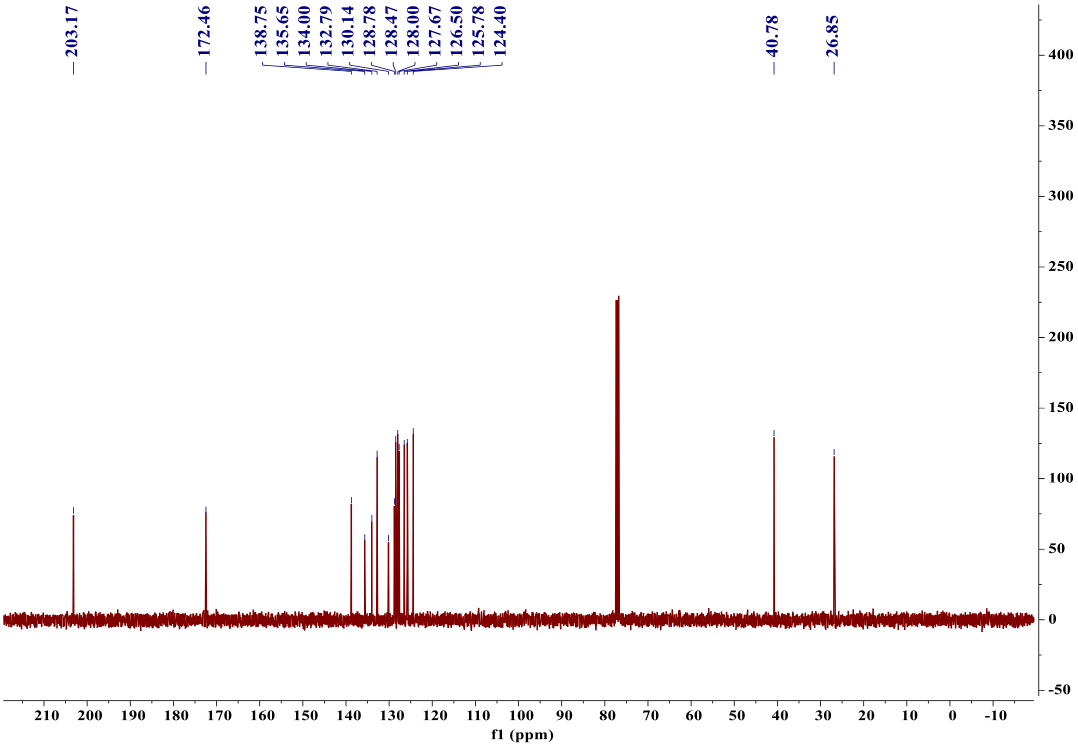

^{13}C NMR (101 MHz, CDCl₃) spectrum of **4k**

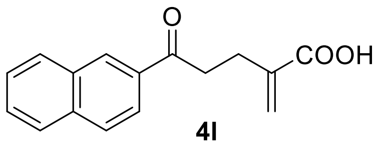

4l

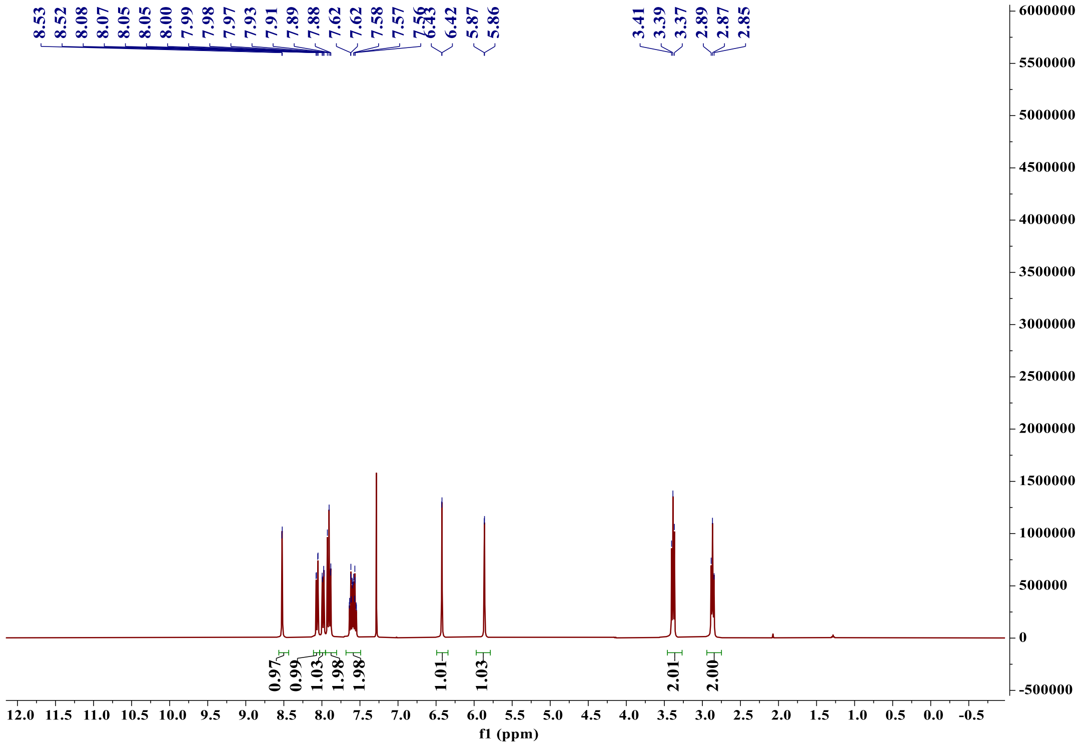

^{1}H NMR (400 MHz, $CDCl_3$) spectrum of **4l**

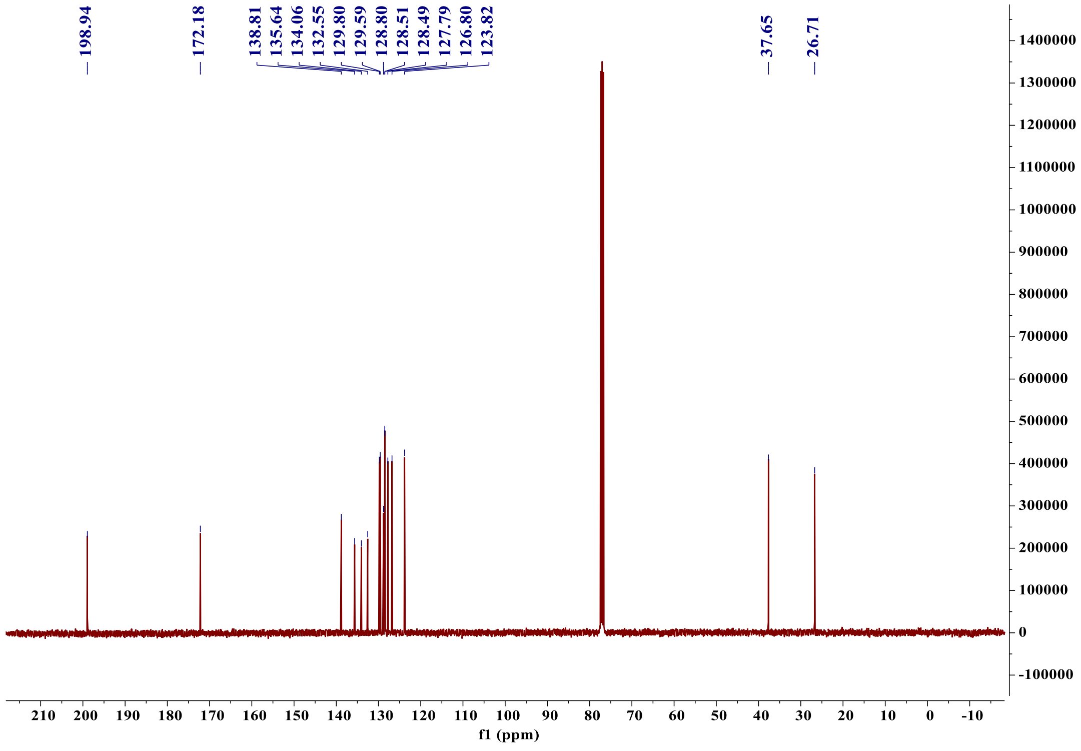

^{13}C NMR (101 MHz, $CDCl_3$) spectrum of **4l**

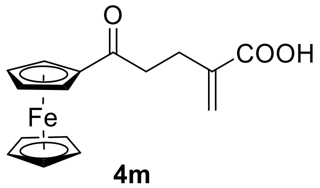

4m

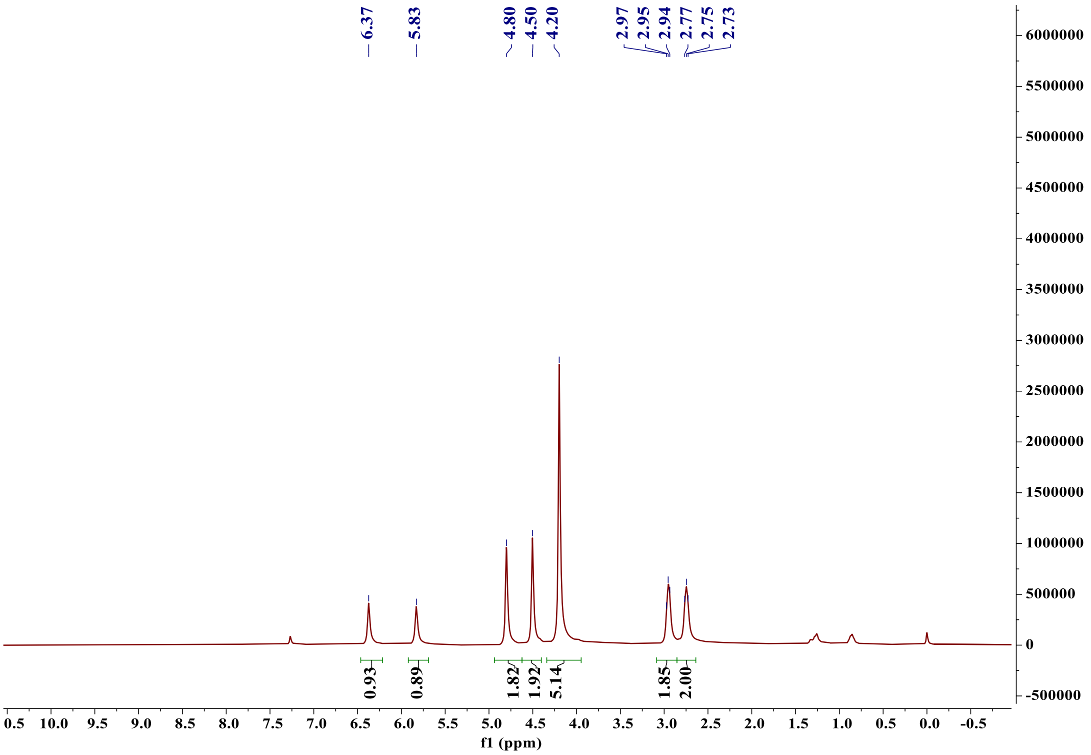

^{1}H NMR (400 MHz, $CDCl_3$) spectrum of **4m**

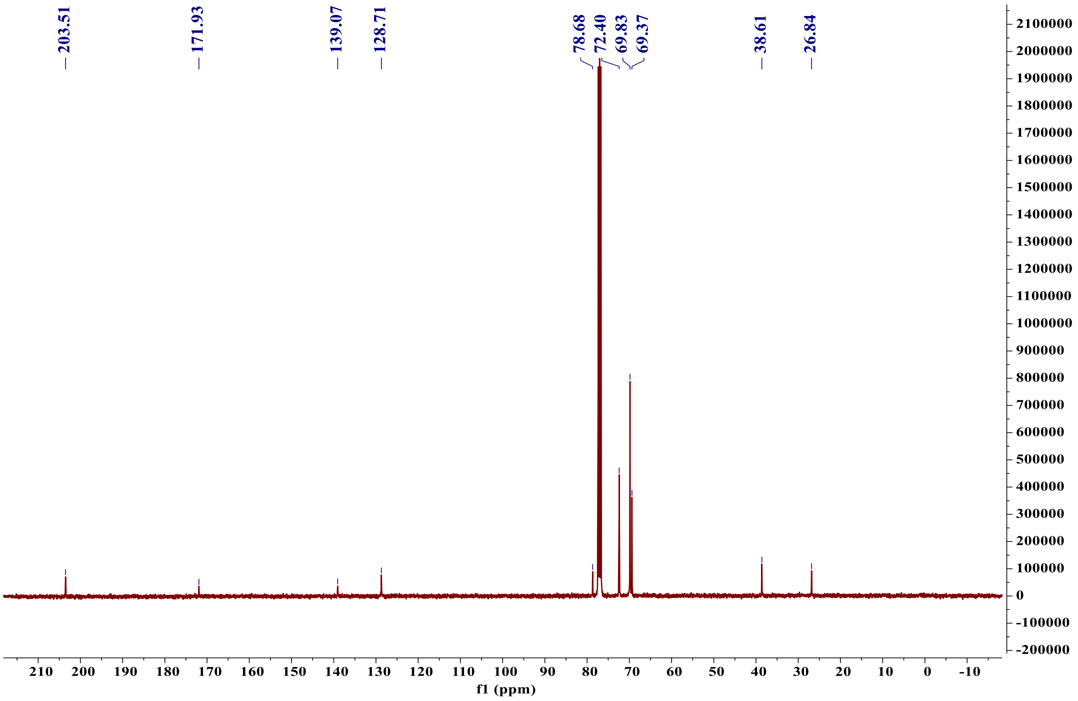

^{13}C NMR (101 MHz, $CDCl_3$) spectrum of **4m**

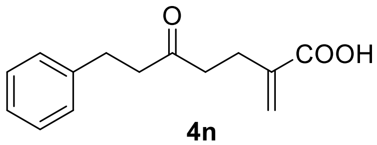

4n

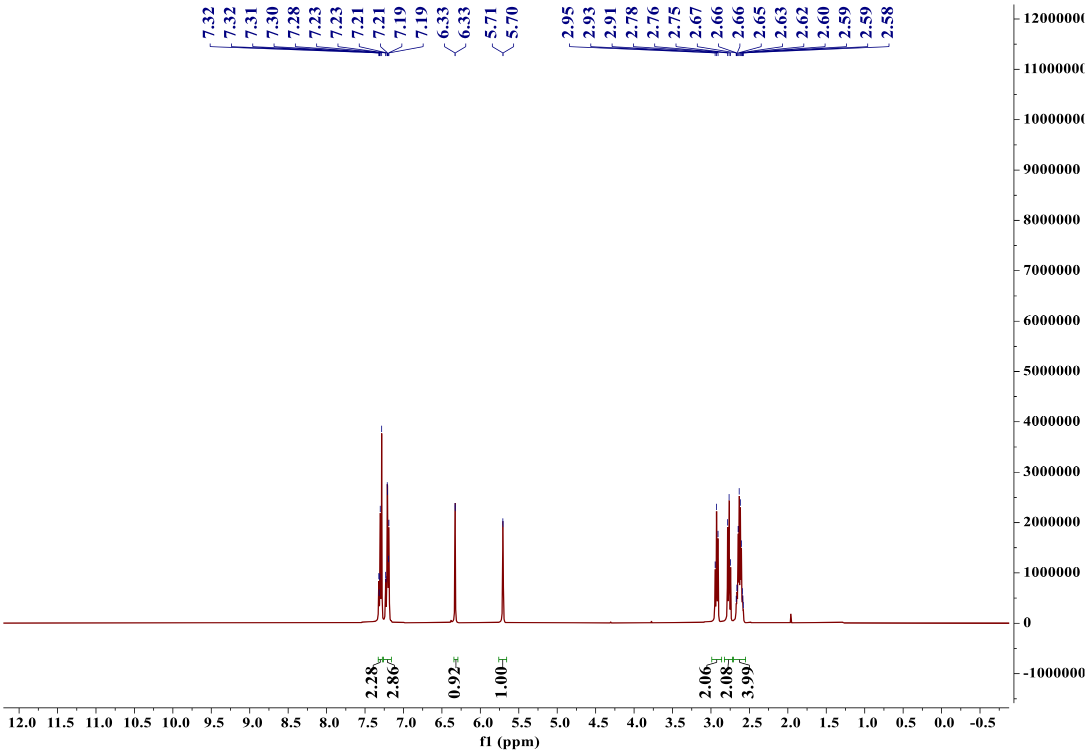

^{1}H NMR (400 MHz, $CDCl_3$) spectrum of **4n**

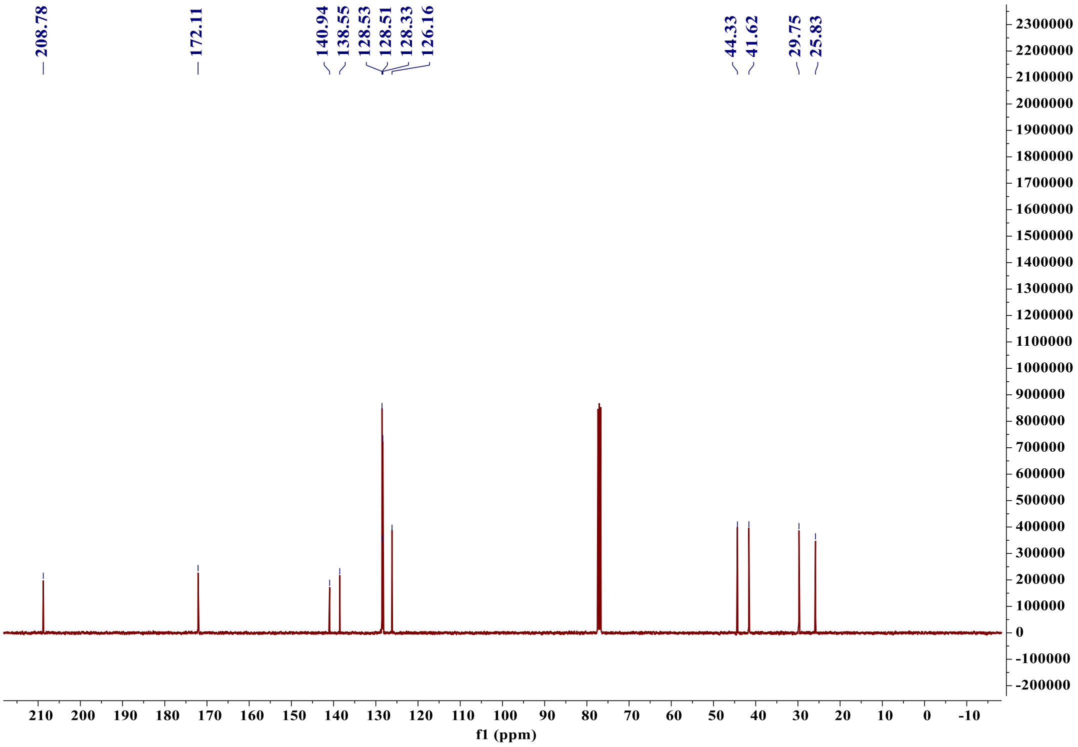

^{13}C NMR (101 MHz, $CDCl_3$) spectrum of **4n**

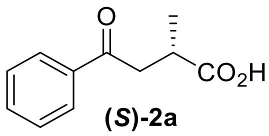

(S)-2a

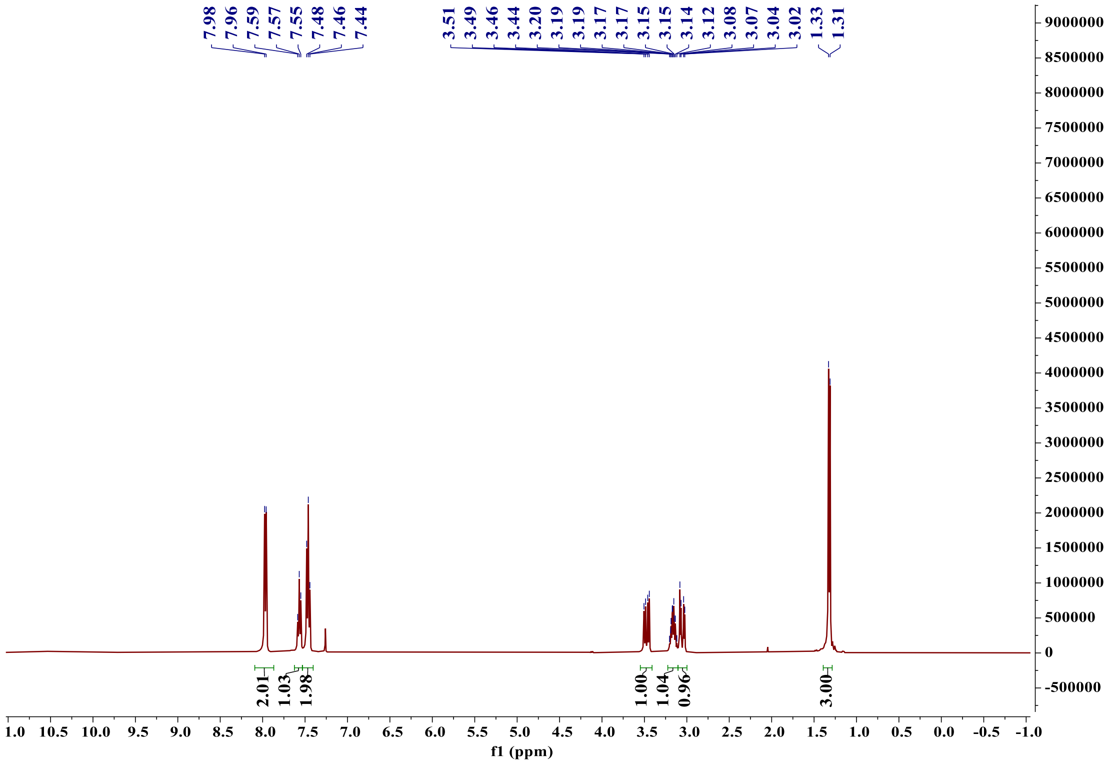

^{1}H NMR (400 MHz, $CDCl_3$) spectrum of **(*S*)-2a**

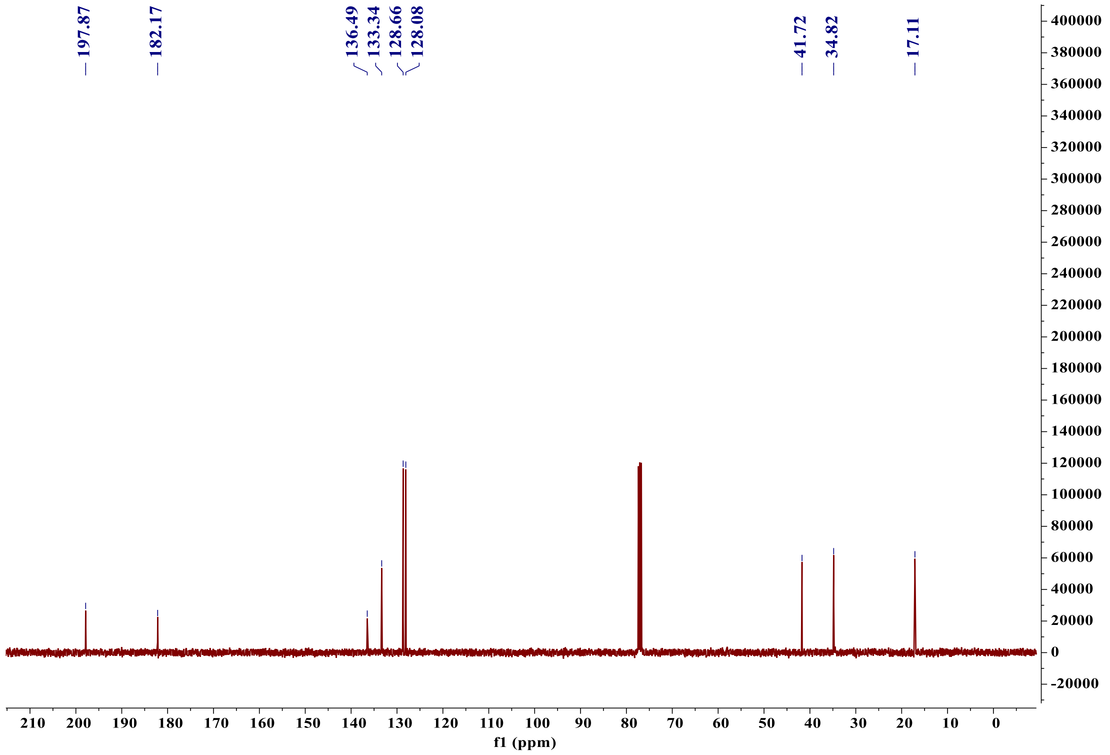

^{13}C NMR (101 MHz, $CDCl_3$) spectrum of **(*S*)-2a**

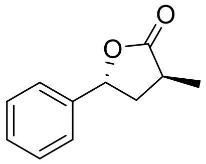

(*S*,*R*)-3a

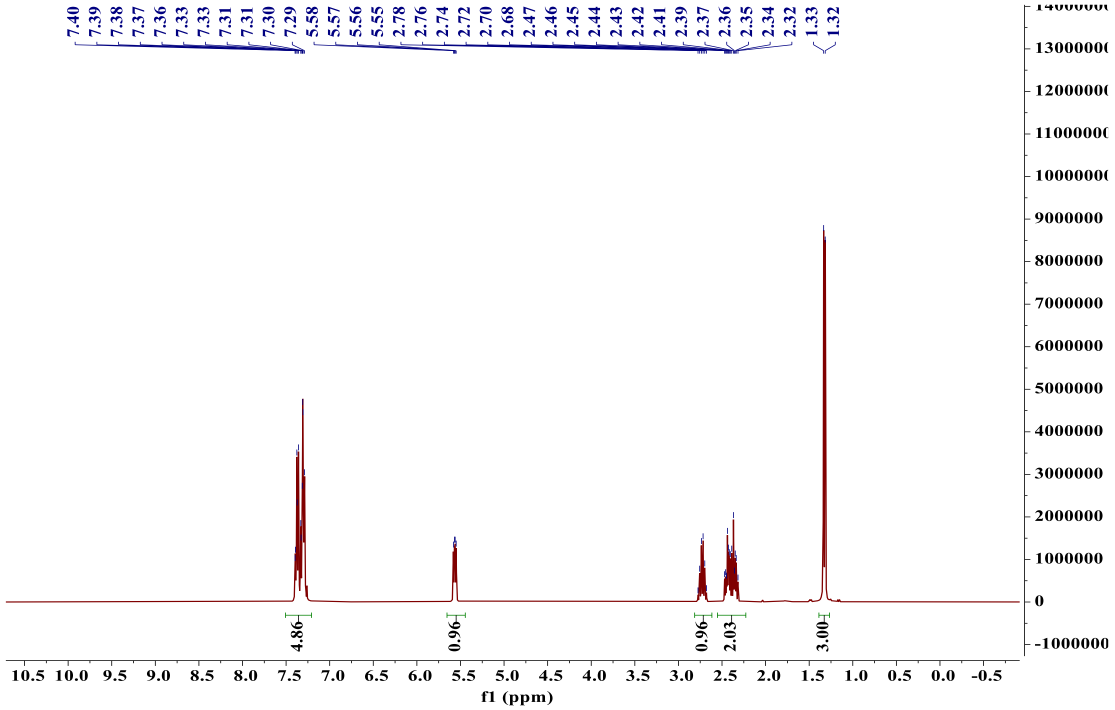

^{1}H NMR (400 MHz, $CDCl_3$) spectrum of **(*S*,*R*)-3a**

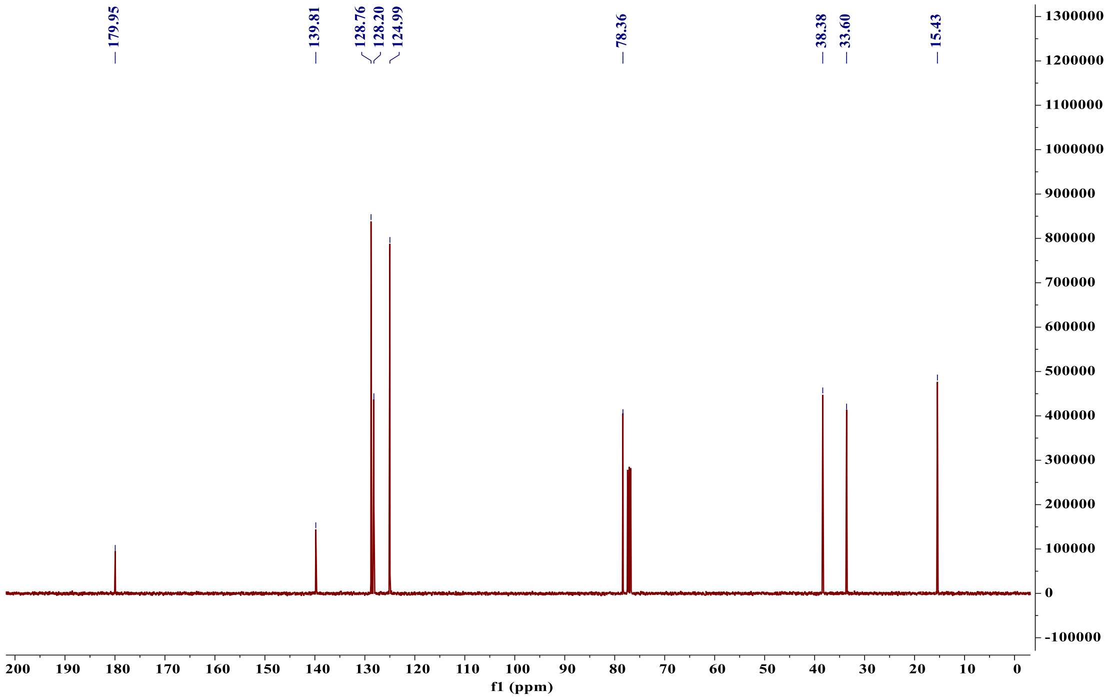

^{13}C NMR (101 MHz, $CDCl_3$) spectrum of **(*S*,*R*)-3a**

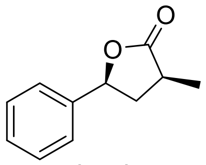

(*S*,*S*)-3a

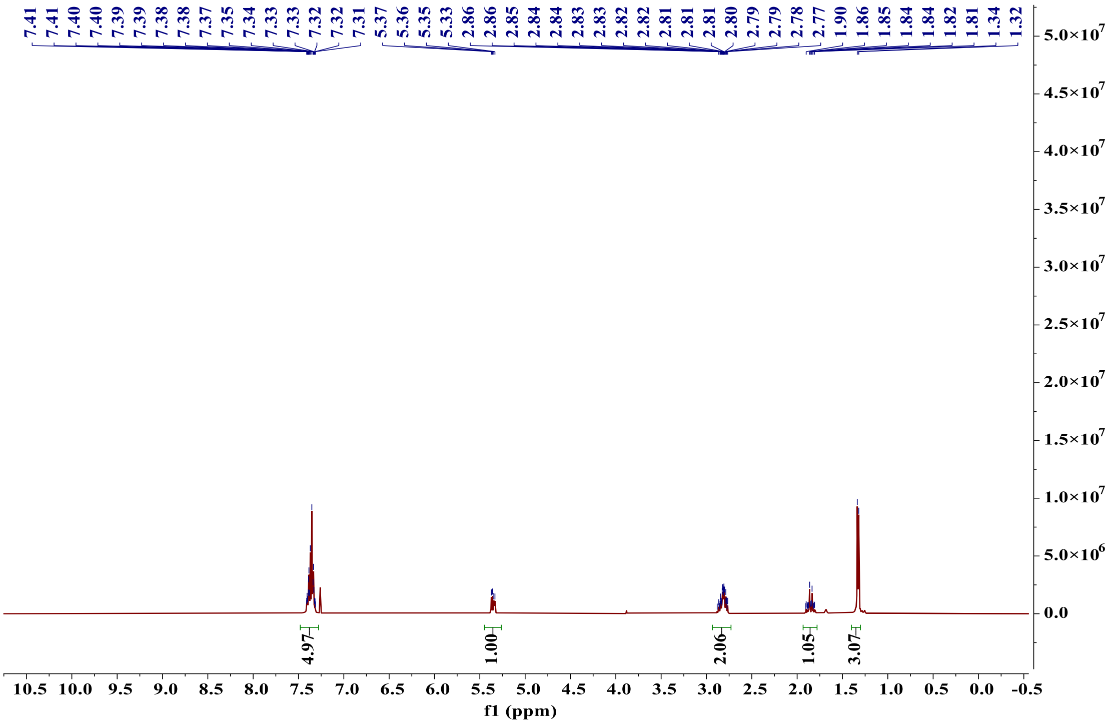

^{1}H NMR (400 MHz, $CDCl_3$) spectrum of **(*S*,*S*)-3a**

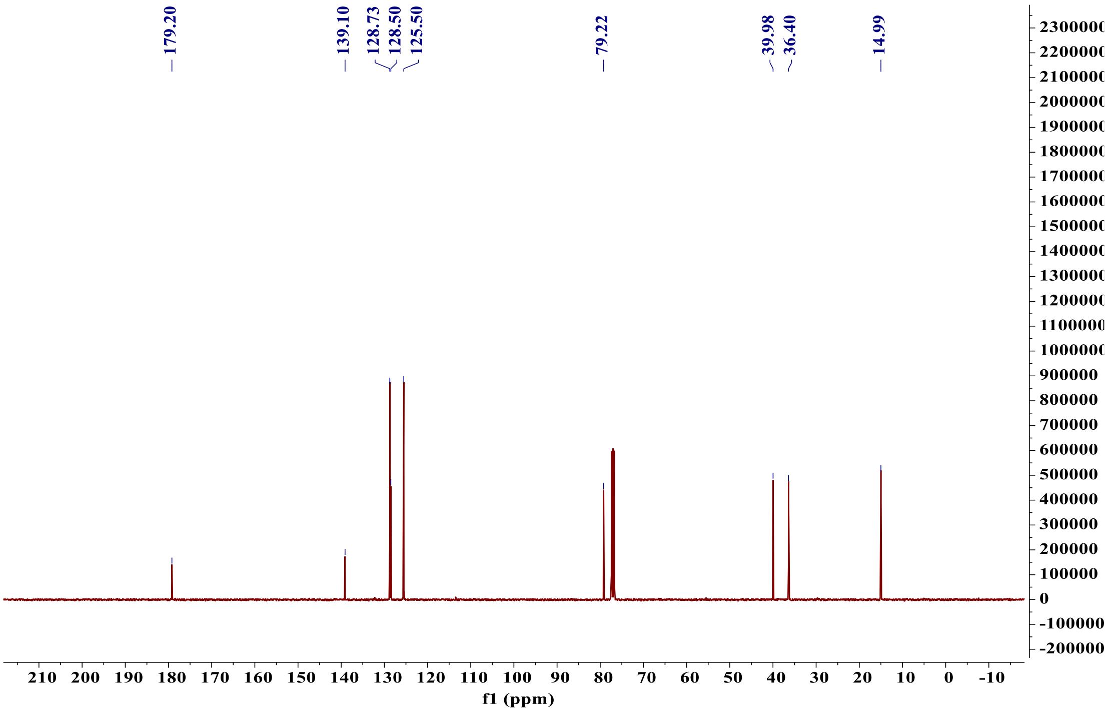

^{13}C NMR (101 MHz, $CDCl_3$) spectrum of **(*S*,*S*)-3a**

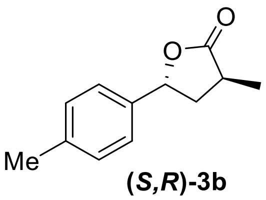

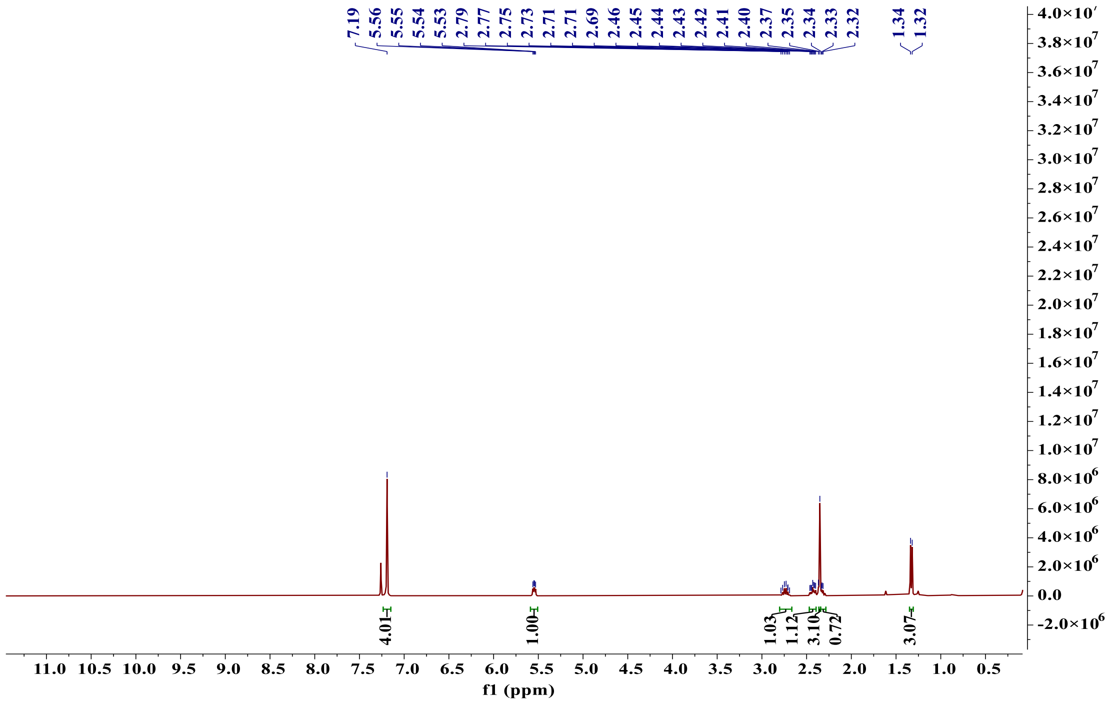

^{1}H NMR (400 MHz, $CDCl_3$) spectrum of ***(S,R)*-3b**

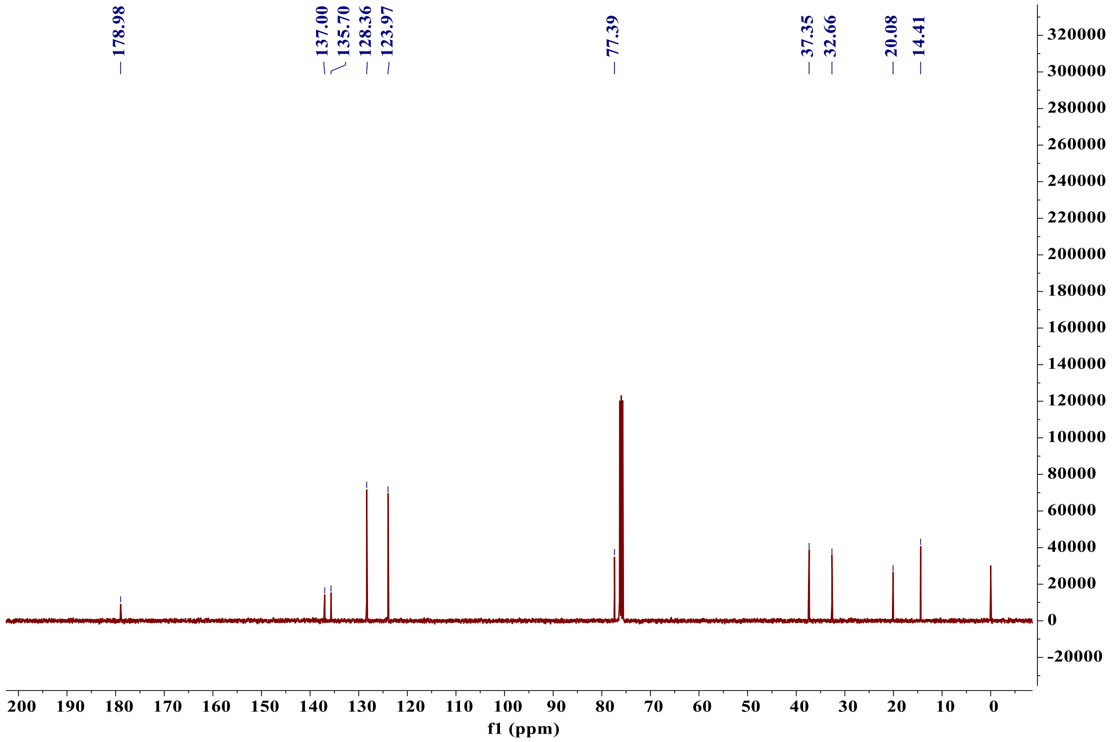

^{13}C NMR (101 MHz, $CDCl_3$) spectrum of ***(S,R)*-3b**

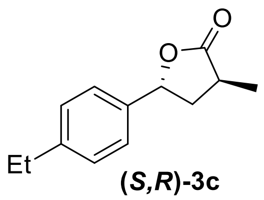

(*S*,*R*)-3c

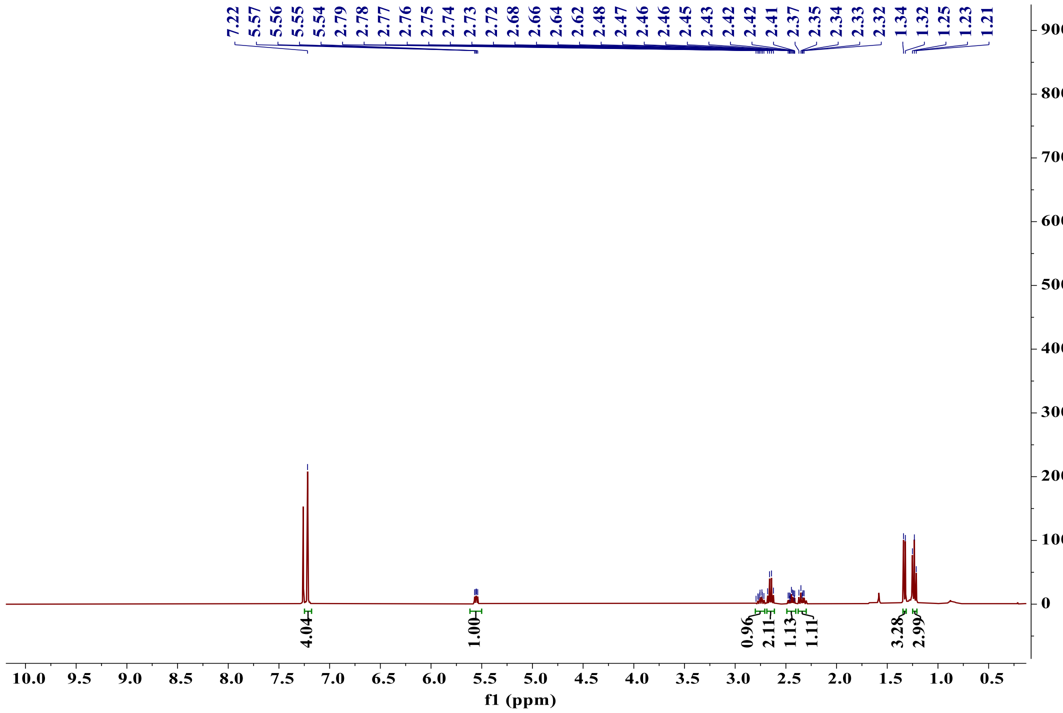

^{1}H NMR (400 MHz, $CDCl_3$) spectrum of **(*S*,*R*)-3c**

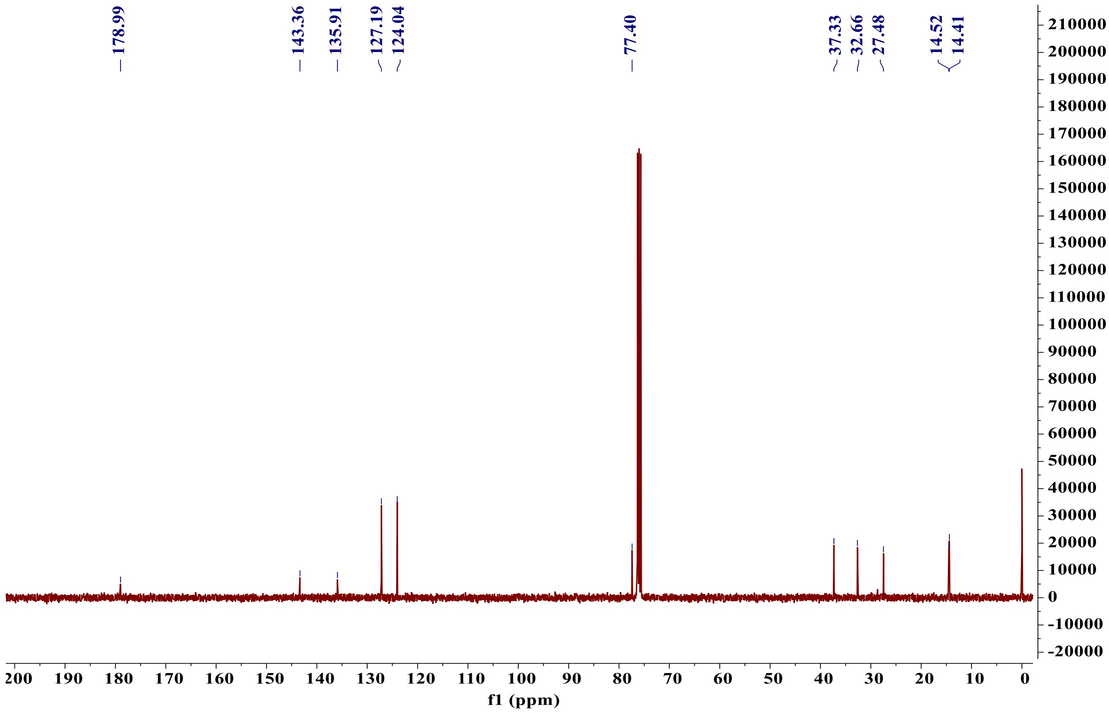

^{13}C NMR (101 MHz, $CDCl_3$) spectrum of **(*S*,*R*)-3c**

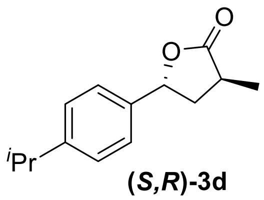

(*S*,*R*)-3d

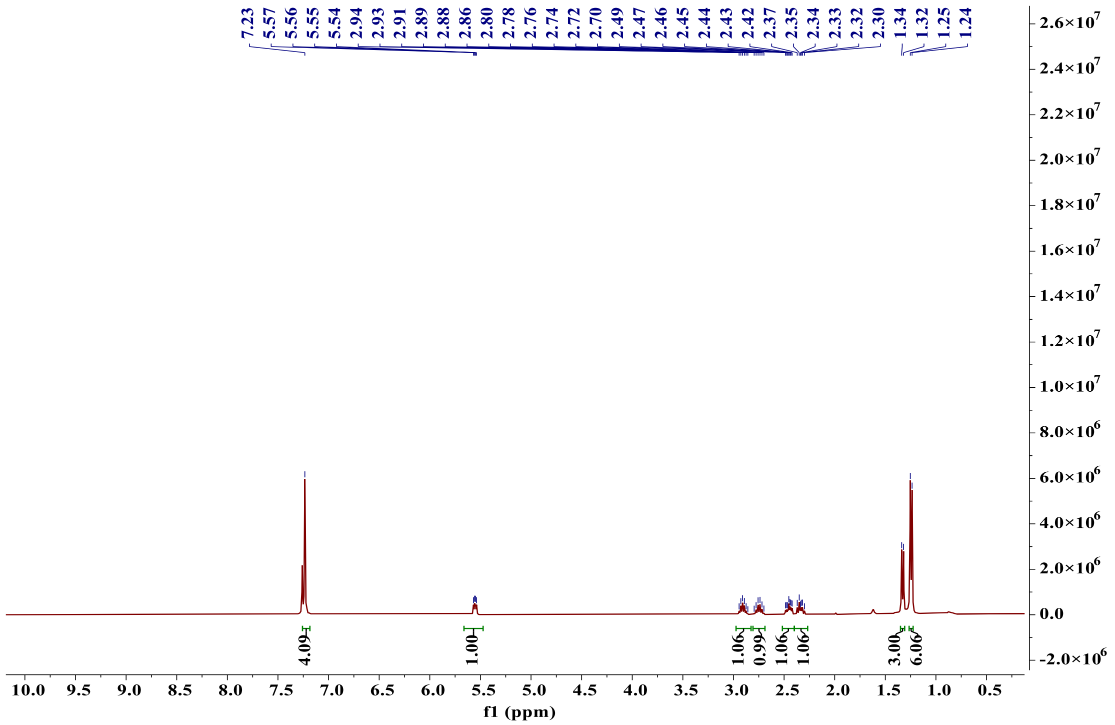

^{1}H NMR (400 MHz, $CDCl_3$) spectrum of **(*S*,*R*)-3d**

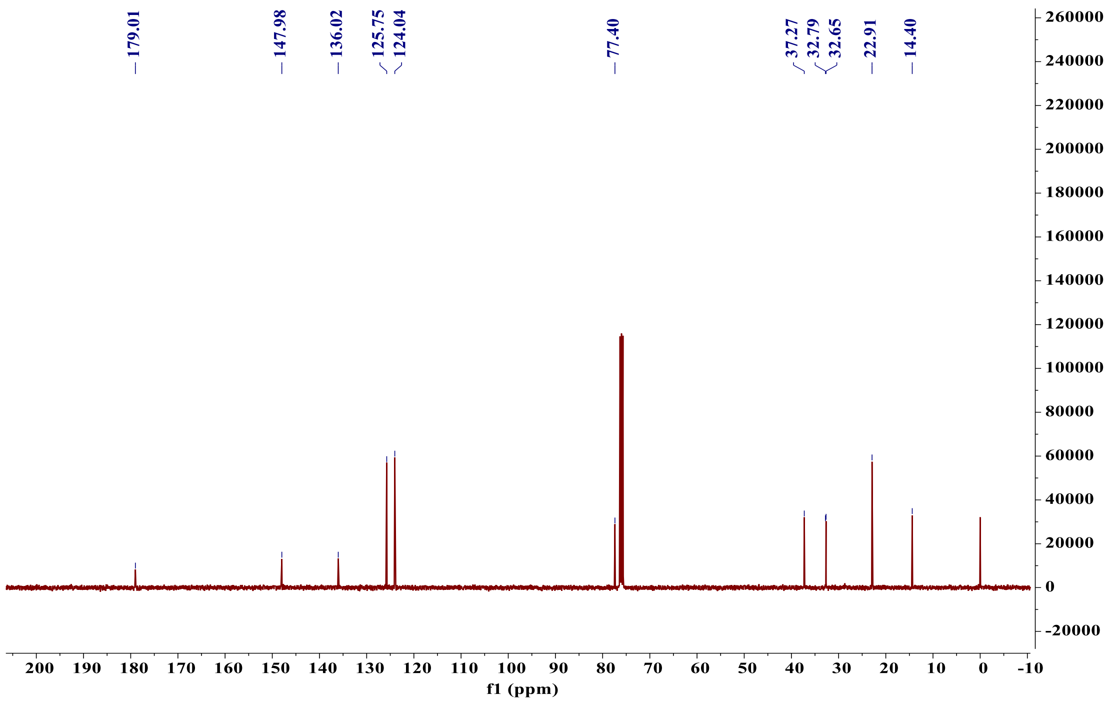

^{13}C NMR (101 MHz, $CDCl_3$) spectrum of **(*S*,*R*)-3d**

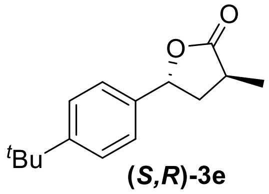

(*S*,*R*)-3e

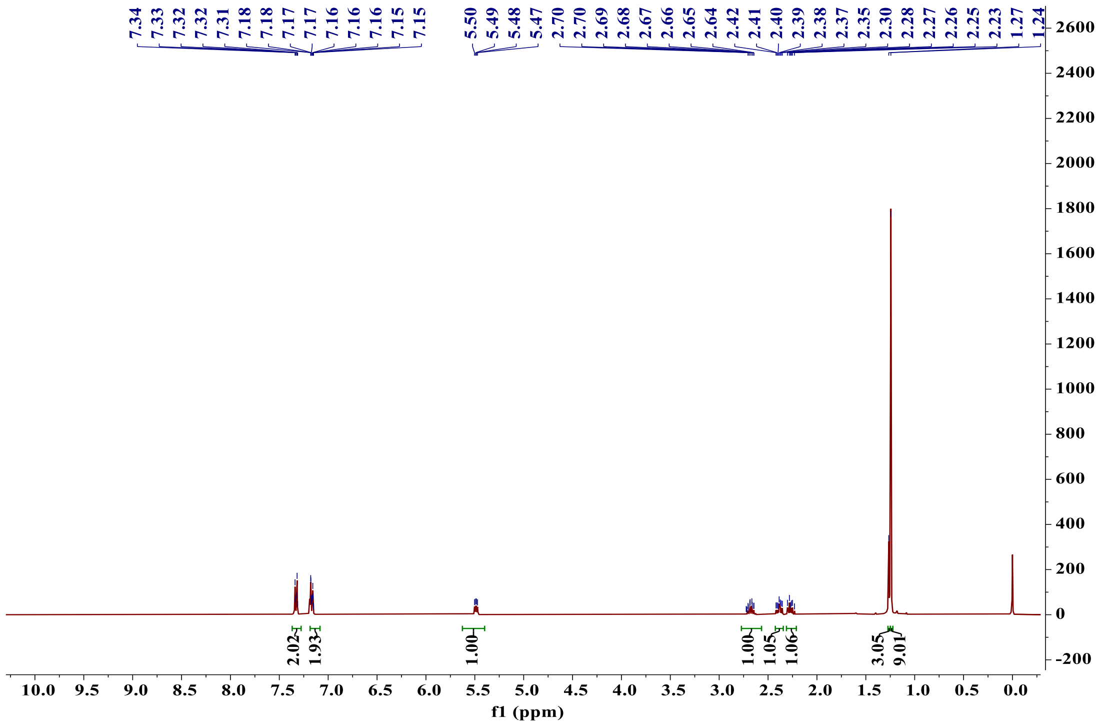

^1H NMR (400 MHz, $CDCl_3$) spectrum of **(*S*,*R*)-3e**

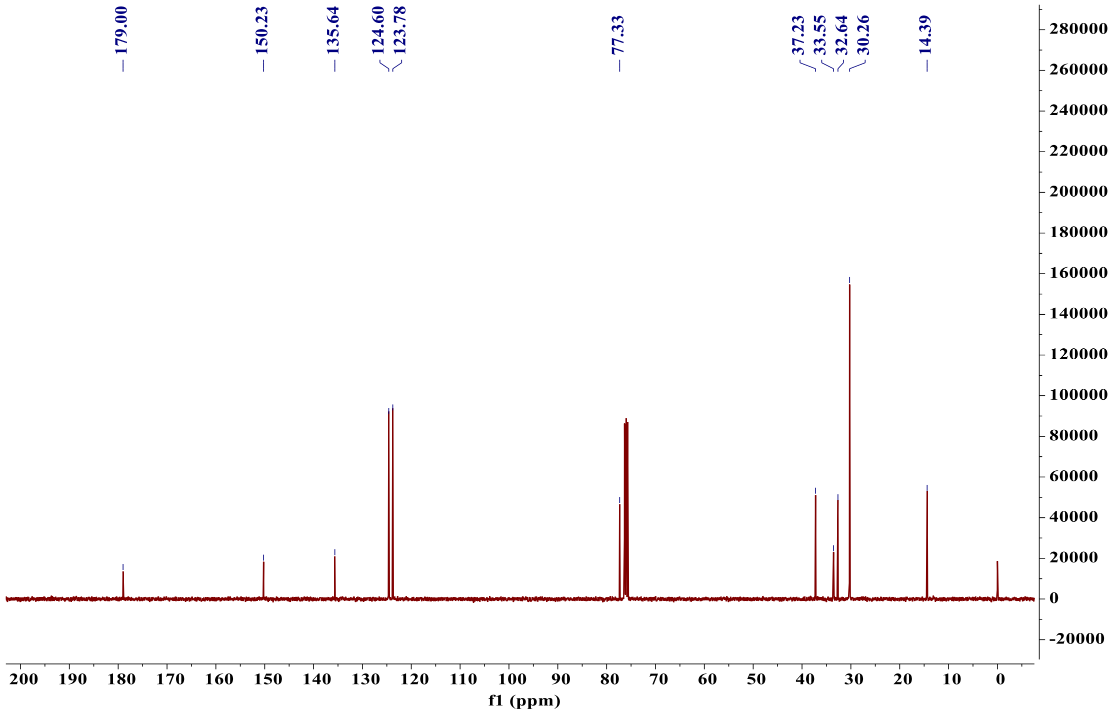

^{13}C NMR (101 MHz, $CDCl_3$) spectrum of **(*S*,*R*)-3e**

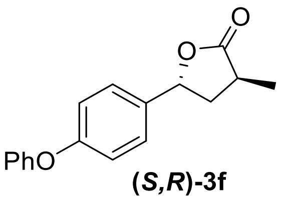

(*S*,*R*)-3f

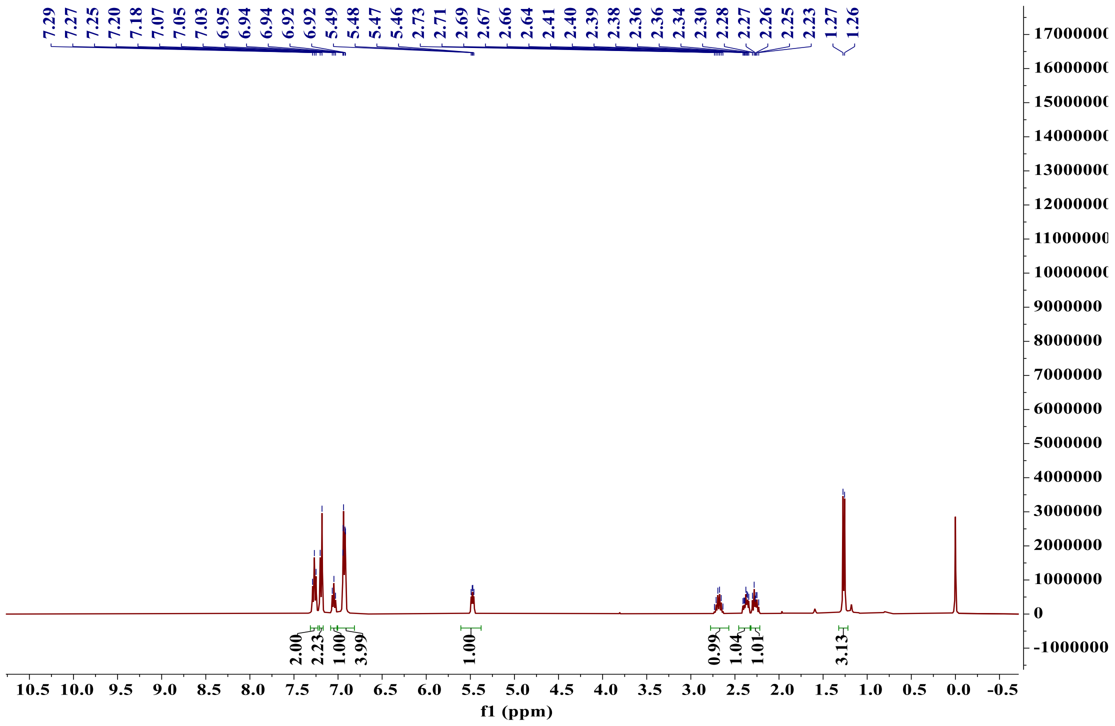

^{1}H NMR (400 MHz, $CDCl_3$) spectrum of **(*S*,*R*)-3f**

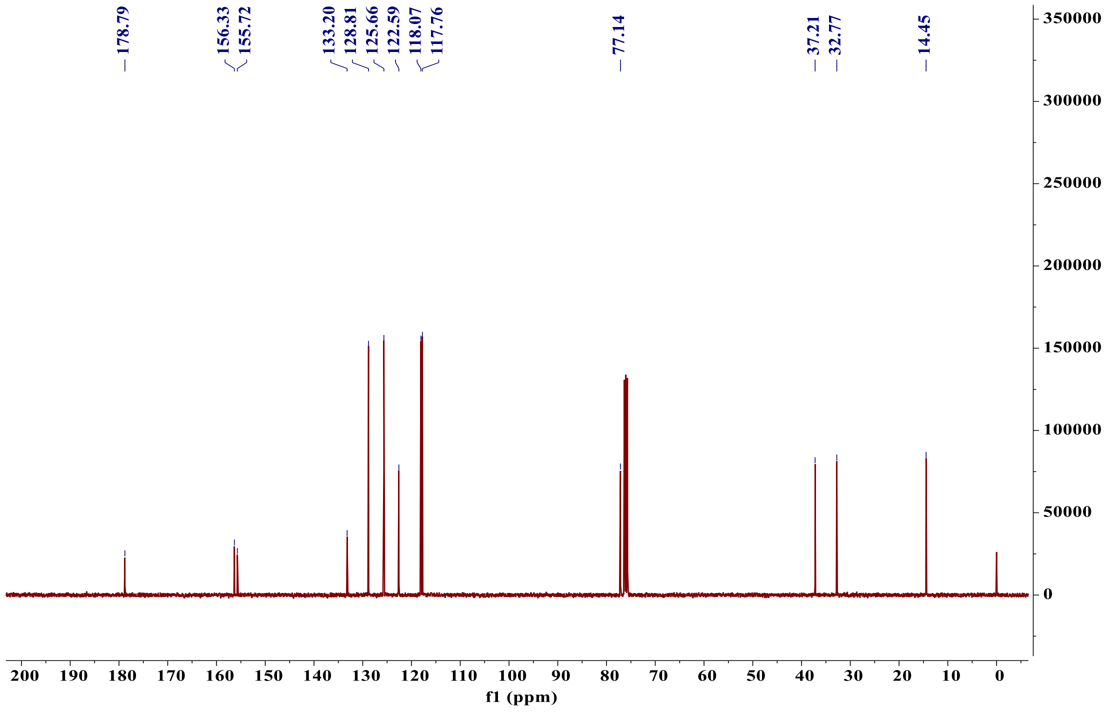

^{13}C NMR (101 MHz, $CDCl_3$) spectrum of **(*S*,*R*)-3f**

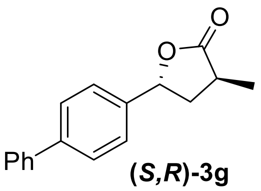

(*S*,*R*)-3g

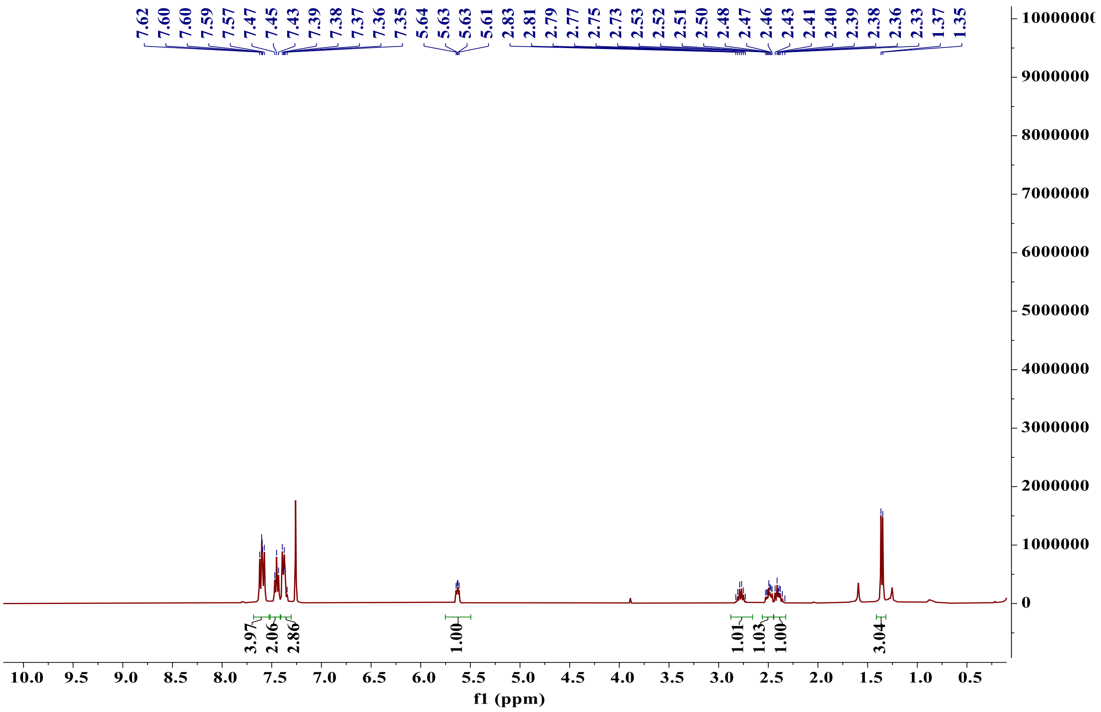

^{1}H NMR (400 MHz, CDCl3) spectrum of **(*S*,*R*)-3g**

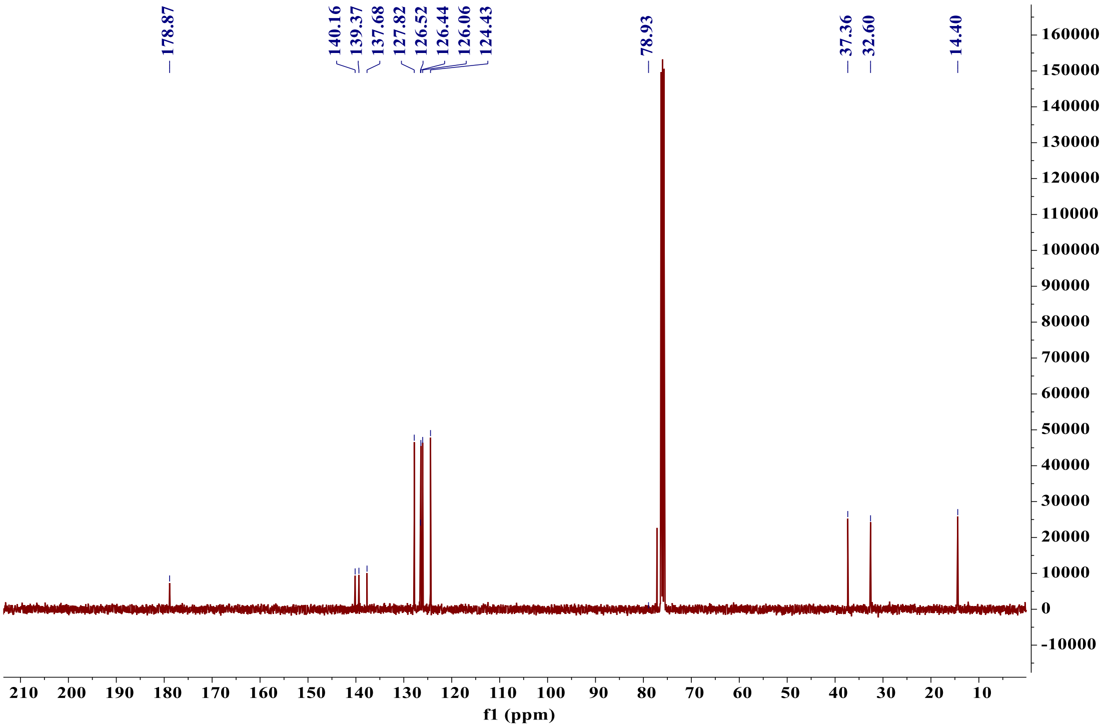

^{13}C NMR (101 MHz, CDCl3) spectrum of **(*S*,*R*)-3g**

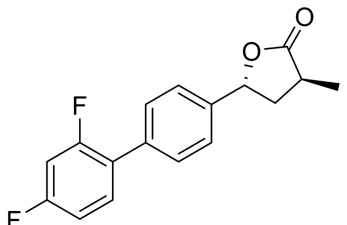

(*S*,*R*)-3h

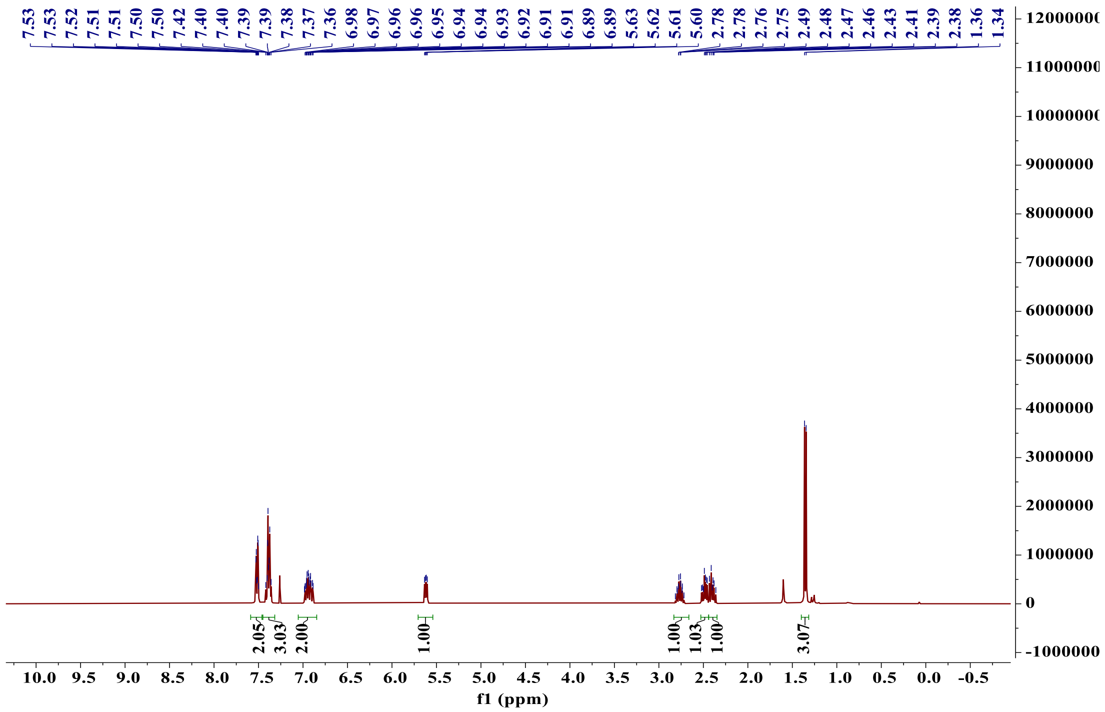

^{1}H NMR (400 MHz, $CDCl_3$) spectrum of **(*S*,*R*)-3h**

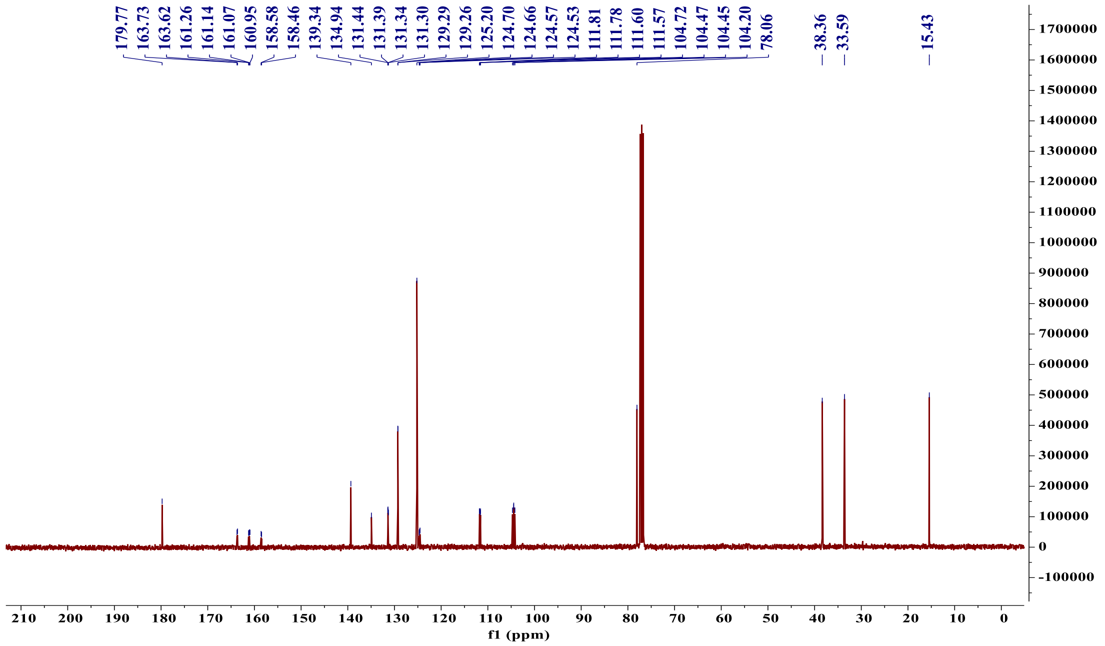

^{13}C NMR (101 MHz, $CDCl_3$) spectrum of **(*S*,*R*)-3h**

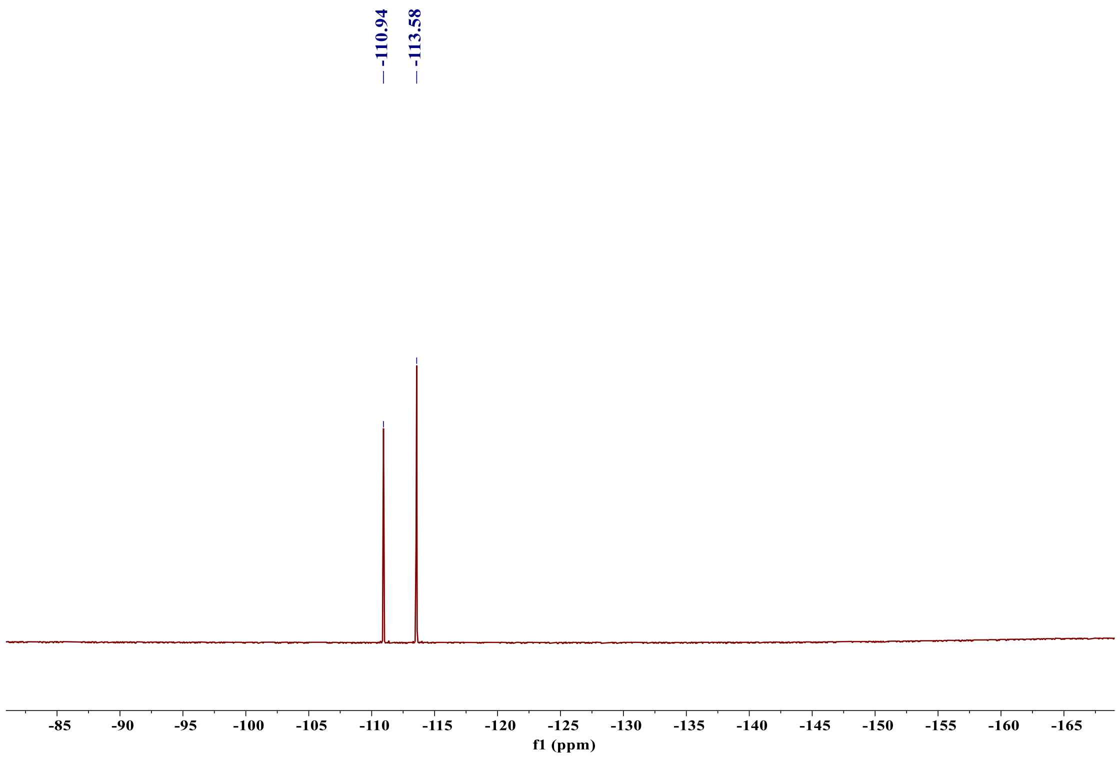

^{19}F NMR (377 MHz, $CDCl_3$) spectrum of **(*S*,*R*)-3h**

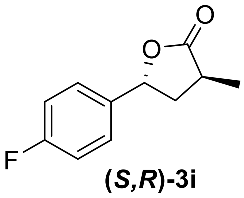

(*S*,*R*)-3i

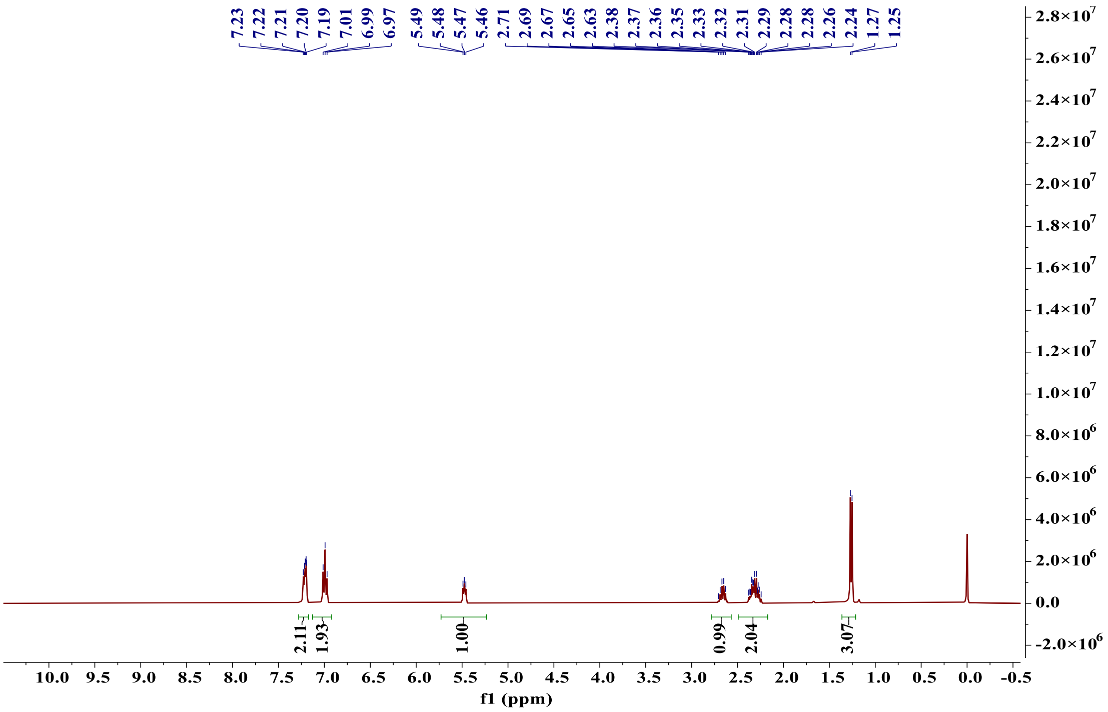

^{1}H NMR (400 MHz, $CDCl_3$) spectrum of **(*S*,*R*)-3i**

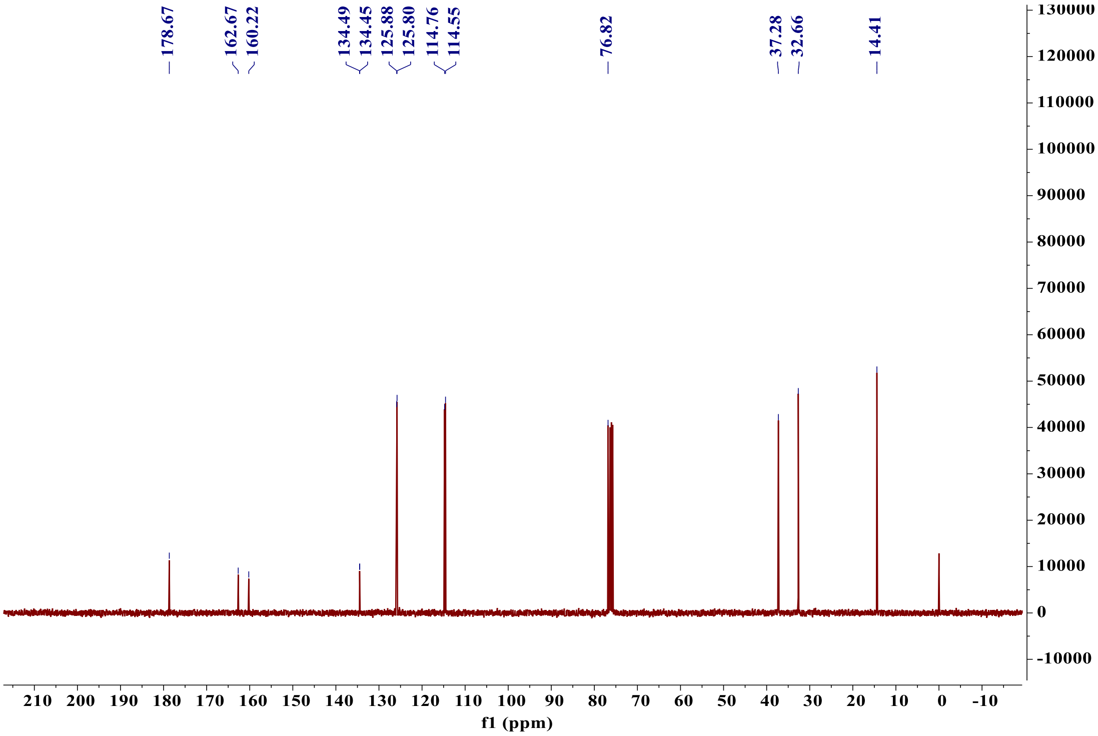

^{13}C NMR (101 MHz, $CDCl_3$) spectrum of **(*S*,*R*)-3i**

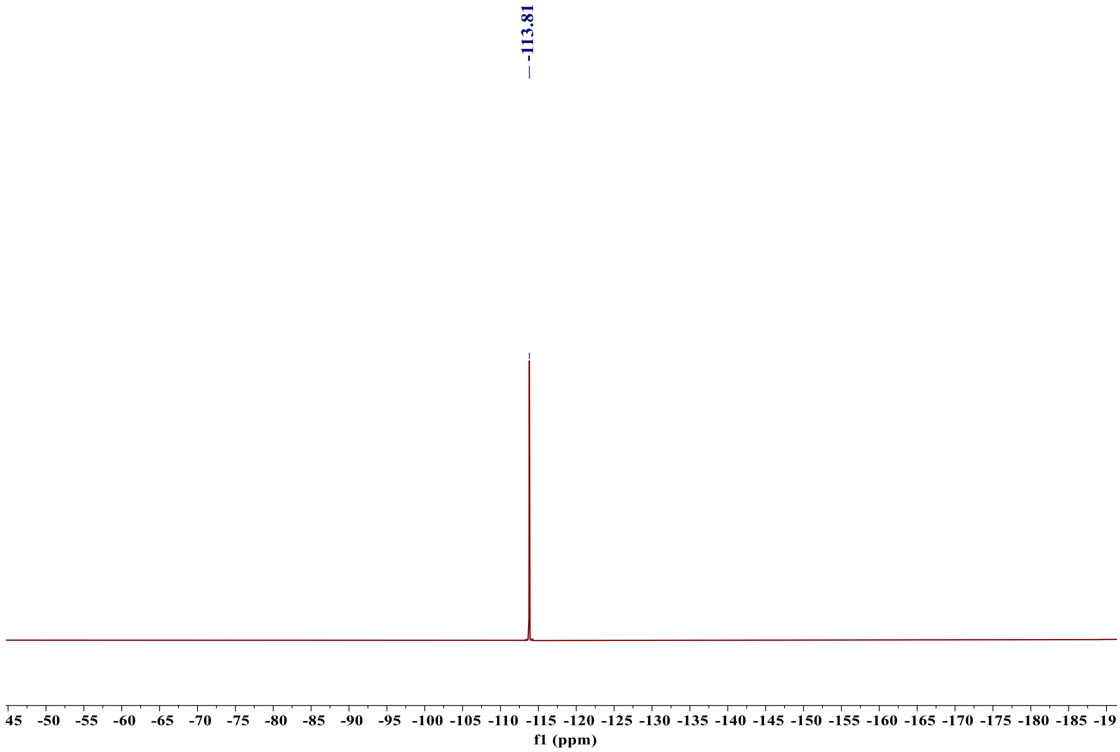

^{19}F NMR (377 MHz, $CDCl_3$) spectrum of **(*S*,*R*)-3i**

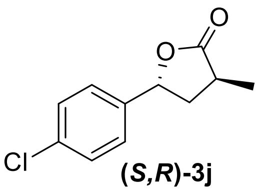

(*S*,*R*)-3j

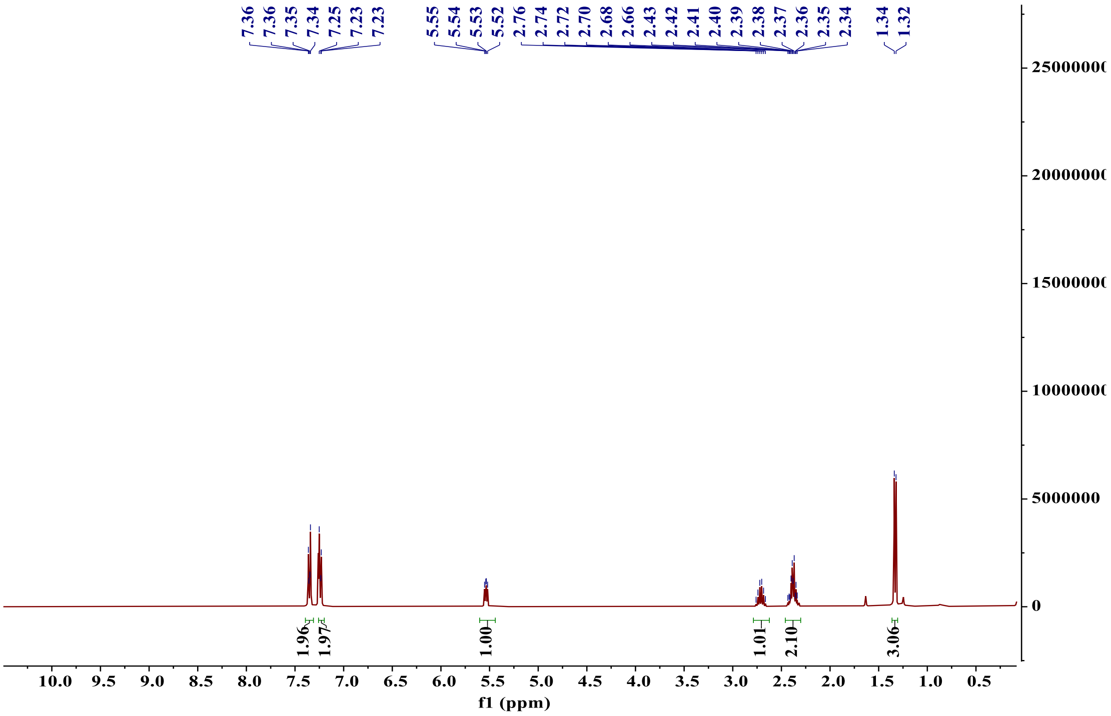

^{1}H NMR (400 MHz, $CDCl_3$) spectrum of **(*S*,*R*)-3j**

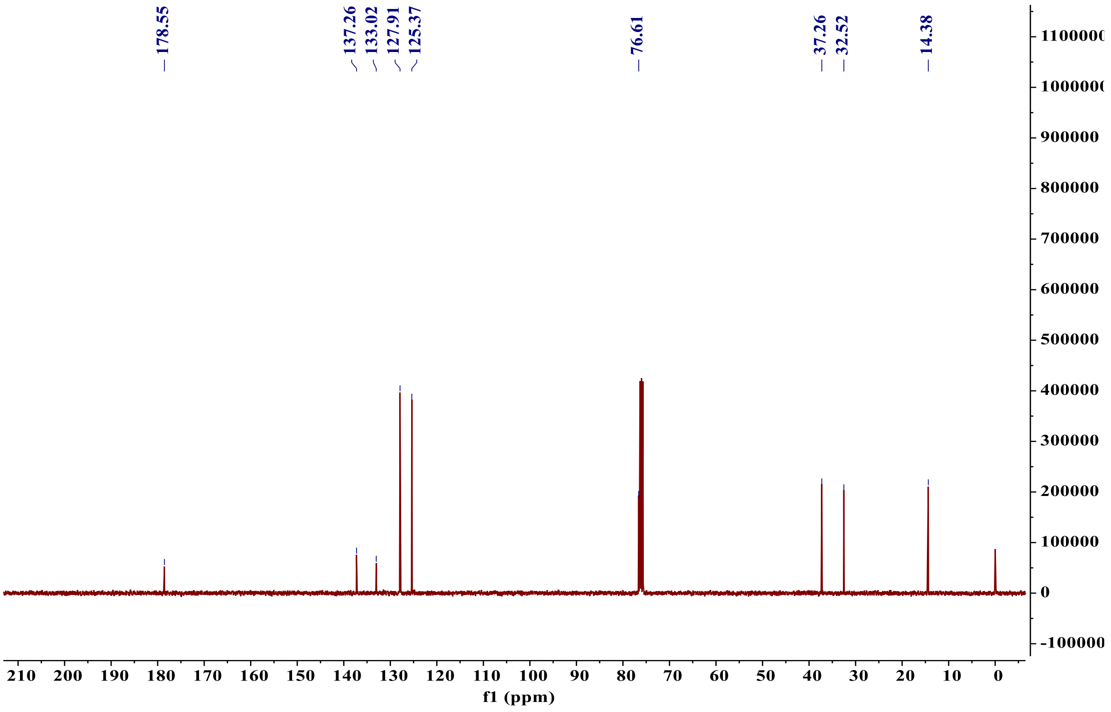

^{13}C NMR (101 MHz, $CDCl_3$) spectrum of **(*S*,*R*)-3j**

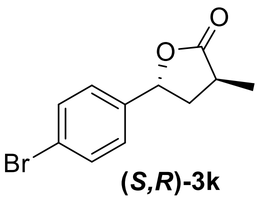

(*S*,*R*)-3k

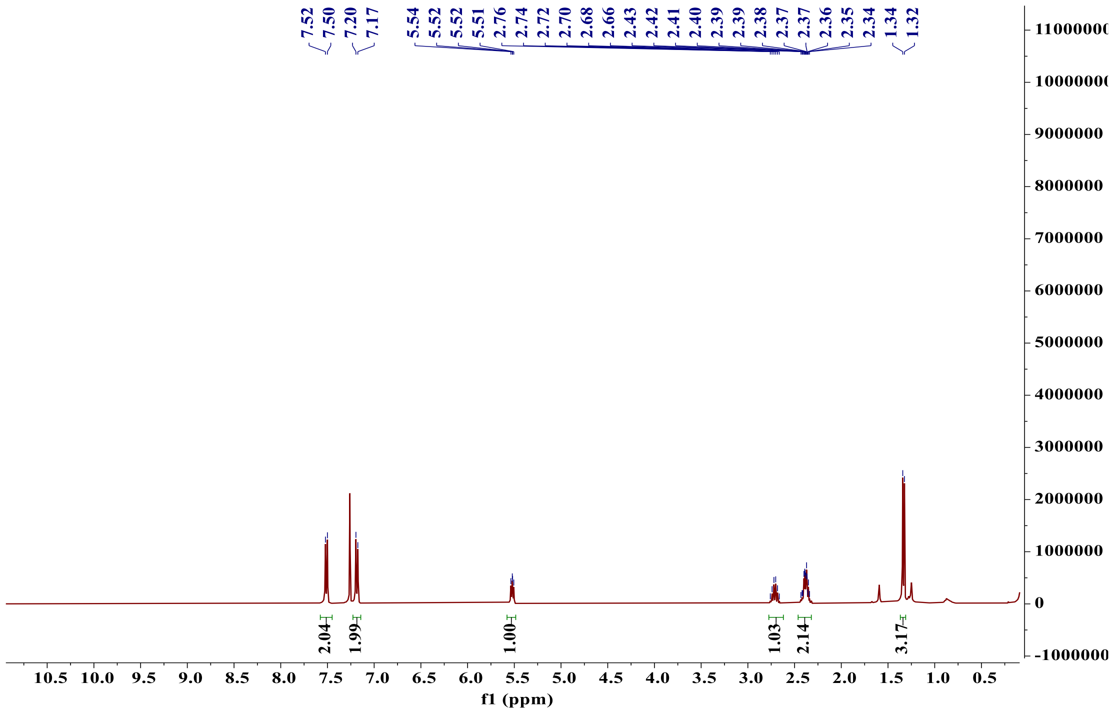

^{1}H NMR (400 MHz, $CDCl_3$) spectrum of **(*S*,*R*)-3k**

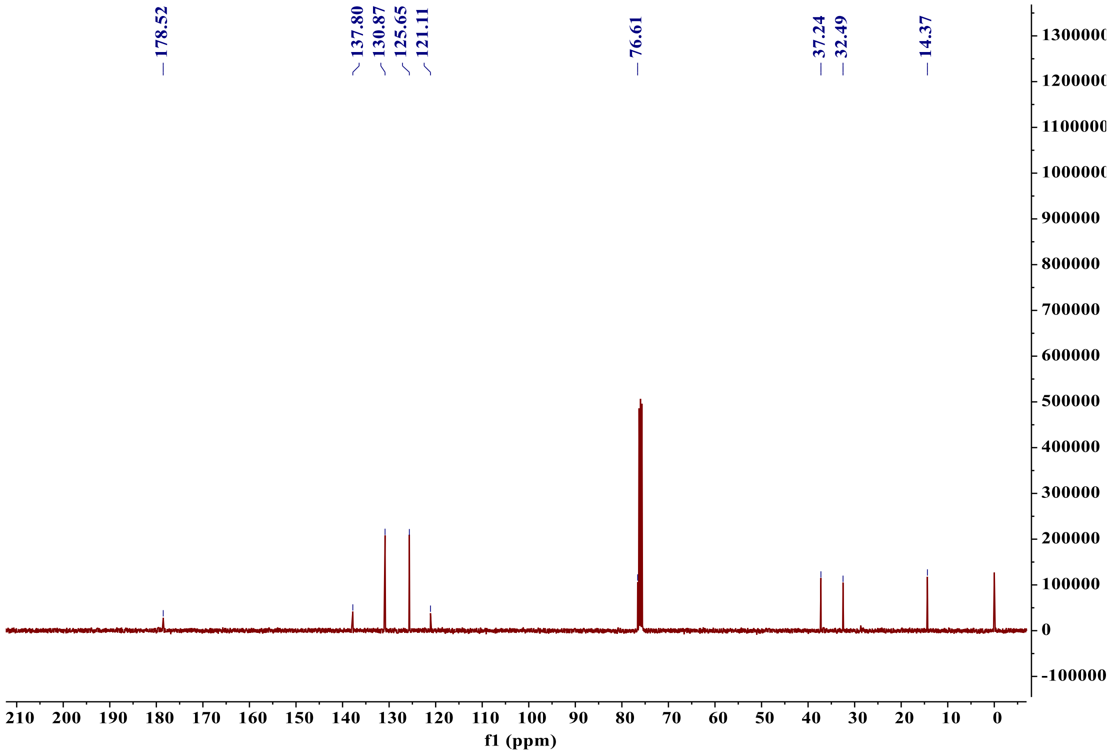

^{13}C NMR (101 MHz, $CDCl_3$) spectrum of **(*S*,*R*)-3k**

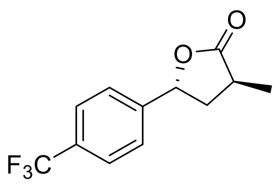

(*S*,*R*)-3l

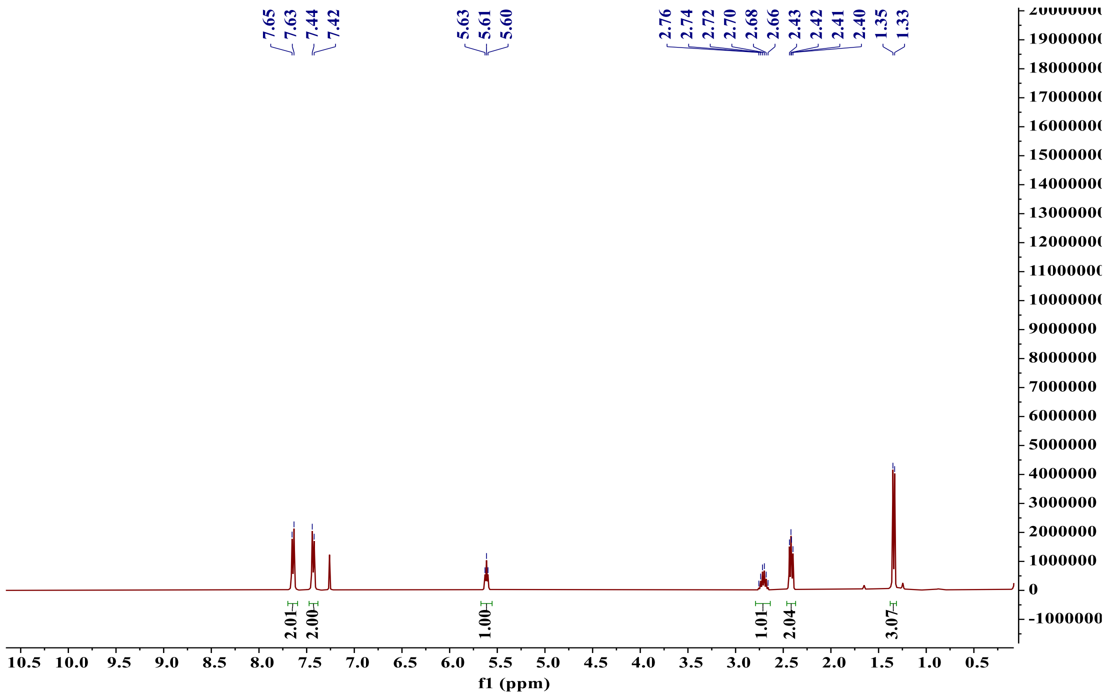

^{1}H NMR (400 MHz, $CDCl_3$) spectrum of **(*S*,*R*)-3l**

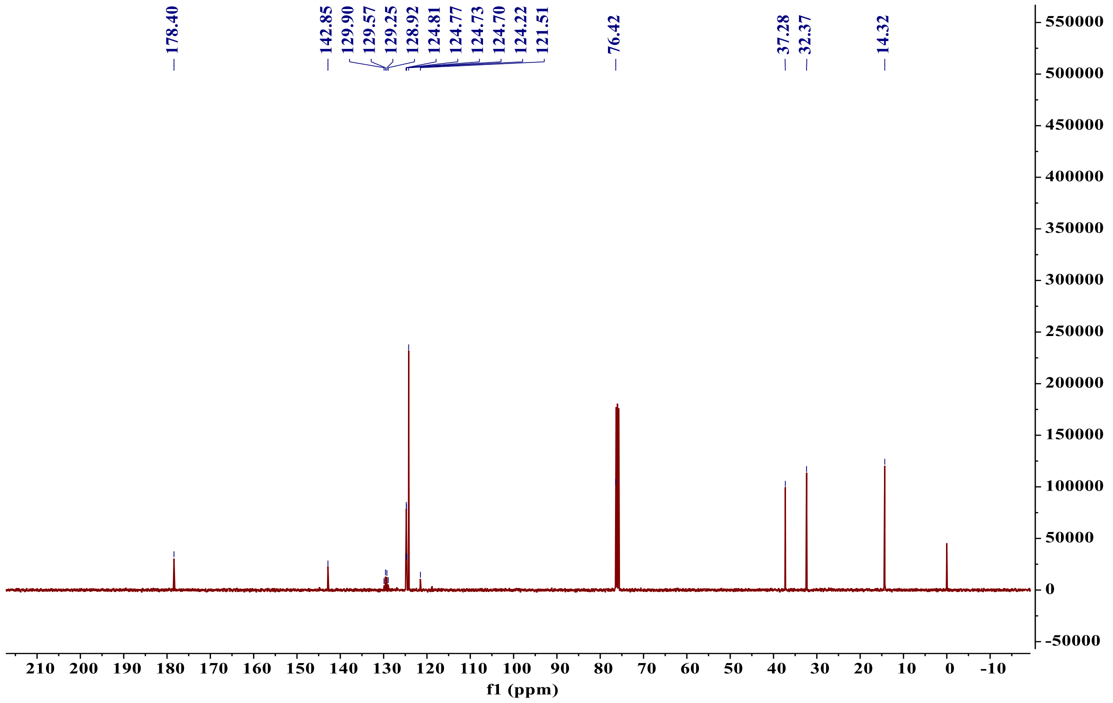

^{13}C NMR (101 MHz, $CDCl_3$) spectrum of **(*S*,*R*)-3l**

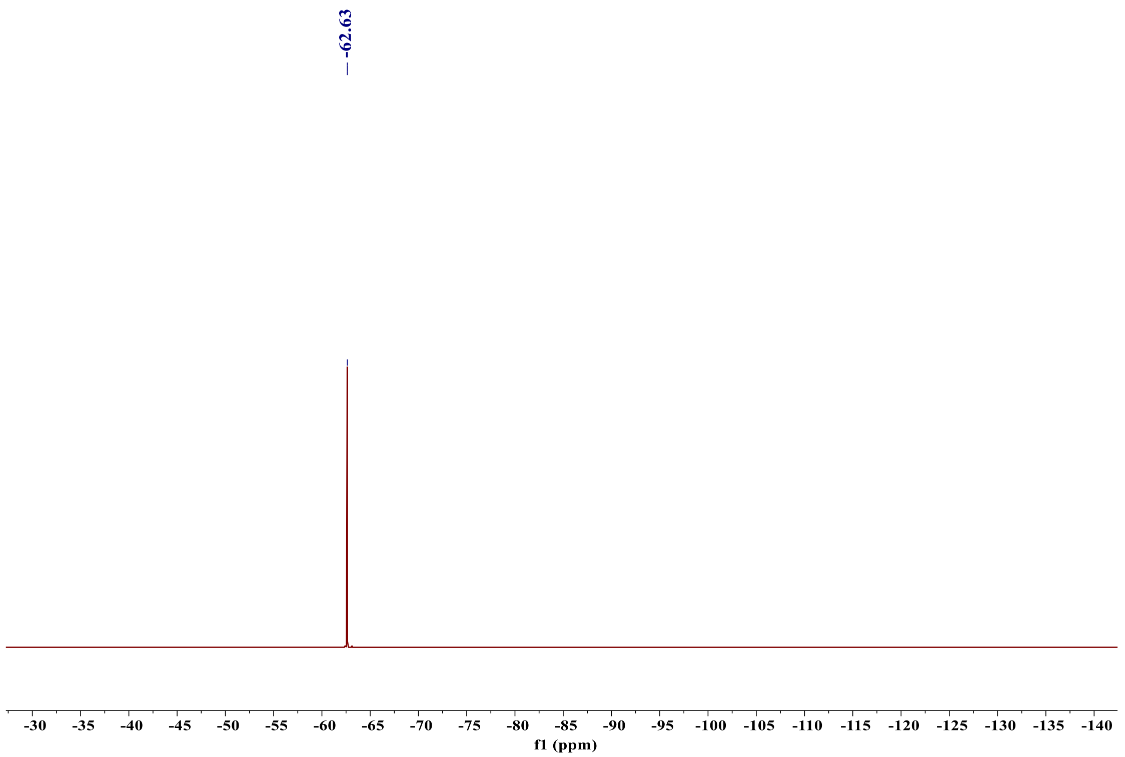

^{19}F NMR (377 MHz, $CDCl_3$) spectrum of **(*S*,*R*)-3l**

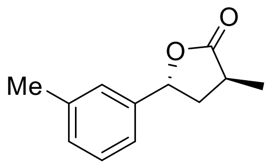

(*S*,*R*)-3m

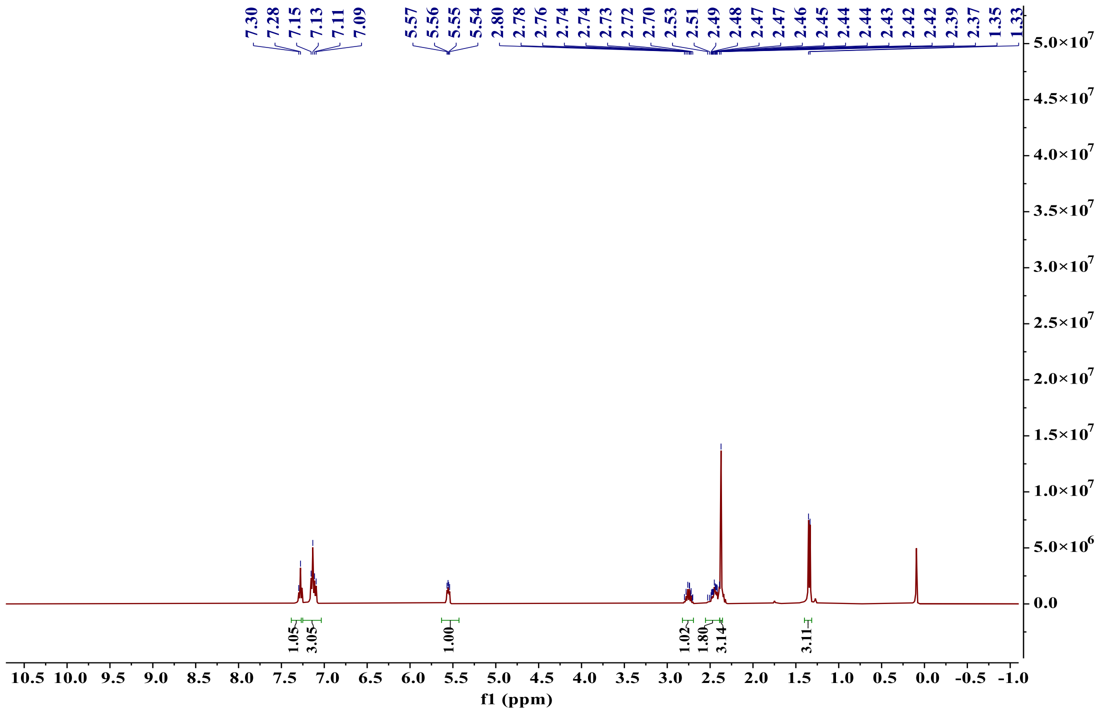

^{1}H NMR (400 MHz, $CDCl_3$) spectrum of **(*S*,*R*)-3m**

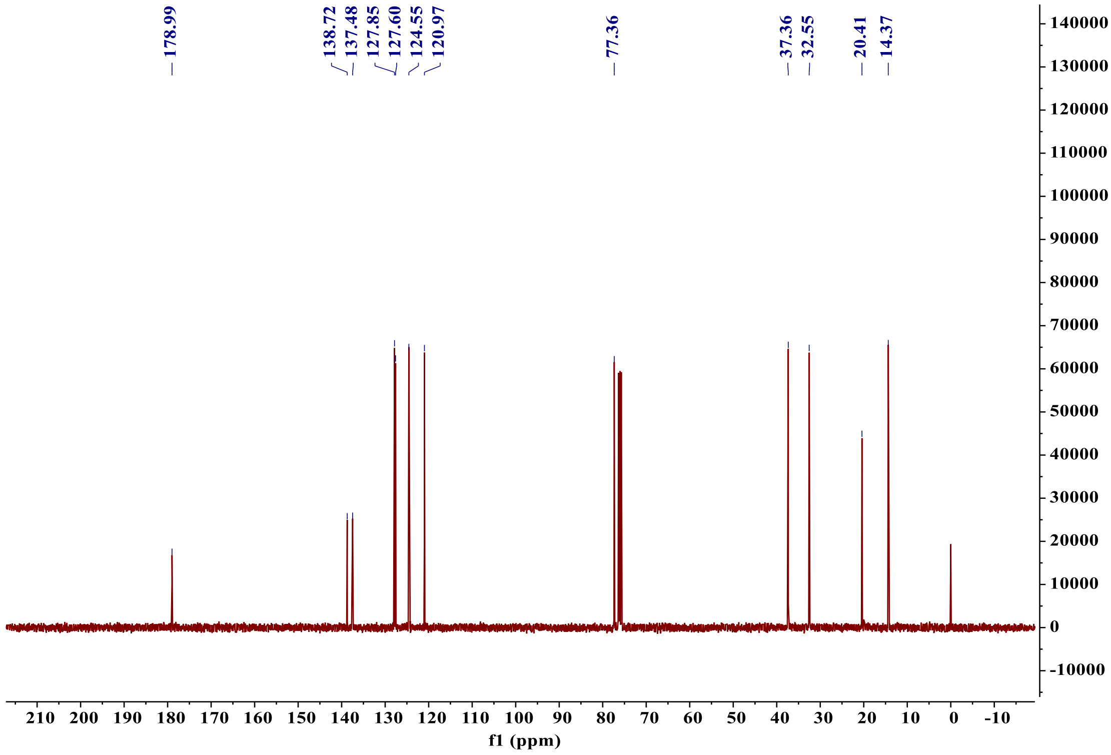

^{13}C NMR (101 MHz, $CDCl_3$) spectrum of **(*S*,*R*)-3m**

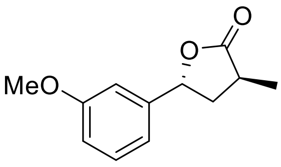

(*S*,*R*)-3n

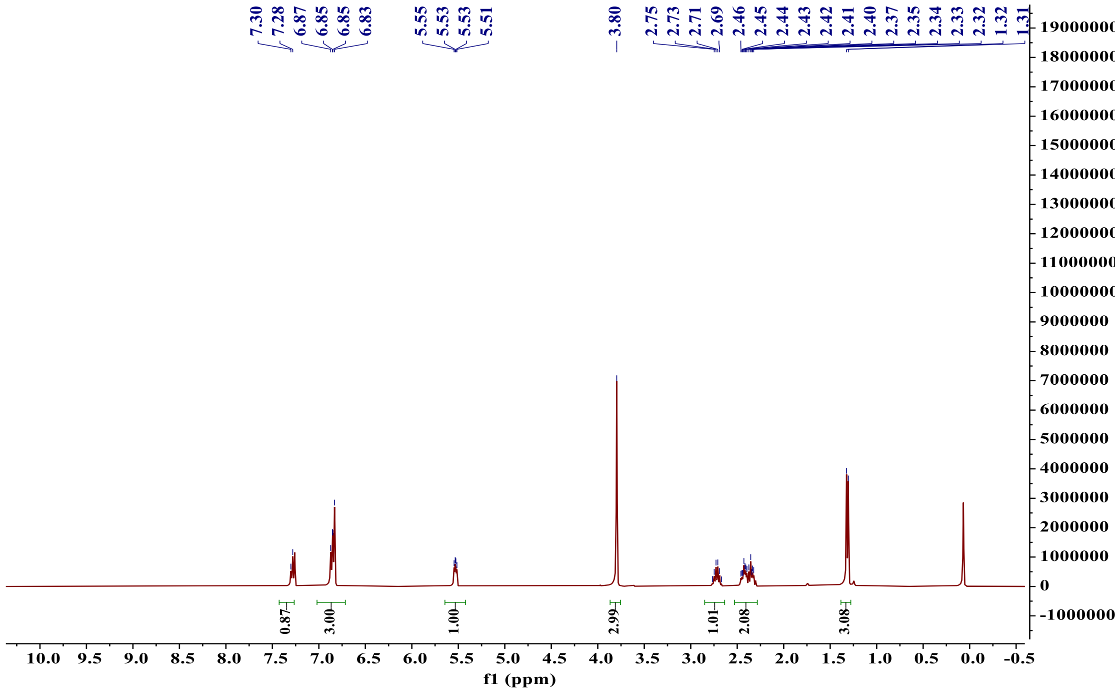

^{1}H NMR (400 MHz, $CDCl_3$) spectrum of **(*S*,*R*)-3n**

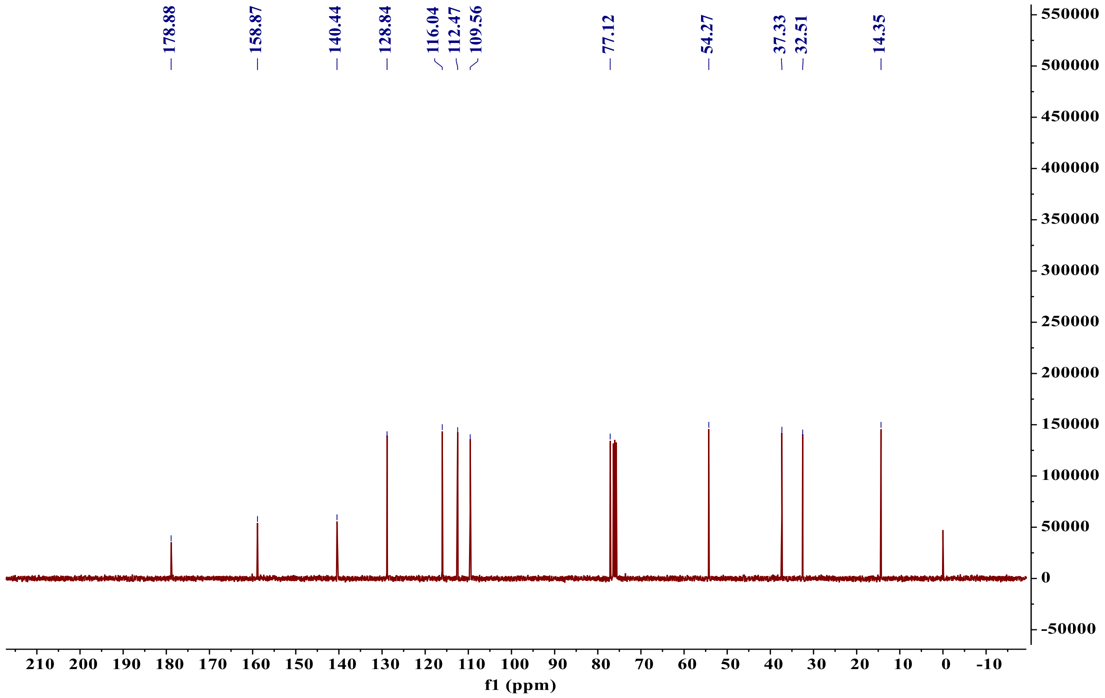

^{13}C NMR (101 MHz, $CDCl_3$) spectrum of **(*S*,*R*)-3n**

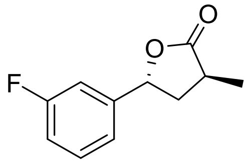

(*S*,*R*)-3o

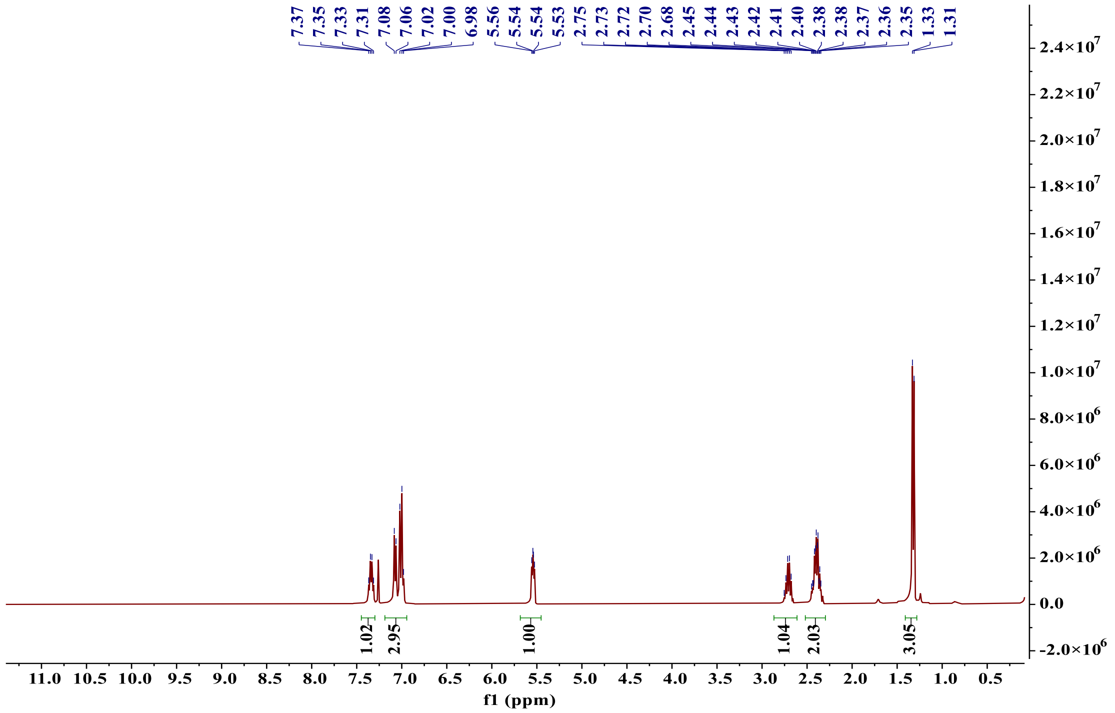

^{1}H NMR (400 MHz, $CDCl_3$) spectrum of **(*S*,*R*)-3o**

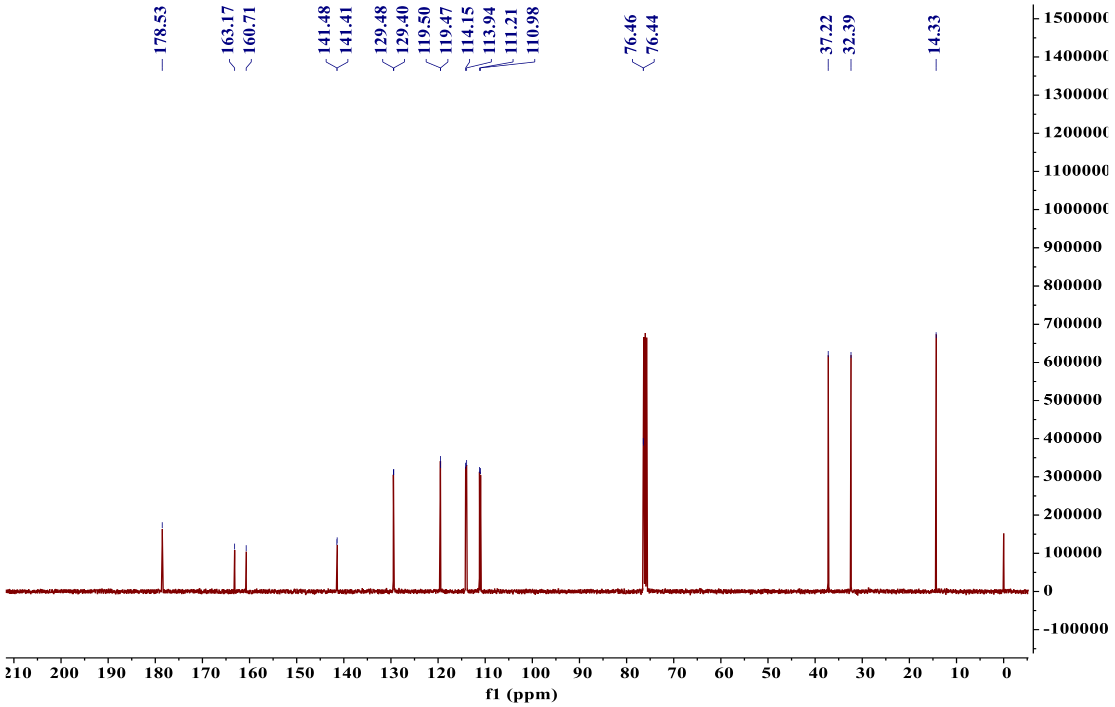

^{13}C NMR (101 MHz, $CDCl_3$) spectrum of **(*S*,*R*)-3o**

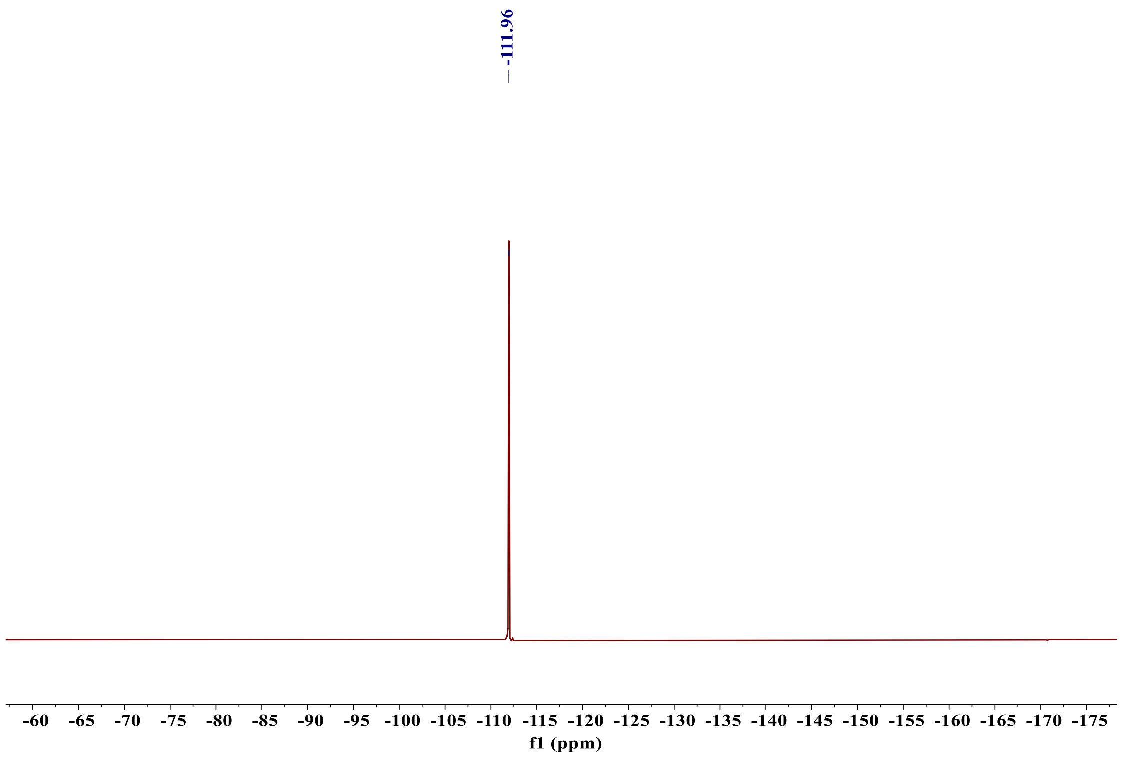

^{19}F NMR (377 MHz, $CDCl_3$) spectrum of **(*S*,*R*)-3o**

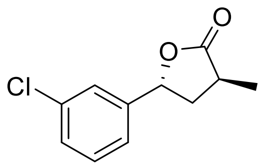

(*S*,*R*)-3p

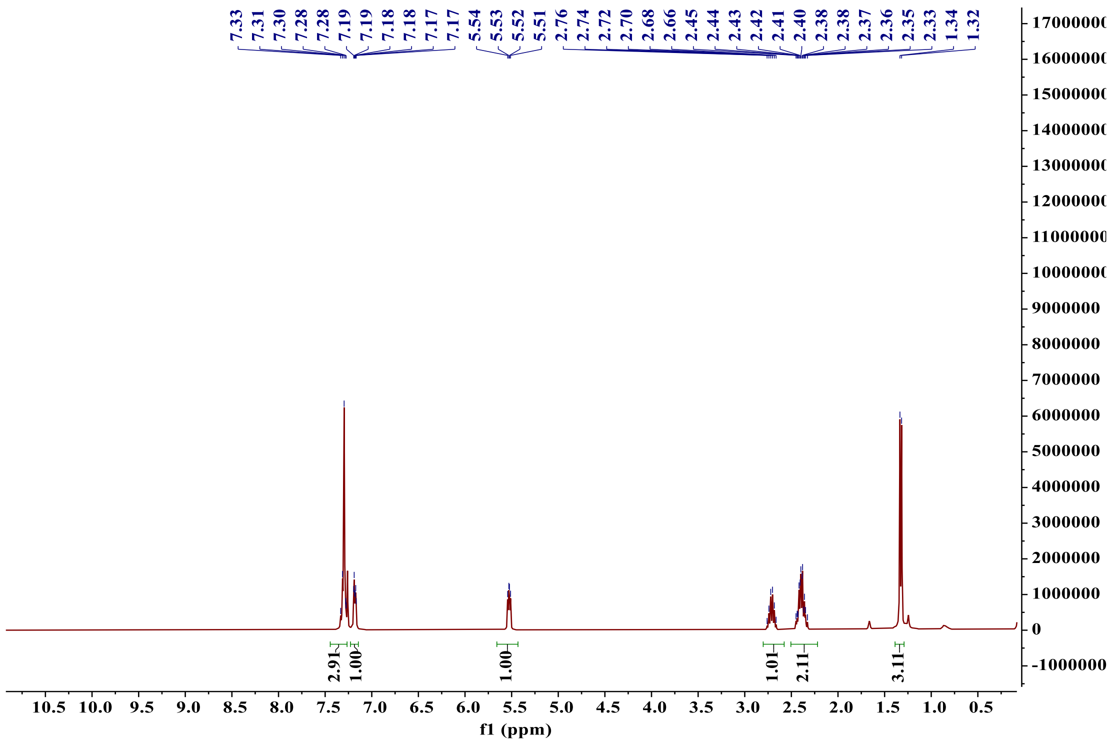

^{1}H NMR (400 MHz, $CDCl_3$) spectrum of **(*S*,*R*)-3p**

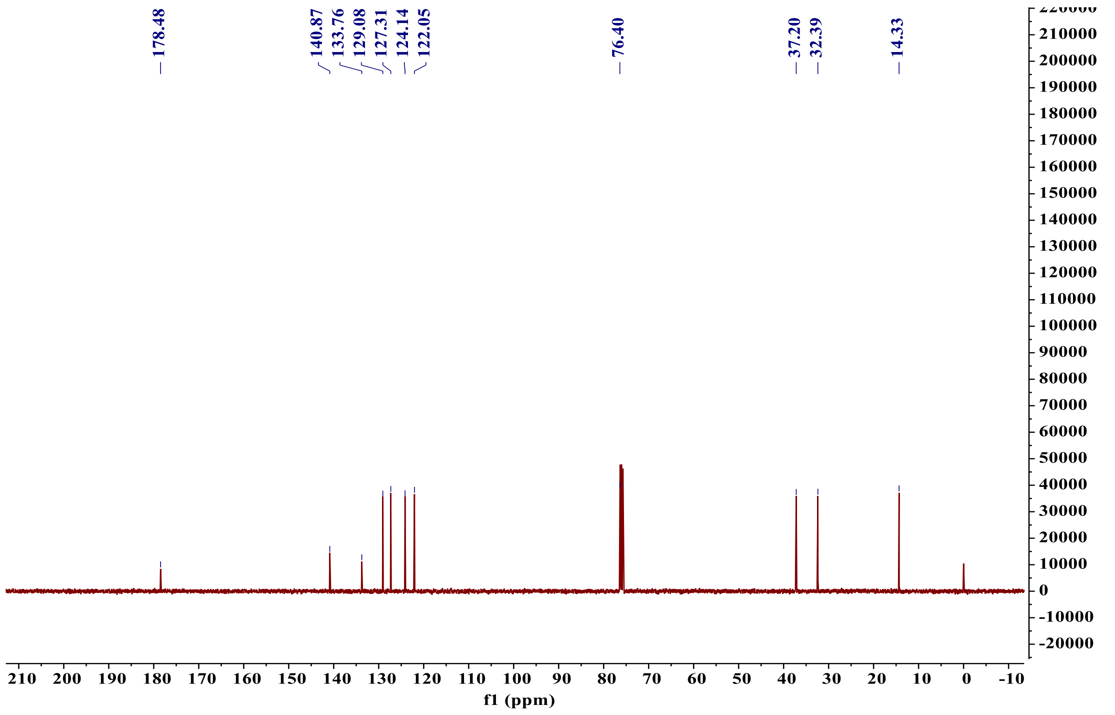

^{13}C NMR (101 MHz, $CDCl_3$) spectrum of **(*S*,*R*)-3p**

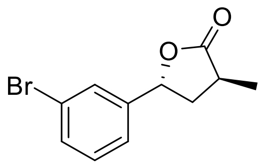

(*S*,*R*)-3q

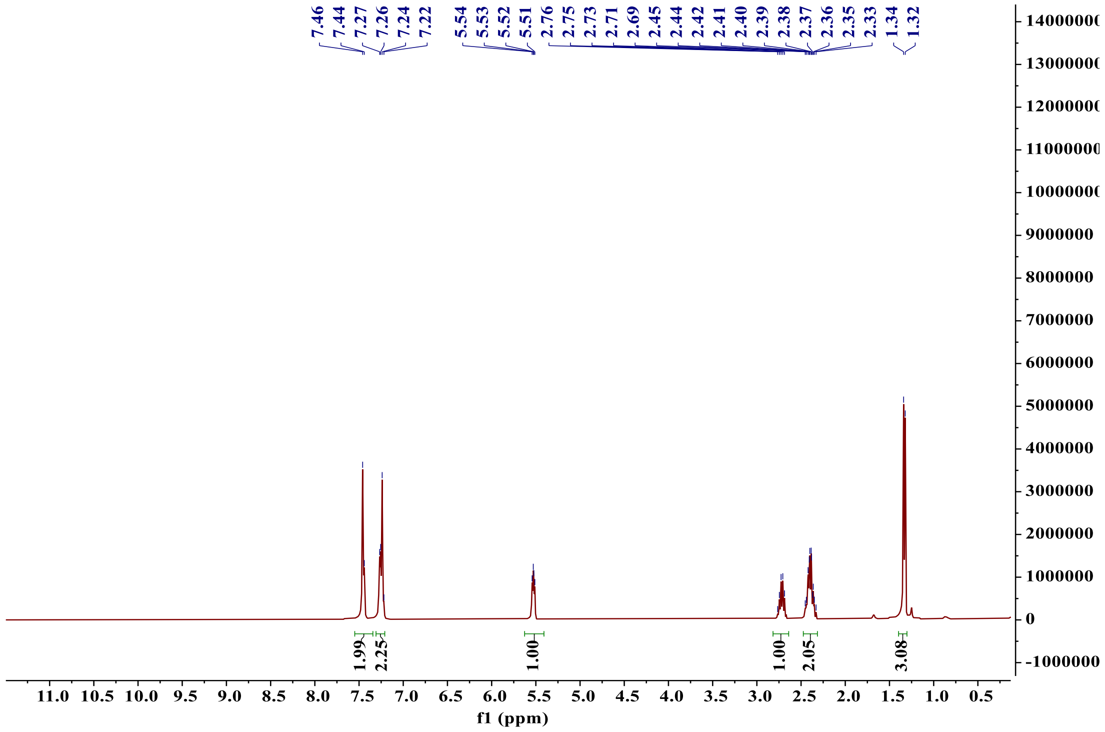

^{1}H NMR (400 MHz, $CDCl_3$) spectrum of **(*S*,*R*)-3q**

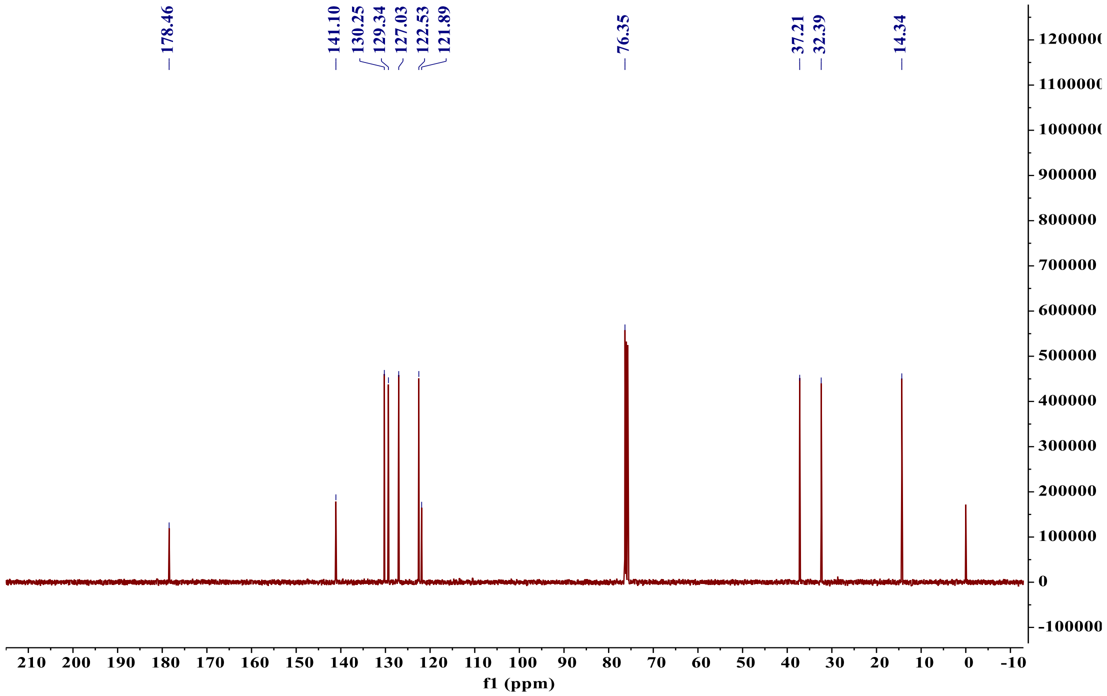

^{13}C NMR (101 MHz, $CDCl_3$) spectrum of **(*S*,*R*)-3q**

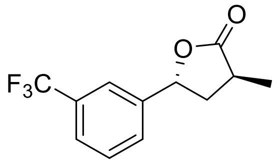

(*S*,*R*)-3r

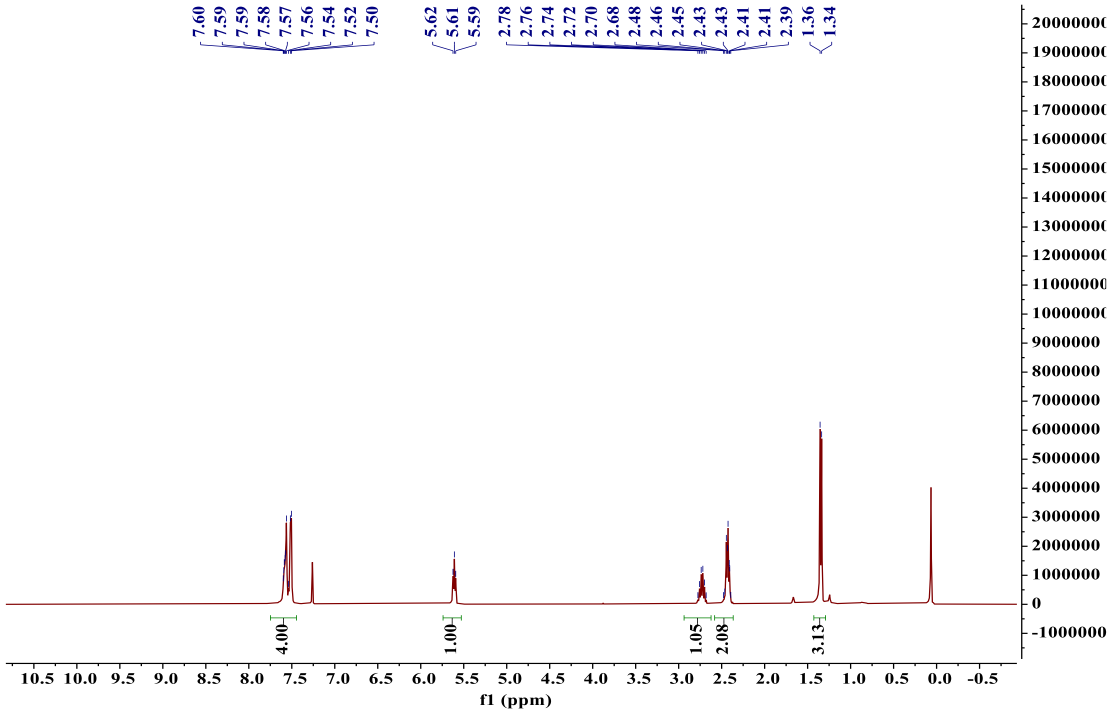

^{1}H NMR (400 MHz, $CDCl_3$) spectrum of **(*S*,*R*)-3r**

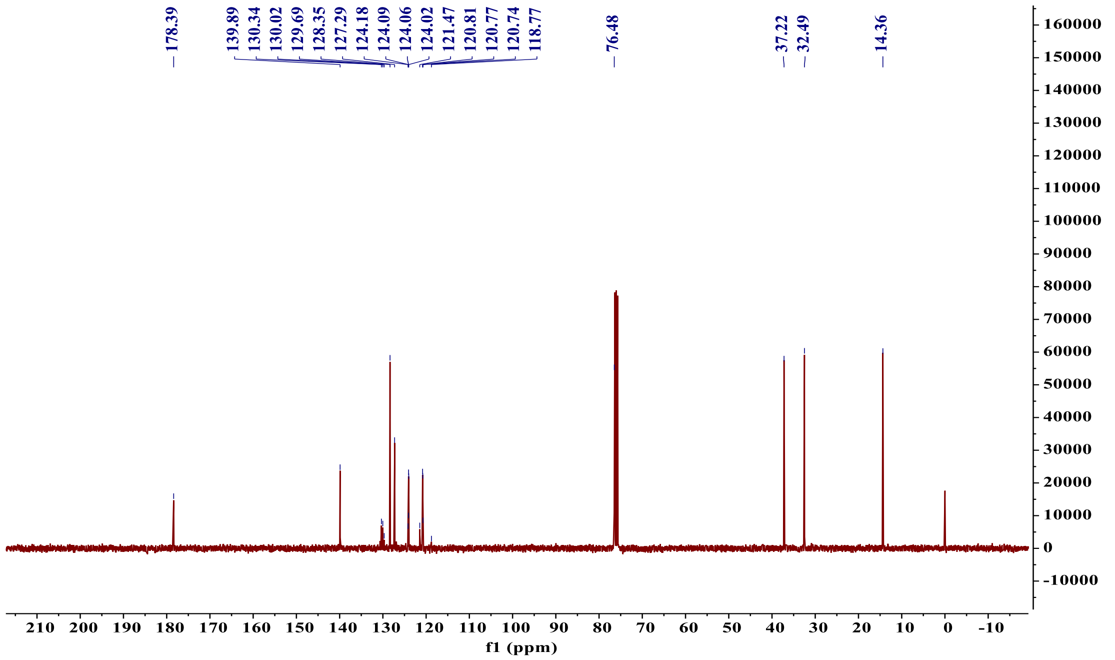

^{13}C NMR (101 MHz, $CDCl_3$) spectrum of **(*S*,*R*)-3r**

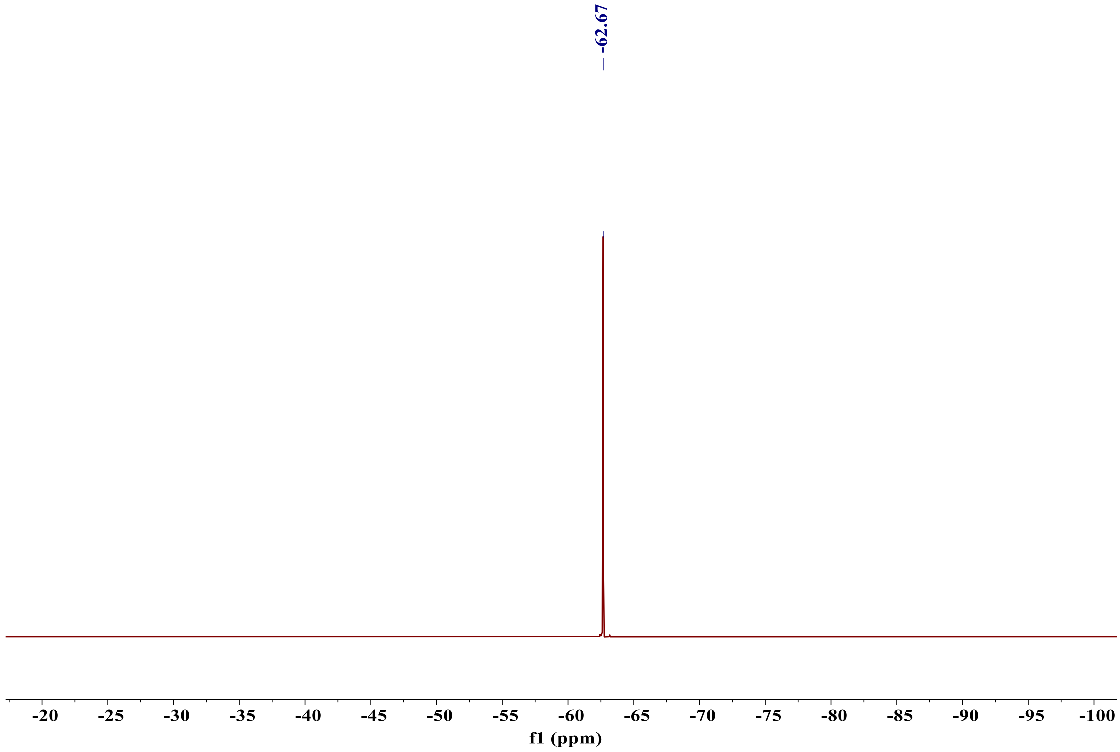

^{19}F NMR (377 MHz, $CDCl_3$) spectrum of **(*S*,*R*)-3r**

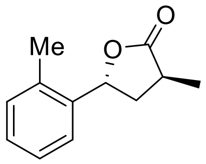

(*S*,*R*)-3s

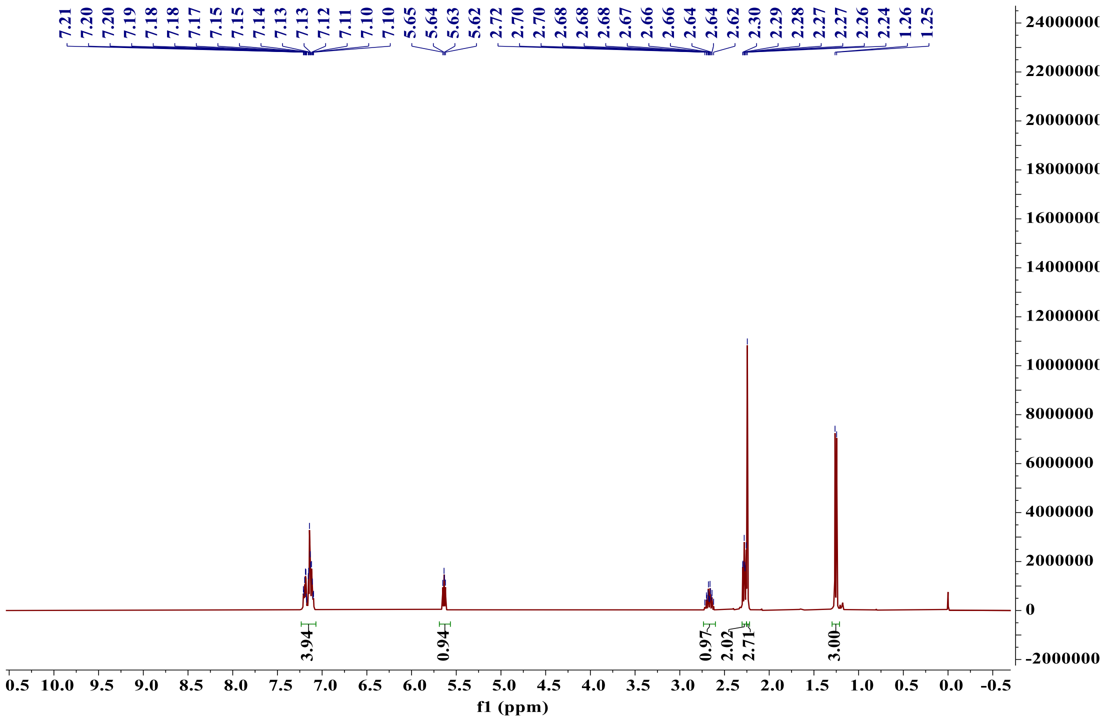

^{1}H NMR (400 MHz, CDCl$_3$) spectrum of **(*S*,*R*)-3s**

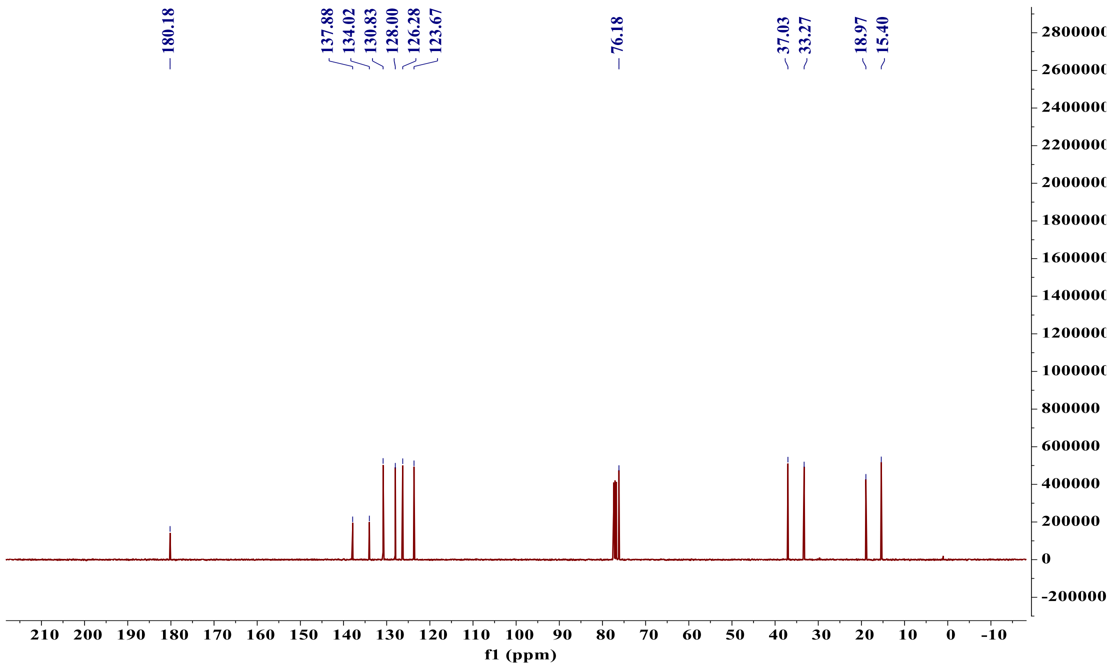

^{13}C NMR (101 MHz, CDCl$_3$) spectrum of **(*S*,*R*)-3s**

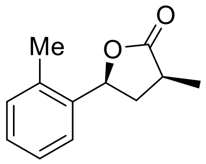

(*S*,*S*)-3s

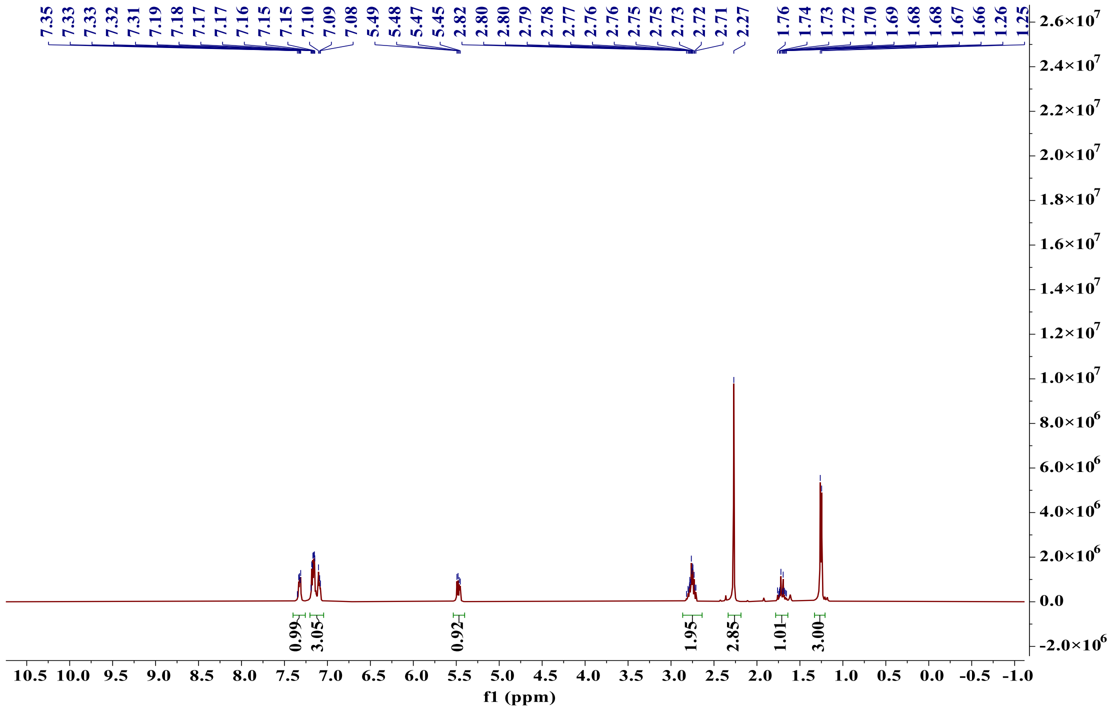

^{1}H NMR (400 MHz, $CDCl_3$) spectrum of **(*S*,*S*)-3s**

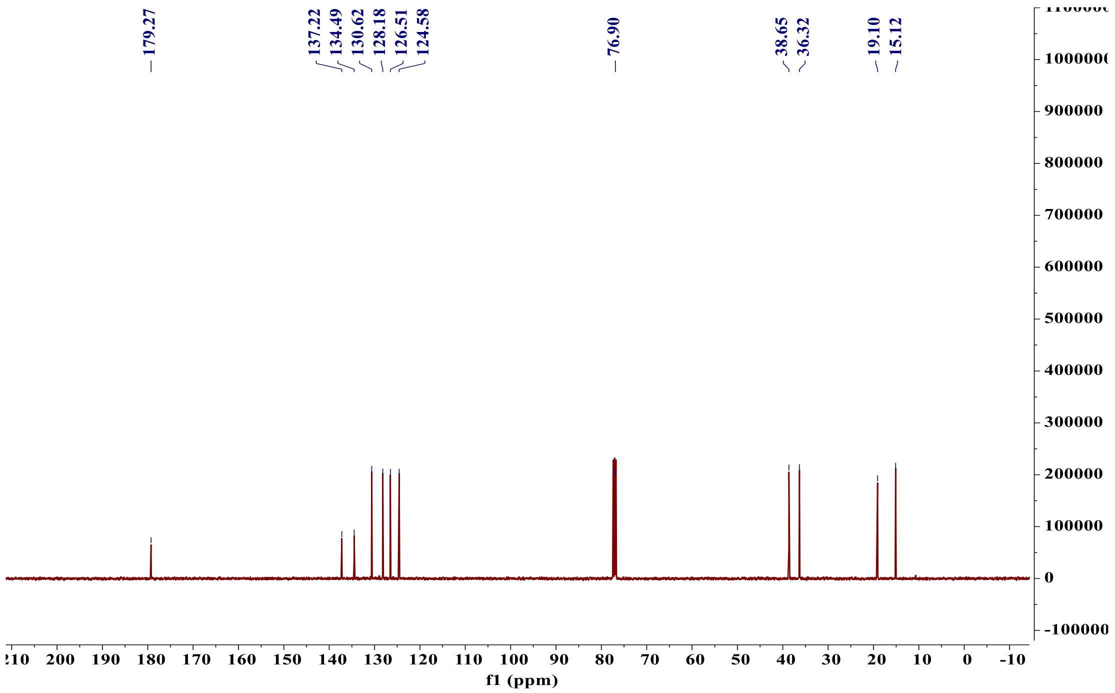

^{13}C NMR (101 MHz, $CDCl_3$) spectrum of **(*S*,*S*)-3s**

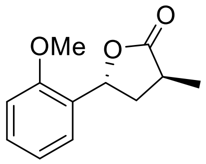

(***S***,***R***)-3t

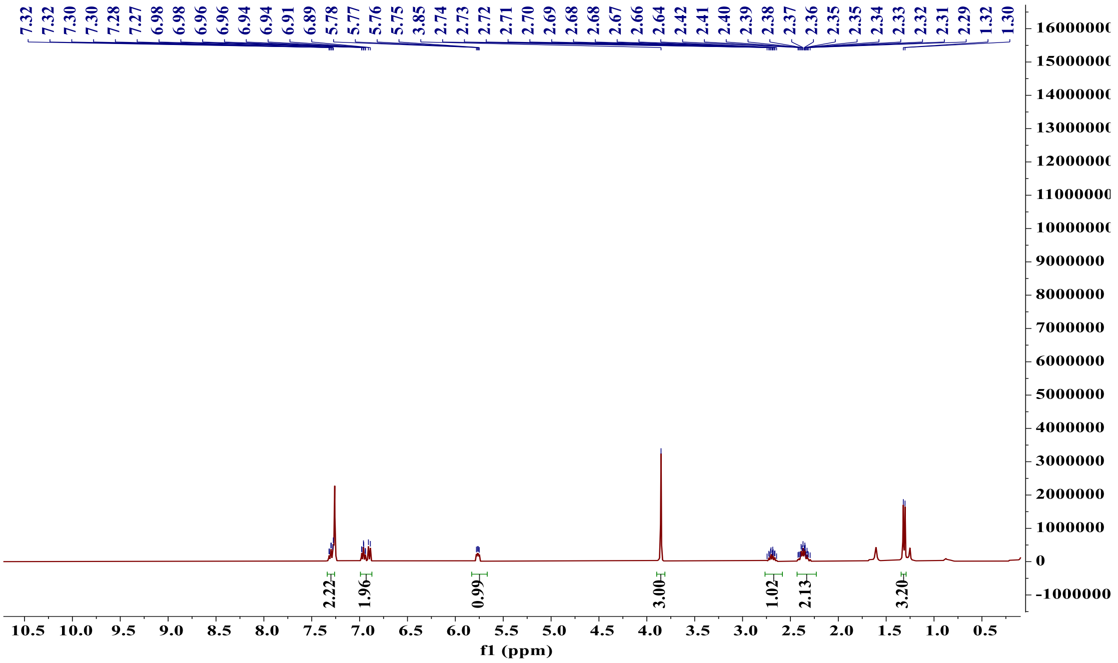

^{1}H NMR (400 MHz, $CDCl_3$) spectrum of **(*S*,*R*)-3t**

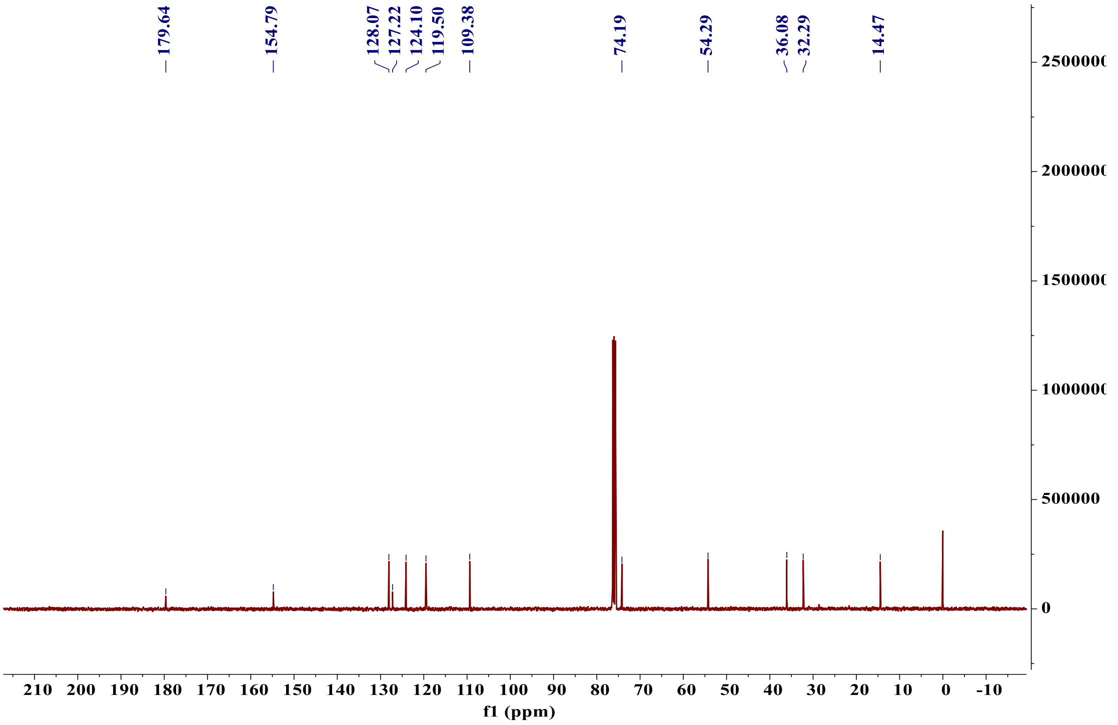

^{13}C NMR (101 MHz, $CDCl_3$) spectrum of **(*S*,*R*)-3t**

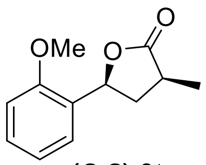

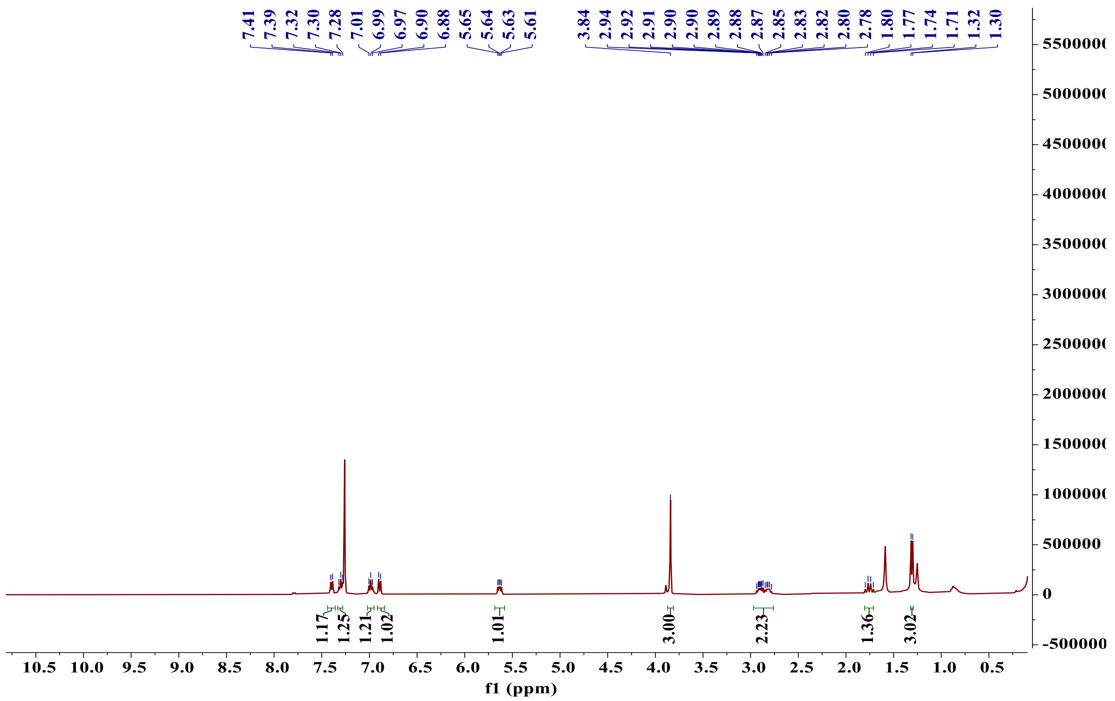

^{1}H NMR (400 MHz, $CDCl_3$) spectrum of ***(S,S)*-3t**

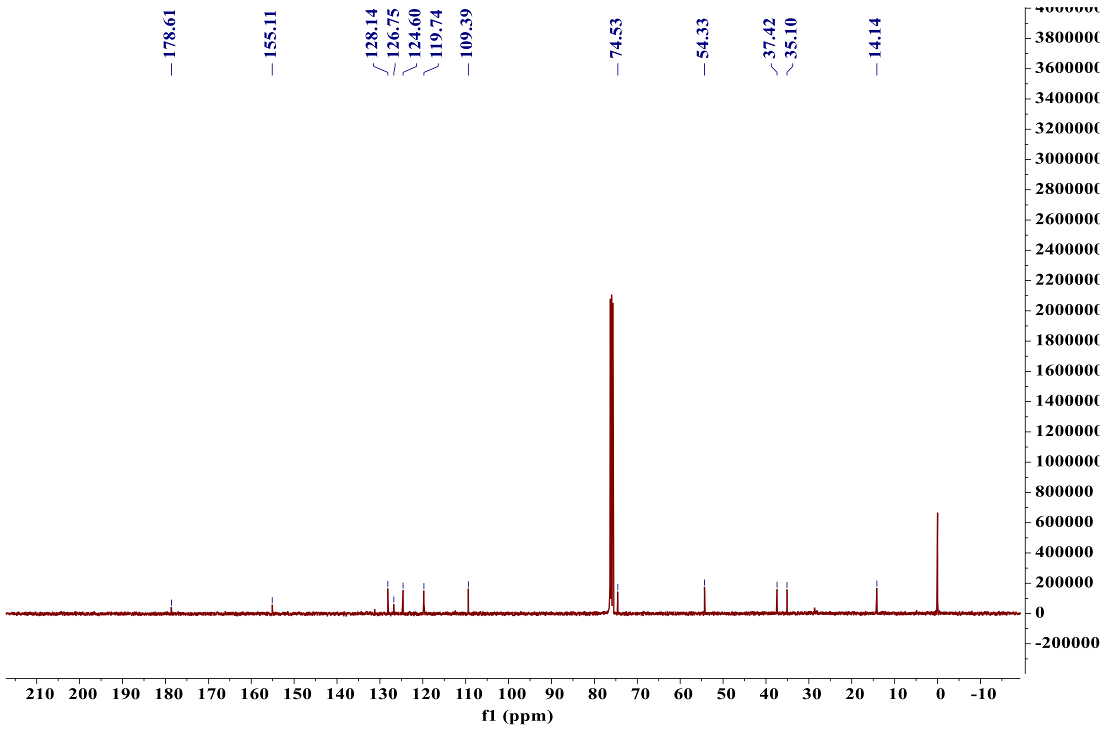

^{13}C NMR (101 MHz, $CDCl_3$) spectrum of ***(S,S)*-3t**

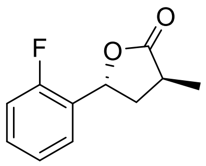

(*S*,*R*)-3u

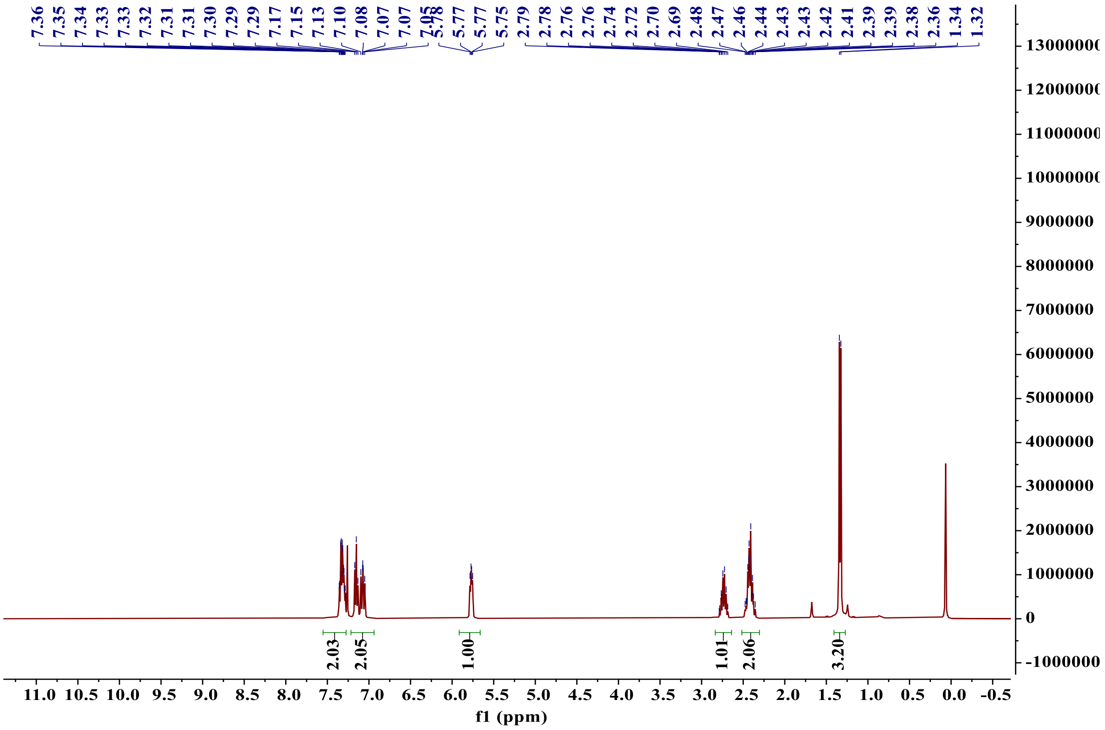

^{1}H NMR (400 MHz, $CDCl_3$) spectrum of **(*S*,*R*)-3u**

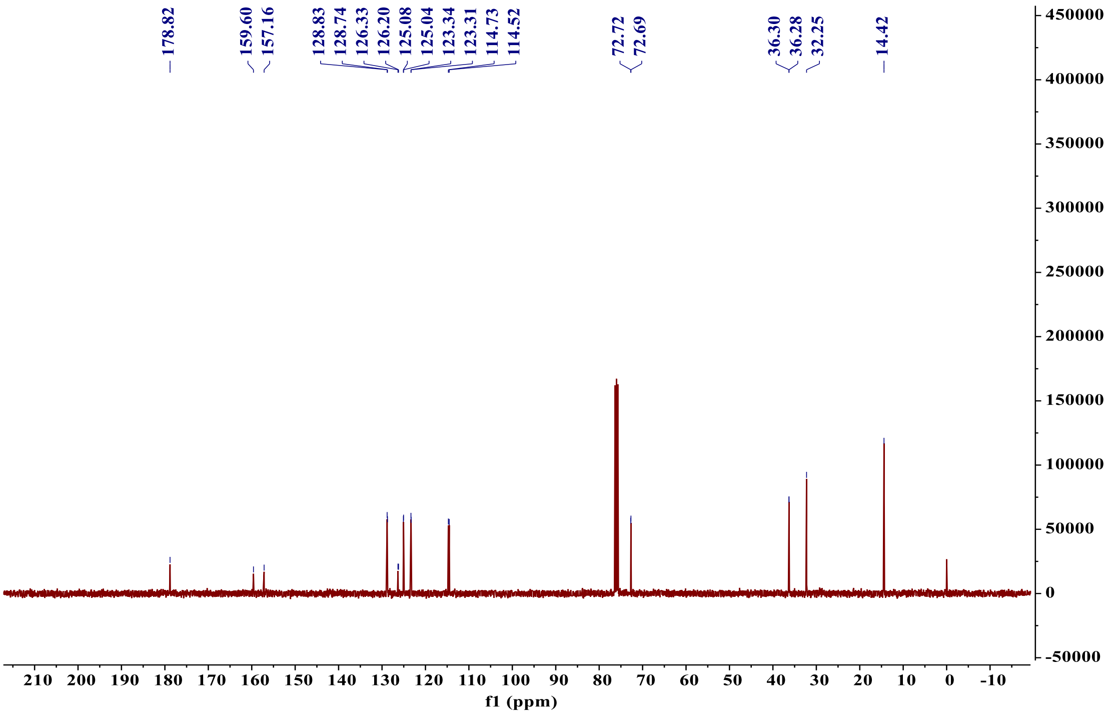

^{13}C NMR (101 MHz, $CDCl_3$) spectrum of **(*S*,*R*)-3u**

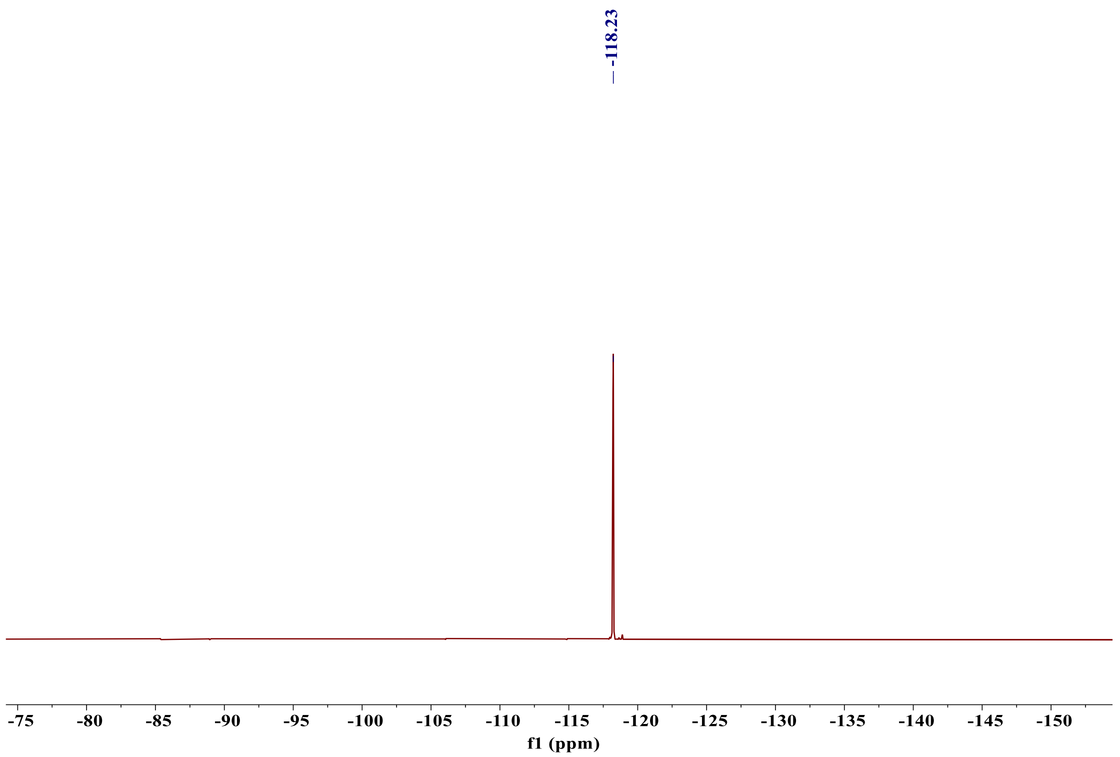

^{19}F NMR (101 MHz, $CDCl_3$) spectrum of **(*S*,*R*)-3u**

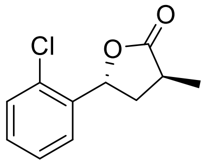

(*S*,*R*)-3v

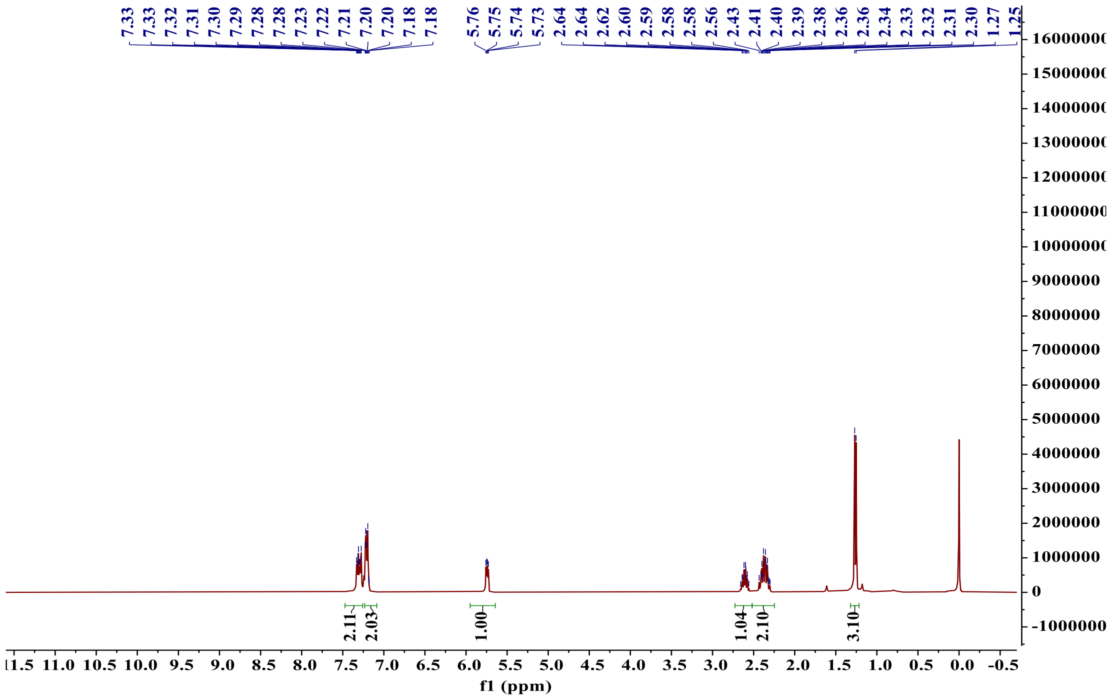

^{1}H NMR (400 MHz, $CDCl_3$) spectrum of **(*S*,*R*)-3v**

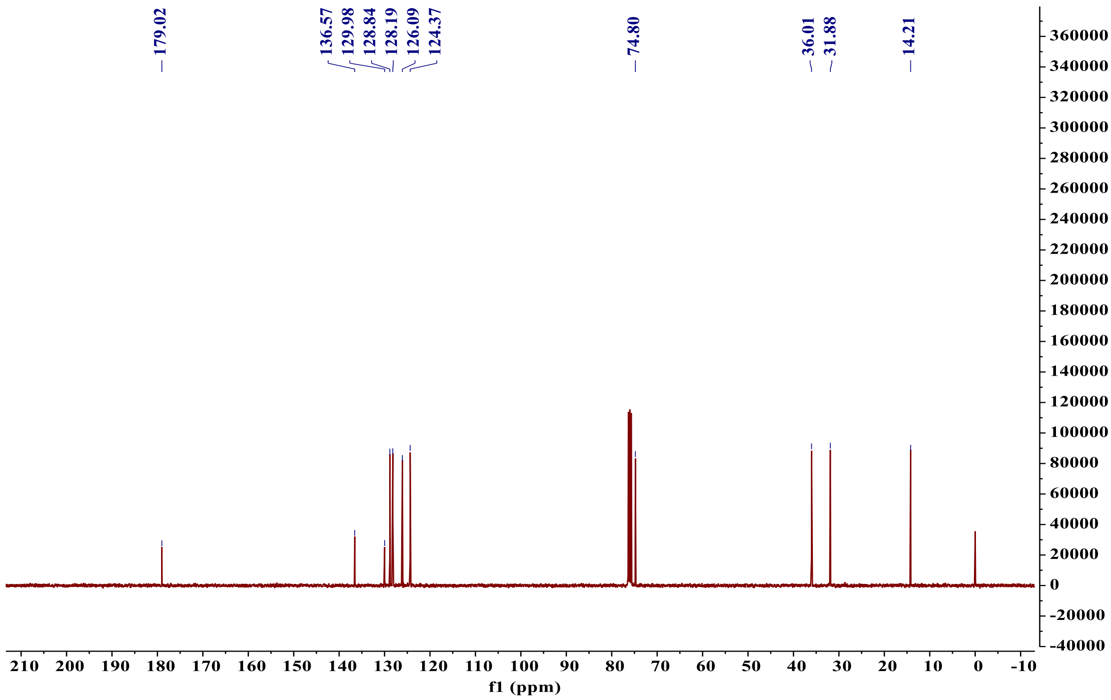

^{13}C NMR (101 MHz, $CDCl_3$) spectrum of **(*S*,*R*)-3v**

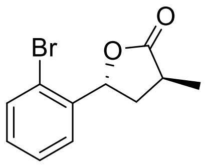

(*S*,*R*)-3w

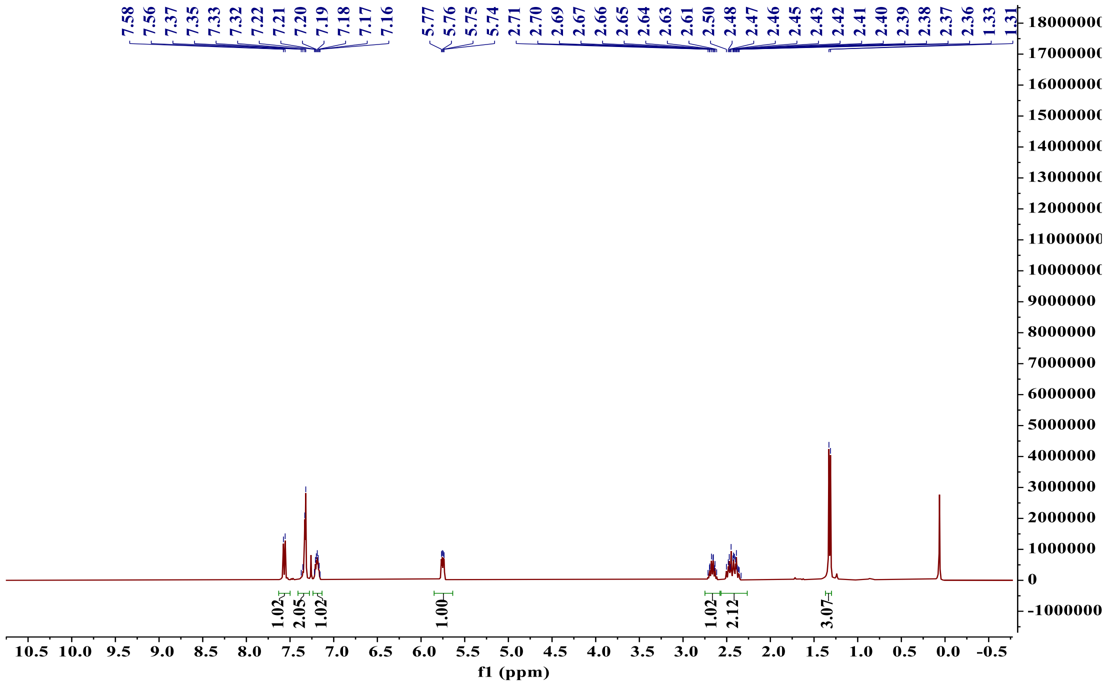

^{1}H NMR (400 MHz, $CDCl_3$) spectrum of **(*S*,*R*)-3w**

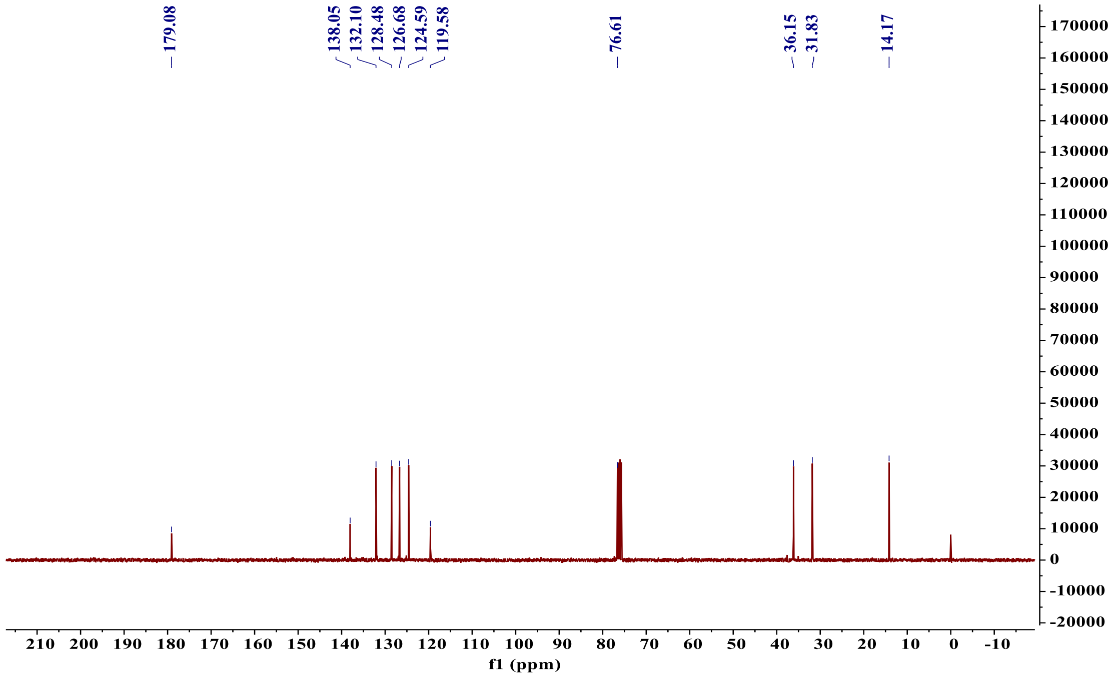

^{13}C NMR (101 MHz, $CDCl_3$) spectrum of **(*S*,*R*)-3w**

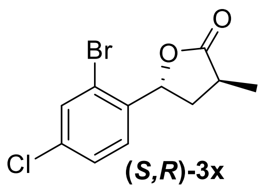

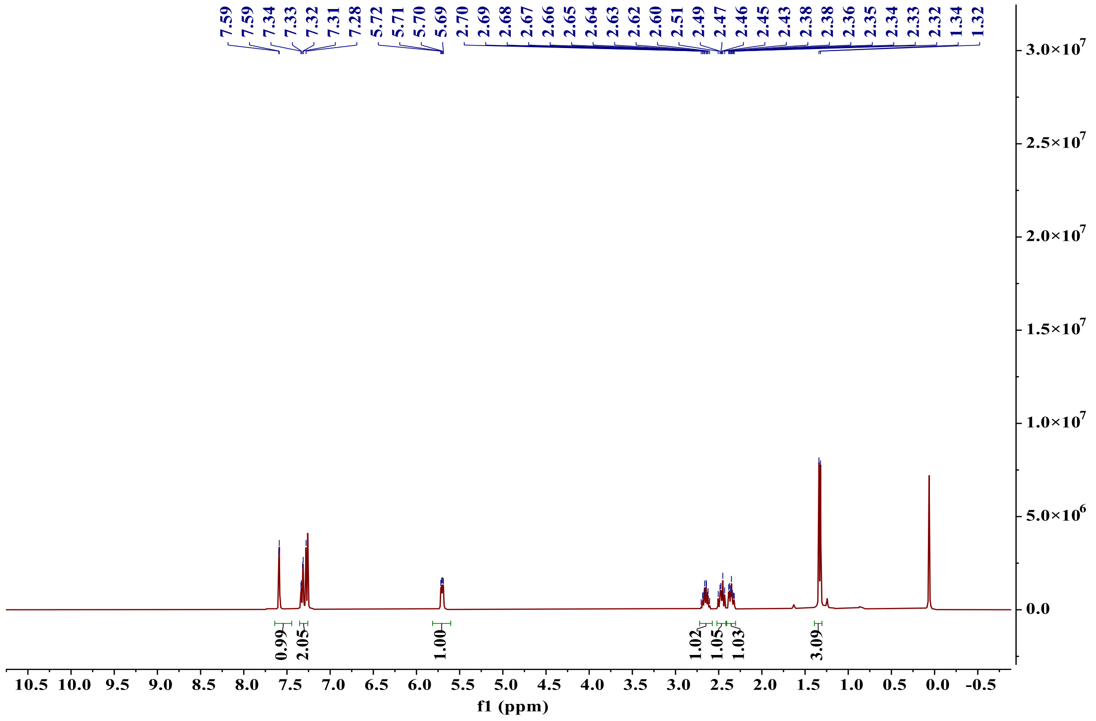

^{1}H NMR (400 MHz, $CDCl_3$) spectrum of ***(S,R)*-3x**

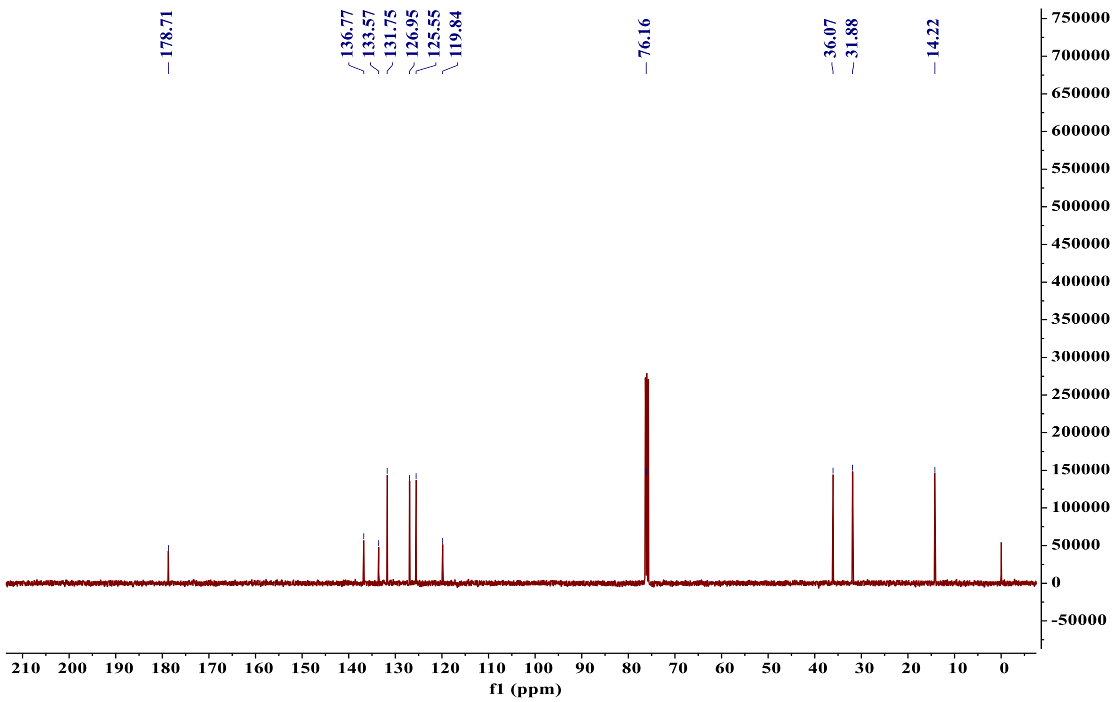

^{13}C NMR (101 MHz, $CDCl_3$) spectrum of ***(S,R)*-3x**

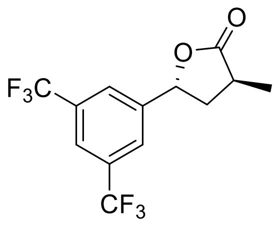

(*S*,*R*)-3y

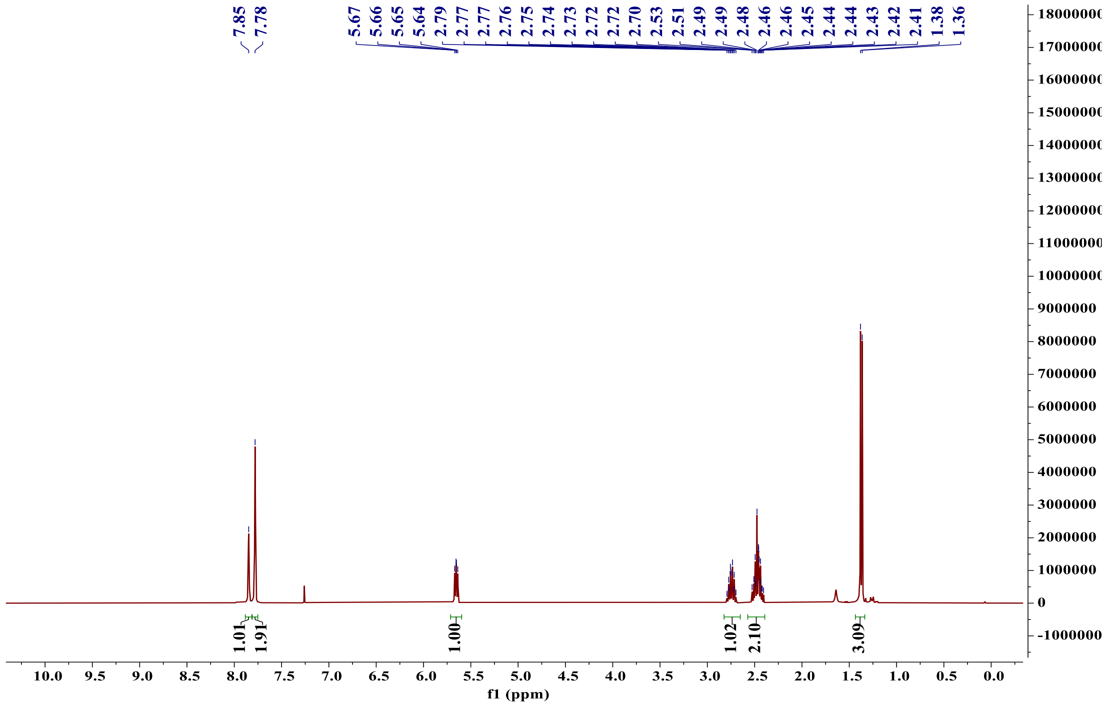

^{1}H NMR (400 MHz, $CDCl_3$) spectrum of **(*S*,*R*)-3y**

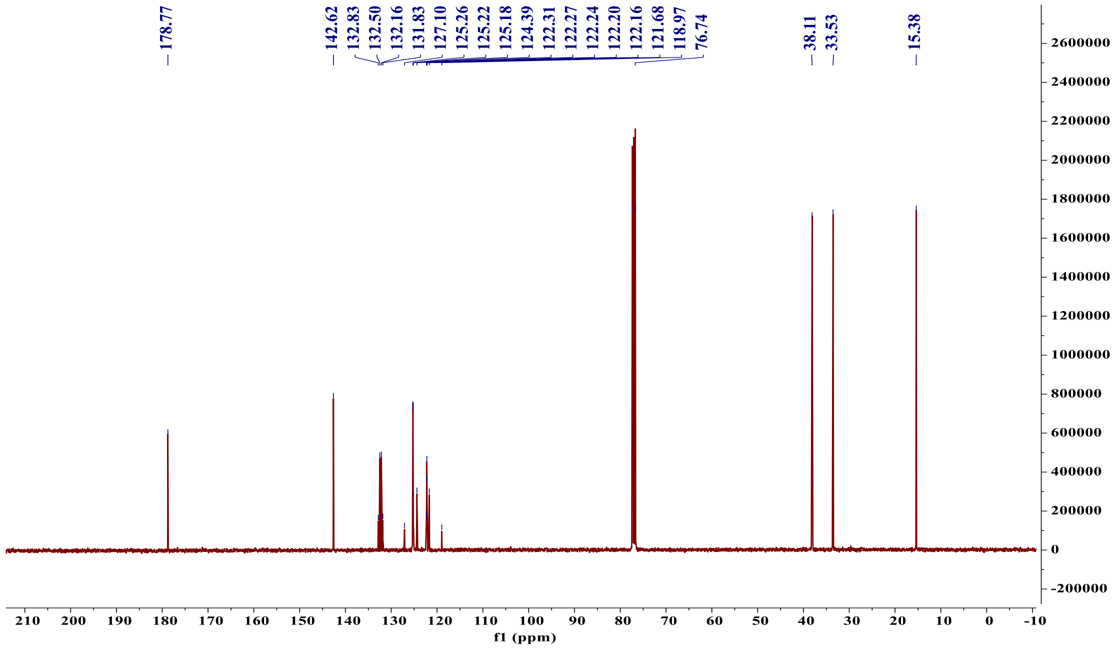

^{13}C NMR (101 MHz, $CDCl_3$) spectrum of **(*S*,*R*)-3y**

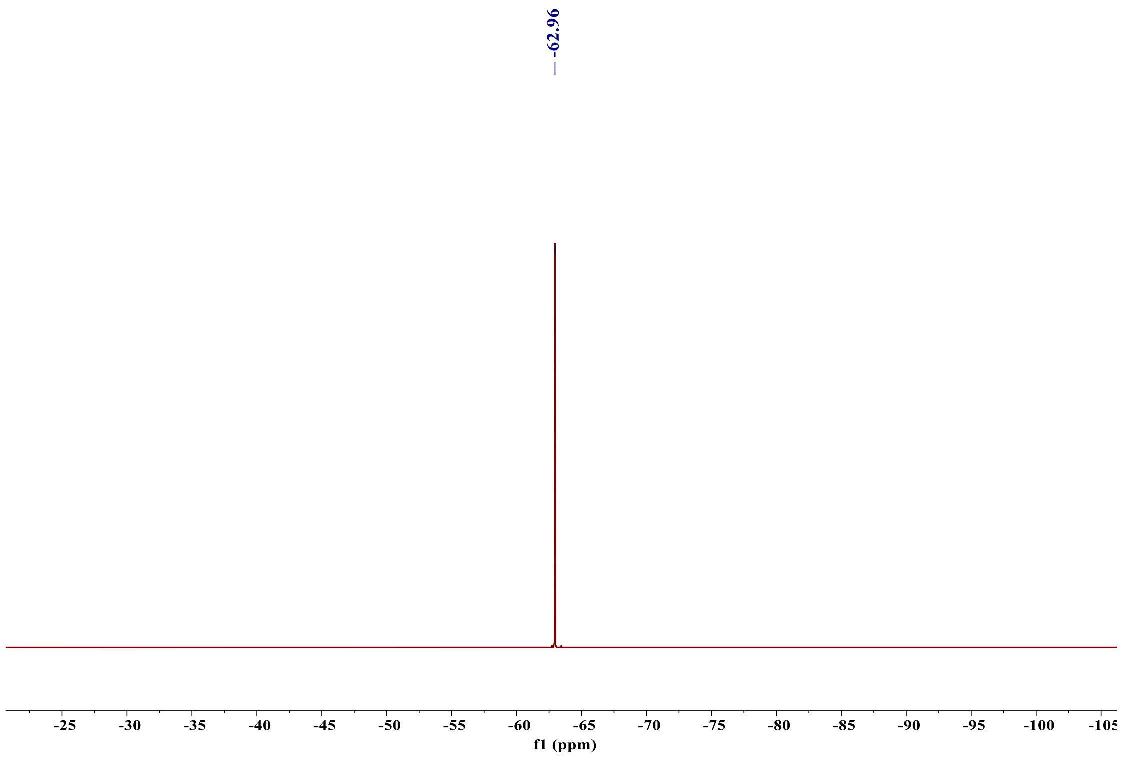

^{19}F NMR (101 MHz, $CDCl_3$) spectrum of ***(S,R)*-3y**

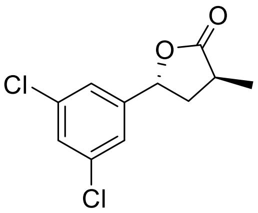

(*S*,*R*)-3z

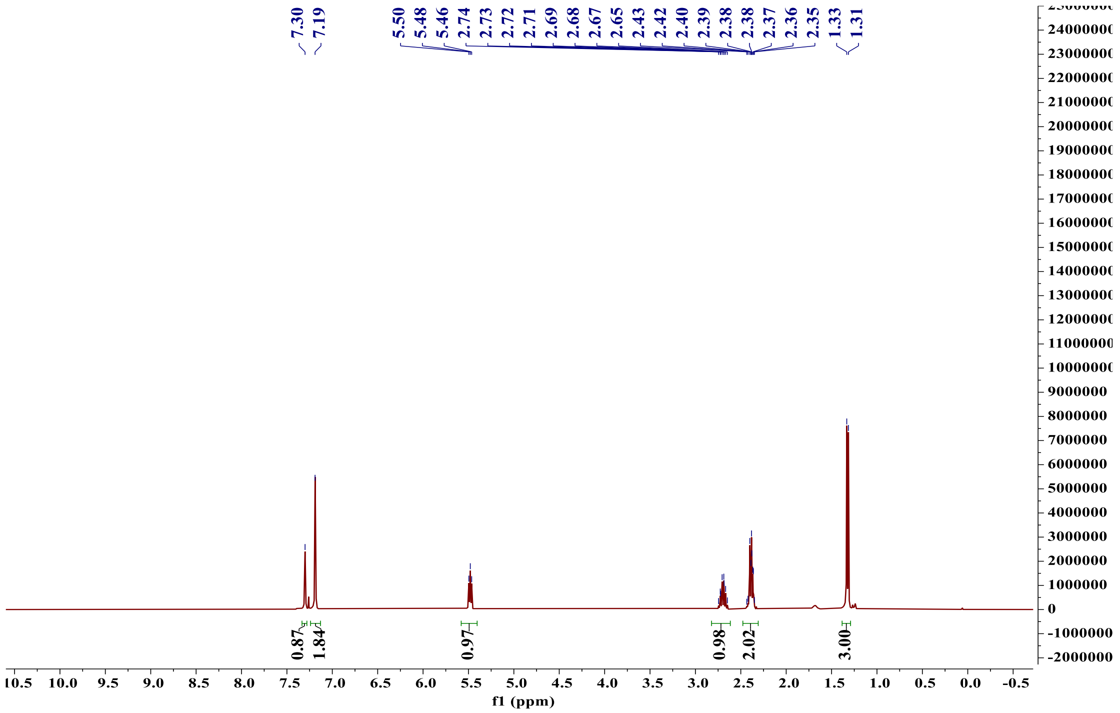

^{1}H NMR (400 MHz, $CDCl_3$) spectrum of **(*S*,*R*)-3z**

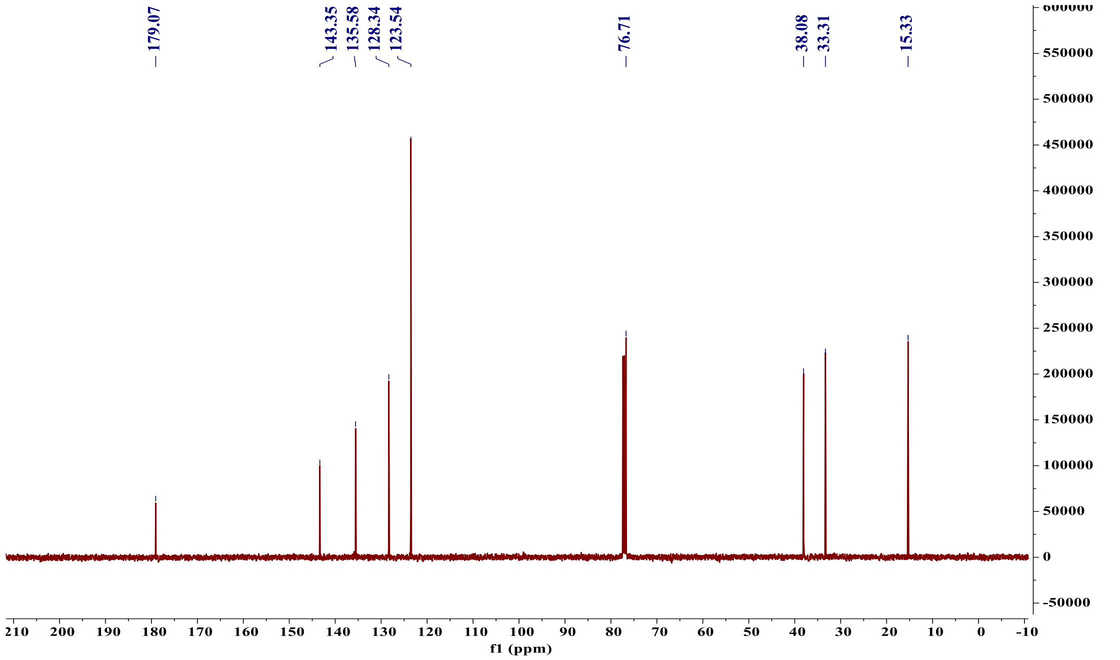

^{13}C NMR (101 MHz, $CDCl_3$) spectrum of **(*S*,*R*)-3z**

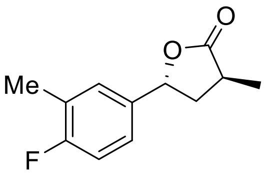

(*S*,*R*)-3aa

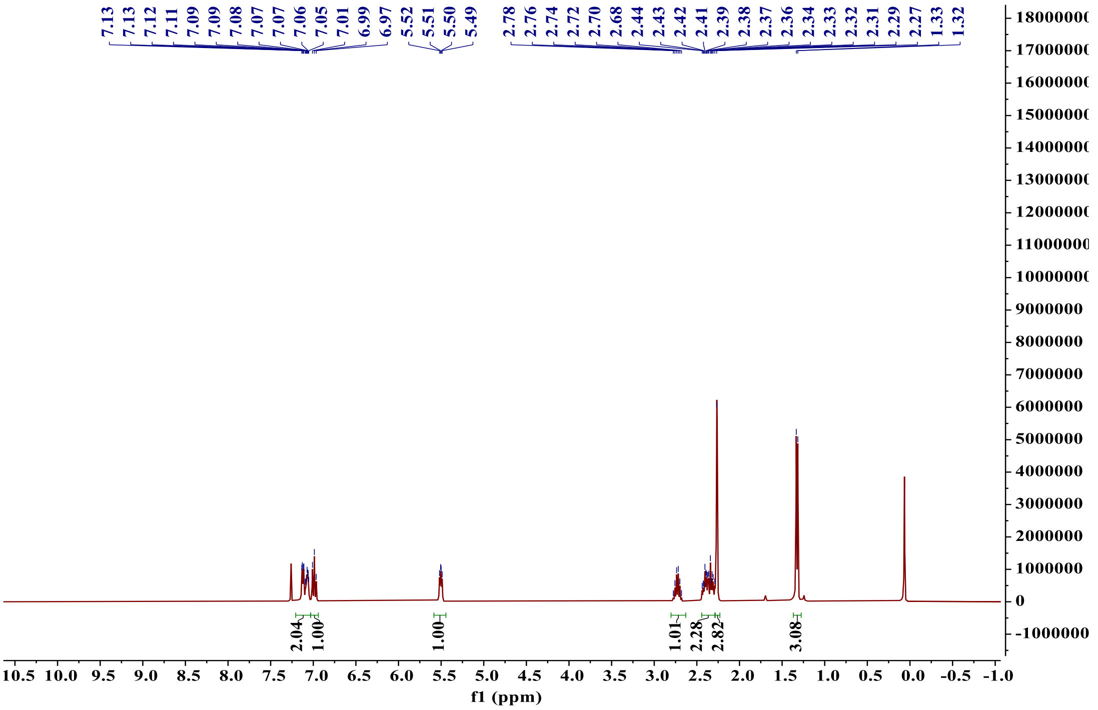

^{1}H NMR (400 MHz, $CDCl_3$) spectrum of **(*S*,*R*)-3aa**

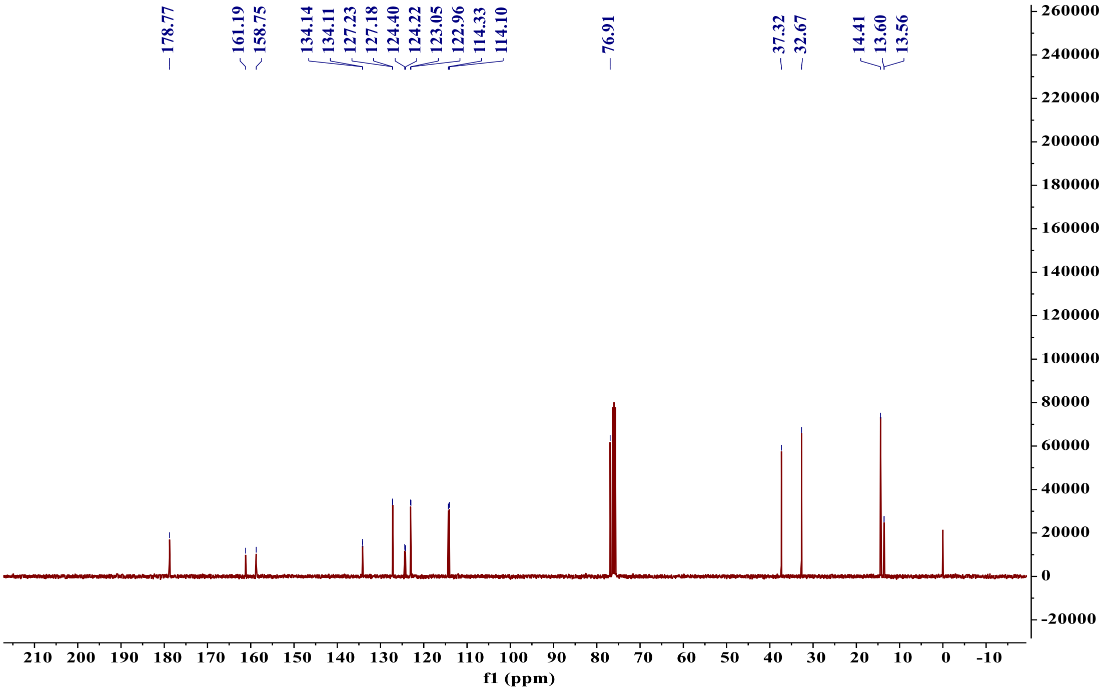

^{13}C NMR (101 MHz, $CDCl_3$) spectrum of **(*S*,*R*)-3aa**

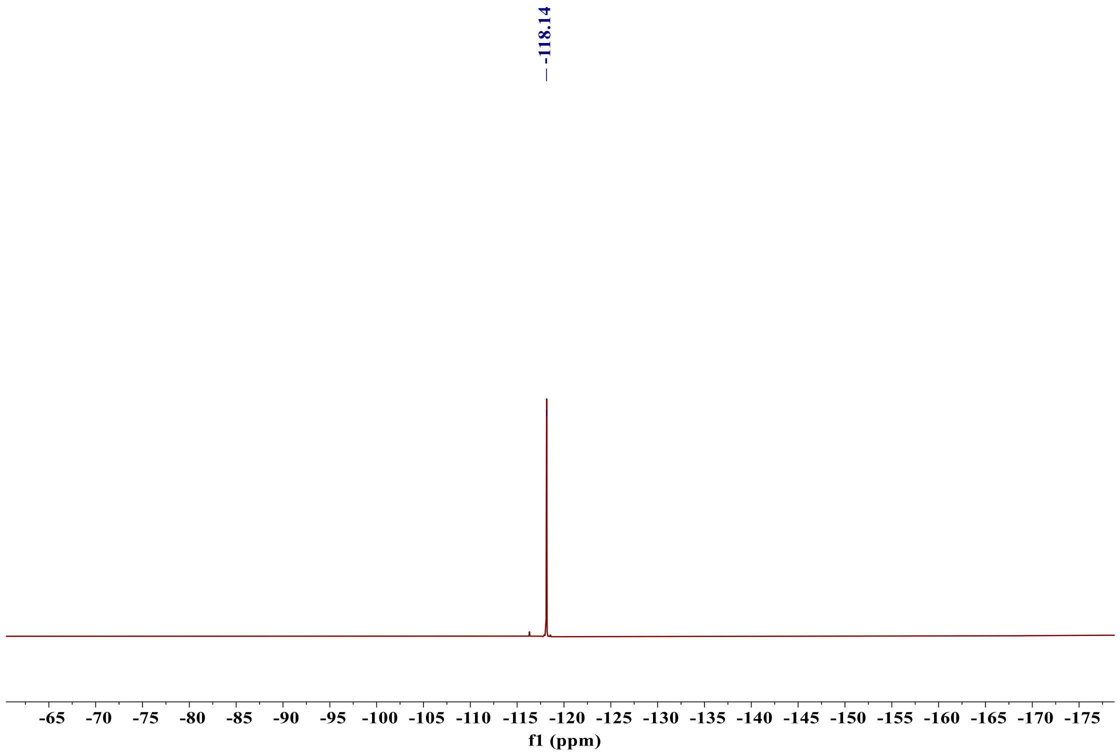

^{19}F NMR (101 MHz, $CDCl_3$) spectrum of ***(S,R)*-3aa**

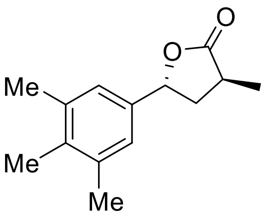

(*S*,*R*)-3ab

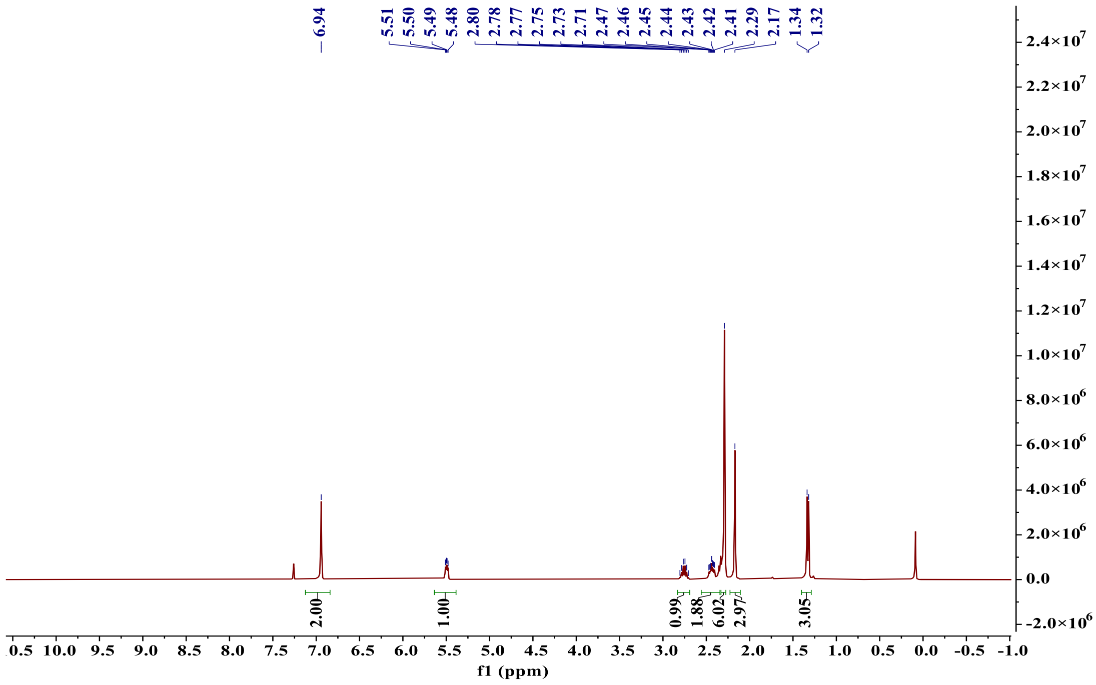

^{1}H NMR (400 MHz, $CDCl_3$) spectrum of **(*S*,*R*)-3ab**

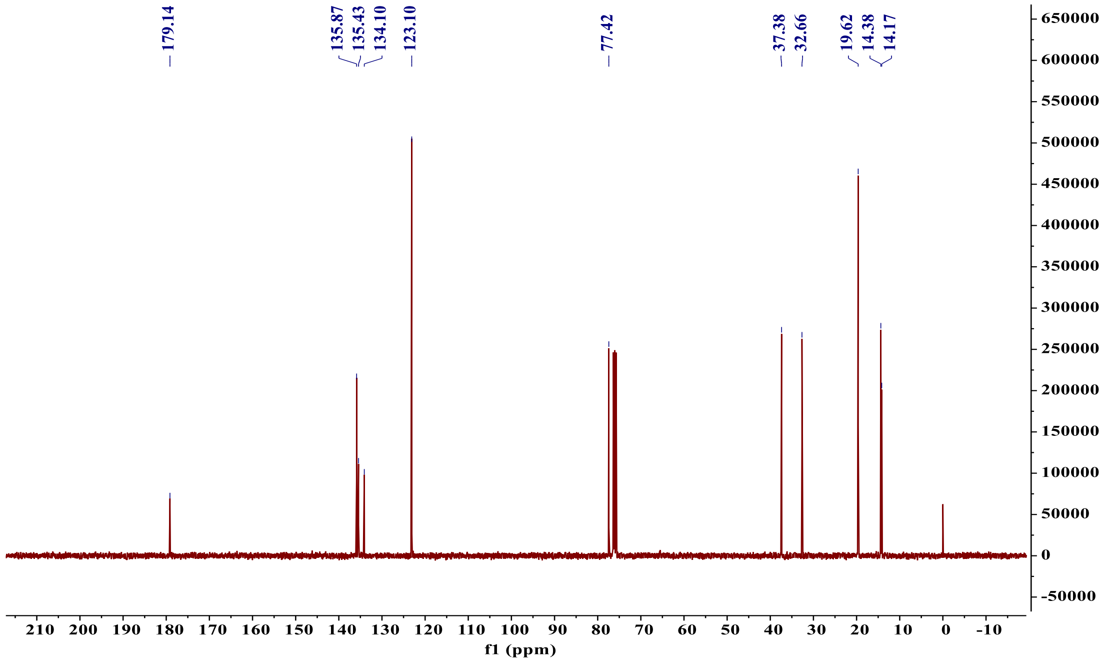

^{13}C NMR (101 MHz, $CDCl_3$) spectrum of **(*S*,*R*)-3ab**

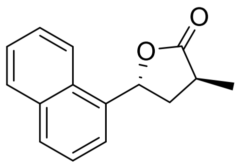

(*S*,*R*)-3ac

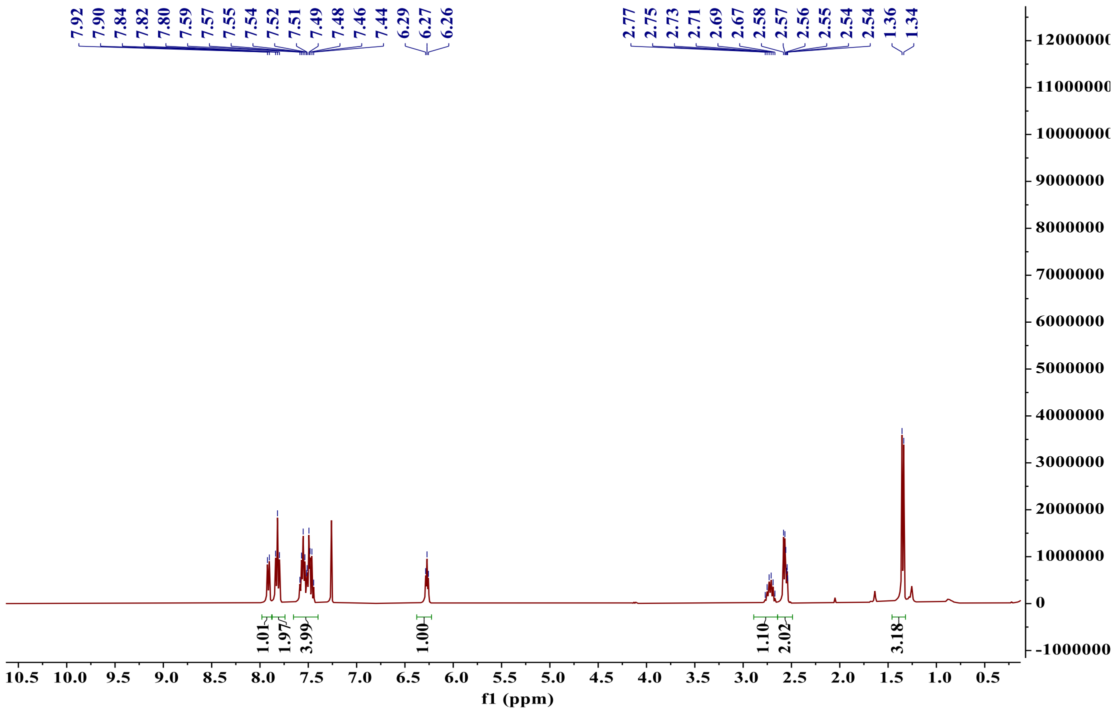

^{1}H NMR (400 MHz, $CDCl_3$) spectrum of **(*S*,*R*)-3ac**

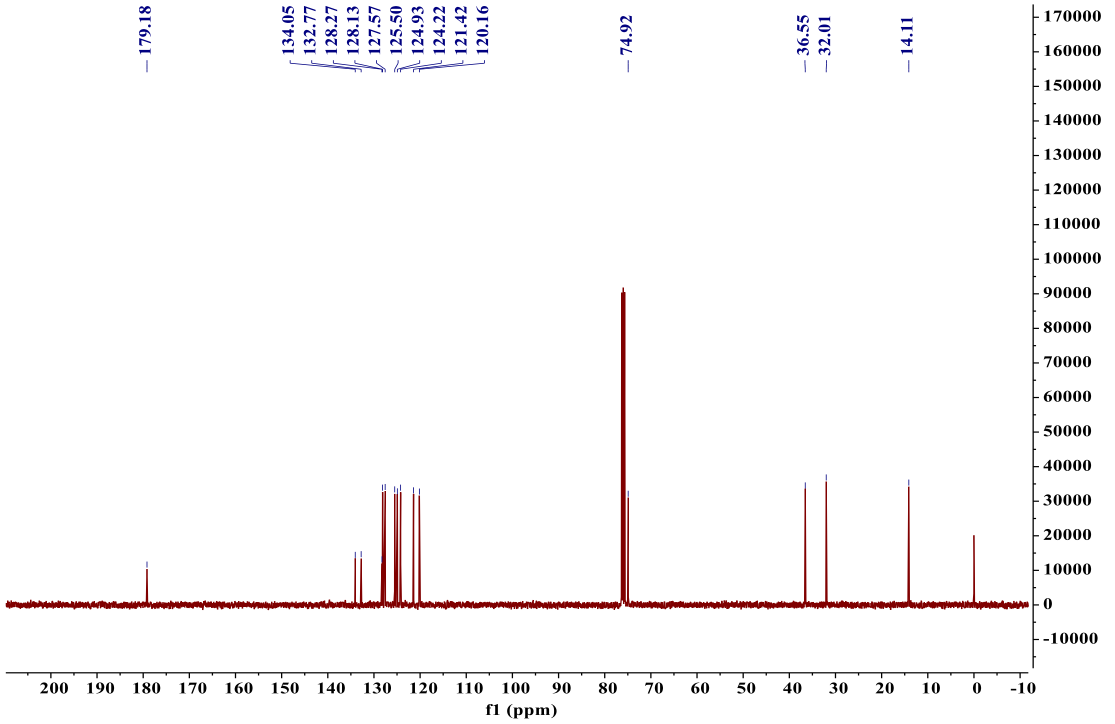

^{13}C NMR (101 MHz, $CDCl_3$) spectrum of **(*S*,*R*)-3ac**

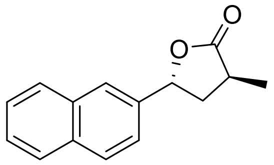

(*S*,*R*)-3ad

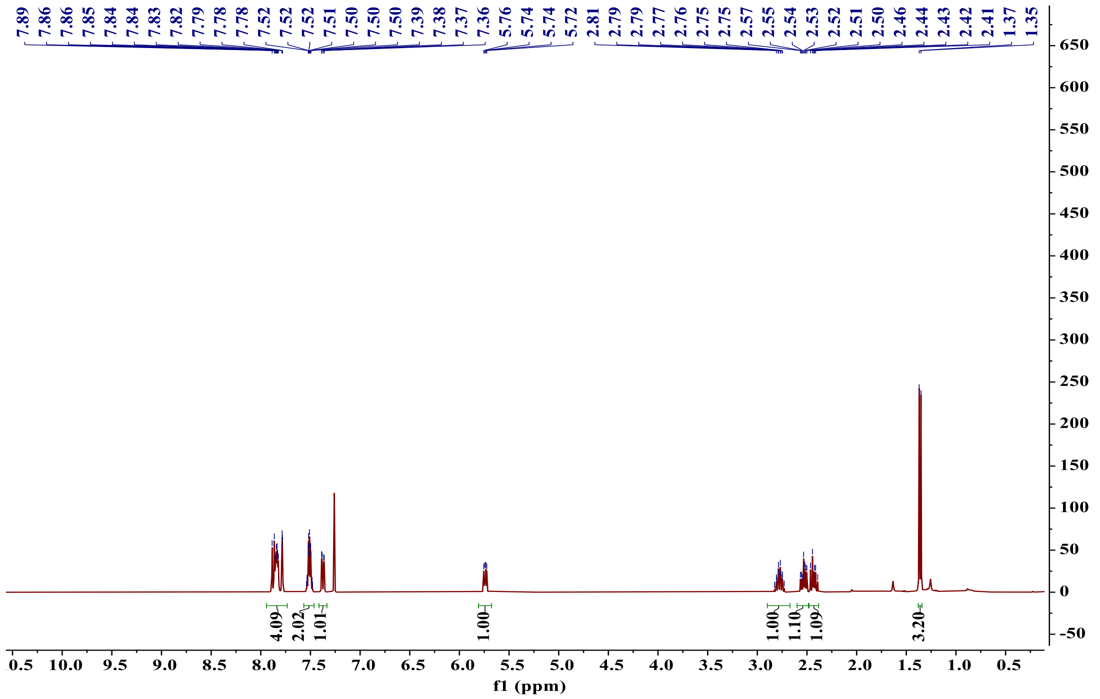

^{1}H NMR (400 MHz, $CDCl_3$) spectrum of **(*S*,*R*)-3ad**

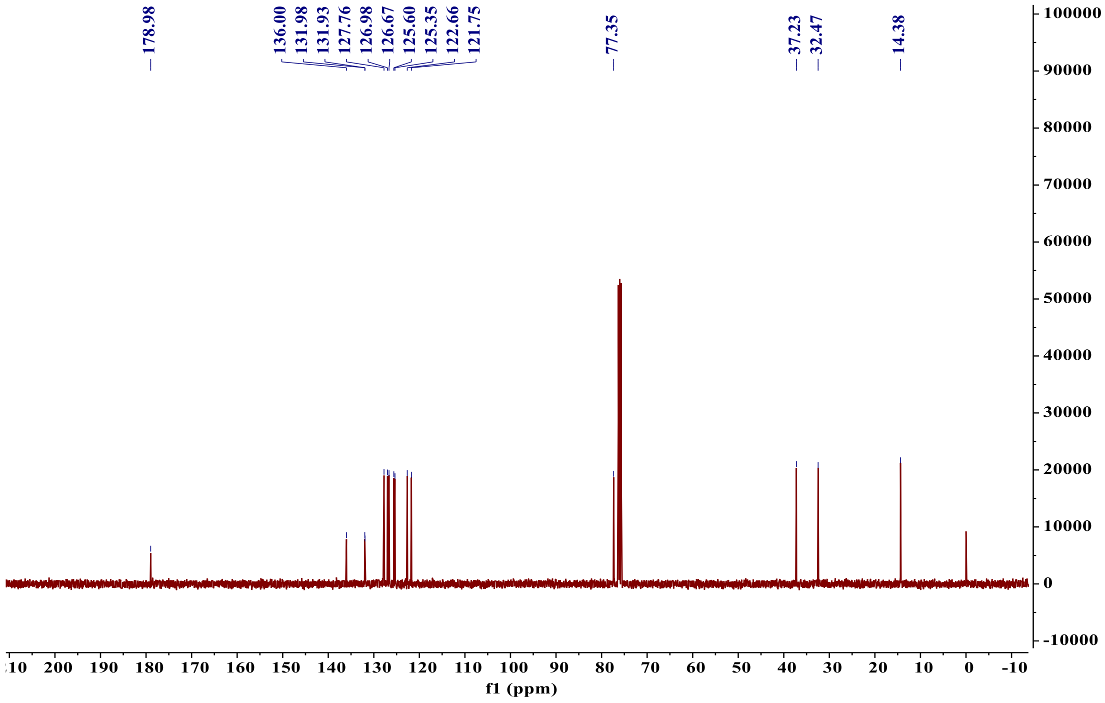

^{13}C NMR (101 MHz, $CDCl_3$) spectrum of **(*S*,*R*)-3ad**

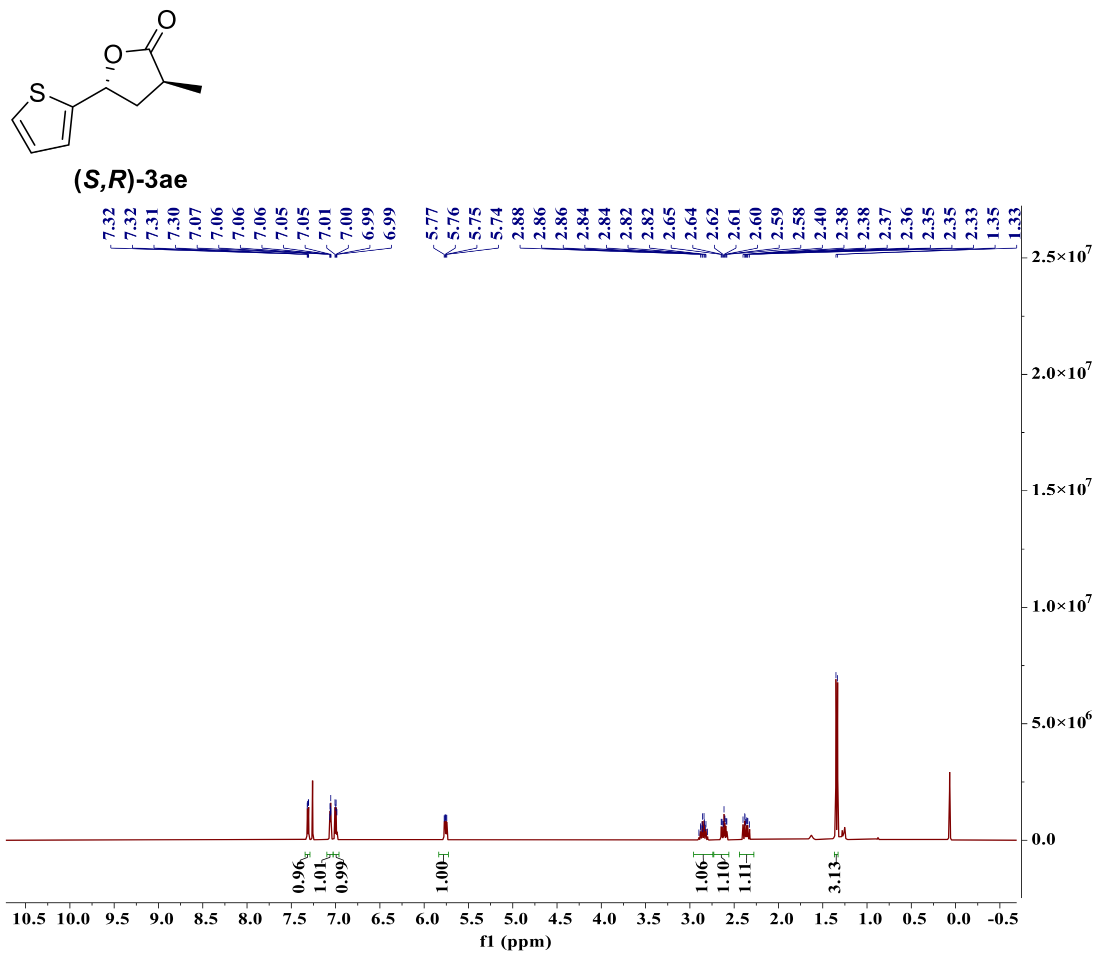

^{1}H NMR (400 MHz, $CDCl_3$) spectrum of ***(S,R)*-3ae**

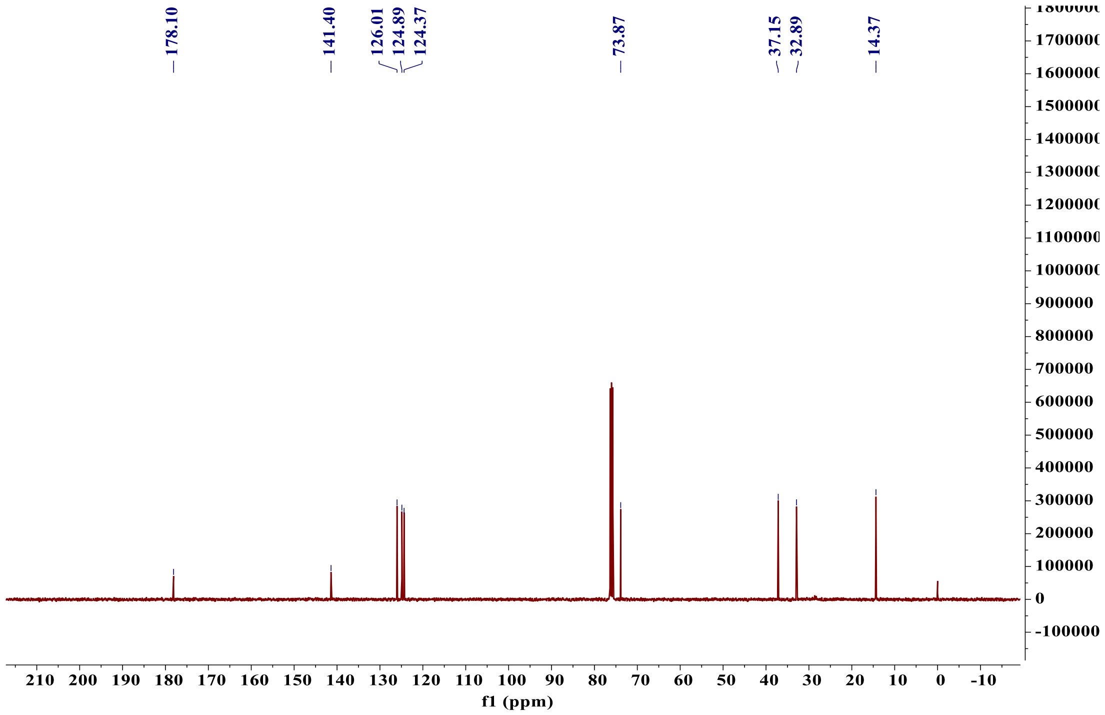

^{13}C NMR (101 MHz, $CDCl_3$) spectrum of ***(S,R)*-3ae**

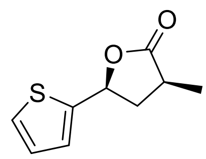

(*S*,*S*)-3ae

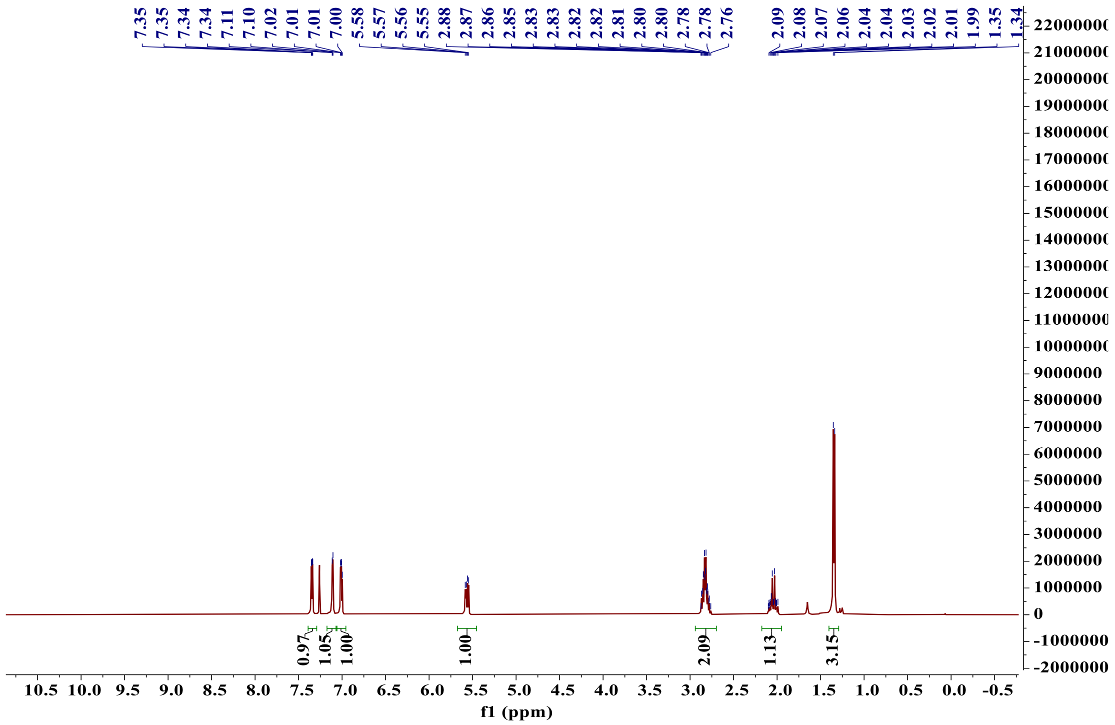

^{1}H NMR (400 MHz, $CDCl_3$) spectrum of **(*S*,*S*)-3ae**

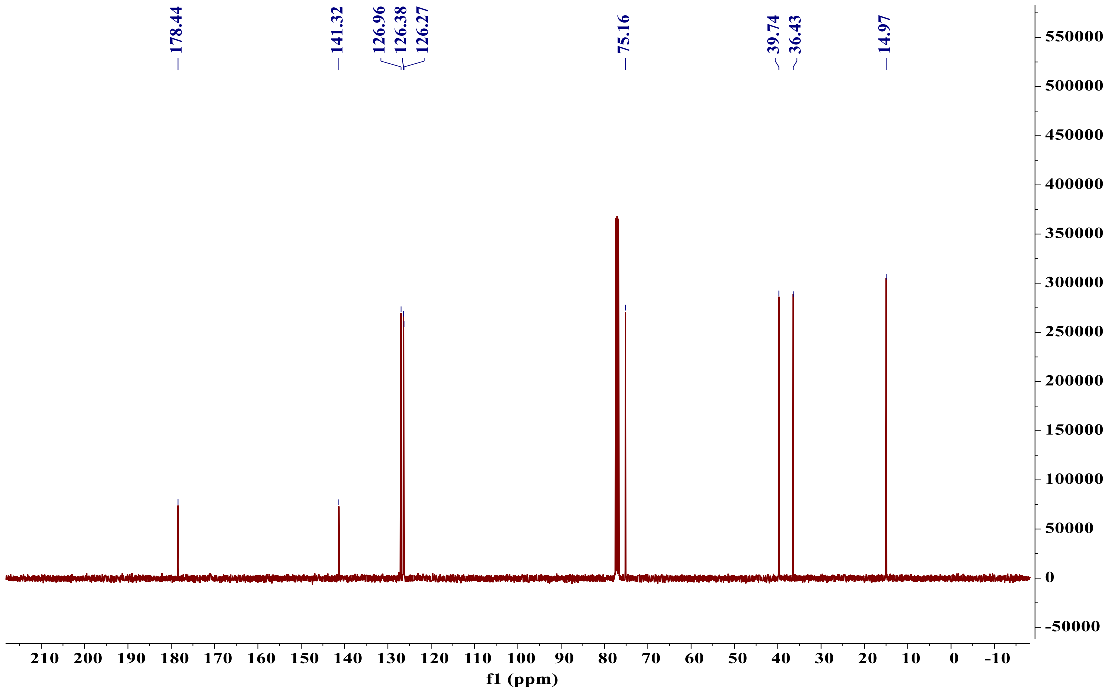

^{13}C NMR (101 MHz, $CDCl_3$) spectrum of **(*S*,*S*)-3ae**

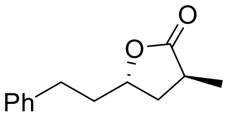

(*S*,*S*)-3af

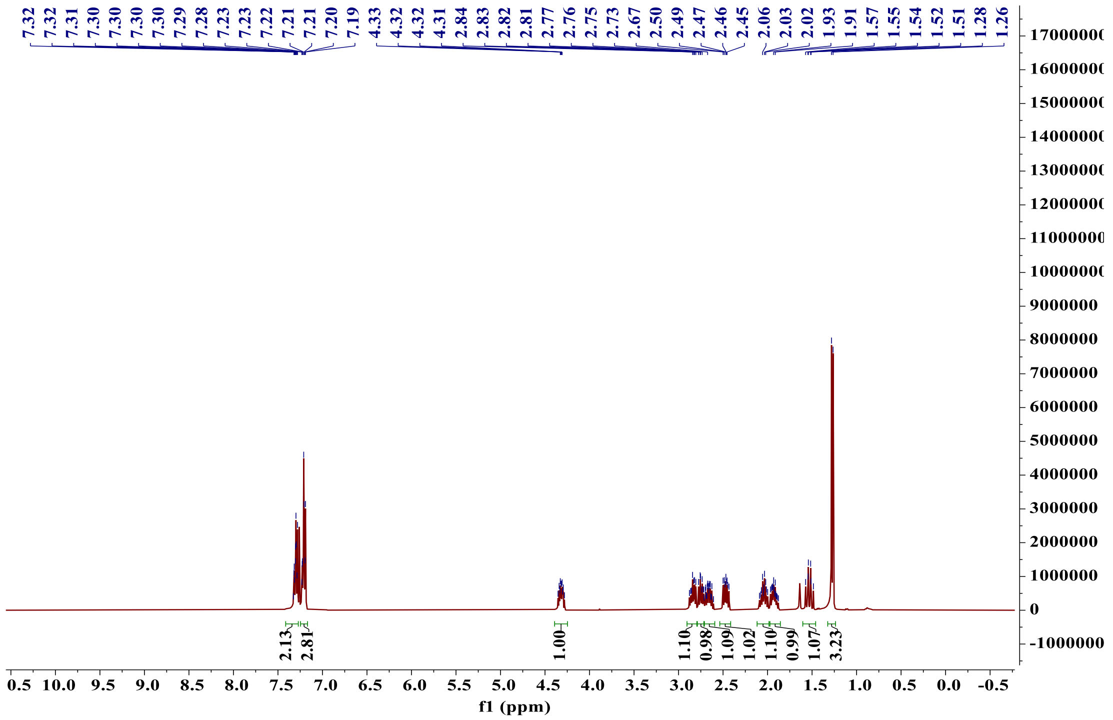

^{1}H NMR (400 MHz, $CDCl_3$) spectrum of **(*S*,*S*)-3af**

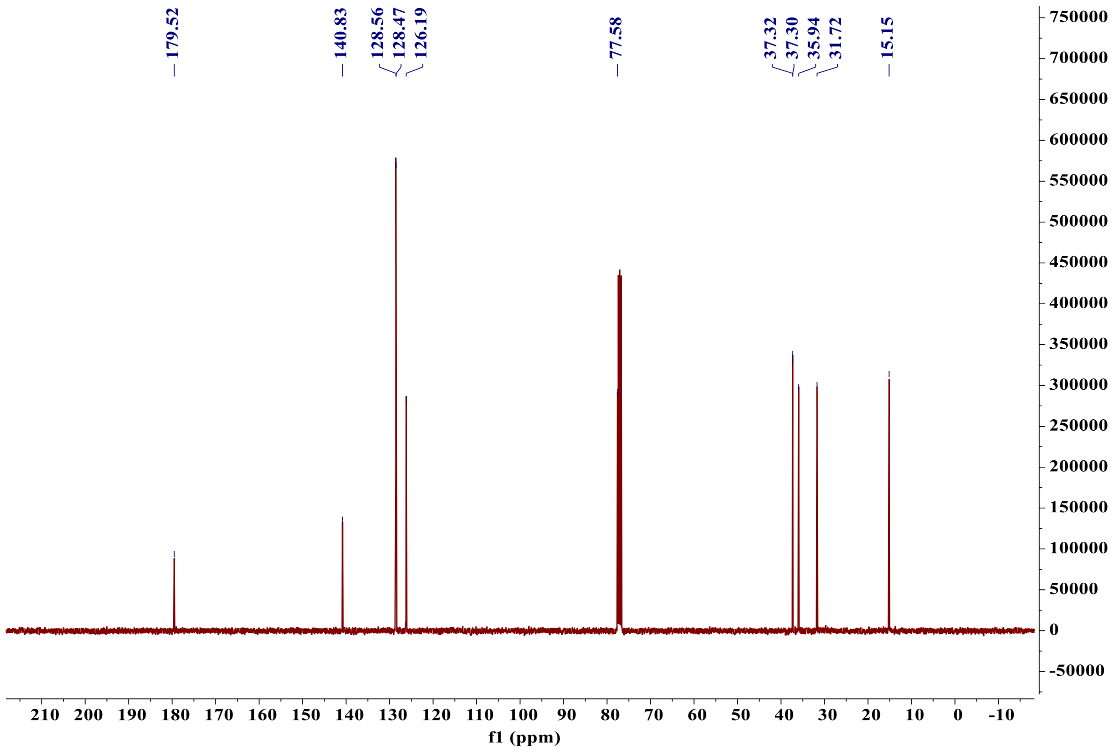

^{13}C NMR (101 MHz, $CDCl_3$) spectrum of **(*S*,*S*)-3af**

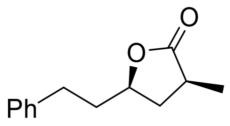

(*S*,*R*)-3af

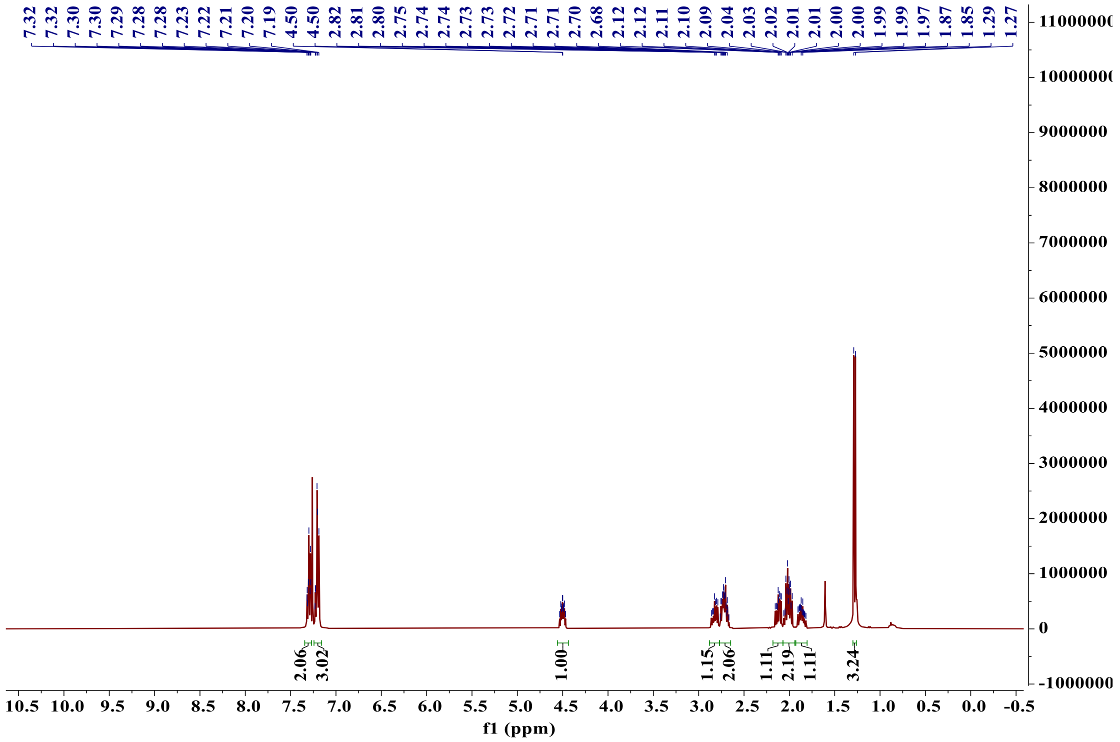

^{1}H NMR (400 MHz, $CDCl_3$) spectrum of **(*S*,*R*)-3af**

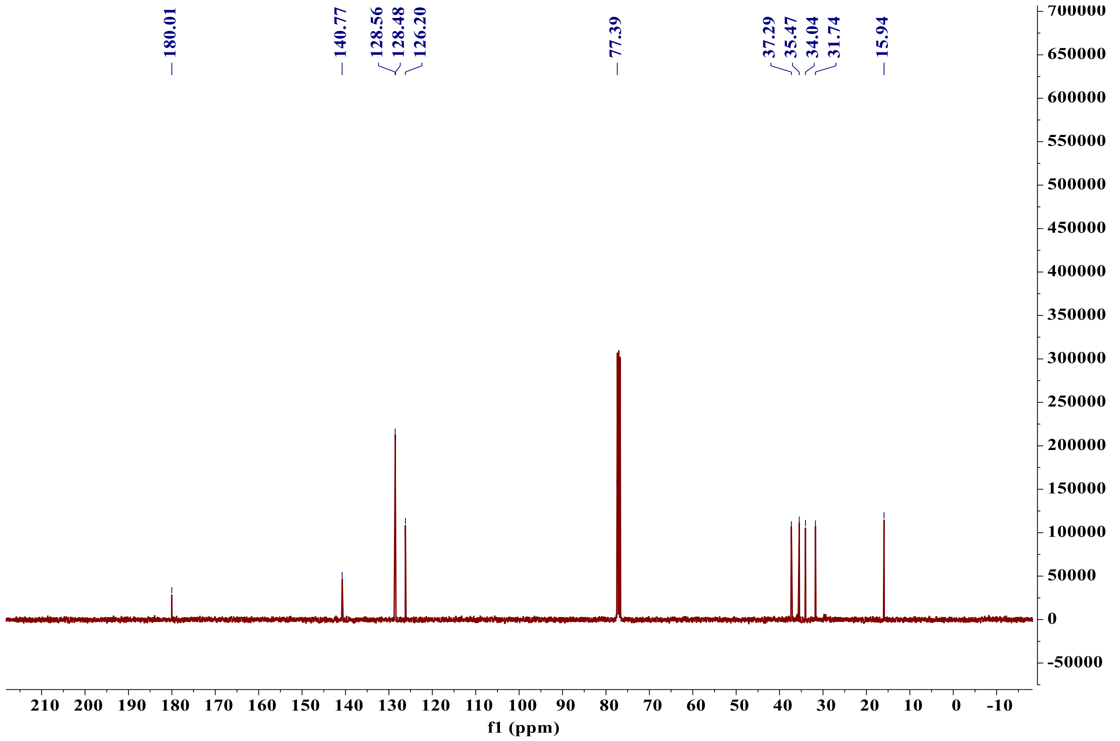

^{13}C NMR (101 MHz, $CDCl_3$) spectrum of **(*S*,*R*)-3af**

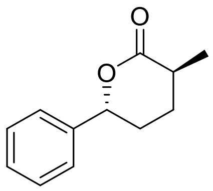

(*S*,*R*)-5a

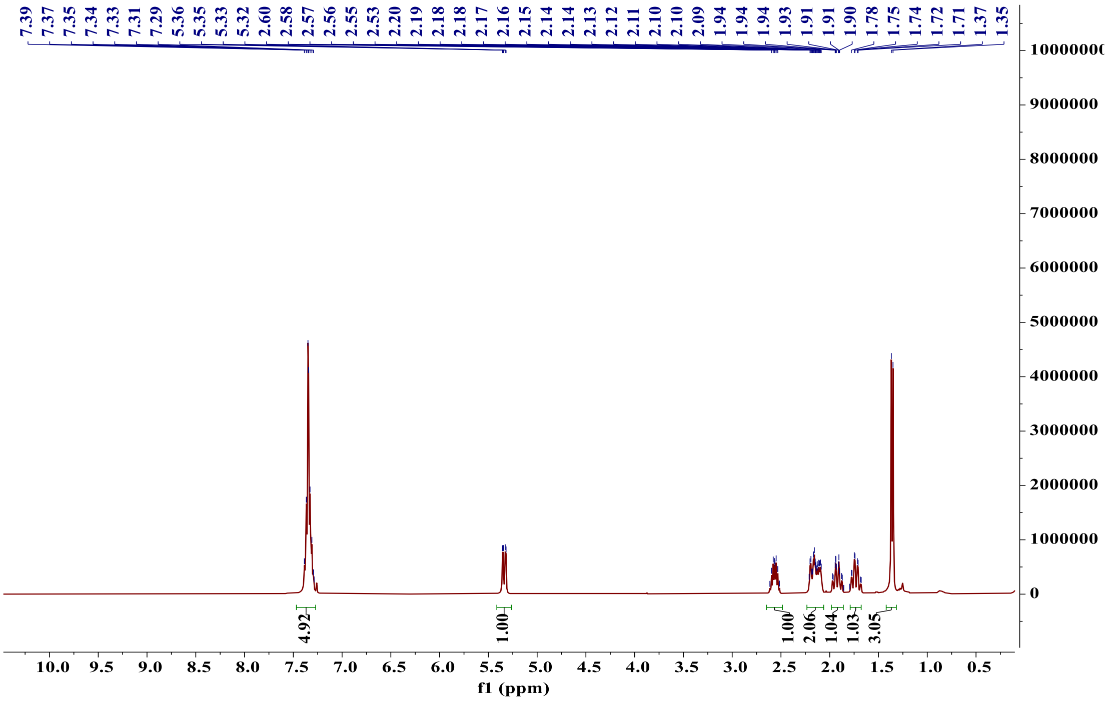

^{1}H NMR (400 MHz, $CDCl_3$) spectrum of **(*S*,*R*)-5a**

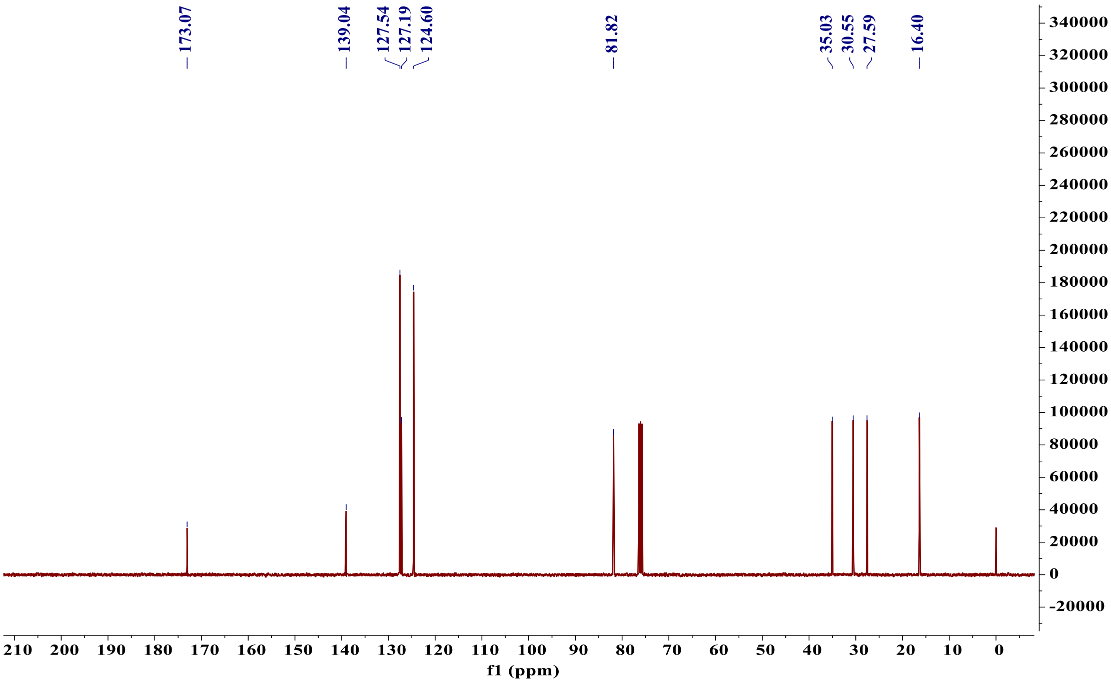

^{13}C NMR (101 MHz, $CDCl_3$) spectrum of **(*S*,*R*)-5a**

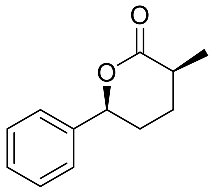

(*S*,*S*)-5a

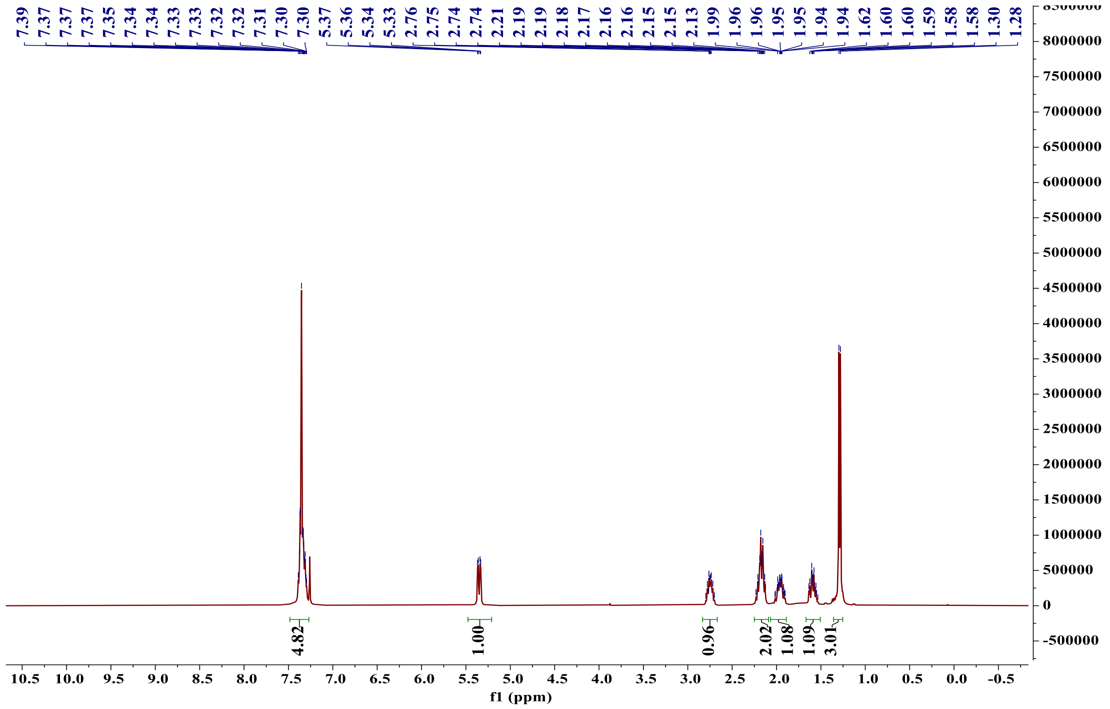

^{1}H NMR (400 MHz, $CDCl_3$) spectrum of **(*S*,*S*)-5a**

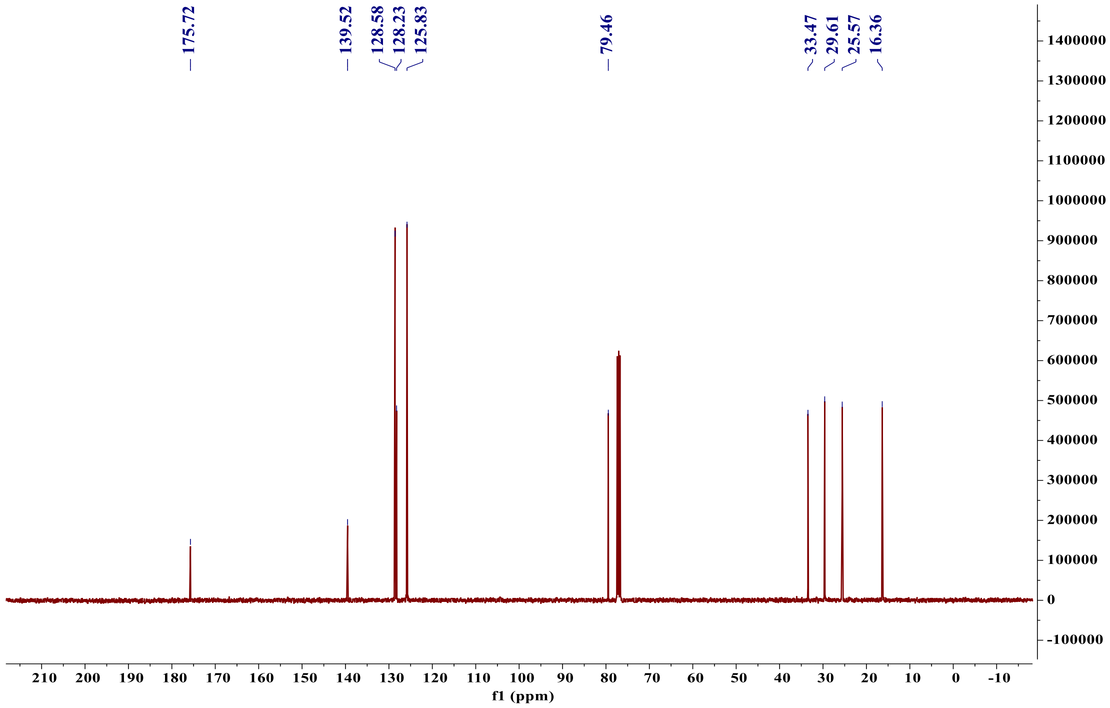

^{13}C NMR (101 MHz, $CDCl_3$) spectrum of **(*S*,*S*)-5a**

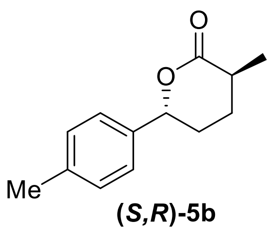

(*S*,*R*)-5b

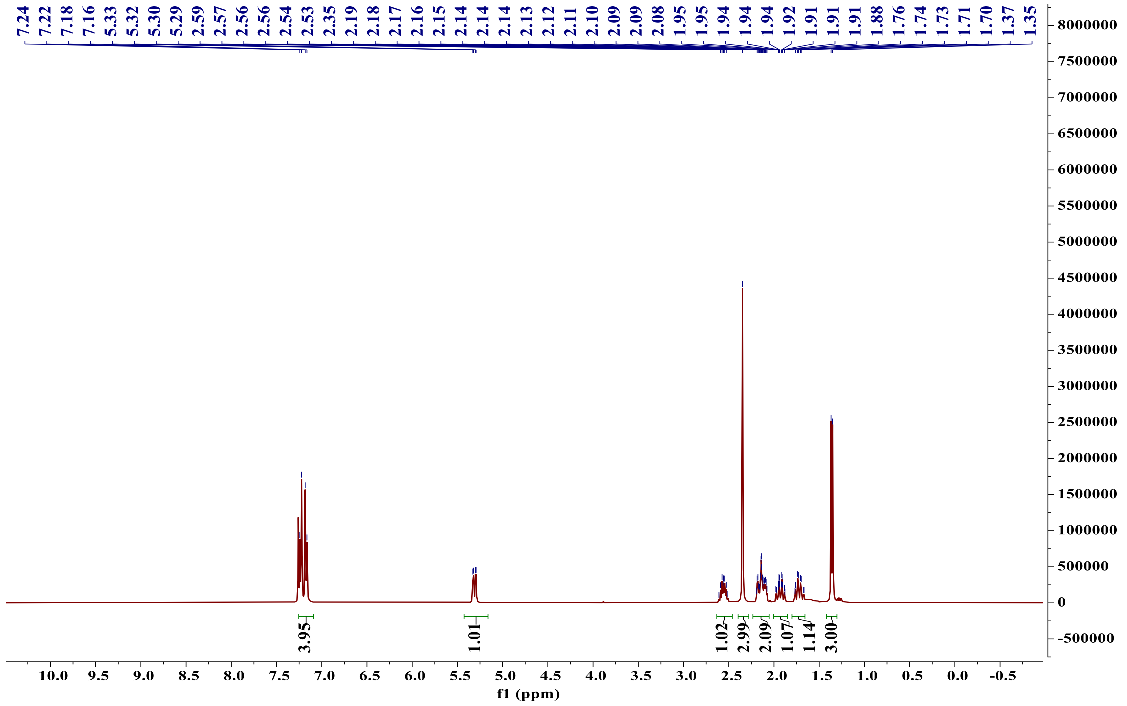

^{1}H NMR (400 MHz, $CDCl_3$) spectrum of **(*S*,*R*)-5b**

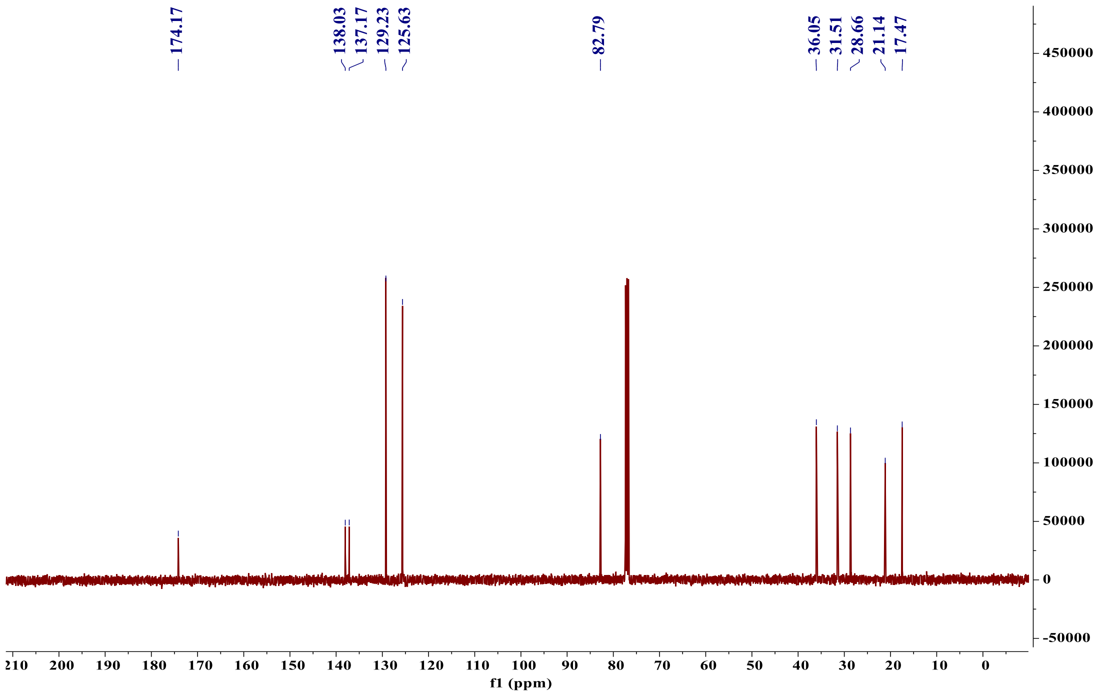

^{13}C NMR (101 MHz, $CDCl_3$) spectrum of **(*S*,*R*)-5b**

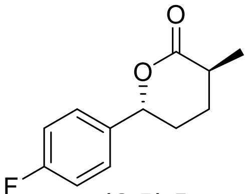

(*S*,*R*)-5c

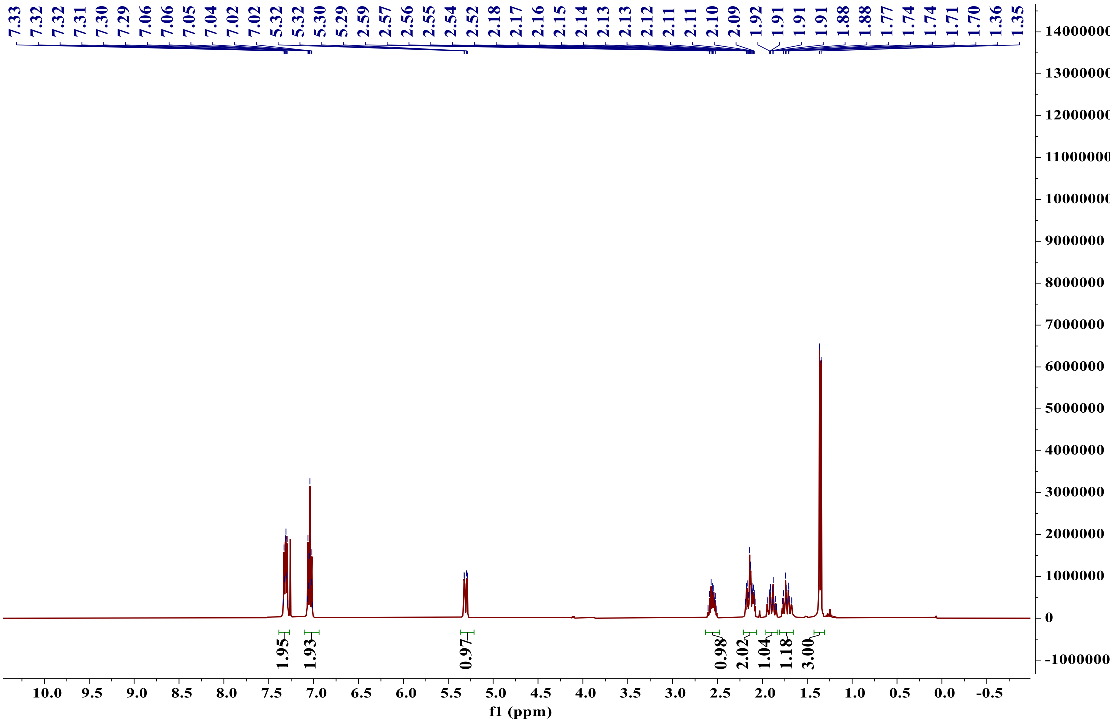

^{1}H NMR (400 MHz, $CDCl_3$) spectrum of **(*S*,*R*)-5c**

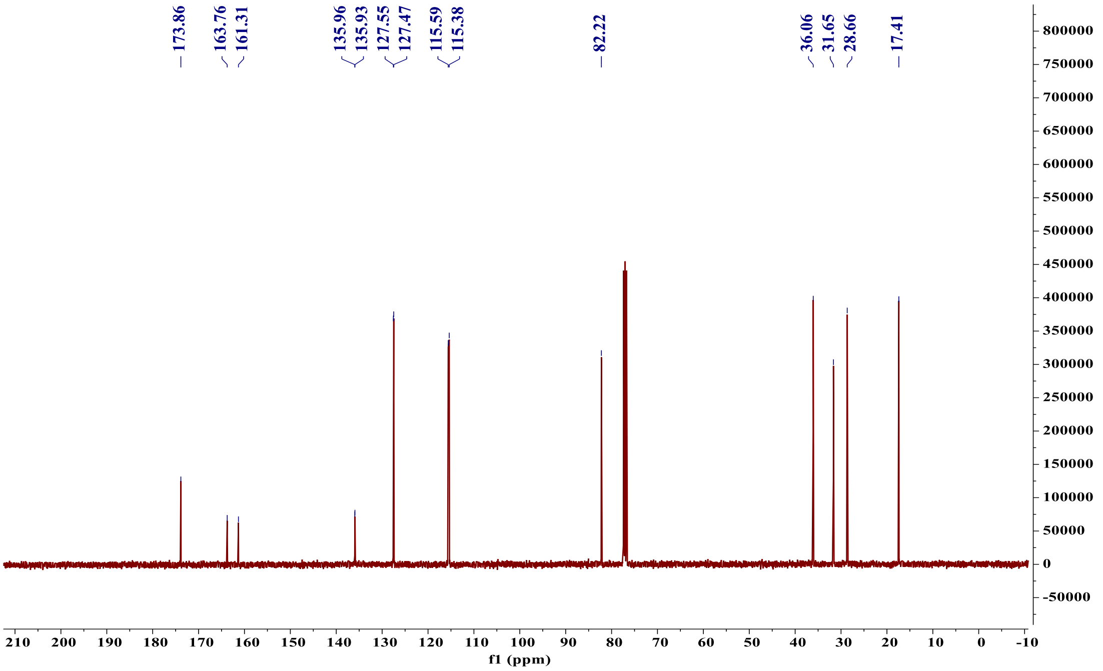

^{13}C NMR (101 MHz, $CDCl_3$) spectrum of **(*S*,*R*)-5c**

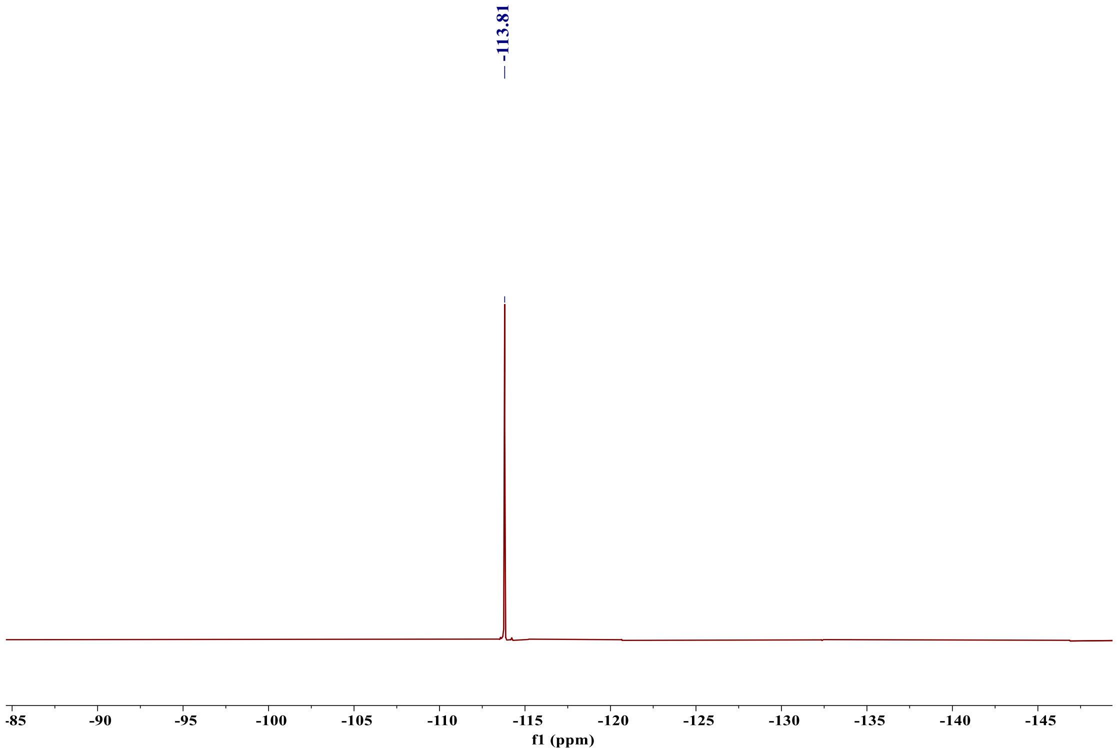

^{19}F NMR (377 MHz, $CDCl_3$) spectrum of **(*S*,*R*)-5c**

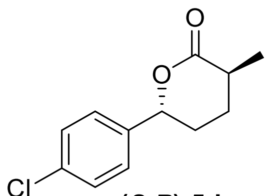

(*S*,*R*)-5d

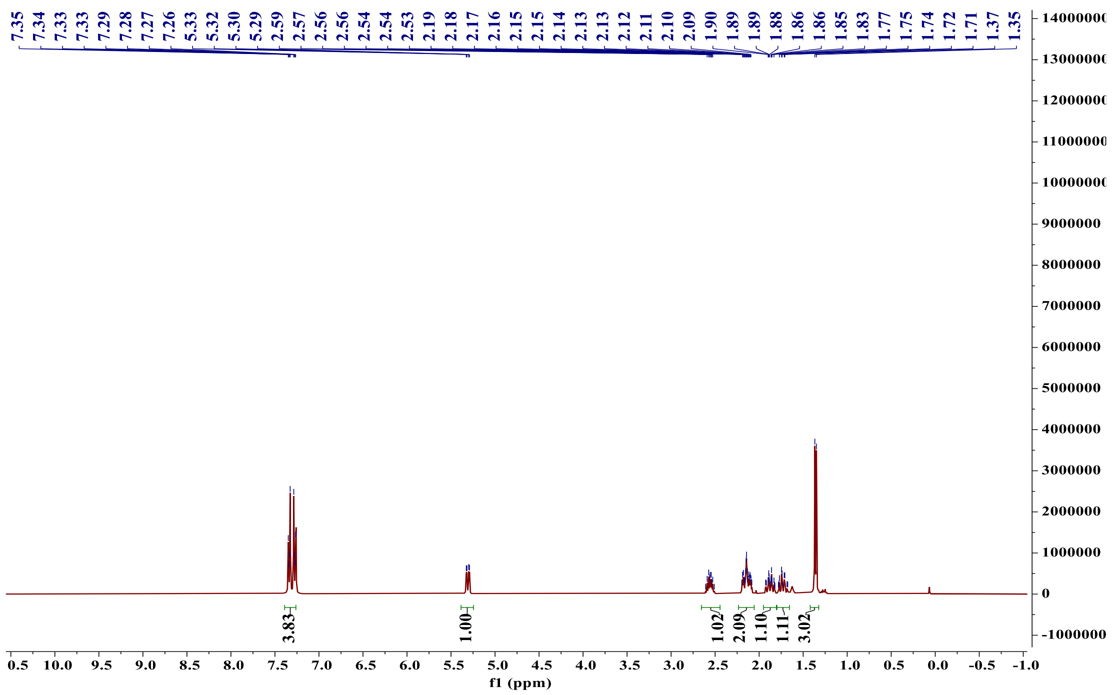

^{1}H NMR (400 MHz, $CDCl_3$) spectrum of **(*S*,*R*)-5d**

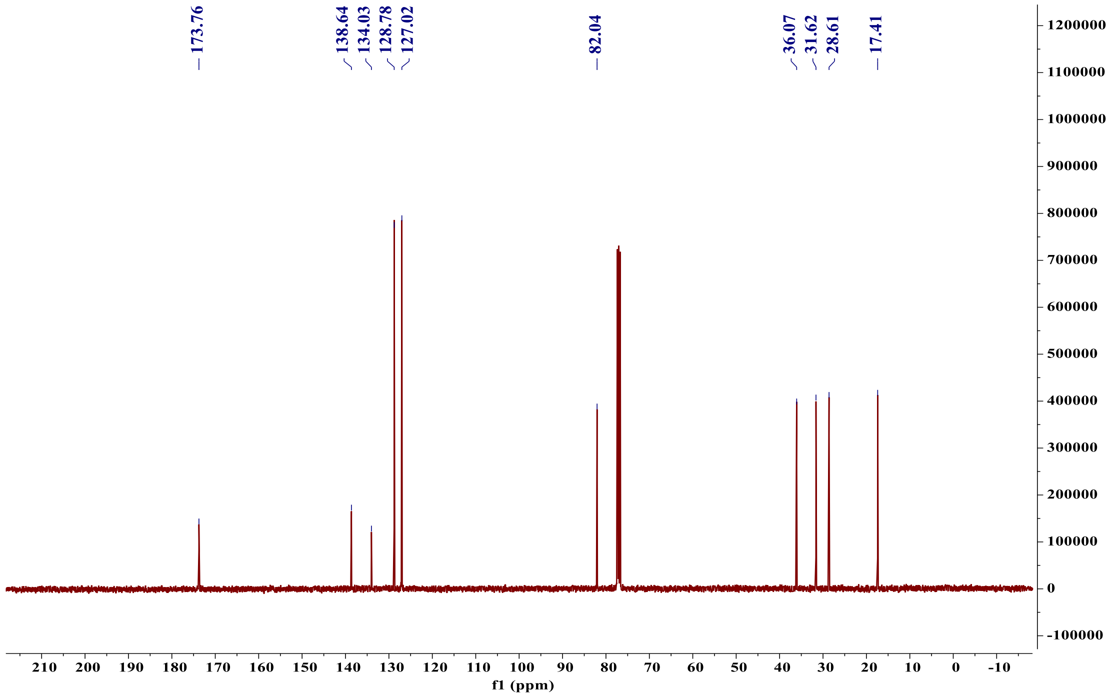

^{13}C NMR (101 MHz, $CDCl_3$) spectrum of **(*S*,*R*)-5d**

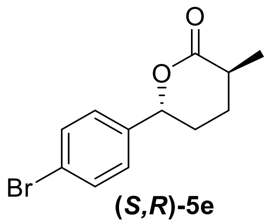

(***S***,***R***)**-5e**

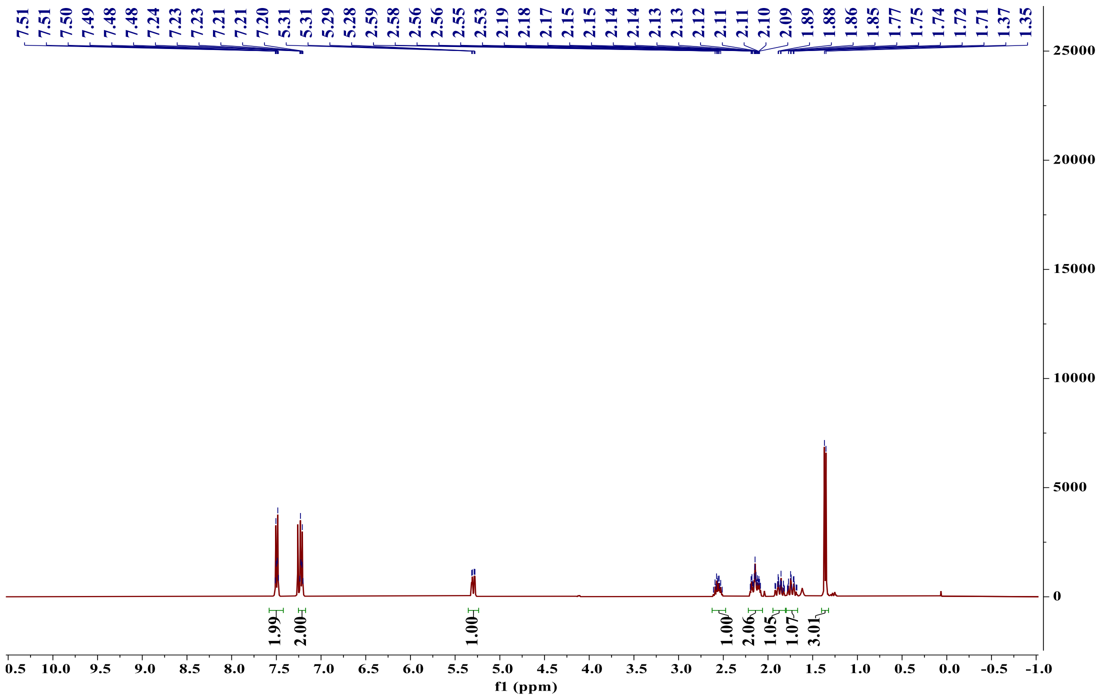

^{1}H NMR (400 MHz, $CDCl_3$) spectrum of (***S***,***R***)**-5e**

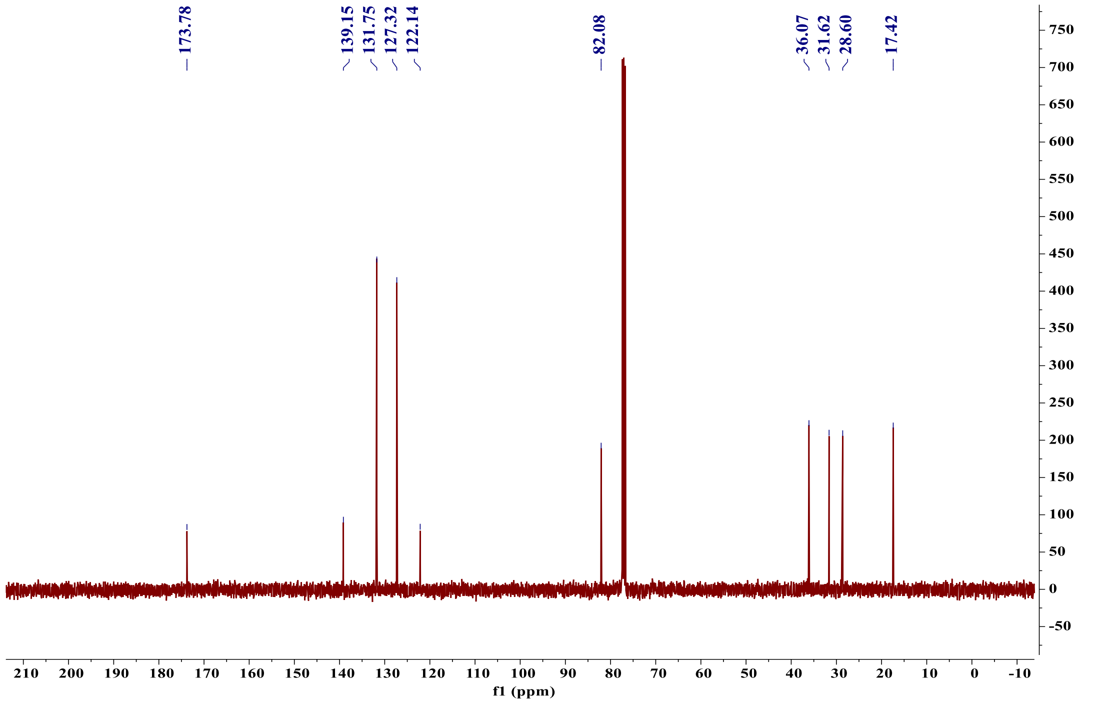

^{13}C NMR (101 MHz, $CDCl_3$) spectrum of (***S***,***R***)**-5e**

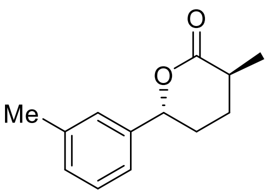

(*S*,*R*)-5f

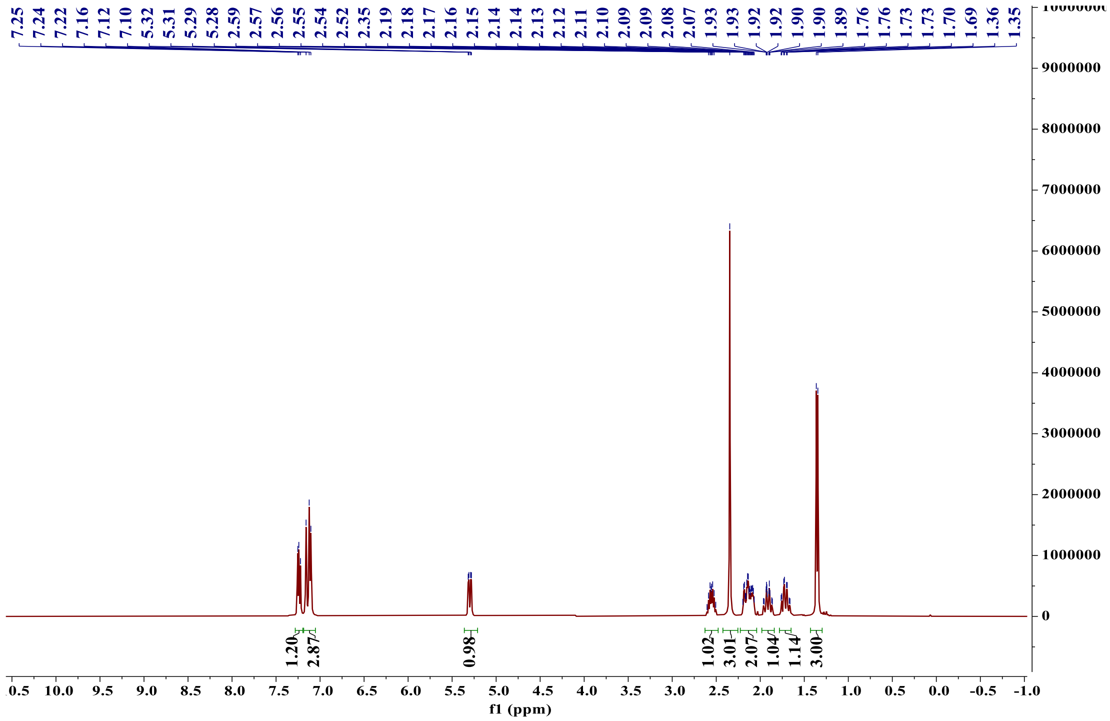

^{1}H NMR (400 MHz, $CDCl_3$) spectrum of **(*S*,*R*)-5f**

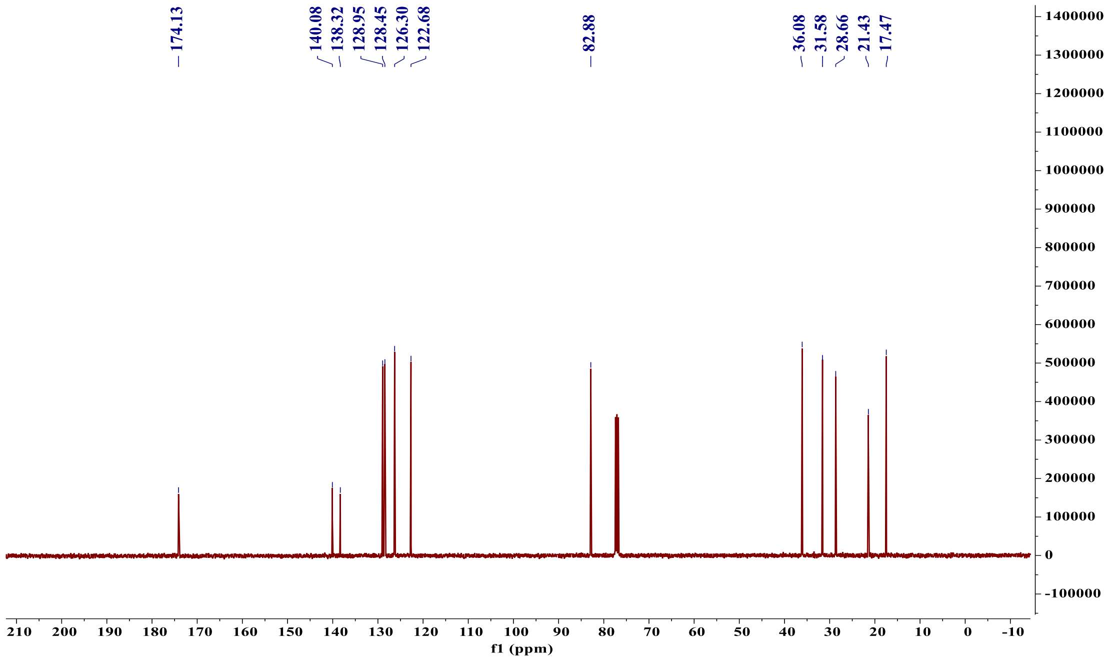

^{13}C NMR (101 MHz, $CDCl_3$) spectrum of **(*S*,*R*)-5f**

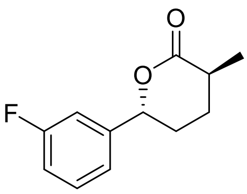

(*S*,*R*)-5g

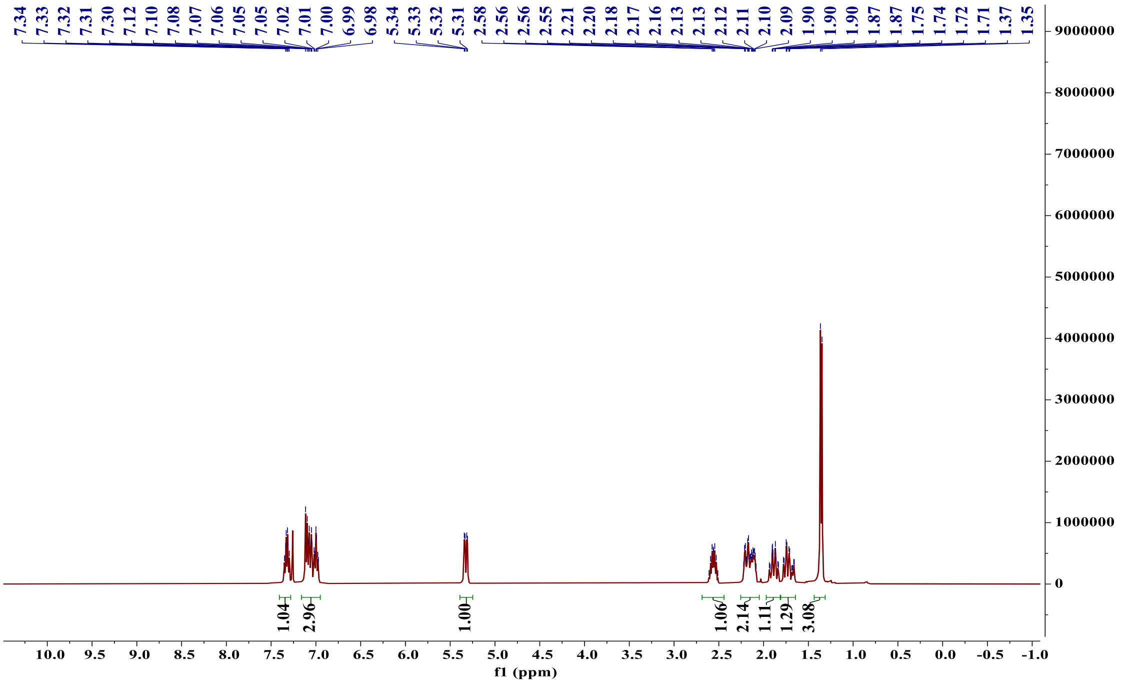

^{1}H NMR (400 MHz, $CDCl_3$) spectrum of **(*S*,*R*)-5g**

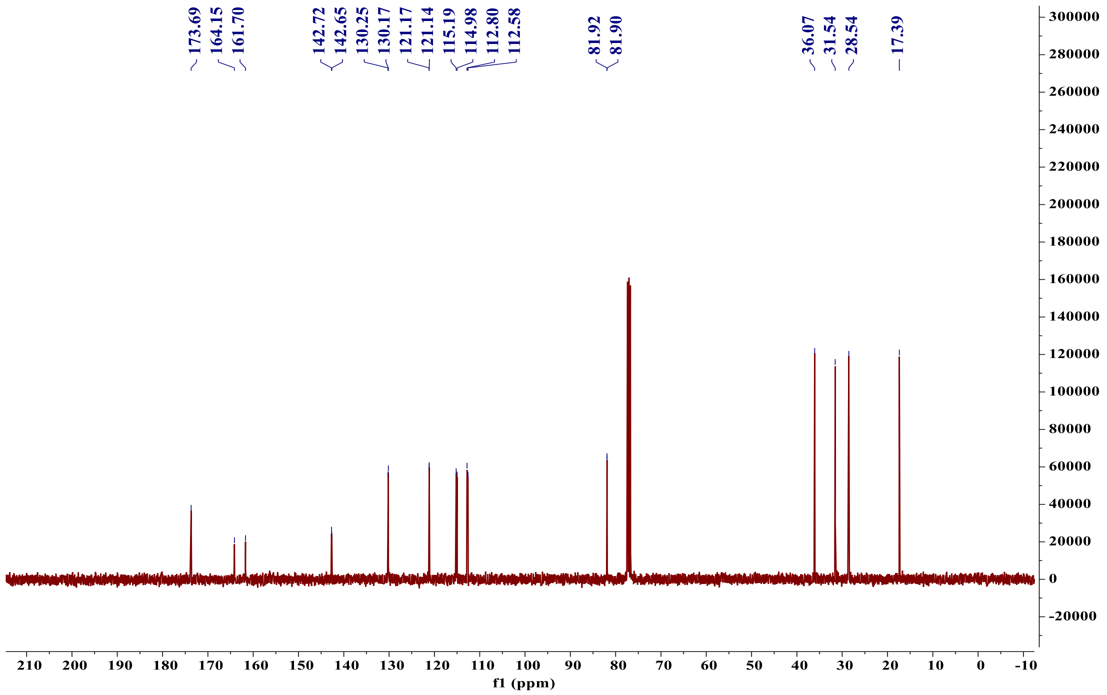

^{13}C NMR (101 MHz, $CDCl_3$) spectrum of **(*S*,*R*)-5g**

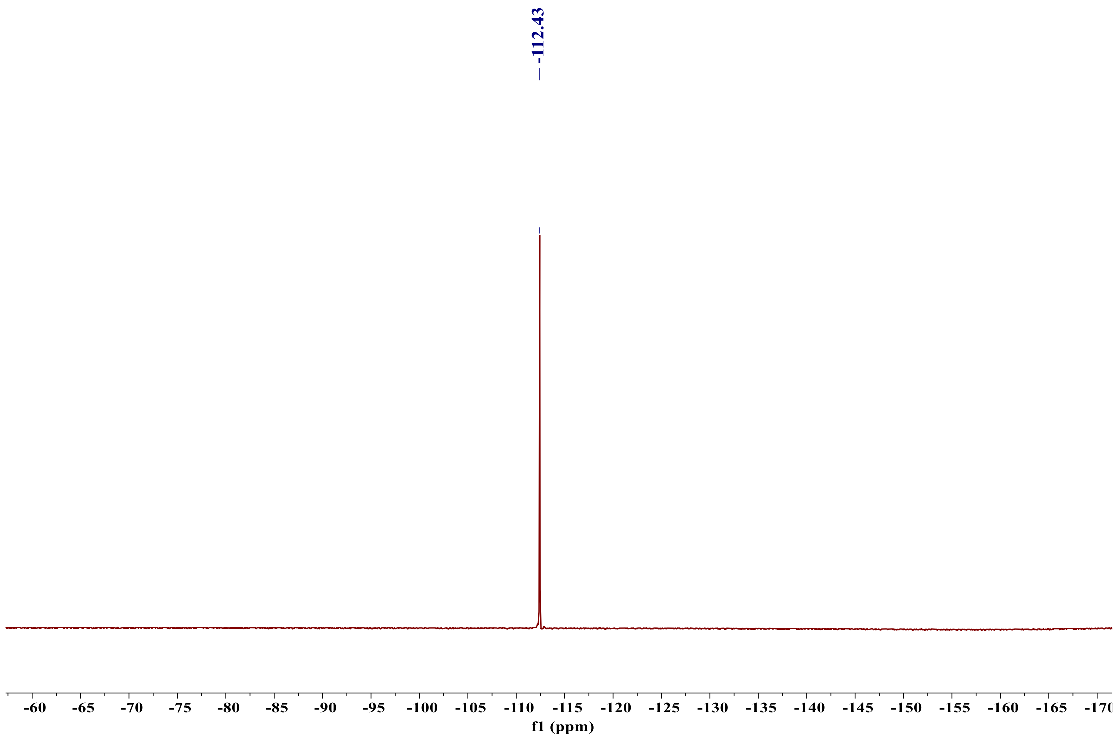

^{19}F NMR (377 MHz, $CDCl_3$) spectrum of **(*S*,*R*)-5g**

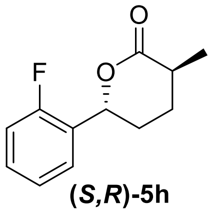

(*S*,*R*)-5h

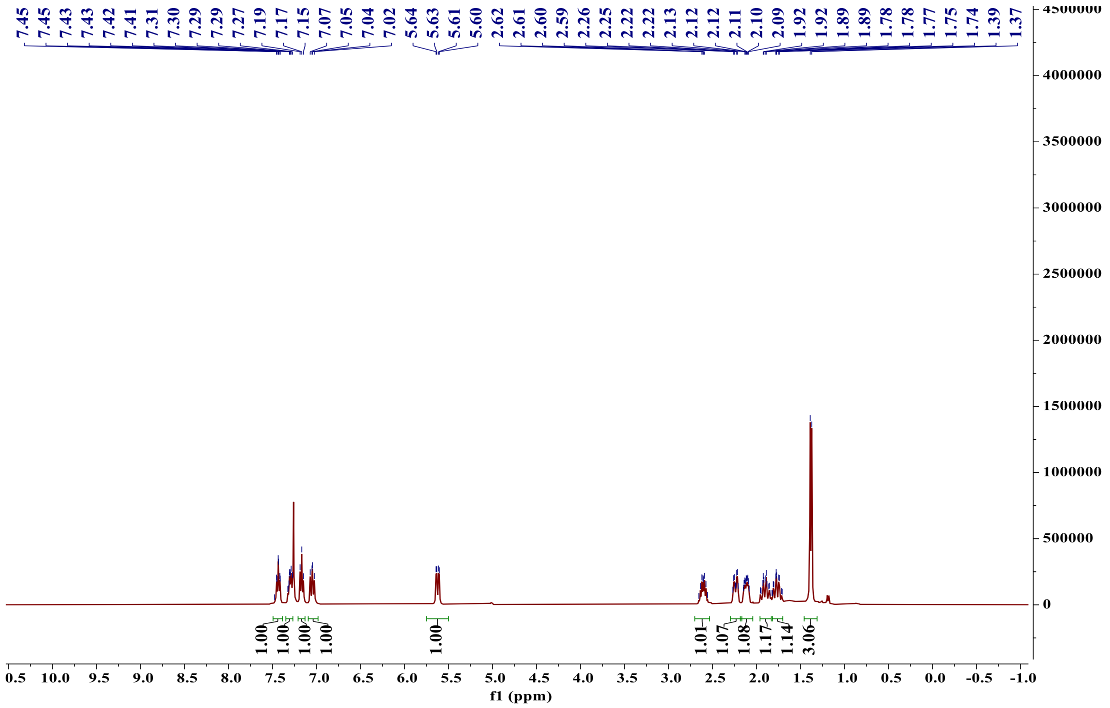

^{1}H NMR (400 MHz, $CDCl_3$) spectrum of **(*S*,*R*)-5h**

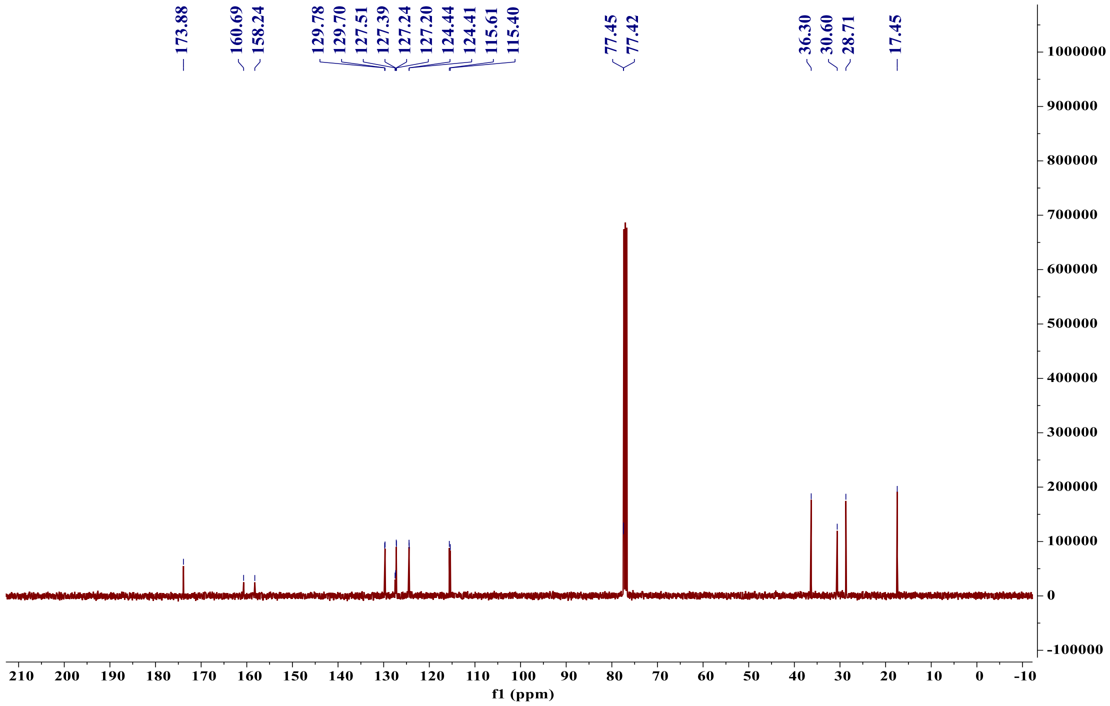

^{13}C NMR (101 MHz, $CDCl_3$) spectrum of **(*S*,*R*)-5h**

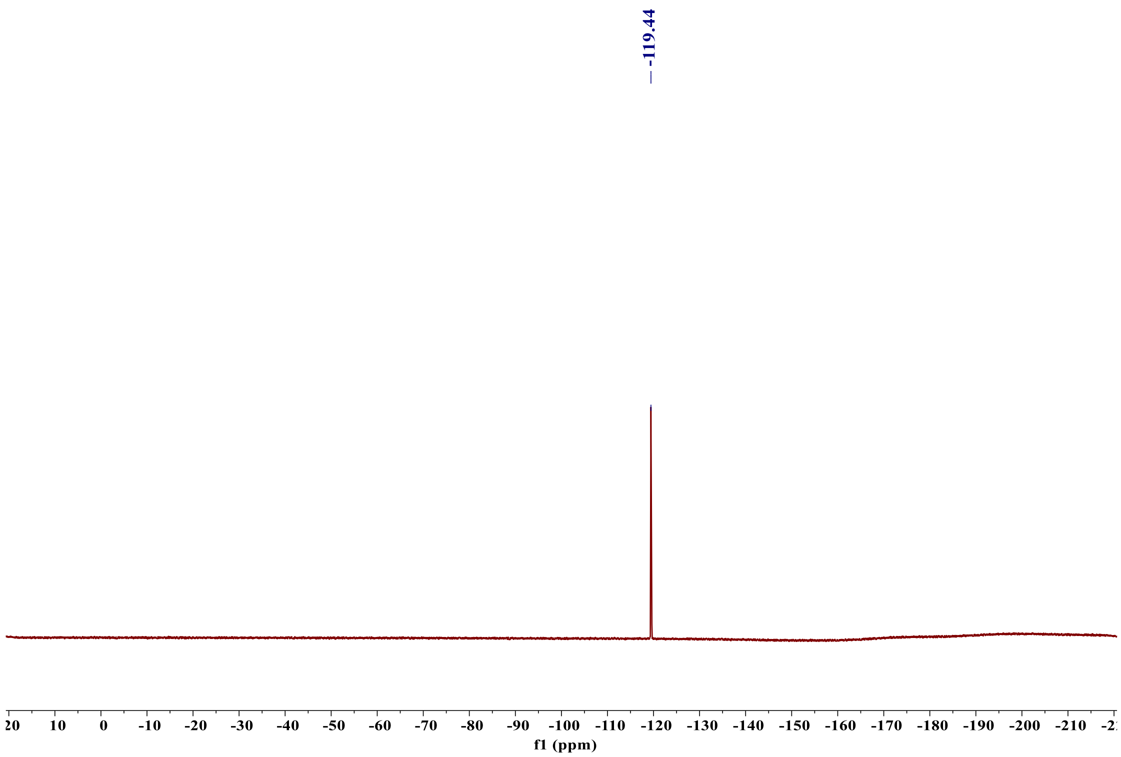

^{19}F NMR (377 MHz, $CDCl_3$) spectrum of **(*S*,*R*)-5h**

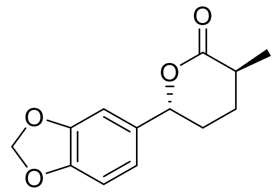

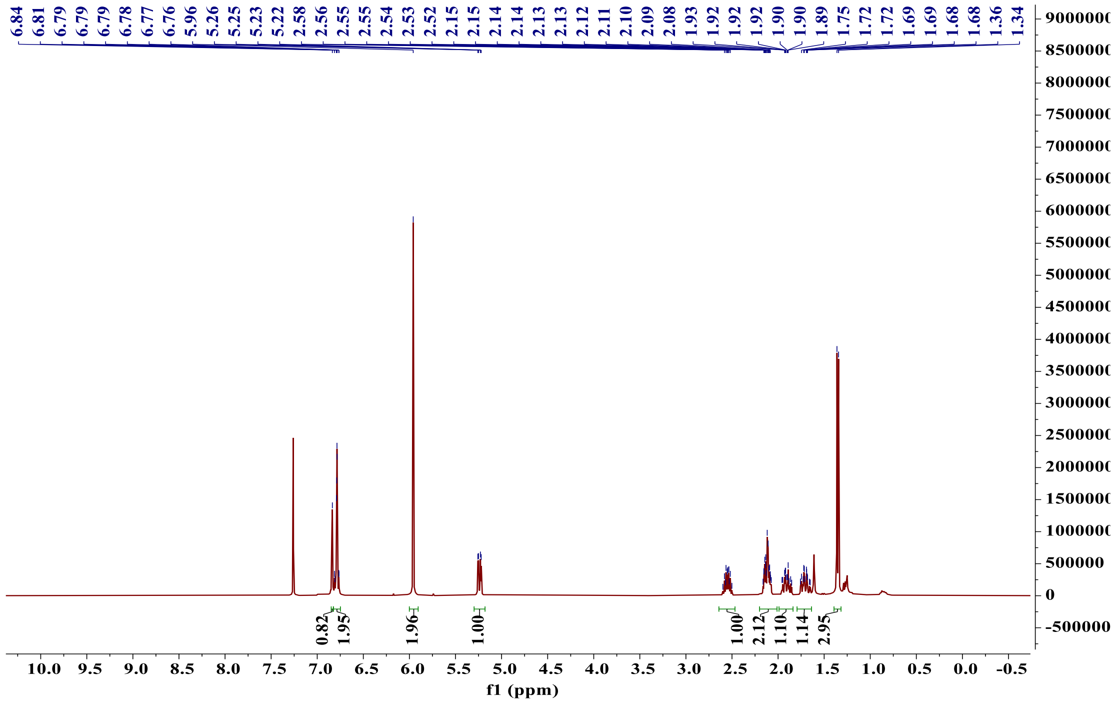

^{1}H NMR (400 MHz, CDCl$_3$) spectrum of ***(S,R)*-5i**

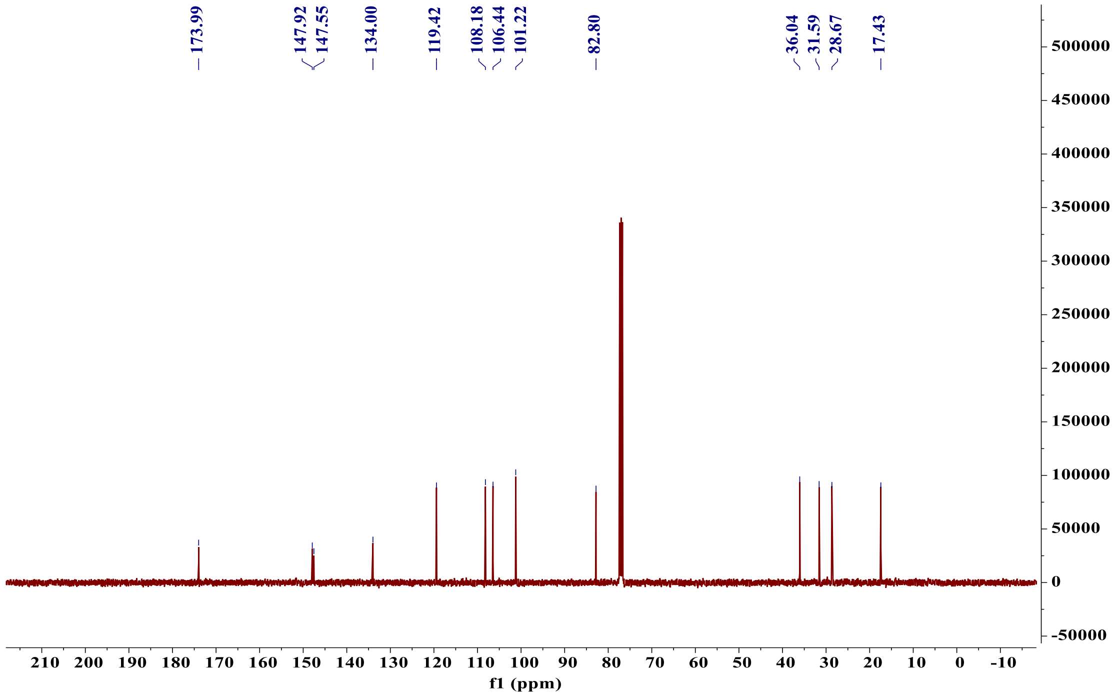

^{13}C NMR (101 MHz, CDCl$_3$) spectrum of ***(S,R)*-5i**

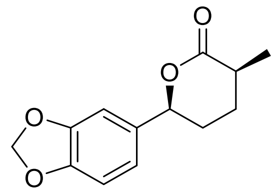

(*S*,*S*)-5i

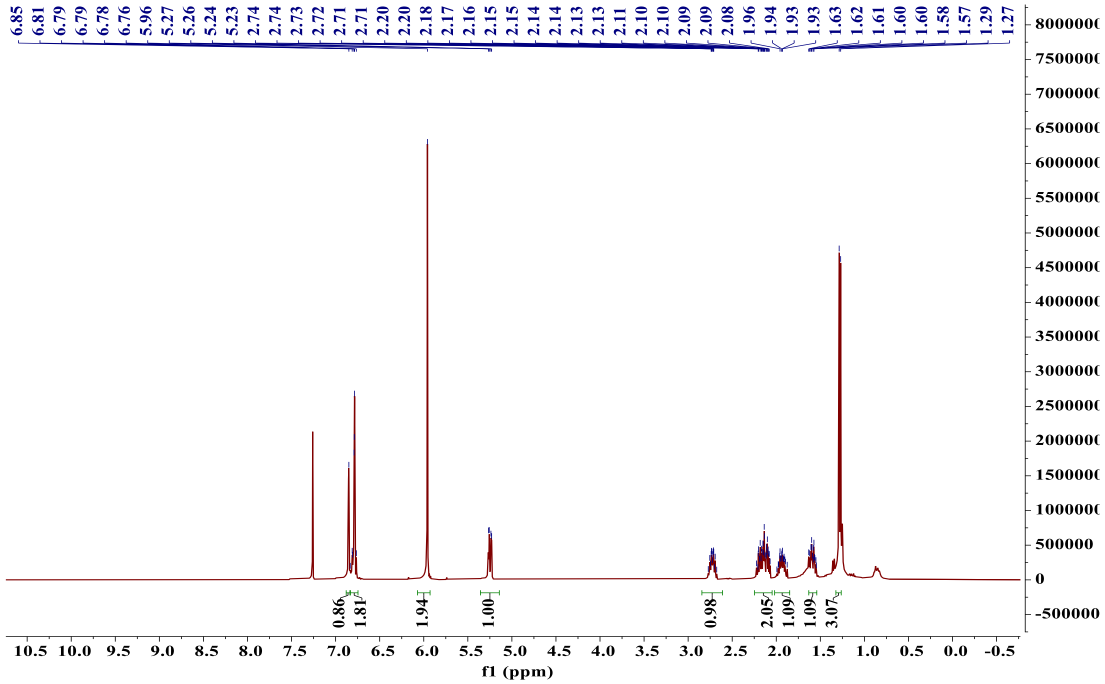

^{1}H NMR (400 MHz, $CDCl_3$) spectrum of **(*S*,*S*)-5i**

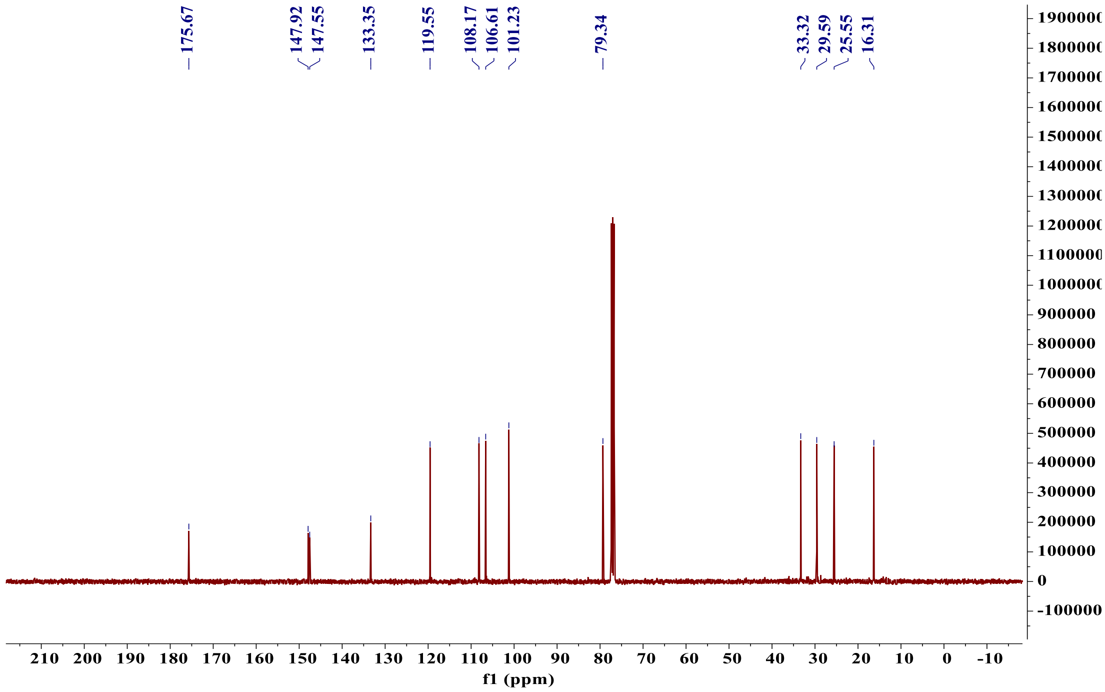

^{13}C NMR (101 MHz, $CDCl_3$) spectrum of **(*S*,*S*)-5i**

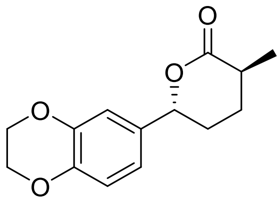

(*S*,*R*)-5j

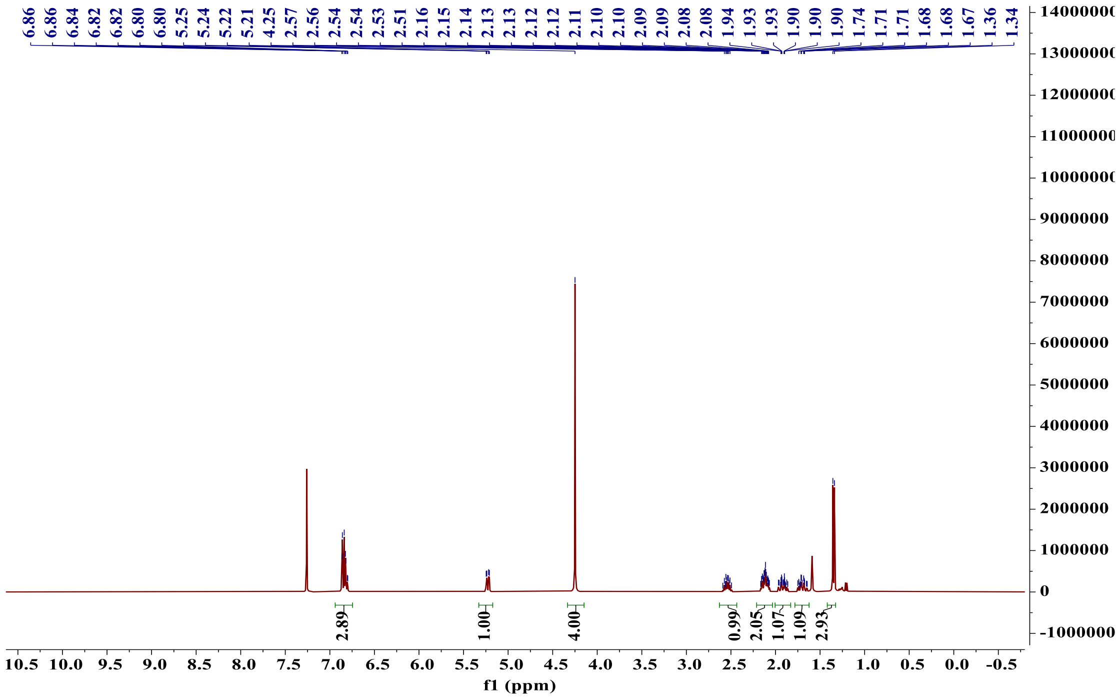

^{1}H NMR (400 MHz, $CDCl_3$) spectrum of **(*S*,*R*)-5j**

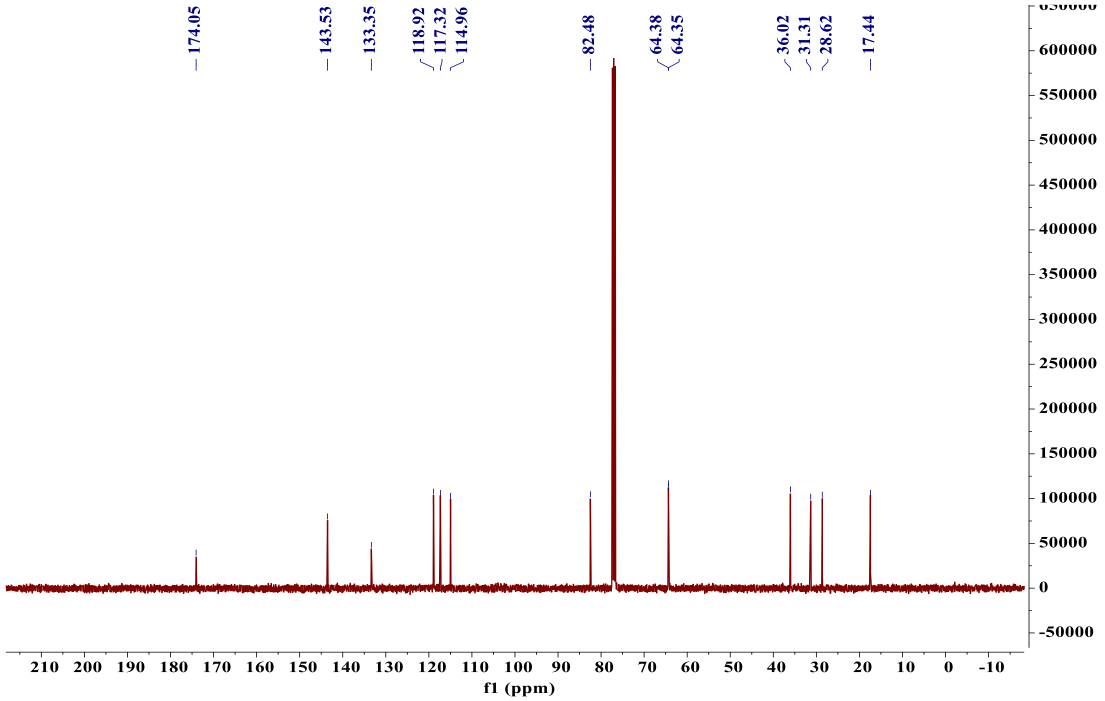

^{13}C NMR (101 MHz, $CDCl_3$) spectrum of **(*S*,*R*)-5j**

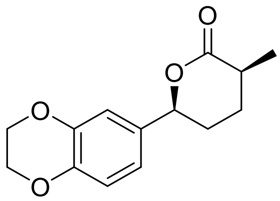
(*S*,*S*)-5j

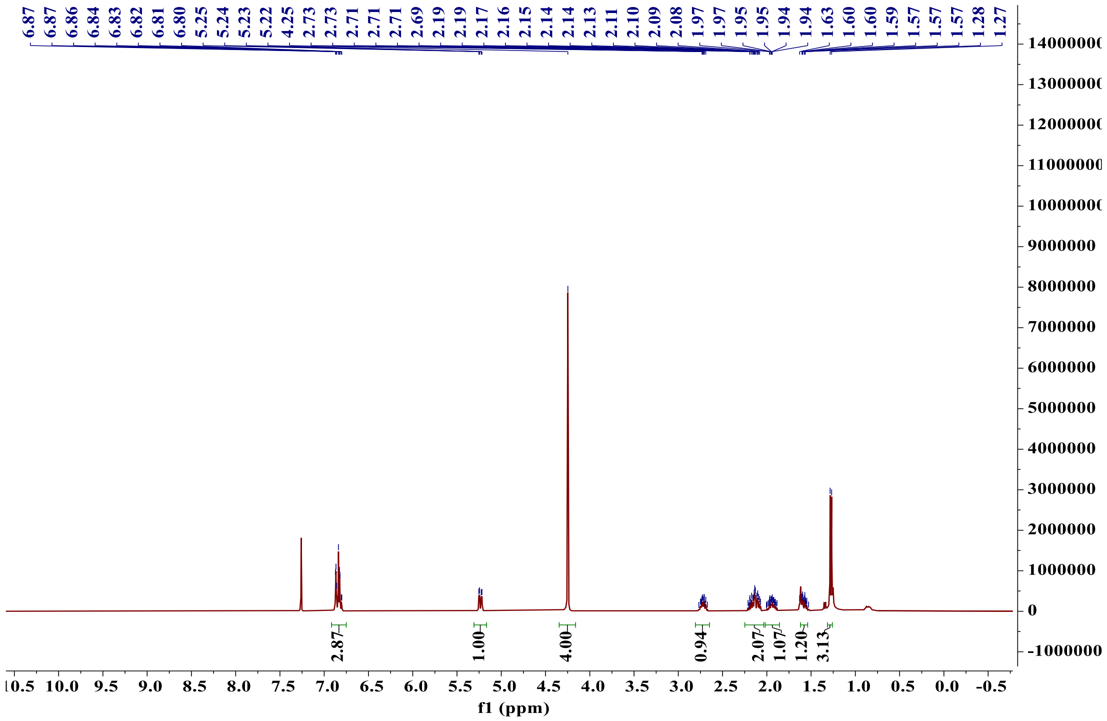

^{1}H NMR (400 MHz, $CDCl_3$) spectrum of **(*S*,*S*)-5j**

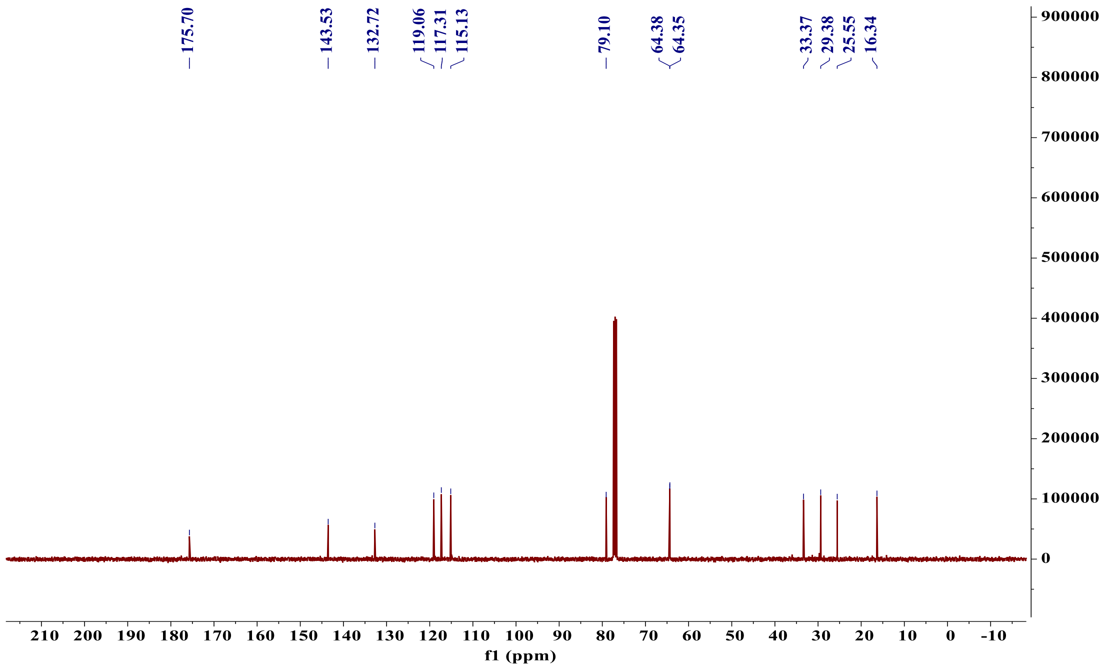

^{13}C NMR (101 MHz, $CDCl_3$) spectrum of **(*S*,*S*)-5j**

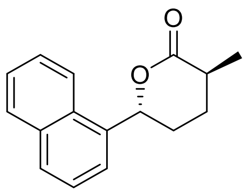

(*S*,*R*)-5k

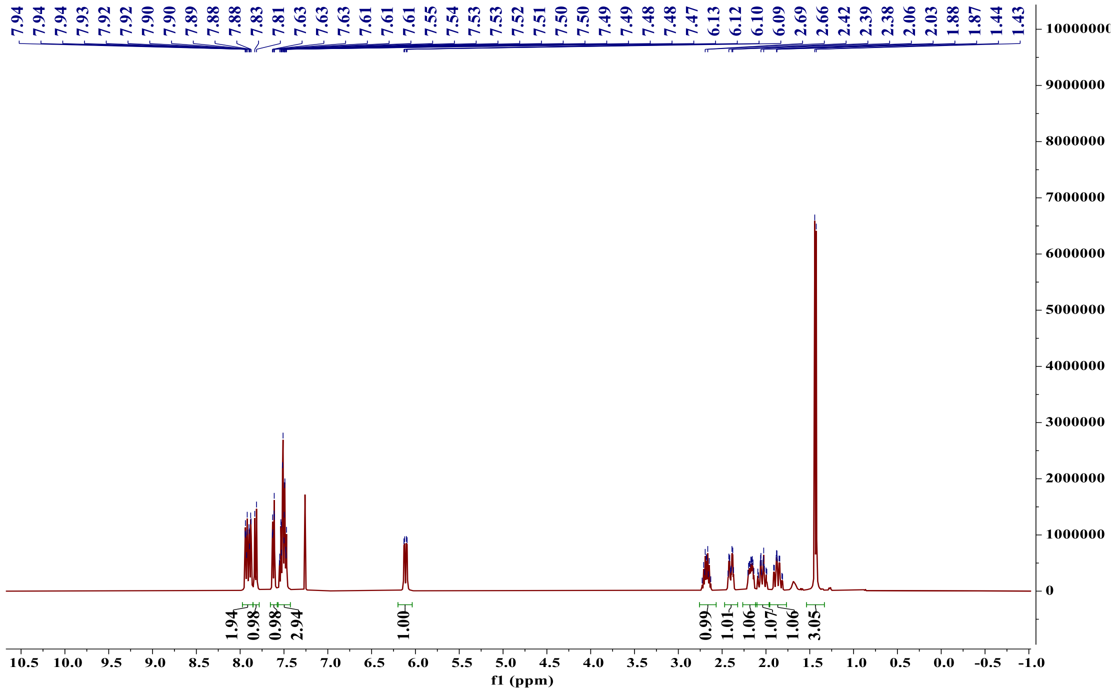

^{1}H NMR (400 MHz, $CDCl_3$) spectrum of **(*S*,*R*)-5k**

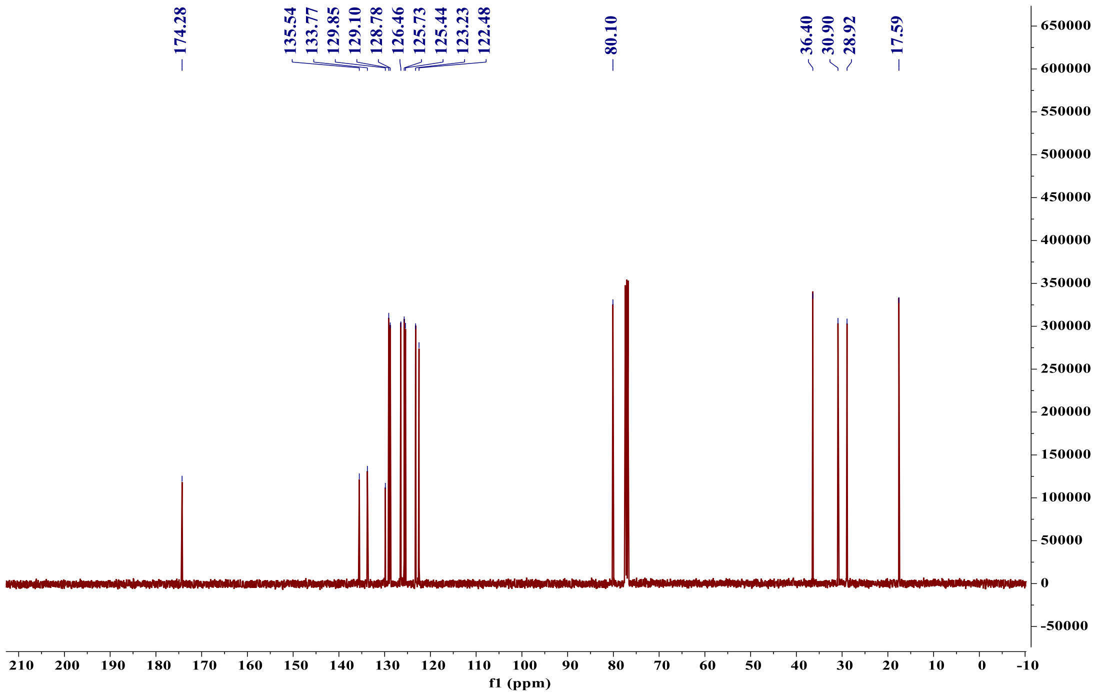

^{13}C NMR (101 MHz, $CDCl_3$) spectrum of **(*S*,*R*)-5k**

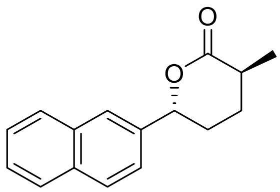

(*S*,*R*)-5l

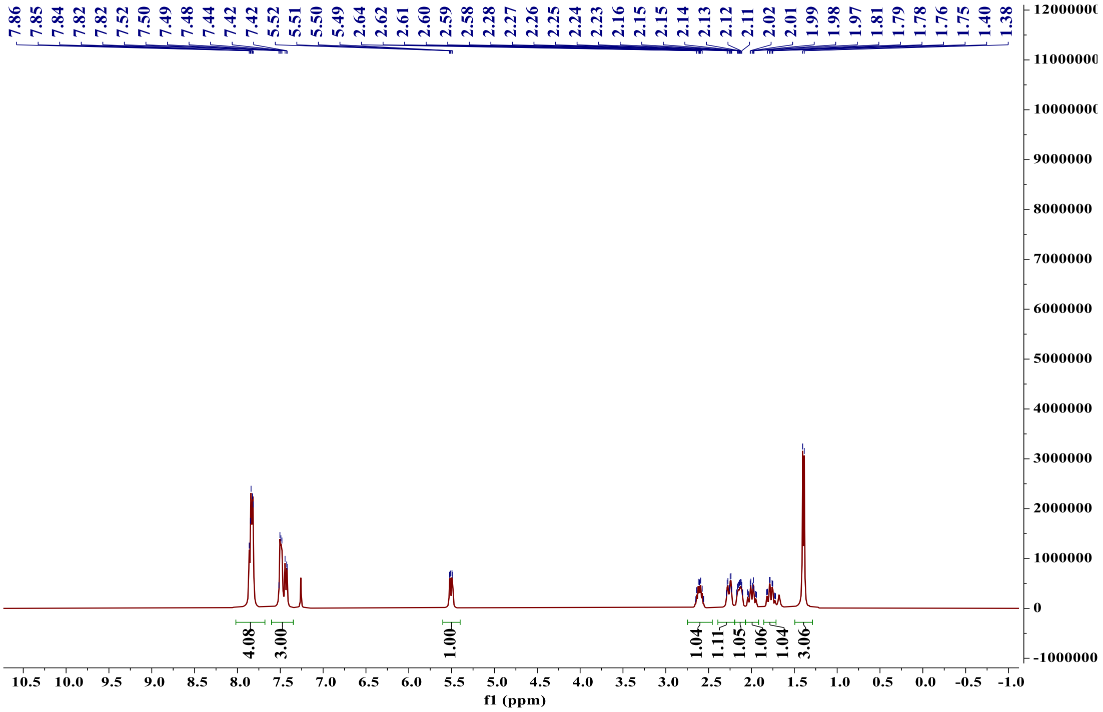

^{1}H NMR (400 MHz, CDCl$_3$) spectrum of **(*S*,*R*)-5l**

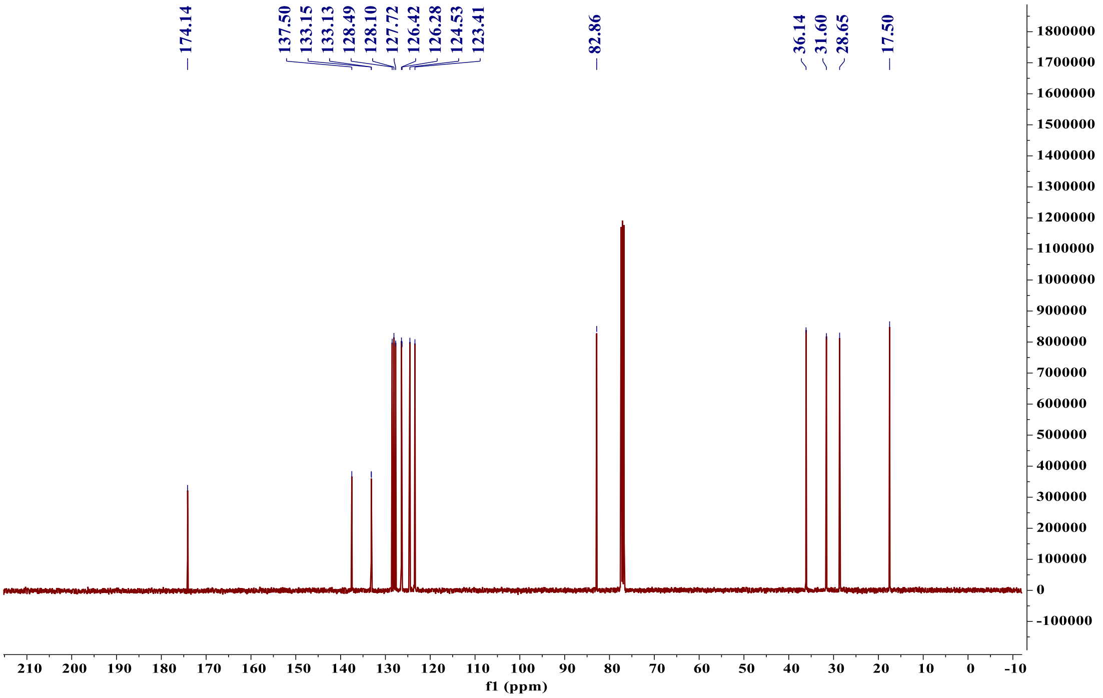

^{13}C NMR (101 MHz, CDCl$_3$) spectrum of **(*S*,*R*)-5l**

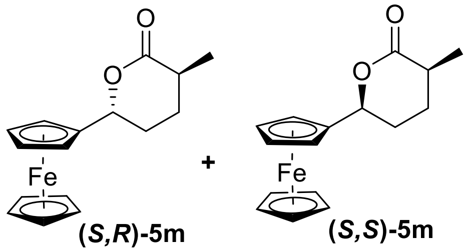

(*S*,*R*)-5m + (*S*,*S*)-5m

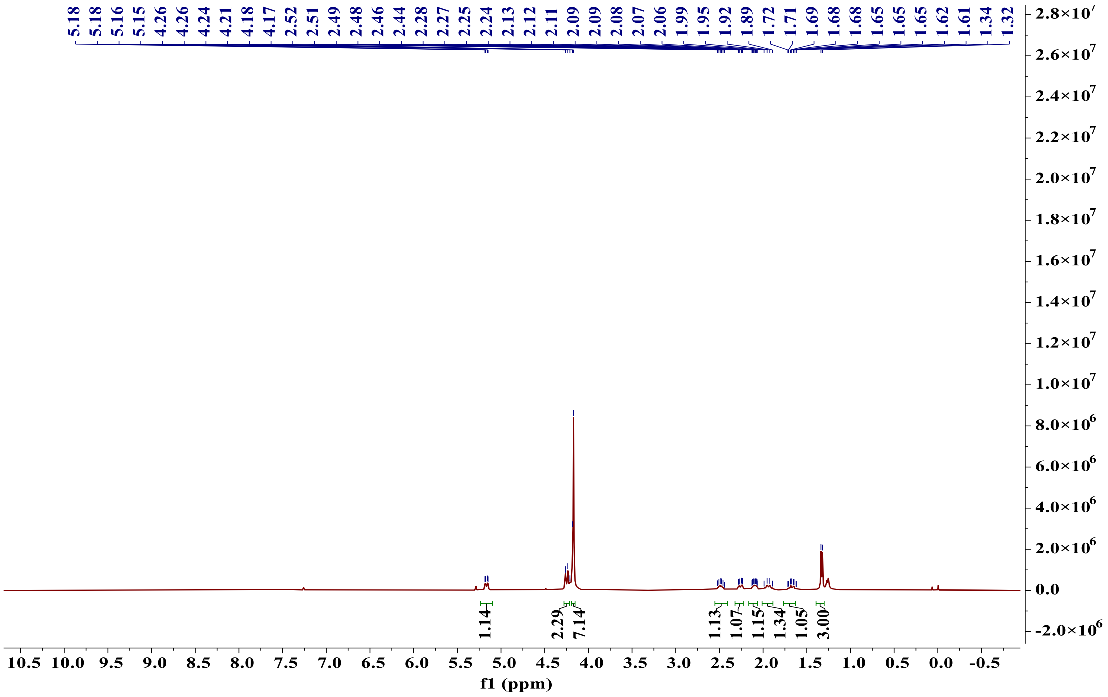

^{1}H NMR (400 MHz, $CDCl_3$) spectrum of **(*S*,*R*)-5m** and **(*S*,*S*)-5m**

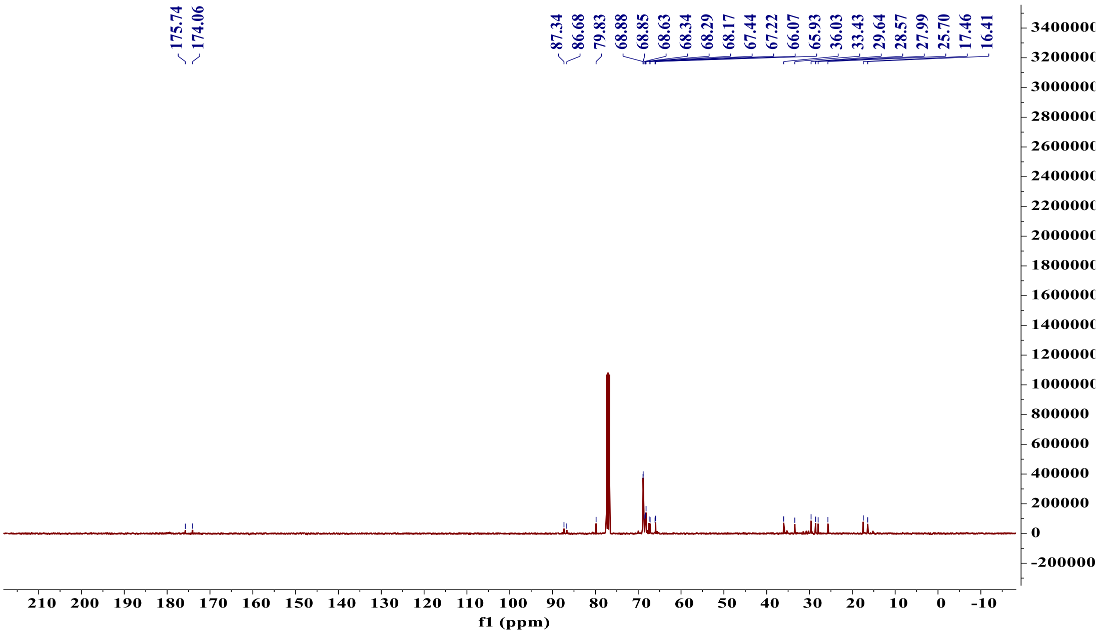

^{13}C NMR (101 MHz, $CDCl_3$) spectrum of **(*S*,*R*)-5m** and **(*S*,*S*)-5m**

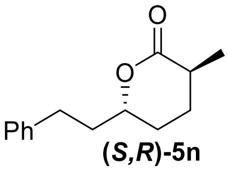

(*S*,*R*)-5n

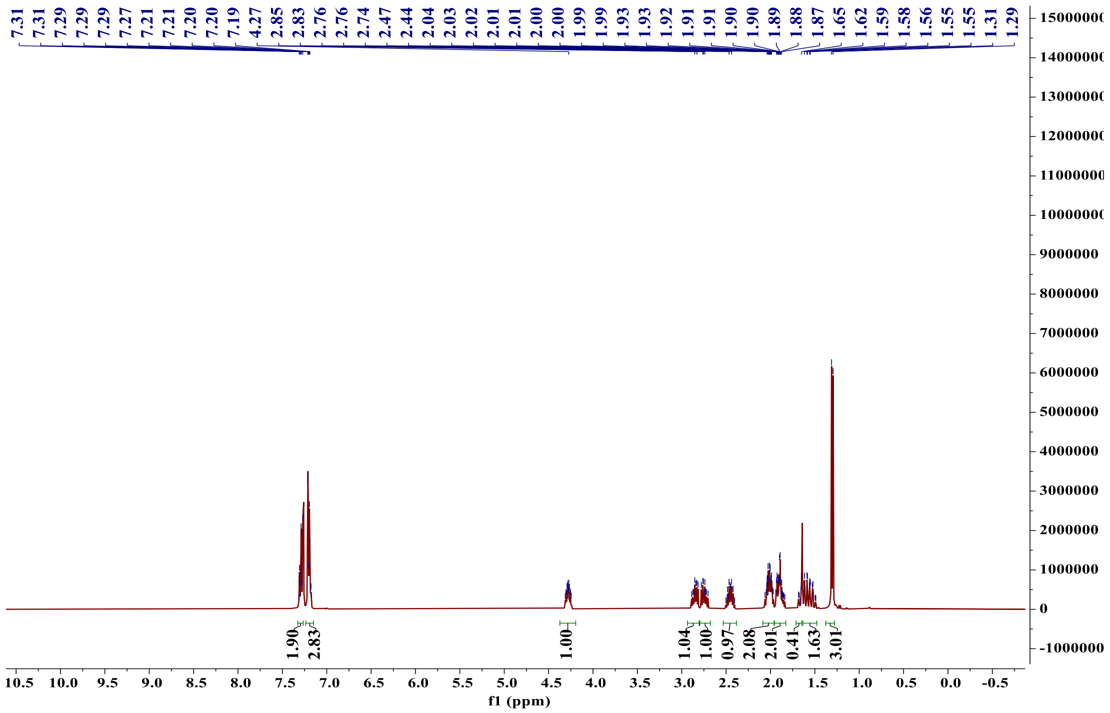

^{1}H NMR (400 MHz, $CDCl_3$) spectrum of **(*S*,*R*)-5n**

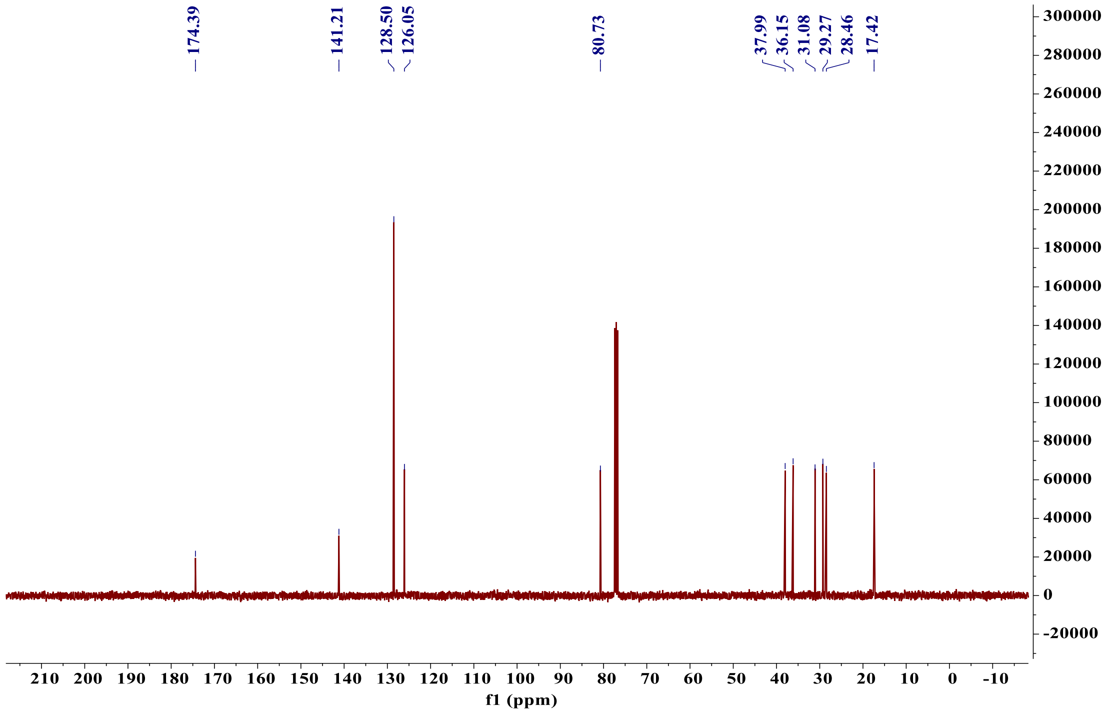

^{13}C NMR (101 MHz, $CDCl_3$) spectrum of **(*S*,*R*)-5n**

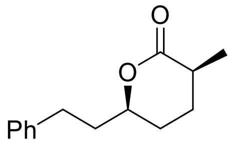

(*S*,*S*)-5n

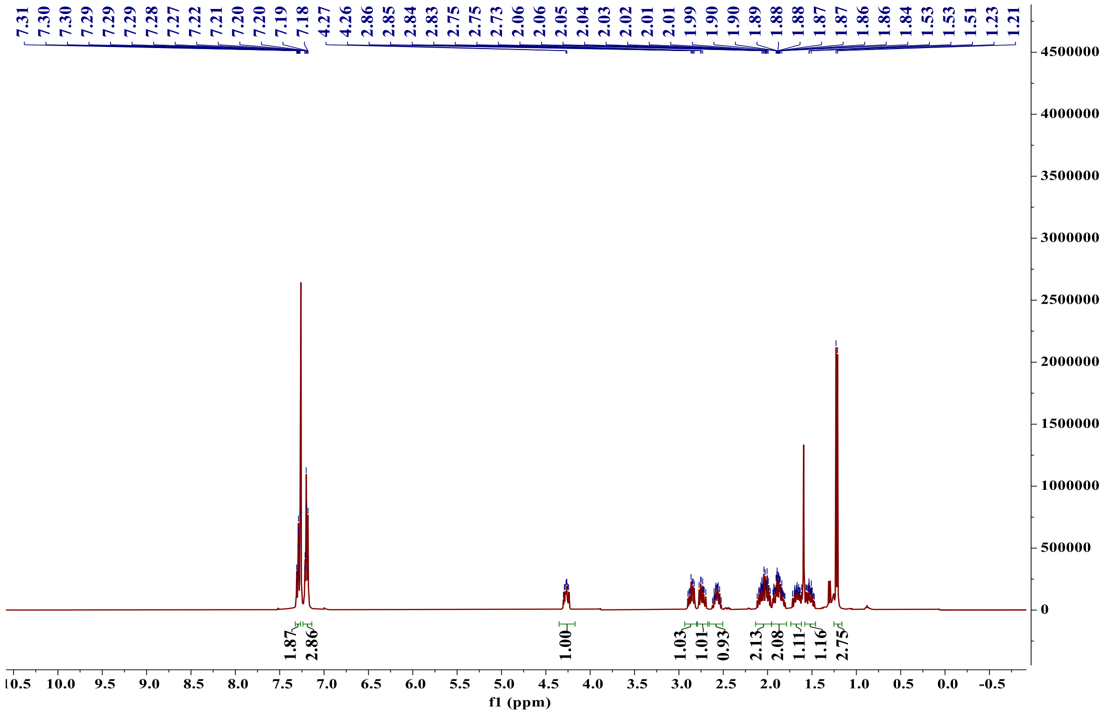

^{1}H NMR (400 MHz, $CDCl_3$) spectrum of **(*S*,*S*)-5n**

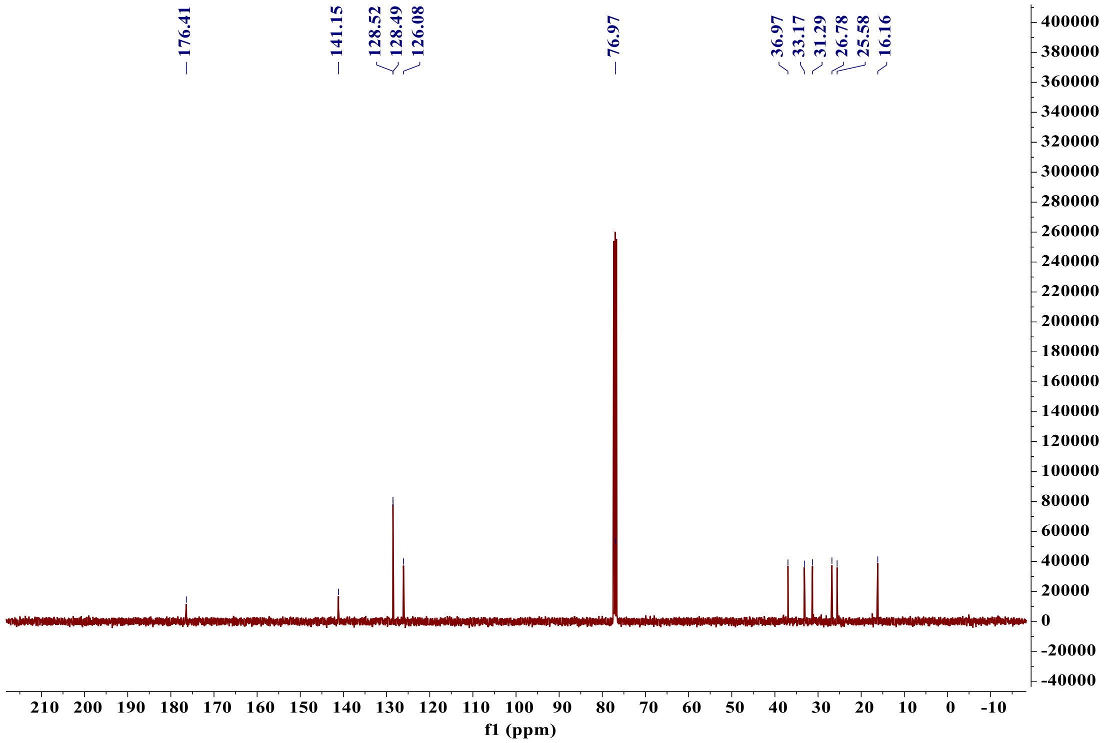

^{13}C NMR (101 MHz, $CDCl_3$) spectrum of **(*S*,*S*)-5n**

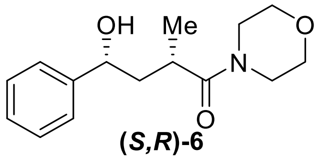

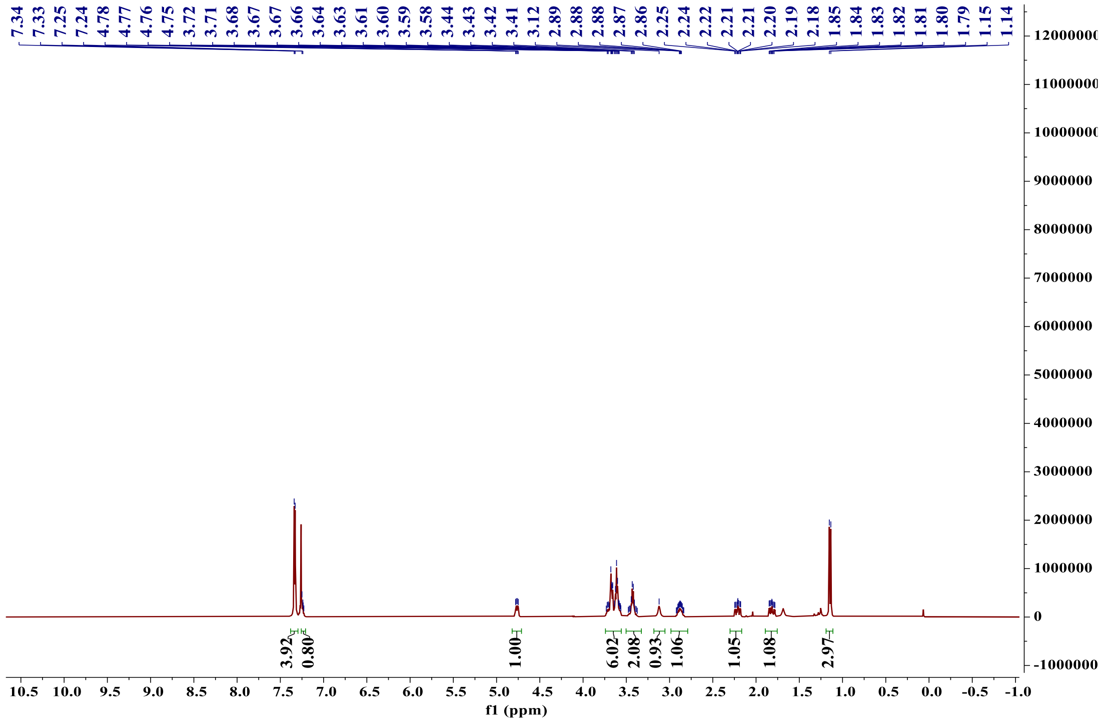

^{1}H NMR (400 MHz, $CDCl_3$) spectrum of ***(S,R)*-6**

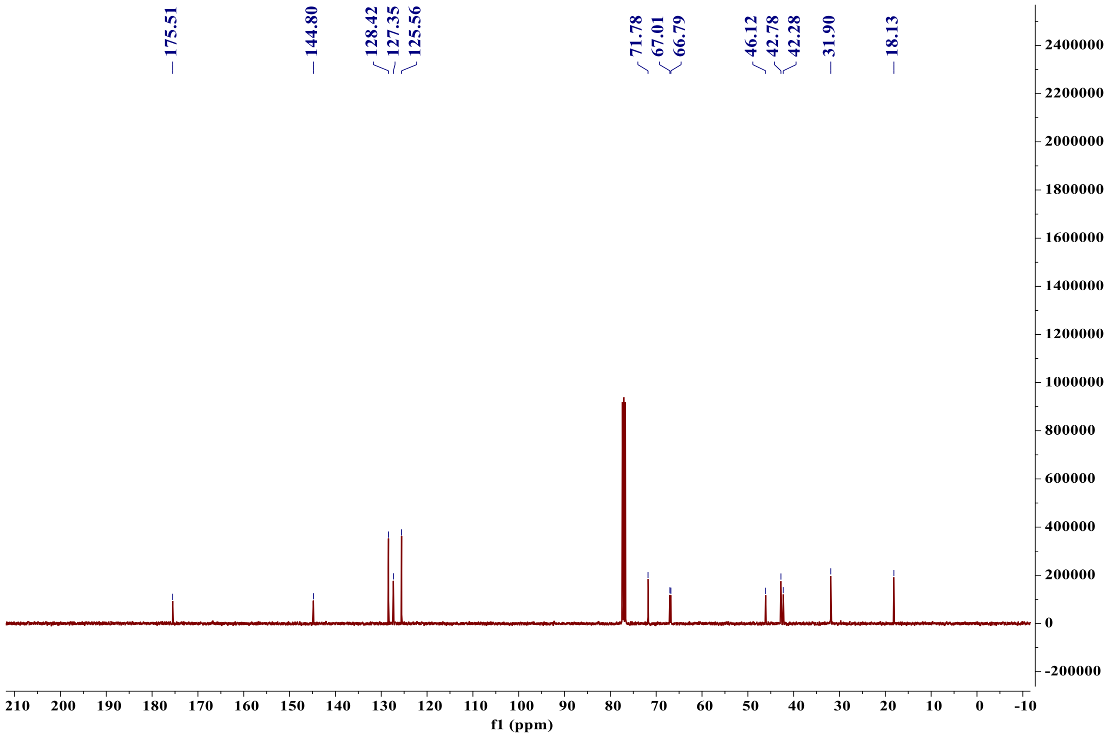

^{13}C NMR (101 MHz, $CDCl_3$) spectrum of ***(S,R)*-6**

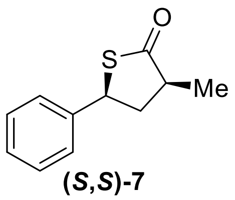

(*S*,*S*)-7

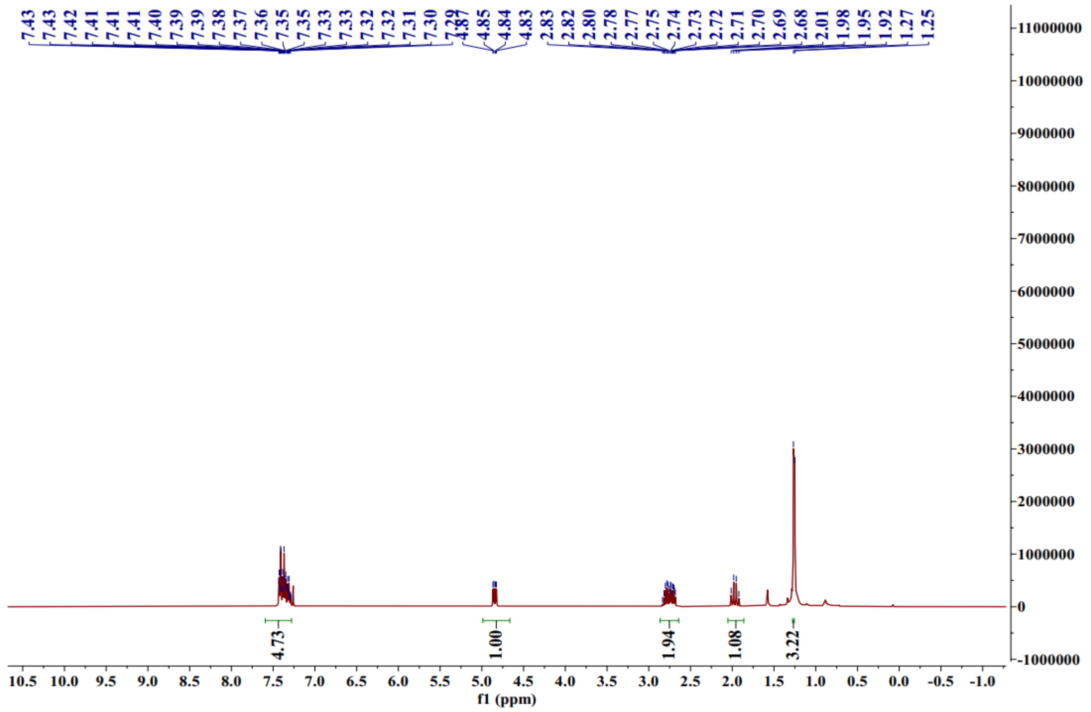

^{1}H NMR (400 MHz, $CDCl_3$) spectrum of **(*S*,*S*)-7**

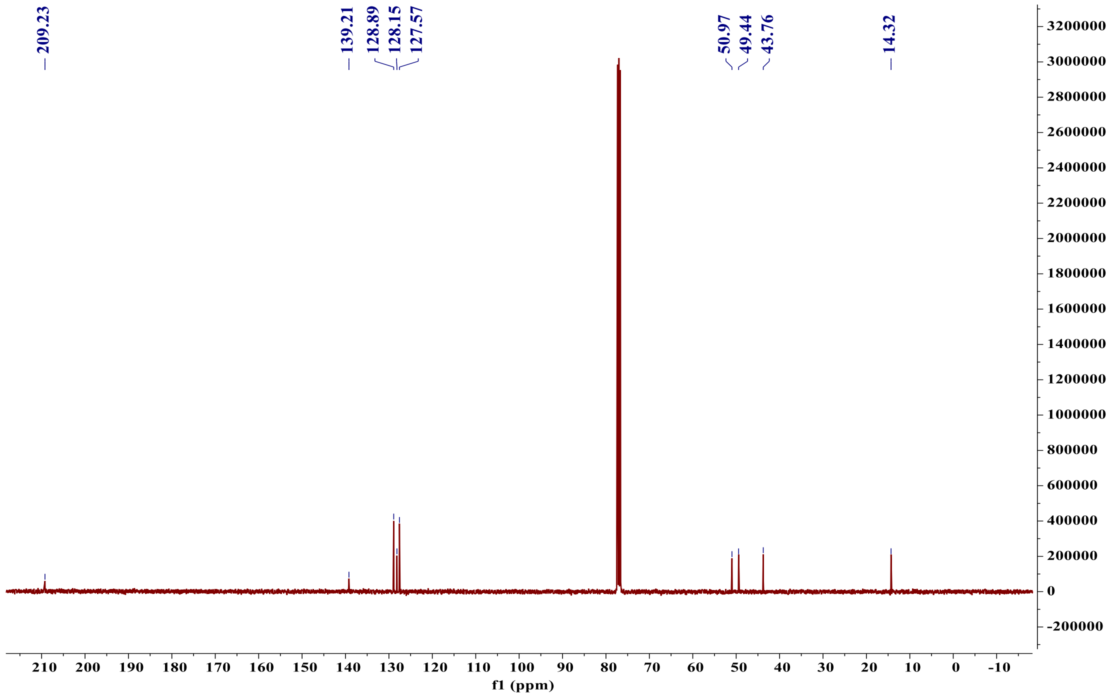

^{13}C NMR (101 MHz, $CDCl_3$) spectrum of **(*S*,*S*)-7**

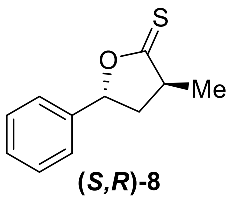

(*S*,*R*)-8

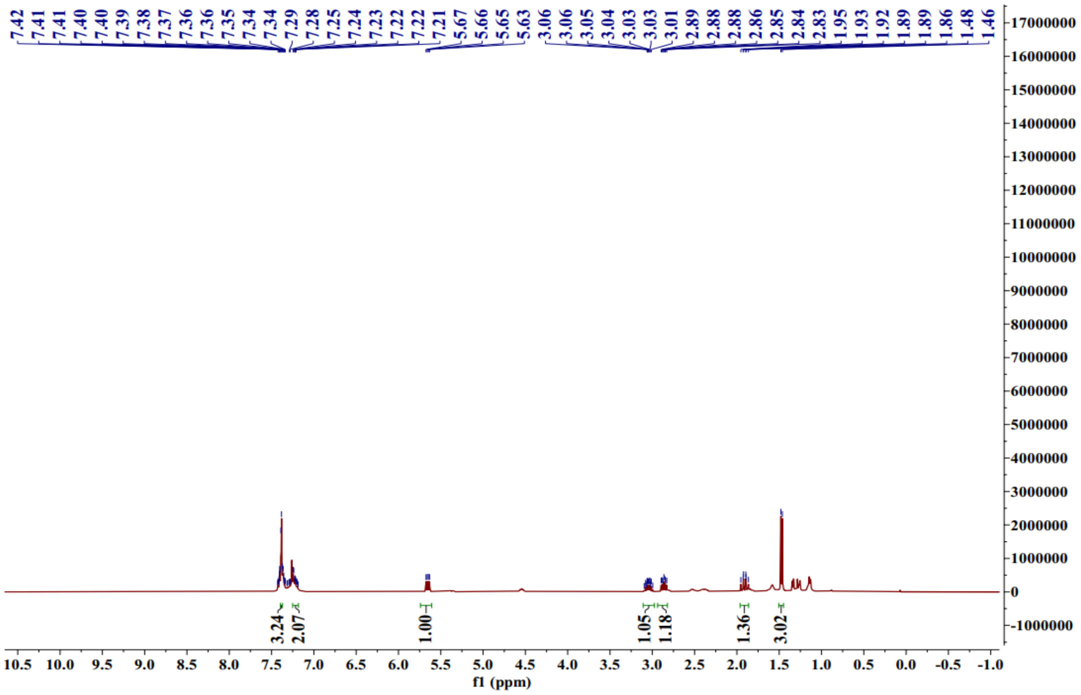

^{1}H NMR (400 MHz, $CDCl_3$) spectrum of **(*S*,*R*)-8**

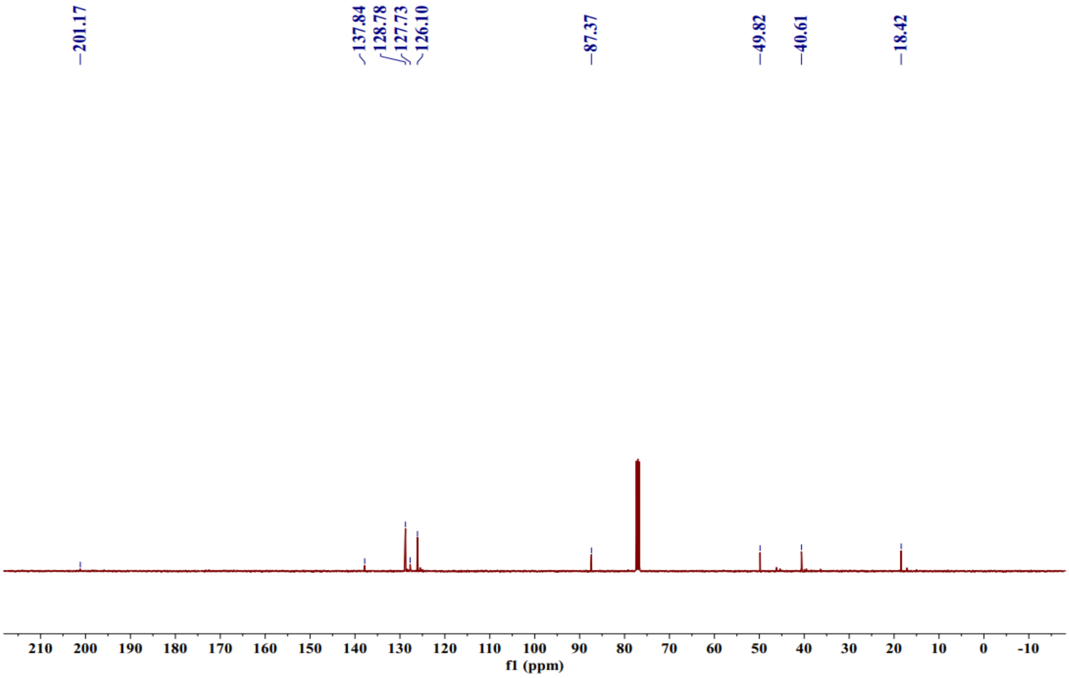

^{13}C NMR (101 MHz, $CDCl_3$) spectrum of **(*S*,*R*)-8**

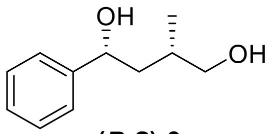

(*R*,*S*)-9

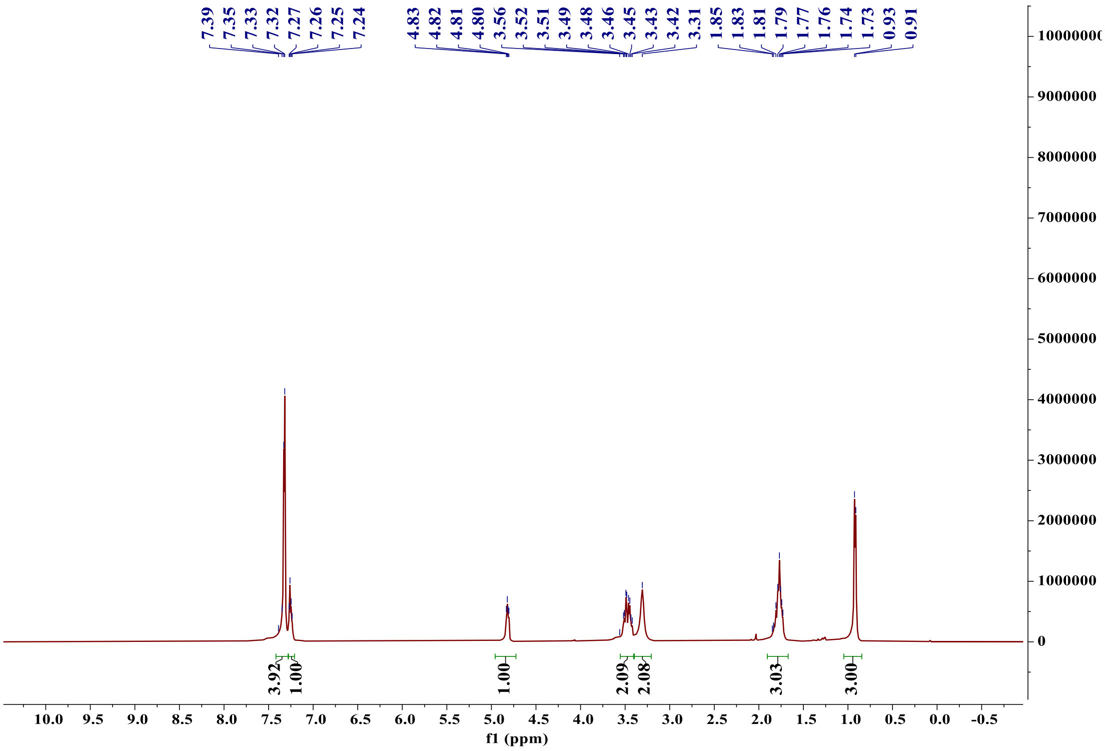

^{1}H NMR (400 MHz, $CDCl_3$) spectrum of ***(R,S)*-9**

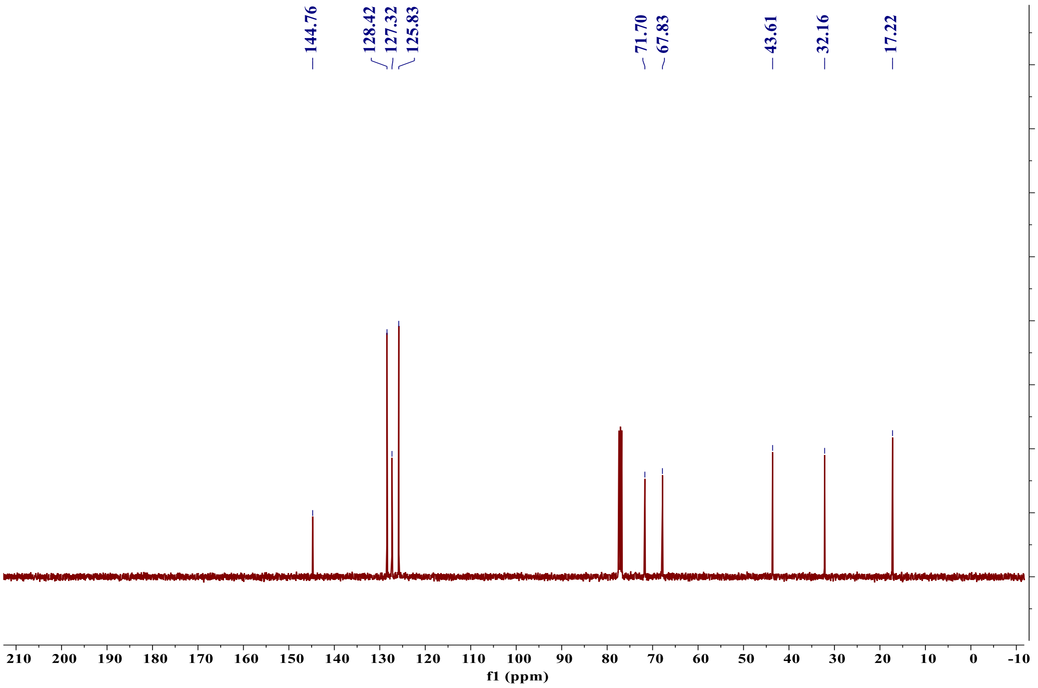

^{13}C NMR (101 MHz, $CDCl_3$) spectrum of ***(R,S)*-9**

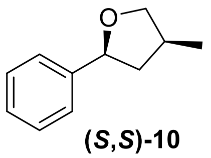

(*S*,*S*)-10

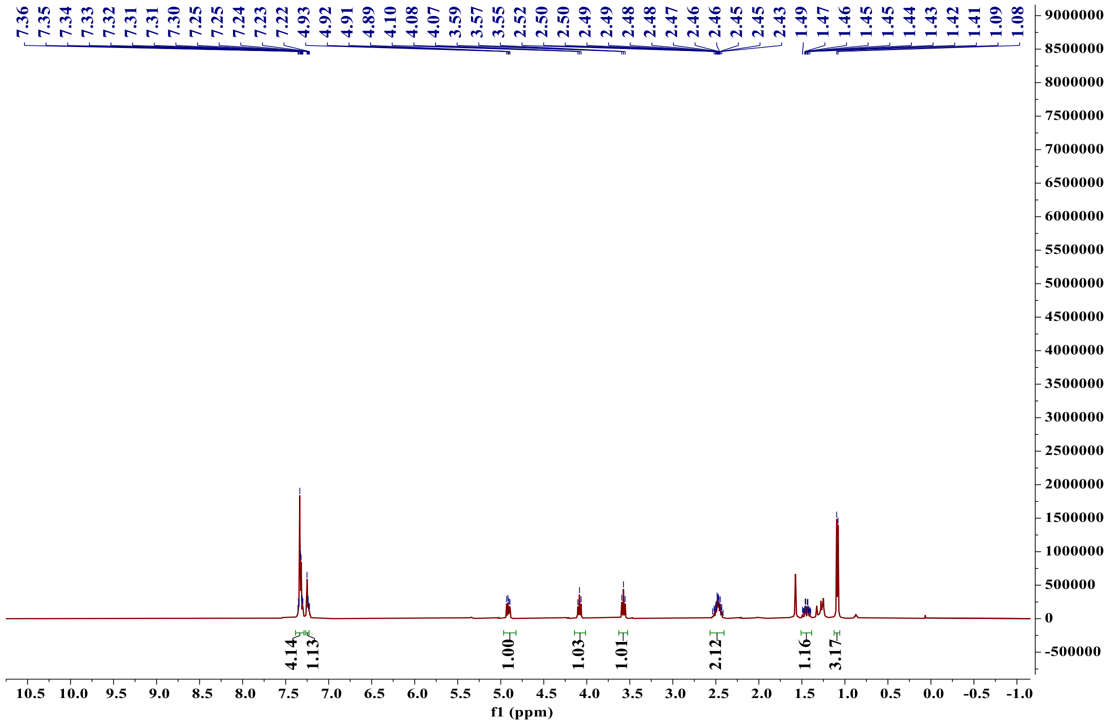

^{1}H NMR (400 MHz, $CDCl_3$) spectrum of **(*S*,*S*)-10**

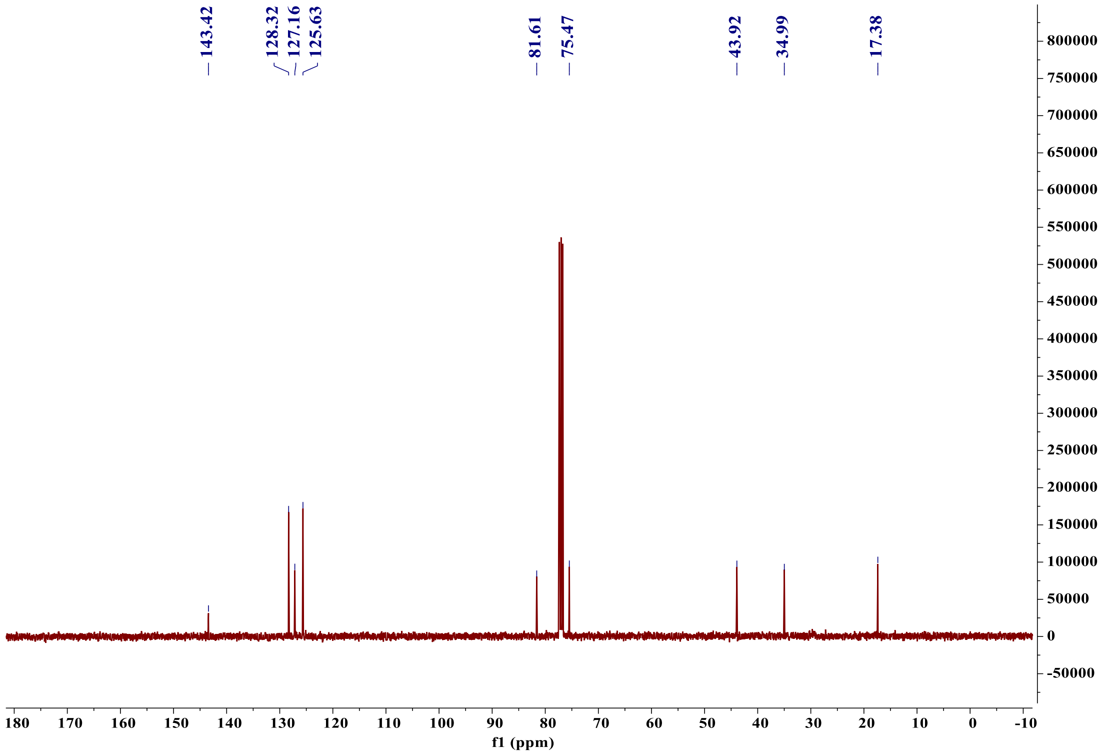

^{13}C NMR (101 MHz, $CDCl_3$) spectrum of **(*S*,*S*)-10**

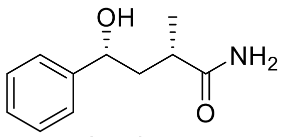

(*S*,*R*)-11

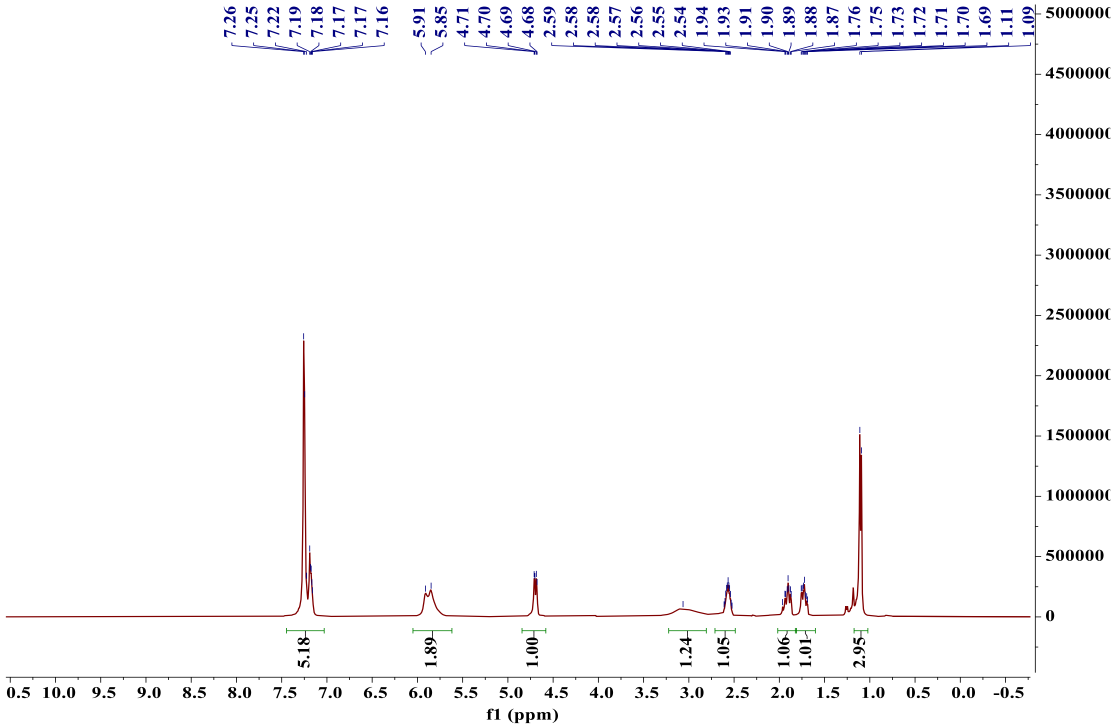

^{1}H NMR (400 MHz, $CDCl_3$) spectrum of **(*S*,*R*)-11**

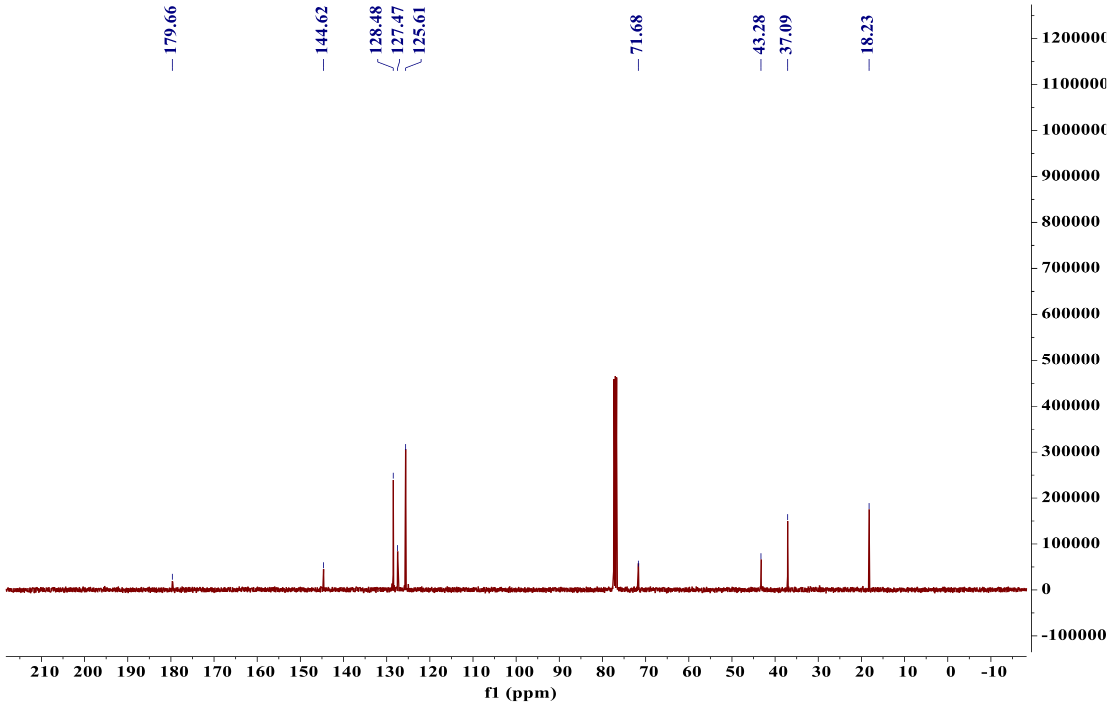

^{13}C NMR (101 MHz, $CDCl_3$) spectrum of **(*S*,*R*)-11**

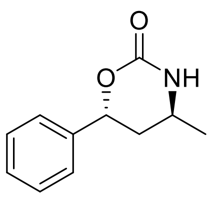

(*S*,*R*)-12

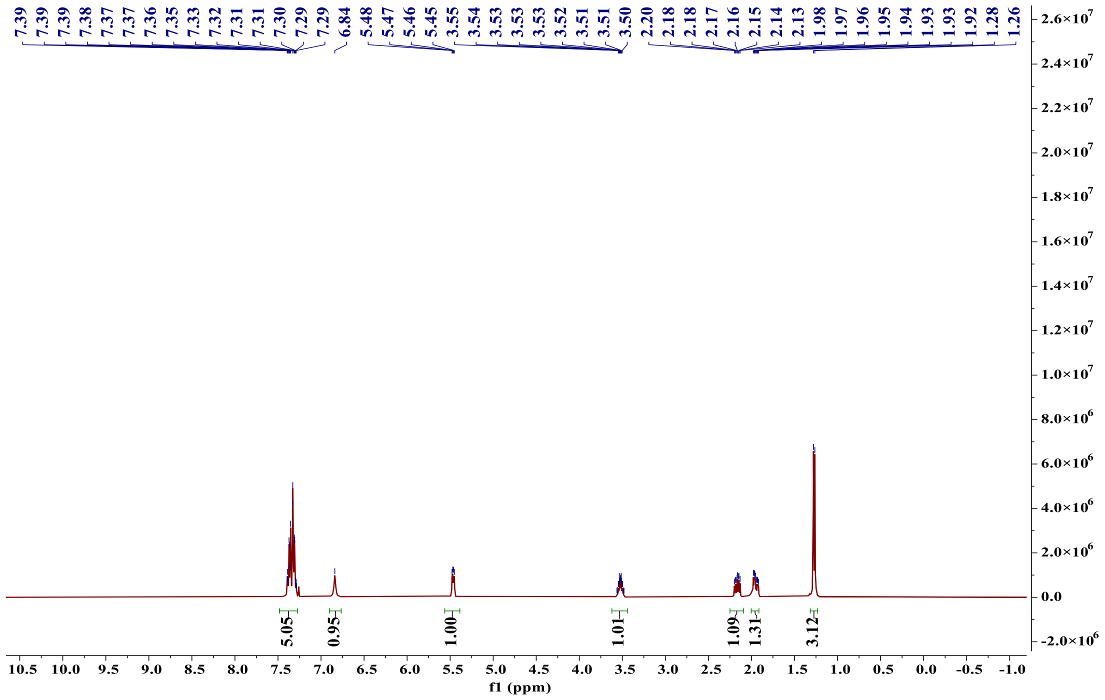

^{1}H NMR (400 MHz, $CDCl_3$) spectrum of **(*S*,*R*)-12**

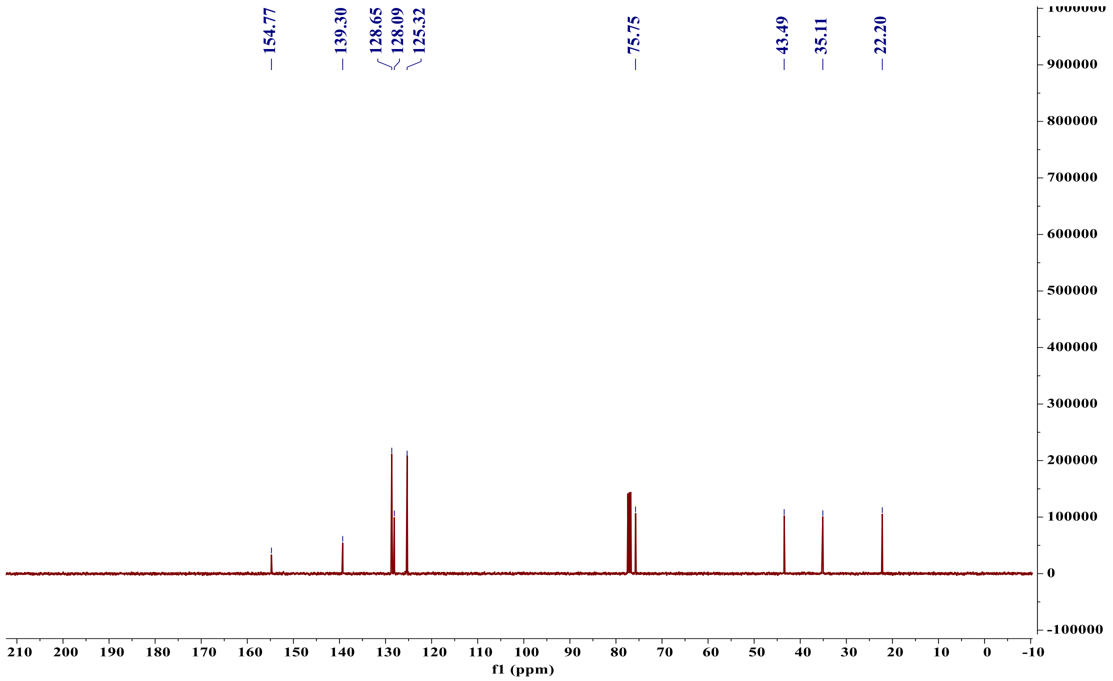

^{13}C NMR (101 MHz, $CDCl_3$) spectrum of **(*S*,*R*)-12**

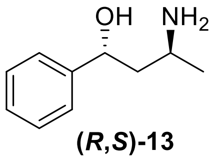

(*R*,*S*)-13

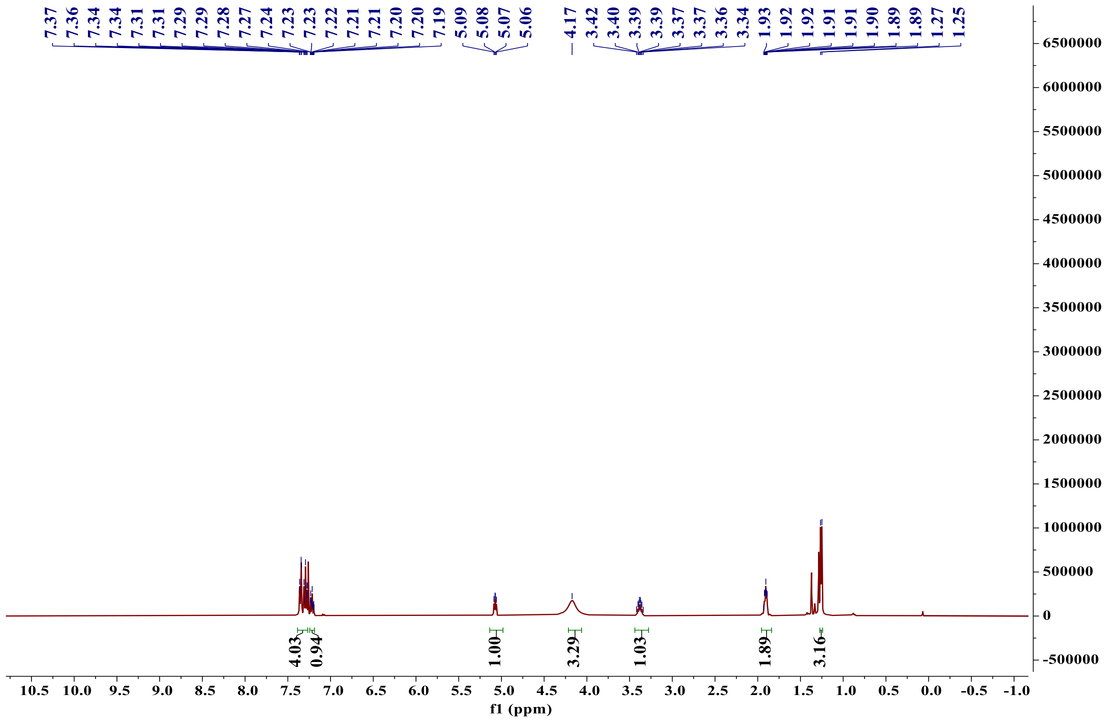

^{1}H NMR (400 MHz, $CDCl_3$) spectrum of ***(R,S)*-13**

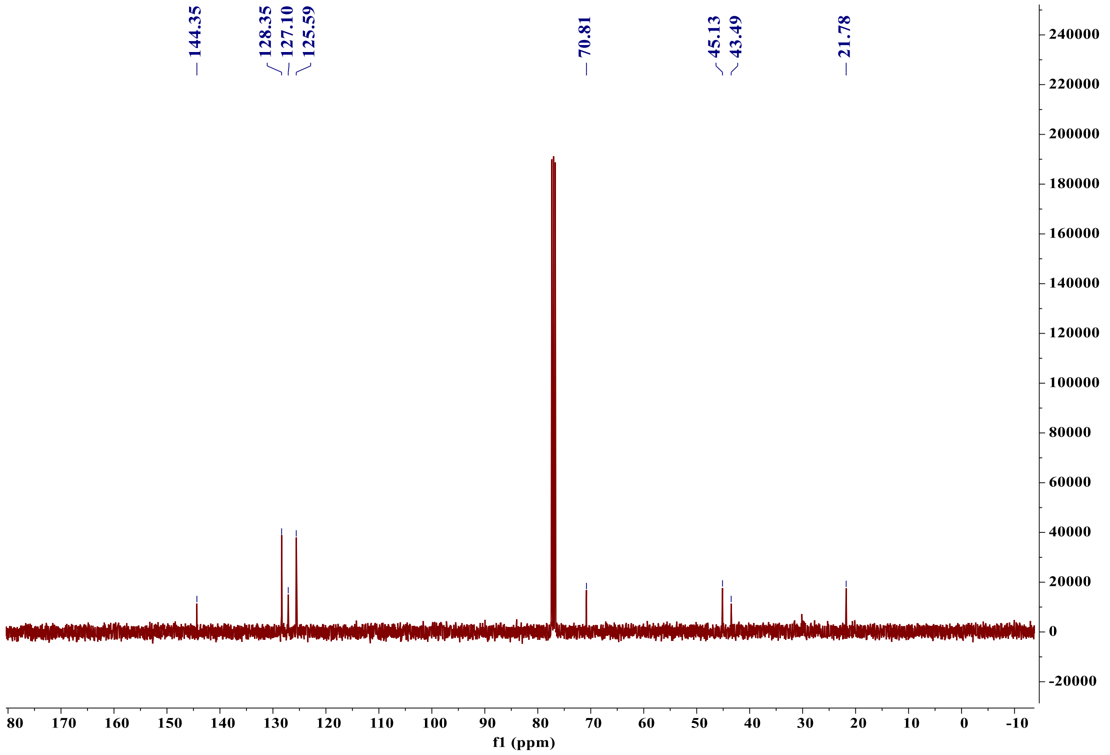

^{13}C NMR (101 MHz, $CDCl_3$) spectrum of ***(R,S)*-13**

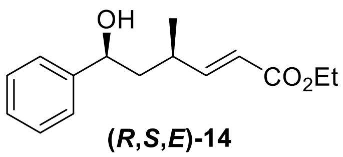

(*R*,*S*,*E*)-14

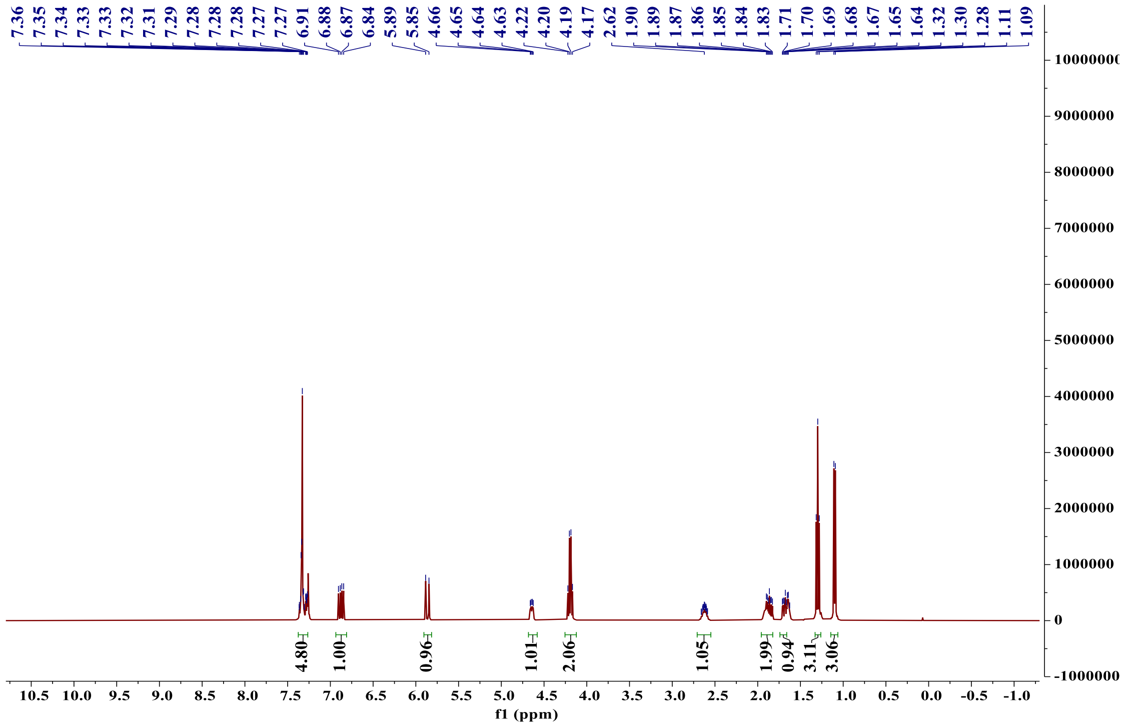

^{1}H NMR (400 MHz, $CDCl_3$) spectrum of ***(R,S,E)*-14**

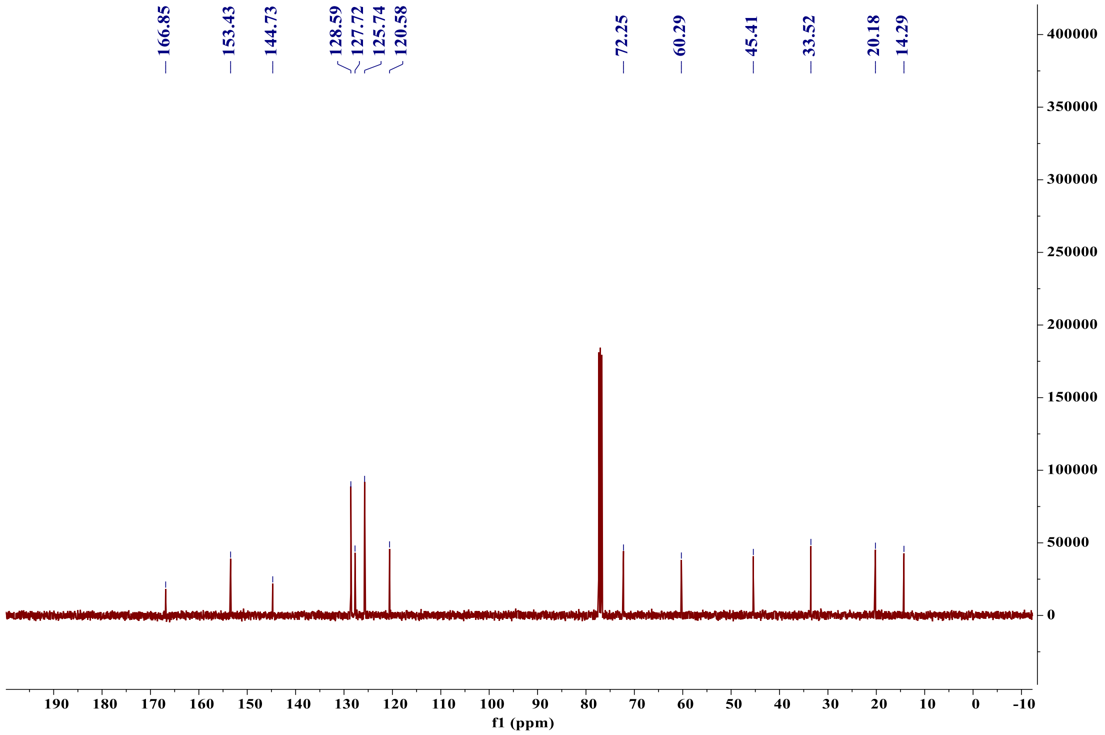

^{13}C NMR (101 MHz, $CDCl_3$) spectrum of ***(R,S,E)*-14**

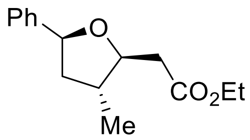

(*S*,*R*,*S*)-15

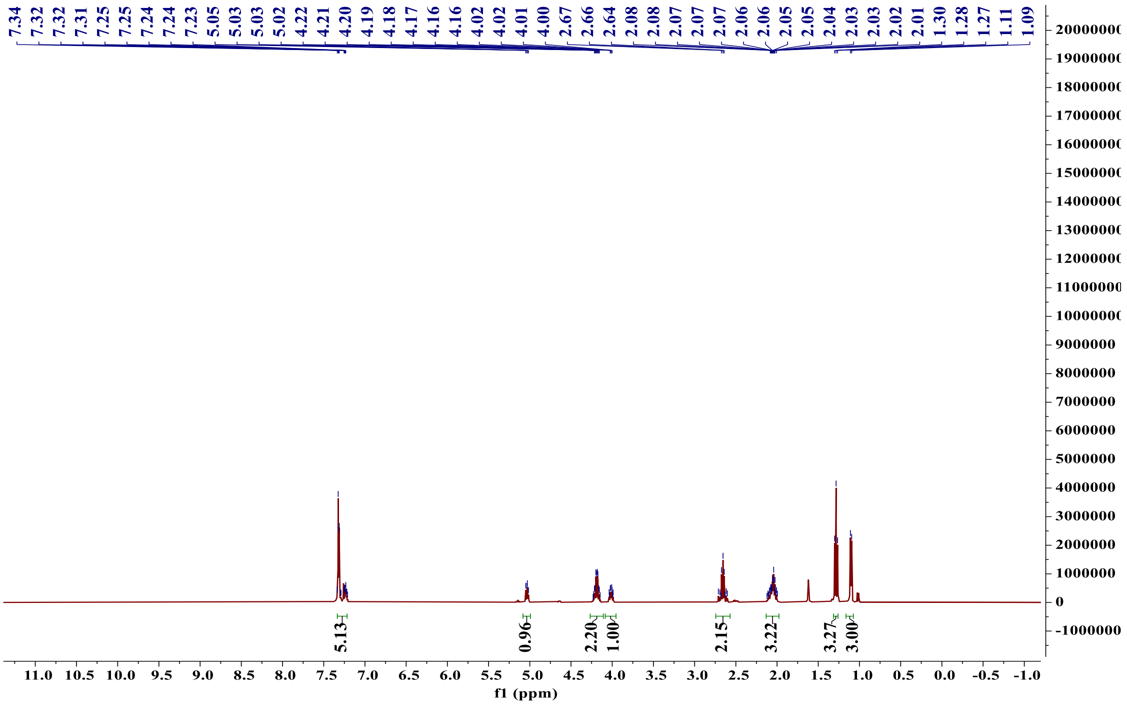

^{1}H NMR (400 MHz, $CDCl_3$) spectrum of **(*S*,*R*,*S*)-15**

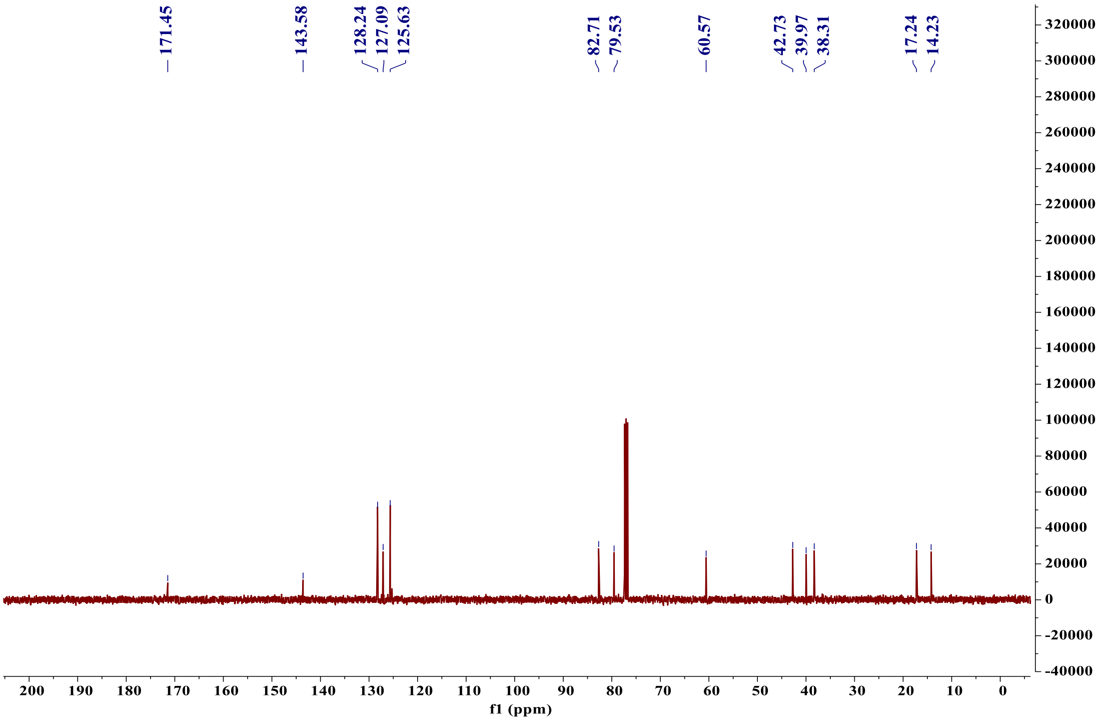

^{13}C NMR (101 MHz, $CDCl_3$) spectrum of **(*S*,*R*,*S*)-15**

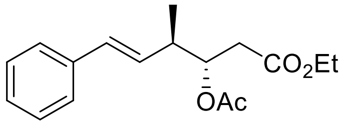

(*S*,*R*,*E*)-16

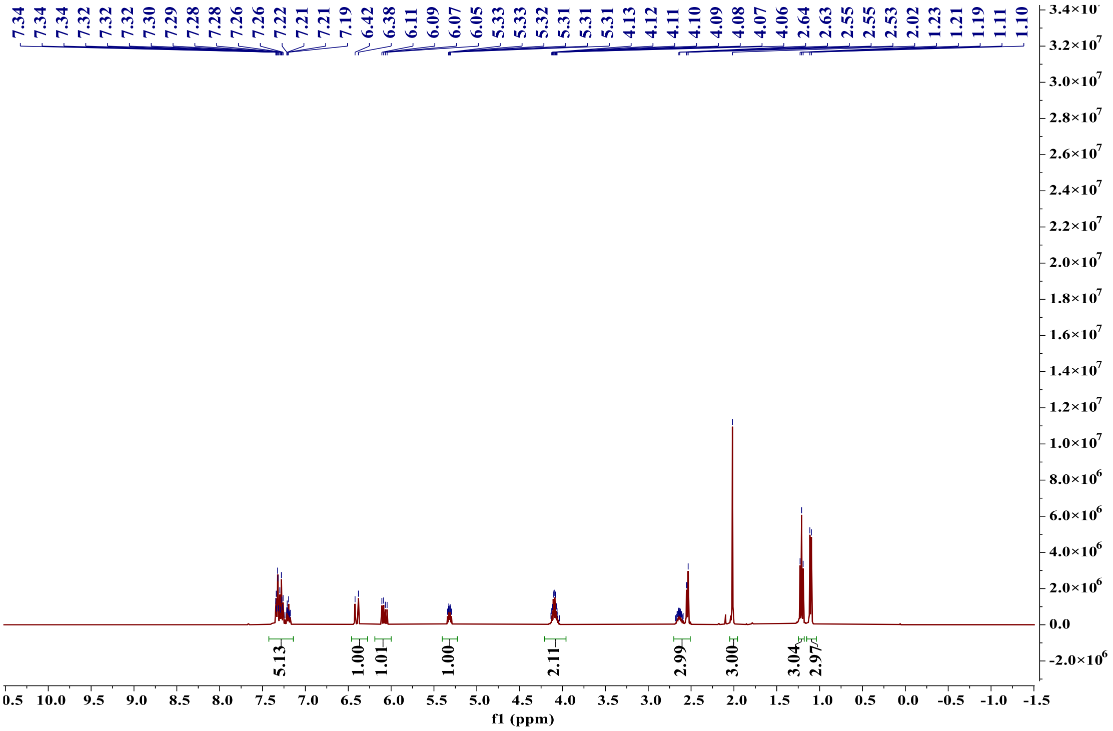

^{1}H NMR (400 MHz, $CDCl_3$) spectrum of **(*S*,*R*,*E*)-16**

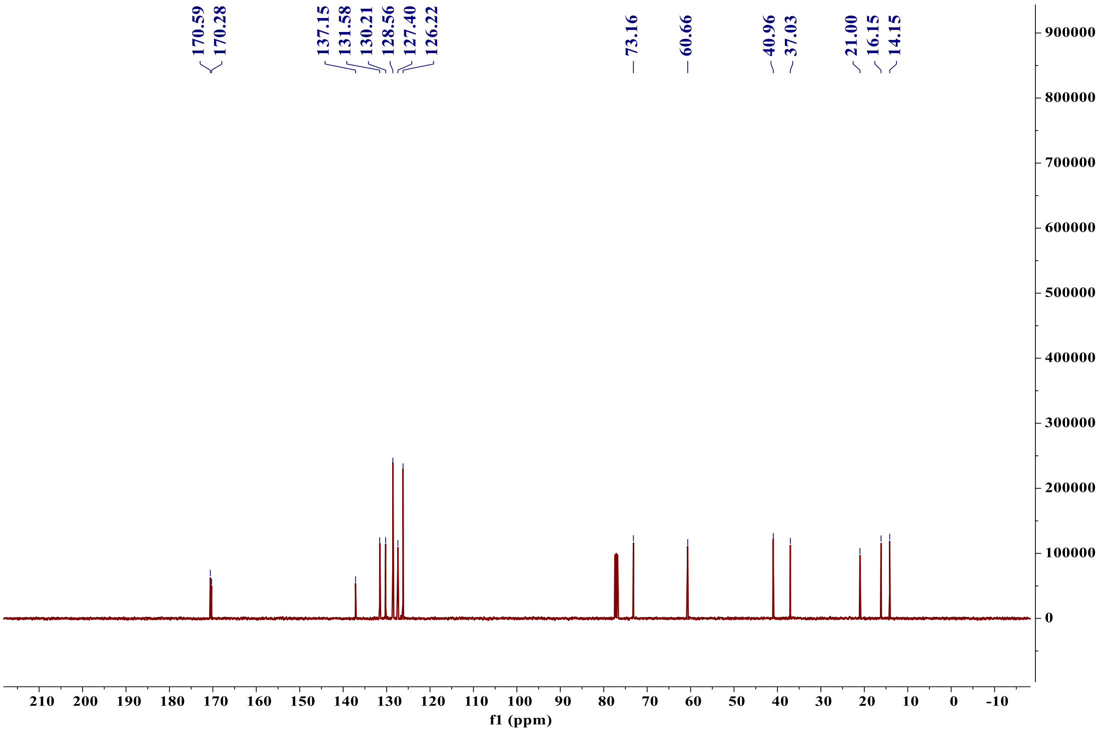

^{13}C NMR (101 MHz, $CDCl_3$) spectrum of **(*S*,*R*,*E*)-16**

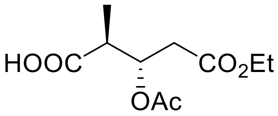

(*S*,*S*)-17

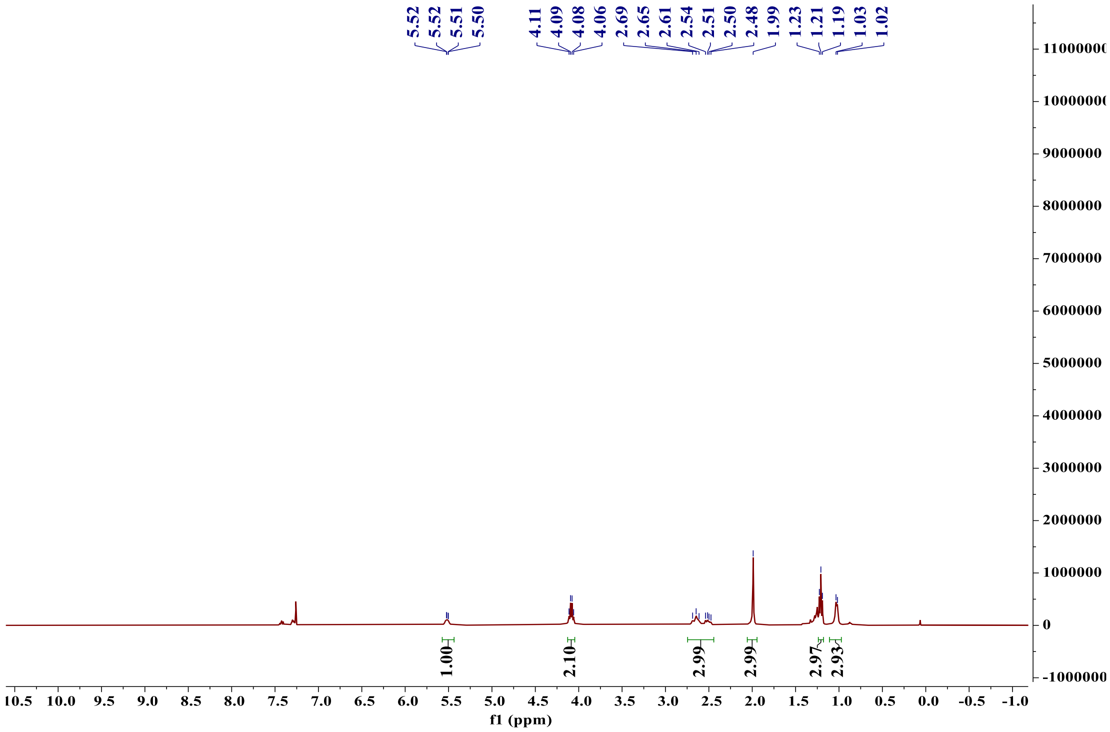

^{1}H NMR (400 MHz, $CDCl_3$) spectrum of **(*S*,*S*)-17**

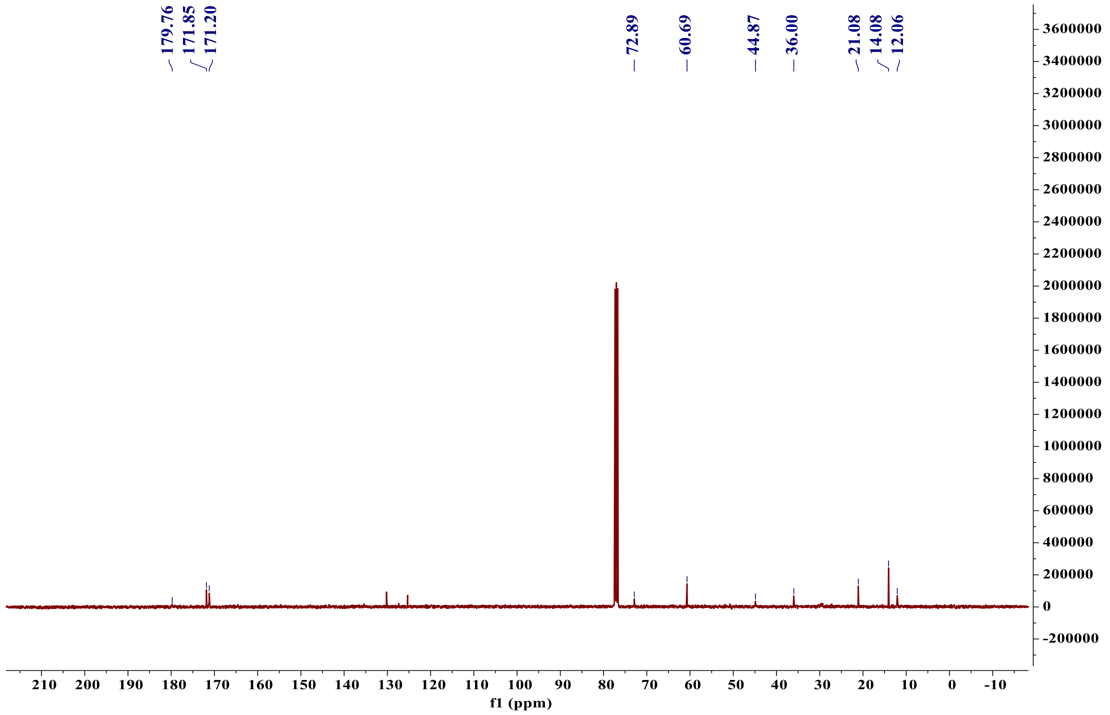

^{13}C NMR (101 MHz, $CDCl_3$) spectrum of **(*S*,*S*)-17**

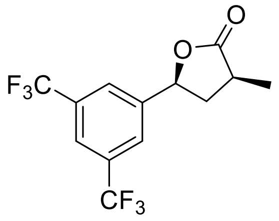

(*S*,*S*)-3y

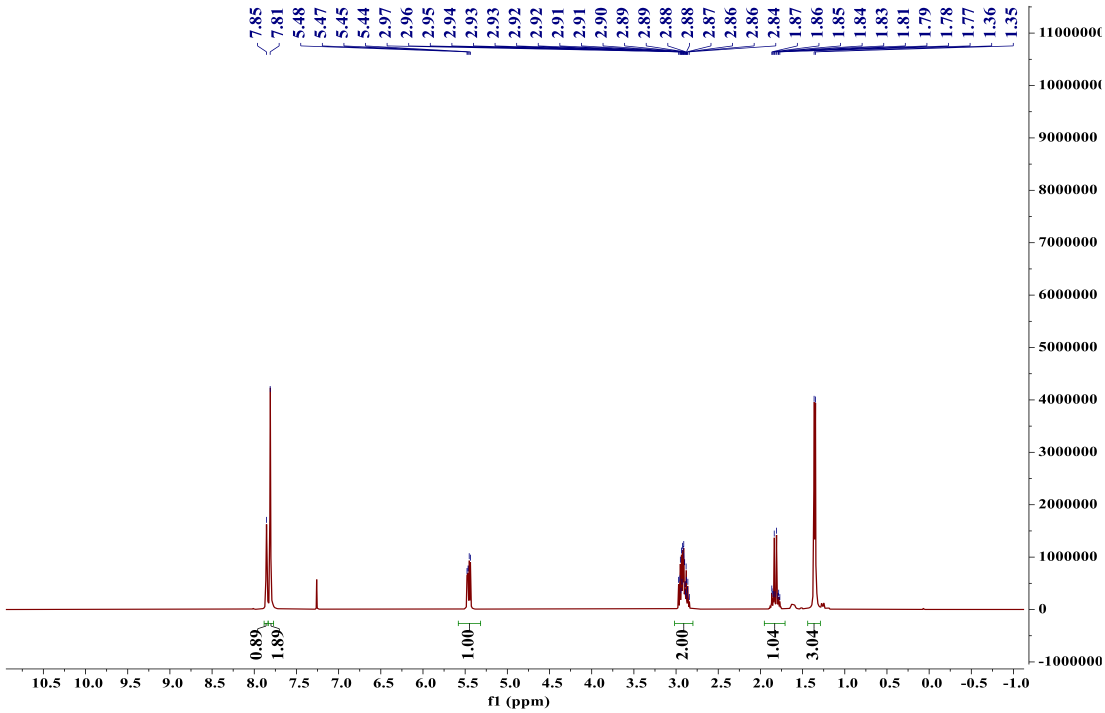

^{1}H NMR (400 MHz, $CDCl_3$) spectrum of **(*S*,*S*)-3y**

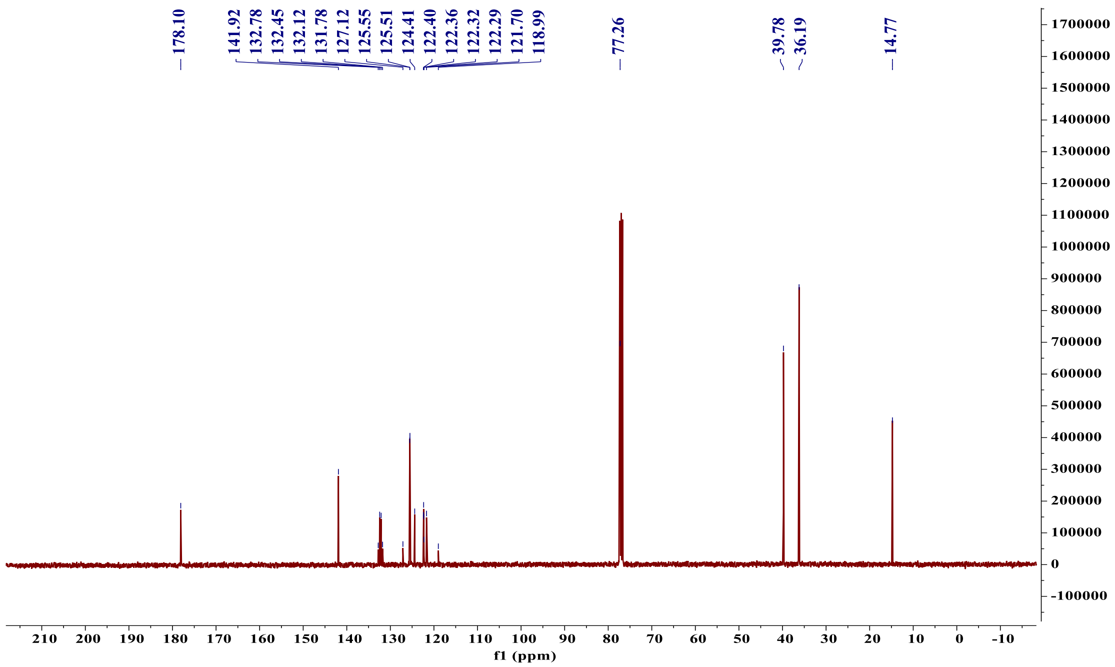

^{13}C NMR (101 MHz, $CDCl_3$) spectrum of **(*S*,*S*)-3y**

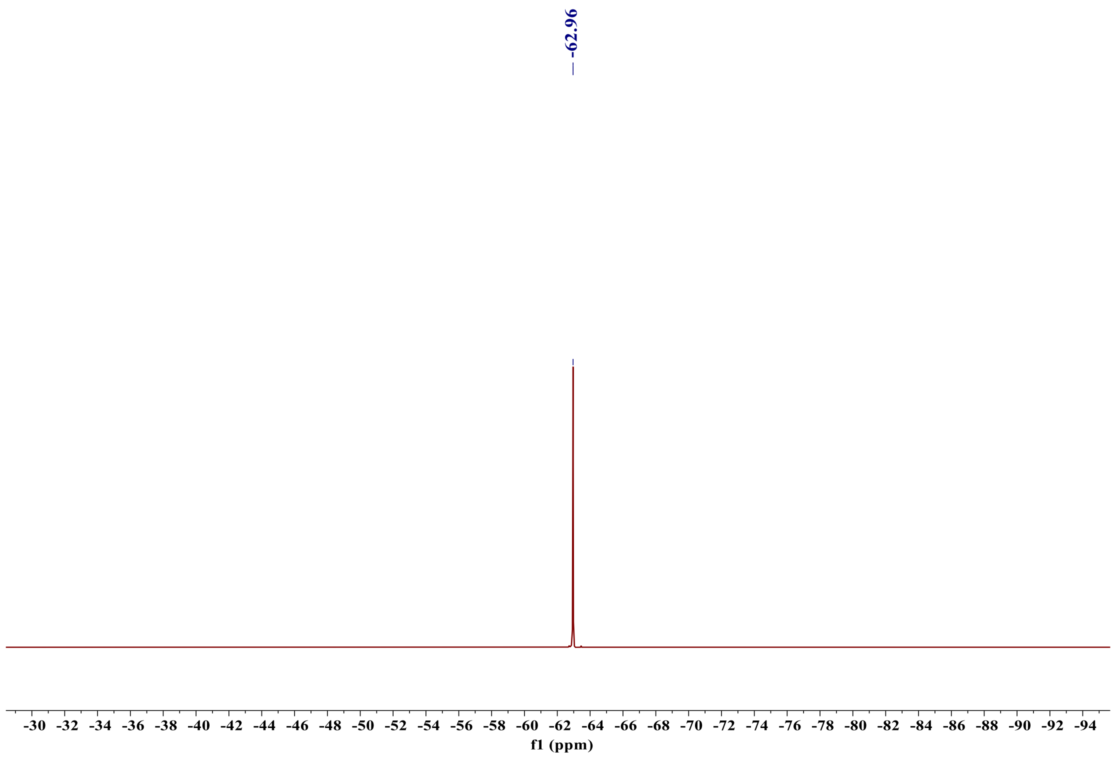

^{19}F NMR (377 MHz, $CDCl_3$) spectrum of **(*S*,*S*)-3y**

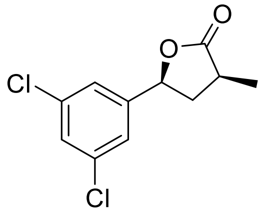

(*S*,*S*)-3z

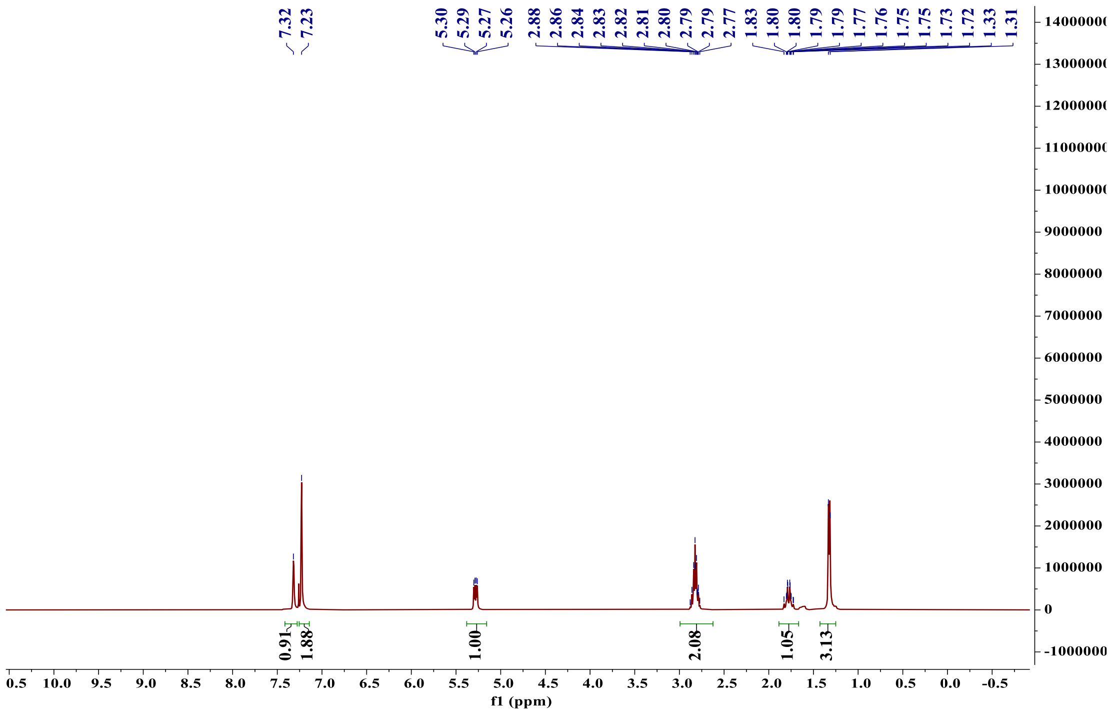

^{1}H NMR (400 MHz, $CDCl_3$) spectrum of **(*S*,*S*)-3z**

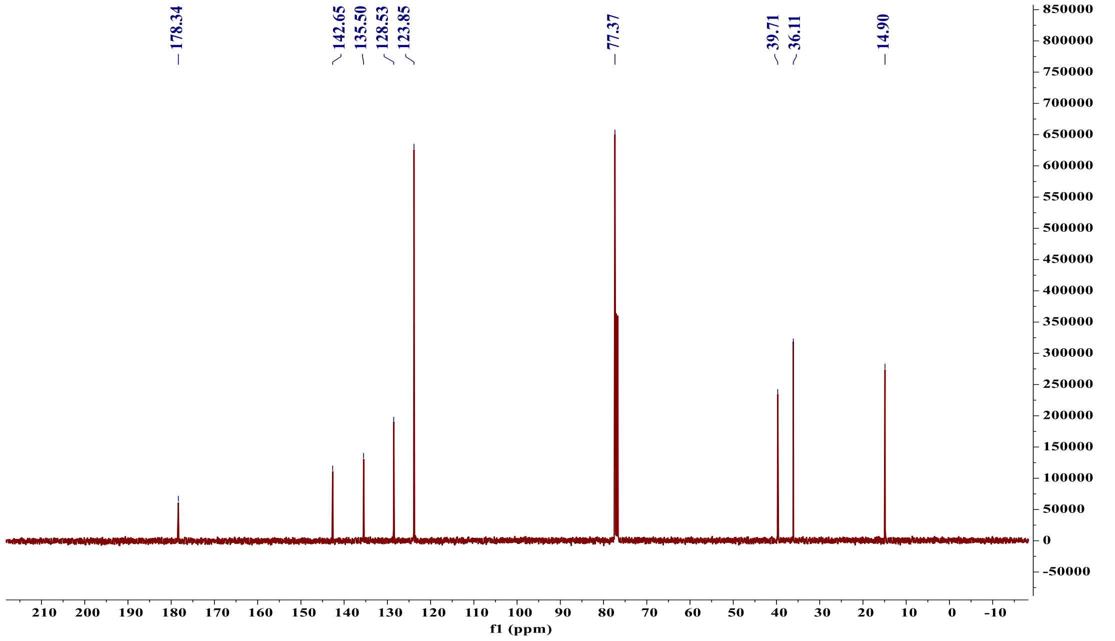

^{13}C NMR (101 MHz, $CDCl_3$) spectrum of **(*S*,*S*)-3z**

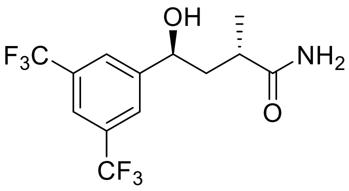

(*S*,*S*)-18

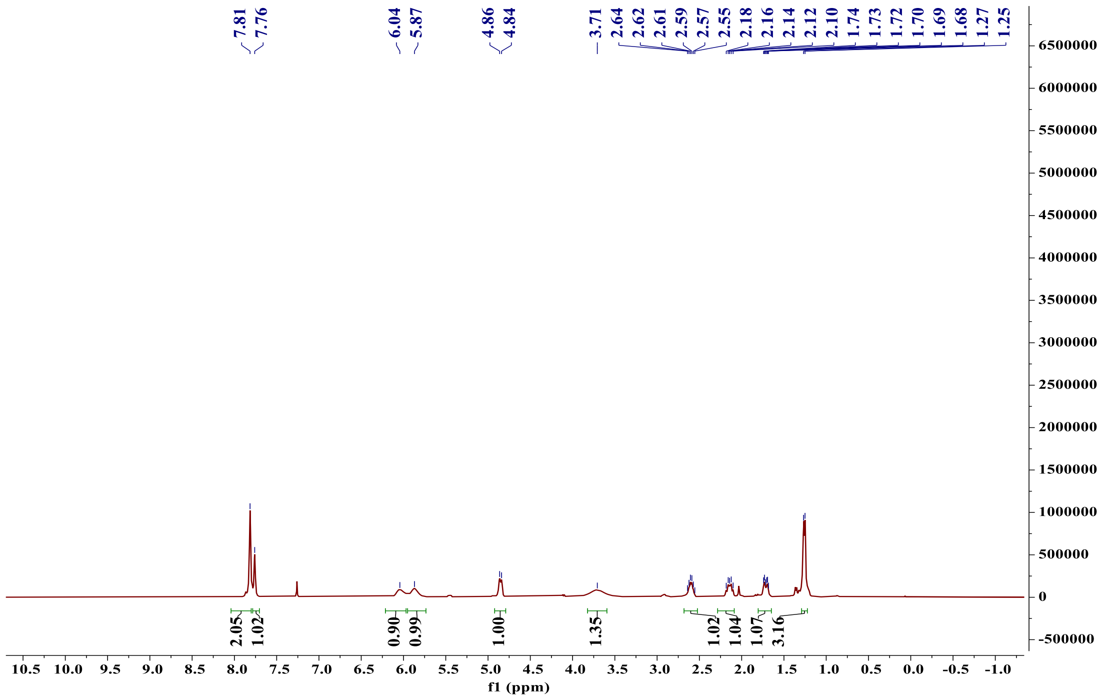

^{1}H NMR (400 MHz, $CDCl_3$) spectrum of ***(S,S)*-18**

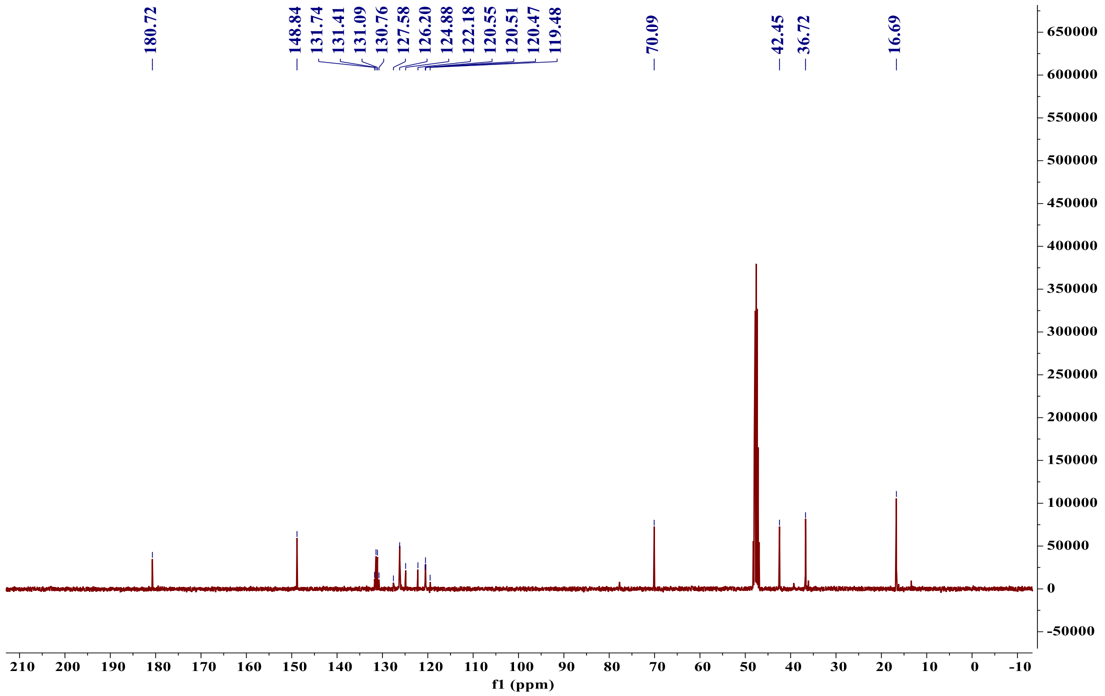

^{13}C NMR (101 MHz, $CDCl_3$) spectrum of ***(S,S)*-18**

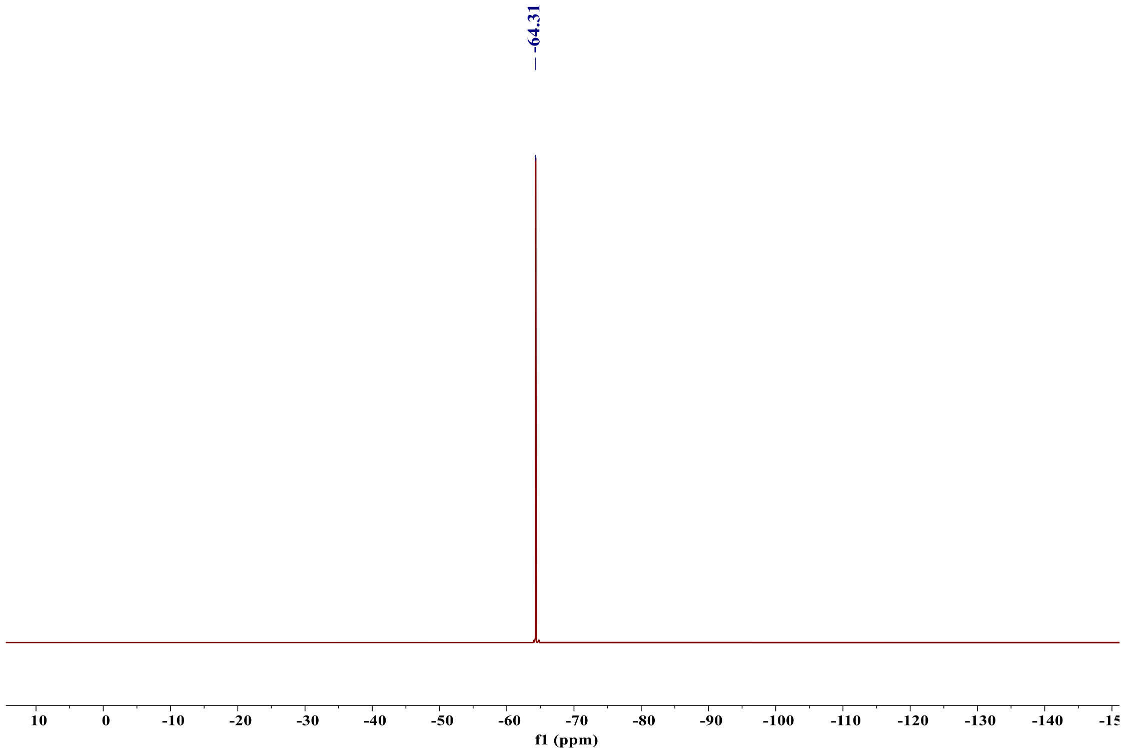

^{19}F NMR (377 MHz, $CDCl_3$) spectrum of **(*S*,*S*)-18**

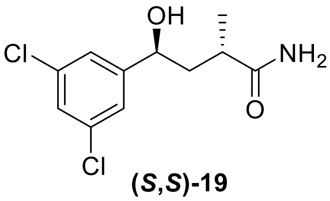

(***S***,***S***)-**19**

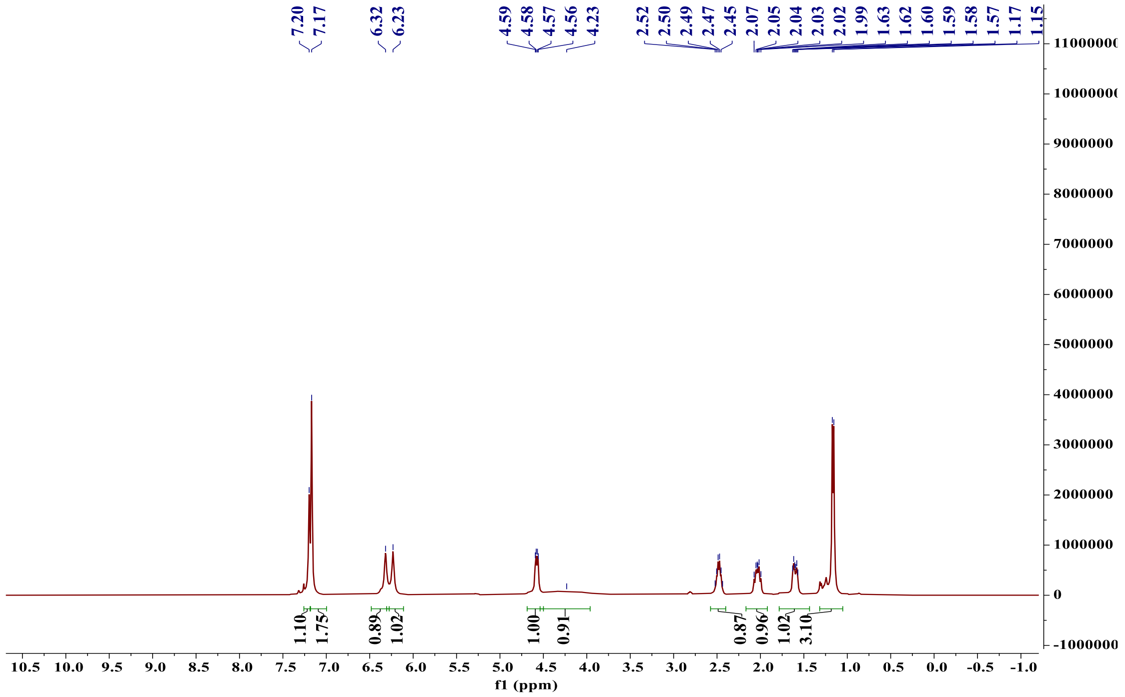

^{1}H NMR (400 MHz, $CDCl_3$) spectrum of (***S***,***S***)-**19**

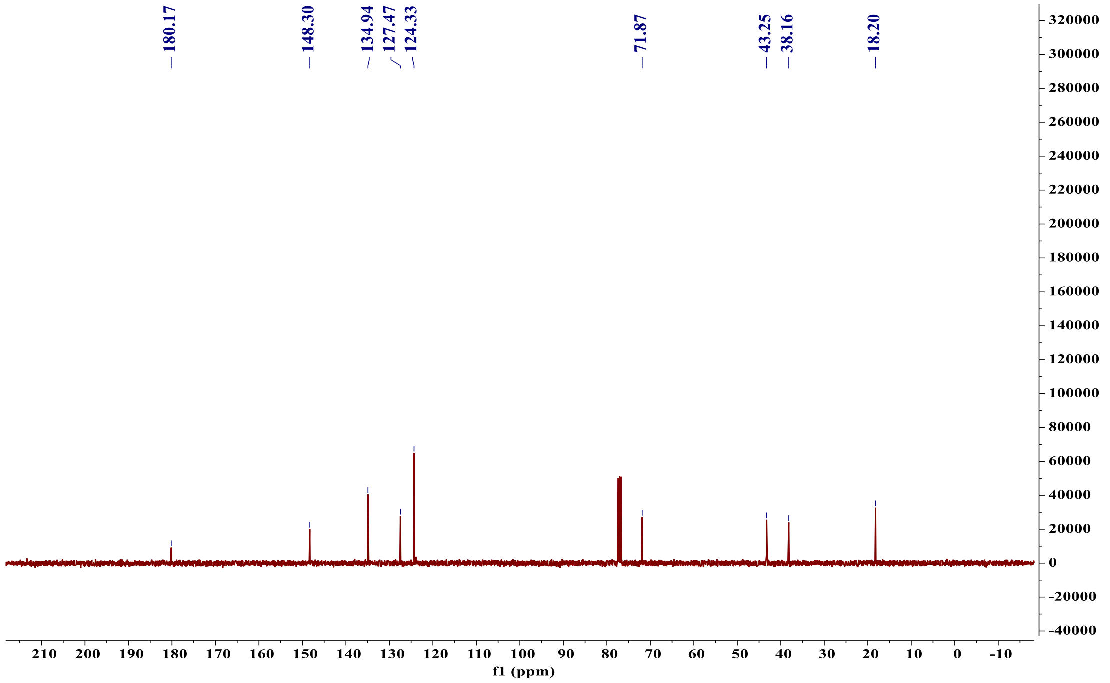

^{13}C NMR (101 MHz, $CDCl_3$) spectrum of (***S***,***S***)-**19**

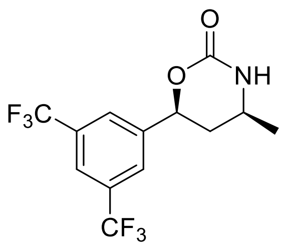

(*S*,*S*)-20

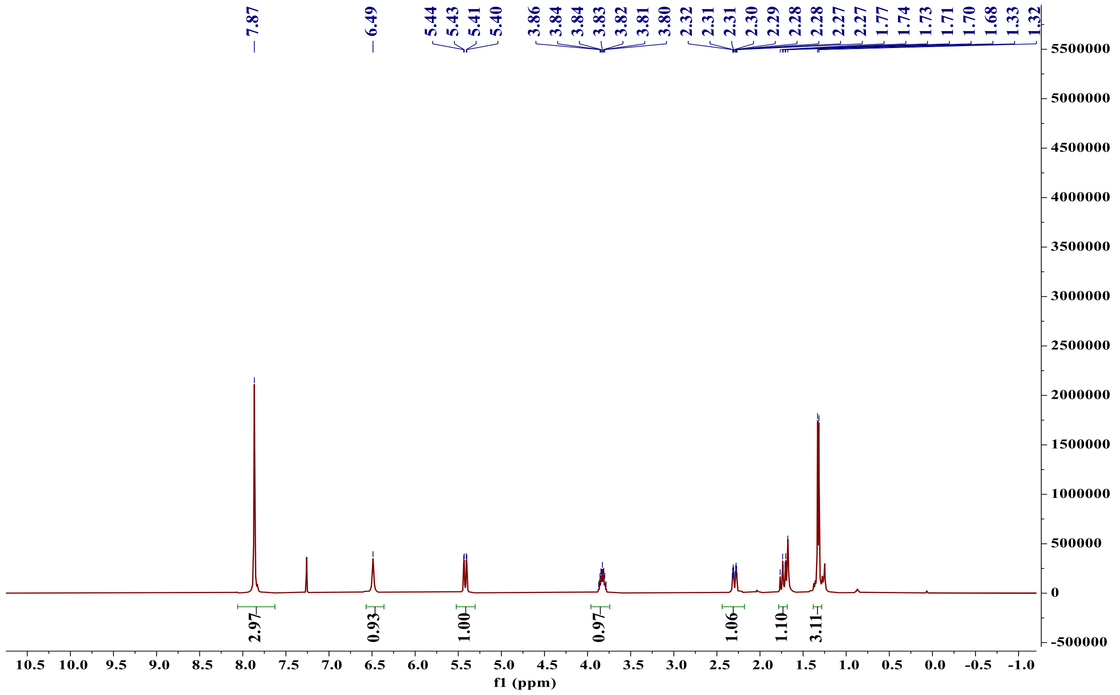

^{1}H NMR (400 MHz, $CDCl_3$) spectrum of **(*S*,*S*)-20**

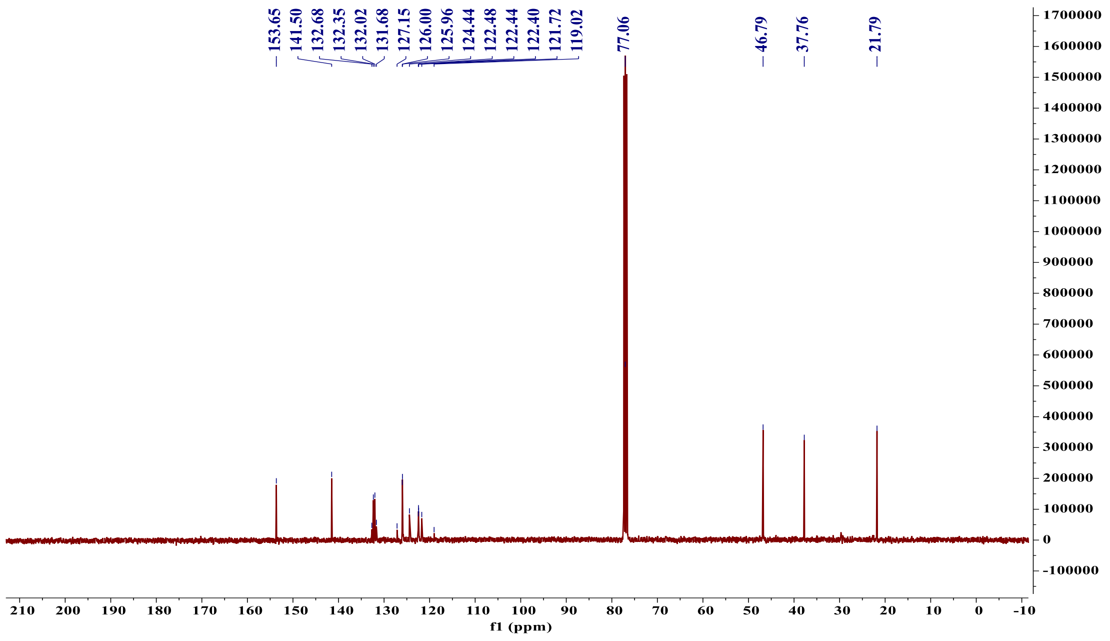

^{13}C NMR (101 MHz, $CDCl_3$) spectrum of **(*S*,*S*)-20**

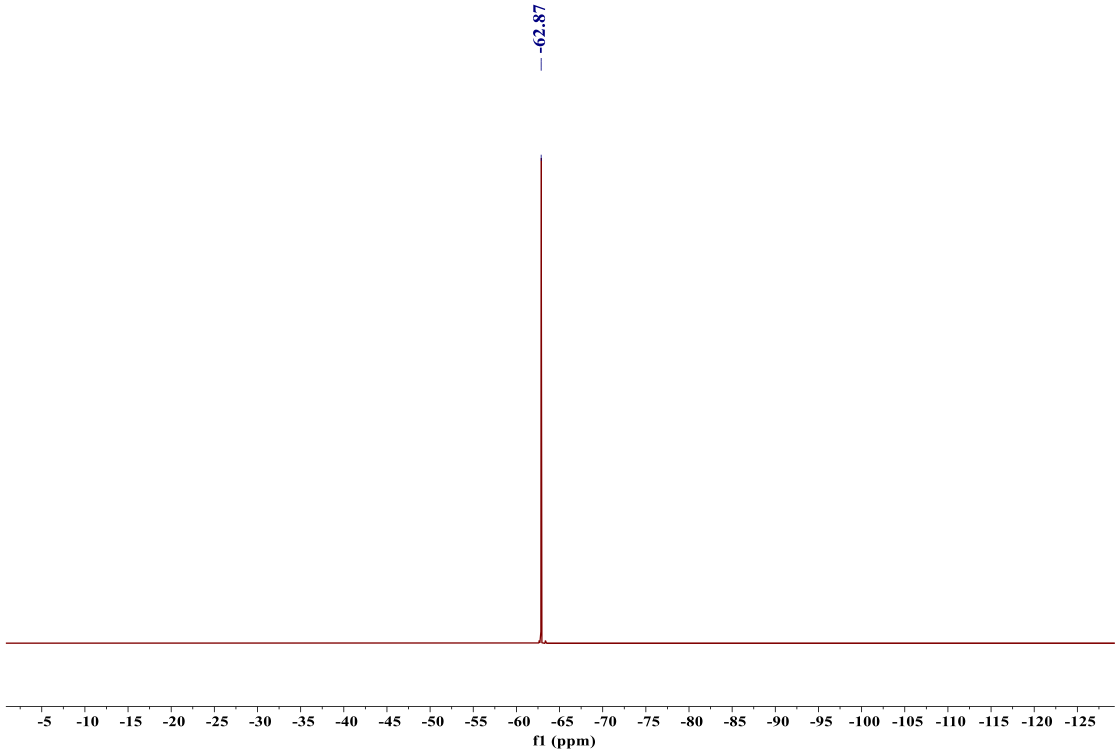

^{19}F NMR (377 MHz, $CDCl_3$) spectrum of ***(S,S)*-20**

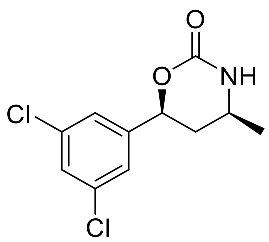

(*S*,*S*)-21

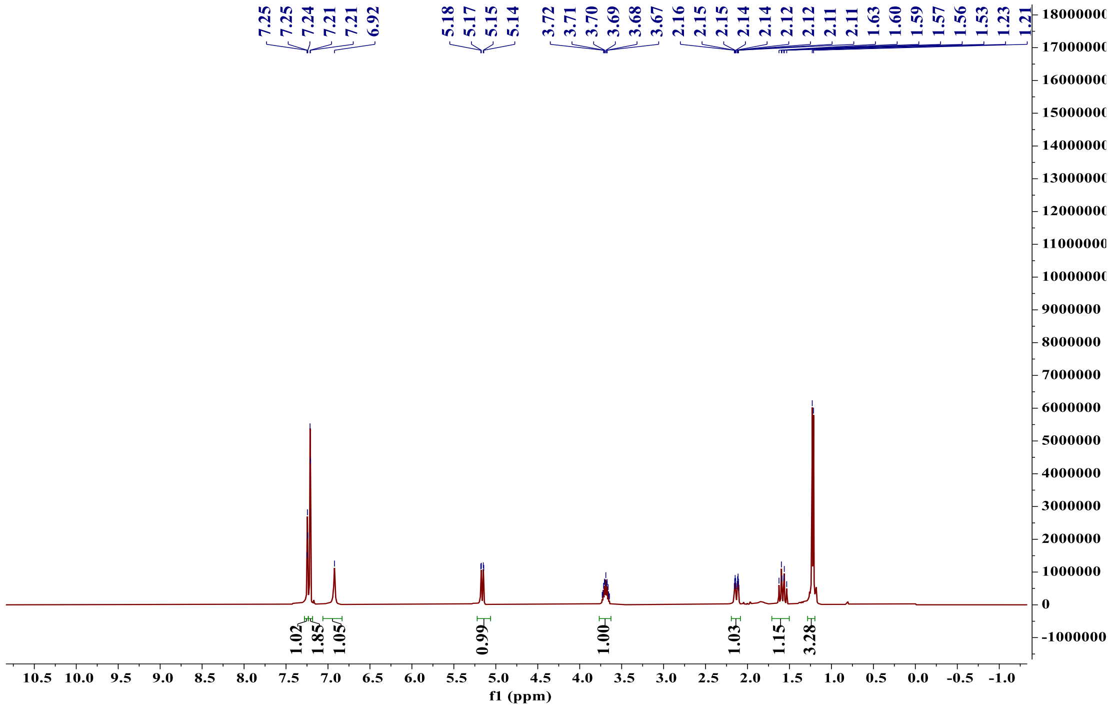

^{1}H NMR (400 MHz, $CDCl_3$) spectrum of **(*S*,*S*)-21**

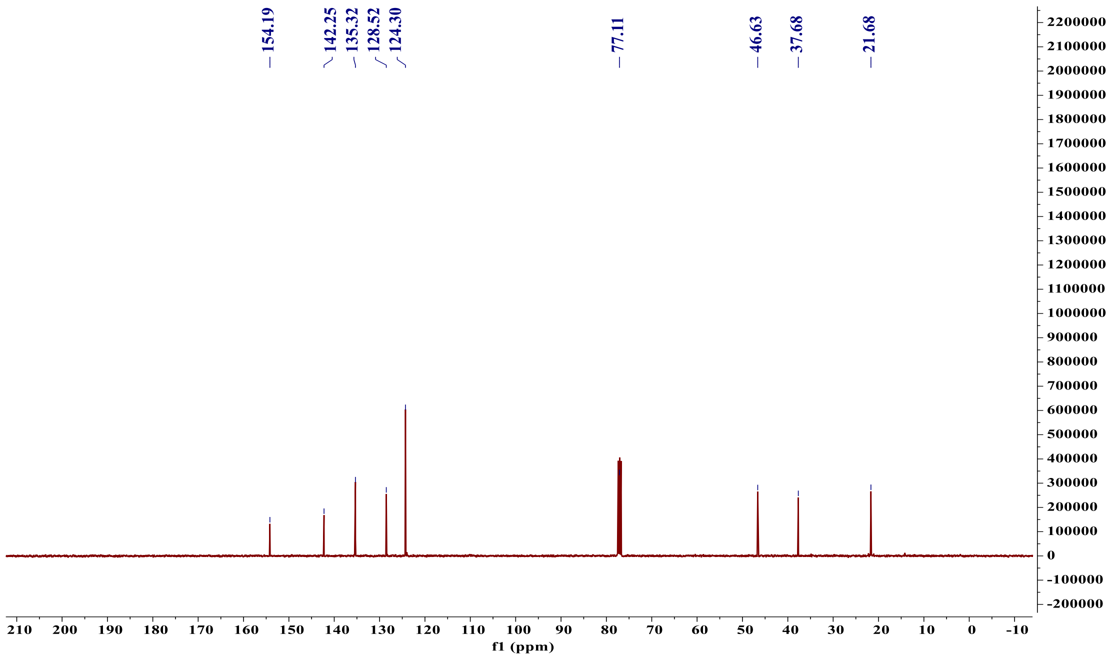

^{13}C NMR (101 MHz, $CDCl_3$) spectrum of **(*S*,*S*)-21**

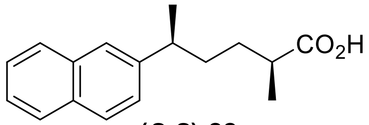

(*S*,*S*)-22

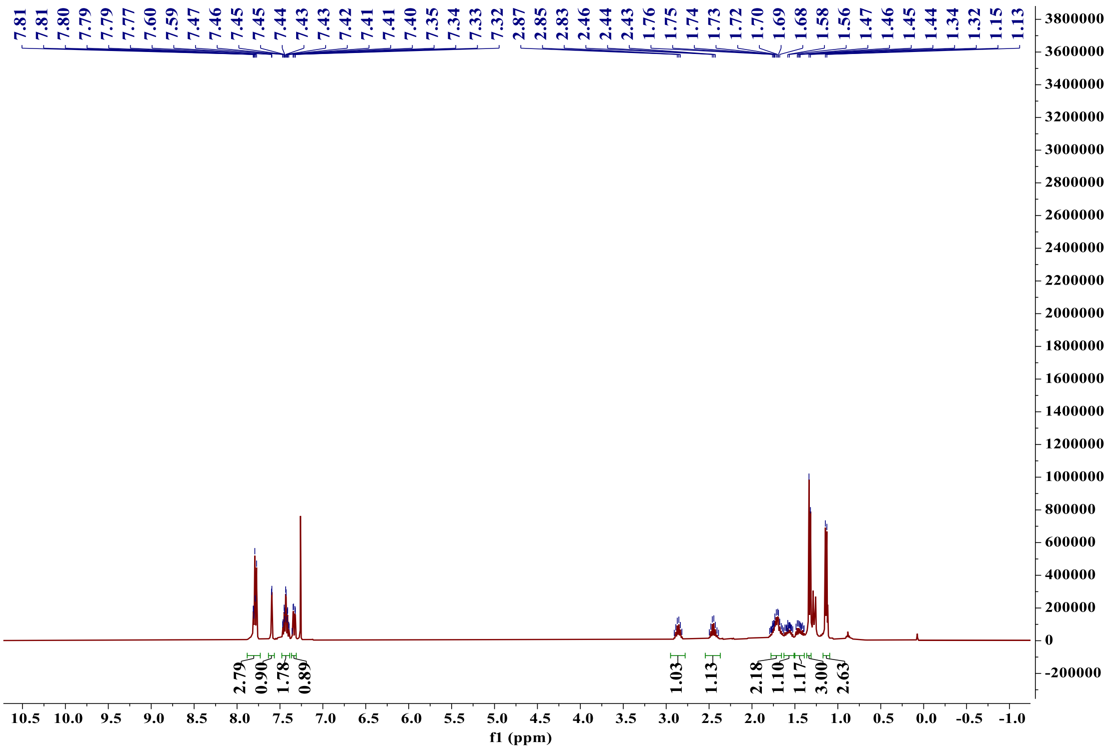

^{1}H NMR (400 MHz, CDCl3) spectrum of **(*S*,*S*)-22**

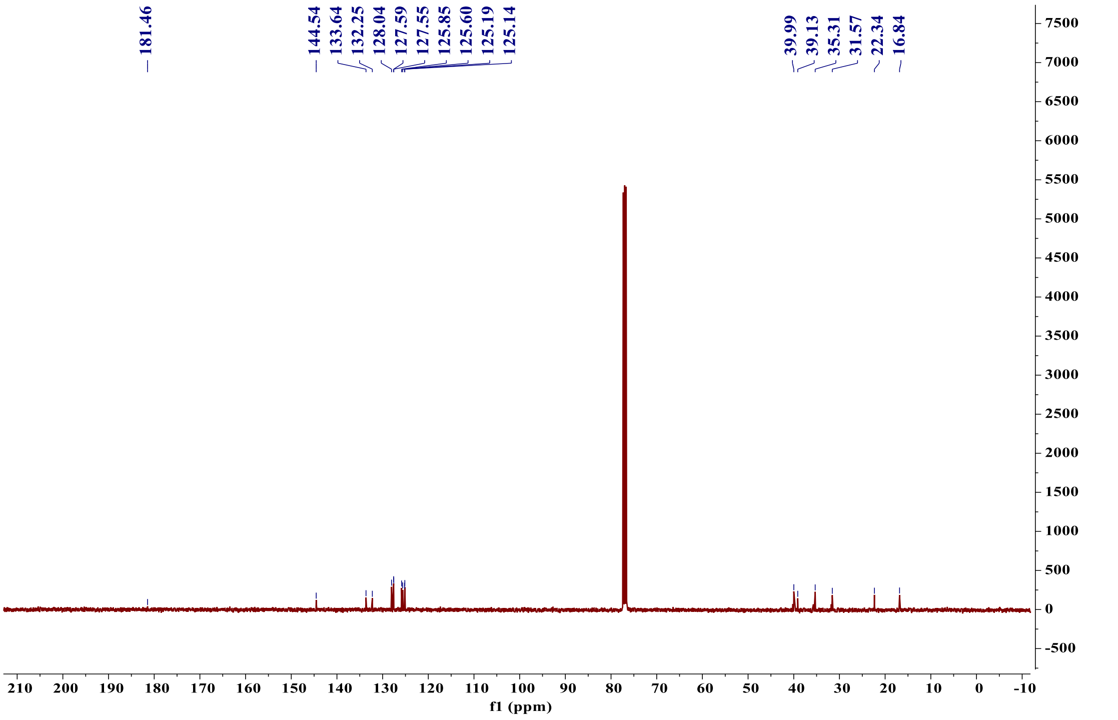

^{13}C NMR (101 MHz, CDCl3) spectrum of **(*S*,*S*)-22**

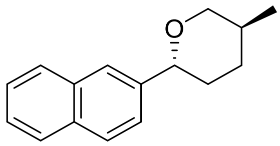

(*R*,*S*)-23

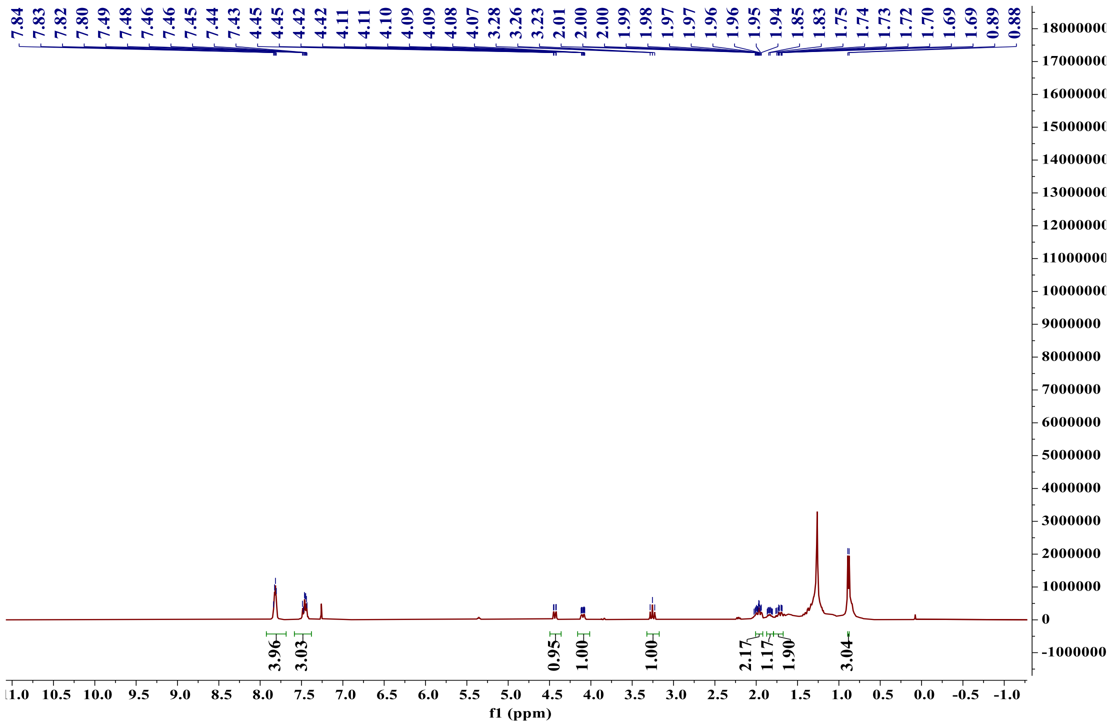

^{1}H NMR (400 MHz, $CDCl_3$) spectrum of **(*R*,*S*)-23**

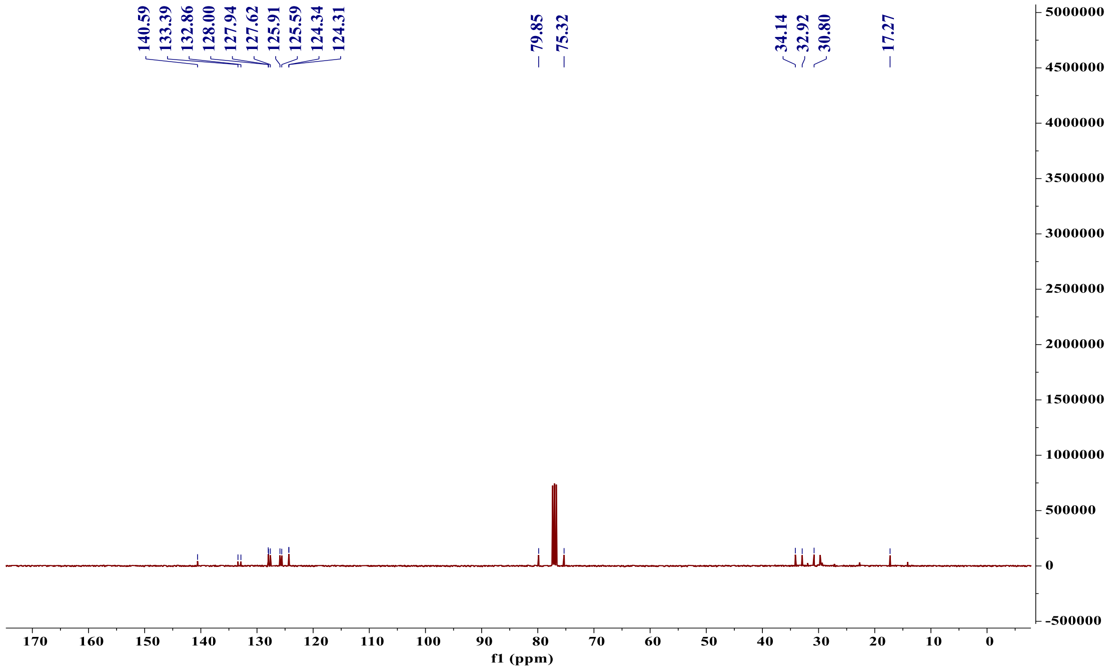

^{13}C NMR (101 MHz, $CDCl_3$) spectrum of **(*R*,*S*)-23**

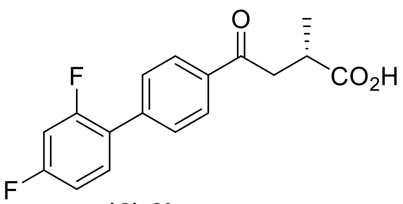

(*S*)-2h

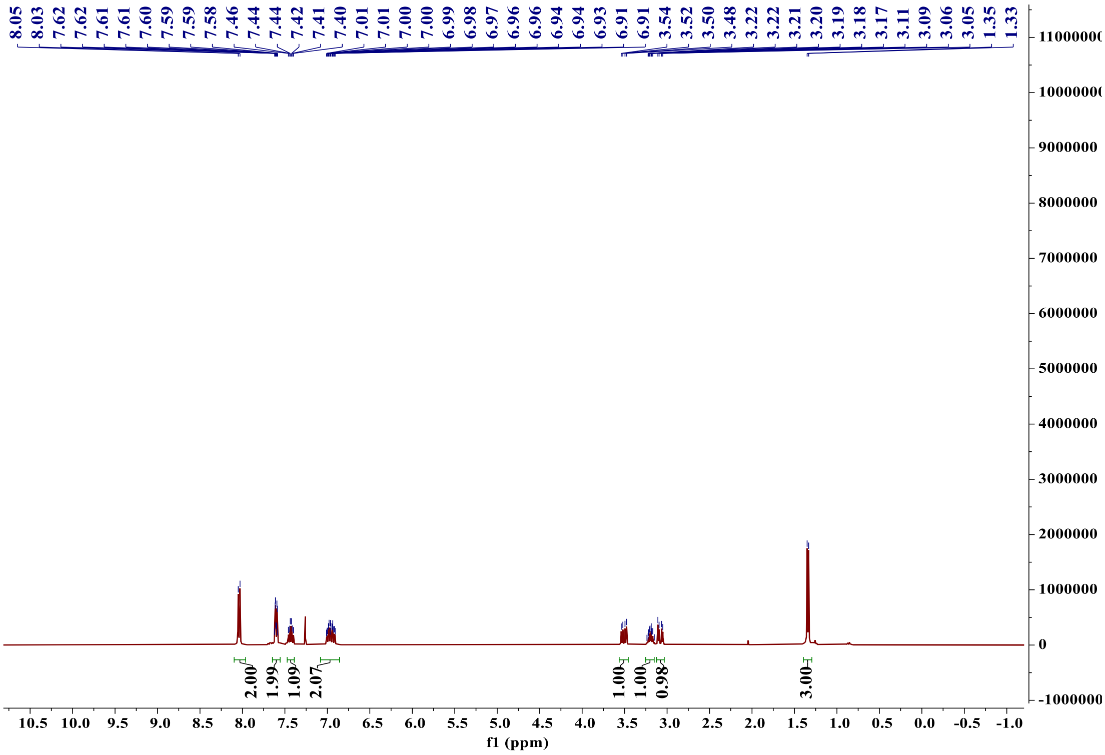

^{1}H NMR (400 MHz, $CDCl_3$) spectrum of **(*S*)-2h**

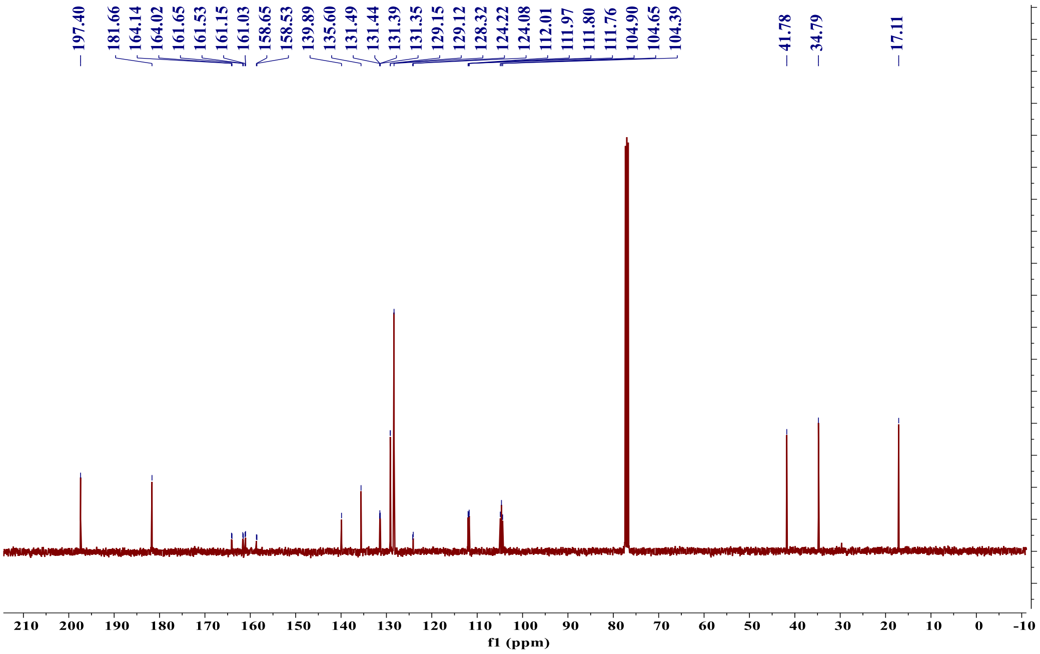

^{13}C NMR (101 MHz, $CDCl_3$) spectrum of **(*S*)-2h**

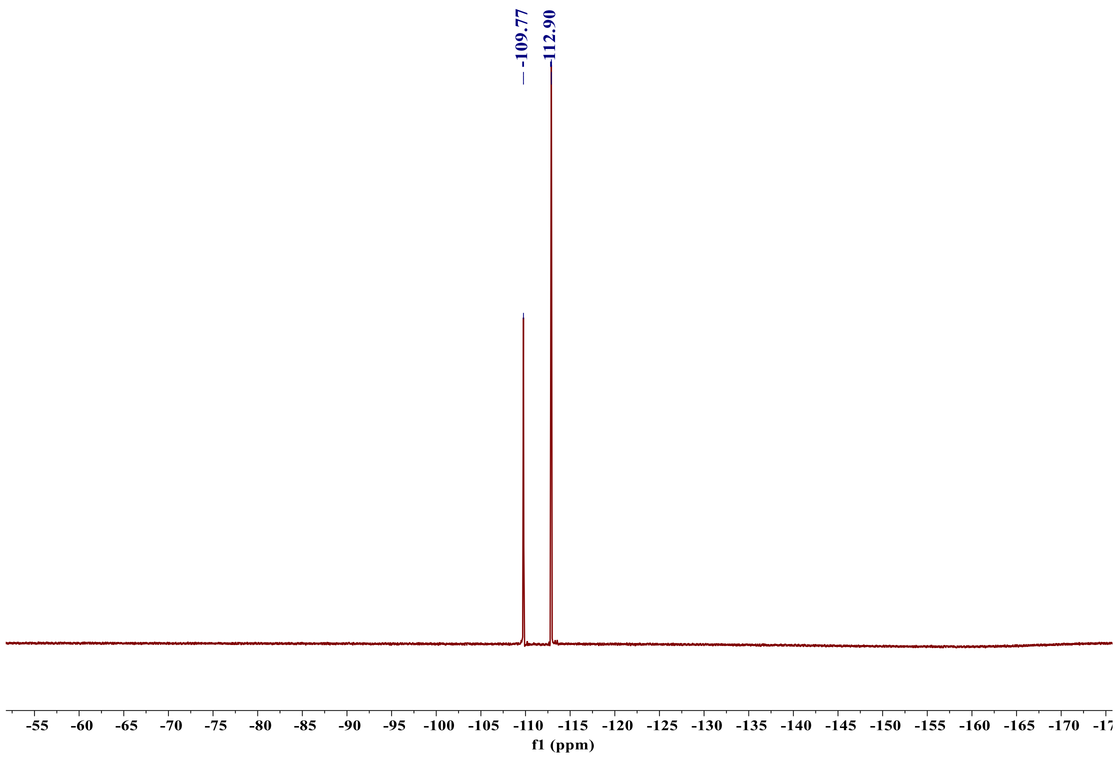

^{19}F NMR (377 MHz, $CDCl_3$) spectrum of **(*S*)-2h**

8. HPLC Data

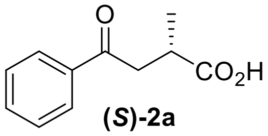

(S)-2a

HPLC conditions: Chiralpak AD Column, *n*-hexane/*i*-PrOH = 97/3, 210 nm, 0.8 mL/min, t_{major} = 39.254 min, t_{minor} = 35.401 min.

Racemate

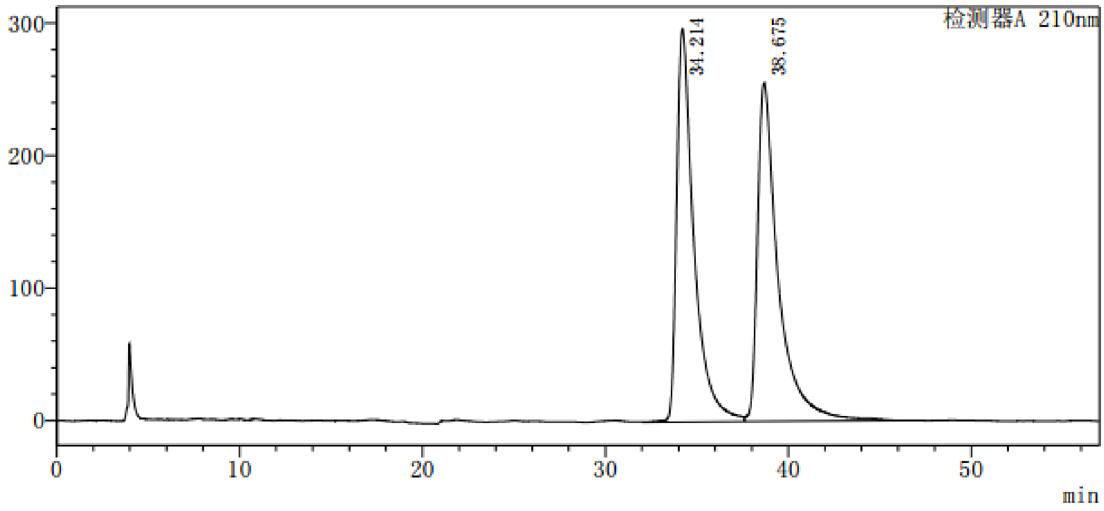

Chiral

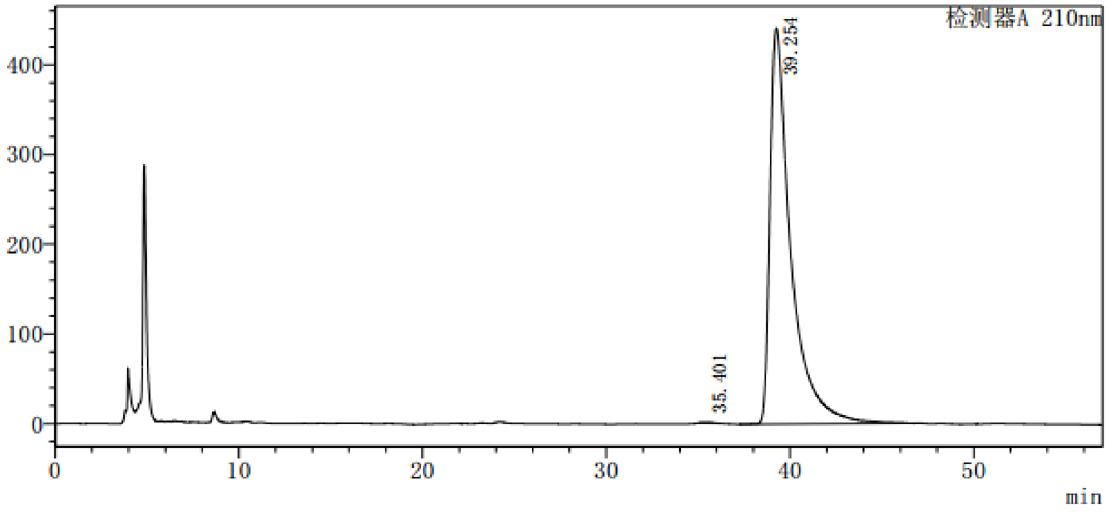

	Retention Time (min)	Area (%)	
**Peak 1**	35.401	0.123	>99% ee
**Peak 2**	39.254	99.877	

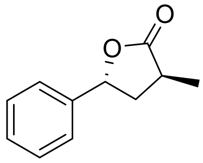

(*S*,*R*)-3a

HPLC conditions: Chiralcel OD Column, *n*-hexane/*i*-PrOH = 98/2, 210 nm, 0.6 mL/min, t_{major} = 28.580 min, t_{minor} = 27.232 min.

Racemate

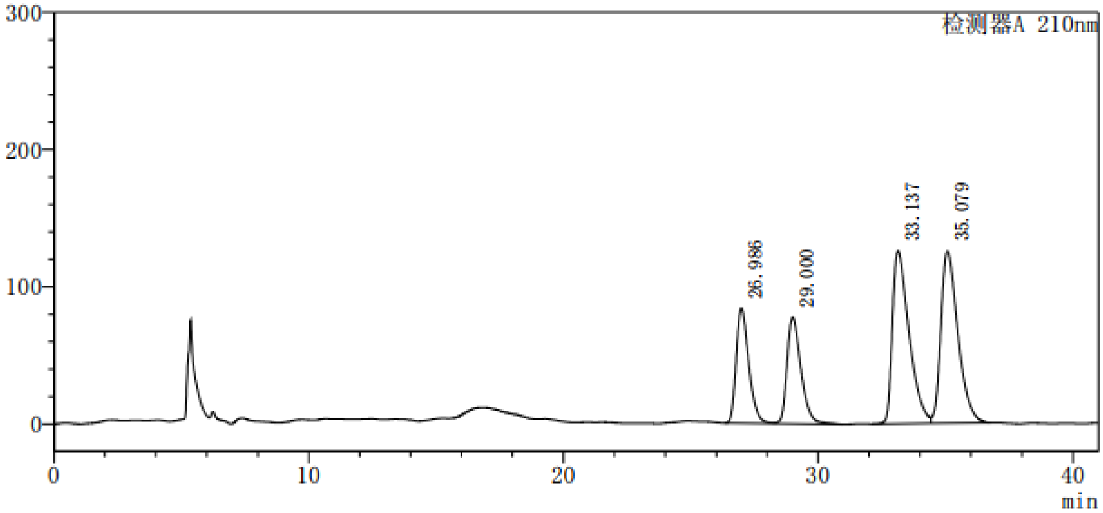

Chiral

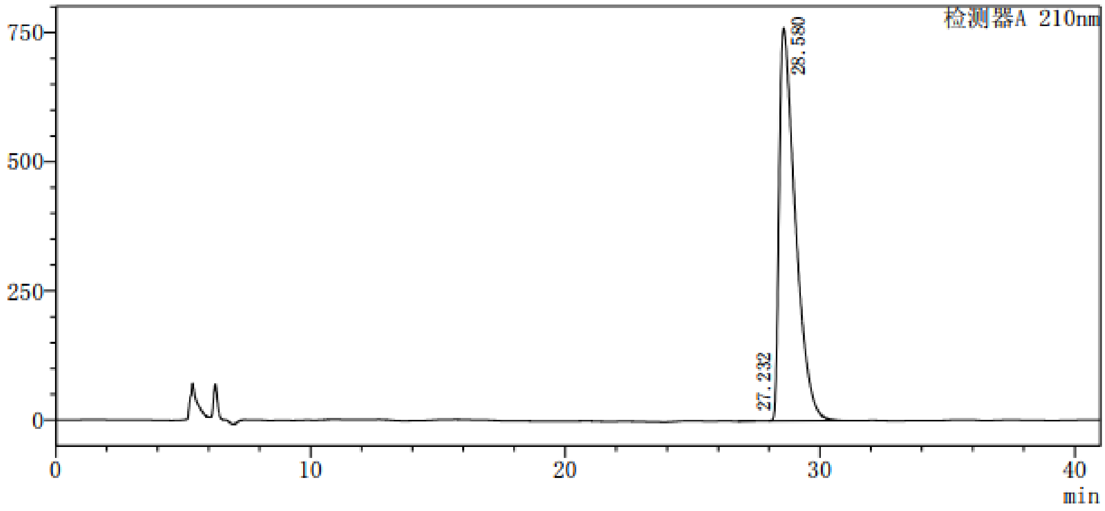

	Retention Time (min)	Area (%)	
**Peak 1**	27.232	0.015	>99% ee
**Peak 2**	28.580	99.985	

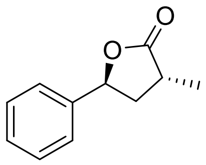

(*R*,*S*)-3a

HPLC conditions: Chiralcel OD Column, *n*-hexane/*i*-PrOH = 98/2, 210 nm, 0.6 mL/min, t_{major} = 26.110 min, t_{minor} = 28.263 min.

Racemate

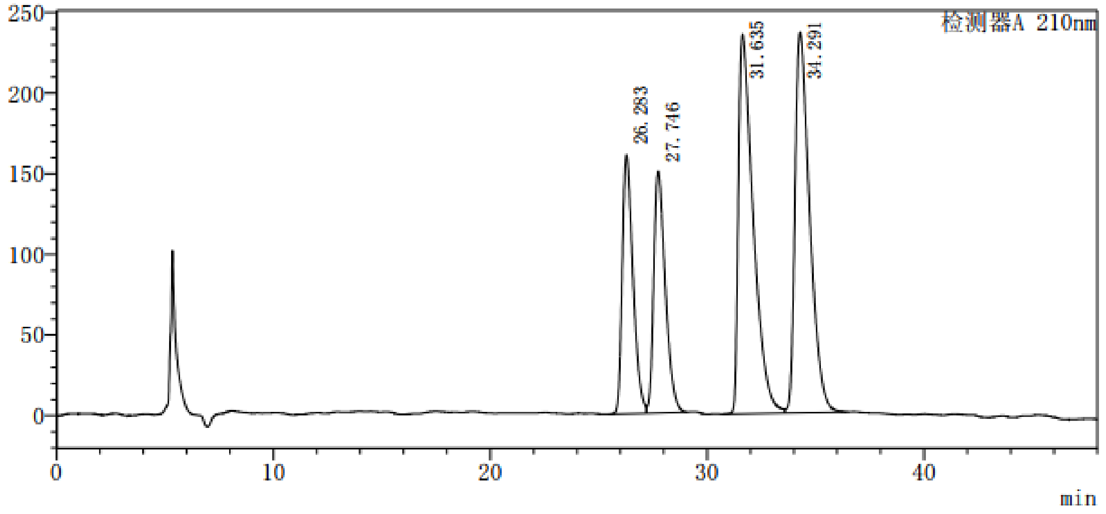

Chiral

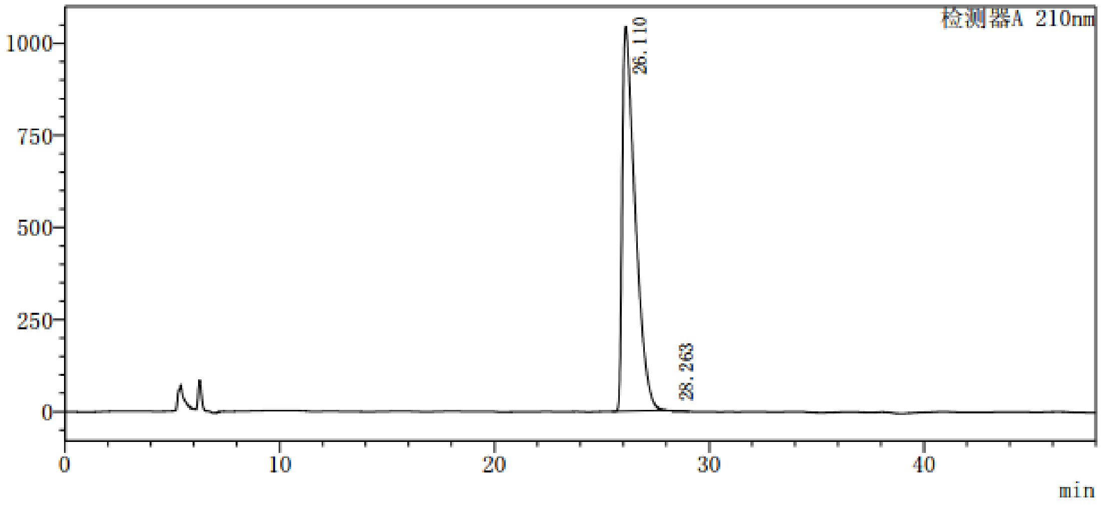

	Retention Time (min)	Area (%)	
**Peak 1**	26.110	99.860	>99% ee
**Peak 2**	28.263	0.140	

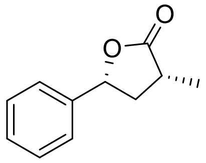

(*R*,*R*)-3a

HPLC conditions: Chiralcel OD Column, *n*-hexane/*i*-PrOH = 98/2, 210 nm, 0.6 mL/min, t_{major} = 31.661 min, t_{minor} = 34.904 min.

Racemate

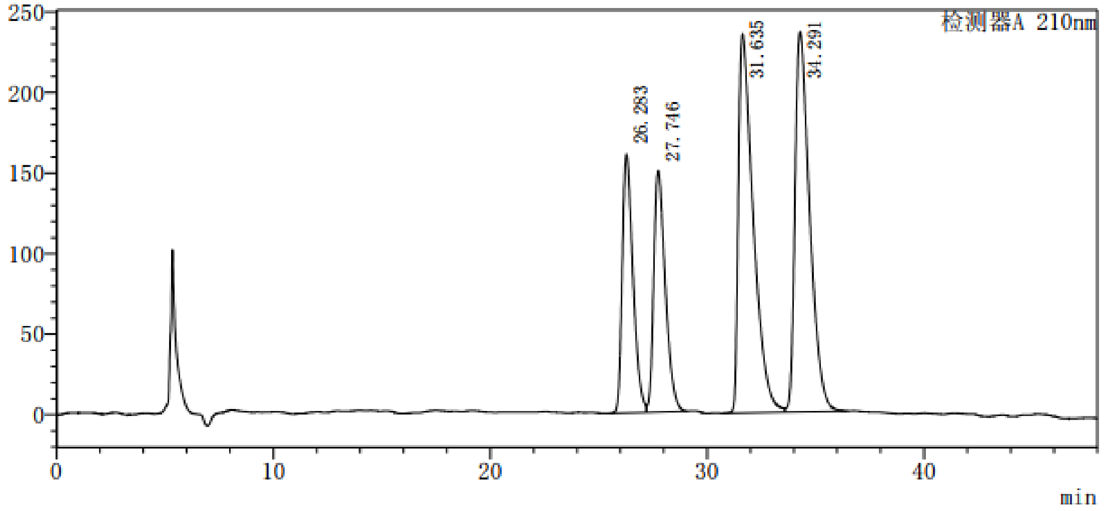

Chiral

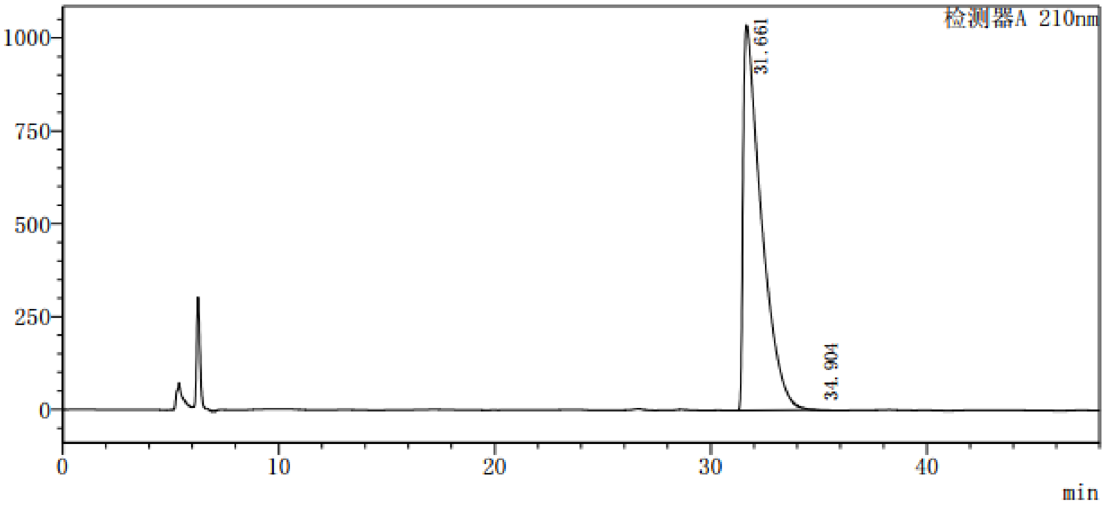

	Retention Time (min)	Area (%)	
**Peak 1**	31.661	99.965	>99% ee
**Peak 2**	34.904	0.035	

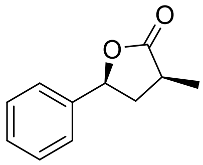

(*S*,*S*)-3a

HPLC conditions: Chiralcel OD Column, *n*-hexane/*i*-PrOH = 98/2, 210 nm, 0.6 mL/min, t_{major} = 33.807 min, t_{minor} = 32.796 min.

Racemate

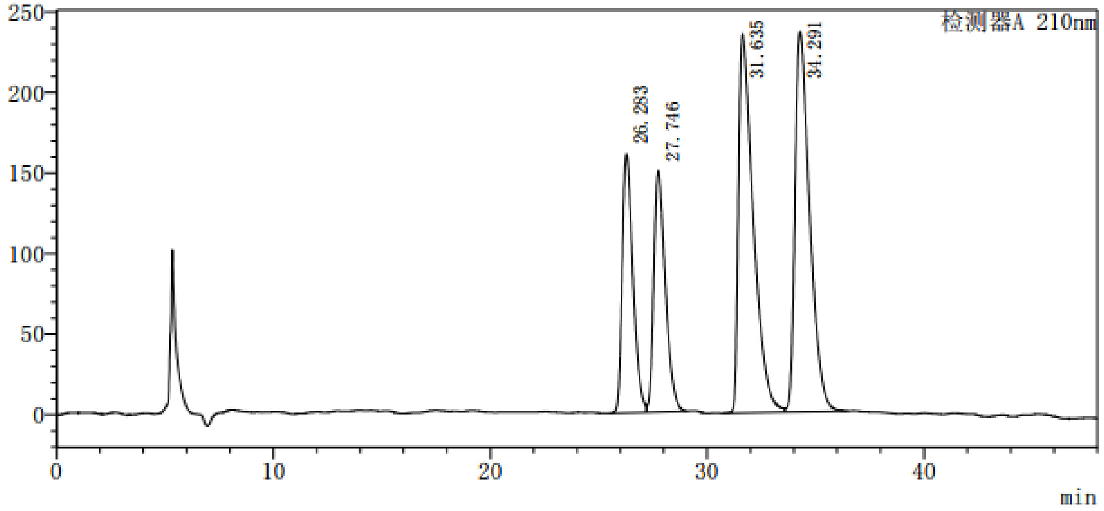

Chiral

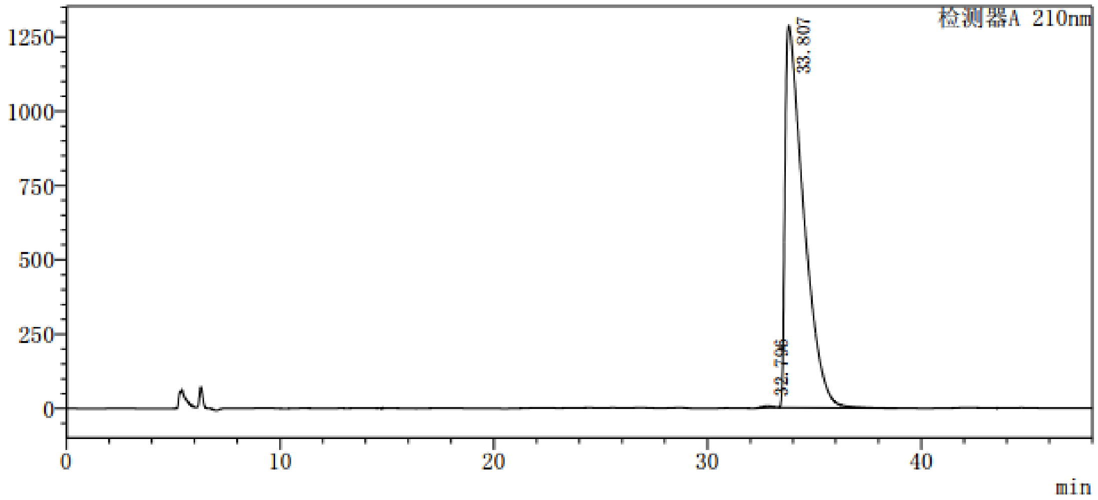

	Retention Time (min)	Area (%)	
**Peak 1**	32.796	0.250	>99% ee
**Peak 2**	33.807	99.750	

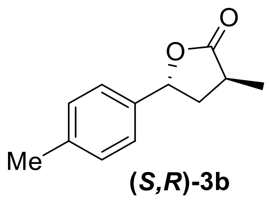

(*S*,*R*)-3b

HPLC conditions: Chiralcel OZ Column, *n*-hexane/*i*-PrOH = 98/2, 210 nm, 0.6 mL/min, t_{major} = 29.784 min, t_{minor} = 26.568 min.

Racemate

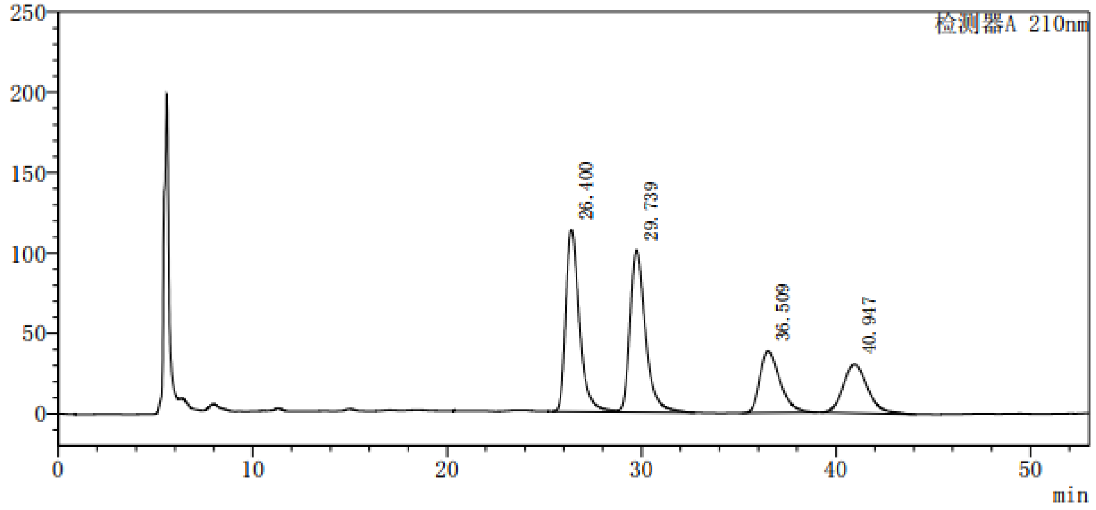

Chiral

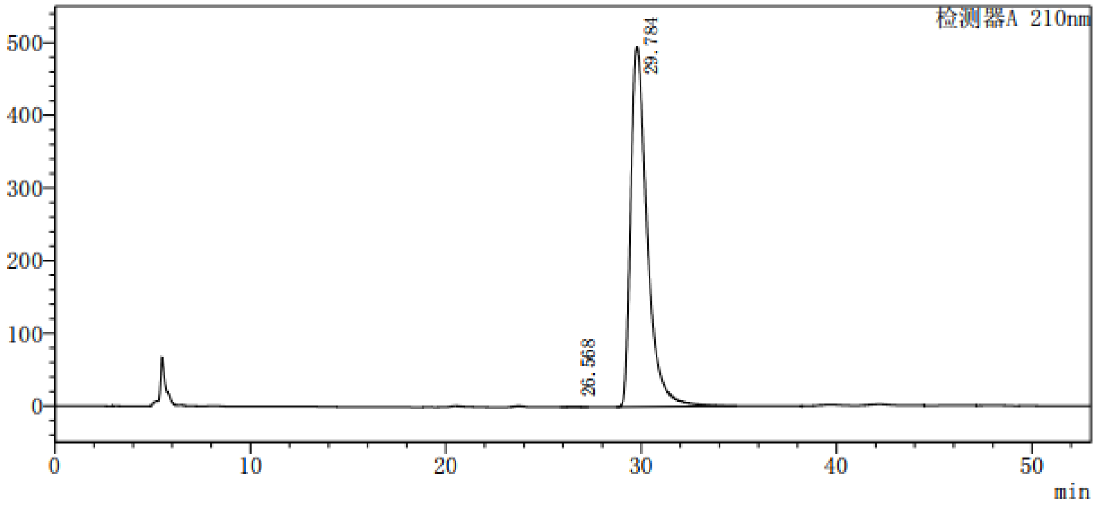

	Retention Time (min)	Area (%)	
**Peak 1**	26.568	0.086	>99% ee
**Peak 2**	29.784	99.914	

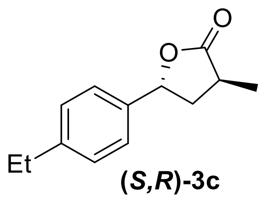
(*S*,*R*)-3c

HPLC conditions: Chiralcel OD Column, *n*-hexane/*i*-PrOH = 98/2, 210 nm, 0.6 mL/min, t_{major} = 16.586 min, t_{minor} = 19.258 min.

Racemate

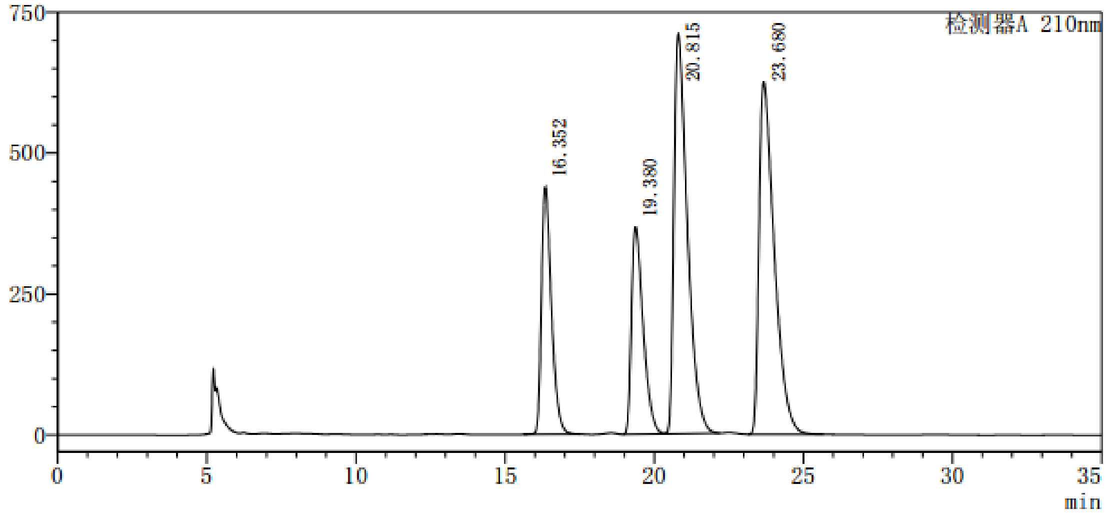

Chiral

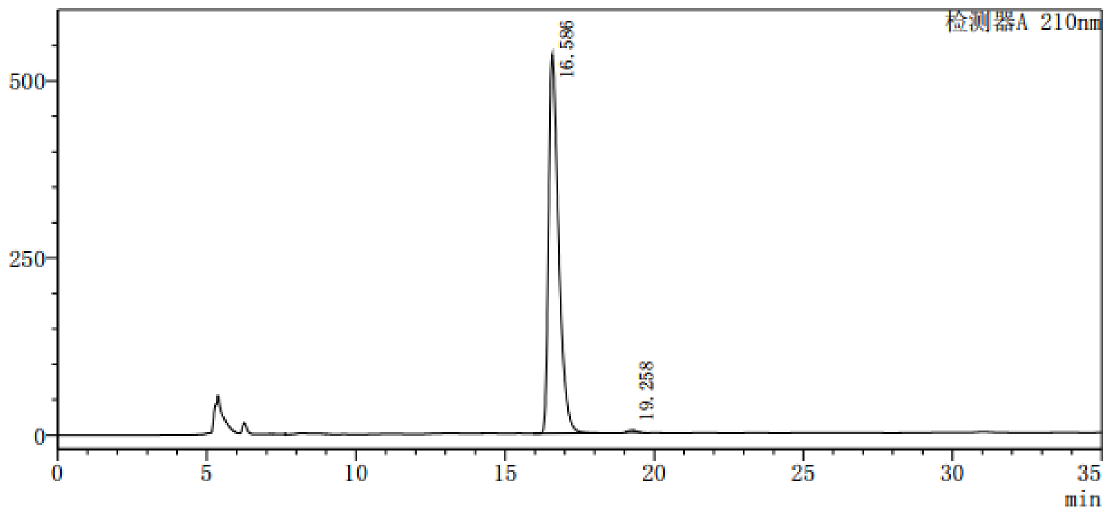

	Retention Time (min)	Area (%)	
**Peak 1**	16.586	99.913	>99% ee
**Peak 2**	19.258	0.087	

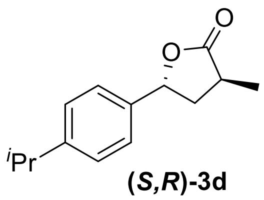

(*S*,*R*)-3d

HPLC conditions: Chiralcel OZ Column, *n*-hexane/*i*-PrOH = 98/2, 210 nm, 0.6 mL/min, t_{major} = 23.237 min, t_{minor} = 19.879 min.

Racemate

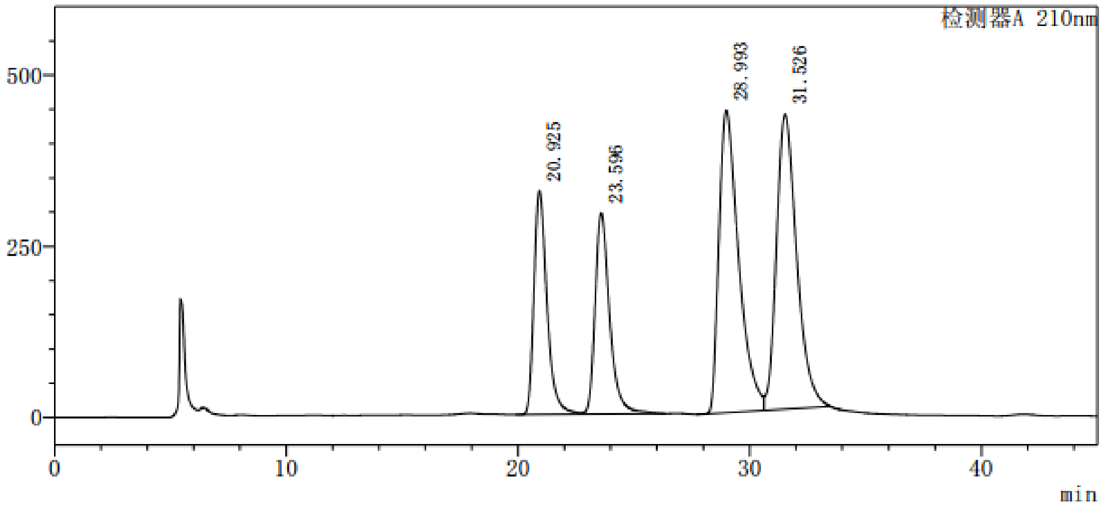

Chiral

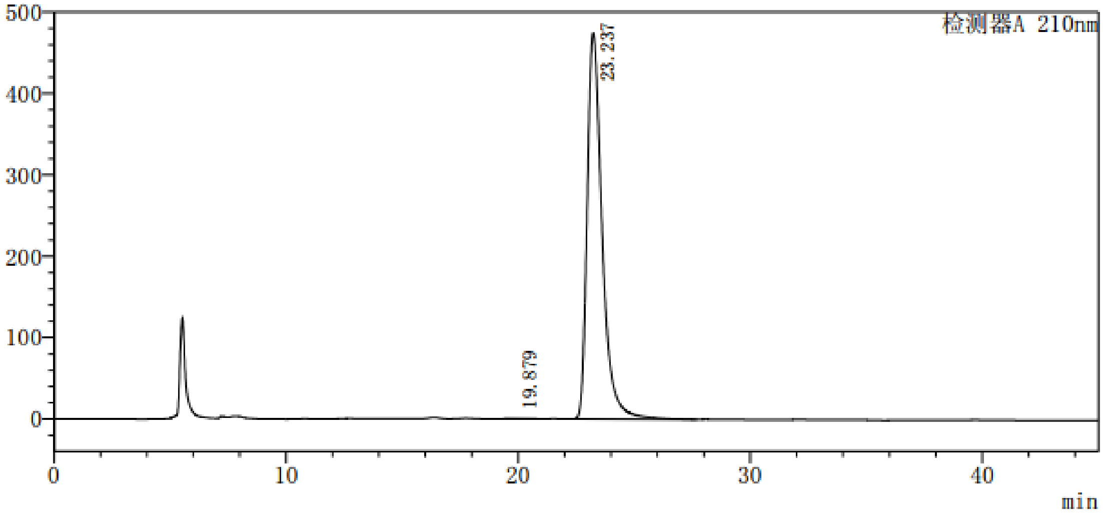

	Retention Time (min)	Area (%)	
**Peak 1**	19.879	0.045	>99% ee
**Peak 2**	23.237	99.955	

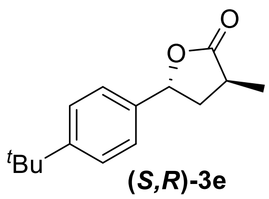

(*S*,*R*)-3e

HPLC conditions: Chiralcel OD Column, *n*-hexane/*i*-PrOH = 98/2, 210 nm, 0.6 mL/min, t_{major} = 14.896 min, t_{minor} = 15.804 min.

Racemate

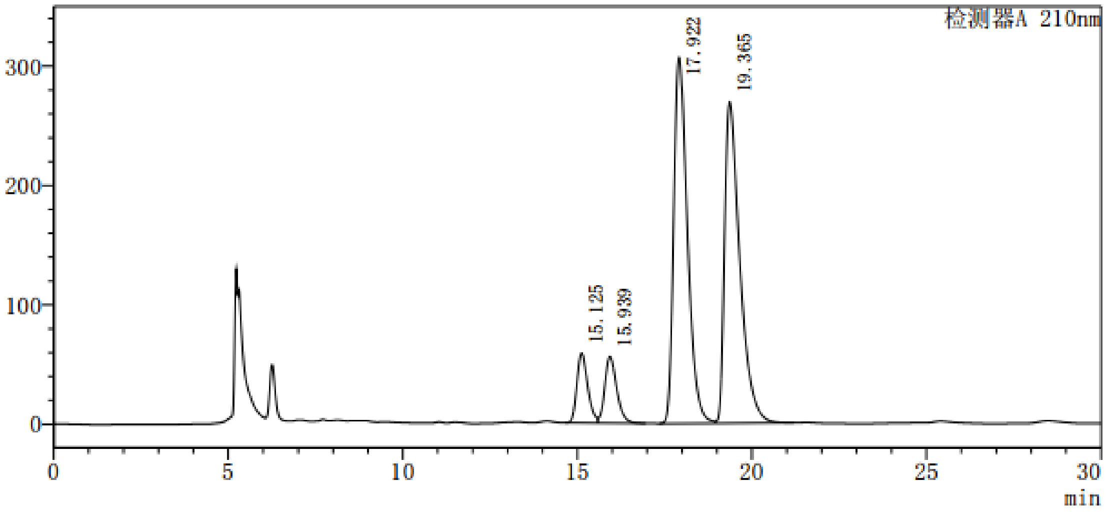

Chiral

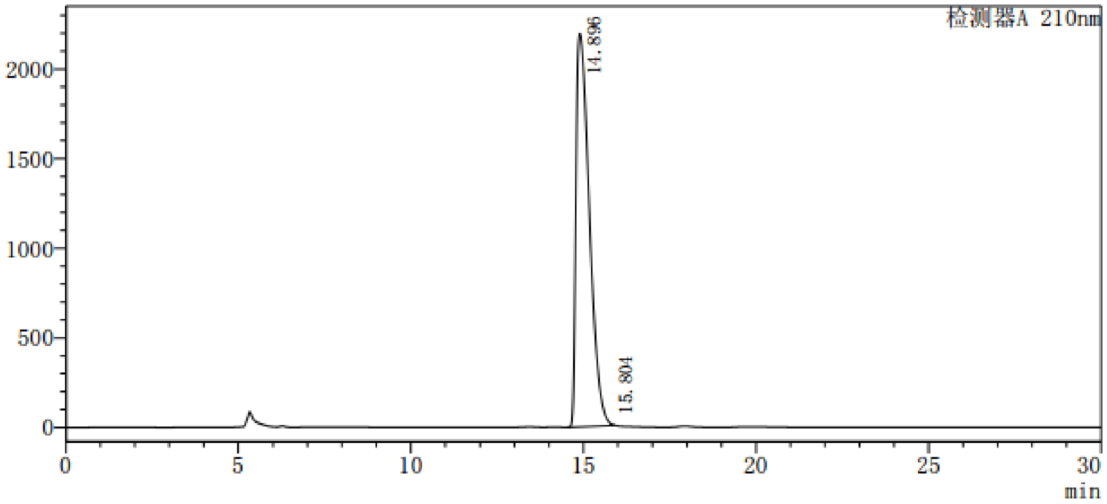

	Retention Time (min)	Area (%)	
**Peak 1**	14.896	99.951	>99% ee
**Peak 2**	15.804	0.049	

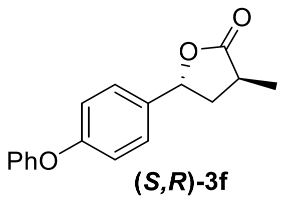

(*S*,*R*)-3f

HPLC conditions: Chiralcel OD Column, *n*-hexane/*i*-PrOH = 98/2, 210 nm, 0.6 mL/min, t_{major} = 39.095 min, t_{minor} = 54.683 min.

Racemate

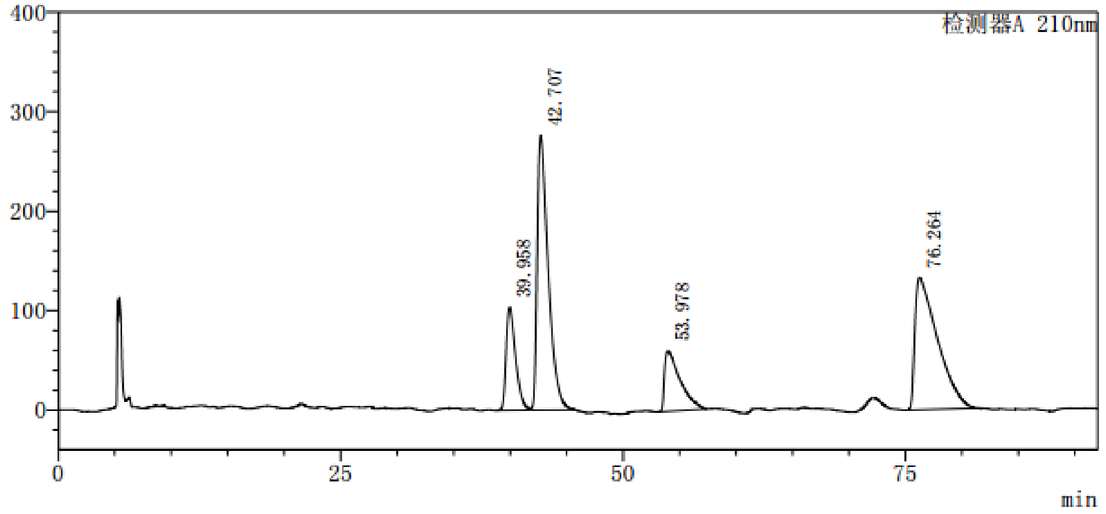

Chiral

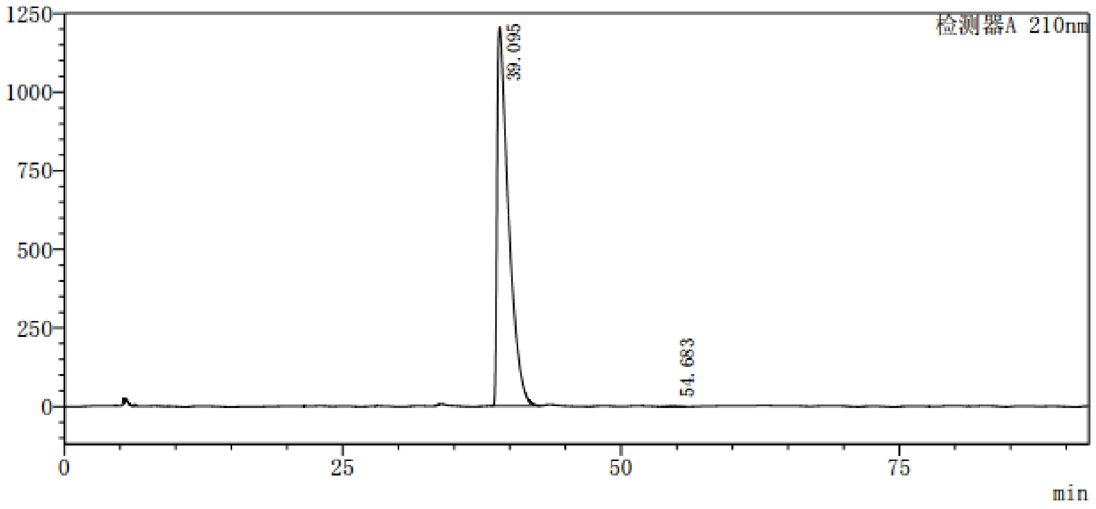

	Retention Time (min)	Area (%)	
**Peak 1**	39.095	99.923	>99% ee
**Peak 2**	54.683	0.077	

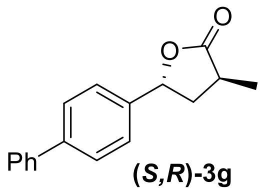

(*S*,*R*)-3g

HPLC conditions: Chiralcel OD Column, *n*-hexane/*i*-PrOH = 96/4, 210 nm, 1.0 mL/min, t_{major} = 15.573 min, t_{minor} = 23.541 min.

Racemate

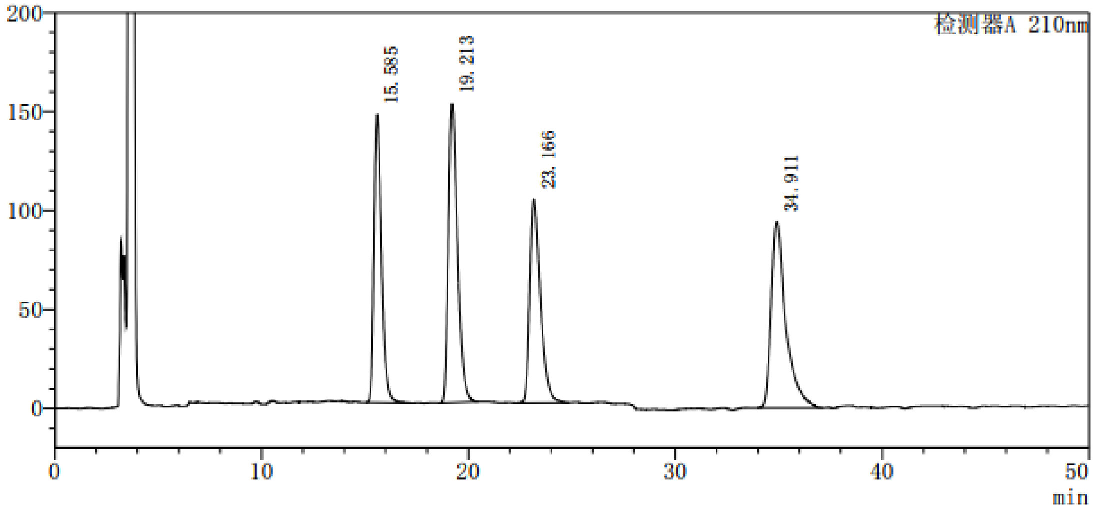

Chiral

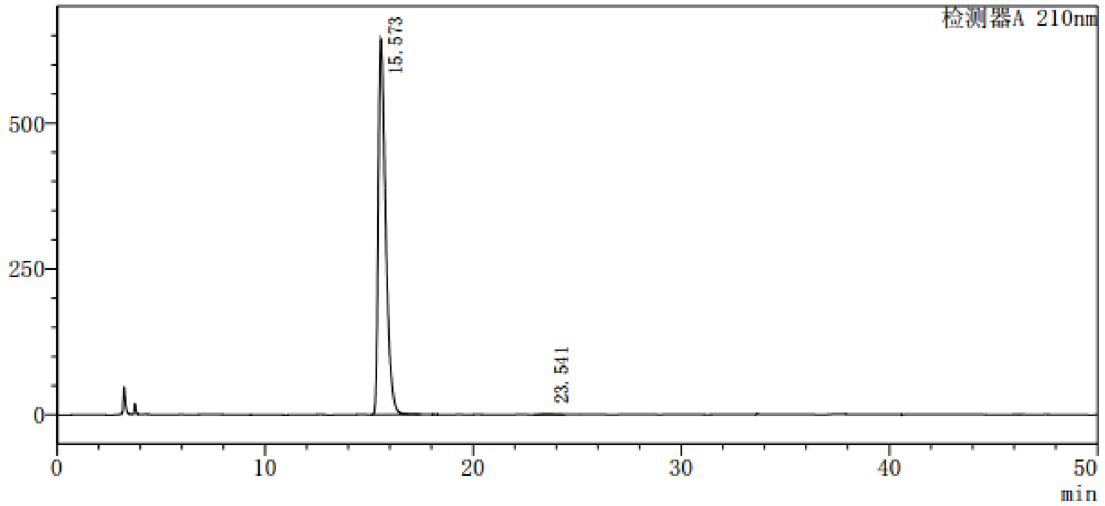

	Retention Time (min)	Area (%)	
**Peak 1**	15.573	99.770	>99% ee
**Peak 2**	23.541	0.230	

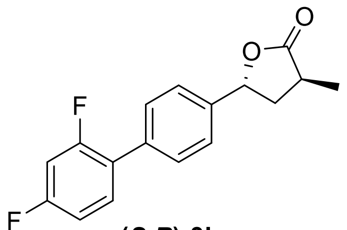

(*S*,*R*)-3h

HPLC conditions: Chiralpak AS Column, *n*-hexane/*i*-PrOH = 93/7, 254 nm, 1.0 mL/min, t_{major} = 20.541 min, t_{minor} = 29.204 min.

Racemate

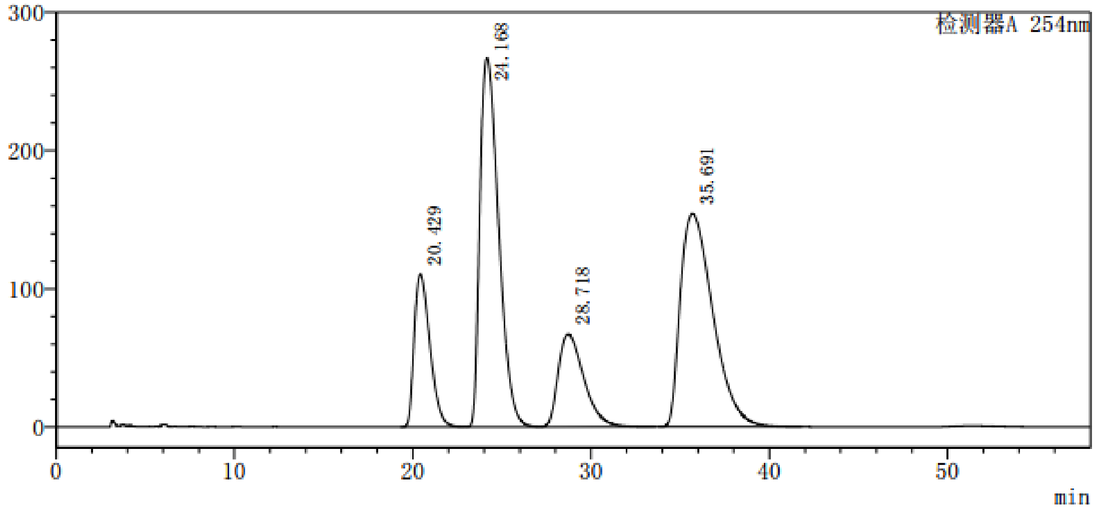

Chiral

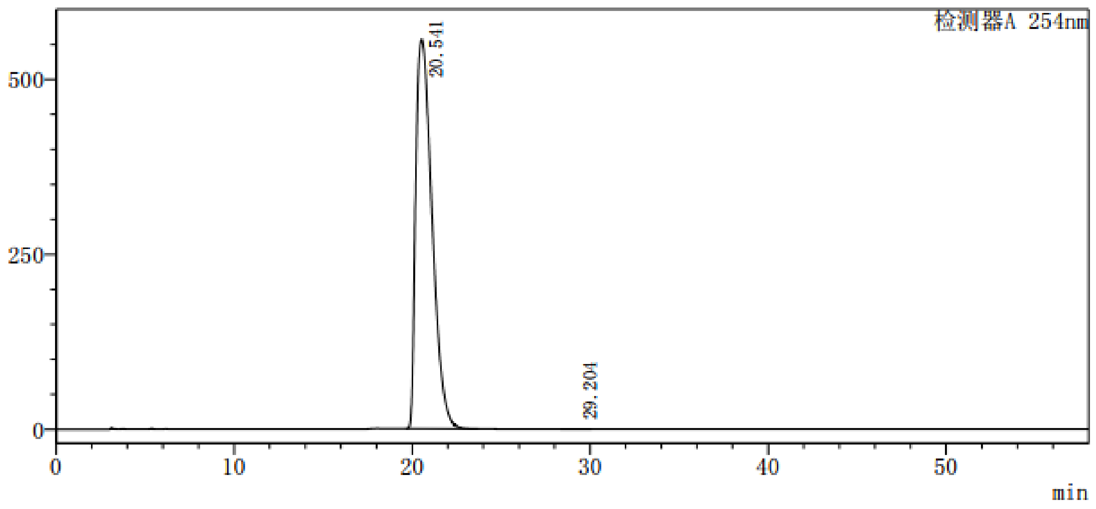

	Retention Time (min)	Area (%)	
**Peak 1**	20.541	99.971	>99% ee
**Peak 2**	29.204	0.029	

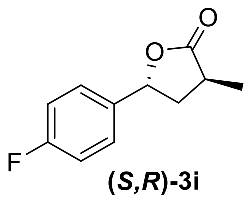

(*S*,*R*)-3i

HPLC conditions: Chiralcel OD Column, *n*-hexane/*i*-PrOH = 98/2, 210 nm, 0.6 mL/min, t_{major} = 20.418 min, t_{minor} = 23.853 min.

Racemate

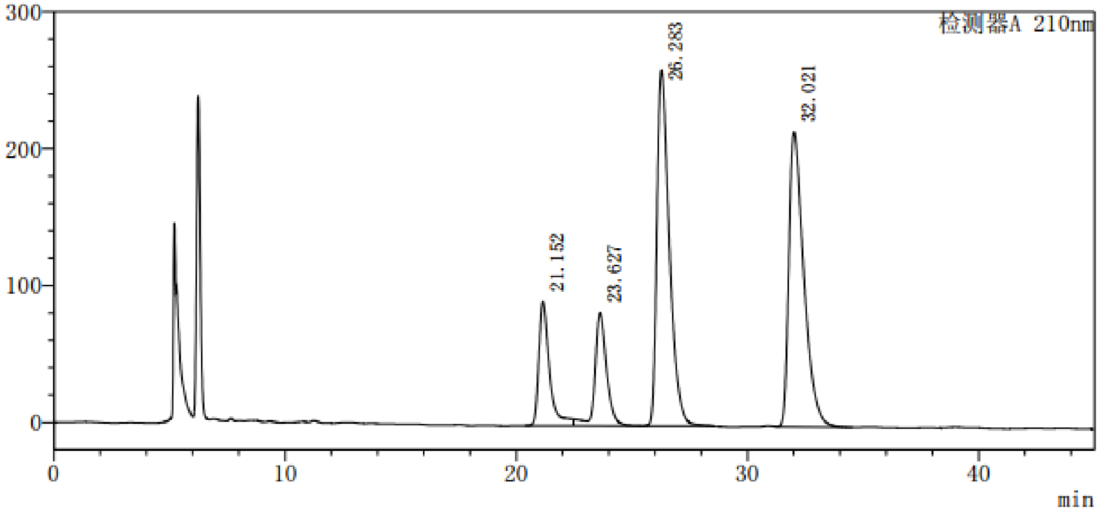

Chiral

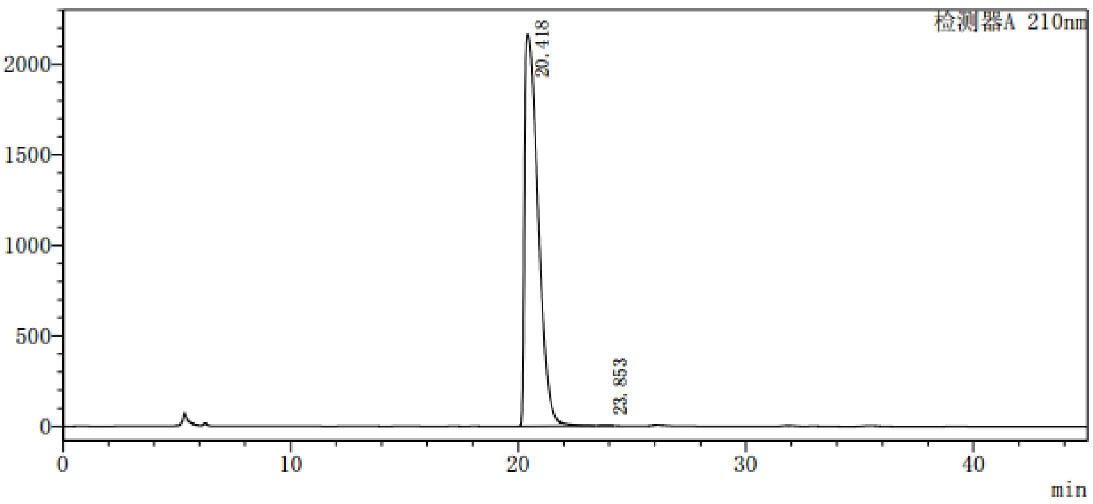

	Retention Time (min)	Area (%)	
**Peak 1**	20.418	99.950	>99% ee
**Peak 2**	23.853	0.050	

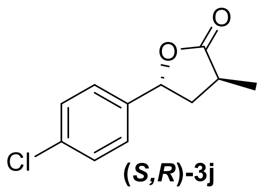

(*S*,*R*)-3j

HPLC conditions: Chiralcel OD Column, *n*-hexane/*i*-PrOH = 98/2, 210 nm, 0.6 mL/min, t_{major} = 21.979 min, t_{minor} = 24.438 min.

Racemat

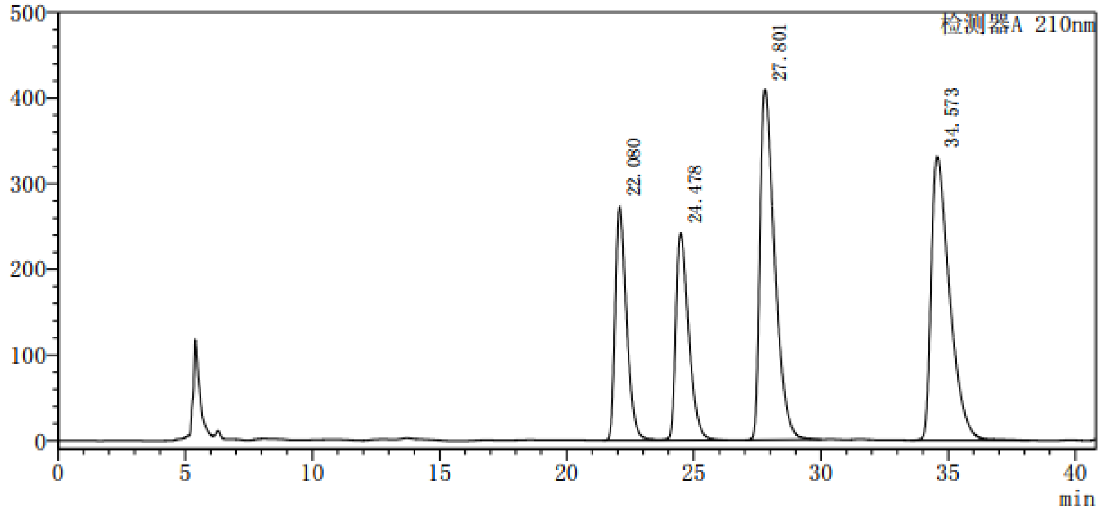

Chiral

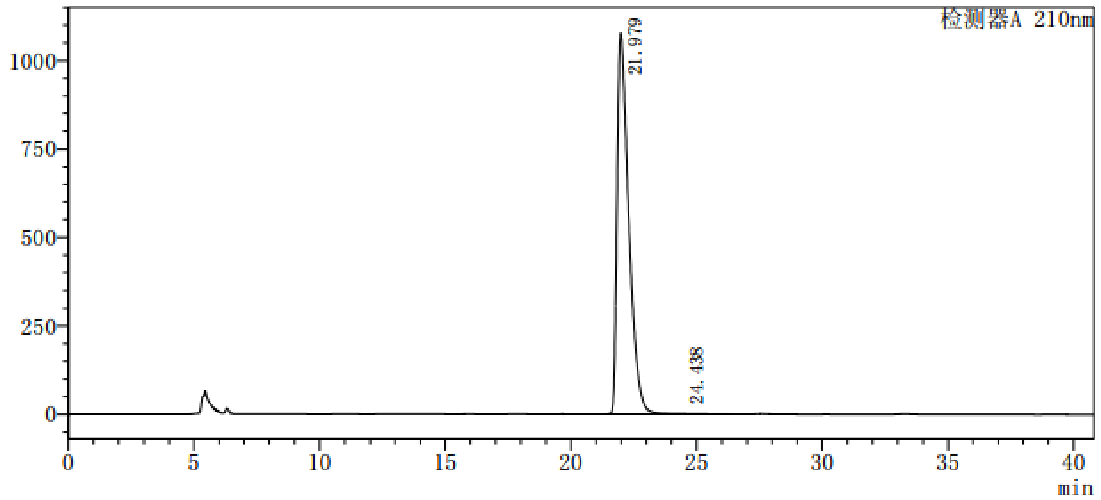

	Retention Time (min)	Area (%)	
**Peak 1**	21.979	99.792	>99% ee
**Peak 2**	24.438	0.208	

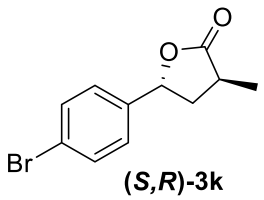

(*S*,*R*)-3k

HPLC conditions: Chiralcel OD Column, *n*-hexane/*i*-PrOH = 98/2, 210 nm, 0.6 mL/min, t_{major} = 24.124 min, t_{minor} = 26.720 min.

Racemate

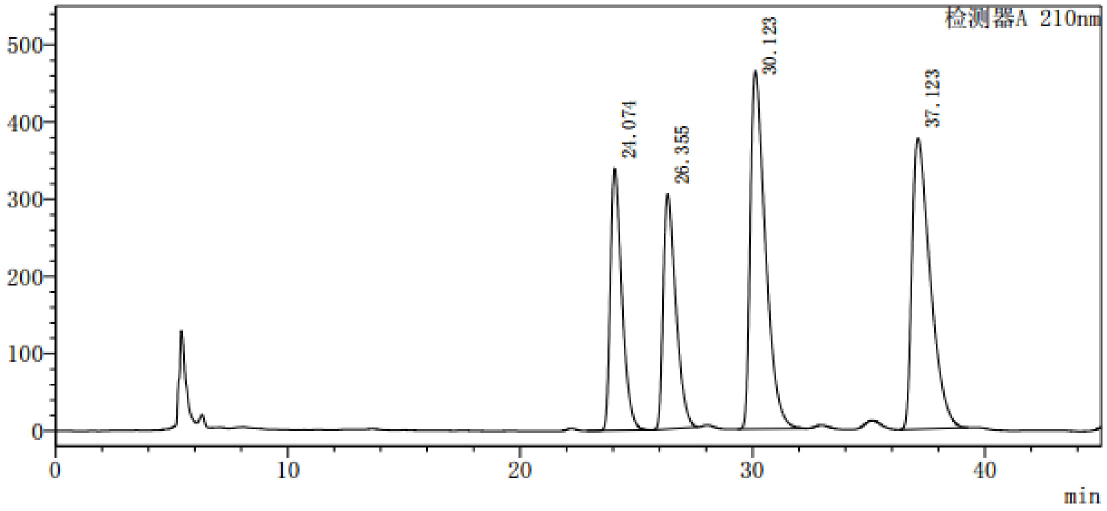

Chiral

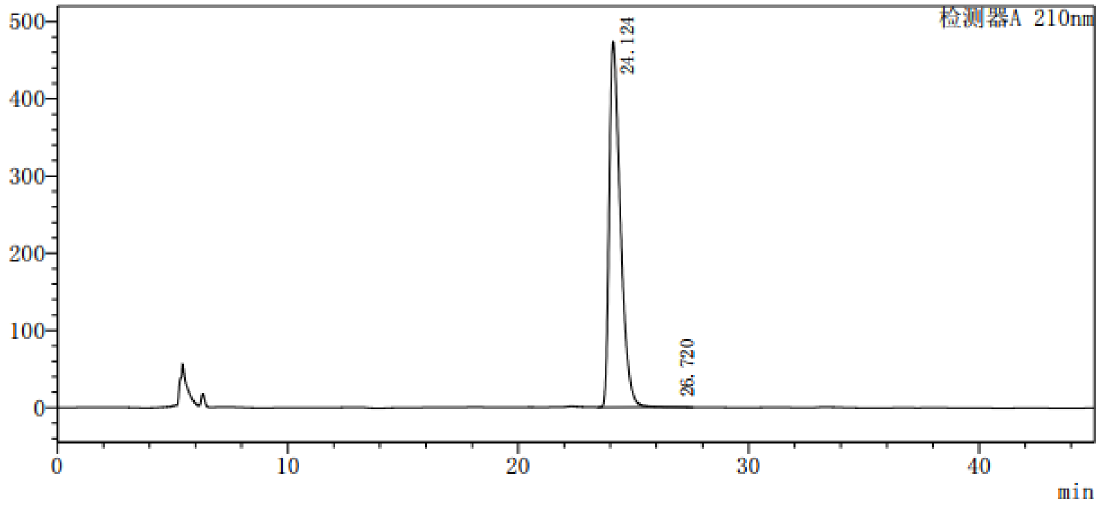

	Retention Time (min)	Area (%)	
**Peak 1**	24.124	99.945	>99% ee
**Peak 2**	26.720	0.055	

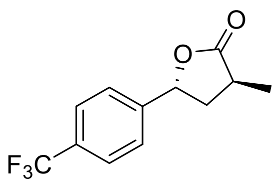

(*S*,*R*)-3l

HPLC conditions: Chiralcel OD-OZ Column, *n*-hexane/*i*-PrOH = 98/2, 210 nm, 0.7 mL/min, t_{major} = 60.647 min, t_{minor} = 58.477 min.

Racemate

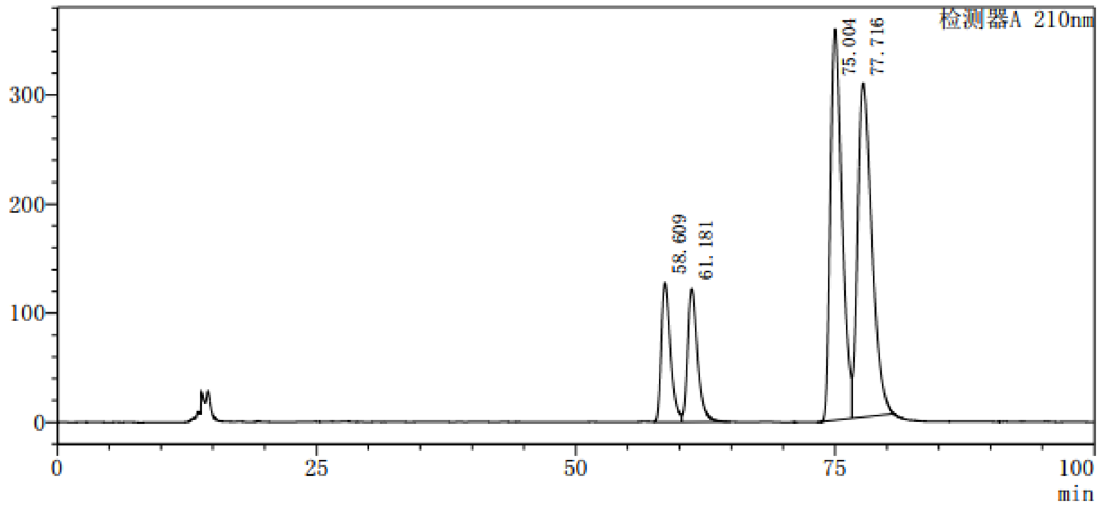

Chiral

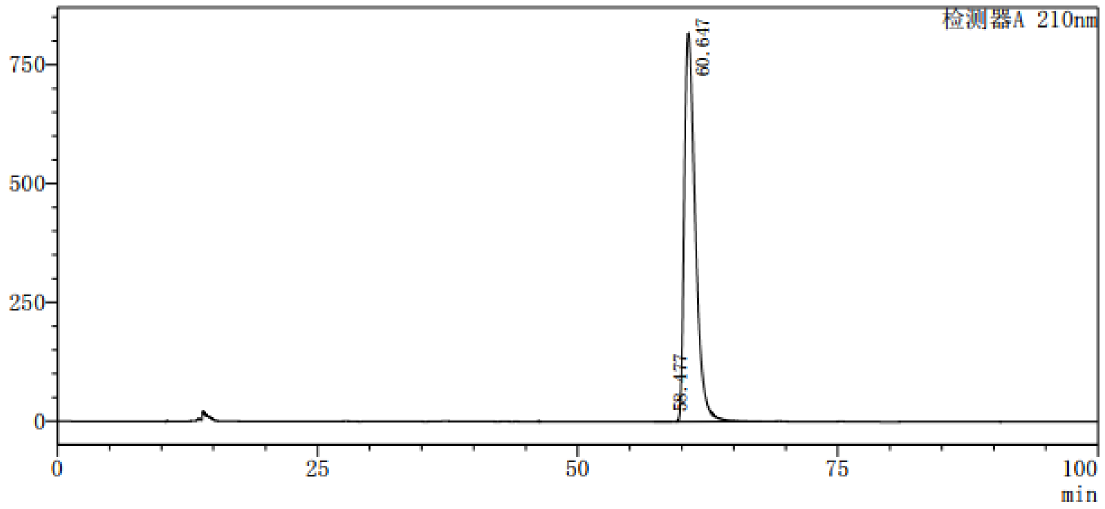

	Retention Time (min)	Area (%)	
**Peak 1**	58.477	0.024	>99% ee
**Peak 2**	60.647	99.976	

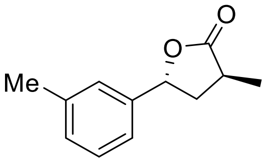

(*S*,*R*)-3m

HPLC conditions: Chiralcel OD Column, *n*-hexane/*i*-PrOH = 98/2, 210 nm, 0.6 mL/min, t_{major} = 20.120 min, t_{minor} = 23.108 min.

Racemate

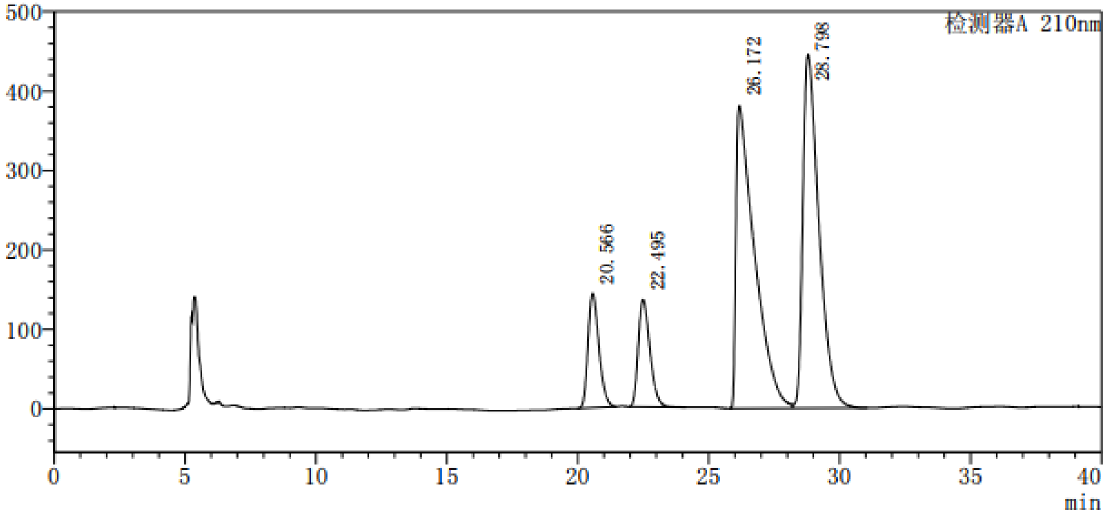

Chiral

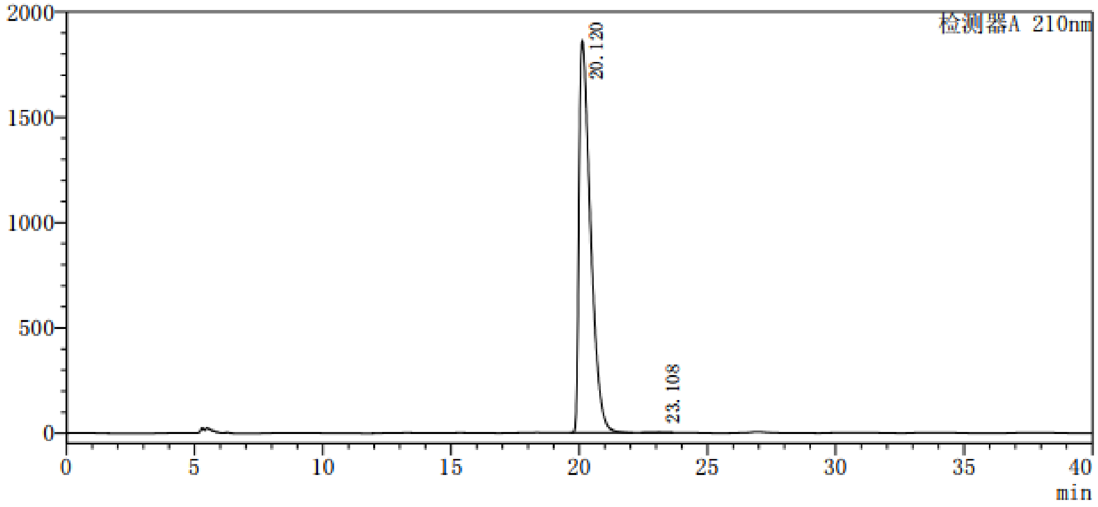

	Retention Time (min)	Area (%)	
**Peak 1**	20.120	99.914	>99% ee
**Peak 2**	23.108	0.086	

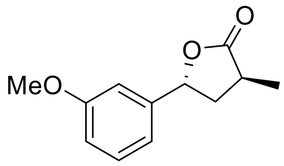

(*S*,*R*)-3n

HPLC conditions: Chiralcel OD and Chiralpak IC column, *n*-hexane/*i*-PrOH = 95/5, 210 nm, 1.0 mL/min, t_{major} = 62.212 min, t_{minor} = 59.408 min.

Racemate

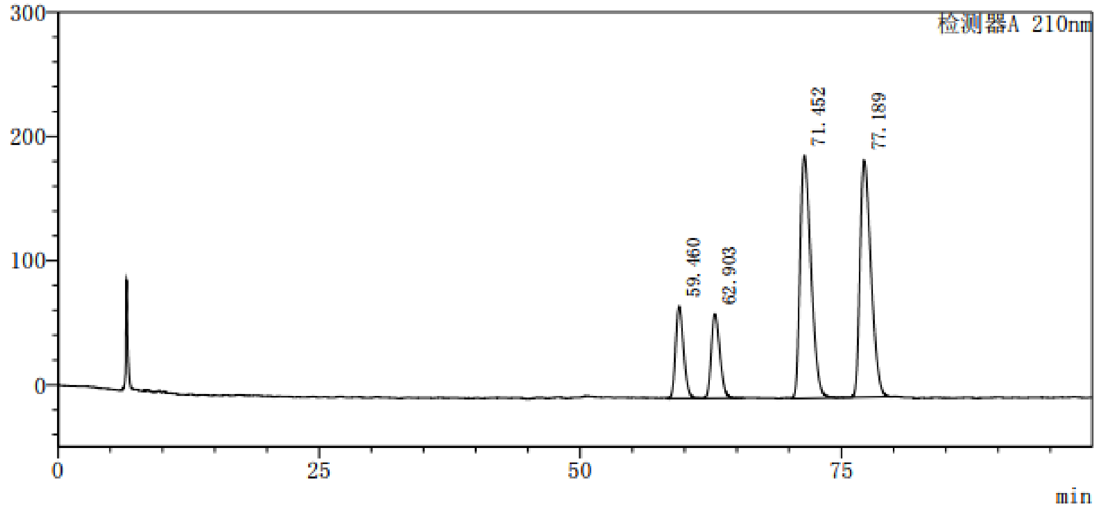

Chiral

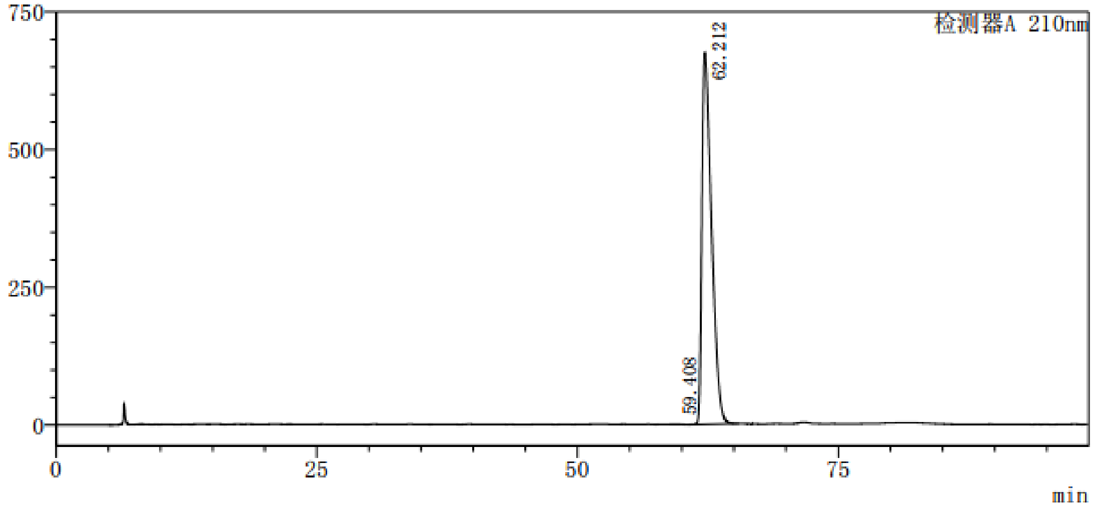

	Retention Time (min)	Area (%)	
**Peak 1**	59.408	0.014	>99% ee
**Peak 2**	62.212	99.986	

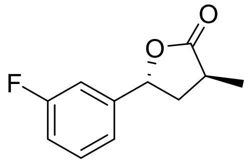

(*S*,*R*)-3o

HPLC conditions: Chiralcel OD and Chiralpak IC column, *n*-hexane/*i*-PrOH = 97/3, 210 nm, 0.7 mL/min, t_{major} = 58.560 min, t_{minor} = 56.484 min.

Racemate

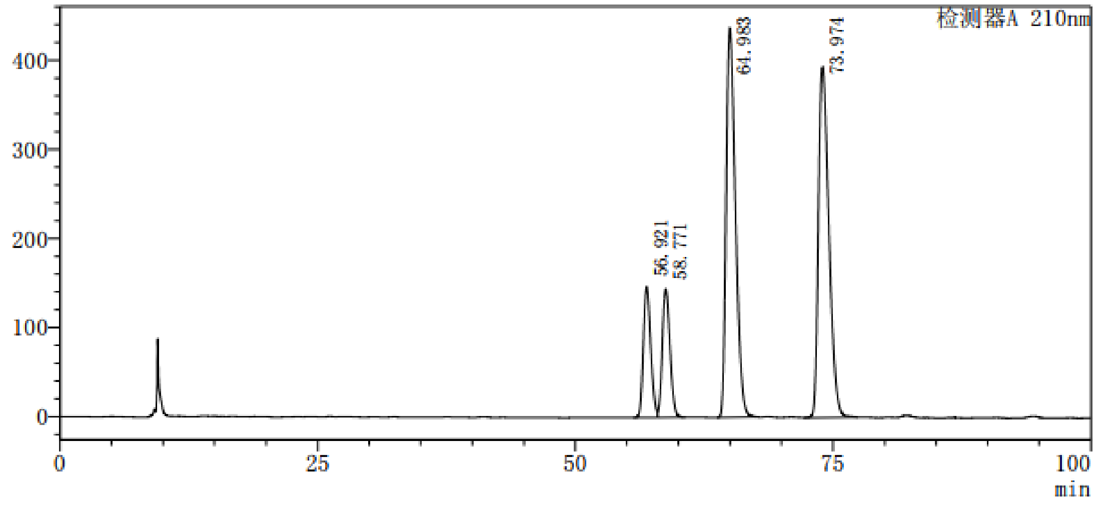

Chiral

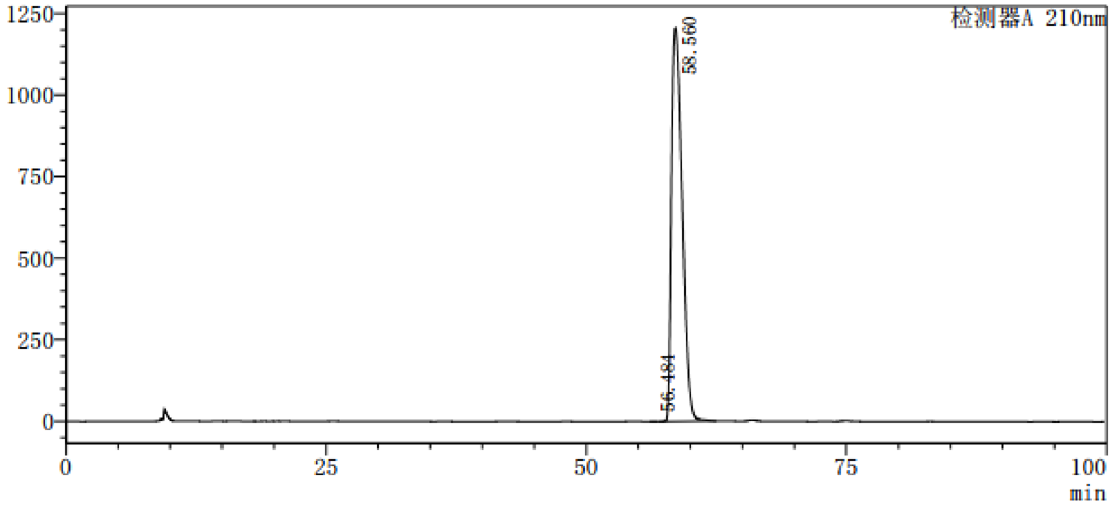

	Retention Time (min)	Area (%)	
**Peak 1**	56.484	0.002	>99% ee
**Peak 2**	58.560	99.998	

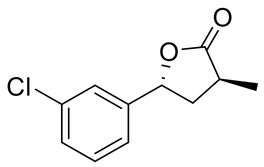

(*S*,*R*)-3p

HPLC conditions: Chiralcel OD Column, *n*-hexane/*i*-PrOH = 98/2, 210 nm, 0.6 mL/min, t_{major} = 27.614 min, t_{minor} = 25.138 min.

Racemate

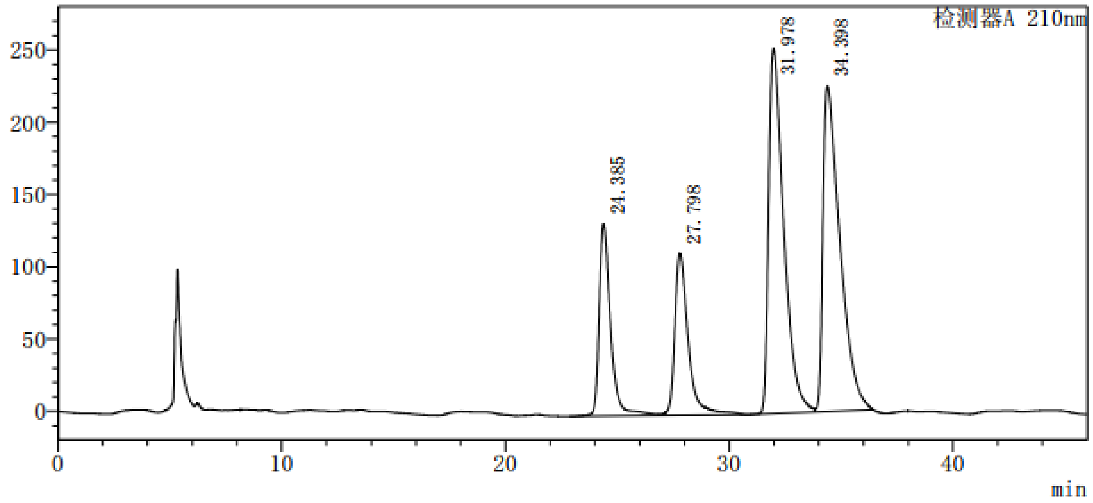

Chiral

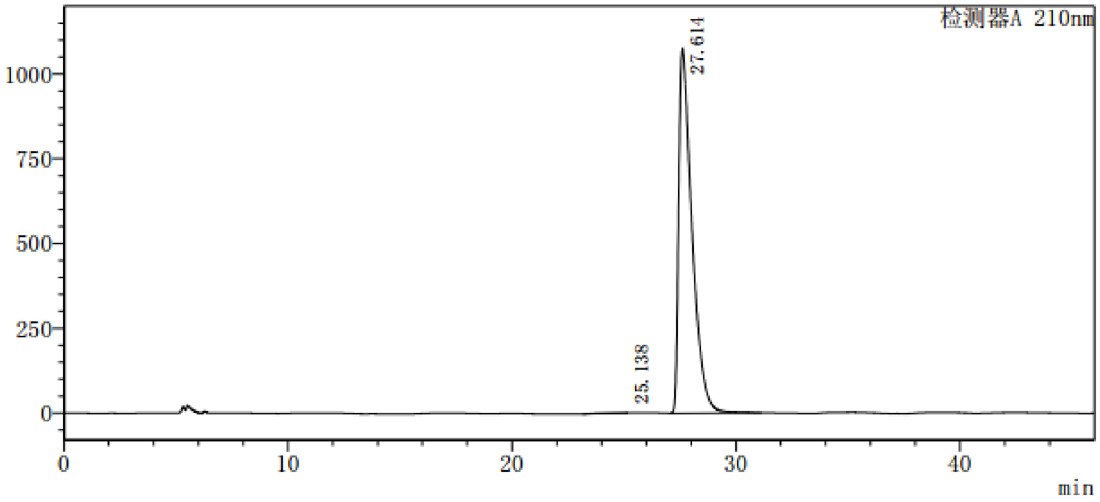

	Retention Time (min)	Area (%)	
**Peak 1**	25.138	0.172	>99% ee
**Peak 2**	27.614	99.828	

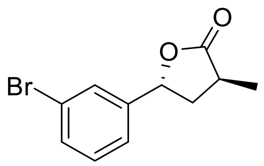

(*S*,*R*)-3q

HPLC conditions: Chiralcel OD Column, *n*-hexane/*i*-PrOH = 98/2, 210 nm, 0.6 mL/min, t_{major} = 31.240 min, t_{minor} = 28.012 min.

Racemate

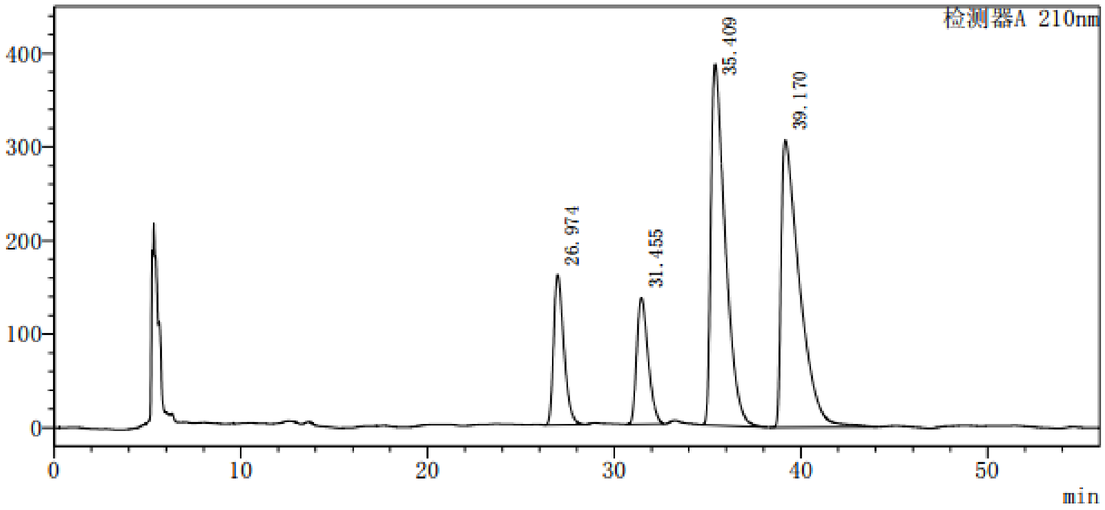

Chiral

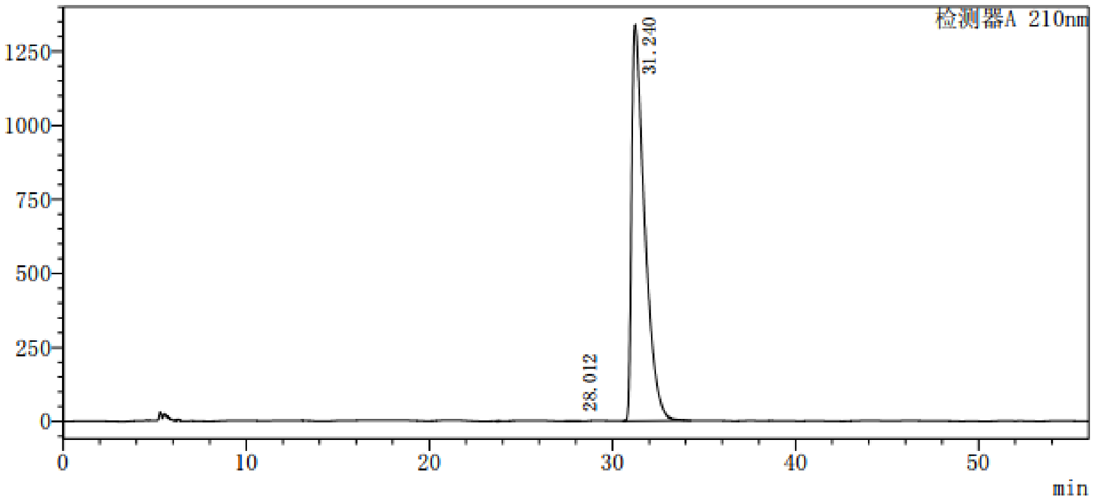

	Retention Time (min)	Area (%)	
**Peak 1**	28.012	0.003	>99% ee
**Peak 2**	31.240	99.997	

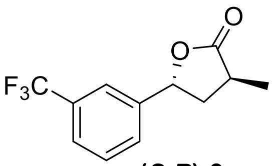

(*S*,*R*)-3r

HPLC conditions: Chiralcel OD Column, *n*-hexane/*i*-PrOH = 98/2, 210 nm, 0.6 mL/min, t_{major} = 18.083 min, t_{minor} = 21.231 min.

Racemate

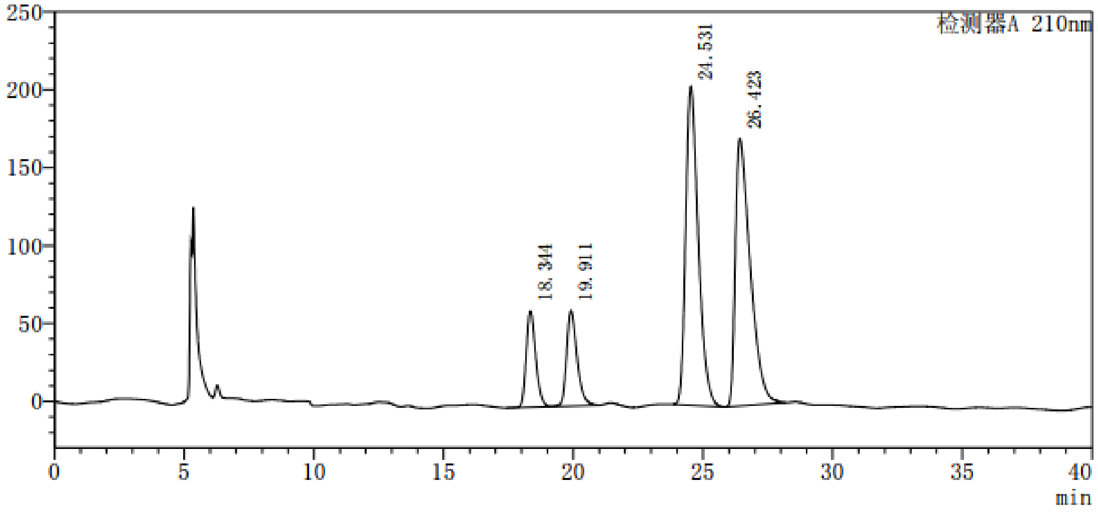

Chiral

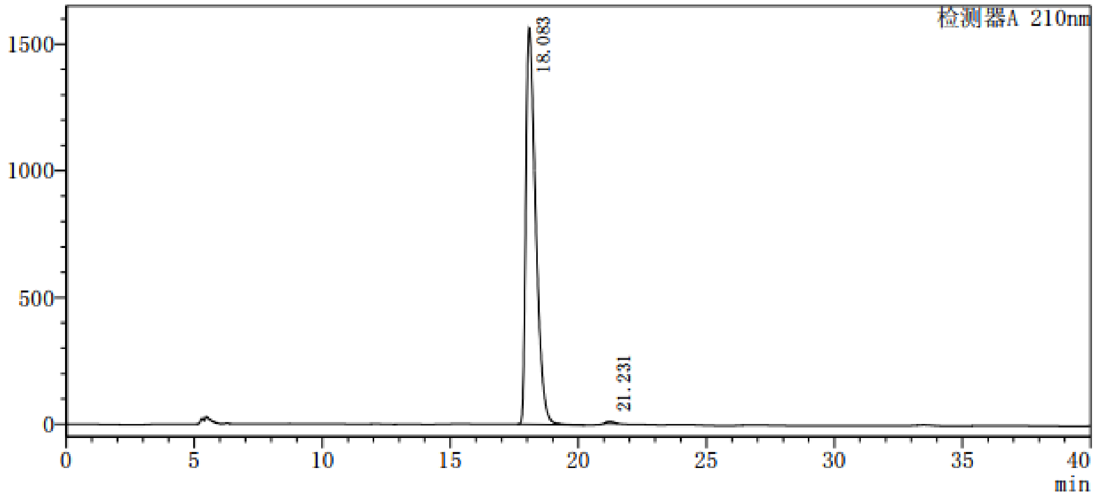

	Retention Time (min)	Area (%)	
**Peak 1**	18.083	99.606	>99% ee
**Peak 2**	21.231	0.394	

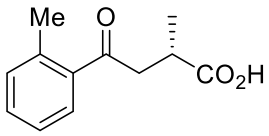

(*S*)-2s

HPLC conditions: Chiralcel OJ Column, *n*-hexane/*i*-PrOH = 95/5, 210 nm, 1.0 mL/min, t_{major} = 12.215 min, t_{minor} = 15.613 min.

Racemate

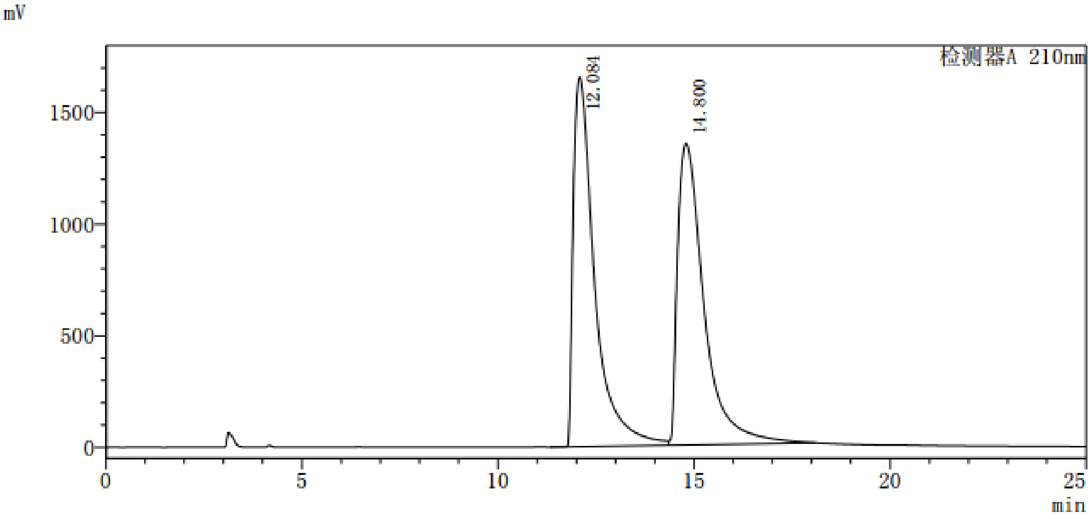

Chiral

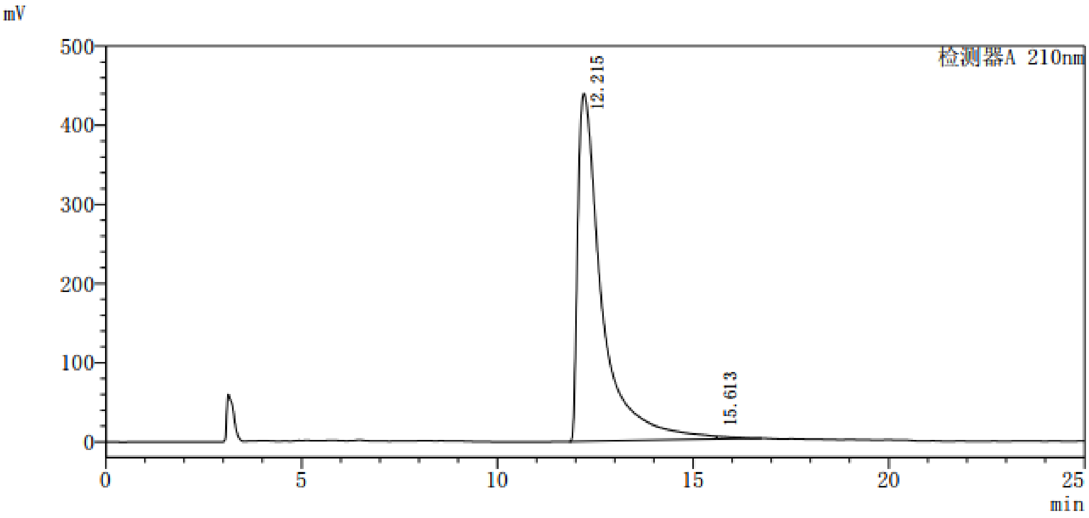

	Retention Time (min)	Area (%)	
**Peak 1**	12.215	99.418	99% ee
**Peak 2**	15.613	0.582	

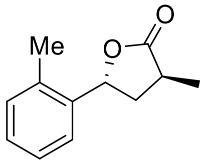

(*S*,*R*)-3s

HPLC conditions: Chiralcel OD Column, *n*-hexane/*i*-PrOH = 98/2, 210 nm, 0.6 mL/min, t_{major} = 31.347 min, t_{minor} = 27.915 min.

Racemate

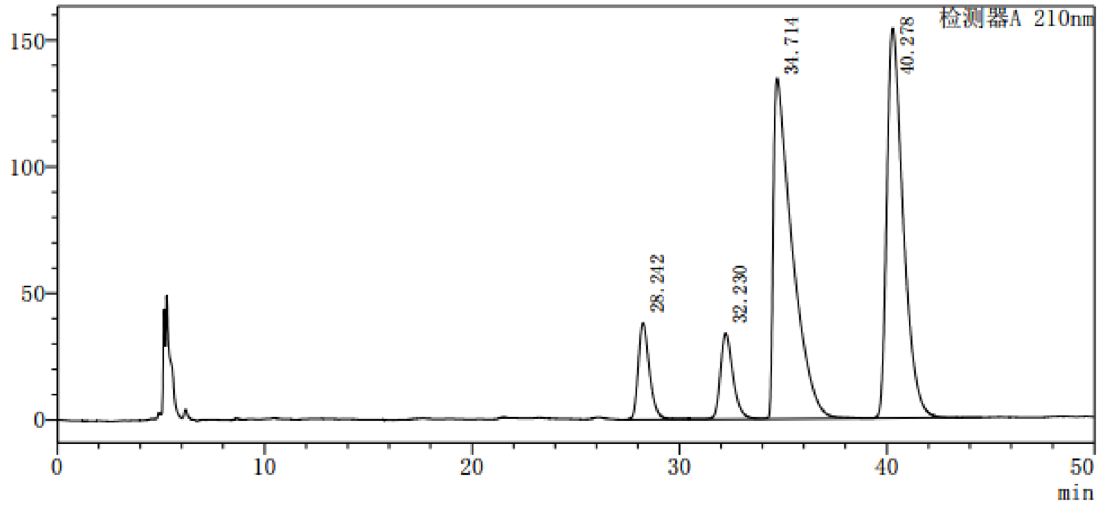

Chiral

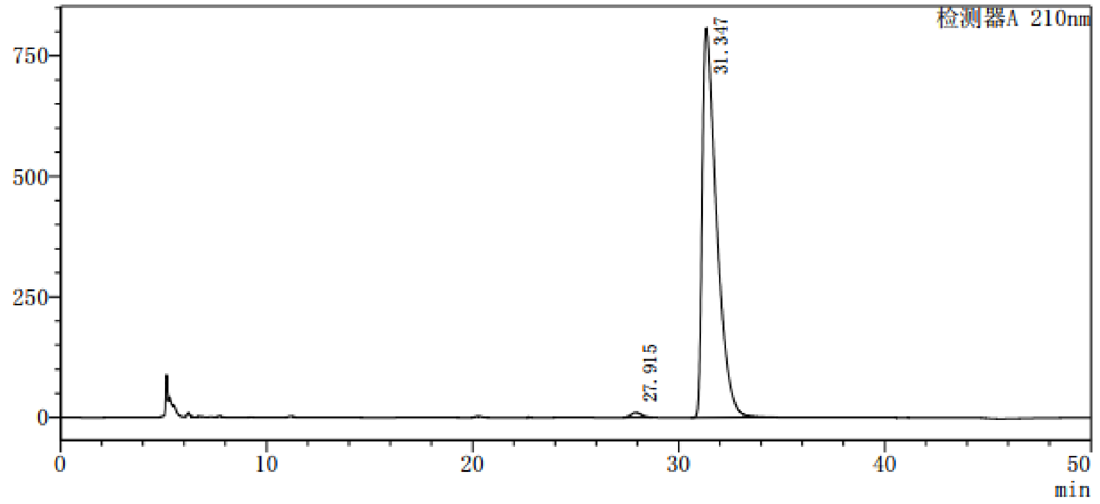

	Retention Time (min)	Area (%)	
**Peak 1**	27.915	0.871	98% ee
**Peak 2**	31.347	99.129	

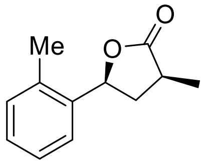

(*S*,*S*)-3s

HPLC conditions: Chiralcel OD Column, *n*-hexane/*i*-PrOH = 98/2, 210 nm, 0.6 mL/min, t_{major} = 38.997 min, t_{minor} = 34.935 min.

Racemate

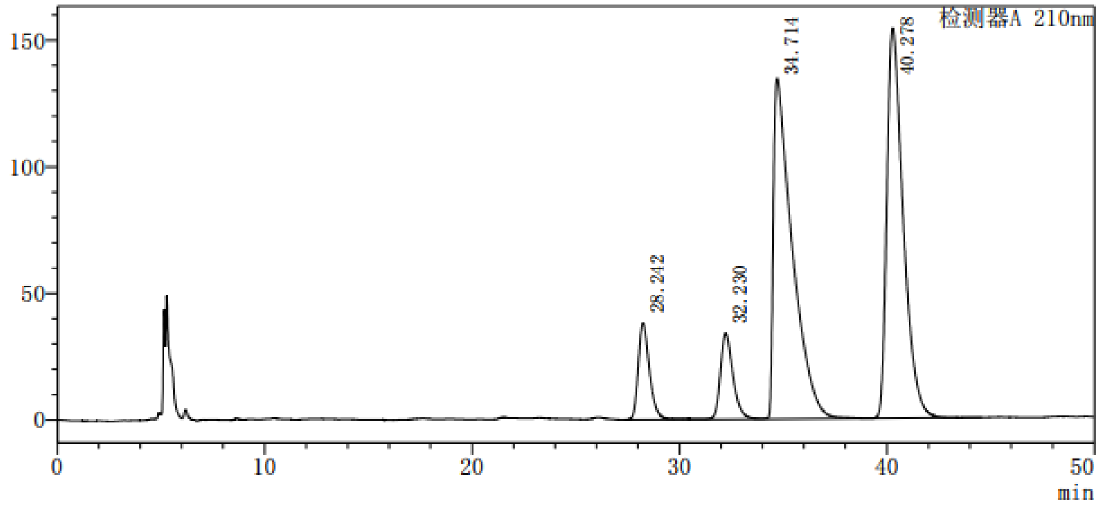

Chiral

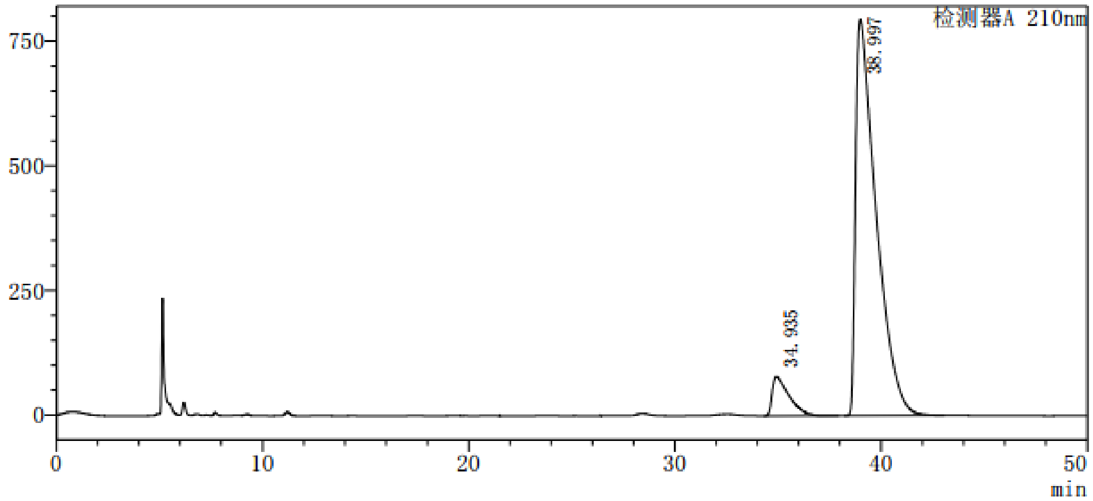

	Retention Time (min)	Area (%)	
**Peak 1**	34.935	7.421	85% ee
**Peak 2**	38.997	92.579	

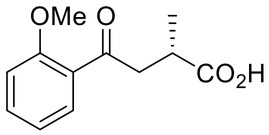

(*S*)-2t

HPLC conditions: Chiralcel OZ Column, *n*-hexane/*i*-PrOH = 95/5, 210 nm, 1.0 mL/min, t_{major} = 31.107 min, t_{minor} = 50.783 min.

Racemate

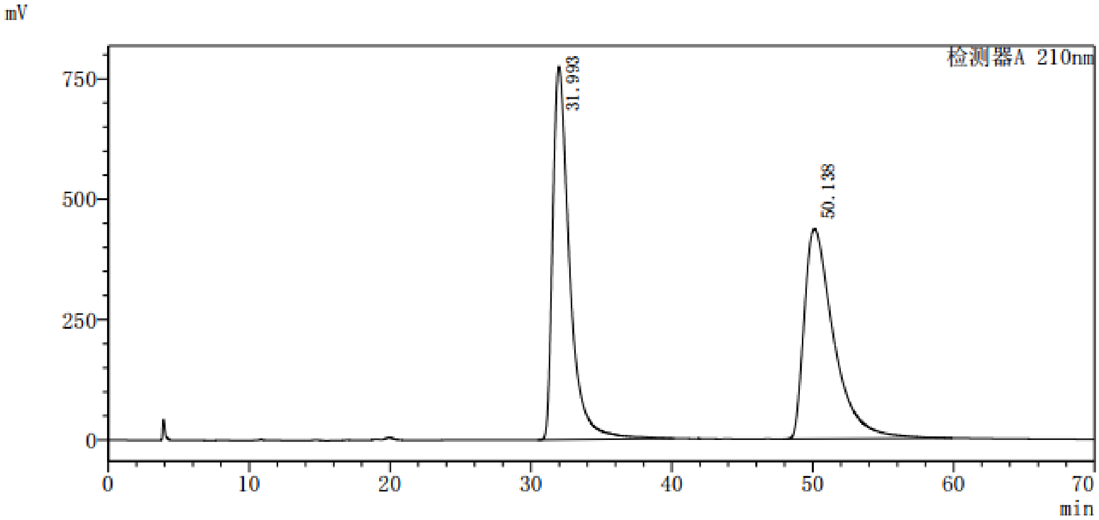

Chiral

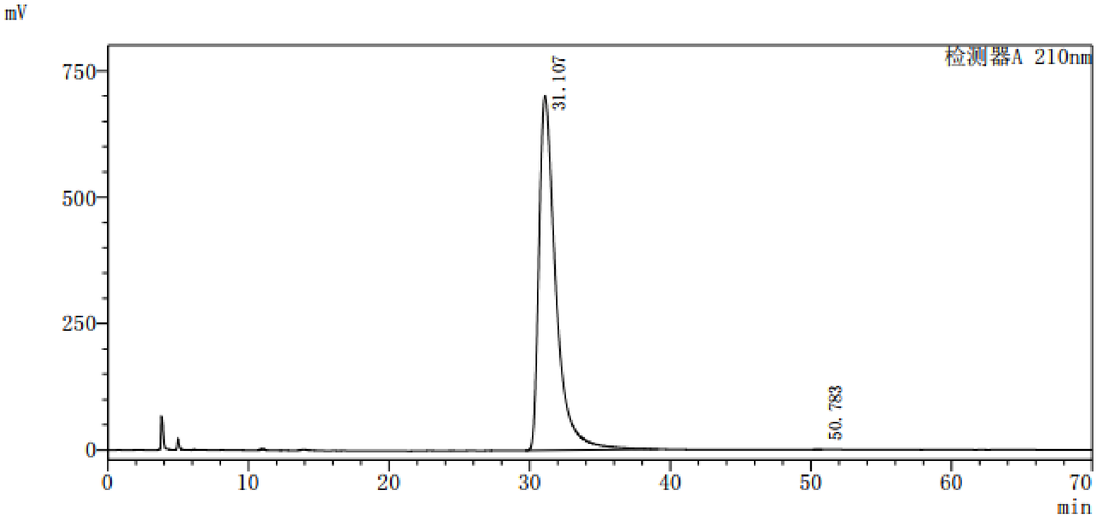

	Retention Time (min)	Area (%)	
**Peak 1**	31.107	99.967	>99% ee
**Peak 2**	50.783	0.033	

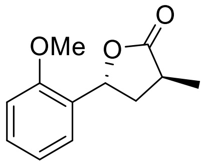

(*S*,*R*)-3t

HPLC conditions: Chiralcel OD Column, *n*-hexane/*i*-PrOH = 98/2, 210 nm, 0.6 mL/min, t_{major} = 25.564 min, t_{minor} = 23.804 min.

Racemate

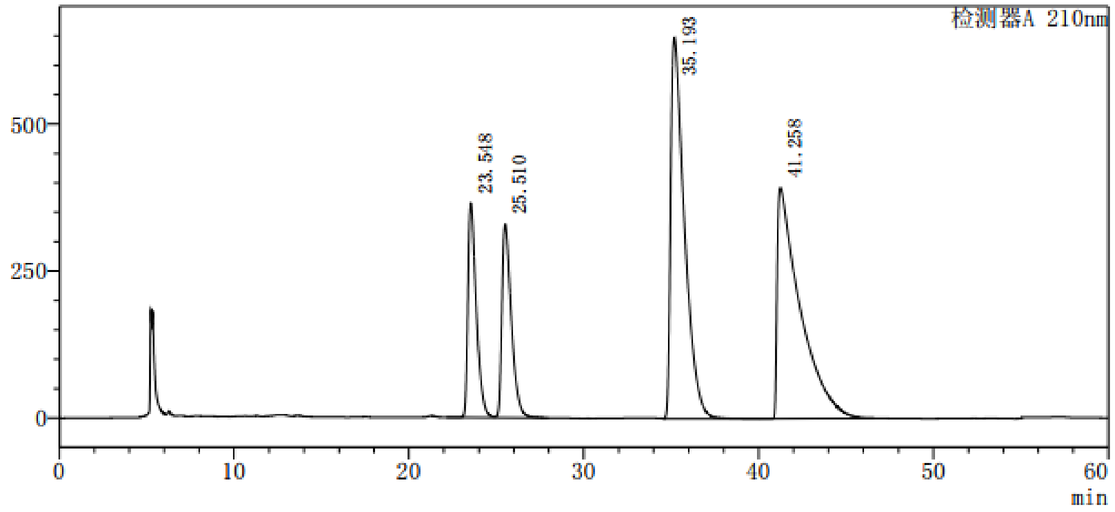

Chiral

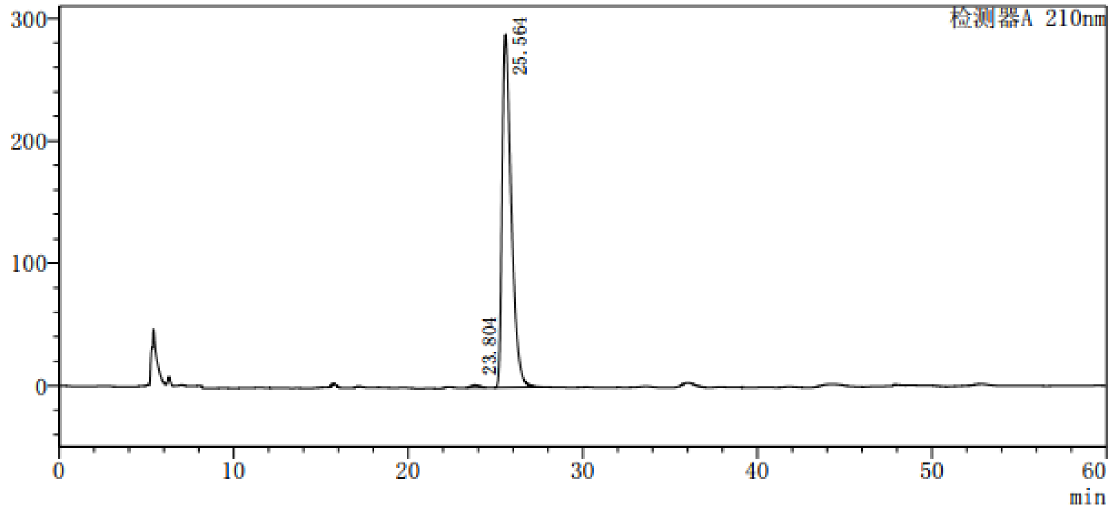

	Retention Time (min)	Area (%)	
**Peak 1**	23.804	0.568	99% ee
**Peak 2**	25.564	99.432	

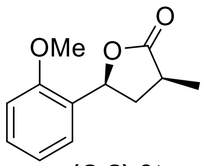

(*S*,*S*)-3t

HPLC conditions: Chiralcel OD Column, *n*-hexane/*i*-PrOH = 98/2, 210 nm, 0.6 mL/min, t_{major} = 35.757 min, t_{minor} = 44.059 min.

Racemate

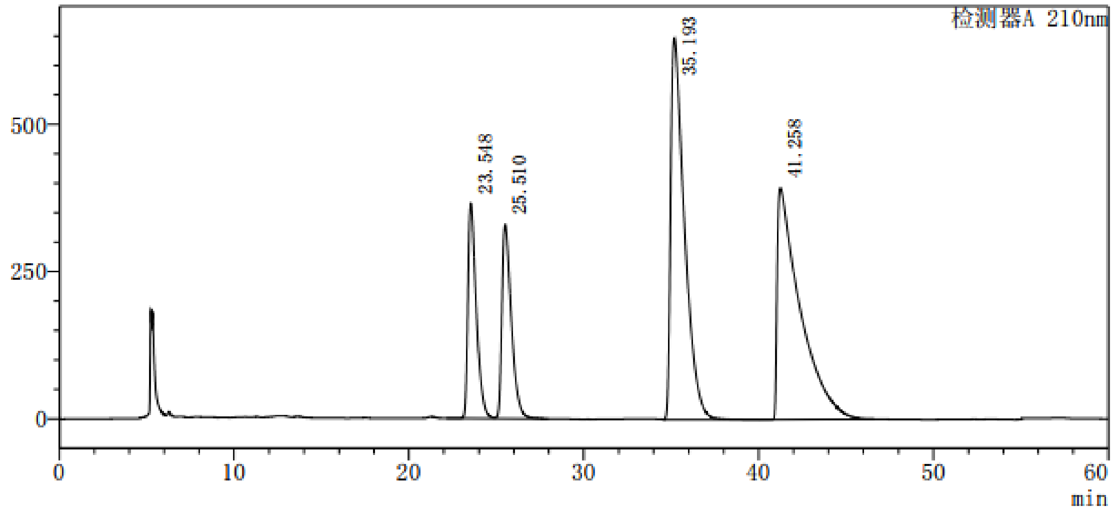

Chiral

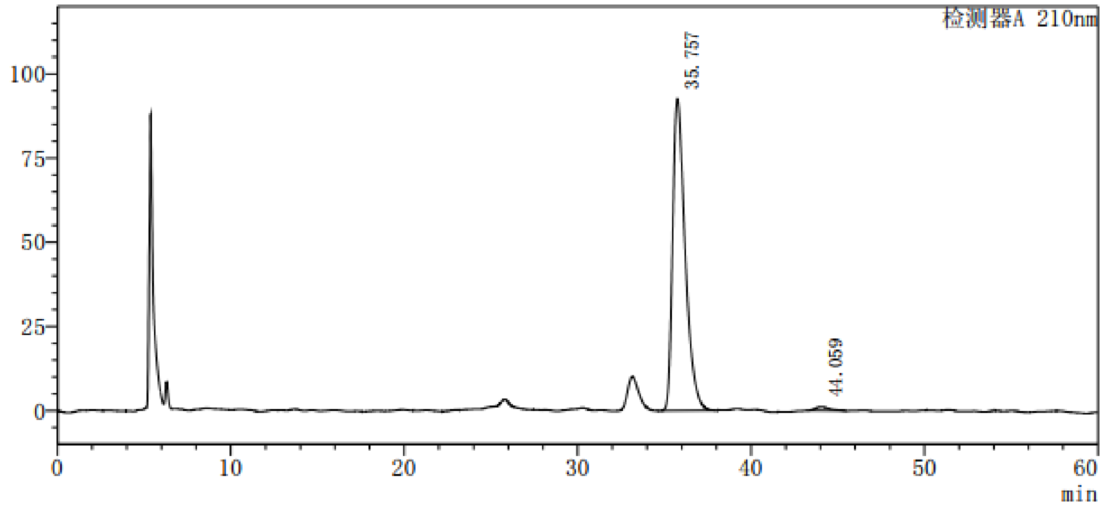

	Retention Time (min)	Area (%)	
**Peak 1**	35.757	98.688	97% ee
**Peak 2**	44.059	1.312	

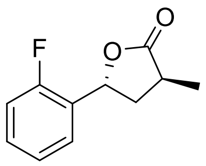

(*S*,*R*)-3u

HPLC conditions: Chiralpak IC-AD Column, *n*-hexane/*i*-PrOH = 97/3, 210 nm, 0.8 mL/min, t_{major} = 38.187 min, t_{minor} = 42.032 min.

Racemate

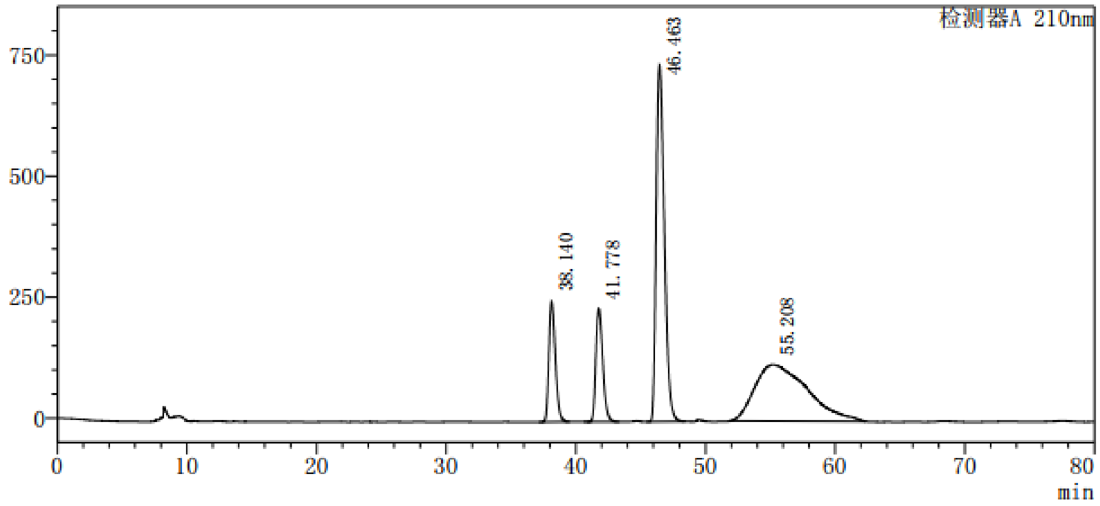

Chiral

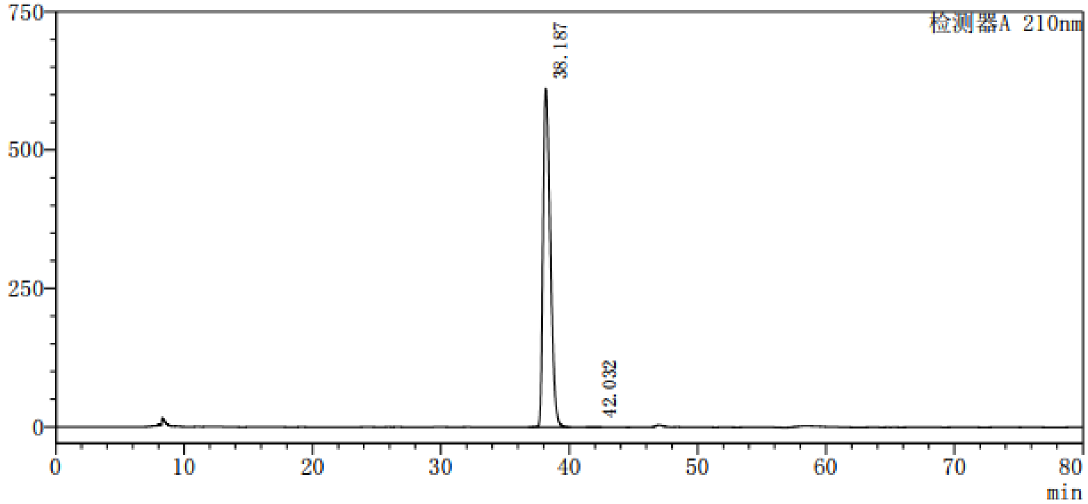

	Retention Time (min)	Area (%)	
**Peak 1**	38.187	99.973	>99% ee
**Peak 2**	42.032	0.027	

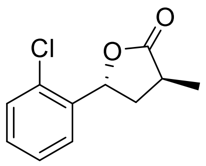

(*S*,*R*)-3v

HPLC conditions: Chiralcel OJ Column, *n*-hexane/*i*-PrOH = 98/2, 210 nm, 0.7 mL/min, t_{major} = 16.842 min, t_{minor} = 18.257 min.

Racemate

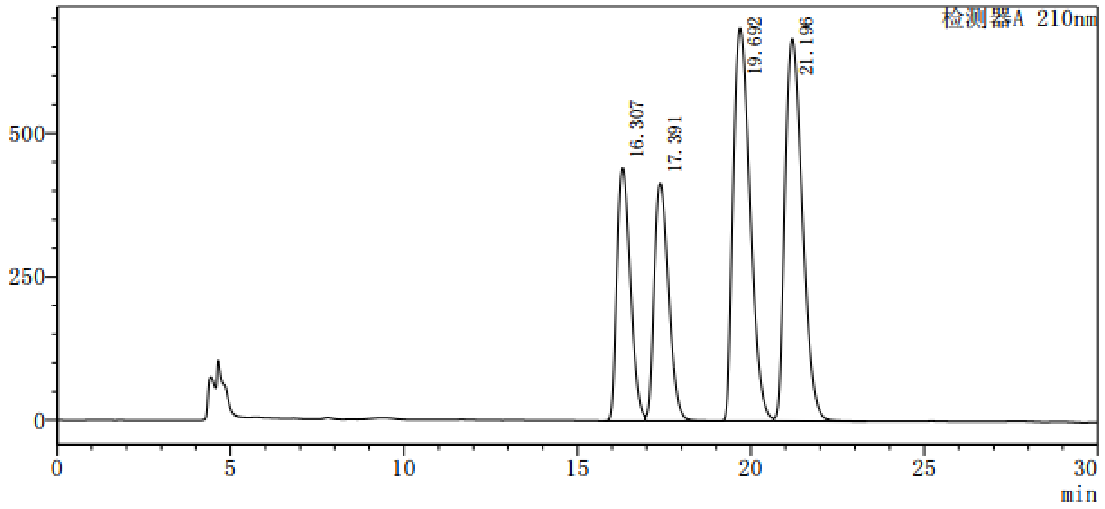

Chiral

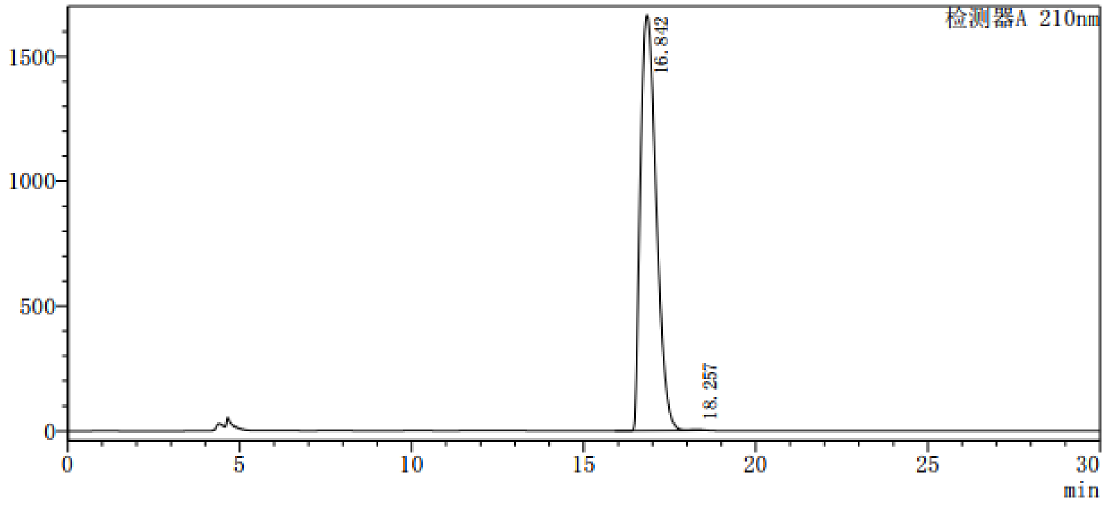

	Retention Time (min)	Area (%)	
**Peak 1**	16.842	99.863	>99% ee
**Peak 2**	18.257	0.137	

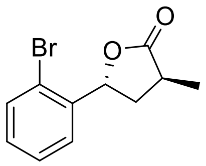

(*S*,*R*)-3w

HPLC conditions: Chiralcel OD Column, *n*-hexane/*i*-PrOH = 98/2, 210 nm, 0.6 mL/min, t_{major} = 21.459 min, t_{minor} = 23.697 min.

Racemate

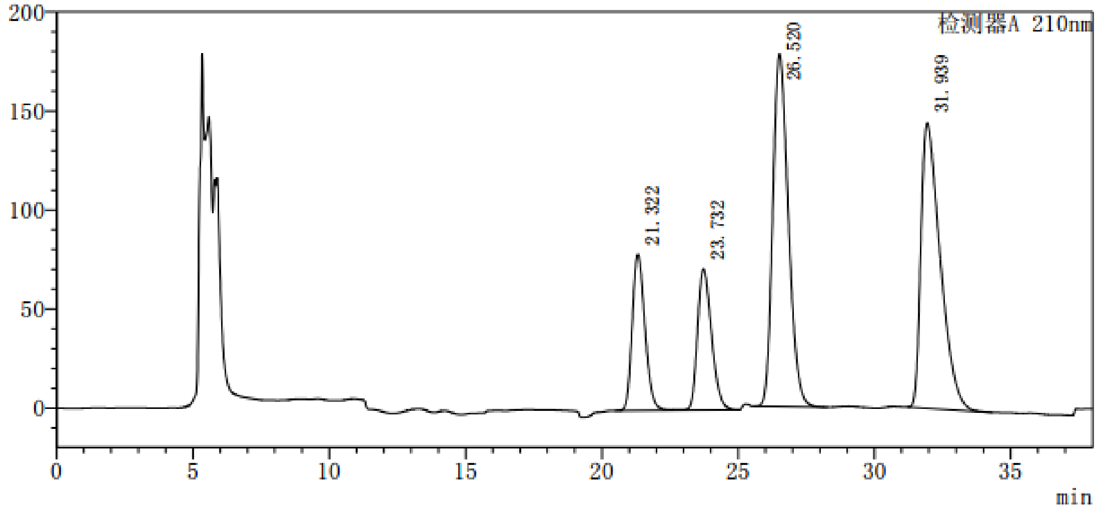

Chiral

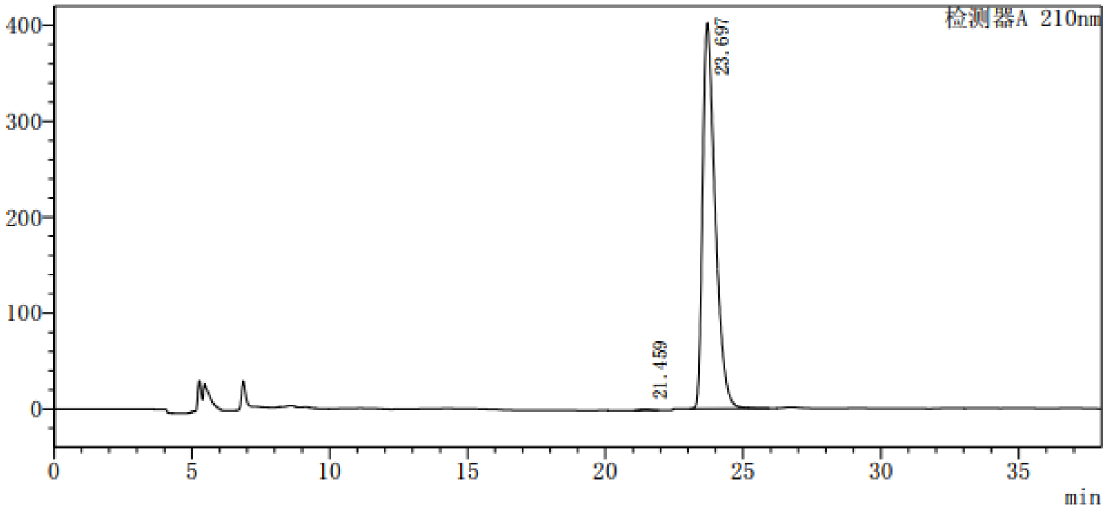

	Retention Time (min)	Area (%)	
**Peak 1**	21.459	0.214	>99% ee
**Peak 2**	23.697	99.786	

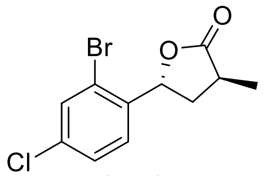

(*S*,*R*)-3x

HPLC conditions: Chiralcel OZ Column, *n*-hexane/*i*-PrOH = 98/2, 210 nm, 0.6 mL/min, t_{major} = 100.980 min, t_{minor} = 105.966 min.

Racemate

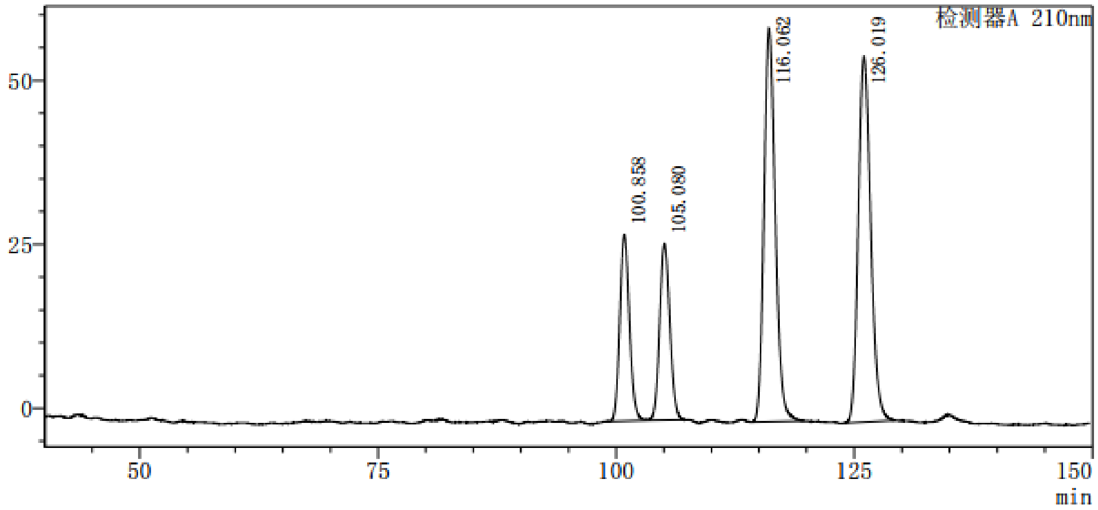

Chiral

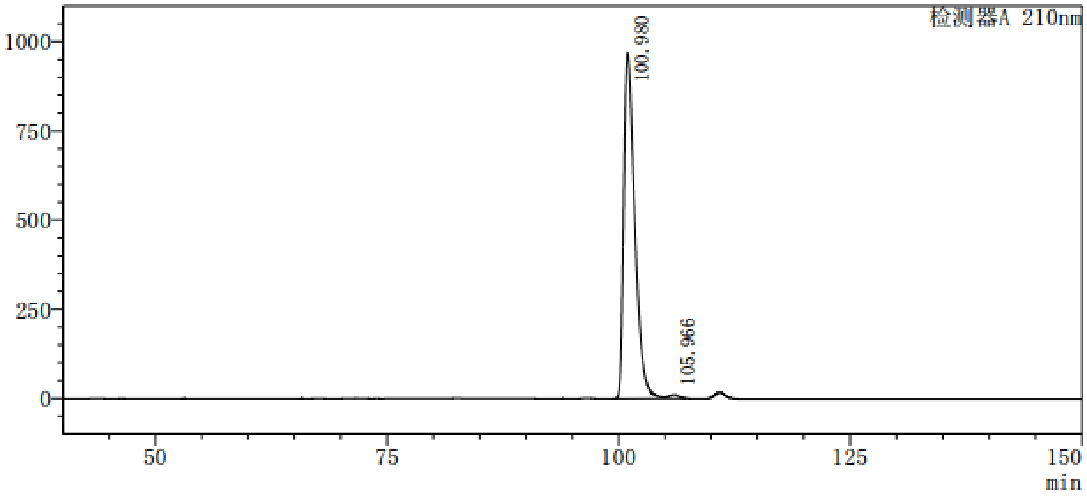

	Retention Time (min)	Area (%)	
**Peak 1**	100.980	98.982	98% ee
**Peak 2**	105.966	1.018	

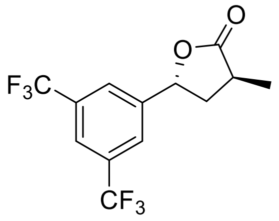

(*S*,*R*)-3y

HPLC conditions: Chiralcel OZ Column, *n*-hexane/*i*-PrOH = 98/2, 210 nm, 0.6 mL/min, t_{major} = 12.962 min, t_{minor} = 14.563 min.

Racemate

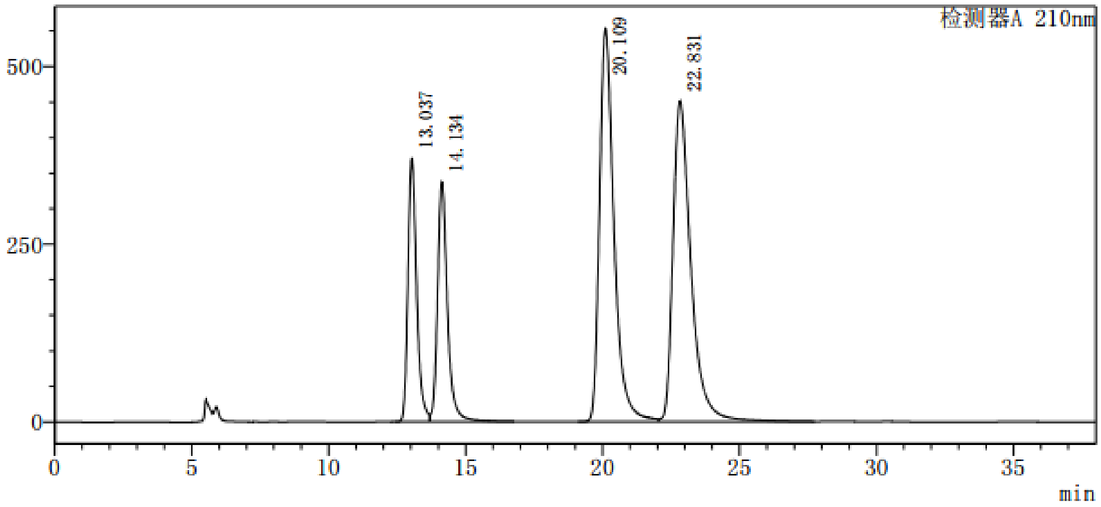

Chiral

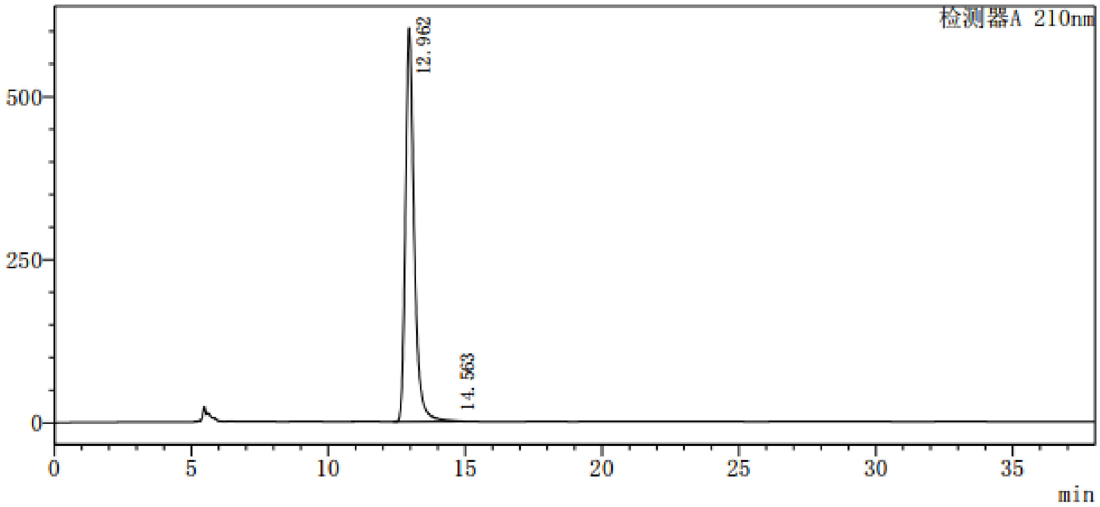

	Retention Time (min)	Area (%)	
**Peak 1**	12.962	99.939	>99% ee
**Peak 2**	14.563	0.061	

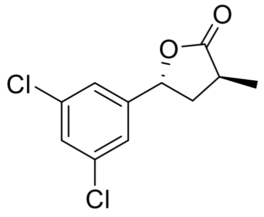

(*S*,*R*)-3z

HPLC conditions: Chiralcel OD Column, *n*-hexane/*i*-PrOH = 99/1, 210 nm, 0.3 mL/min, t_{major} = 47.492 min, t_{minor} = 51.622 min.

Racemate

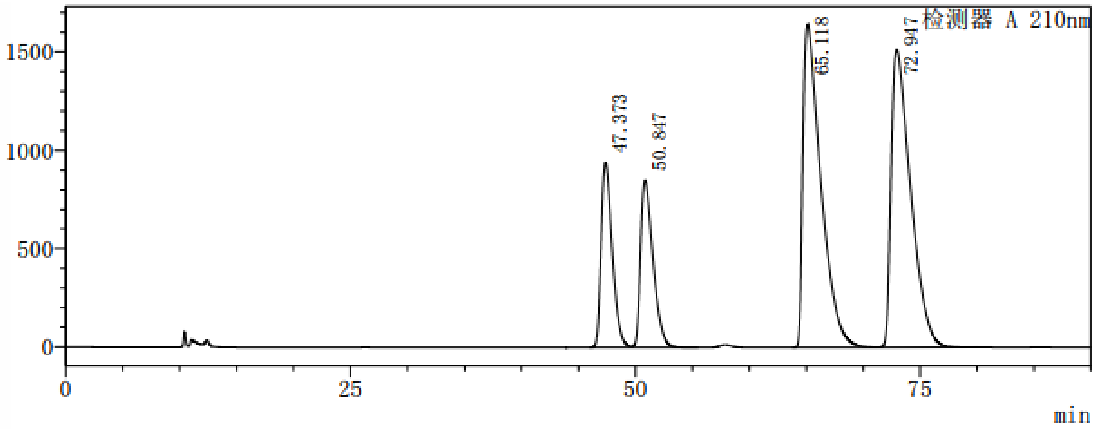

Chiral

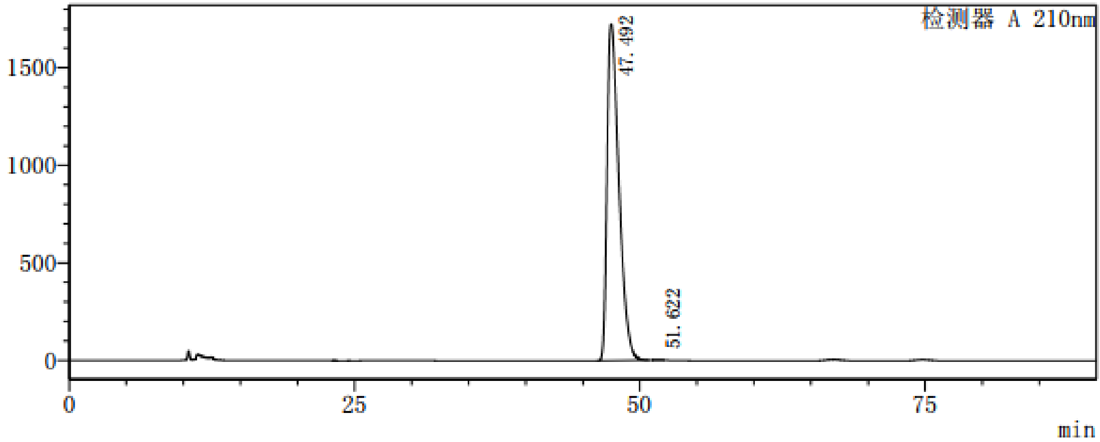

	Retention Time (min)	Area (%)	
**Peak 1**	47.492	99.941	>99% ee
**Peak 2**	51.622	0.059	

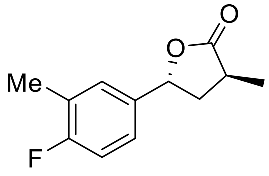

(*S*,*R*)-3aa

HPLC conditions: Chiralcel OD Column, *n*-hexane/*i*-PrOH = 98/2, 210 nm, 0.6 mL/min, t_{major} = 16.791 min, t_{minor} = 18.679 min.

Racemate

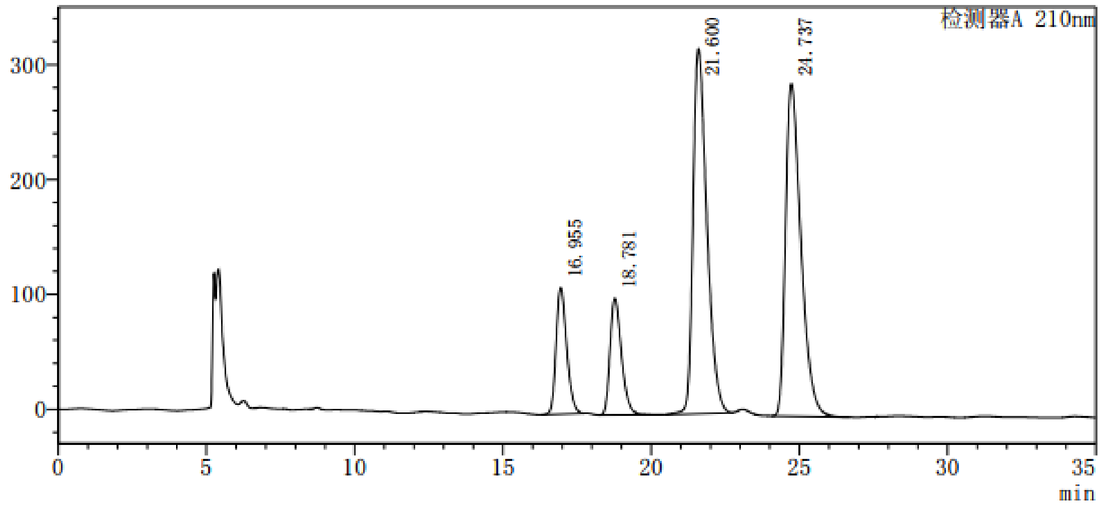

Chiral

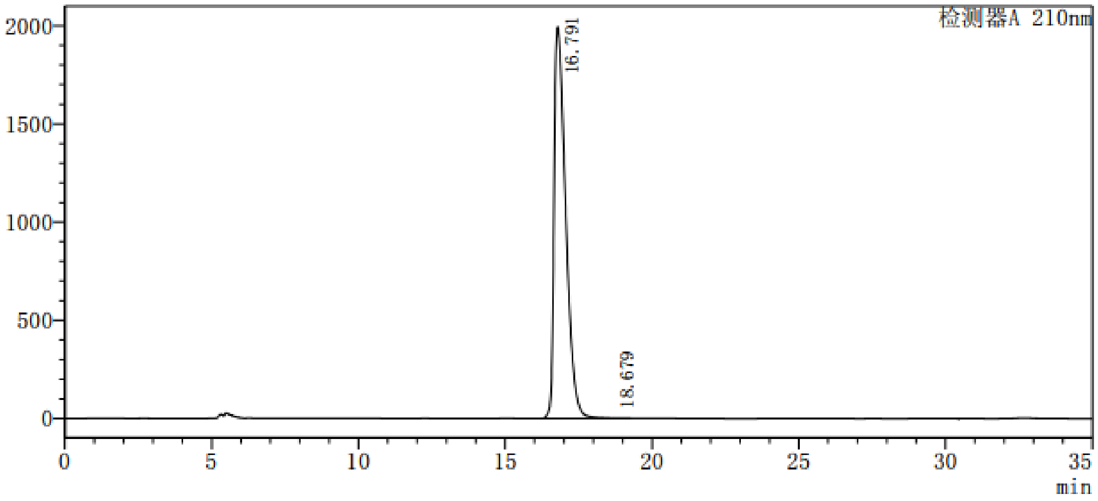

	Retention Time (min)	Area (%)	
**Peak 1**	16.791	99.962	>99% ee
**Peak 2**	18.679	0.038	

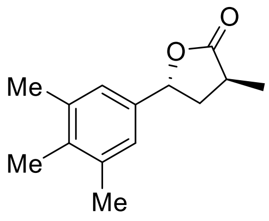

(*S*,*R*)-3ab

HPLC conditions: Chiralcel OD Column, *n*-hexane/*i*-PrOH = 98/2, 210 nm, 0.6 mL/min, t_{major} = 16.799 min, t_{minor} = 18.479 min.

Racemate

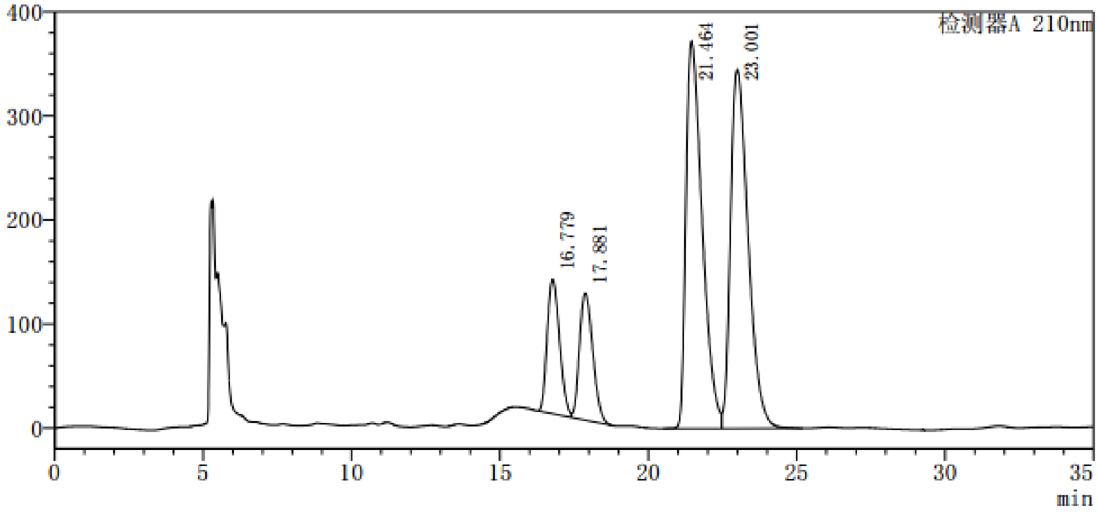

Chiral

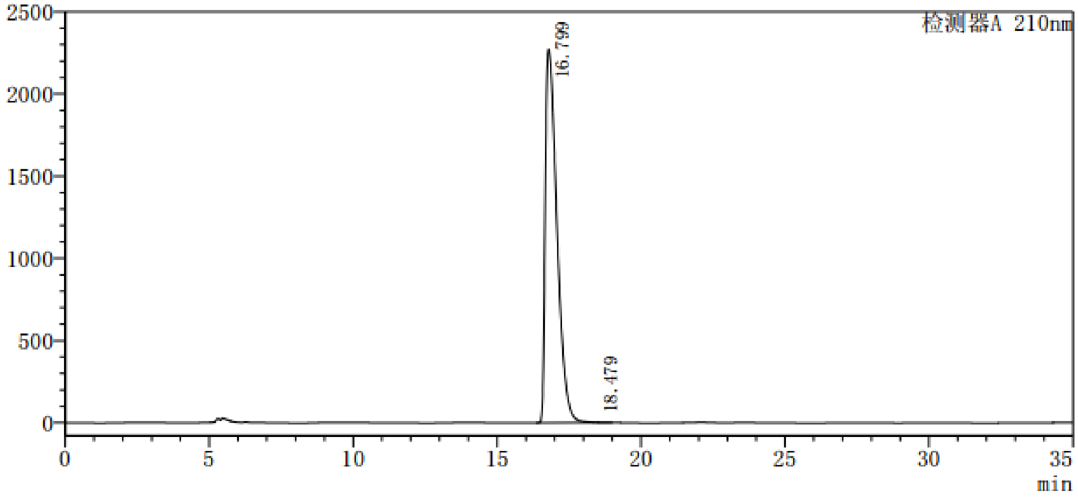

	Retention Time (min)	Area (%)	
**Peak 1**	16.799	99.965	>99% ee
**Peak 2**	18.479	0.035	

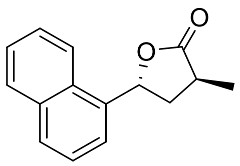

(*S*,*R*)-3ac

HPLC conditions: Chiralcel OZ Column, *n*-hexane/*i*-PrOH = 98/2, 210 nm, 0.6 mL/min, t_{major} = 33.541 min, t_{minor} = 42.212 min.

Racemate

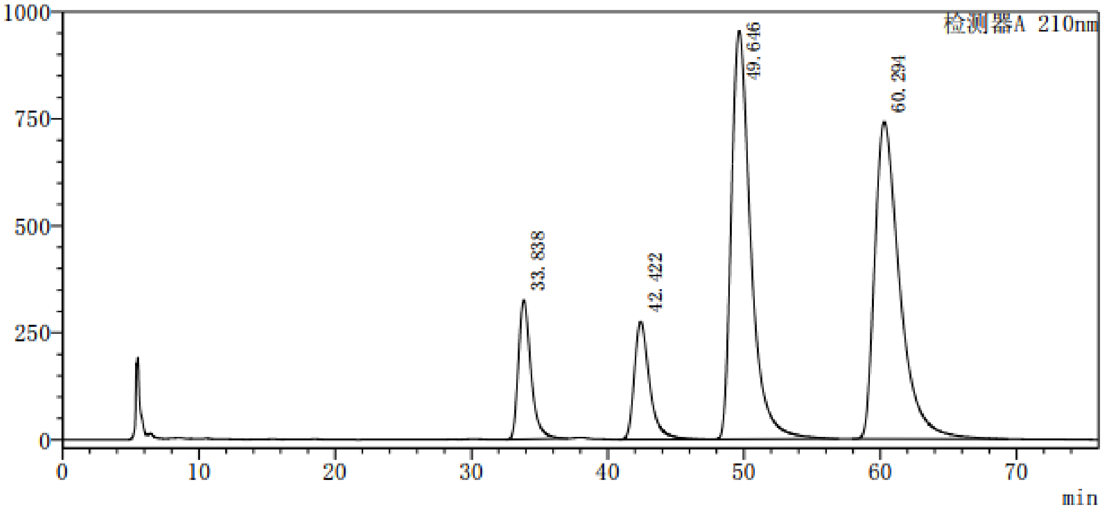

Chiral

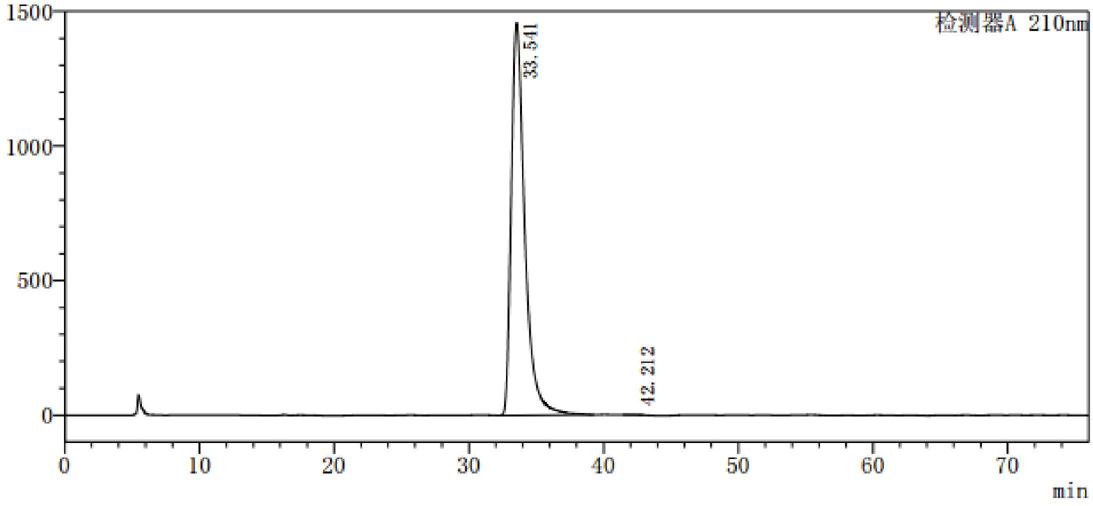

	Retention Time (min)	Area (%)	
**Peak 1**	33.541	99.950	>99% ee
**Peak 2**	42.212	0.050	

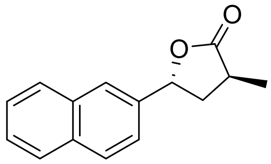

(*S*,*R*)-3ad

HPLC conditions: Chiralcel OD Column, *n*-hexane/*i*-PrOH = 98/2, 210 nm, 0.6 mL/min, t_{major} = 34.917 min, t_{minor} = 41.135 min.

Racemate

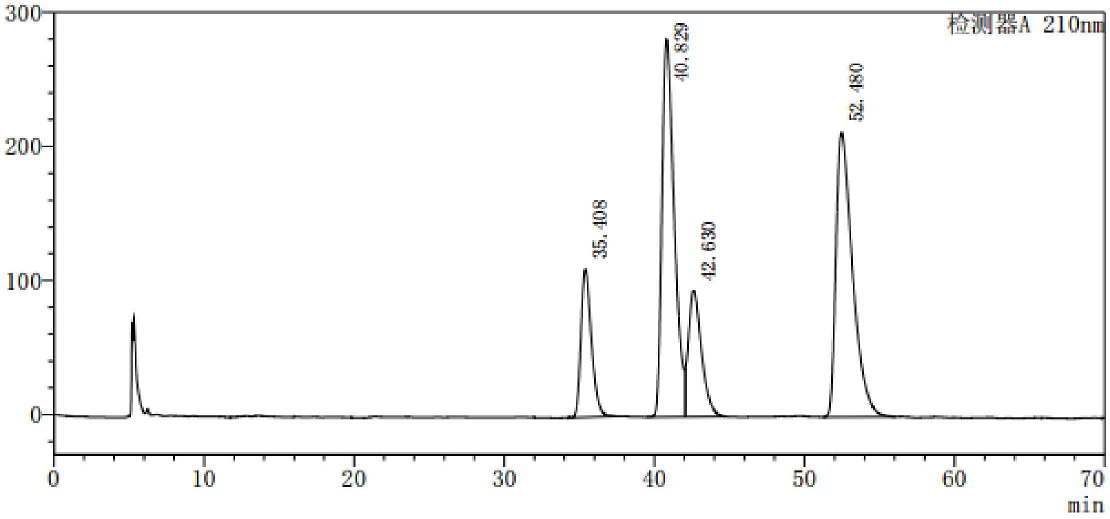

Chiral

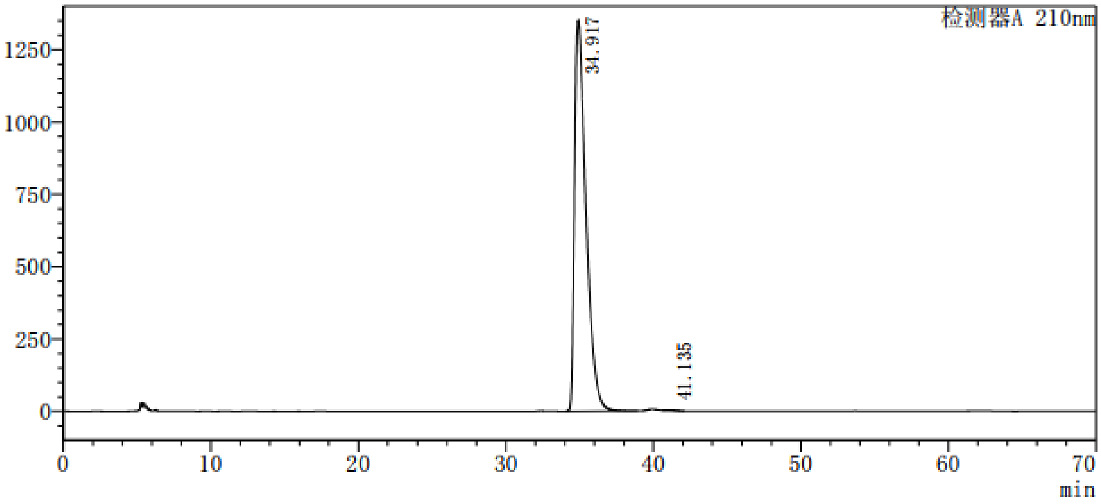

	Retention Time (min)	Area (%)	
**Peak 1**	34.917	99.883	>99% ee
**Peak 2**	41.135	0.117	

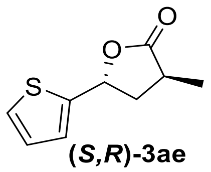

(*S*,*R*)-3ae

HPLC conditions: Chiralpak IE Column, *n*-hexane/*i*-PrOH = 95/5, 210 nm, 1.0 mL/min, t_{major} = 31.082 min, t_{minor} = 26.050 min.

Racemate

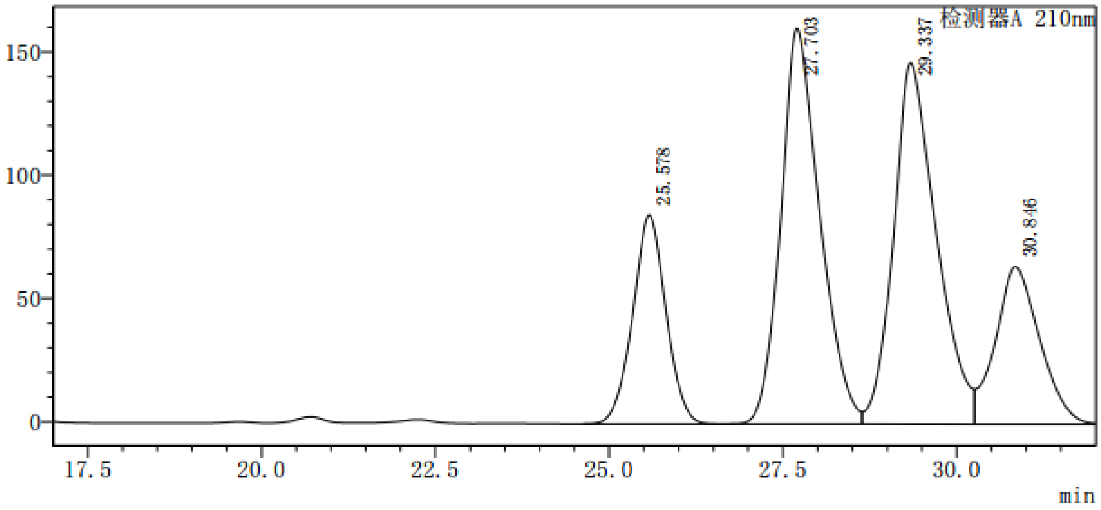

Chiral

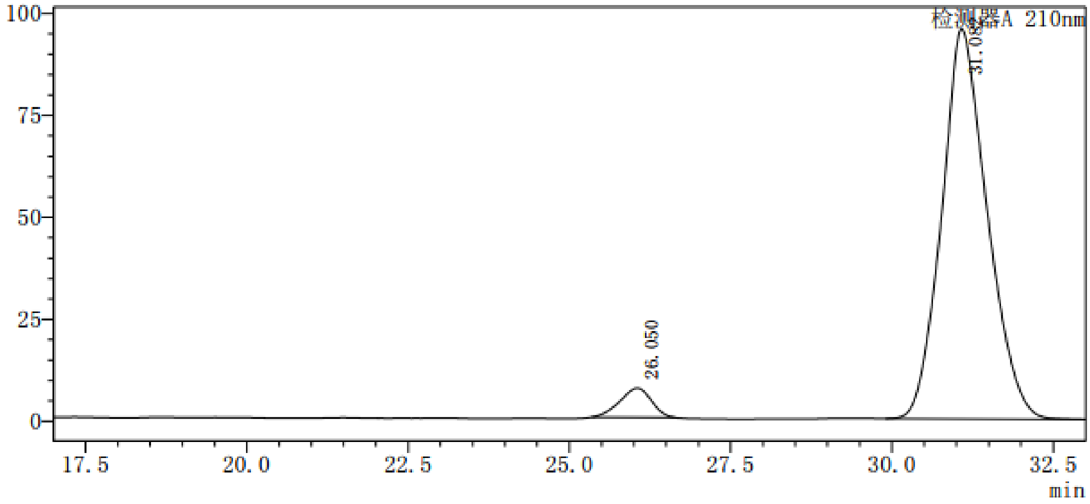

	Retention Time (min)	Area (%)	
**Peak 1**	26.050	4.924	90% ee
**Peak 2**	31.082	95.076	

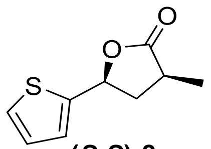

(*S*,*S*)-3ae

HPLC conditions: Chiralpak IE Column, *n*-hexane/*i*-PrOH = 95/5, 210 nm, 1.0 mL/min, t_{major} = 29.542 min, t_{minor} = 28.237 min.

Racemate

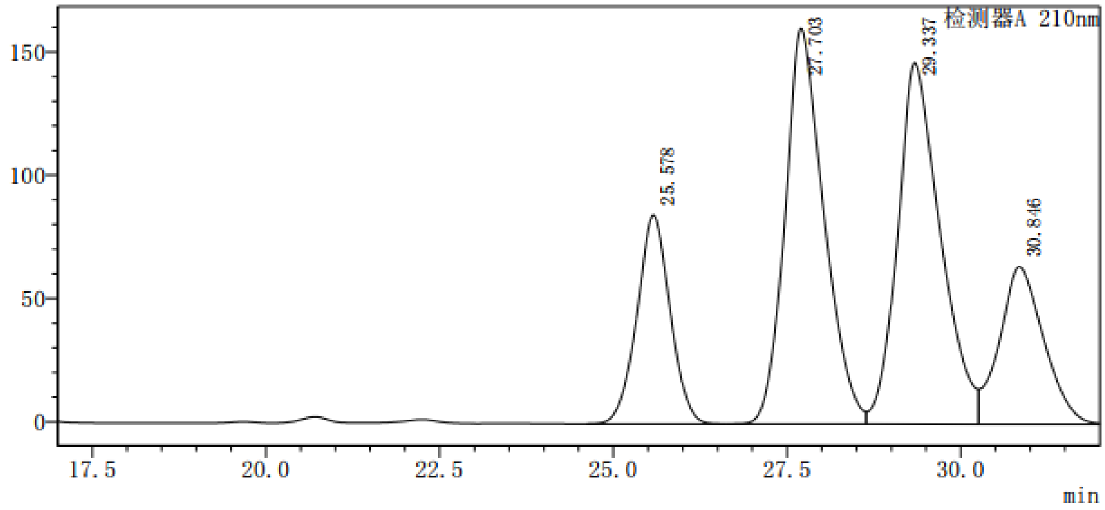

Chiral

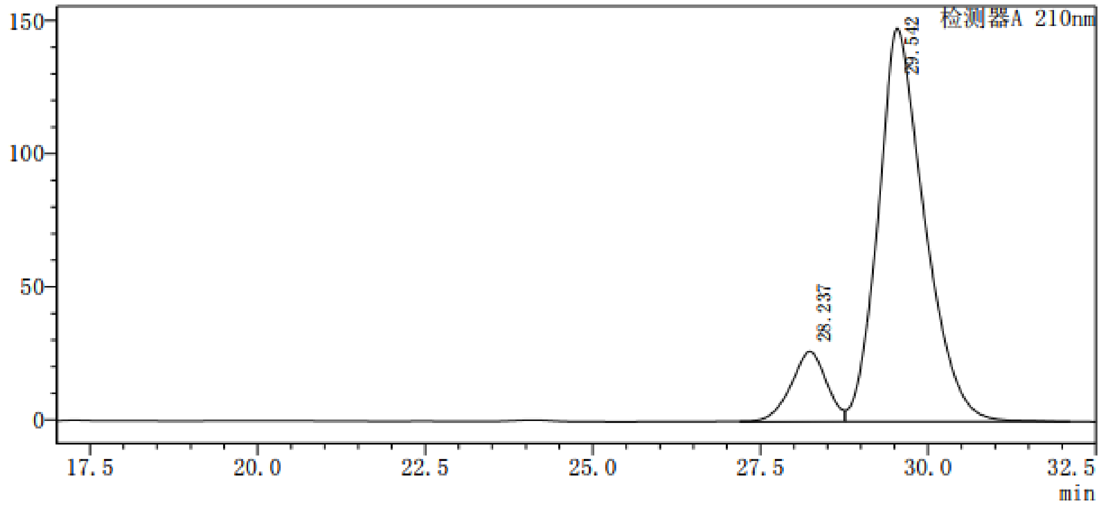

	Retention Time (min)	Area (%)	
**Peak 1**	28.237	12.086	76% ee
**Peak 2**	29.542	87.914	

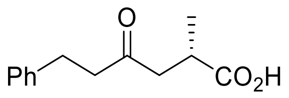

(*S*)-2af

HPLC conditions: Chiralcel OJ Column, *n*-hexane/*i*-PrOH = 97/3, 210 nm, 1.0 mL/min, t_{major} = 60.000 min, t_{minor} = 43.871 min.

Racemate

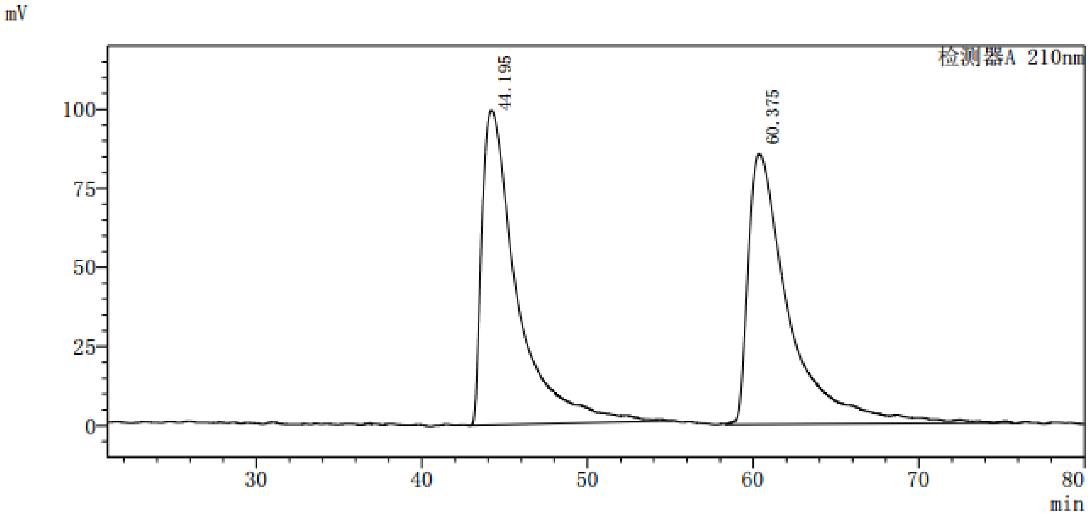

Chiral

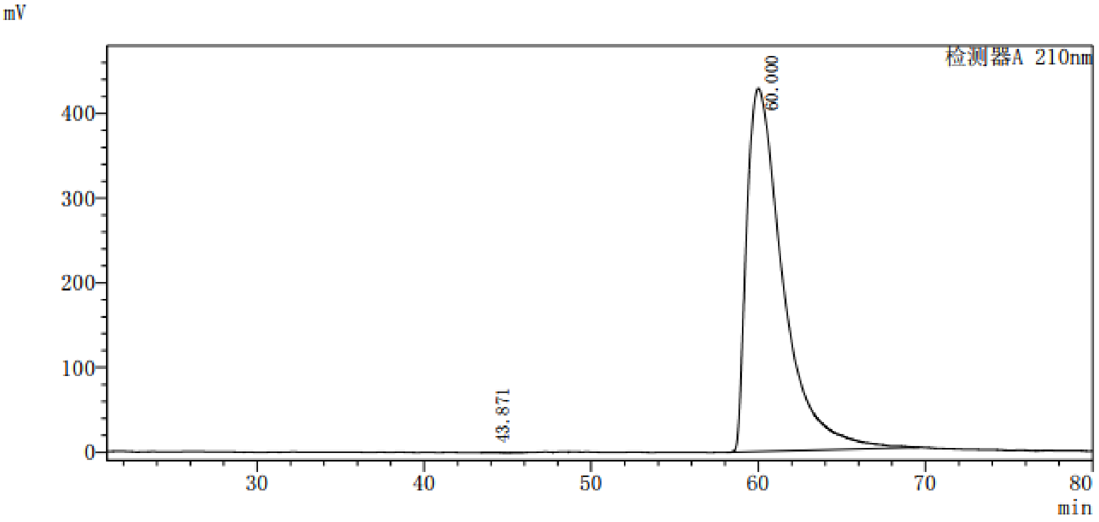

	Retention Time (min)	Area (%)	
**Peak 1**	43.871	0.013	>99% ee
**Peak 2**	60.000	99.987	

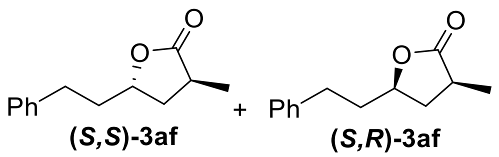

HPLC conditions: Chiralcel OJ Column, *n*-hexane/*i*-PrOH = 95/5, 210 nm, 0.8 mL/min, **(*S*,*S*)-3af**: t_{major} = 30.916 min, t_{minor} = 47.449 min; **(*S*,*R*)-3af**: t_{major} = 56.262 min, t_{minor} = 37.867 min.

Racemate

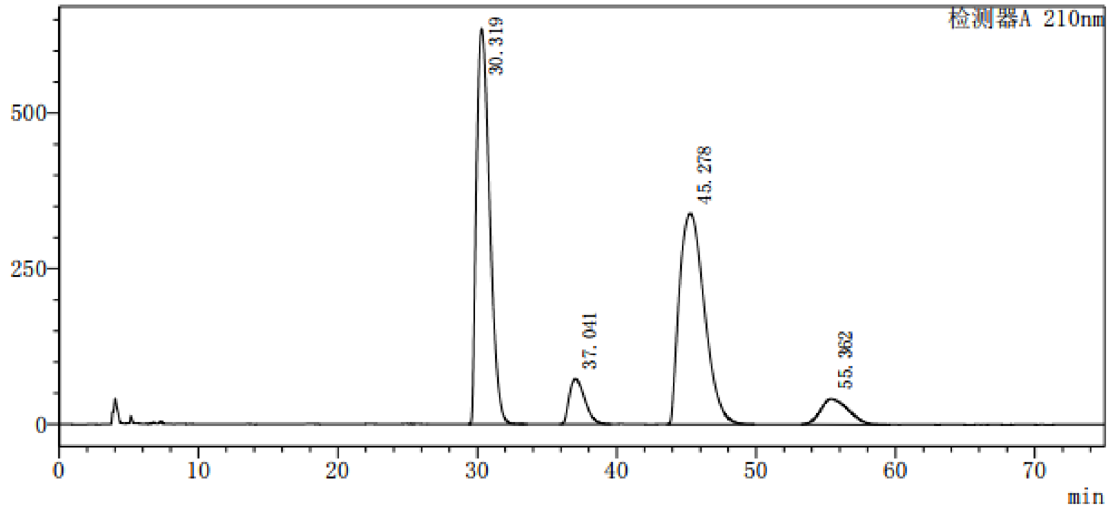

Chiral

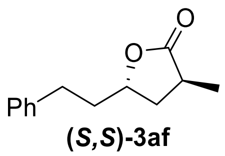

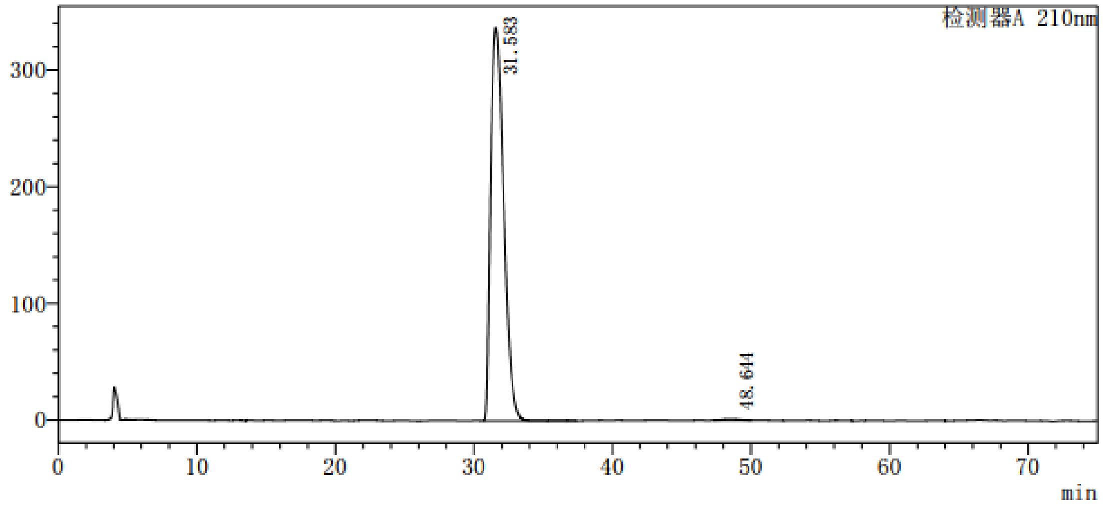

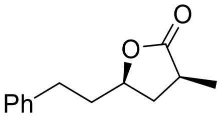

(*S*,*R*)-3af

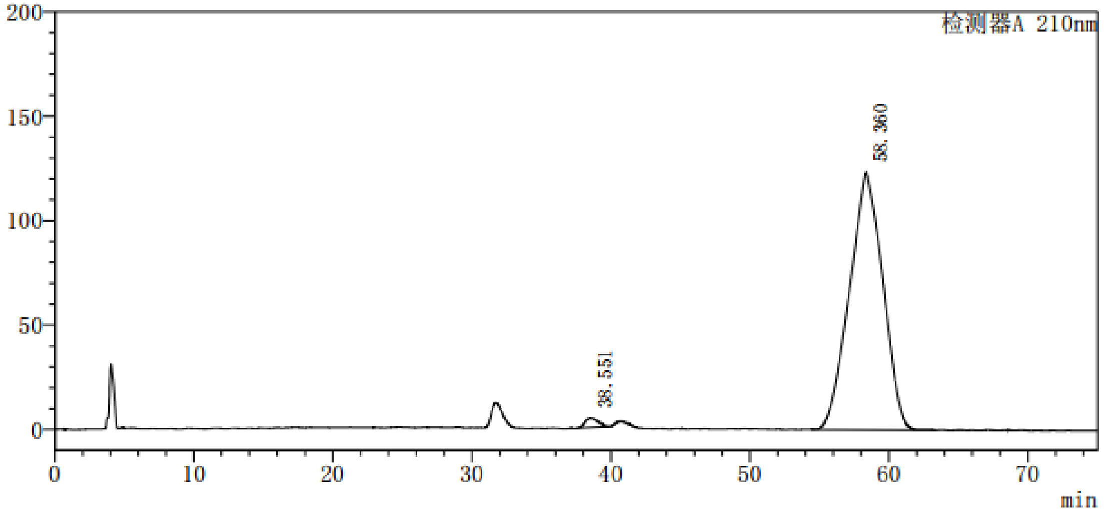

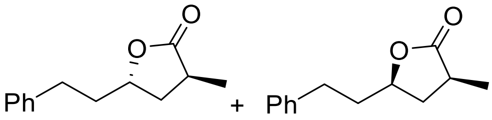

(*S*,*S*)-3af + (*S*,*R*)-3af

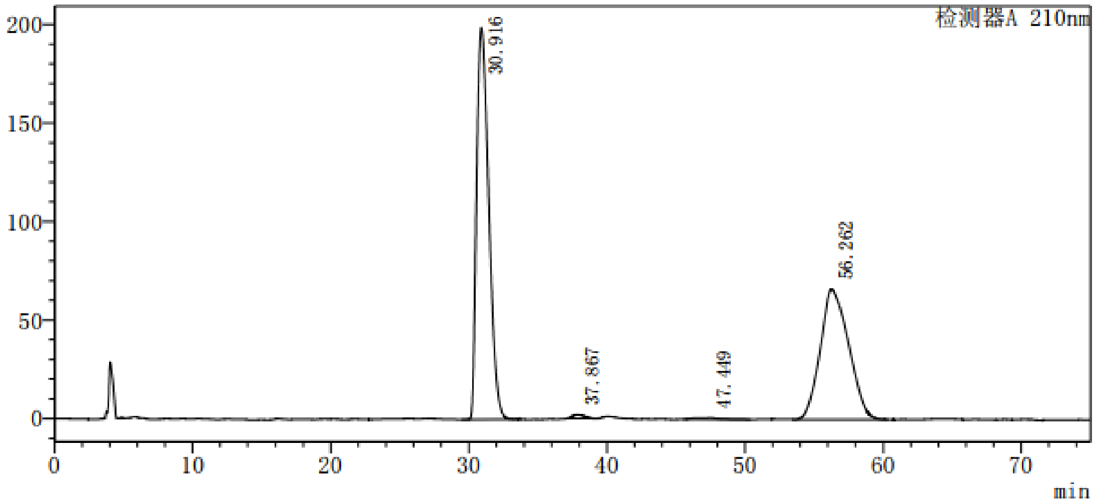

	Retention Time (min)	Area (%)	Ee (%)
**Peak 1**	30.916	55.944	**(*S*,*S*)-3af:** 98 (Peaks 1 and 3)
**Peak 2**	37.867	0.517	
**Peak 3**	47.449	0.459	**(S,*R*)-3af:** 98 (Peaks 2 and 4)
**Peak 4**	56.262	43.080	

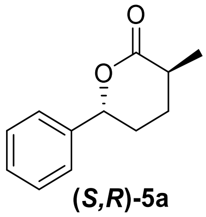

(*S*,*R*)-5a

HPLC conditions: Chiralcel OD-OJ Column, *n*-hexane/*i*-PrOH = 92/8, 210 nm, 1.0 mL/min, t_{major} = 42.082 min, t_{minor} = 38.588 min.

Racemate

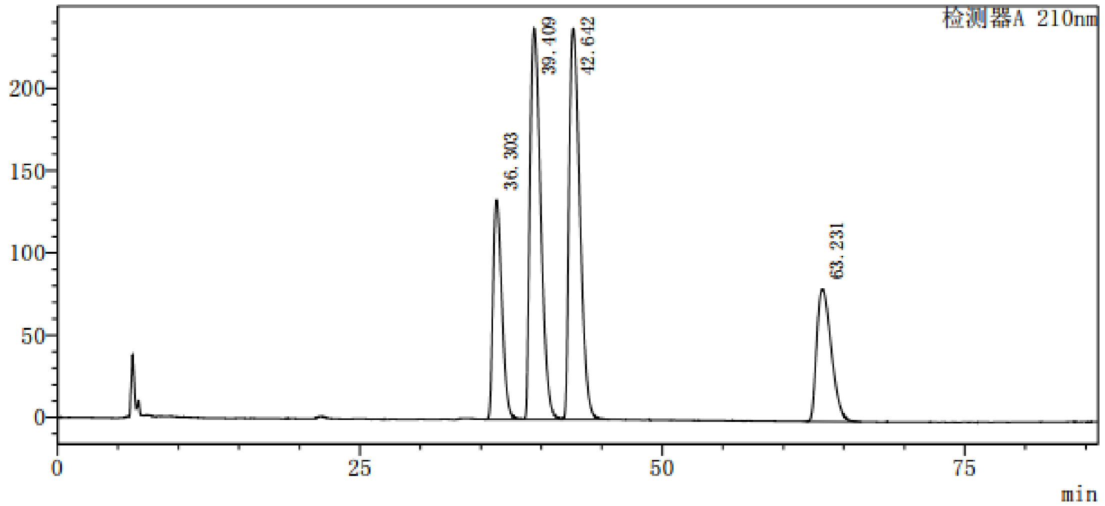

Chiral

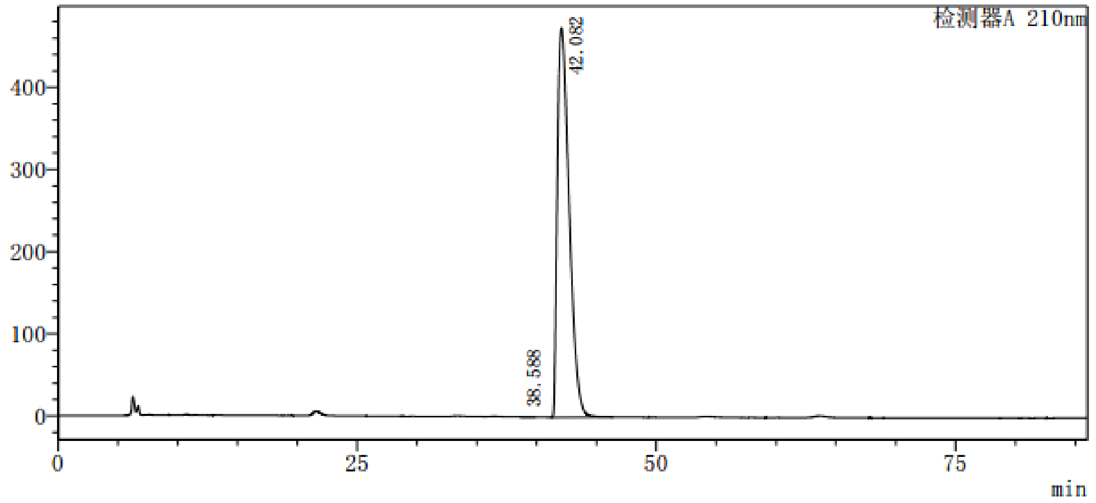

	Retention Time (min)	Area (%)	
**Peak 1**	38.588	0.013	>99% ee
**Peak 2**	42.082	99.987	

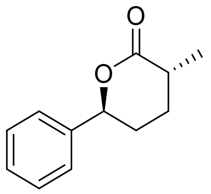

(*R*,*S*)-5a

HPLC conditions: Chiralcel OD-OJ Column, *n*-hexane/*i*-PrOH = 92/8, 210 nm, 1.0 mL/min, t_{major} = 39.402 min, t_{minor} = 42.858 min.

Racemate

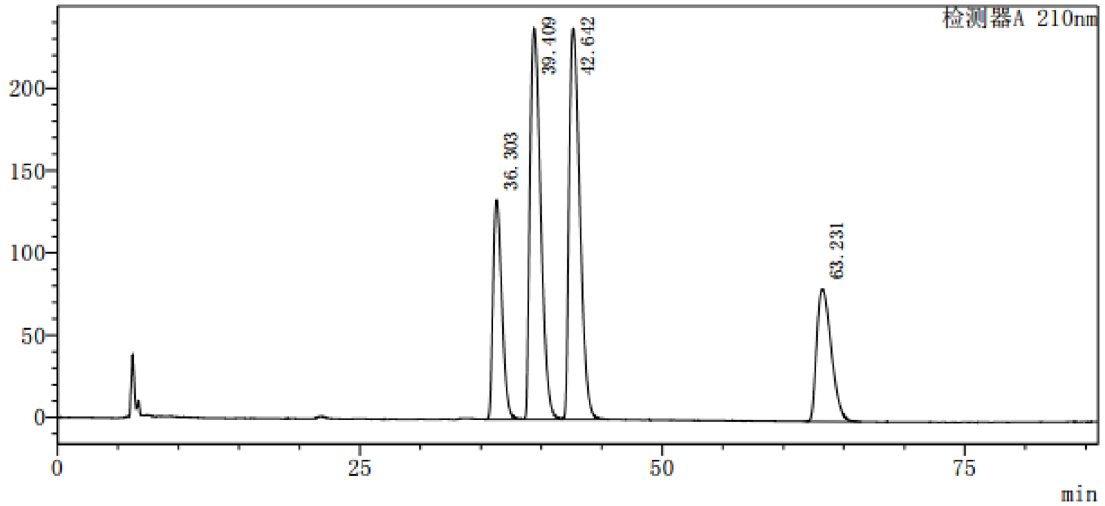

Chiral

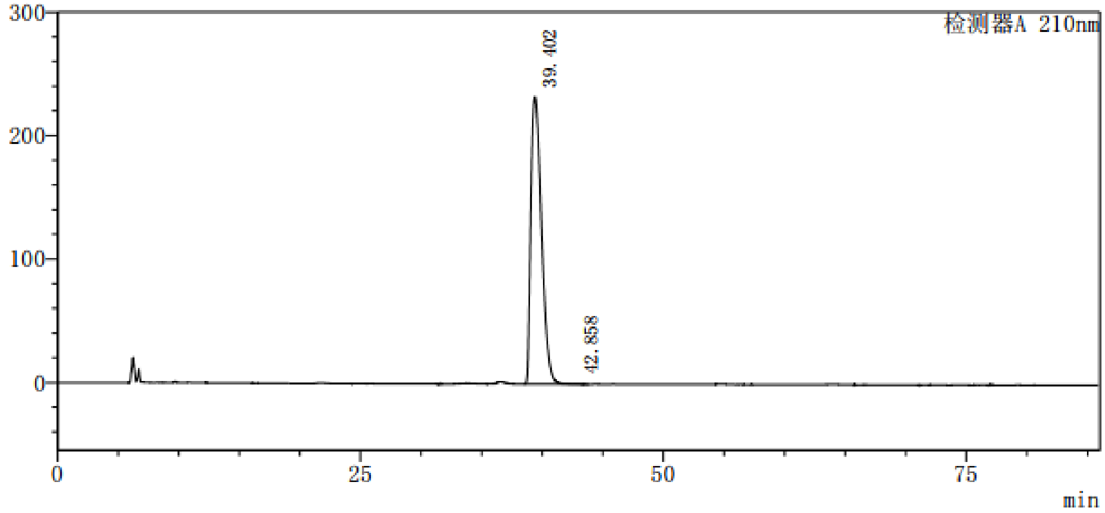

	Retention Time (min)	Area (%)	
**Peak 1**	39.402	99.972	>99% ee
**Peak 2**	42.858	0.028	

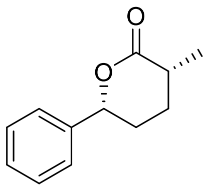

(*R*,*R*)-5a

HPLC conditions: Chiralcel OD-OJ Column, *n*-hexane/*i*-PrOH = 92/8, 210 nm, 1.0 mL/min, t_{major} = 61.897 min, t_{minor} = 36.489 min.

Racemate

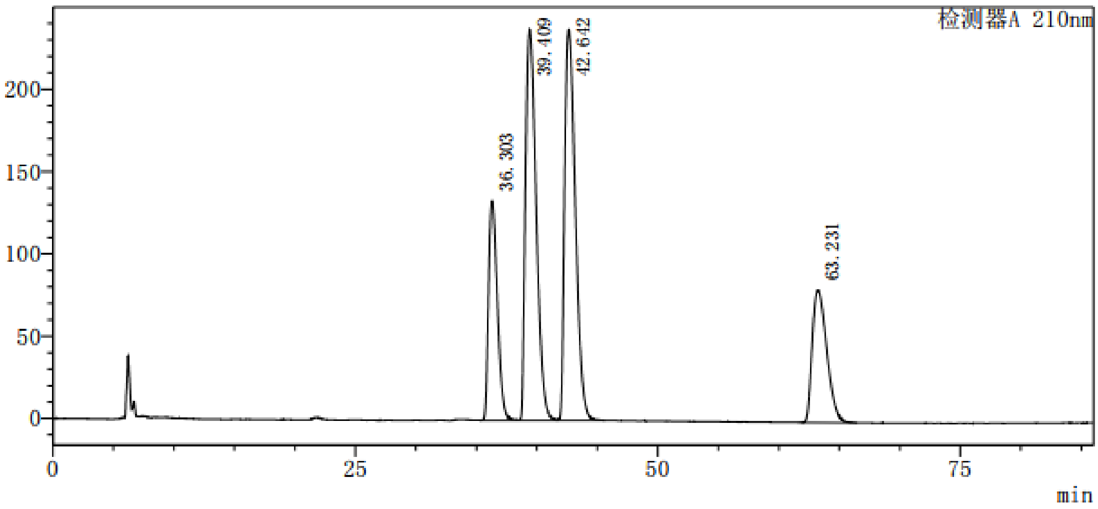

Chiral

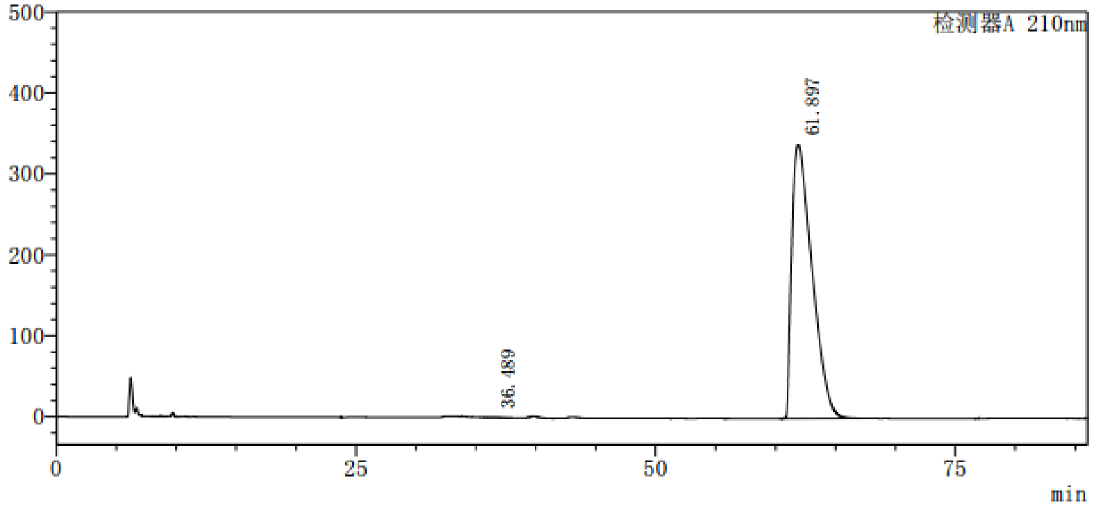

	Retention Time (min)	Area (%)	
**Peak 1**	36.489	0.037	>99% ee
**Peak 2**	61.897	99.963	

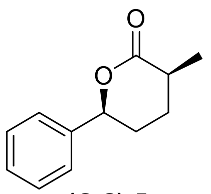

(*S*,*S*)-5a

HPLC conditions: Chiralcel OD-OJ Column, *n*-hexane/*i*-PrOH = 92/8, 210 nm, 1.0 mL/min, t_{major} = 36.017 min, t_{minor} = 62.544 min.

Racemate

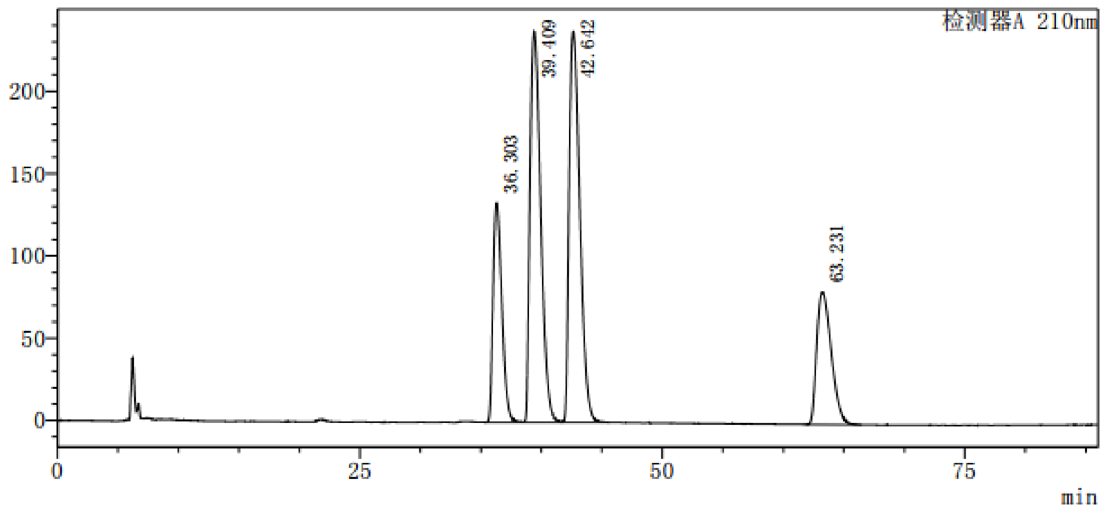

Chiral

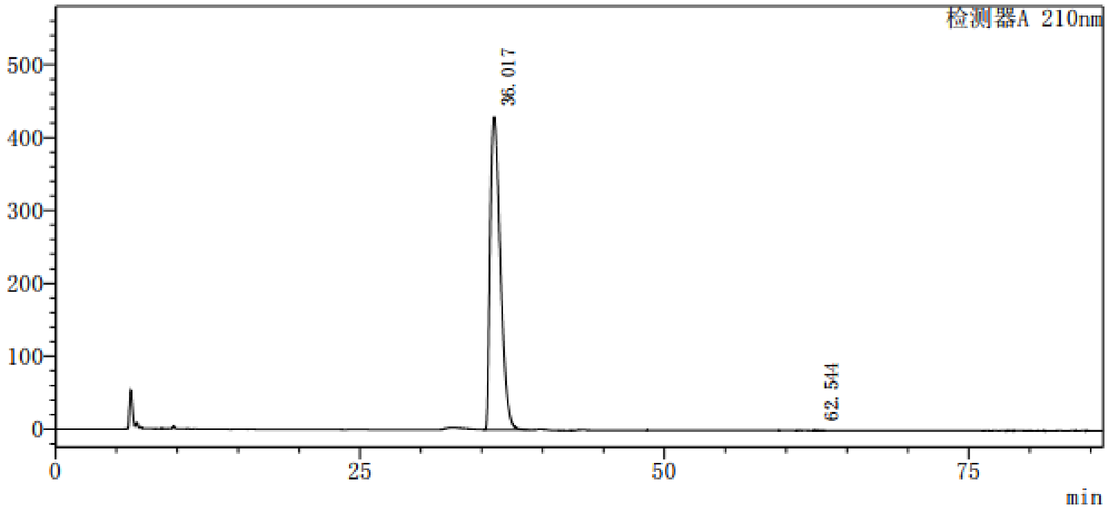

	Retention Time (min)	Area (%)	
**Peak 1**	36.017	99.988	>99% ee
**Peak 2**	62.544	0.012	

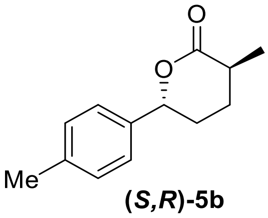

(*S*,*R*)-5b

HPLC conditions: Chiralpak AS Column, *n*-hexane/*i*-PrOH = 97/3, 210 nm, 0.7 mL/min, t_{major} = 41.125 min, t_{minor} = 70.700 min.

Racemate

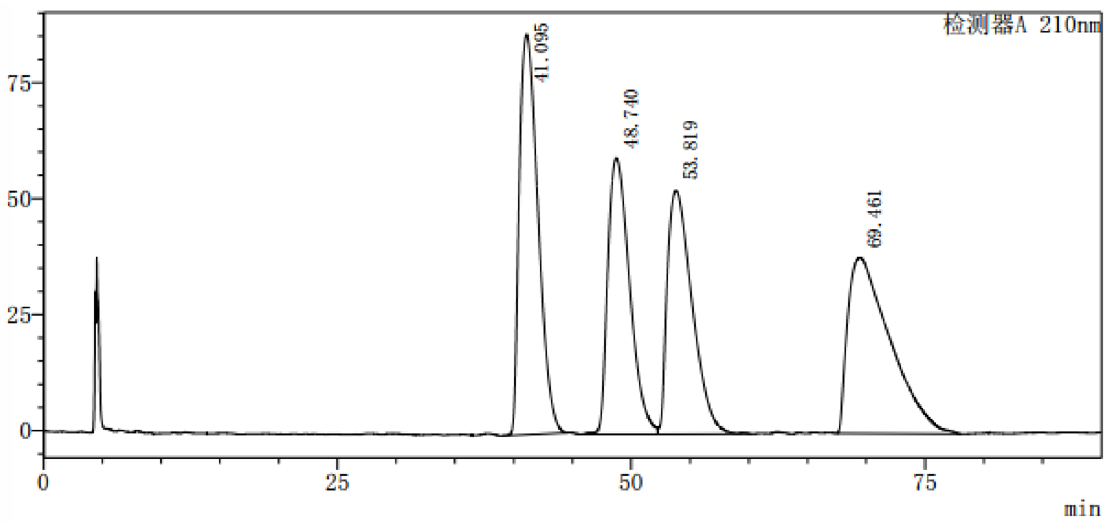

Chiral

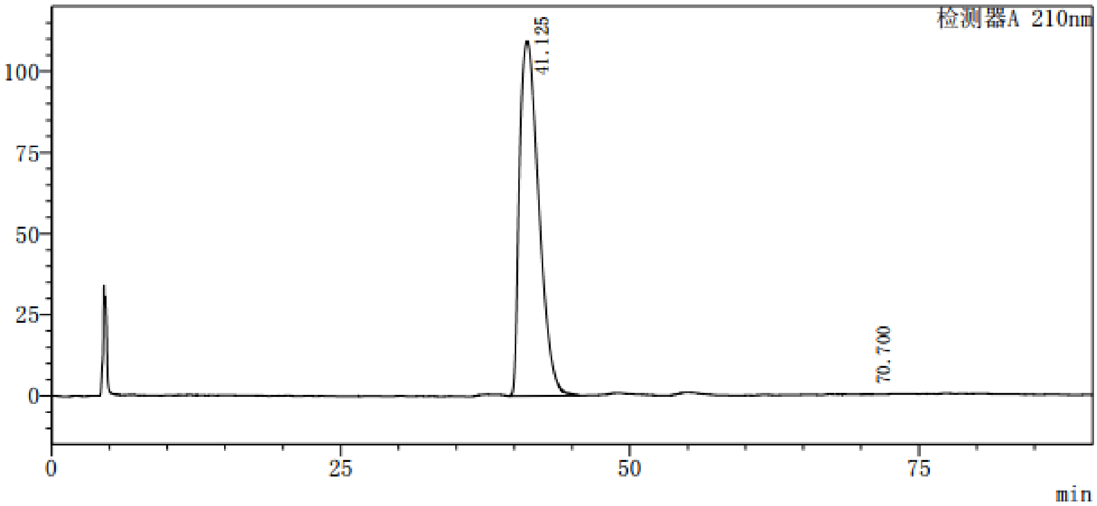

	Retention Time (min)	Area (%)	
**Peak 1**	41.125	99.979	>99% ee
**Peak 2**	70.700	0.021	

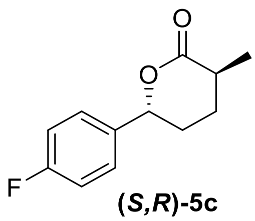

(*S*,*R*)-5c

HPLC conditions: Chiralcel OJ Column, *n*-hexane/*i*-PrOH = 95/5, 210 nm, 0.8 mL/min, t_{major} = 30.928 min, t_{minor} = 34.407 min.

Racemate

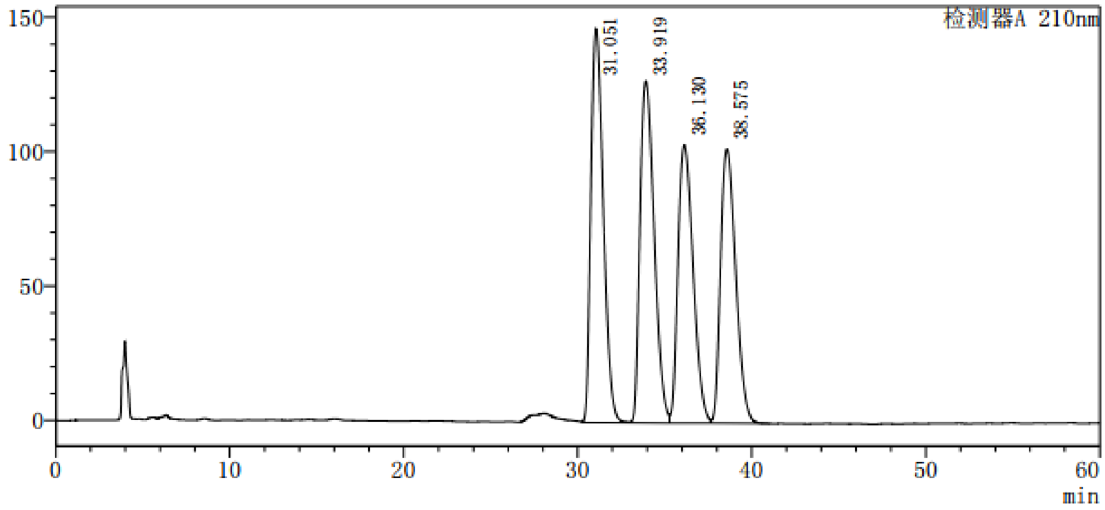

Chiral

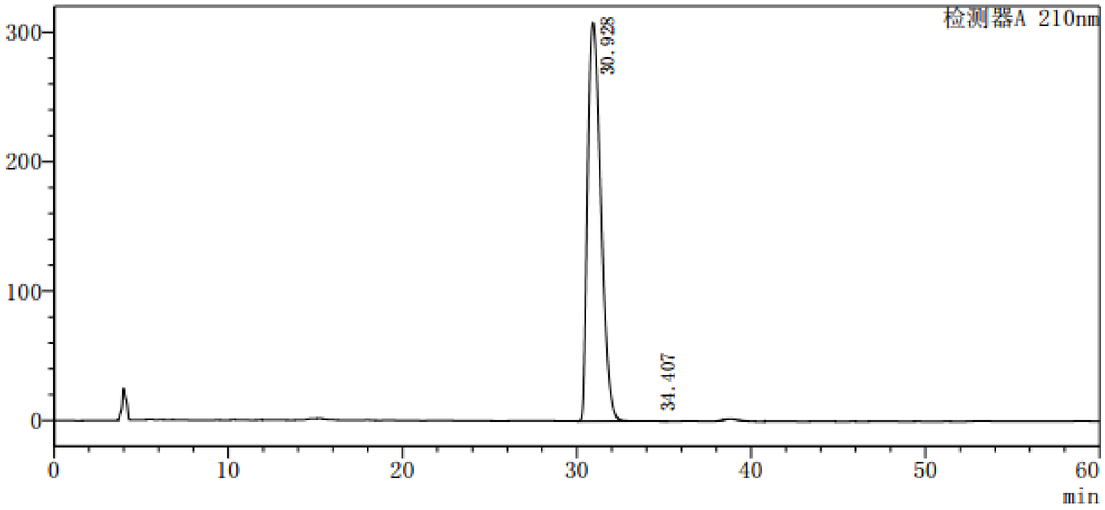

	Retention Time (min)	Area (%)	
**Peak 1**	30.928	99.995	>99% ee
**Peak 2**	34.407	0.005	

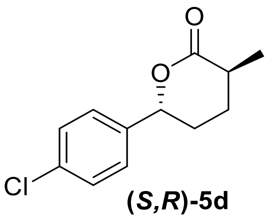

(*S*,*R*)-5d

HPLC conditions: Chiralpak AS Column, *n*-hexane/*i*-PrOH = 97/3, 210 nm, 0.7 mL/min, t_{major} = 51.115 min, t_{minor} = 73.926 min.

Racemate

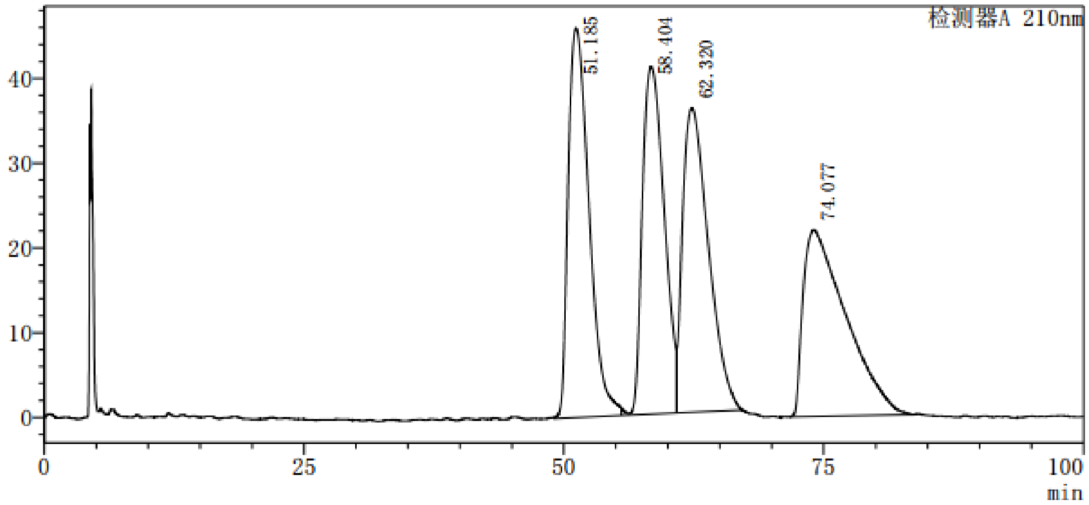

Chiral

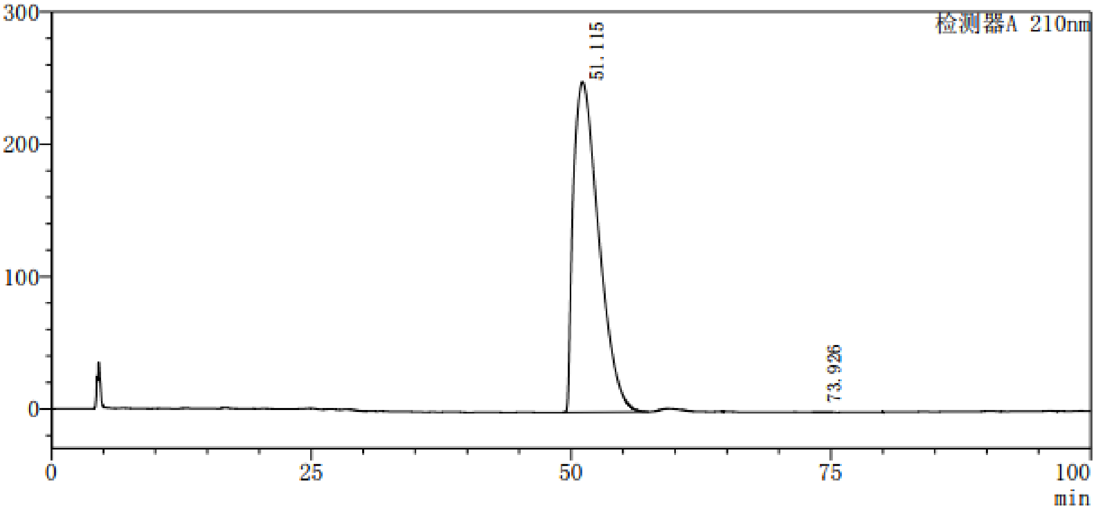

	Retention Time (min)	Area (%)	
**Peak 1**	51.115	99.984	>99% ee
**Peak 2**	73.926	0.016	

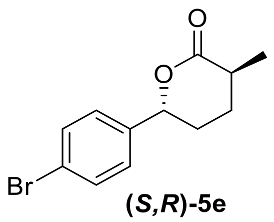

(*S*,*R*)-5e

HPLC conditions: Chiralpak AS Column, *n*-hexane/*i*-PrOH = 97/3, 210 nm, 0.7 mL/min, t_{major} = 54.604 min, t_{minor} = 75.215 min.

Racemate.

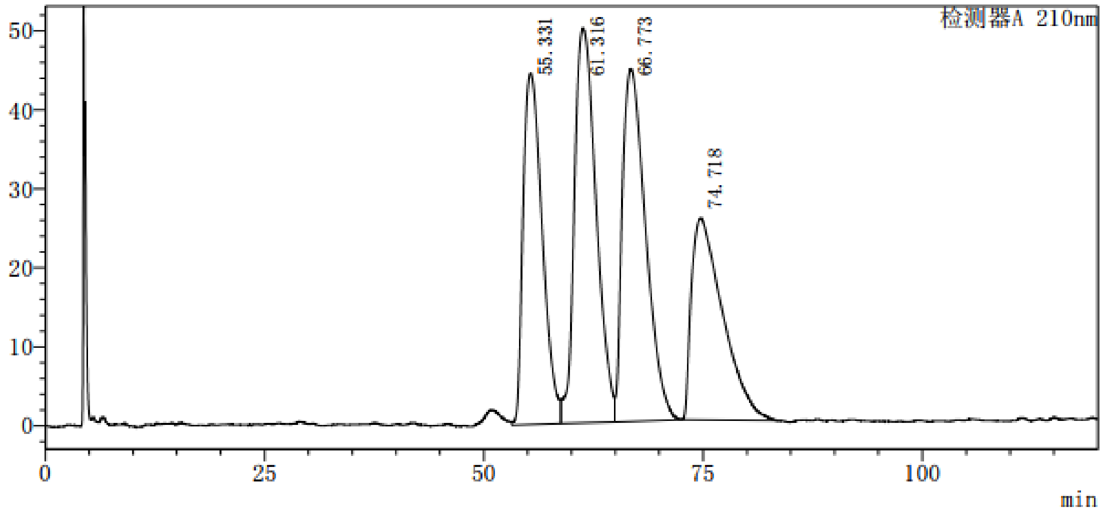

Chiral

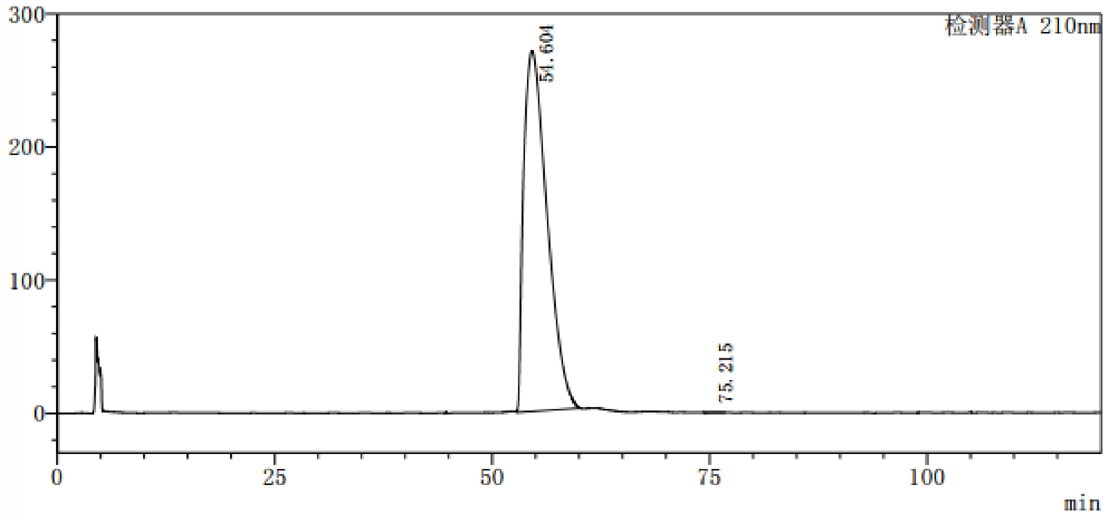

	Retention Time (min)	Area (%)	
**Peak 1**	54.604	99.981	>99% ee
**Peak 2**	75.215	0.019	

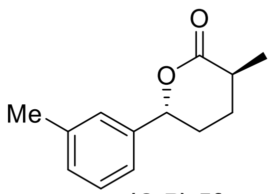

(*S*,*R*)-5f

HPLC conditions: Chiralcel OD-OD Column, *n*-hexane/*i*-PrOH = 97/3, 210 nm, 0.7 mL/min, t_{major} = 56.006 min, t_{minor} = 49.880 min.

Racemate

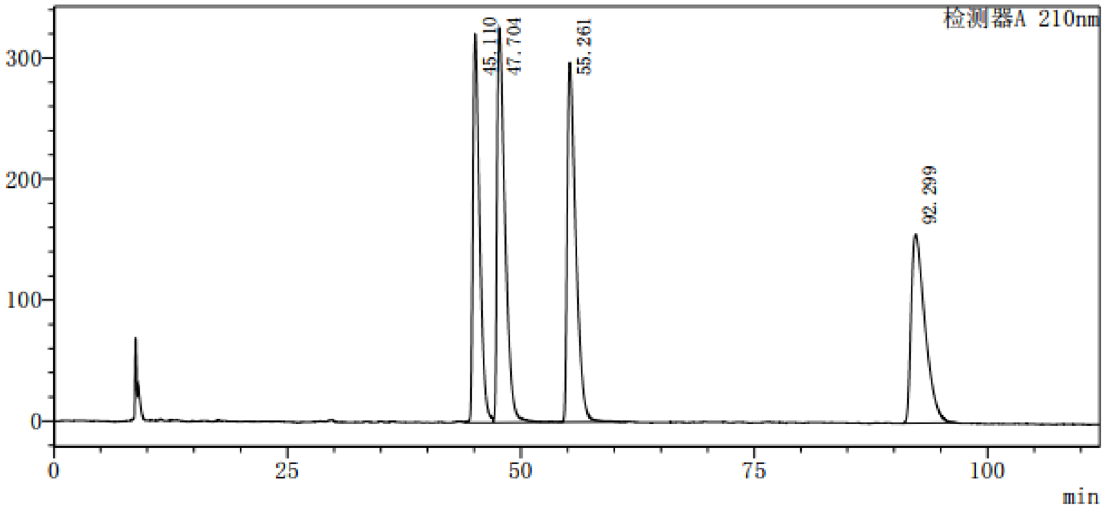

Chiral

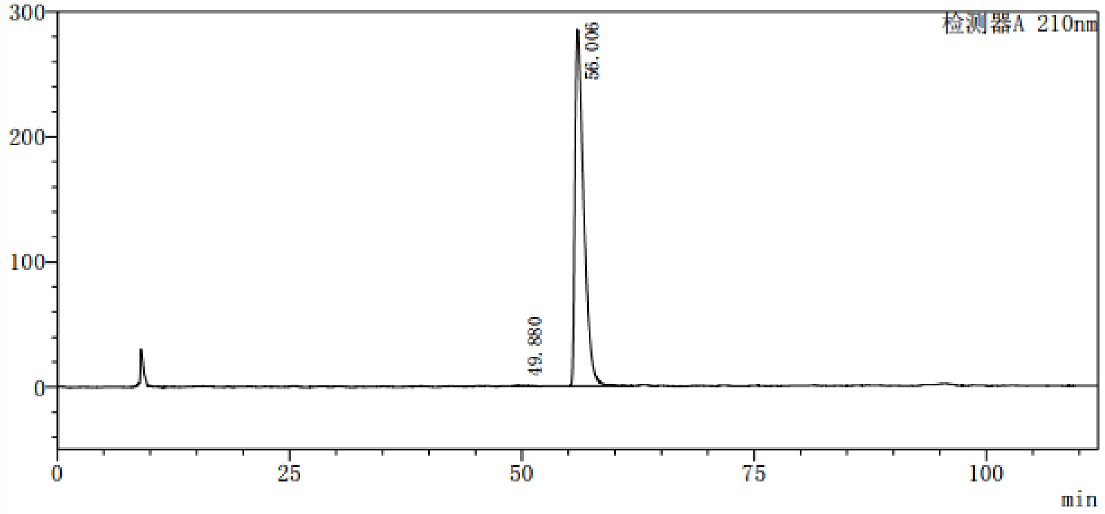

	Retention Time (min)	Area (%)	
**Peak 1**	49.880	0.270	>99% ee
**Peak 2**	56.006	99.730	

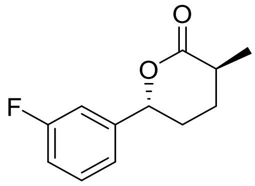

(*S*,*R*)-5g

HPLC conditions: Chiralcel OJ-OZ Column, *n*-hexane/*i*-PrOH = 97/3, 210 nm, 0.7 mL/min, t_{major} = 65.032 min, t_{minor} = 58.434 min.

Racemate

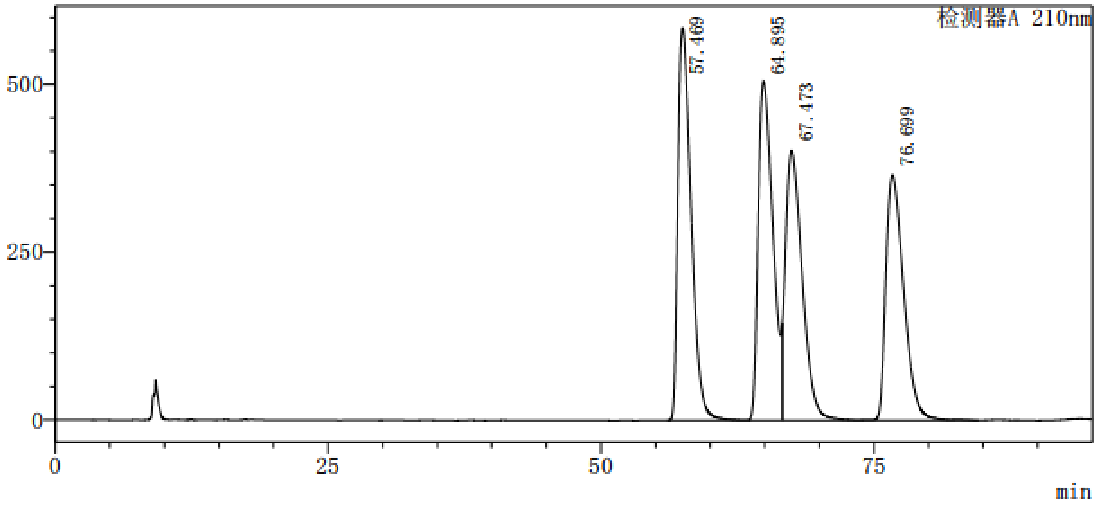

Chiral

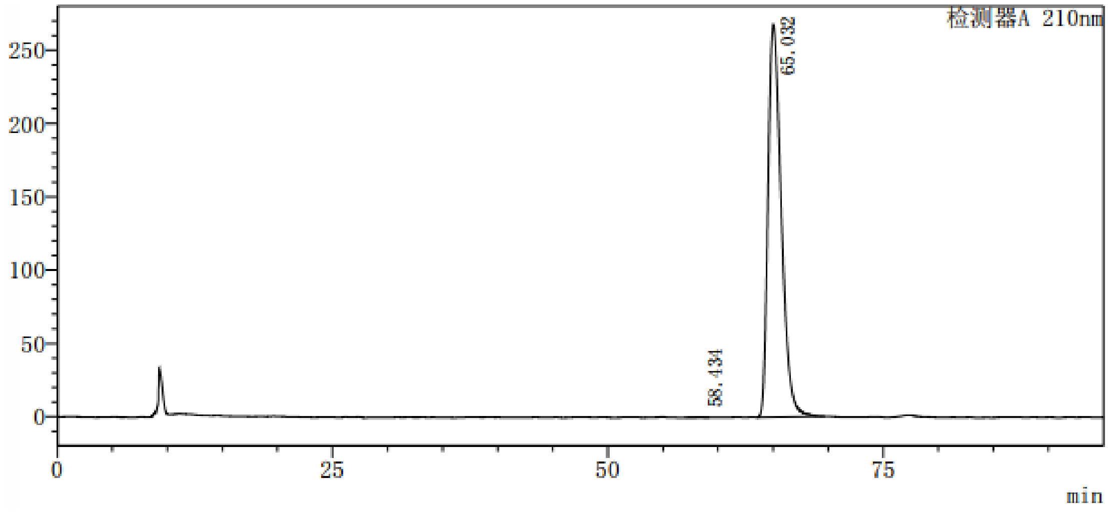

	Retention Time (min)	Area (%)	
**Peak 1**	58.434	0.046	>99% ee
**Peak 2**	65.032	99.954	

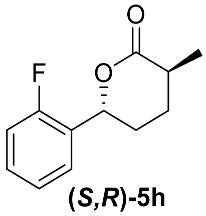

(*S*,*R*)-5h

HPLC conditions: Chiralcel OD Column, *n*-hexane/*i*-PrOH = 97/3, 210 nm, 0.7 mL/min, t_{major} = 16.335 min, t_{minor} = 15.639 min.

Racemate

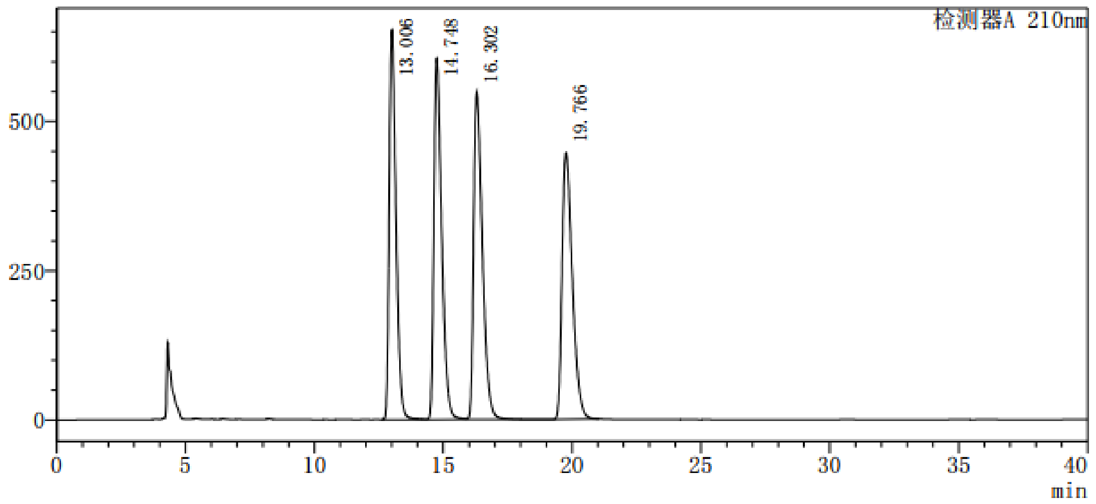

Chiral

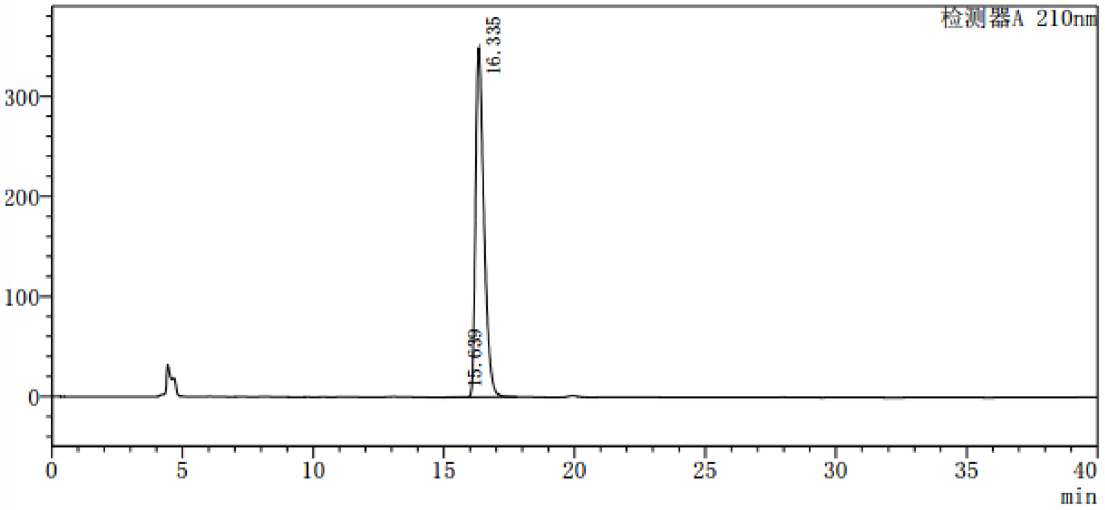

	Retention Time (min)	Area (%)	
**Peak 1**	15.639	0.034	>99% ee
**Peak 2**	16.335	99.966	

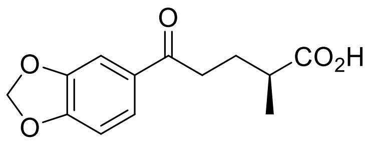

HPLC conditions: Chiralcel OJ Column, *n*-hexane/*i*-PrOH = 95/5, 210 nm, 1.0 mL/min, t_{major} = 11.957 min, t_{minor} = 22.943 min.

Racemate

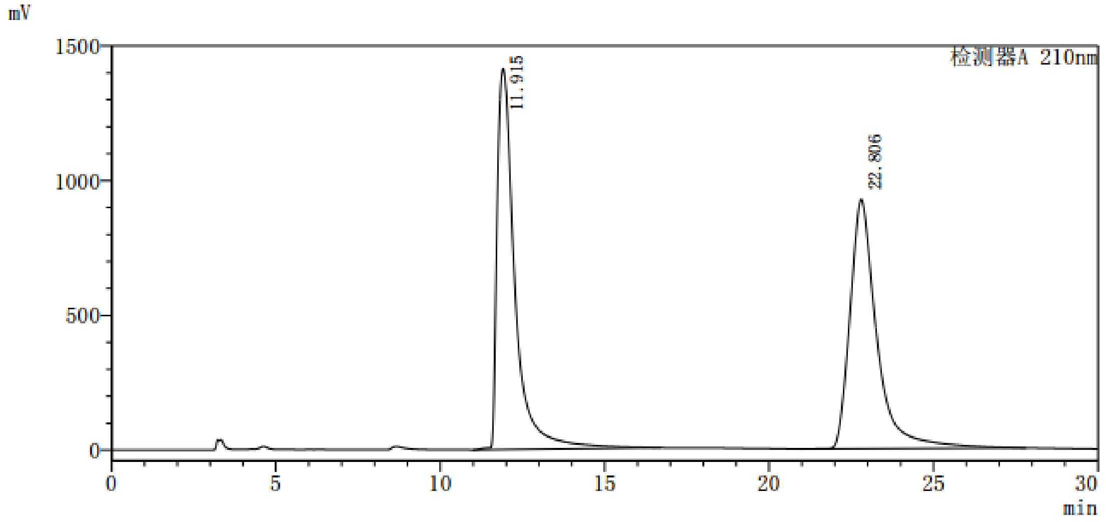

Chiral

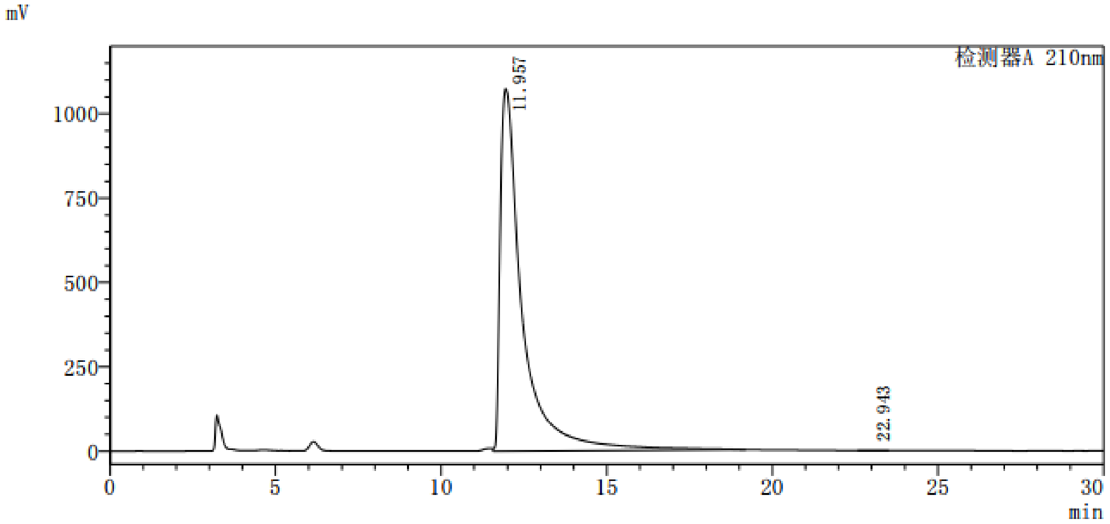

	Retention Time (min)	Area (%)	
**Peak 1**	11.957	99.987	>99% ee
**Peak 2**	22.943	0.013	

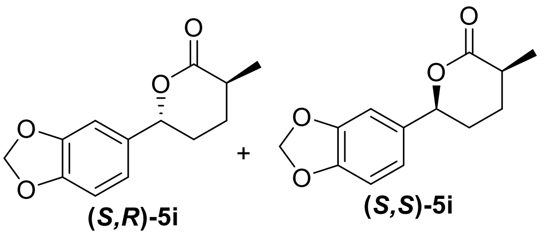

(*S*,*R*)-5i (*S*,*S*)-5i

HPLC conditions: Chiralcel OJ Column, *n*-hexane/*i*-PrOH = 89/11, 210 nm, 1.0 mL/min, **(*S*,*R*)-5i**: t_{major} = 50.444 min, t_{minor} = 65.631 min; **(*S*,*S*)-5i**: t_{major} = 39.008 min, t_{minor} = 36.060 min.

Racemate

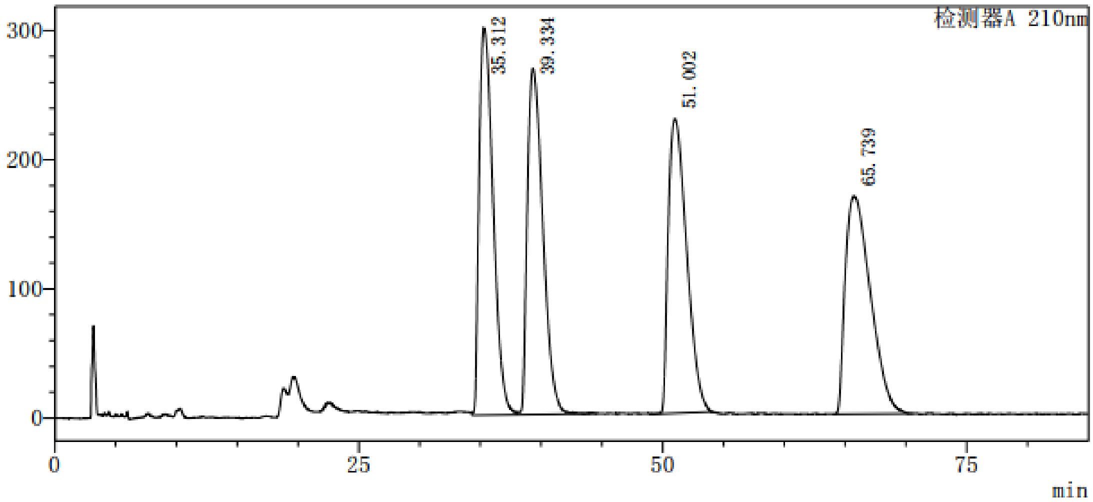

Chiral

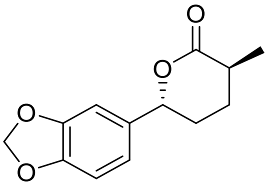

(*S*,*R*)-5i

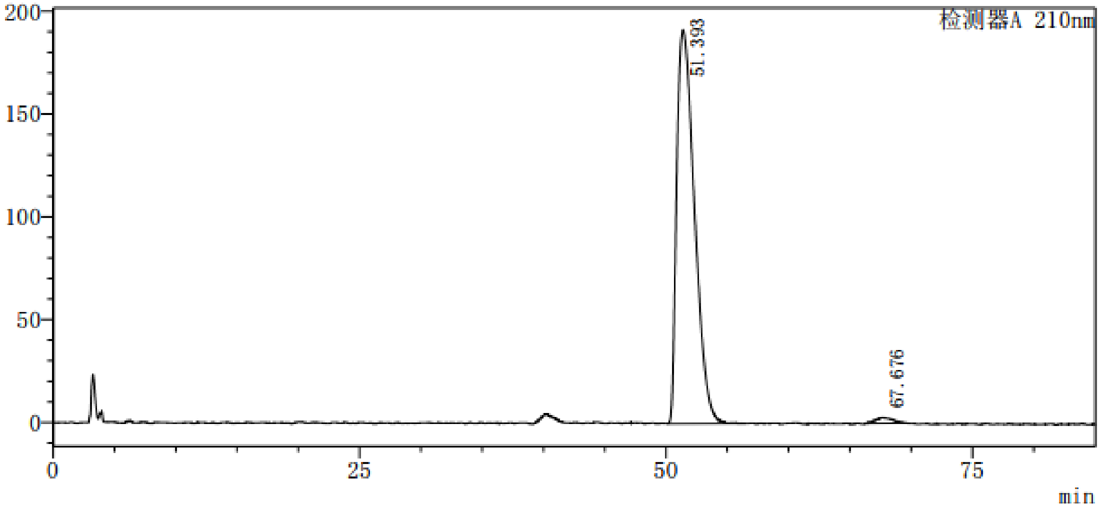

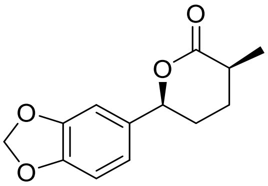

(*S*,*S*)-5i

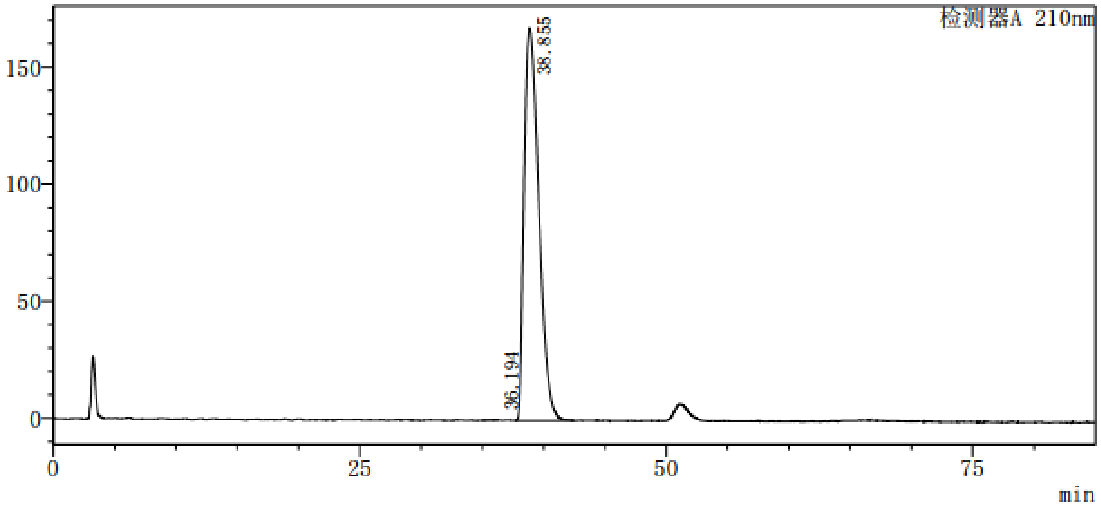

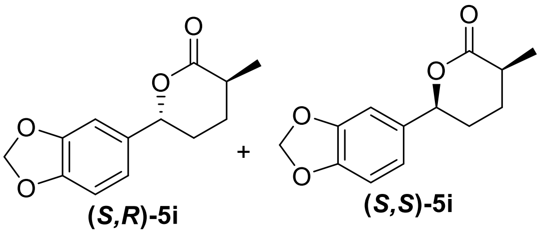

(*S*,*R*)-5i + (*S*,*S*)-5i

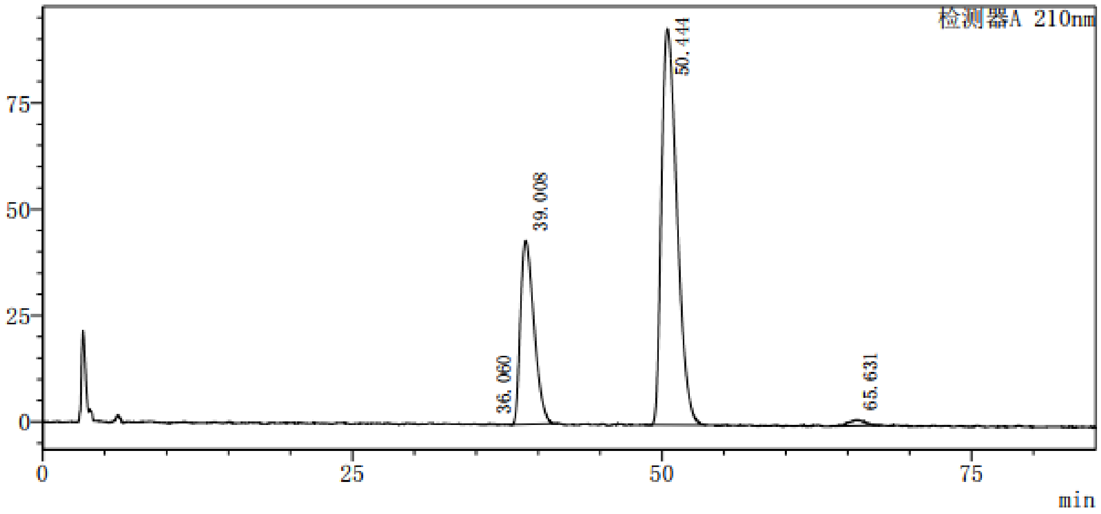

	Retention Time (min)	Area (%)	Ee (%)
**Peak 1**	36.060	0.048	**(*S*,*R*)-5i:** 97
**Peak 2**	39.008	28.337	(Peaks 3 and 4)
**Peak 3**	50.444	70.480	**(S,*S*)-5i:** >99
**Peak 4**	65.631	1.135	(Peaks 1 and 2)

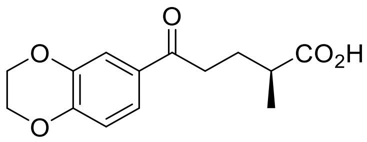

HPLC conditions: Chiralcel OJ Column, *n*-hexane/*i*-PrOH = 95/5, 210 nm, 1.0 mL/min, t_{major} = 10.970 min, t_{minor} = 21.975 min.

Racemate

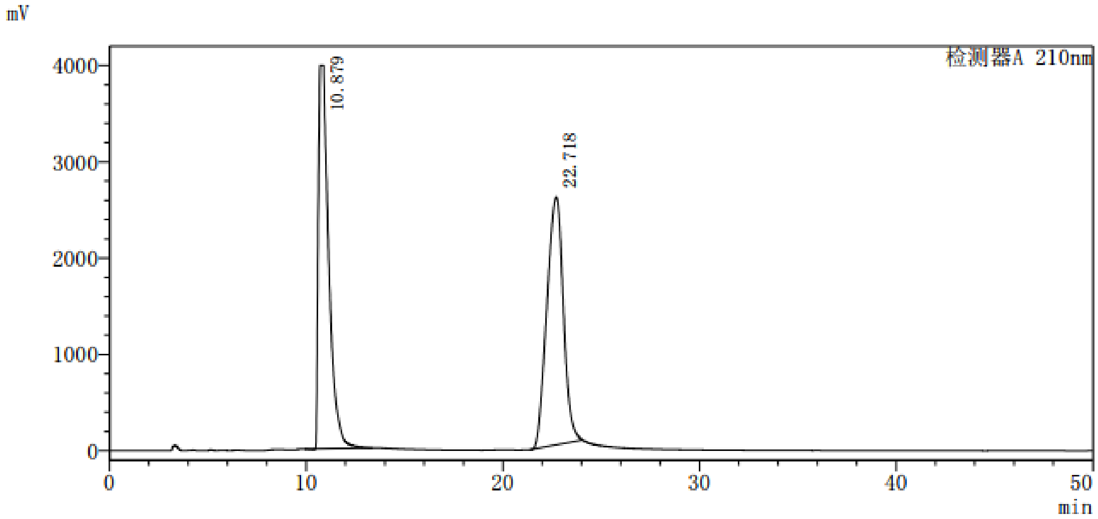

Chiral

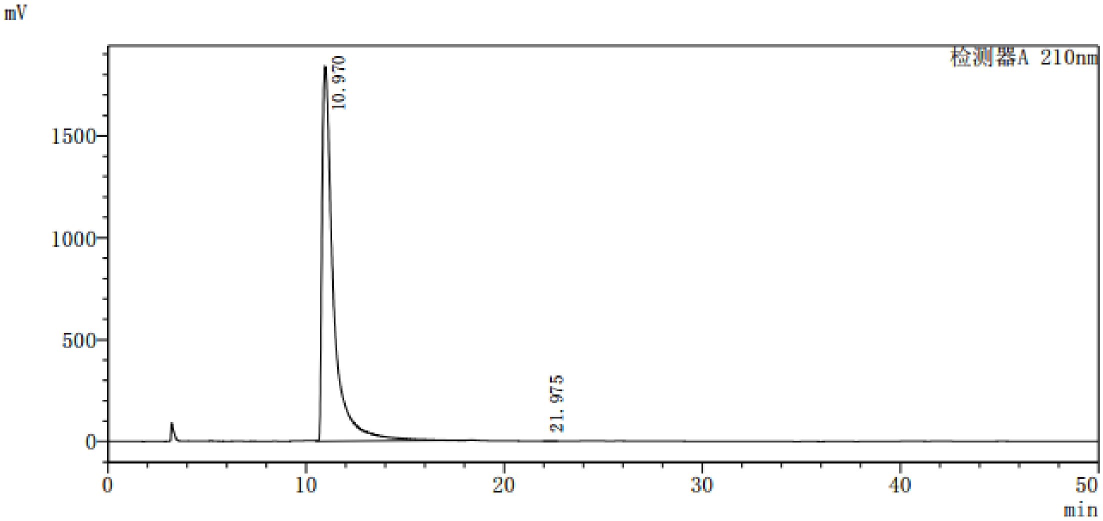

	Retention Time (min)	Area (%)	
**Peak 1**	10.970	99.985	>99% ee
**Peak 2**	21.975	0.015	

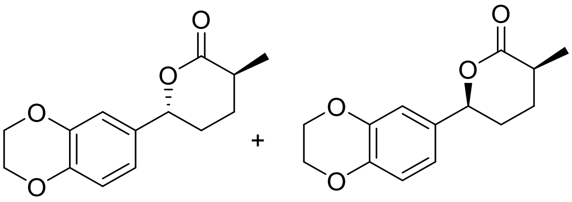

(*S*,*R*)-5j **(*S*,*S*)-5j**

HPLC conditions: Chiralcel OJ Column, *n*-hexane/*i*-PrOH = 89/11, 210 nm, 1.0 mL/min, **(*S*,*R*)-5j**: t_{major} = 66.152 min, t_{minor} = 46.897 min; **(*S*,*S*)-5j**: t_{major} = 54.405 min, t_{minor} = 71.899 min.

Racemate

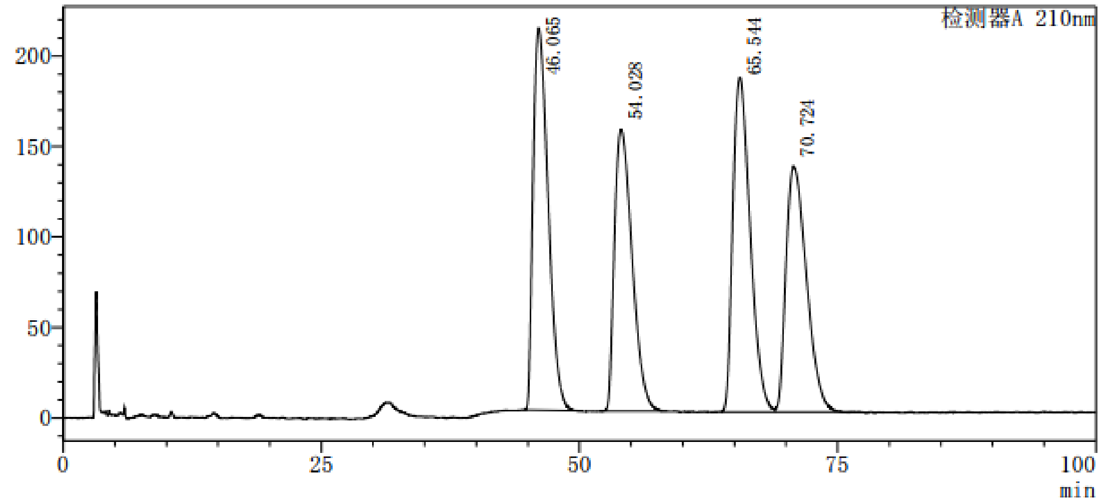

Chiral

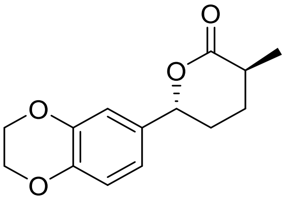

(*S*,*R*)-5j

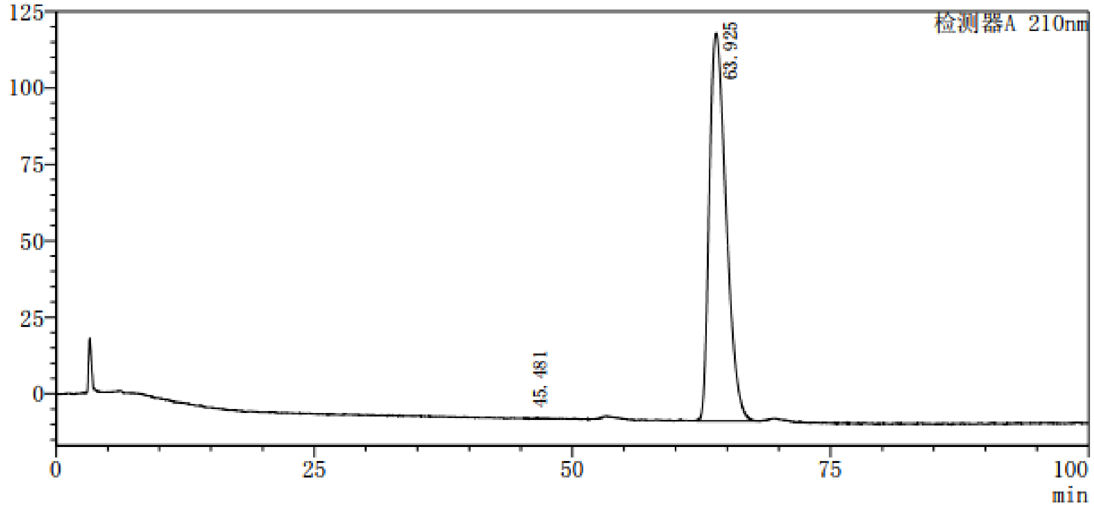

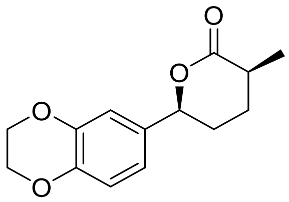

(*S*,*S*)-5j

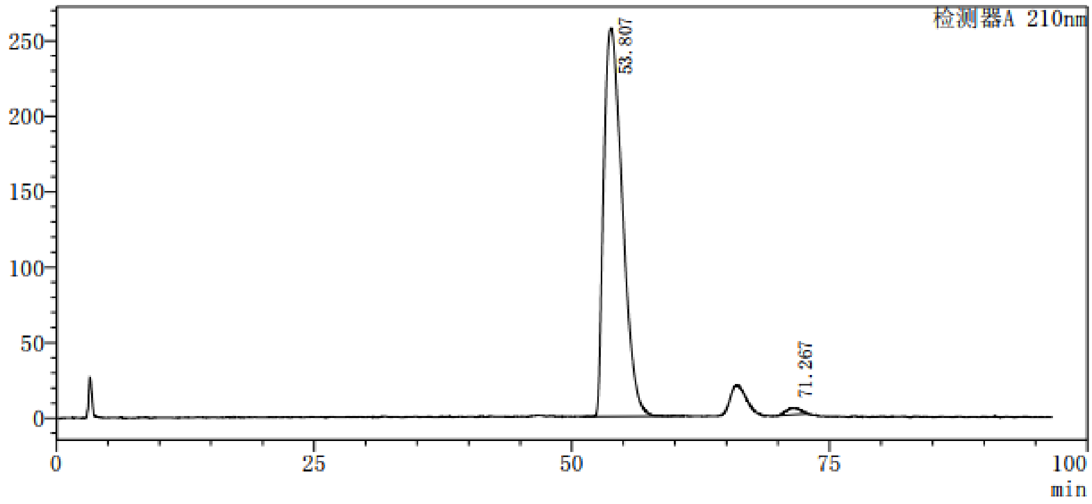

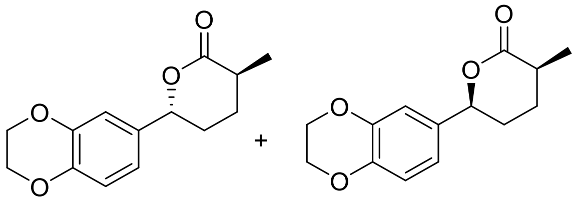

(*S*,*R*)-5j + **(*S*,*S*)-5j**

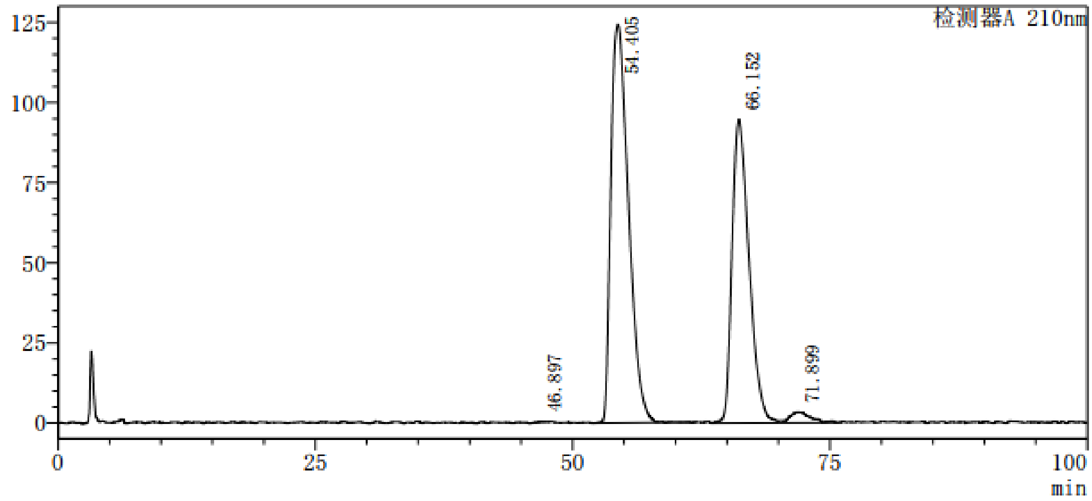

	Retention Time (min)	Area (%)	Ee (%)
**Peak 1**	46.897	0.057	**(*S*,*R*)-5j:** >99
**Peak 2**	54.405	56.584	(Peaks 1 and 3)
**Peak 3**	66.152	41.479	**(S,*S*)-5j:** 93
**Peak 4**	71.899	1.880	(Peaks 2 and 4)

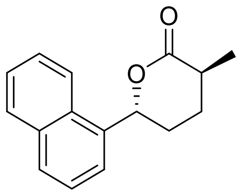

(*S*,*R*)-5k

HPLC conditions: Chiralcel OJ Column, *n*-hexane/*i*-PrOH = 95/5, 210 nm, 1.0 mL/min, t_{major} = 31.065 min, t_{minor} = 47.398 min.

Racemate

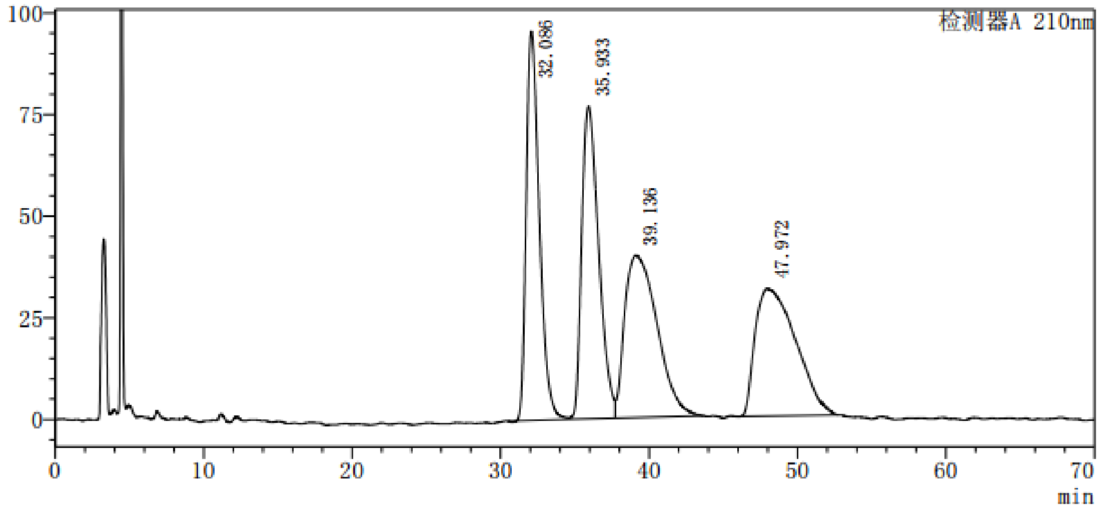

Chiral

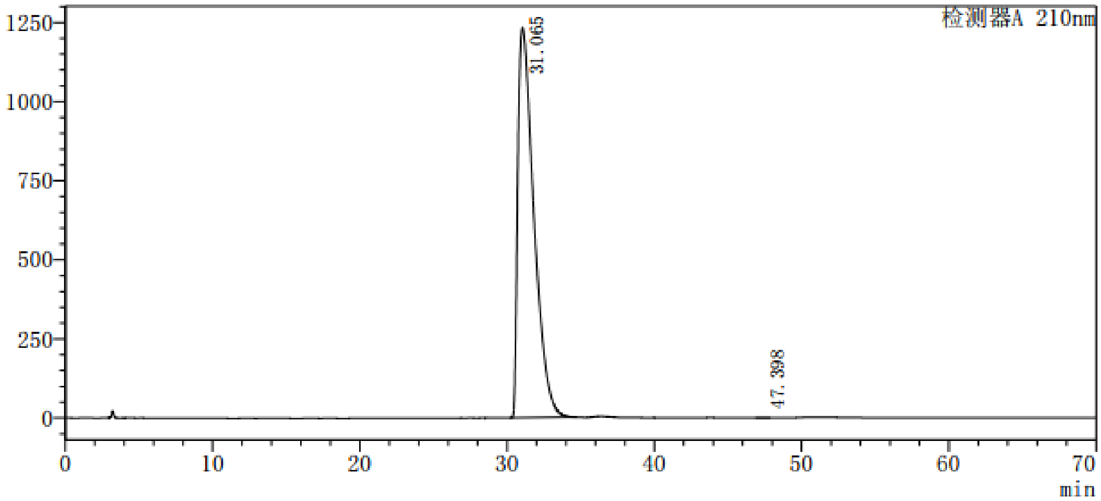

	Retention Time (min)	Area (%)	
**Peak 1**	31.065	99.995	>99% ee
**Peak 2**	47.398	0.005	

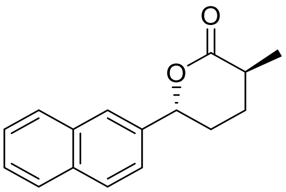

(*S*,*R*)-5l

HPLC conditions: Chiralpak IE Column, *n*-hexane/*i*-PrOH = 95/5, 210 nm, 1.0 mL/min, t_{major} = 49.468 min, t_{minor} = 59.271 min.

Racemate

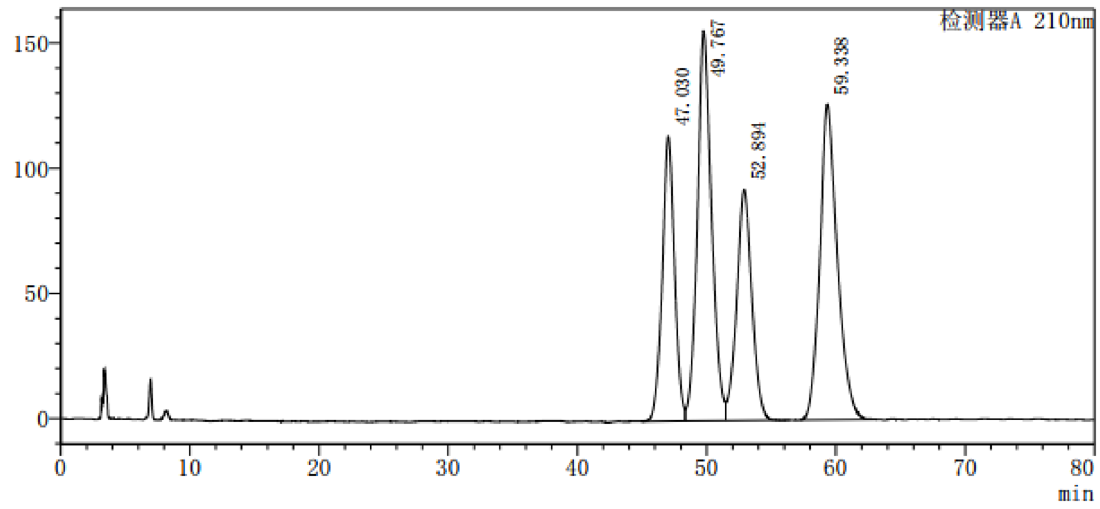

Chiral

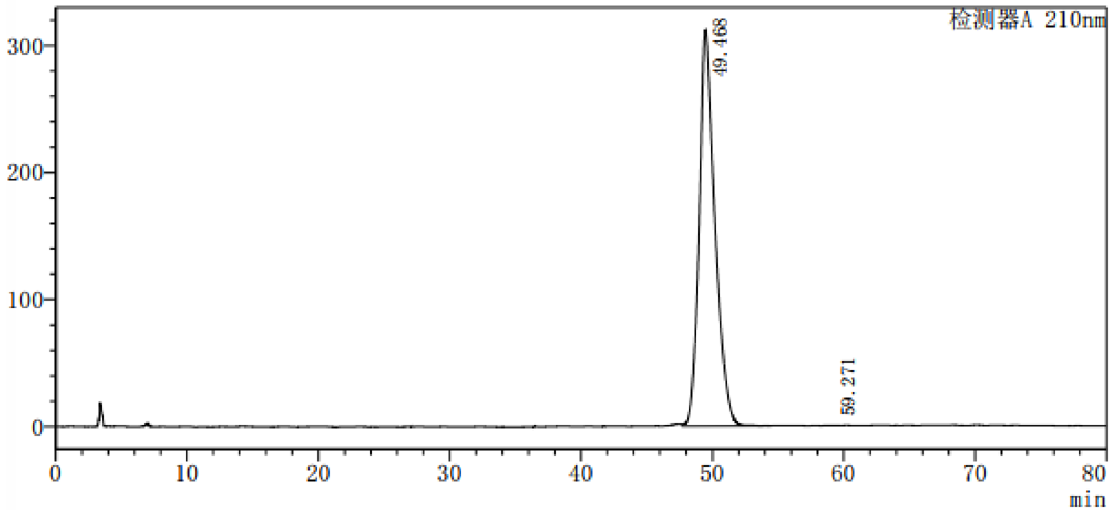

	Retention Time (min)	Area (%)	
**Peak 1**	49.468	99.980	>99% ee
**Peak 2**	59.271	0.020	

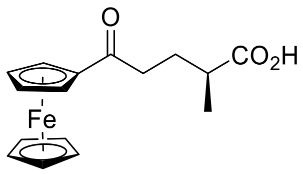

HPLC conditions: Chiralpak IE Column, *n*-hexane/*i*-PrOH = 90/10, 210 nm, 1.0 mL/min, t_{major} = 22.907 min, t_{minor} = 22.320 min.

Racemate

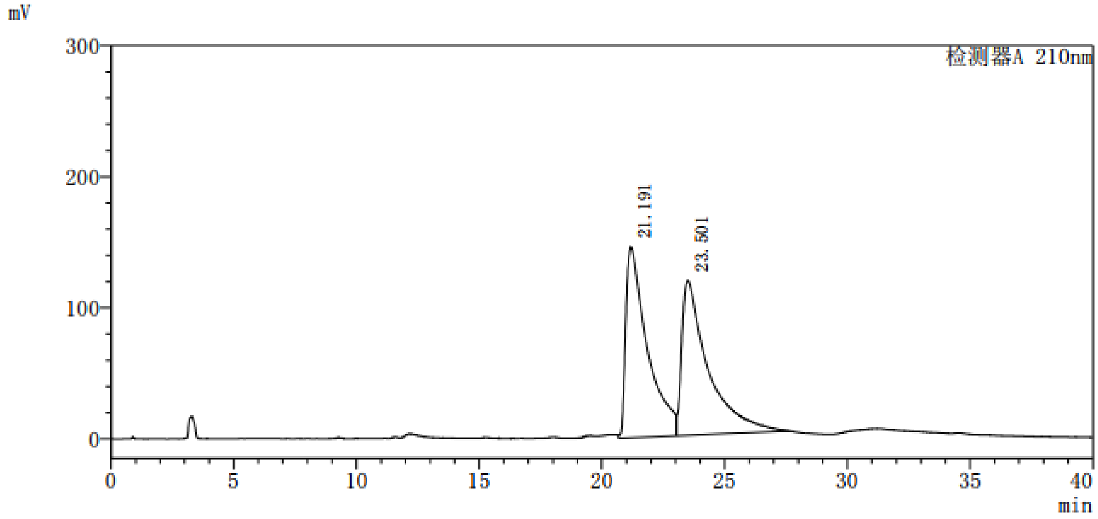

Chiral

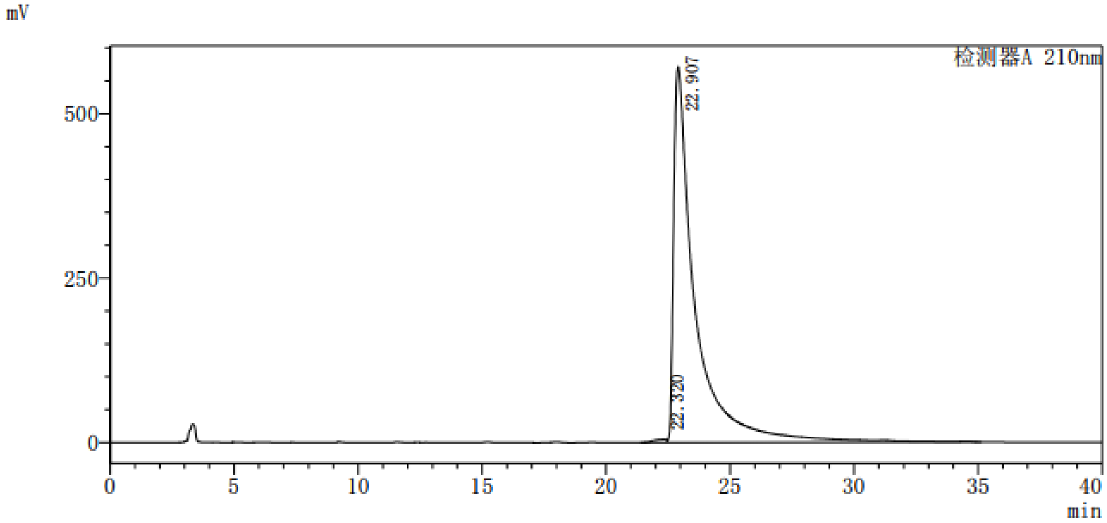

	Retention Time (min)	Area (%)	
**Peak 1**	22.320	0.475	>99% ee
**Peak 2**	22.907	99.525	

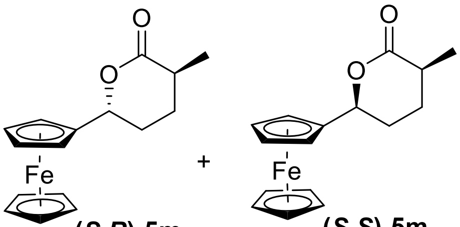

(*S*,*R*)-5m + (*S*,*S*)-5m

HPLC conditions: Chiralcel OZ Column, *n*-hexane/*i*-PrOH = 90/10, 210 nm, 1.0 mL/min, **(*S*,*R*)-5m**: t_{major} = 16.919 min, t_{minor} = 19.951 min; **(*S*,*S*)-5m**: t_{major} = 21.448 min, t_{minor} = 25.360 min.

Racemate

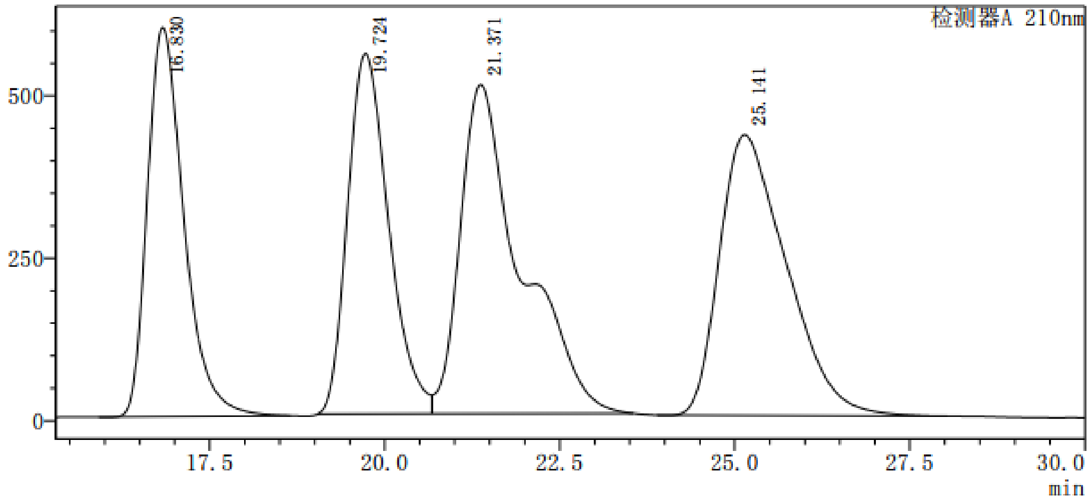

Chiral

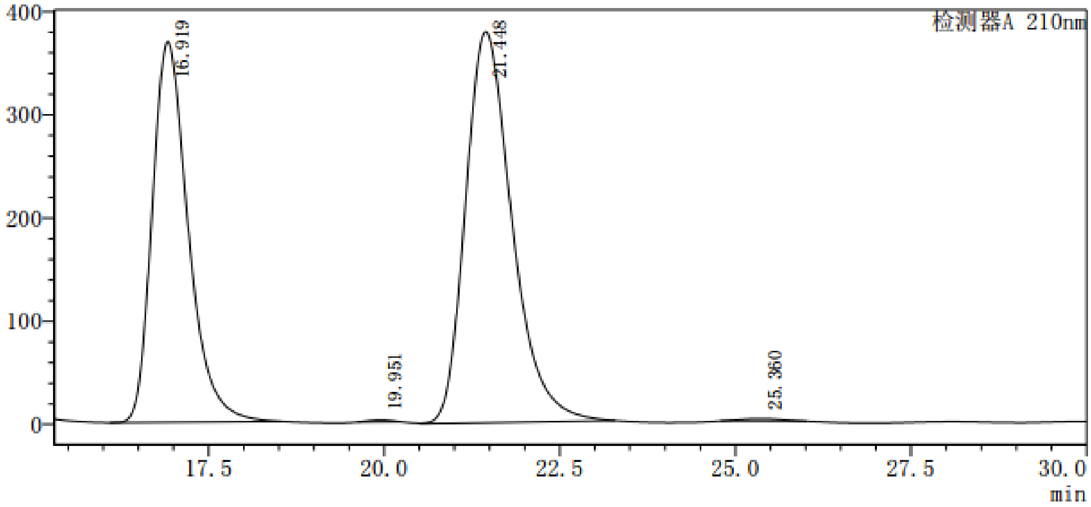

	Retention Time (min)	Area (%)	Ee (%)
**Peak 1**	16.919	42.767	**(*S*,*R*)-5m:** >99
**Peak 2**	19.951	0.128	(Peaks 1 and 2)
**Peak 3**	21.448	56.792	**(S,*S*)-5m:** 99
**Peak 4**	25.360	0.312	(Peaks 3 and 4)

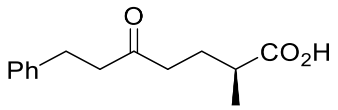

HPLC conditions: Chiralcel OJ Column, *n*-hexane/*i*-PrOH = 90/10, 210 nm, 1.0 mL/min, t_{major} = 16.204 min, t_{minor} = 13.927 min.

Racemate

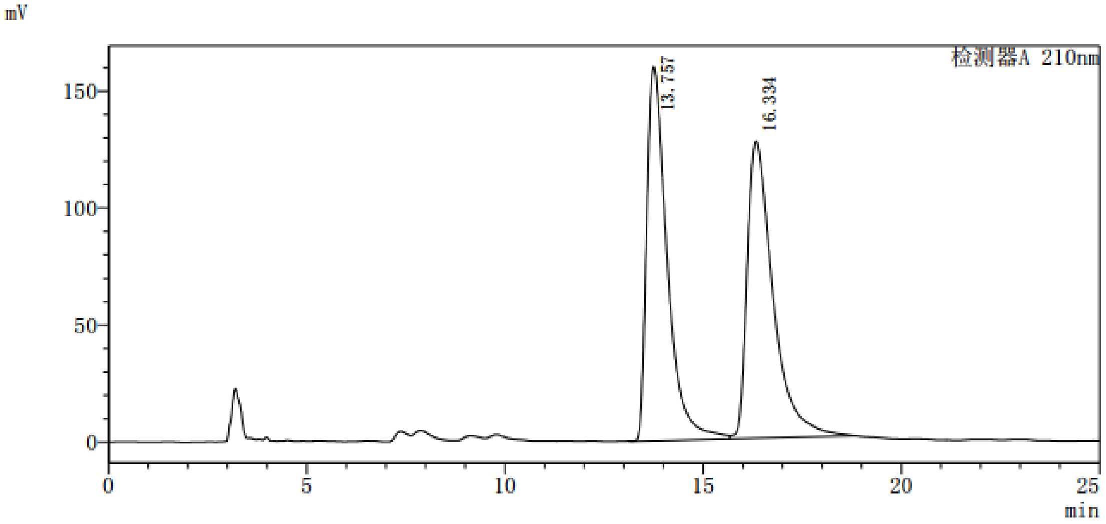

Chiral

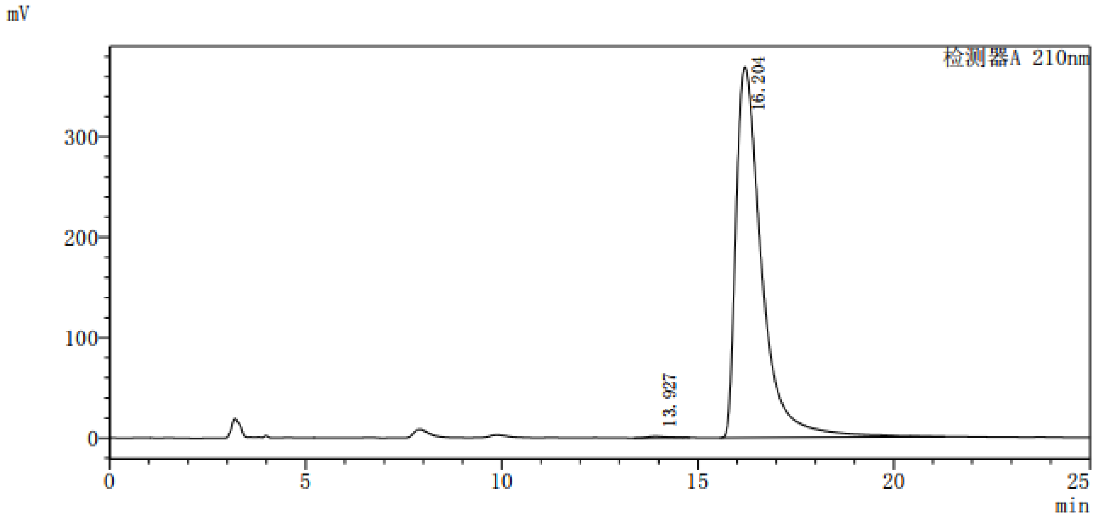

	Retention Time (min)	Area (%)	
**Peak 1**	13.927	0.308	>99% ee
**Peak 2**	16.204	99.962	

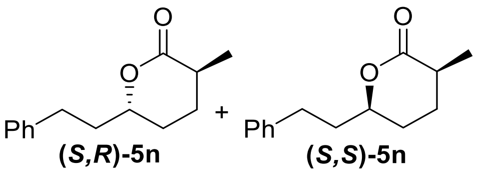

(*S*,*R*)-5n (*S*,*S*)-5n

HPLC conditions: Chiralcel OJ Column, *n*-hexane/*i*-PrOH = 90/10, 210 nm, 1.0 mL/min, **(*S*,*R*)-5n**: t_{major} = 22.804 min, t_{minor} = 13.543 min; **(*S*,*S*)-5n**: t_{major} = 15.791 min, t_{minor} = 17.198 min.

Racemate

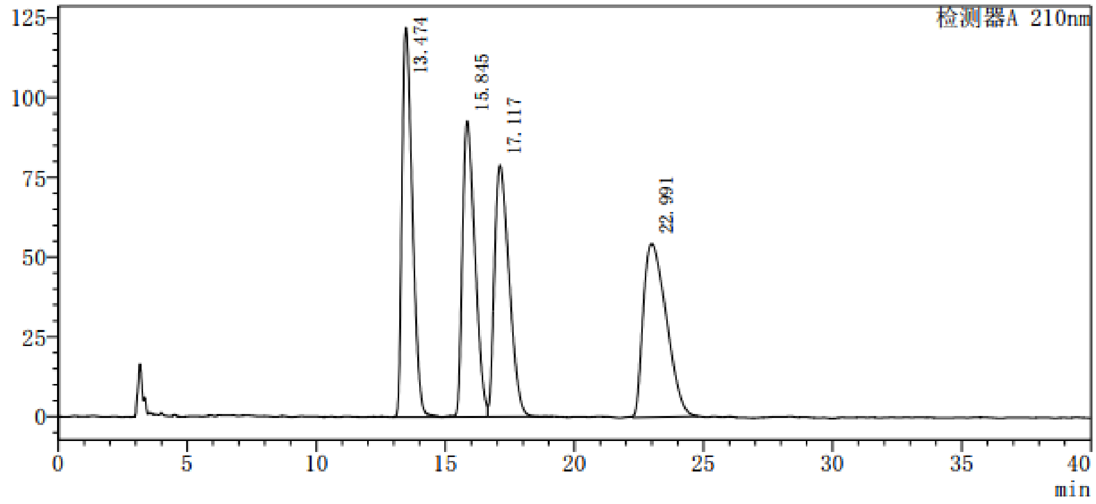

Chiral

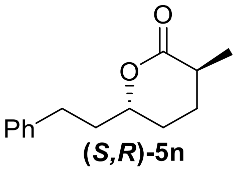

(*S*,*R*)-5n

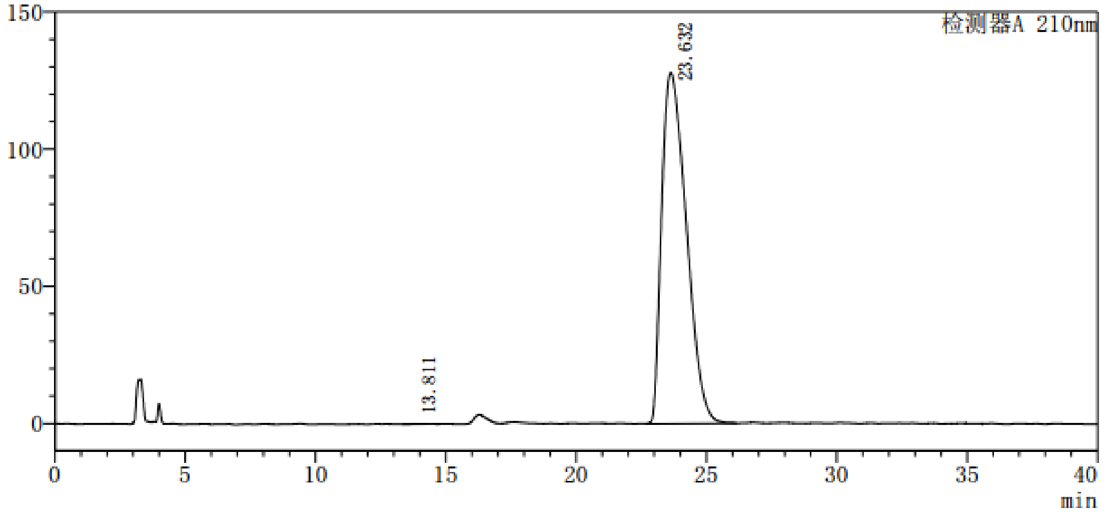

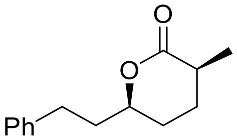

(*S*,*S*)-5n

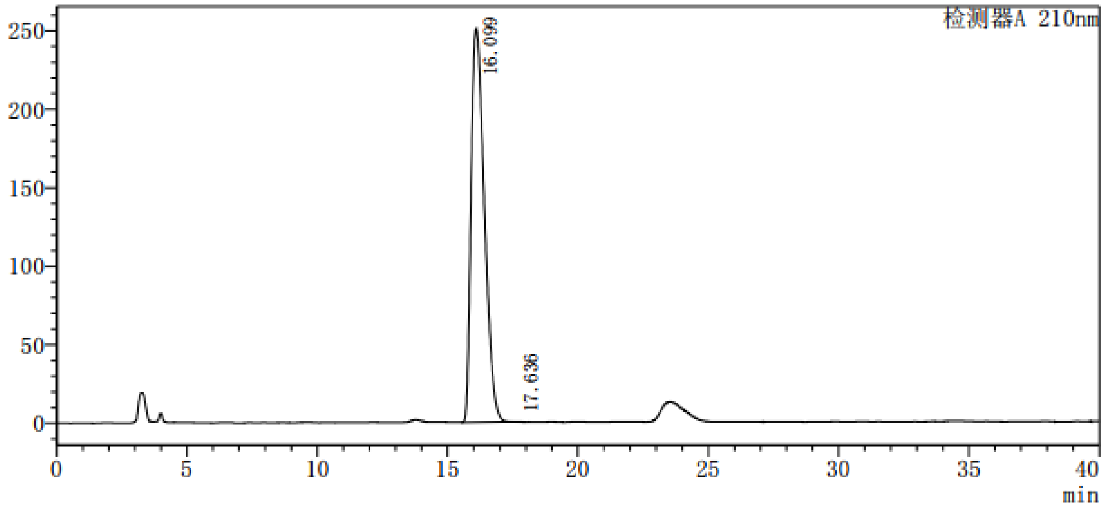

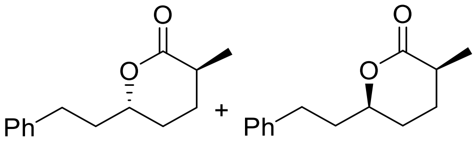

(*S*,*R*)-5n + (*S*,*S*)-5n

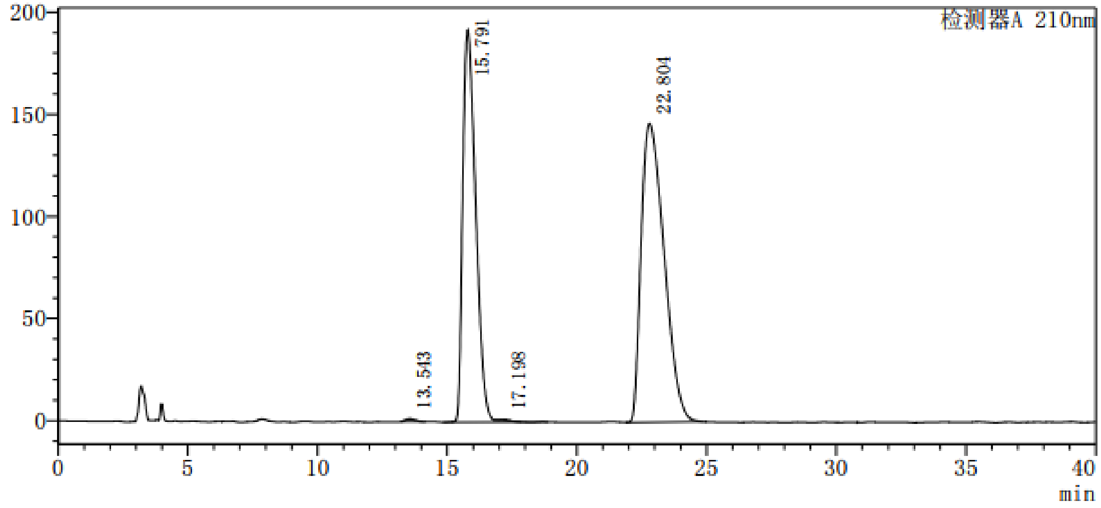

	Retention Time (min)	Area (%)	Ee (%)
**Peak 1**	13.543	0.228	**(*S*,*R*)-5n:** >99
**Peak 2**	15.791	42.470	(Peaks 1 and 4)
**Peak 3**	17.198	0.065	**(S,*S*)-5n:** >99
**Peak 4**	22.804	57.237	(Peaks 2 and 3)

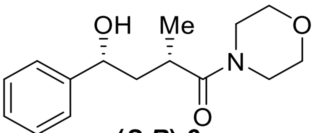

(*S*,*R*)-6

HPLC conditions: Chiralpak AD Column, *n*-hexane/*i*-PrOH = 90/10, 210 nm, 1.0 mL/min, t_{major} = 17.101 min, t_{minor} = 10.694 min.

Racemate

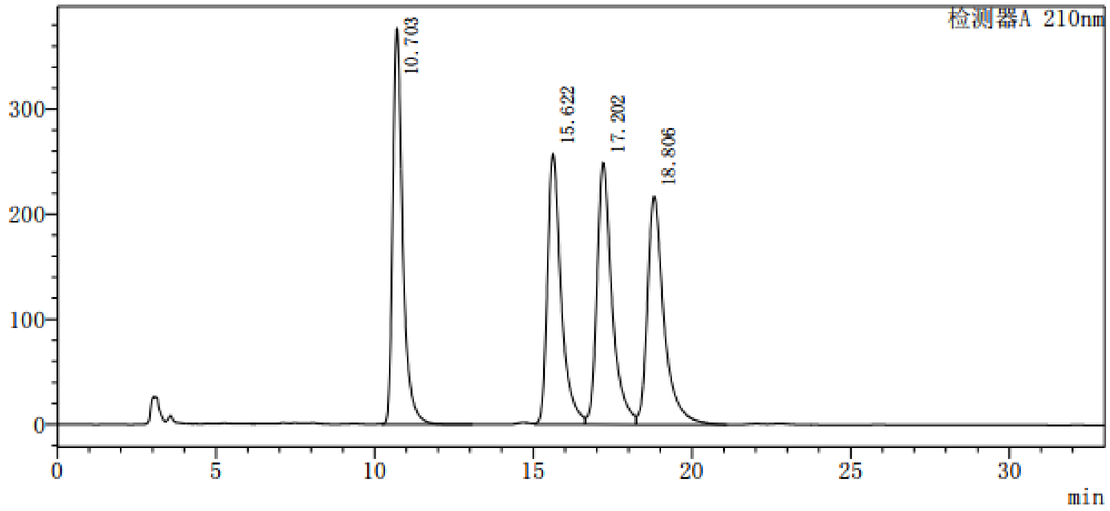

Chiral

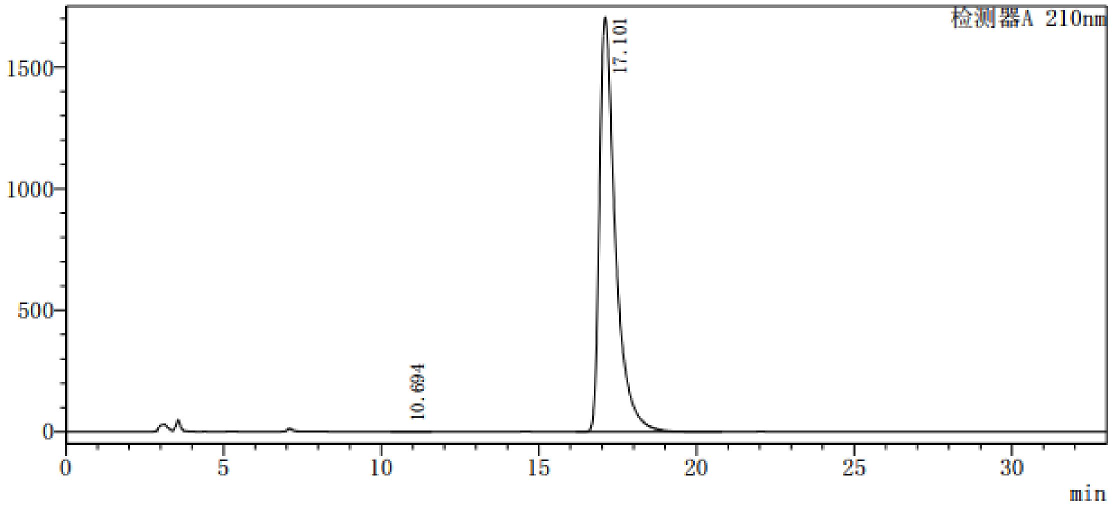

	Retention Time (min)	Area (%)	
**Peak 1**	10.694	0.014	>99% ee
**Peak 2**	17.101	99.986	

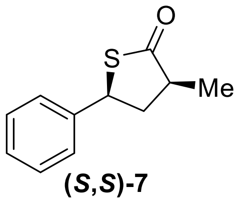

(*S*,*S*)-7

HPLC conditions: Chiralpak IC Column, *n*-hexane/*i*-PrOH = 97/3, 210 nm, 0.8 mL/min, t_{major} = 19.169 min, t_{minor} = 15.570 min.

Racemate

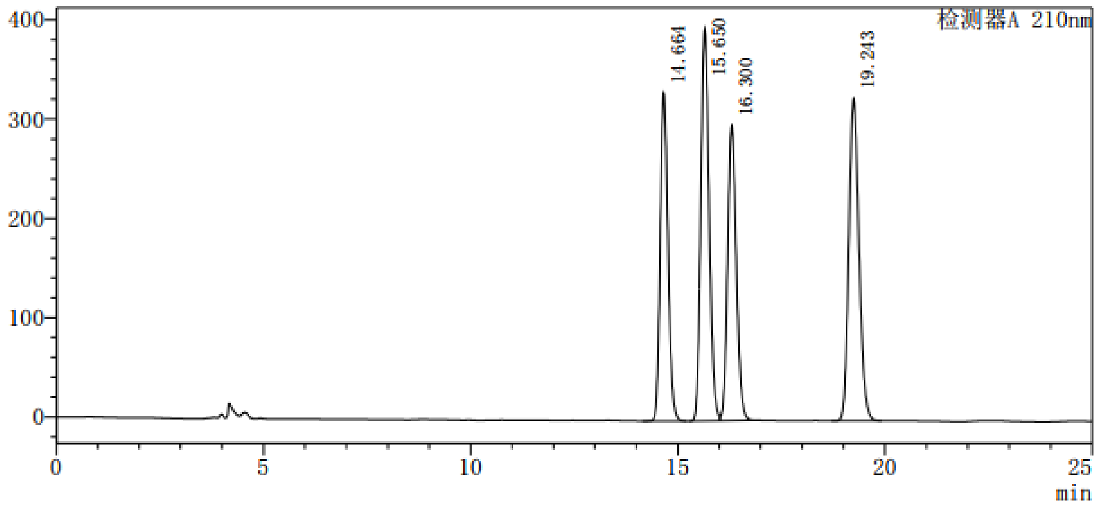

Chiral

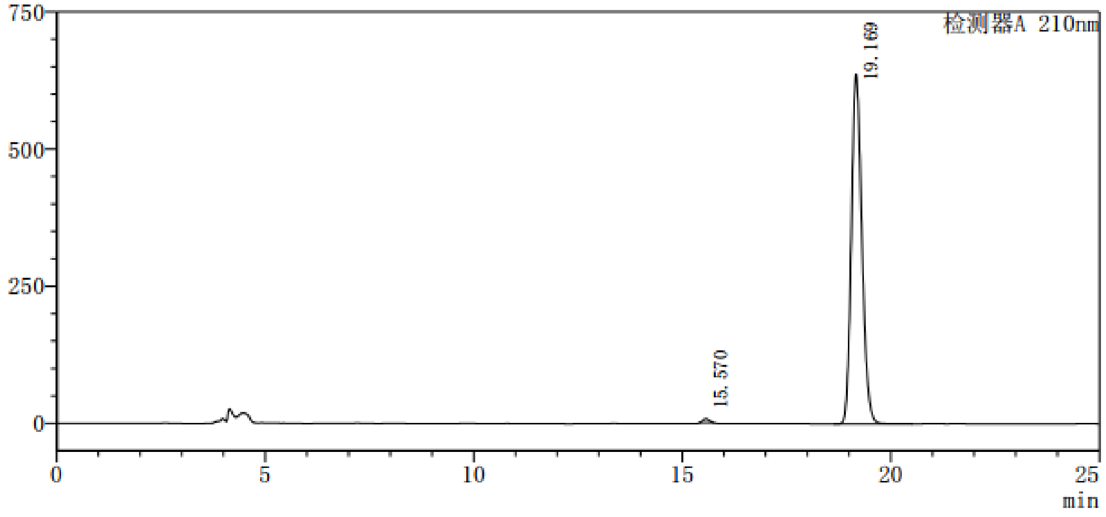

	Retention Time (min)	Area (%)	
**Peak 1**	15.570	0.757	99% ee
**Peak 2**	19.169	99.243	

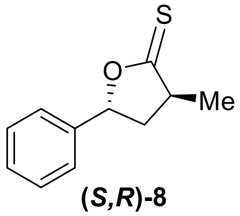

(*S*,*R*)-8

HPLC conditions: Chiralpak IC Column, *n*-hexane/*i*-PrOH = 97/3, 210 nm, 0.6 mL/min, t_{major} = 17.542 min, t_{minor} = 21.337 min.

Racemate

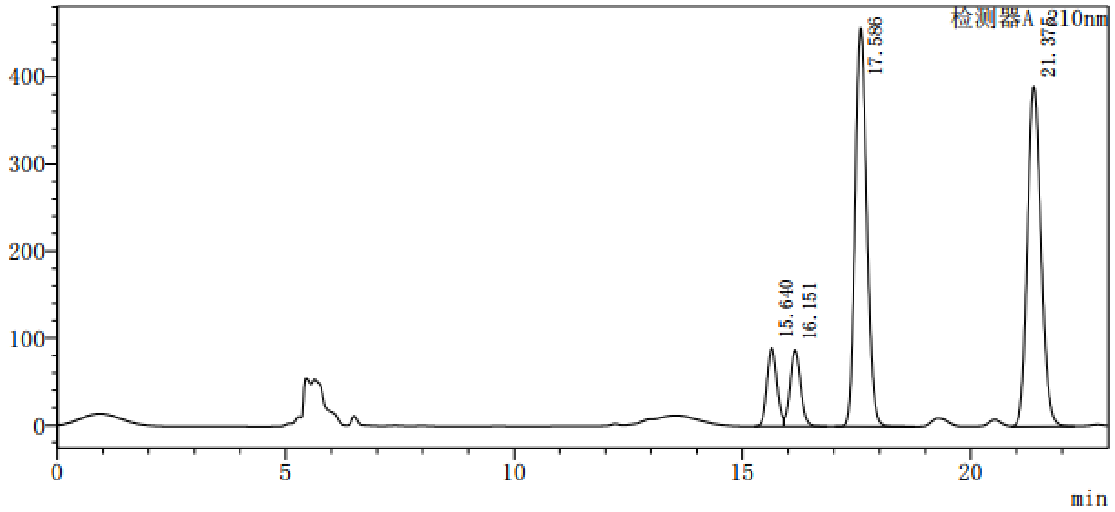

Chiral

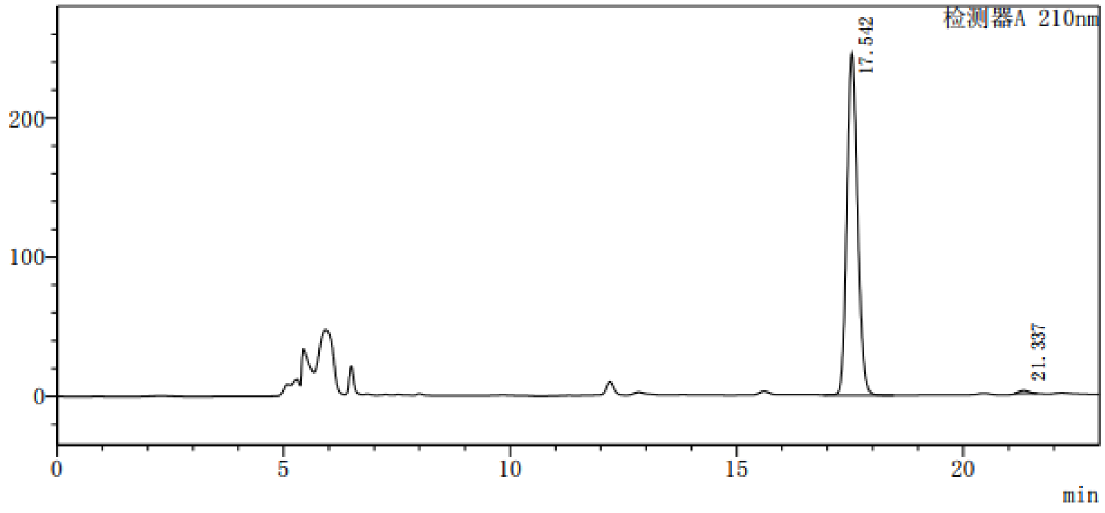

	Retention Time (min)	Area (%)	
**Peak 1**	17.542	99.066	98% ee
**Peak 2**	21.337	0.934	

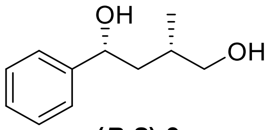

(*R*,*S*)-9

HPLC conditions: Chiralcel OC Column, *n*-hexane/*i*-PrOH = 97/3, 210 nm, 0.8 mL/min, t_{major} = 61.398 min, t_{minor} = 56.211 min.

Racemate

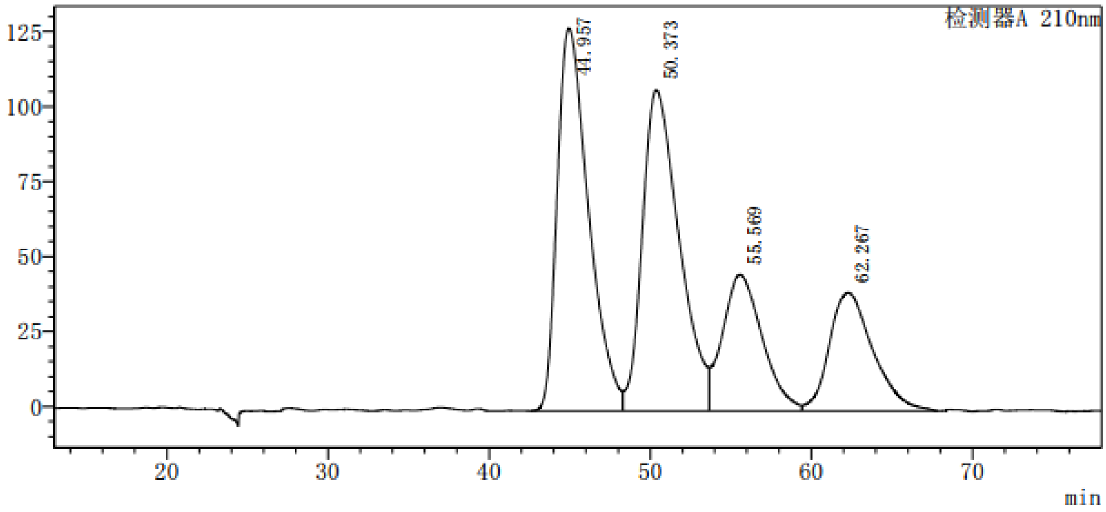

Chiral

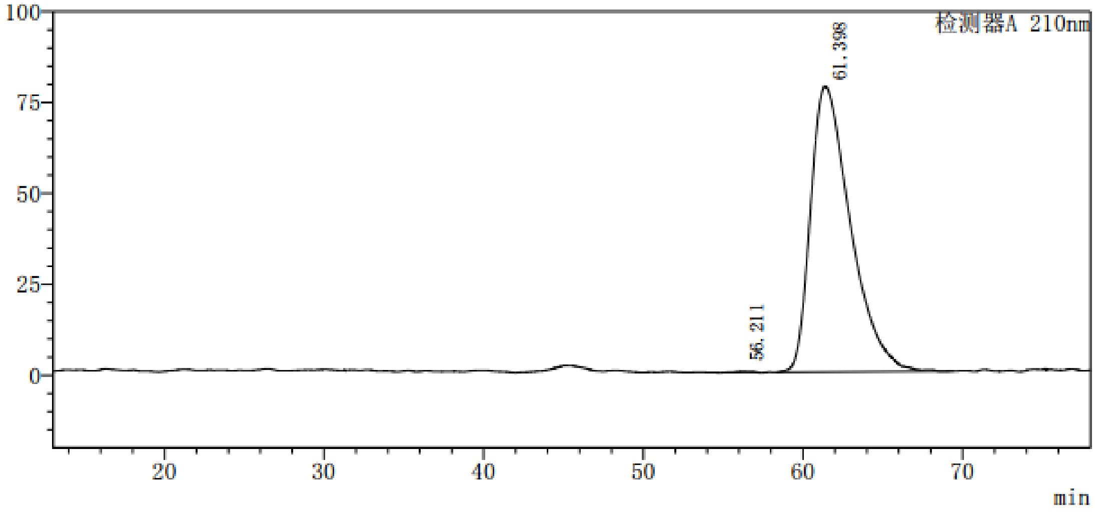

	Retention Time (min)	Area (%)	
**Peak 1**	56.211	0.088	>99% ee
**Peak 2**	61.398	99.912	

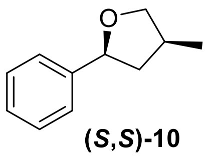

(*S*,*S*)-10

HPLC conditions: Chiralcel OD Column, *n*-hexane/*i*-PrOH = 98/2, 210 nm, 0.4 mL/min, t_{major} = 40.279 min, t_{minor} = 25.903 min.

Racemate

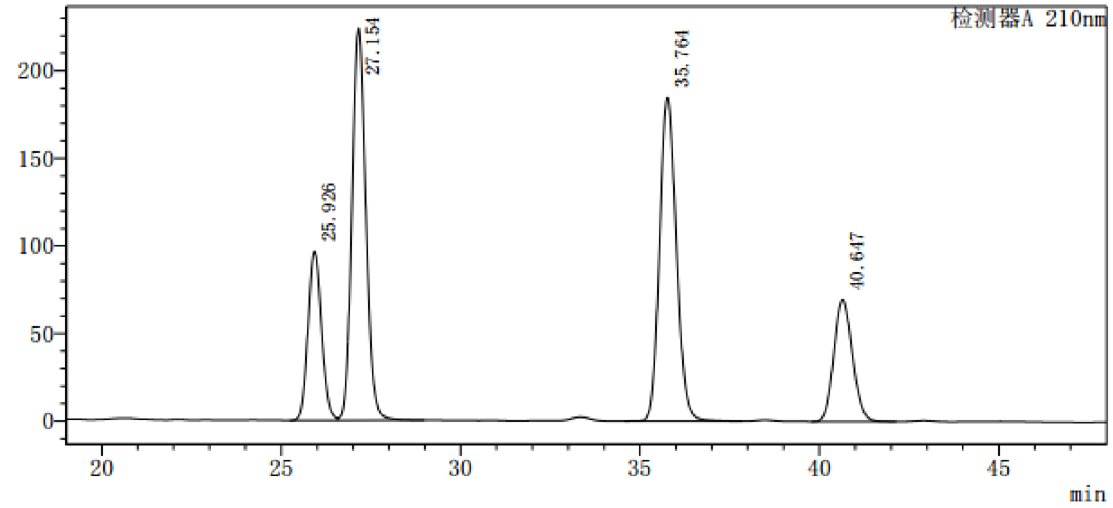

Chiral

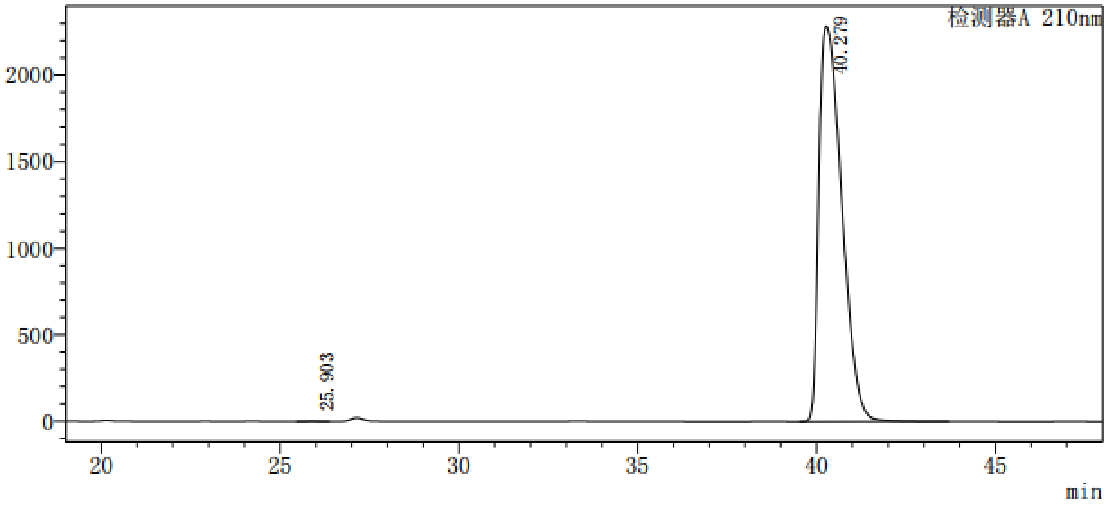

	Retention Time (min)	Area (%)	
**Peak 1**	25.903	0.023	>99% ee
**Peak 2**	40.279	99.977	

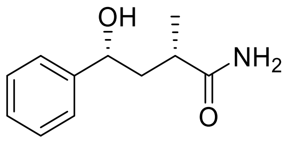

(*S*,*R*)-11

HPLC conditions: Chiralpak AS Column, *n*-hexane/*i*-PrOH = 85/15, 210 nm, 1.0 mL/min, t_{major} = 40.566 min, t_{minor} = 24.071 min.

Racemate

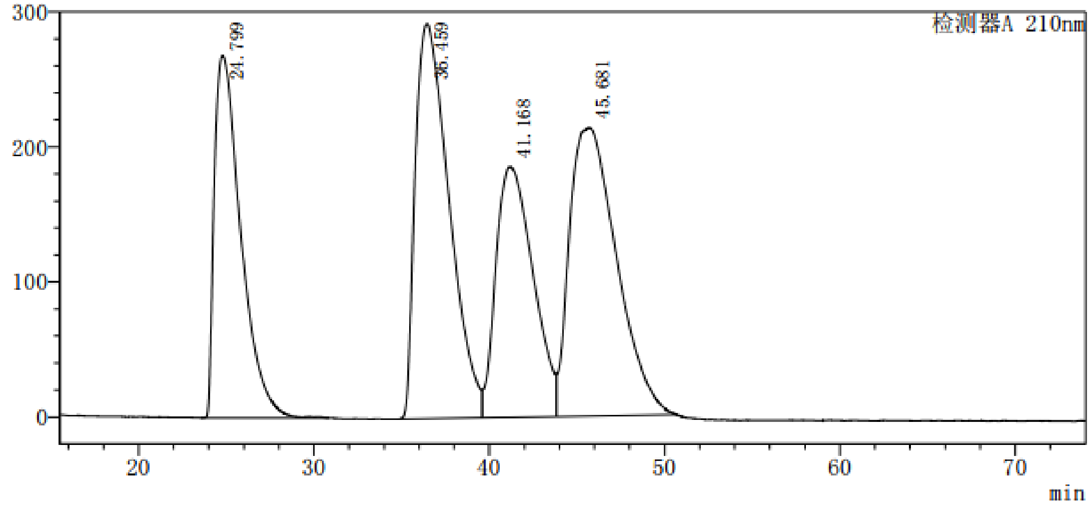

Chiral

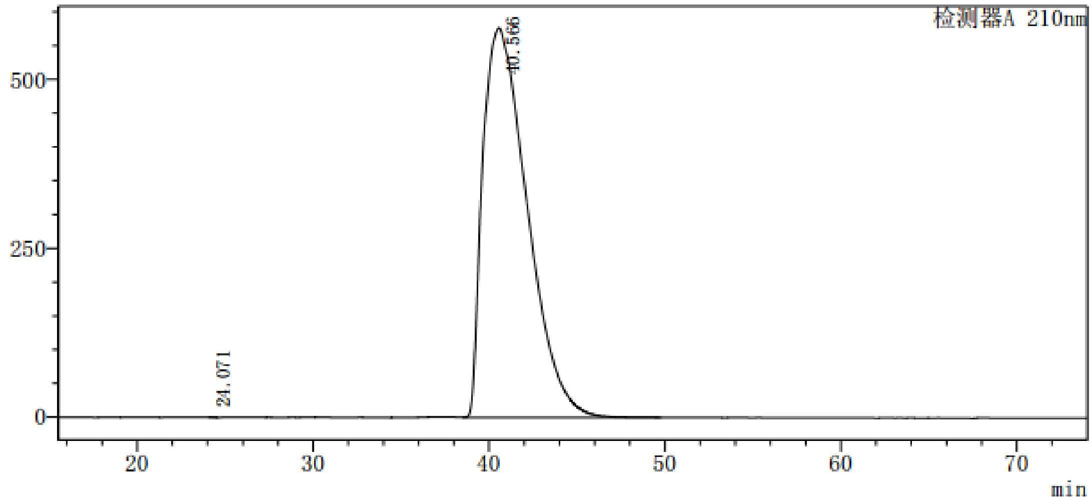

	Retention Time (min)	Area (%)	
**Peak 1**	24.071	0.004	>99% ee
**Peak 2**	40.566	99.996	

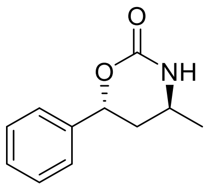

(*S*,*R*)-12

HPLC conditions: Chiralpak AS Column, *n*-hexane/*i*-PrOH = 75/25, 210 nm, 1.0 mL/min, t_{major} = 45.102 min, t_{minor} = 76.683 min.

Racemate

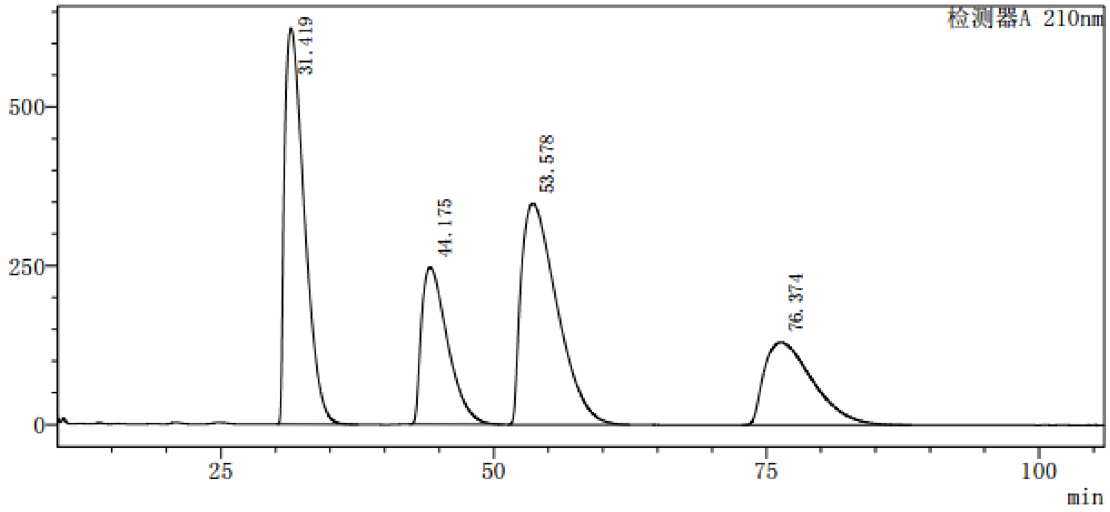

Chiral

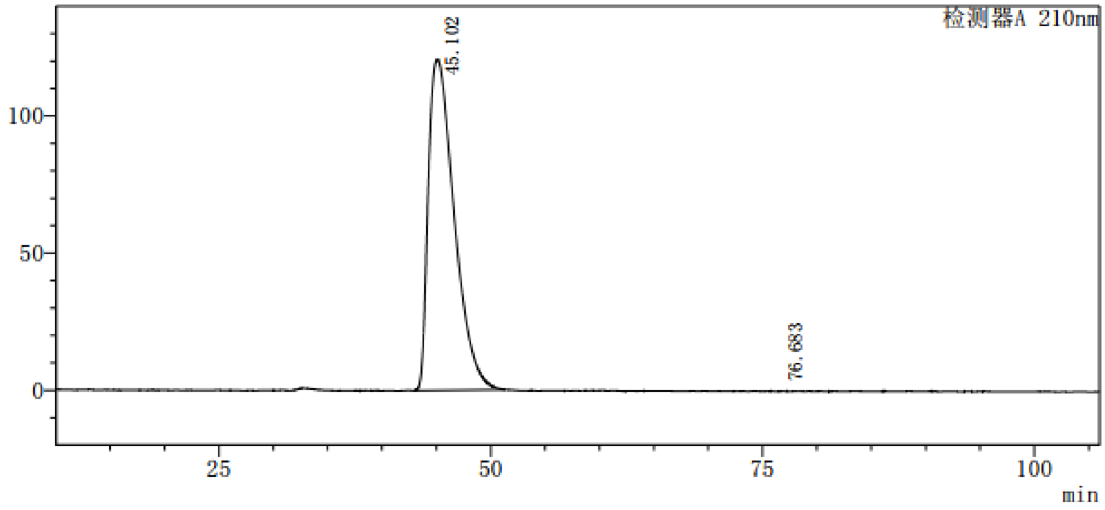

	Retention Time (min)	Area (%)	
**Peak 1**	45.102	99.998	>99% ee
**Peak 2**	76.683	0.002	

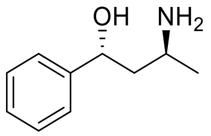

(*R*,*S*)-13

HPLC conditions: Chiralcel OZ Column, *n*-hexane/*i*-PrOH = 90/10, 210 nm, 1.0 mL/min, t_{major} = 18.525 min, t_{minor} = 14.297 min.

Racemate

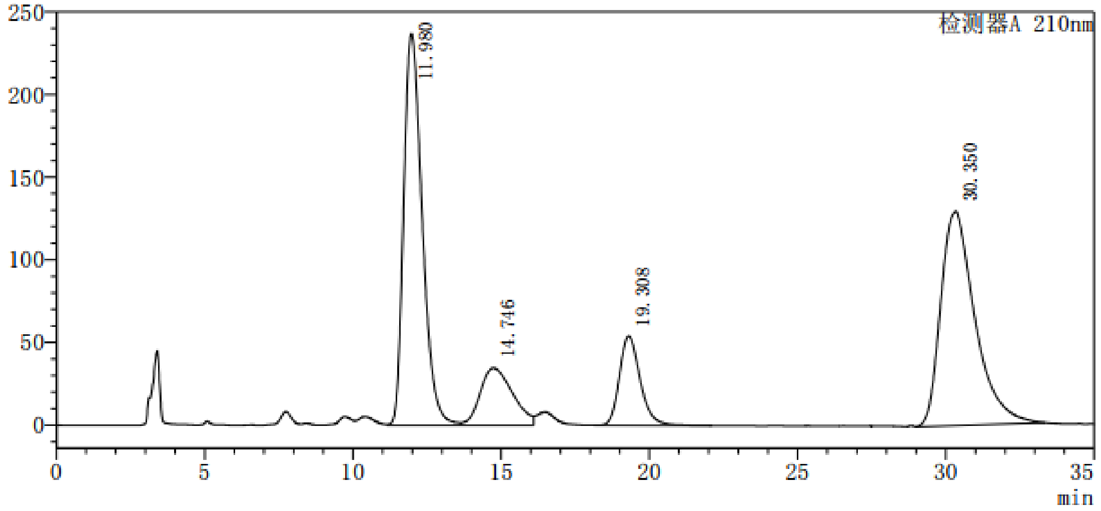

Chiral

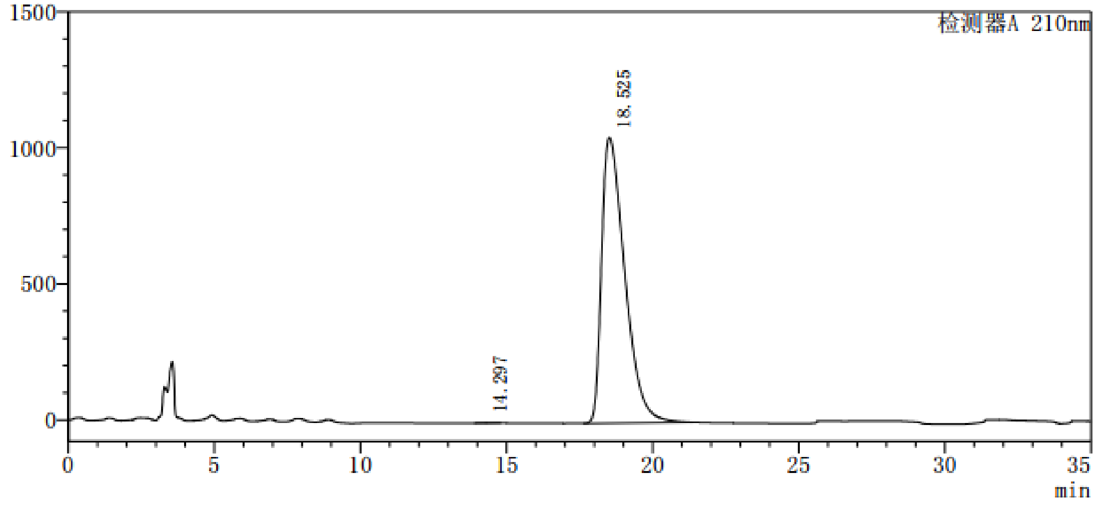

	Retention Time (min)	Area (%)	
**Peak 1**	14.297	0.053	>99% ee
**Peak 2**	18.525	99.947	

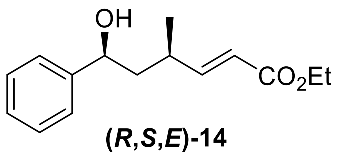

(*R*,*S*,*E*)-14

HPLC conditions: Chiralcel OD Column, *n*-hexane/*i*-PrOH = 96/4, 210 nm, 0.8 mL/min, t_{major} = 16.163 min, t_{minor} = 14.096 min.

Racemate

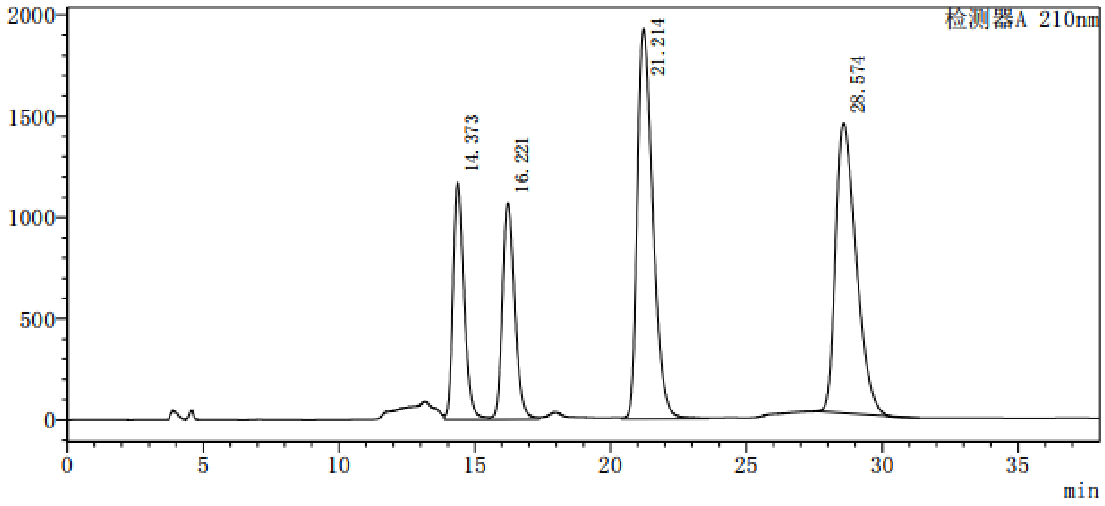

Chiral

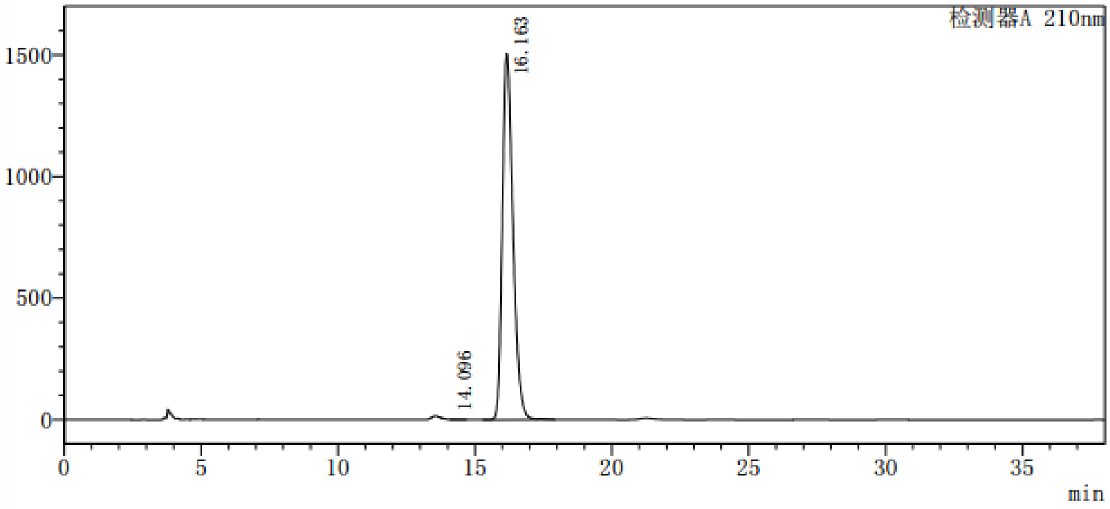

	Retention Time (min)	Area (%)	
**Peak 1**	14.096	0.015	>99% ee
**Peak 2**	16.163	99.985	

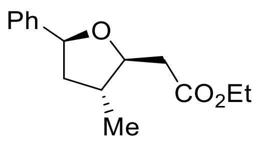

(*S*,*R*,*S*)-15

HPLC conditions: Chiralcel OD-OD Column, *n*-hexane/*i*-PrOH = 98/2, 210 nm, 0.4 mL/min, t_{major} = 37.276 min, t_{minor} = 105.951 min.

Racemate

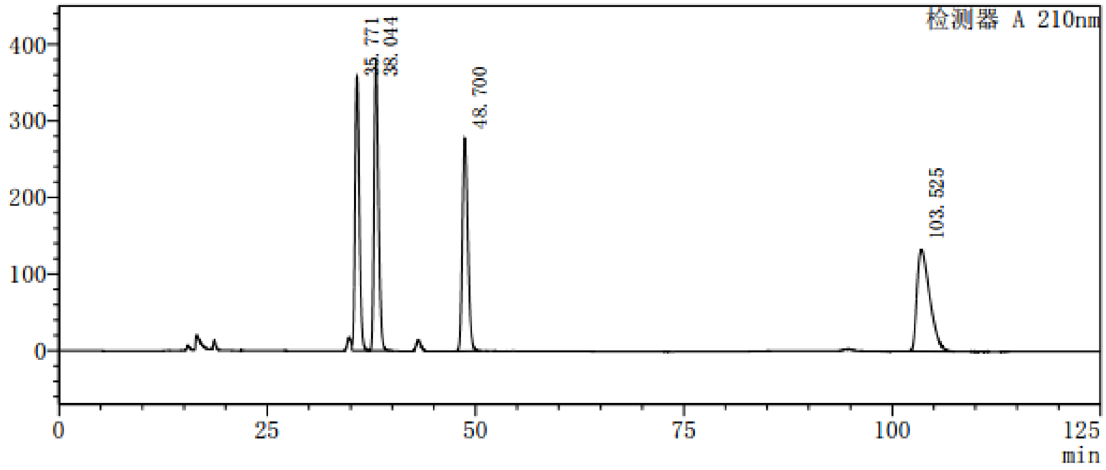

Chiral

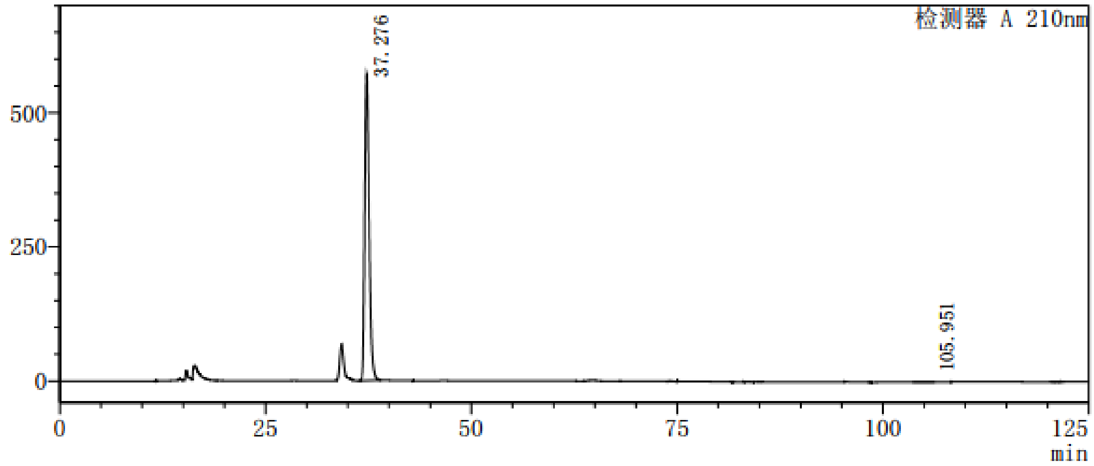

	Retention Time (min)	Area (%)	
**Peak 1**	37.276	99.974	>99% ee
**Peak 2**	105.951	0.026	

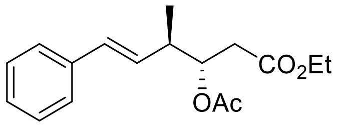

(*S*,*R*,*E*)-16

HPLC conditions: Chiralpak AS Column, *n*-hexane/*i*-PrOH = 95/5, 210 nm, 1.0 mL/min, t_{major} = 5.989 min, t_{minor} = 7.452 min.

Racemate

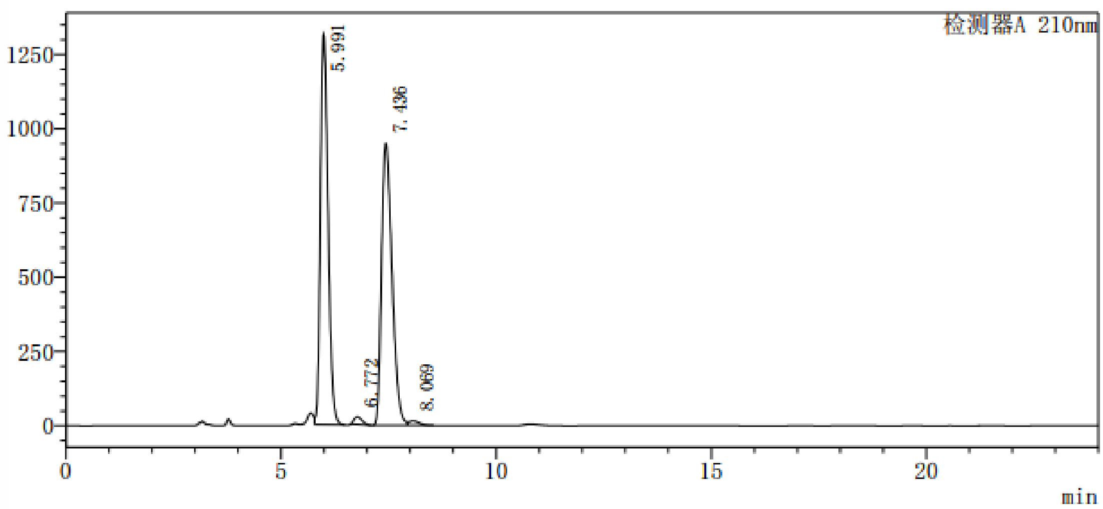

Chiral

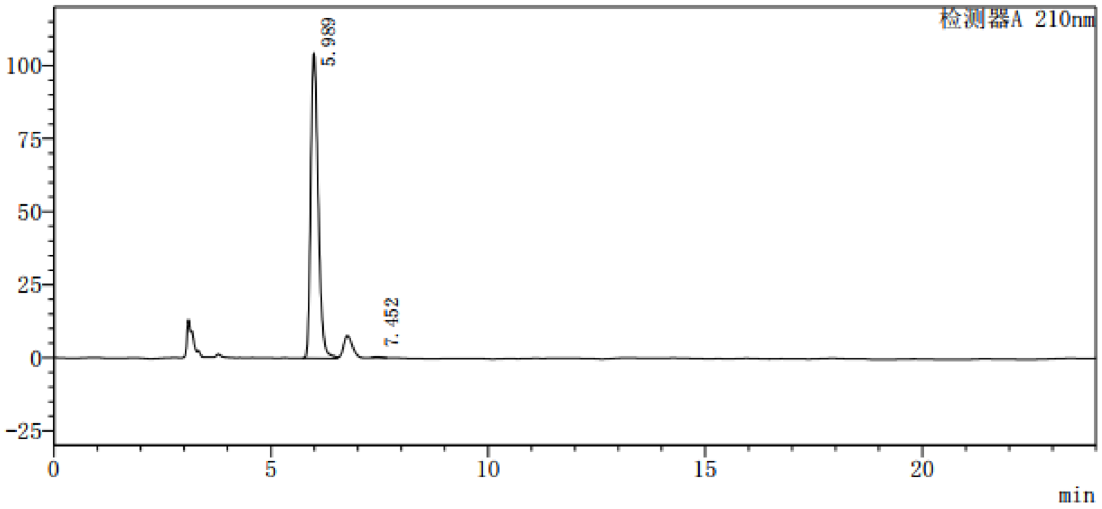

	Retention Time (min)	Area (%)	
**Peak 1**	5.989	99.686	>99% ee
**Peak 2**	7.452	0.314	

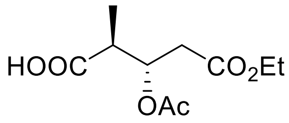

(*S*,*S*)-17

HPLC conditions: Chiralpak AS Column, *n*-hexane/*i*-PrOH = 85/15, 210 nm, 1.0 mL/min, t_{major} = 36.223 min, t_{minor} = 40.620 min.

Racemate

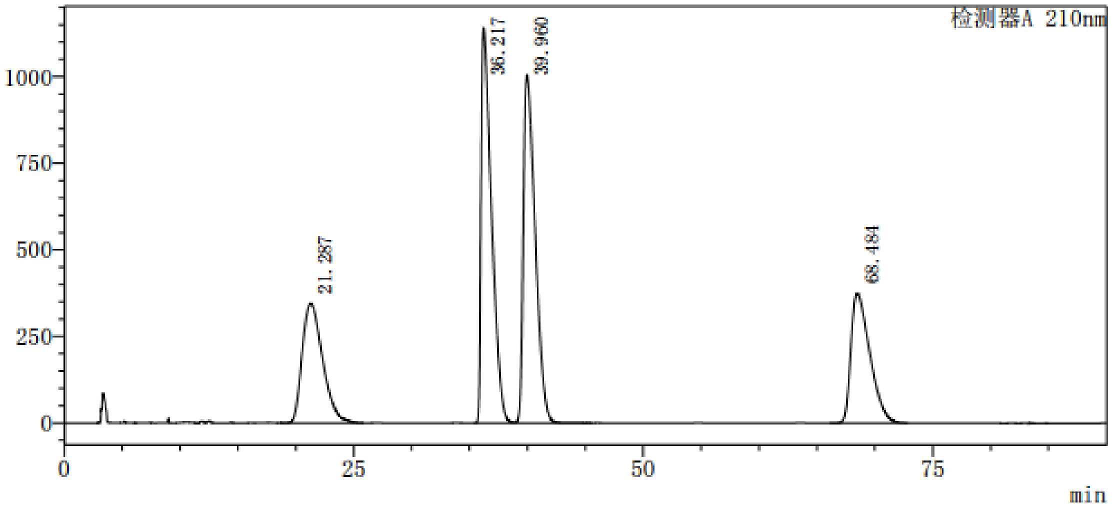

Chiral

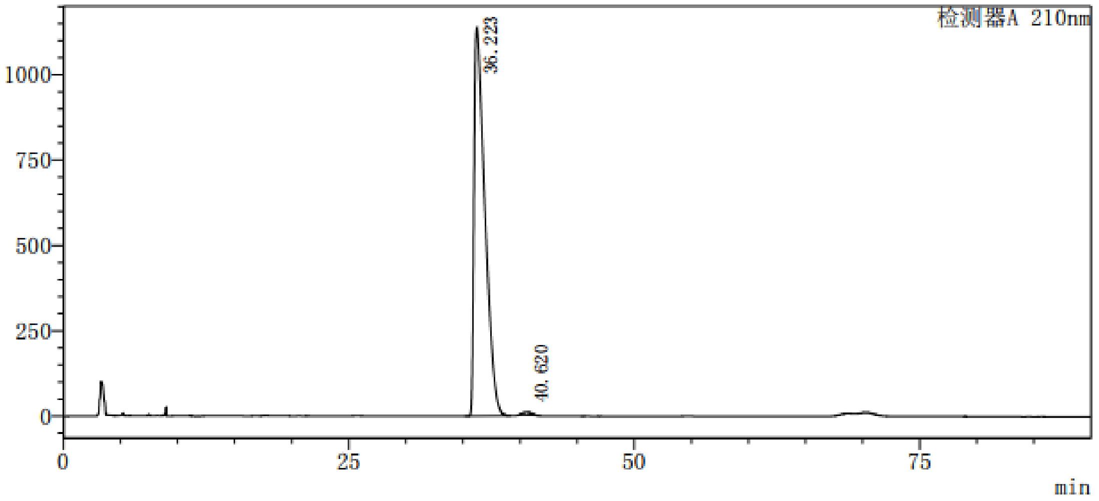

	Retention Time (min)	Area (%)	
**Peak 1**	36.223	99.359	99% ee
**Peak 2**	40.620	0.641	

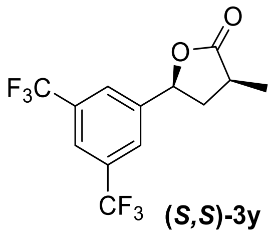

(*S*,*S*)-3y

HPLC conditions: Chiralcel OZ Column, *n*-hexane/*i*-PrOH = 98/2, 210 nm, 0.6 mL/min, t_{major} = 23.962 min, t_{minor} = 20.852 min.

Racemate

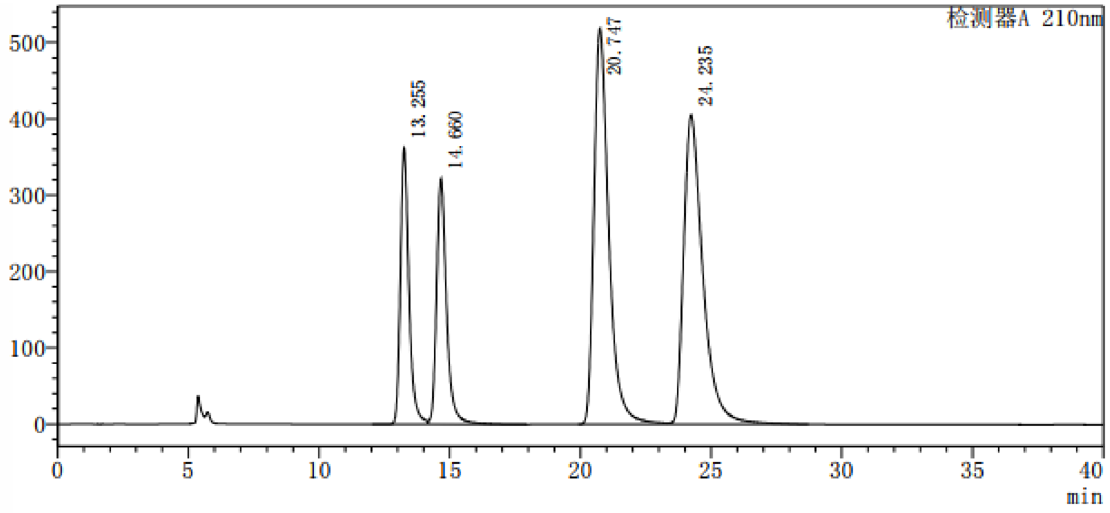

Chiral

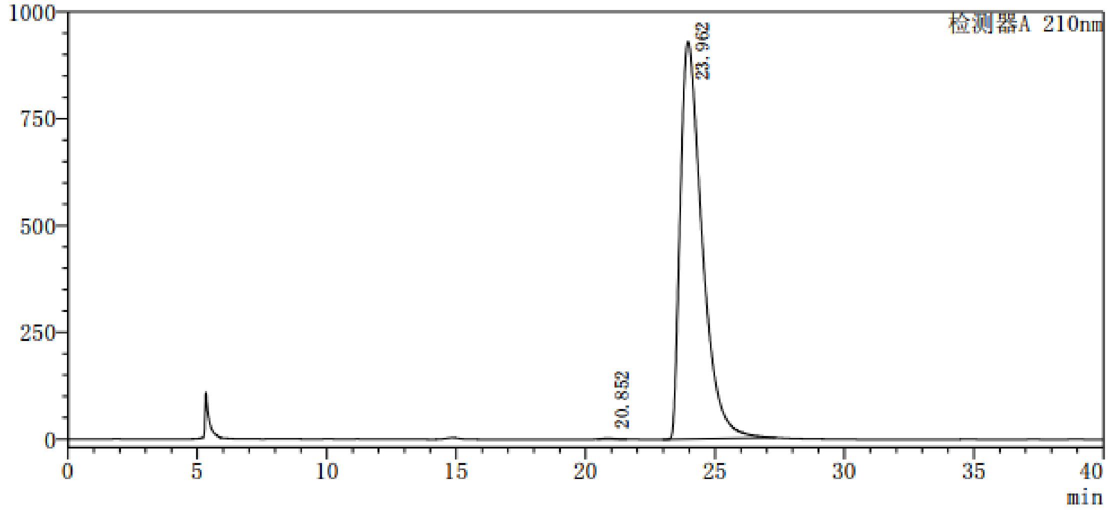

	Retention Time (min)	Area (%)	
**Peak 1**	20.852	0.122	>99% ee
**Peak 2**	23.962	99.878	

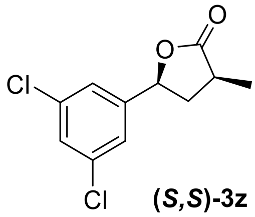

HPLC conditions: Chiralcel OD Column, *n*-hexane/*i*-PrOH = 99/1, 210 nm, 0.3 mL/min, t_{major} = 72.933 min, t_{minor} = 65.790 min.

Racemate

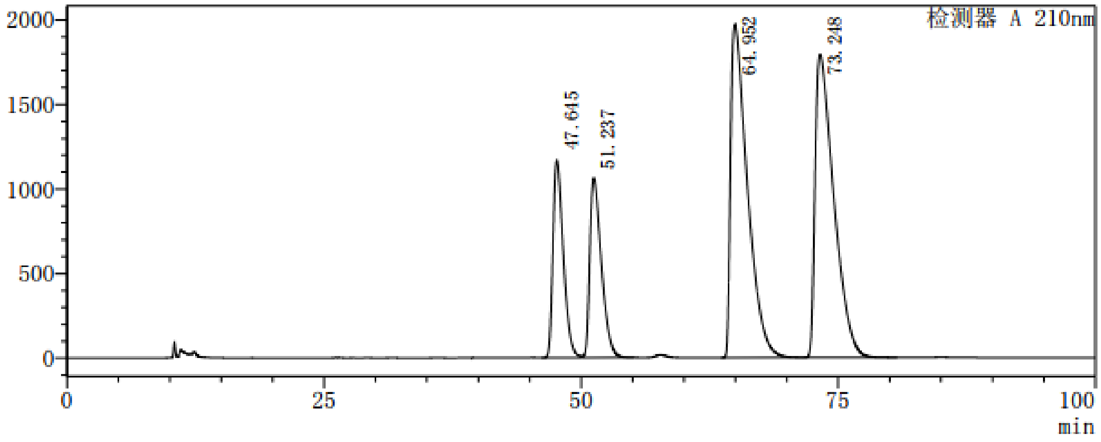

Chiral

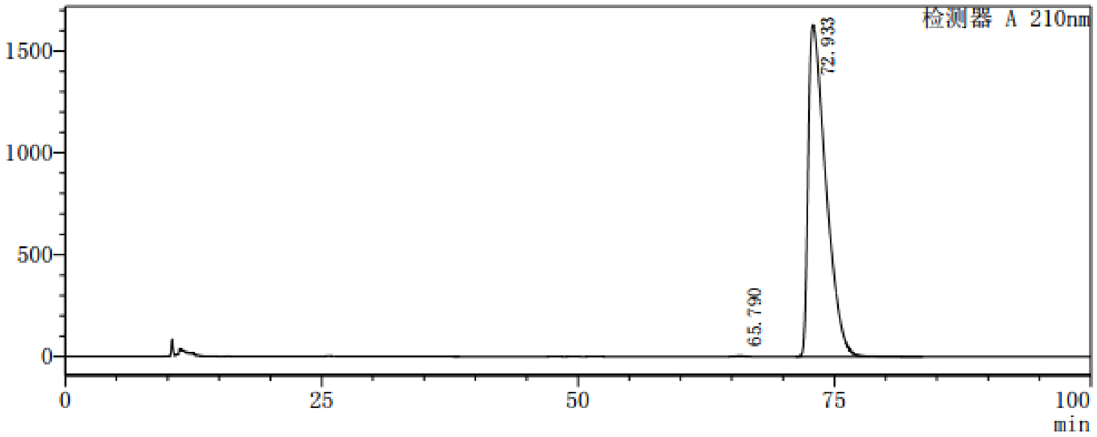

	Retention Time (min)	Area (%)	
**Peak 1**	65.790	0.067	>99% ee
**Peak 2**	72.933	99.933	

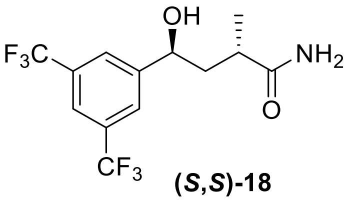

(*S*,*S*)-18

HPLC conditions: Chiralpak AS Column, *n*-hexane/*i*-PrOH = 85/15, 210 nm, 1.0 mL/min, t_{major} = 11.717 min, t_{minor} = 7.206 min.

Racemate

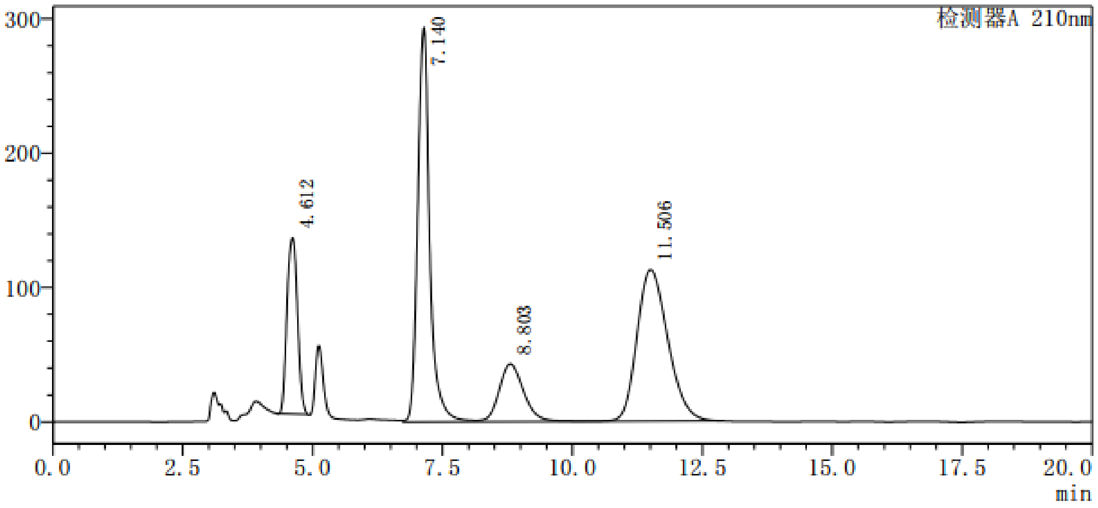

Chiral

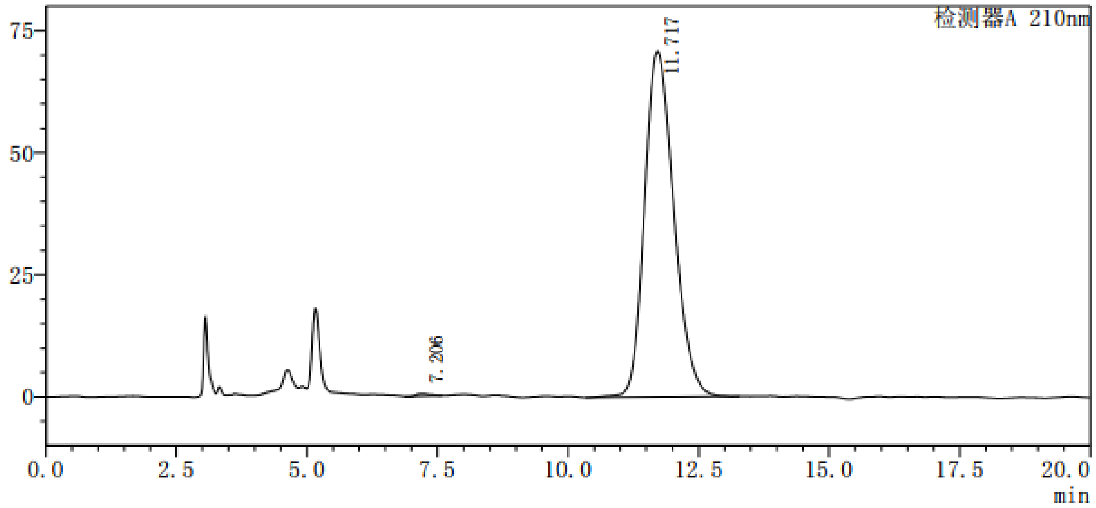

	Retention Time (min)	Area (%)	
**Peak 1**	7.206	0.285	>99% ee
**Peak 2**	11.717	99.715	

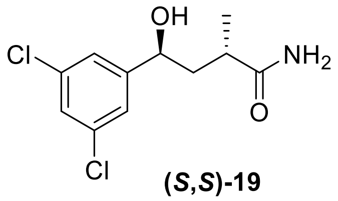

(*S*,*S*)-19

HPLC conditions: Chiralpak AS Column, *n*-hexane/*i*-PrOH = 85/15, 210 nm, 1.0 mL/min, t_{major} = 36.636 min, t_{minor} = 24.347 min.

Racemate

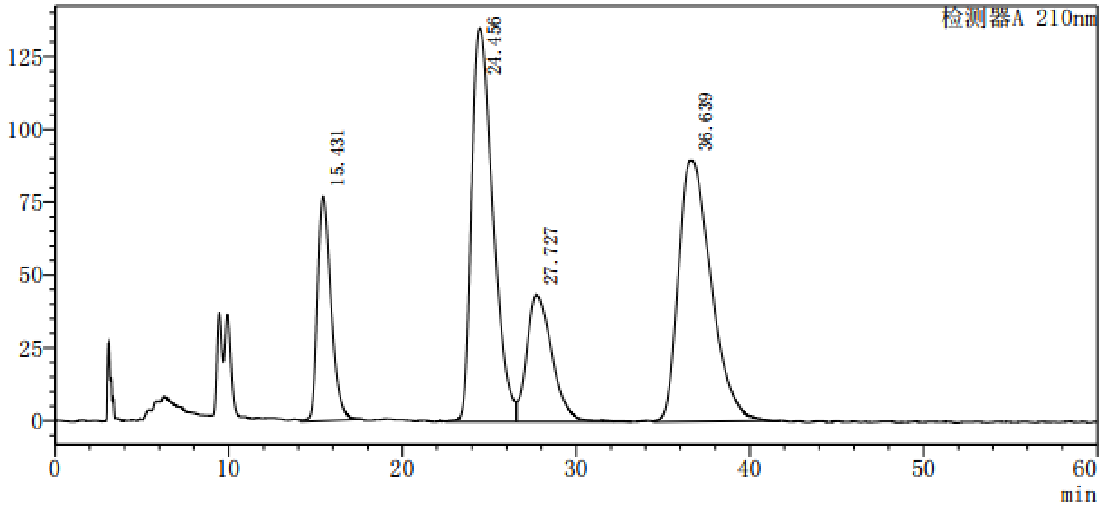

Chiral

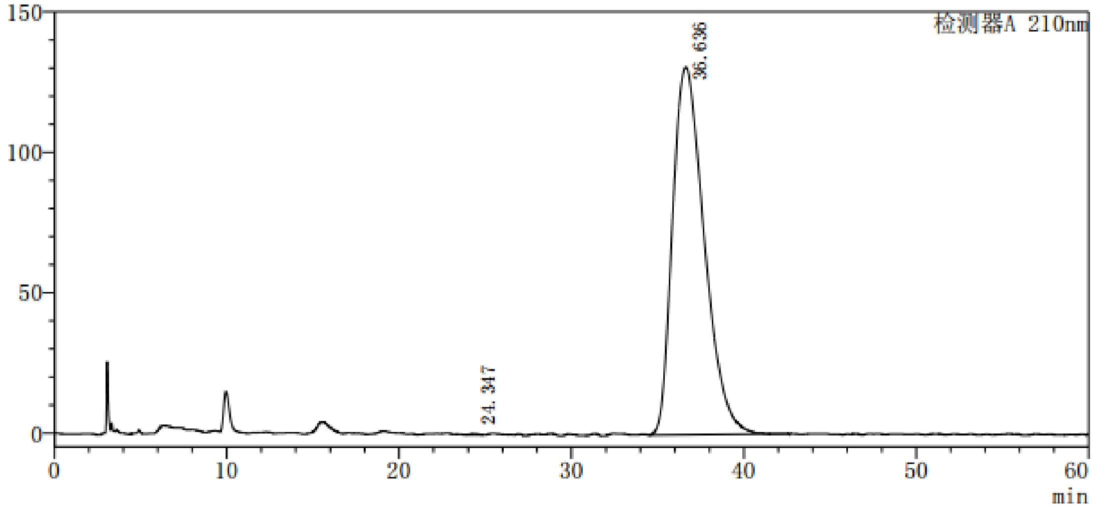

	Retention Time (min)	Area (%)	
**Peak 1**	24.347	0.076	>99% ee
**Peak 2**	36.636	99.924	

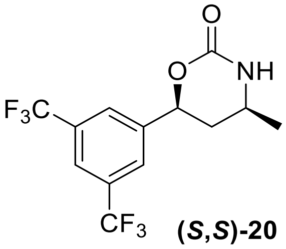

(*S*,*S*)-**20**

HPLC conditions: Chiralpak IC Column, *n*-hexane/*i*-PrOH = 87/13, 210 nm, 1.0 mL/min, t_{major} = 17.448 min, t_{minor} = 30.446 min.

Racemate

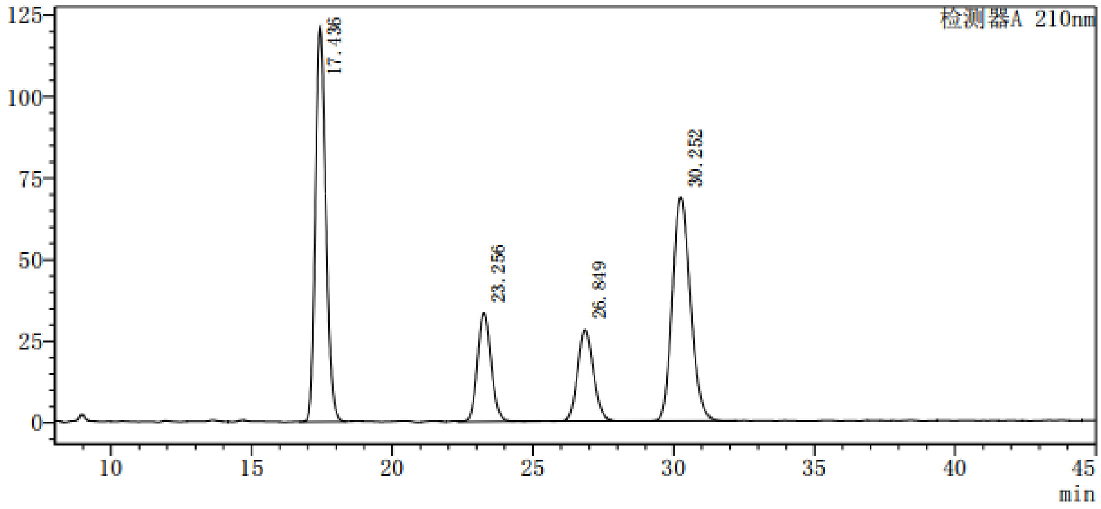

Chiral

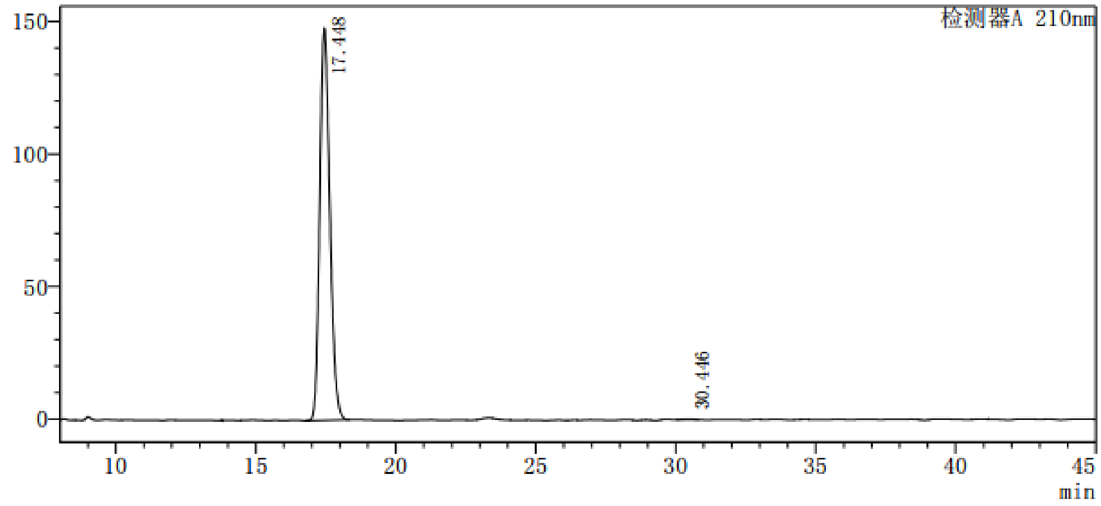

	Retention Time (min)	Area (%)	
**Peak 1**	17.448	99.780	>99% ee
**Peak 2**	30.446	0.220	

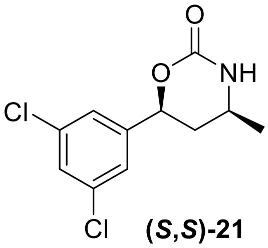

(*S*,*S*)-21

HPLC conditions: Chiralpak AS Column, *n*-hexane/*i*-PrOH = 80/20, 210 nm, 1.0 mL/min, t_{major} = 40.590 min, t_{minor} = 25.173 min.

Racemate

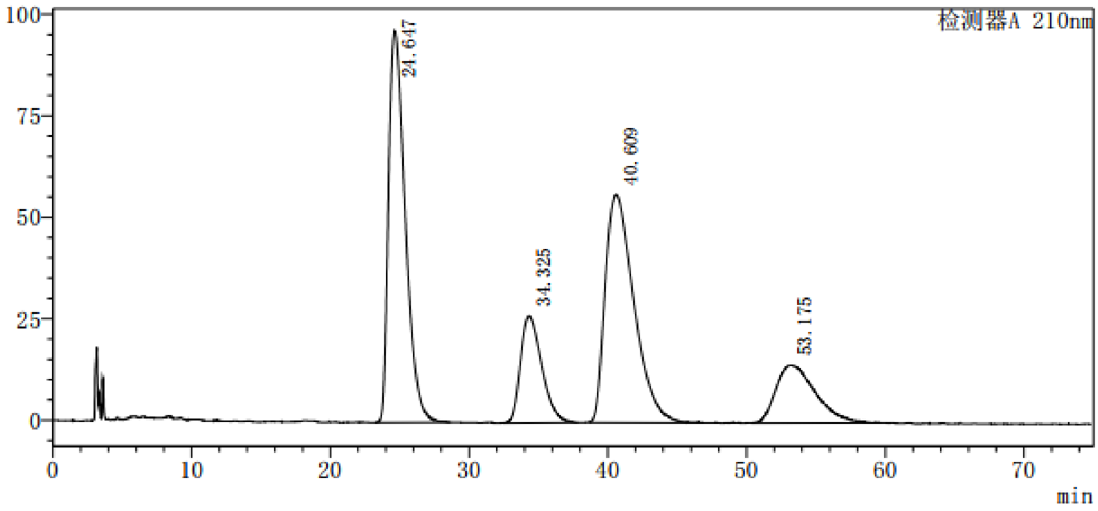

Chiral

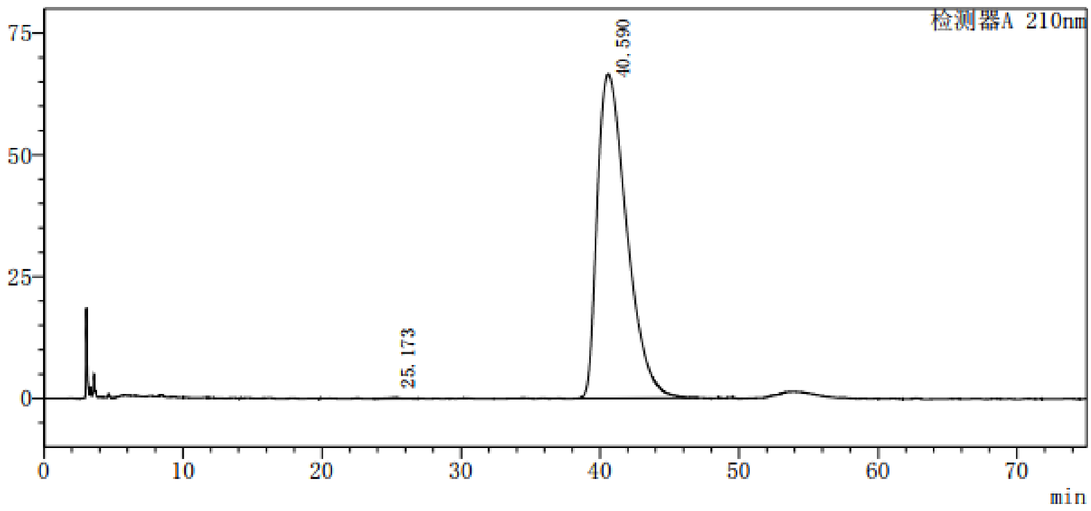

	Retention Time (min)	Area (%)	
**Peak 1**	25.173	0.115	>99% ee
**Peak 2**	40.590	99.885	

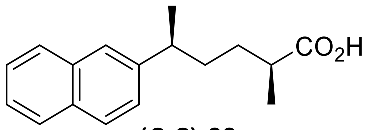

(*S*,*S*)-22

HPLC conditions: Chiralpak IC Column, *n*-hexane/*i*-PrOH = 90/10, 210 nm, 1.0 mL/min, t_{major} = 14.269 min, t_{minor} = 21.248 min.

Racemate

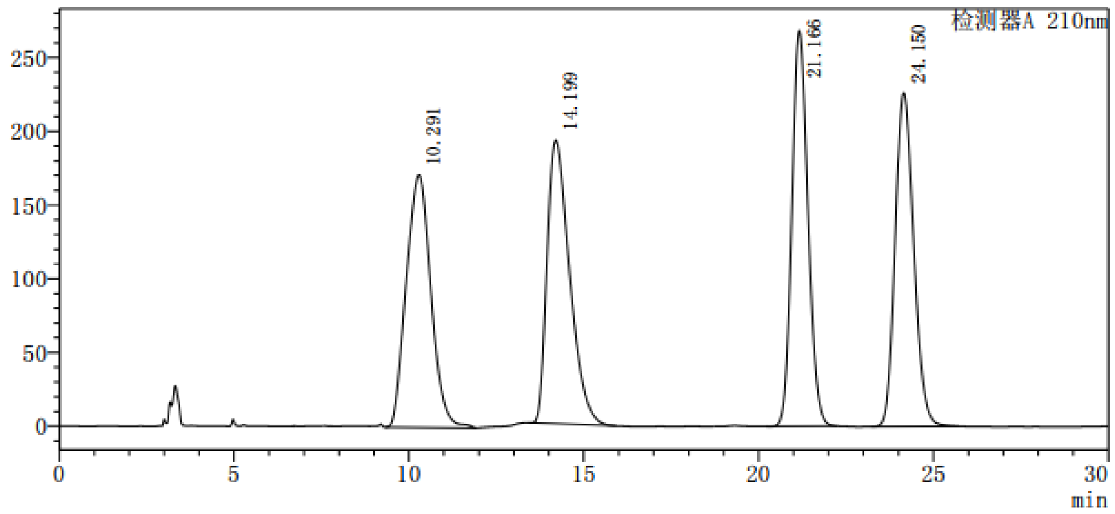

Chiral

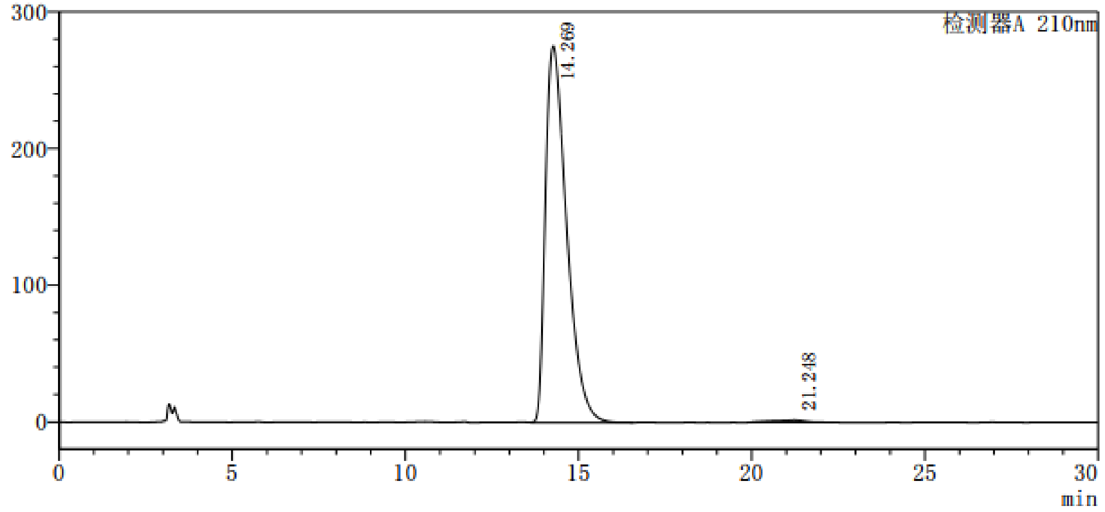

	Retention Time (min)	Area (%)	
**Peak 1**	14.269	99.263	99% ee
**Peak 2**	21.248	0.737	

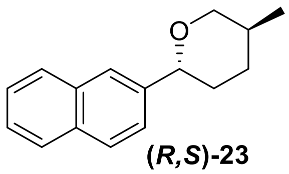

(*R*,*S*)-23

HPLC conditions: Chiralpak IC-IC Column, *n*-hexane/*i*-PrOH = 99/1, 220 nm, 0.2 mL/min, t_{major} = 83.786 min, t_{minor} = 175.951 min.

Racemate

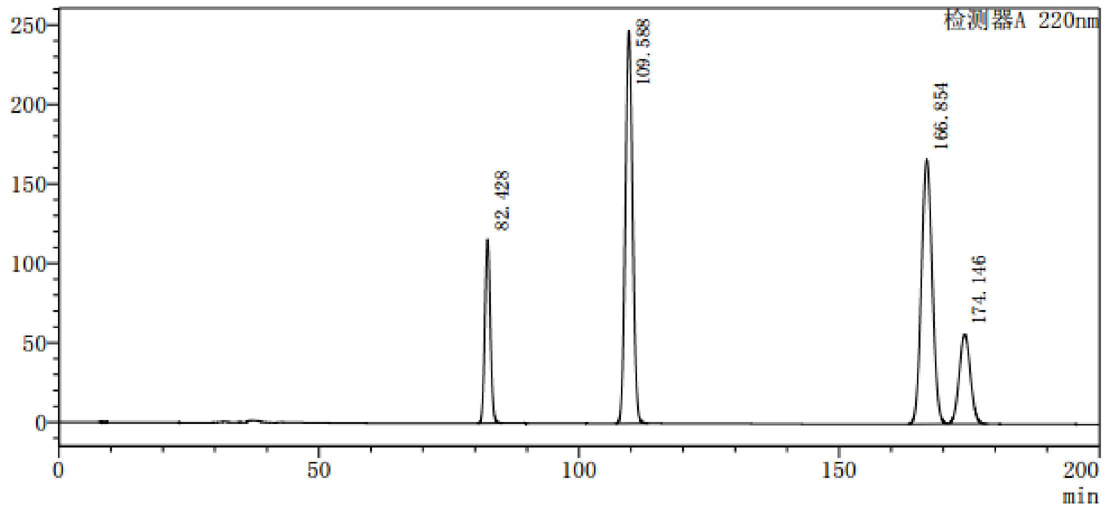

Chiral

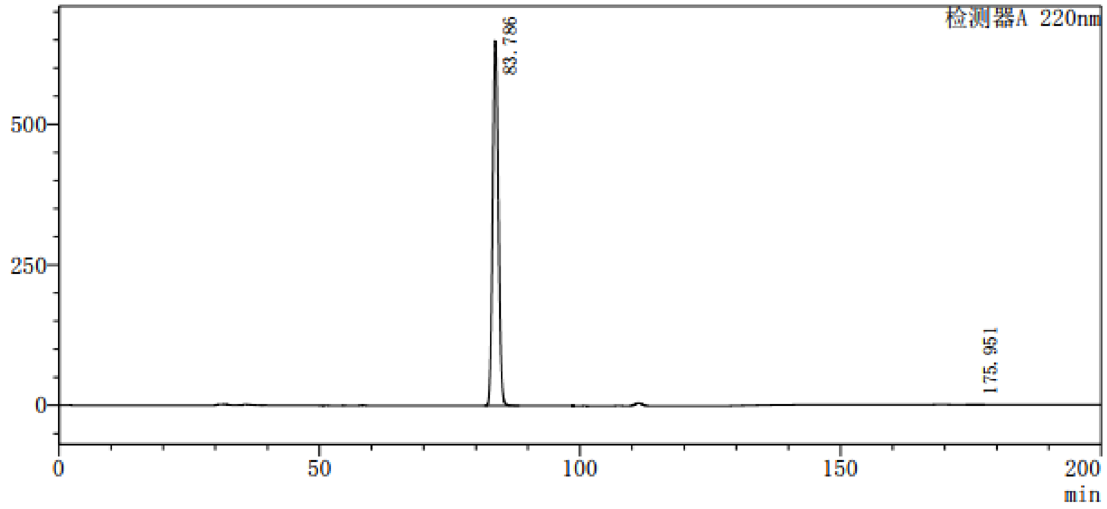

	Retention Time (min)	Area (%)	
**Peak 1**	83.786	99.992	>99% ee
**Peak 2**	175.951	0.008	

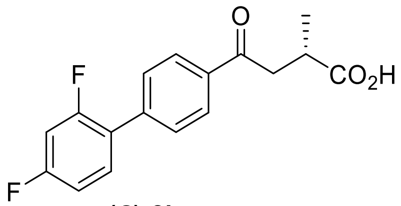

(*S*)-2h

HPLC conditions: Chiralcel OJ Column, *n*-hexane/*i*-PrOH = 95/5, 254 nm, 1.0 mL/min, t_{major} = 38.879 min, t_{minor} = 44.262 min.

Racemate

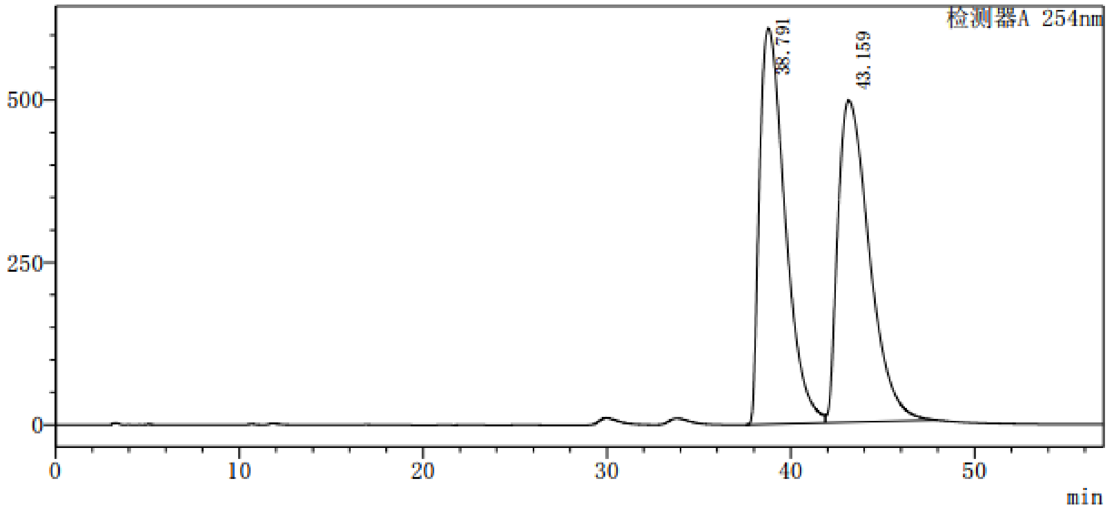

Chiral

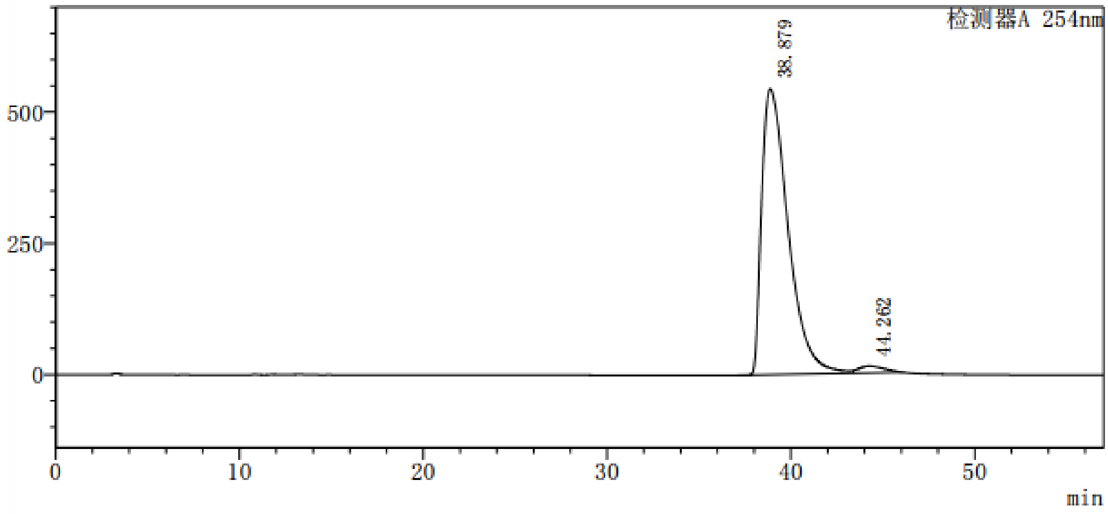

	Retention Time (min)	Area (%)	
**Peak 1**	38.879	97.804	96% ee
**Peak 2**	44.262	2.196	
